# Overexpression of an *NF-YC2* gene confers alkali tolerance to transgenic alfalfa (*Medicago sativa* L.)

**DOI:** 10.3389/fpls.2022.960160

**Published:** 2022-08-05

**Authors:** Jinqiu Yu, Yuying Yuan, Wenkai Zhang, Tingting Song, Xiangyin Hou, Lingzelai Kong, Guowen Cui

**Affiliations:** Department of Grassland Science, College of Animal Science and Technology, Northeast Agricultural University, Harbin, China

**Keywords:** *Medicago sativa*, NF-YC transcription factor, alkali tolerance, physiological index, transgenic alfalfa

## Abstract

Alkaline stress severely limits plant growth and yield worldwide. NF-YC transcription factors (TFs) respond to abiotic stress by activating gene expression. However, the biological function of NF-YC TFs in alfalfa (*Medicago sativa* L.) is not clear. In our study, an *NF-YC2* gene was identified and transgenic plants were obtained by constructing overexpression vector and cotyledon node transformation system in alfalfa. The open reading frame of *MsNF-YC2* is 879 bp with 32.4 kDa molecular mass. *MsNF-YC2* showed tissue expression specificity and was induced by a variety of abiotic stresses including drought, salt, and alkali stress in alfalfa. Under alkali stress treatment, transgenic plants exhibited higher levels of antioxidant enzyme activities and proline (Pro), correlating with a lower levels of hydrogen peroxide (H_2_O_2_), superoxide anion (O_2_^–^) compared with wild-type (WT) plants. Transcriptomic results showed that overexpression of *MsNF-YC2* regulated the expression of phytohormone signal transduction and photosynthesis-related genes under normal and alkaline stress treatments. These results suggest that the *MsNF-YC2* gene plays crucial role enhance alkali adaptation abilities in alfalfa.

## Introduction

Plant growth and development are always limited by various types of abiotic stresses, mainly including alkali, salinity, drought, and high temperature, etc. (Zhao et al., 2019). A total of 438 million hectares of land worldwide are affected by alkali stress, which severely affects the yield and quality of crops (Li et al., 2020). Alkali stress is well-recognized as a highly important environmental factor that severely threatens growth, development and yield of plant to a certain extent. The main manifestations of alkali stress on plant growth include damage to the root structure, reduced water content and root vitality, and reduced photosynthetic pigment synthesis (Guo et al., 2010; Jiao et al., 2019; Liu et al., 2019). Alkali stress is a great threat to crop production, but few studies have investigated the molecular regulatory mechanism of plants under alkali stress.

Nuclear factor Y (NF-Y) TFs are types of TFs widely distributed in eukaryotes. It is also known as CCAAT-binding factors (CBFs) or heme activator proteins (HAPs) and consists of NF-YA, NF-YB, and NF-YC subunits that constitute heterotrimeric capable of binding CCAAT boxes through high affinity and sequence specificity (Zhao et al., 2016; Bi et al., 2017; Wang et al., 2019). Research shows that NF-Y family members may work together or alone in their ability to interact with and coordinate with other TFs with respect to gene expression regulation (Benatti et al., 2008; Yamamoto et al., 2009). The size of NF-YC peptides is generally between that of the NF-YA and NF-YB peptides, and NF-YC peptides contain a conserved histone fold motifs (HFMs) domain whose structure is similar to that of H_2_A proteins, which involved in protein–protein and protein–DNA interactions (Nardone et al., 2017). This domain contains 3 α-helices (called α1, α2, and α3) separated by two β-chain loop domains, with lengths of 7, 27, and 8 amino acids, respectively (Dolfini et al., 2012).

Previous research has shown NF-YC transcription factors (TFs) interact with the promoters of defense-related genes; thus, these TFs ultimately participate in plant growth and development (Cho and Choi, 2009). *AtNF-YC2* was the first NF-YC family member isolated from plants, and its overexpression can increase the transcription of flowering locust T (FT) and accelerate the flowering process (Hackenberg et al., 2012). *AtNF-YC3*, *AtNF-YC4*, and *AtNF-YC9* combine with *AtNF-YB2* and *AtNF-YB3* to form dimers and participate in the CO-mediated photoperiod flowering pathway (Hwang et al., 2019). Subsequent reports have confirmed that CO regulates its expression by binding to the promoter of FT, but the presence of NF-Y and CCAAT boxes can enhance the activity of CO-regulated genes (Kumimoto et al., 2010; Tiwari et al., 2010). In addition to the regulation of flowering, *NF-YC* genes play a role in the development of pollen tubes. Overexpression of *PwNF-YC* promotes the development of pollen tubes, and the effect of *PwNF-YC* is promoted by Ca^2+^ in *Picea wilsonii* (Yu et al., 2011; Qu et al., 2015). Transcription of the *CdtNF-YC1* gene in response to salt stress requires the participation of hydrogen peroxide (H_2_O_2_), nitrous oxide (NO), and abscisic acid (ABA) (Chen et al., 2015). A new *OsNF-YC13* gene has revealed from the rice activation mutant DS-16 is significantly up-regulated under salt stress treatment (Manimaran et al., 2017). Recently, researchers screened two *HvNF-Y* genes from the barley genome, *HvNF-YA1* and *HvNF-YA6*, which responded significantly to salt stress. *AtNF-YC1* transgenic lines can enhance freezing tolerance in *Arabidopsis*, and the freezing tolerance of nf-yc1 mutants decreases, indicating that the *NF-YC1* gene positively regulates the freezing response of that species (Shi and Chan, 2014).

Alfalfa (*Medicago sativa* L.) is an economically and ecologically important legume herbaceous species, good palatability and high yield and effective in soil remediation and improvement (Gréard et al., 2018; Cui et al., 2019). As the alfalfa whole genome sequencing data becomes public, we have an opportunity to systematically investigate mechanisms at the molecular level in alfalfa (Chen et al., 2020). Many reports have been conducted on the responses to plant development process and abiotic stress tolerance of *NFYC* in various species such as *Arabidopsis*, *P. wilsonii*, rice and barley. However, few studies have been reported to explore the functions and diferentially expressed genes (DEGs)/pathways through analysis of transcriptome under alkali stresses in alfalfa.

In our previous study, a transcription factor *NFYC2* was identified from the transcriptome data, which is associated with abiotic stress, especially alkaline stress in alfalfa. We speculated that NFYC transcription factors also have similar alkali tolerance functions in alfalfa. Therefore, molecular mechanism of *NF-YC2*-mediated alkali responses was investigated in our study. Transgenic alfalfa overexpressing *NF-YC2* exhibited enhanced alkali tolerance at all stages of growth. We also identified downstream target genes within the *NF-YC2*-mediated alkali tolerance pathway; these genes are involved in hormone signal transduction and photosynthesis. Comprehensive transcriptome analysis also suggested that *NF-YC2* regulated transcript levels of 26 overlapping genes, which enhanced the alkali tolerance in alfalfa. The obtained information provides more profound insights into the alfalfa molecular mechanism in improving breeding strategies for the development of transgenic plants, which can better resist the alkaline stress.

## Materials and methods

### Isolation and bioinformatic analysis of the *NF-YC2* genomic sequence

RNA extraction kits were used to extract total RNA from alfalfa leaves by the instructions (ComWin, Beijing, China). The synthesis of the first strand of cDNA is also performed according to the instructions (Vazyme, Nanjing, China). As shown in Supplementary Table 1, *NFYC2* specific primers based on the cDNA sequence was designed using primer 5.0 software. The polymerase chain reaction (PCR) mixture comprised 10 μl of Easy Taq Buffer, 1.8 μl of DNTPs, 0.2 μl of Easy Taq DNA Polymerase, 0.5 μl each of forward and reverse primers (10 μM), 1 μl of cDNA (1,000 ng/μl) and 6 μl of ddH_2_O, for a total of 20 μl. The amplified fragments were ligated into pMD18-T vector and then sequenced. We used ExPASy website to analyze the molecular mass and isoelectric point of *NF-YC2* gene.^1^ DNAMAN and MEGA software were used to analyze the multiple sequence alignment and to construct a phylogenetic tree with 1,000 bootstrap replicates of NF-YCs proteins, respectively (Yuan et al., 2022).

### Analysis of *MsNF-YC2* gene expression pattern

The expression level of *NF-YC2* was measured by RT-PCR in alfalfa. The extraction of cDNA was performed as described above. The design methods of *NF-YC2* RT-PCR and internal reference gene (*GAPDH* gene) were the same as above (Supplementary Table 1). The PCR mixture consisted of 10 μl of Top Green qPCR Super Mix (Transgen Biotech Co., Ltd., Beijing, China), 1 μl of cDNA (100 ng/μl), 0.5 μl each of forward and reverse primers (10 μM), and sterile water, for total volume of 20 μl. The PCR amplification program was as follows: 98°C for 10 s; 40 cycles of 95°C for 5 s, 60°C for 34 s, and 95°C for 15 s; 60°C for 1 min; and then 95°C for 15 s (Haddad and Baldwin, 2010). Relative gene expression levels were evaluated according to the Kameda 2^–ΔΔCt^ method (Ish-Shalom and Lichter, 2010). The analysis of each sample included three biological replicates.

### Generation of *NF-YC2* overexpressing transgenic plants

The coding DNA sequence (CDS) of *NF-YC2* was amplified and subcloned into a pMDC123 vector and driven by the 35S promoter of cauliflower mosaic virus (Supplementary Figure 1). The plasmids of constructed overexpression vector were introduced into *Agrobacterium tumefaciens* strain LBA4404, and then the cotyledon node transformation system was used to complete the genetic transformation of alfalfa (Sun et al., 2016). Semi-quantitative and quantitative RT–PCR were used to identify positive transgene plants (Ning et al., 2017). Three transgenic lines (OV#L1, OV#L2, and OV#L3) were used for further stress treatments.

### Alkali stress treatment and determination of the physiological indexes of transgenic plants

Transgenic plants were propagated using the asexual cutting propagation method. WT alfalfa and transgenic lines were planted in the same plastic pots of 10 cm diameter, 8 cm high with a 5 mm diameter hole. The pots were filled with a mixture of stuffed vermiculite and nutrient soil (1:1) and placed in a greenhouse incubator for growth. The pots of alfalfa plants were placed in trays (56 cm × 78 cm, 30 pots per tray) and watered with Hoagland nutrient solution. After 1 month of growth, plants with uniform growth were selected for alkali stress treatment. For the control group, each tray received Hoagland nutrient solution such that 200 mL was provided to each pot. For the alkali stress treatment group, 150 mmol/L NaHCO_3_ (with a pH of 8.50) was used instead of Hoagland nutrient solution for 5 day. The leaf fresh weight and relative water content were measured after 5 day of alkali treatment (Naus et al., 2016). Three biological replications were included for each line. Likewise, physiological indicators were determined in transgenic and wild-type plants after 5 day of alkali stress treatment. The superoxide dismutase (SOD) activity, peroxidase (POD) activity, H_2_O_2_ content, O_2_^–^ content, proline (Pro) content, and malondialdehyde (MDA) content were measured by using a physiological index kit (Nanjing Jiancheng Bioengineering, Nanjing, China). The indicators measured were repeated three times, each with three biological replicates in this experiment.

### RNA sequencing and analysis

Fresh leaves were collected from WT plants and transgenic alfalfa lines after 5 day of alkali treatment. RNA and cDNA were extracted and synthesized, respectively, in the same manner as that described above. The cDNA library construction and quality inspection were carried out by Beijing Novo Biotechnology Co., Ltd., with the NEB common library construction method (Illumina Hiseq™ 4,000 sequencing system). Clean data were obtained by filtering raw data, checking sequencing error rate and GC content distribution. Then Trinity was used for transcriptome assembly on the basis of clean data (Grabherr et al., 2011).

The EdgeR package and RSEM package were used to adjust read counts and to identify DEGs, respectively (Anders and Huber, 2010). A unigene was considered to be a significantly DEG when its padj was < 0.05 and its | log2(fold change)| was ≥ 1. The GOseq R package and KOBAS software were performed enrichment analysis of differentially expressed genes (DEGs) in Gene Ontology (GO) and Kyoto Encyclopedia of Genes and Genomes (KEGG) pathways (Xie et al., 2011).^2^ Ten genes that were differentially expressed between *MsNF-YC2* overexpression and wild-type plants were selected to validate transcriptome results.

### Statistical analyses

The statistical analysis of the results was performed by one-way Analysis of Variance (ANOVA) using SPSS 19.0 (SPSS Inc., Chicago, IL, United States), and Duncan’s Multiple Range test was used to detect significant differences between groups. The means were considered to be significantly different at *p* < 0.05.

## Results

### Cloning and sequence analysis of *MsNF-YC2* in alfalfa

**FIGURE 1:**
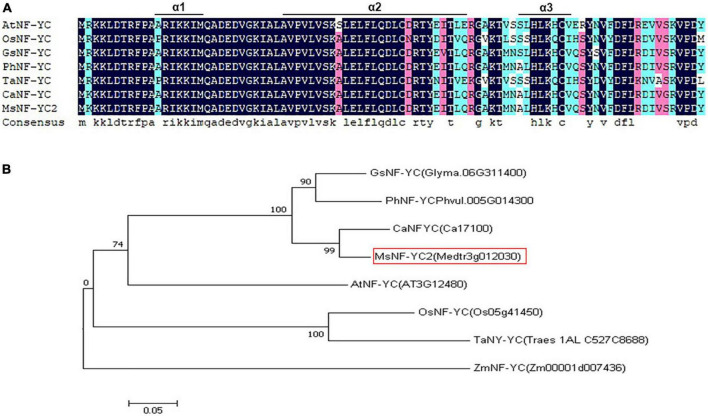
Alignment of sequences and phylogenetic tree of the NFYC proteins. **(A)** Alignment of sequences of the NFYC proteins *MsNFYC2* and homologs from other species. **(B)** Phylogenetic tree of NFYC proteins from alfalfa and other species.

On the basis of previous studies, we isolated a putative *NF-YC-like* gene in the alfalfa genome. cDNA was used a template of full-length sequence amplification to determine the expression level of the gene and to perform PCR in conjunction with primers. The cDNA sequence obtained was referred to as *MsNF-YC2* (MTR_3g012030) and submitted to GenBank of under the sequence accession number MK370092.^3^ The resulting PCR product was approximately 879 bp, which encodes a protein of 292 amino acids with a molecular mass of 32.4 kDa and a pI of 5.12. The PCR product was used for carrier construction (Supplementary Table 2). DNAMAN software was used for sequence alignment, and the alignment results showed that the *MsNF-YC2* protein contained three α-helices (α1, α2, and α3) (Figure 1A). To investigate the evolutionary relationships between alfalfa *MsNF-YC2* and *NF-YCs* from six species (*Arabidopsis thaliana, Oryza sativa, Glycine max, Phaseolus vulgaris, Triticum aestivum* L., and *Cicer arietinum*), a phylogenetic tree of the conserved domains was constructed. *MsNF-YC*2 had the closest genetic relationship with *CaNF-YC* from *C. arietinum* (Figure 1B).

### Analysis of *MsNF-YC2* gene expression

**FIGURE 2:**
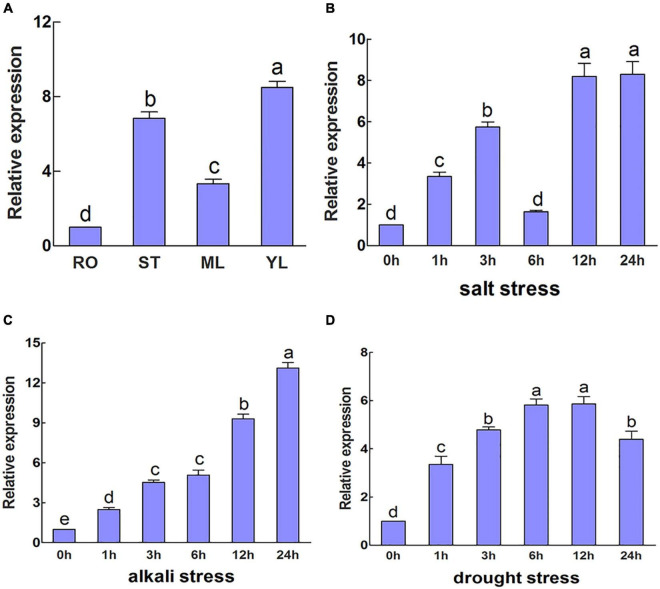
The expression pattern of *MsNF-YC2* in alfalfa. **(A)** Expression levels of *MsNF-YC2* in different tissues. **(B)** Expression levels of *MsNF-YC2* in response to salt treatment. **(C)** Expression levels of *MsNF-YC2* in response to alkali treatment. **(D)** Expression levels of *MsNF-YC2* in response to drought treatment. Each value is the mean ± SE of three biological replicates. Error bars represent the standard deviation of the mean. Significant differences by different letters above the bars at the *P* < 0.05 level according to Duncan’s multiple range test.

The expression of the *MsNF-YC2* gene in four different tissues, i.e., roots, stems, young leaves, and mature leaves, was measured. The results suggested that *MsNF-YC2* was specifically expressed in different tissues, with the transcript being most abundant in the young leaves, followed by the stems, mature leaves and roots (Figure 2A). This result suggested that the *MsNF-YC2* gene is mainly induced and that the transcripts accumulate mainly in young leaves.

To examine whether the expression patterns of *MsNF-YC2* were induced by salt, alkali, and drought stresses, we continually measured the expression in leaves of treated plants for 24 h. The expression levels of the *MsNF-YC2* gene tended to continually fluctuate; the levels increased in the early stage and then decreased, and the level ultimately was greatest at 24 h under salt stress (Figure 2B). Regarding the alkali treatment, *MsNF-YC2* transcripts steadily accumulated at 1 and 24 h and peaked at 24 h (Figure 2C). Moreover, the *MsNF-YC2* transcripts were significantly up-regulated at 1 and 12 h in response to drought treatment, and the level of increase at each time point was similar (Figure 2D). In general, the expression of *MsNF-YC2* was dramatically induced by salt, alkali, and drought stresses treatments.

### Generation of transgenic alfalfa plants overexpressing *MsNF-YC2*

Transgenic alfalfa plants overexpressing *MsNF-YC2* under control of the CaMV 35S promoter were obtained *via* an agrobacterium-mediated cotyledonary node transformation system. Transgenic alfalfa plants overexpressing *MsNF-YC2* were obtained and identified *via* glufosinate selection, and all were found to be positive for the bar gene *via* PCR (Supplementary Figure 2A). The mRNA level of *MsNF-YC2* were further detected by Real-time PCR in transgenic alfalfa lines. The results suggested that the transcript levels of *MsNF-YC2* in the OV#L1, OV#L2, and OV#L3 lines were significantly higher than those in WT lines (Supplementary Figure 2B).

### Overexpression of *MsNF-YC2* significantly increases alkali tolerance in transgenic alfalfa

**FIGURE 3:**
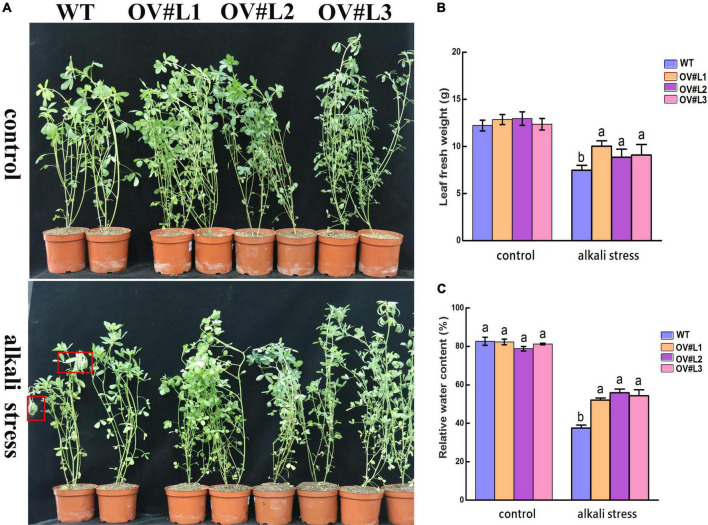
Alkali stress tolerance analysis of *MsNF-YC2* overexpression lines and WT plants. **(A)** Phenotypic analysis of *MsNFYC2* overexpression plants under 5-day alkali stress treatment. **(B)** Relative fresh weight. **(C)** Relative root length. Significant differences by different letters above the bars at the *P* < 0.05 level according to Duncan’s multiple range test.

Our research confirmed that *MsNF-YC2* is induced in response to abiotic stress, especially alkali stress. As such, the alkali tolerance of the *MsNF-YC2* OE lines was further tested. Under 150 mM NaHCO_3_ (pH = 8.5) treatment for 5 days, the three *MsNF-YC2* OE lines (OV#L1, OV#L2, and OV#L3) grew better than the WT plants did, which began to wilt (Figure 3A). After 150 mM NaHCO_3_ treatment, the average fresh weight and leaf relative water content of the *MsNF-YC2*-overexpressing alfalfa plants were significantly higher than those of wild-type plants, while no significant differences were found under 0 mM NaCl and NaHCO_3_ conditions (Figures 3B,C). The alkali tolerance assay indicated that overexpression of *MsNF-YC2* could enhanced the tolerance to alkali stress in alfalfa.

### Physiological analysis of *MsNF-YC2* transgenic lines under alkali stress

**FIGURE 4:**
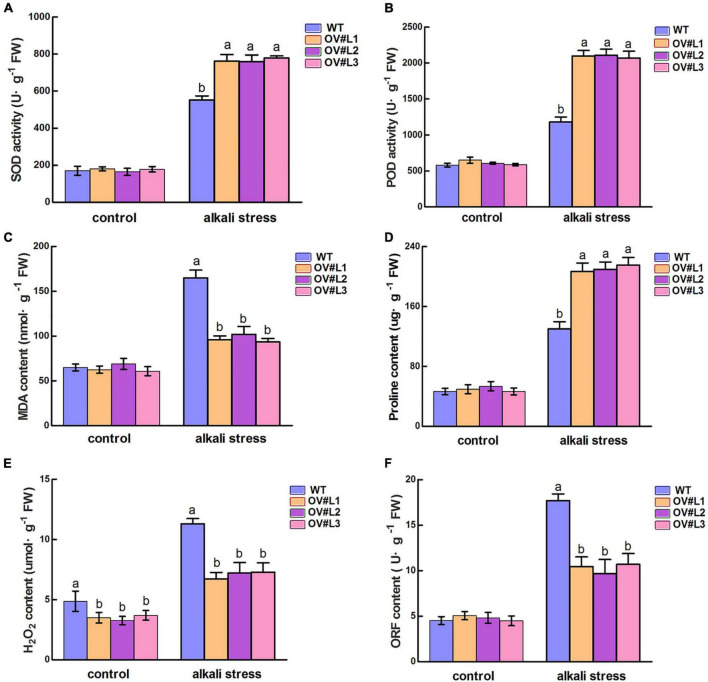
Physiological index analysis of transgenic and wild-type plants under 5-day alkali stress treatment. **(A)** Superoxide dismutase (SOD) activity. **(B)** Peroxidase (POD) activity. **(C)** MDA activity. **(D)** Free proline content. **(E)** H_2_O_2_ content. **(F)** OFR content. Each value is the mean ± SE of three biological replicates. Significant differences by different letters above the bars at the *P* < 0.05 and *P* < 0.01 level according to Duncan’s multiple range test.

The physiological parameters of *MsNF-YC2* OE and WT plants were measured to study the alkali tolerance of transgenic plants. SOD and POD activity were measured to reflect the degree of alkali stress damage in OE and WT plants. As shown in Figures 4A,B, there were no significant differences in SOD or POD activity between the WT and OE lines under normal conditions. After 5 day of alkali stress conditions, however, the SOD and POD activities increased to varying degrees, and there was a more significant increase in the OE plants than in WT plants. Overall, *MsNF-YC2* overexpression plants could enhance the reactive oxygen species (ROS) scavenging ability and reduce the damage to plants under alkaline stress.

Upon exposure to alkali conditions, the MDA content in WT plants was significantly higher than that in transgenic plants (Figure 4C). Proline content was also detected. The results showed that the content of proline was not significantly different between transgenic and wild-type plants under normal conditions, but the level of *MsNF-YC2* overexpression plants was significantly higher than that in wild-type under alkaline stress (Figure 4D). Various environmental stressors can result in the accumulation of large amount of ROS, including H_2_O_2_ and O_2_^–^, in plant cells, resulting in severe damage to proteins, plasma membranes, DNA and other cellular components. Under alkali treatment, the activities in the OE plants were significantly lower than those in the WT plants (Figures 4E,F). In general, these results demonstrated that overexpression of *MsNF-YC2* improved alkali tolerance by increasing the activity of the antioxidant enzymes SOD and POD, increasing the production of Pro and decreasing MDA, H_2_O_2_, and O_2_^–^ levels under alkali stress in alfalfa.

### Transcriptomic comparisons among *NF-YC2* transgenic and wild-type plants under normal conditions or alkali stress

**FIGURE 5:**
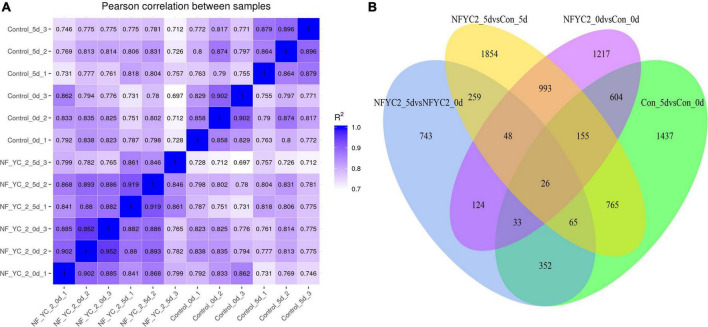
**(A)** Sample correlation analysis of transcriptome data. **(B)** Venn diagram of the DEGs in comparisons in *MsNF-YC2* overexpression and WT plants under normal and alkali stress conditions.

To further explore the molecular mechanism of *NF-YC2* response to alkali stress, total RNA extracted from WT and overexpressing transgenic lines under normal (abbreviated as CN for control-NF-YC2 and CW for control-WT) and alkali stress (AN for alkali-NF-YC2 and AW for alkali-WT) conditions was subjected to RNA sequencing (RNA-seq) analysis. The raw data from transcriptome sequencing have been uploaded to the National Center for Biotechnology Information (NCBI) database with accession number PRJNA795604.^4^ Each condition included three biological replications, sample–sample were highly correlated (Figure 5A). A total of 3,200 DEGs were identified in “CW vs. CN”, with 1,344 genes up-regulated and 1,856 genes down-regulated (Supplementary Figure 3 and Supplementary Table 3). Moreover, a total of 4,165 DEGs were identified in “AW vs. AN”, of which 1,976 genes were up-regulated and 2,189 genes were down-regulated (Supplementary Figure 4 and Supplementary Table 4). Meanwhile, a total of 26 DEGs were identified in four pair-wise comparisons by Venn diagram analysis (Figure 5B). This indicated that *MsNFYC2* may enhance alkali tolerance by regulating these stress -responsive genes, including *MsG0180001988*, *MsG0180000201*, *MsG0180004244*, etc. (Supplementary Table 5). The enriched pathways were significant differences between “CW vs. AW” and “CN vs. AN”. This result suggested that effects of alkali stress and *NF-YC2* overexpression were interacted at the transcriptome level. There are mutual pathways in “CW vs. AW” and “CN vs. AN”, such as the “Plant hormone signal transduction,” “Photosynthesis-antenna proteins,” and “MAPK signaling pathway-plant” Supplementary Figure 5.

**FIGURE 6:**
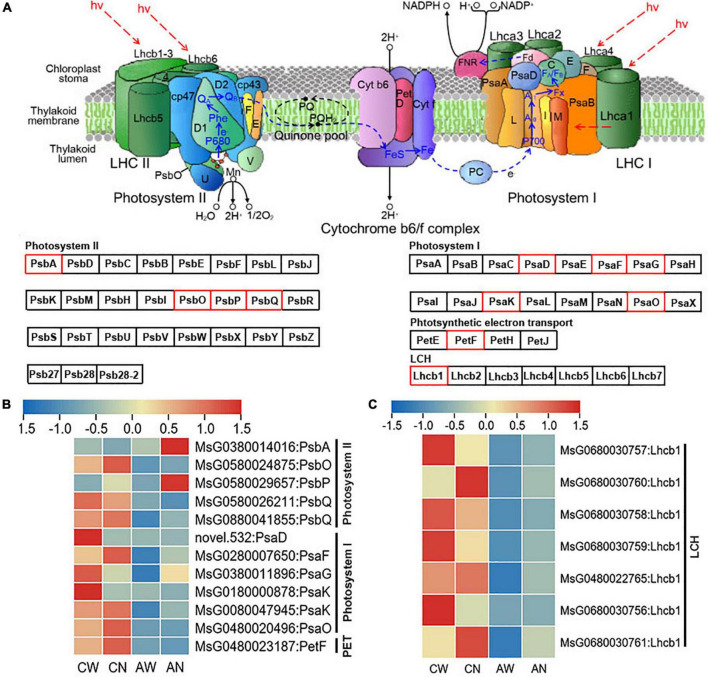
**(A)** Differentially expressed genes involved in photosystem (PSI and PSII), photosynthetic electron transport (PET) process and light harvesting complex (LHC). DEGs are marked in red box. **(B)** Heatmap of DEGs annotated in PET, PSI and PSII according to transcriptome of WT and *MsNF-YC2* overexpression plants. **(C)** Heatmap of DEGs annotated in LHC. Sample abbreviations: under normal abbreviated as CN for control-NF-YC2, and CW for control-WT; under alkali stress abbreviated as AN for alkali-NF-YC2 and AW for alkali-WT.

Genes related to photosynthesis, mainly including photosynthetic electron transport (PET), PSI, PSII, and LHC were greatly affected by the *NF-YC2* overexpression (“CW vs. CN” and “AW vs. AN”) at the transcriptional level (Figure 6A).

Although the DEGs encoding PSI, PSII, PET, and LHC proteins were down-regulated in both *MsNF-YC2* overexpression and wild-type plants under alkaline stress, degrees of changes were lower in transgenic lines than WT plants (Figures 6B,C).

**FIGURE 7:**
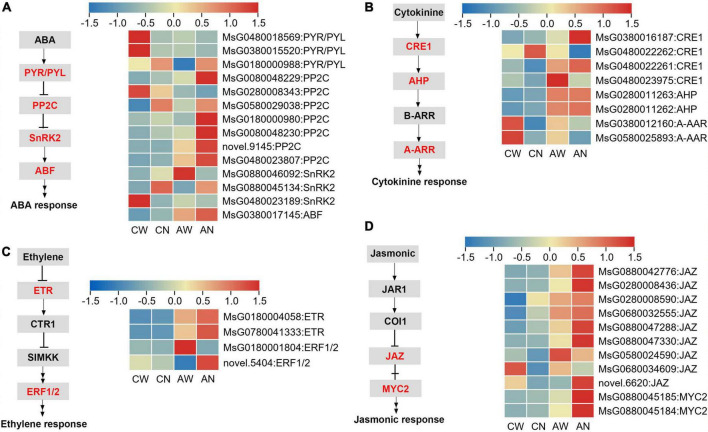
Differentially expressed genes involved in plant hormone signal transduction. **(A)** Abscisic acid (ABA); **(B)** cytokinine; **(C)** ET and **(D)** jasmonic signal transduction pathways.

The expression patterns of DEGs involved in plant hormone signaling pathways were also analyzed. The results showed that signal transduction elements showed altered expression, such as ABA multiple receptors pyrabactin resistance 1/PYR1-like (PYR/PYL), negative regulator protein phosphatases 2C (PP2C), sucrose non-fermenting 1-related protein kinase (SnRK2) and ABRE binding factors (ABF), had higher transcript levels in *NF-YC2* overexpression lines than WT plants under alkali stress (Figure 7A). Meanwhile, in ET, cytokinine and jasmonic signal transduction pathways elements, including ethylene receptor (ETR), ethylene response factor (ERF), cytokinine response elements (CRE), *Arabidopsis* histidine-phospho-transfer proteins (AHP), *Arabidopsis* response regualtors (A-ARR), jasmonate ZIM-domain (Jaz), and myelocytomatosis (Myc) had the same expression pattern as above (Figures 7B–D). These results suggested that signal transduction genes were up-regulated in *NF-YC2* overexpression lines compared to WT under alkali stress.

**FIGURE 8:**
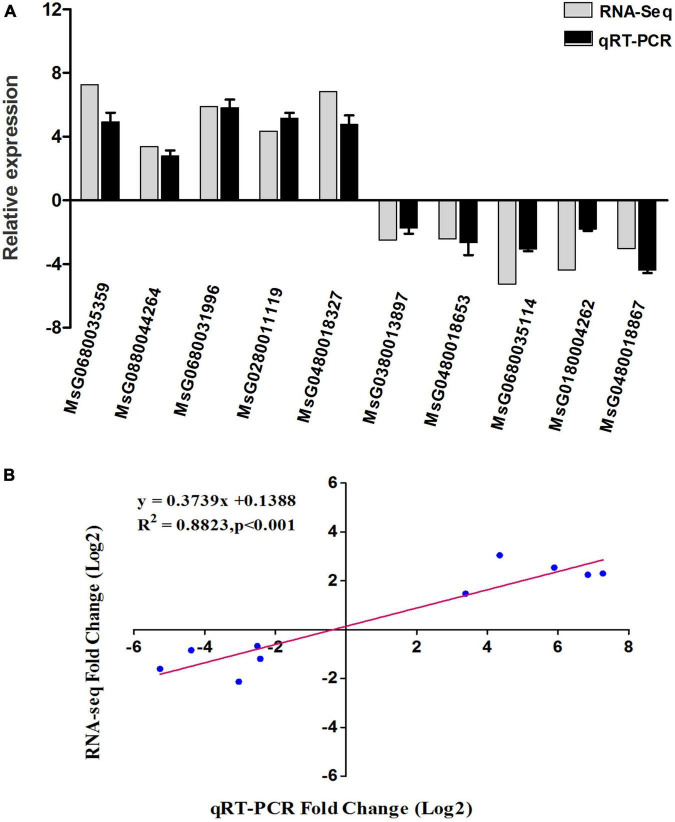
The relative expression levels of 10 DEGs identified in the comparison between RNA-Seq and qRT-PCR. **(A)** The gene relative expression levels were normalized to the expression level of *GAPDH*. Error bars represented the standard deviations of three PCR replicates. **(B)** *R*-values are the correlation coefficients between qRT-PCR and RNA-seq.

Ten DEGs were selected to validate the accuracy of transcriptome data by qRT–PCR. The results indicated that the expression patterns of 10 genes were compatible with the RNA-seq sequencing findings (Figure 8A). RNA-seq and qRT–PCR showed a strong correlation (*R*^2^ = 0.88), which verified the reliability of the RNA-seq analysis in the present research (Figure 8B).

## Discussion

In recent years, a large number of studies have revealed that NFYC transcription factors involved in growth and development processes in plants. Although *NF-YC* genes have been studied in many plant species, such as wheat, *Brachypodium distachyon*, potato, *Brassica napus* and *Arabidopsis*, there have been few studies on *NF-YC* genes in alfalfa (Stephenson et al., 2007; Siefers et al., 2009; Cao et al., 2011; Li et al., 2021; Zheng et al., 2021). Previous studies have shown that *NF-Y* genes show distinct spatiotemporal expression patterns in different tissues and organs (Qu et al., 2020). The expression levels of *MdNF-YC5* and *MdNF-YC8* genes were highly expressed in the sarcocarp and peel. Meanwhile, *MdNF-YC7* and *MdNF-YC8* are specifically expressed in the roots. These results suggested that *NF-YC* genes are critical for growth and development of apple. The transcript levels of most *VvNF-Ys* in the leaves were higher than those in other issues, such as root and stem according to transcriptome data in grape (Ren et al., 2016). In addition, *CsNF-Y2* and *CsNF-YC3* were highly expressed in citrus leaves and flowers, respectively (Pereira et al., 2018). Seventeen *CaNF-Y* genes were identified to be specifically expressed, among which *CaNF-YB03* and *CaNF-YC11* were highly expressed in flower buds in chickpea, indicating that they may play an important role in flower development process (Chu et al., 2018). Twenty-two *RcNF-Ys* genes also showed different expression patterns in tissues, among which *RcNF-YC5* was highly expressed in leaves, *RcNF-YC4*, *RcNF-YC5*, and *RcNF-YC6* were expressed at higher levels in male flowers than other tissues (Wang et al., 2018b). These results indicated that *NF-Ys* genes might be a tissue-specific gene and might plays an important role in synthesis-related biological processes. *PpNF-Ys* showed different expression patterns. Specific *PpNF-YC* genes showed high expression in one or two peach organs (Li et al., 2019), as was the case for *PpNF-YC2* (stems and flowers), *PpNF-YC4* (leaves), and *PpNF-YC6* (fruit). In this study, *MsNF-YC2* was expressed in all the tissues tested, with the transcript being most abundant in young leaves, followed by stems and mature leaves; the expression was lowest in the roots (Figure 1A). These results consistent with previous studies, suggesting that the *MsNF-YC2* plays a critical role in different growth and development stages of alfalfa.

In *Arabidopsis*, the transcript level of *AtNF-YC2* increased sharply in response to heat, cold, and drought stress (Hackenberg et al., 2012). There are also *NF-YC* genes that respond to abiotic stress in other species. For example, the *BnNF-YC2* gene in canola responds to salt and drought stress, *ZmNF-YC6*, *ZmNF-YC8*, and *ZmNF-YC15* were shown to be induced by drought stress and reached highest expression levels in maize (Xu et al., 2014; Zhang et al., 2016). The expression of *MsNF-YC2* was similar to that in the above species and was dramatically induced in response to drought, salt and alkali stress treatments (Figures 1B–D). In this study, an *MsNF-YC2* overexpression vector was constructed and transformed into alfalfa. *MsNF-YC2* overexpression promoted alkali tolerance at all stages of alfalfa growth, which manifested as increased relative water content, improved ROS scavenging system and osmoregulation, reduce cell membrane damage (Figures 3A,B). For example, the relative water content, the activity of SOD and POD and the Pro content in the overexpression *MsNF-YC2* plants were higher than WT plants (Figure 4A,B,D), and the contents of MDA, H_2_O_2_ and ORF were significantly lower than those in the WT plants under alkali stress (Figures 4C,E,F). This shows that changes in physiological indicators in the *MsNF-YC2* OE plants corresponded to their increased alkali tolerance, which was similar to the findings of related studies. For example, under drought stress, the contents of antioxidant enzymes were shown to increase in tobacco overexpressing *AsNF-YC8*, whereas the MDA, H_2_O_2_, and ORF contents decreased (Sun et al., 2017). The relative water content in transgenic overexpressing *CdtNF-YC1* was much higher than that in WT plants (Wu et al., 2018). Similarly, the rice gene *OsNF-YC13* responds to salt stress, and the Pro content increased in *OsNF-YC13*-overexpressing rice (Manimaran et al., 2017). *HAP5* (*NF-YC*) in *Arabidopsis* responds to freezing stress and improves the tolerance of transgenic plants to freezing stress by promoting the activity of SOD and POD and reducing the contents of MDA, H_2_O_2_, and ORF (Shi et al., 2014).

To further investigate the regulatory mechanism of *NFYC2* at the transcriptional level, we performed transcriptome sequencing experiments of the WT and OE plants under alkali stress treatment. Plant hormones such as ABA, auxin, ethylene, salicylic acid and jasmonic acid can amplify signaling cascades or initiate new signaling pathways and thus play critical roles in stress signaling and adaptation (Zhang et al., 2020). It has been reported that plant hormones could regulate stress responses by participating in signal transduction and modifying plant growth and development processes (Sarwat et al., 2013; Wang et al., 2018a). Previous studies have shown that abscisic acid and ethylene play an important role in response to salt stress in *Arabidopsis* (Dong et al., 2011). Compared with WT plants, plants overexpressing *TaNF-YA10-1* are less sensitive to ABA, but their sensitivity to salt stress increases significantly; at the same time, the expression levels of the stress-related genes *AtABI5*, *AtRD29B*, *AtRAB18*, *AtCBF3*, and *AtCBF1* decrease in *Arabidopsis* (Ma et al., 2015). It has been reported that auxin is widely involved in cell growth and differentiation as well as growth and development (Tuan et al., 2019). Studies have found that overexpression of the *TaNF-YB3* gene in wheat increases the chlorophyll content in and photosynthetic rate of leaves (Stephenson et al., 2011). However, overexpression of the *StNF-YB3.1* gene in potato significantly reduced stomatal opening and the photosynthetic rate (Stephenson et al., 2011). In addition, recent studies have identified a CrCO-NF-YB-NF-YC complex from *Chlamydomonas reinhardtii* that is involved in the photoprotective response mechanism (Tokutsu et al., 2019). In our study, comparative transcriptome profiling data showed that the expression of a number of DEGs involved in plant hormone signaling transduction and photosynthesis pathway was significantly regulated. These results indicated that the overexpression of *MsNF-YC2* improved the alkali tolerance of alfalfa, probably through the continuous regulation of key genes involved in hormone signaling and photosynthesis.

## Conclusion

In this study, *MsNF-YC2* was isolated from alfalfa. Overexpression of *MsNF-YC2* enhanced the resistance of transgenic plants to alkali stress. This research has great economic value and provides an important reference for develop alkali tolerant varieties of alfalfa.

## Data availability statement

The datasets presented in this study can be found in online repositories. The names of the repository/repositories and accession number(s) can be found in the article/Supplementary material.

## Author contributions

JY and GC conceived and designed the experiments. JY and YY performed the experiments. JY wrote the manuscript. GC revised the manuscript. All authors analyzed and interpreted the sequence data, read, and approved the final manuscript.

## Footnotes

1
http://web.expasy.org

2
http://kobas.cbi.pku.edu.cn/home.do

3
https://www.ncbi.nlm.nih.gov/Genbank/update.html

4
http://www.ncbi.nlm.nih.gov/bioproject/795604
