# Supplementary material for: Overexpression of an NF-YC2 gene confers alkali tolerance to transgenic alfalfa (Medicago sativa L.)
Source: Front Plant Sci. 2022 Aug 5;13:960160. doi: 10.3389/fpls.2022.960160 (PMC9389336; doi:10.3389/fpls.2022.960160)
Supplement: Supplementary file 1 [file Table_1.docx]

**Supplementary Table 1. Sequences of primers used in qRT-PCR.**

| Primer name | Sequence (5' to 3') |
| --- | --- |
| *MsNF-YC2-*F | ATGAAGAAGAAGCTTGATACTCG |
| *MsNF-YC2-*R | CTACTCTTCCTCCTCATCATAATCT |
| *MsNF-YC2* Real-time PCR- F | AAATGCTTGAGTTGGGCCAC |
| *MsNF-YC2* Real-time PCR- R | TGCCATACTTGGATTGGGGAC |
| *GAPDH-*F | GGCTGCCATCAAGGAGGAAT |
| *GAPDH-*R | TCCAAGCTCAGCCTCATCAAG |
