# Supplementary material for: Overexpression of an NF-YC2 gene confers alkali tolerance to transgenic alfalfa (Medicago sativa L.)
Source: Front Plant Sci. 2022 Aug 5;13:960160. doi: 10.3389/fpls.2022.960160 (PMC9389336; doi:10.3389/fpls.2022.960160)
Supplement: Supplementary file 2 [file Table_2.docx]

Supplementary Table 2: Characterization of the *NF-YC2* gene in alfalfa.

| **Gene name** | **Protein**  **length (aa)** | **MW (kDa)** | **pI** | **Predicted location** |
| --- | --- | --- | --- | --- |
| *MSNF-YC2* | 292 | 32.4 | 5.12 | Nucleus |
