# Supplementary material for: Overexpression of an NF-YC2 gene confers alkali tolerance to transgenic alfalfa (Medicago sativa L.)
Source: Front Plant Sci. 2022 Aug 5;13:960160. doi: 10.3389/fpls.2022.960160 (PMC9389336; doi:10.3389/fpls.2022.960160)
Supplement: Supplementary file 3 [file Table_3.docx]

| **Supplementary Table 3. Differently-expressed genes between transgenic alfalfa plants and WT under normal conditions** | | | | | | | |
| --- | --- | --- | --- | --- | --- | --- | --- |
| gene_id | log2FoldChange | pvalue | padj | Gene  chr | Gene  start | Gene  end | gene_  length |
| novel.4776 | -7.89613 | 2.03E-62 | 6.20E-58 | Chr5 | 91036930 | 91045032 | 6203 |
| MsG0080048743.01 | 6.573355 | 8.17E-46 | 1.25E-41 | contig412end | 2254 | 6979 | 729 |
| MsG0380015003.01 | -5.27797 | 7.26E-44 | 7.39E-40 | Chr3 | 63779636 | 63790311 | 2288 |
| novel.2755 | -5.56793 | 3.55E-35 | 2.71E-31 | Chr3 | 12422350 | 12423458 | 1109 |
| MsG0880042079.01 | 5.372721 | 6.38E-35 | 3.90E-31 | Chr8 | 3471109 | 3481432 | 4710 |
| novel.1419 | 14.80656 | 2.50E-34 | 1.27E-30 | Chr2 | 46351607 | 46352702 | 1096 |
| MsG0480020539.01 | 7.901188 | 2.34E-33 | 1.02E-29 | Chr4 | 43035369 | 43045234 | 2066 |
| MsG0880044521.01 | 3.658665 | 2.94E-29 | 1.12E-25 | Chr8 | 43687317 | 43693649 | 918 |
| novel.1716 | -4.61607 | 1.71E-27 | 5.80E-24 | Chr2 | 8645091 | 8648726 | 3060 |
| novel.7466 | -6.04354 | 2.71E-27 | 8.29E-24 | Chr7 | 38358255 | 38361672 | 2051 |
| novel.3180 | 13.0389 | 1.55E-26 | 4.29E-23 | Chr3 | 99398050 | 99400218 | 2169 |
| novel.2967 | 3.94678 | 2.67E-26 | 6.39E-23 | Chr3 | 65550220 | 65551657 | 745 |
| novel.4999 | 5.679078 | 2.72E-26 | 6.39E-23 | Chr5 | 15655702 | 15660613 | 2341 |
| novel.2268 | 6.973684 | 2.06E-25 | 4.49E-22 | Chr3 | 23285856 | 23294564 | 4802 |
| novel.8806 | -6.23065 | 9.35E-25 | 1.91E-21 | contig127end | 10613 | 16365 | 2365 |
| novel.8934 | 12.44741 | 1.08E-24 | 2.06E-21 | contig315end | 23509 | 26204 | 725 |
| novel.536 | -5.12533 | 1.97E-24 | 3.55E-21 | Chr1 | 95603895 | 95610266 | 3224 |
| MsG0380013598.01 | 4.5539 | 3.77E-24 | 6.39E-21 | Chr3 | 41423445 | 41428939 | 1729 |
| MsG0180004146.01 | -3.47912 | 5.39E-24 | 8.67E-21 | Chr1 | 73978121 | 73981697 | 1520 |
| MsG0880044283.01 | 9.309344 | 3.35E-23 | 5.12E-20 | Chr8 | 39391996 | 39406469 | 2775 |
| novel.5039 | 5.845173 | 3.61E-23 | 5.25E-20 | Chr5 | 25553619 | 25557156 | 3166 |
| MsG0480022947.01 | 6.997225 | 4.84E-23 | 6.72E-20 | Chr4 | 78951727 | 78954311 | 1029 |
| MsG0880043781.01 | -5.22457 | 6.52E-23 | 8.66E-20 | Chr8 | 30510835 | 30513267 | 2118 |
| MsG0780037601.01 | 5.105752 | 1.16E-22 | 1.48E-19 | Chr7 | 29108954 | 29132298 | 8694 |
| MsG0380013725.01 | -4.50213 | 1.78E-22 | 2.18E-19 | Chr3 | 44647588 | 44648367 | 780 |
| MsG0680033567.01 | 7.032095 | 4.27E-22 | 5.02E-19 | Chr6 | 68434691 | 68440587 | 2582 |
| MsG0580025976.01 | 11.6841 | 8.50E-22 | 9.62E-19 | Chr5 | 28445748 | 28445969 | 222 |
| novel.4251 | -3.60531 | 1.61E-21 | 1.76E-18 | Chr4 | 90280549 | 90286200 | 2703 |
| novel.2381 | 12.22041 | 1.71E-21 | 1.80E-18 | Chr3 | 55139168 | 55140887 | 1720 |
| MsG0280010535.01 | -6.34885 | 2.91E-21 | 2.92E-18 | Chr2 | 71130479 | 71131441 | 963 |
| novel.3151 | 2.088685 | 2.96E-21 | 2.92E-18 | Chr3 | 95853414 | 95854674 | 1261 |
| MsG0180001208.01 | 8.609999 | 4.31E-21 | 4.11E-18 | Chr1 | 17402944 | 17407714 | 1686 |
| novel.6937 | 3.645056 | 7.08E-21 | 6.56E-18 | Chr7 | 30006324 | 30014184 | 1566 |
| MsG0480018894.01 | 4.976822 | 1.50E-20 | 1.35E-17 | Chr4 | 11246140 | 11249947 | 298 |
| MsG0480019313.01 | -2.92716 | 1.60E-20 | 1.40E-17 | Chr4 | 18350355 | 18353224 | 2680 |
| MsG0280006695.01 | 2.562924 | 1.66E-20 | 1.41E-17 | Chr2 | 5091435 | 5099625 | 2171 |
| novel.2742 | -5.96227 | 2.26E-20 | 1.87E-17 | Chr3 | 11220521 | 11221459 | 939 |
| MsG0380012312.01 | -6.04932 | 2.61E-20 | 2.10E-17 | Chr3 | 14717168 | 14722996 | 4350 |
| novel.3222 | 2.40045 | 2.77E-20 | 2.17E-17 | Chr4 | 828248 | 831310 | 1839 |
| MsG0480019312.01 | -5.51648 | 1.09E-19 | 8.34E-17 | Chr4 | 18338092 | 18338583 | 492 |
| novel.228 | 7.663855 | 3.18E-19 | 2.37E-16 | Chr1 | 35604955 | 35607365 | 2411 |
| MsG0580025156.01 | -6.48653 | 4.51E-19 | 3.28E-16 | Chr5 | 14581512 | 14587560 | 1230 |
| MsG0880042185.01 | -9.79595 | 5.74E-19 | 4.08E-16 | Chr8 | 5088182 | 5089281 | 948 |
| MsG0080047963.01 | 6.734824 | 6.69E-19 | 4.65E-16 | contig152end | 411542 | 421148 | 870 |
| MsG0180005083.01 | 10.96823 | 1.37E-18 | 9.28E-16 | Chr1 | 86734827 | 86740674 | 1569 |
| novel.6523 | -4.07668 | 1.57E-18 | 1.04E-15 | Chr6 | 67238276 | 67248512 | 6199 |
| novel.5905 | 10.76527 | 1.95E-18 | 1.27E-15 | Chr6 | 80424582 | 80428634 | 3430 |
| novel.8231 | 8.889508 | 3.15E-18 | 2.01E-15 | Chr8 | 82685940 | 82689072 | 1765 |
| MsG0180003711.01 | 6.499285 | 7.68E-18 | 4.79E-15 | Chr1 | 67210716 | 67211126 | 411 |
| MsG0880047145.01 | -7.56108 | 8.20E-18 | 5.01E-15 | Chr8 | 82623803 | 82624731 | 249 |
| novel.7429 | -7.48862 | 1.43E-17 | 8.43E-15 | Chr7 | 29666464 | 29669742 | 3279 |
| MsG0880043721.01 | -6.85683 | 1.44E-17 | 8.43E-15 | Chr8 | 29618874 | 29625806 | 3571 |
| MsG0880045651.01 | 10.5401 | 1.72E-17 | 9.94E-15 | Chr8 | 62178961 | 62182585 | 2011 |
| MsG0880046534.01 | -7.10308 | 1.90E-17 | 1.08E-14 | Chr8 | 74474957 | 74481416 | 2292 |
| MsG0680035017.01 | -3.61227 | 2.81E-17 | 1.56E-14 | Chr6 | 97800515 | 97816056 | 7745 |
| novel.8265 | -5.91193 | 3.23E-17 | 1.76E-14 | Chr8 | 87338896 | 87342529 | 3244 |
| MsG0580025812.01 | -4.03474 | 3.43E-17 | 1.84E-14 | Chr5 | 24554437 | 24561296 | 1997 |
| novel.5836 | -5.36223 | 4.15E-17 | 2.19E-14 | Chr6 | 66628395 | 66633069 | 2120 |
| novel.5328 | -4.38832 | 1.59E-16 | 8.25E-14 | Chr5 | 83451695 | 83457064 | 2514 |
| MsG0180003938.01 | 2.243429 | 2.08E-16 | 1.06E-13 | Chr1 | 70600279 | 70609536 | 2018 |
| MsG0680032751.01 | 4.063091 | 2.13E-16 | 1.07E-13 | Chr6 | 48583915 | 48587341 | 2748 |
| novel.3800 | -4.66046 | 2.20E-16 | 1.09E-13 | Chr4 | 7428924 | 7434471 | 2073 |
| MsG0780036706.01 | -3.59864 | 2.31E-16 | 1.12E-13 | Chr7 | 12216031 | 12219171 | 1334 |
| novel.1841 | -4.17135 | 2.47E-16 | 1.18E-13 | Chr2 | 31663423 | 31667553 | 2061 |
| MsG0780040961.01 | 10.713 | 3.08E-16 | 1.44E-13 | Chr7 | 84858368 | 84869795 | 6321 |
| novel.6117 | 10.27866 | 3.12E-16 | 1.44E-13 | Chr6 | 1.12E+08 | 1.12E+08 | 891 |
| novel.6586 | 10.12749 | 3.32E-16 | 1.51E-13 | Chr6 | 80144635 | 80147746 | 1138 |
| MsG0180003115.01 | 2.72274 | 4.14E-16 | 1.86E-13 | Chr1 | 56840543 | 56843869 | 910 |
| MsG0380013784.01 | 5.216571 | 4.89E-16 | 2.17E-13 | Chr3 | 45652867 | 45655203 | 2337 |
| MsG0880044221.01 | -10.528 | 5.49E-16 | 2.40E-13 | Chr8 | 37869045 | 37870424 | 1380 |
| novel.7921 | 10.48737 | 7.00E-16 | 2.99E-13 | Chr8 | 22726724 | 22728111 | 1271 |
| novel.1314 | 4.995824 | 7.05E-16 | 2.99E-13 | Chr2 | 24703099 | 24705440 | 1598 |
| novel.6505 | 4.385575 | 9.41E-16 | 3.94E-13 | Chr6 | 62849888 | 62853481 | 1121 |
| novel.2220 | -10.4511 | 9.70E-16 | 4.01E-13 | Chr3 | 15986852 | 16006849 | 5728 |
| MsG0680030903.01 | 5.642322 | 1.21E-15 | 4.92E-13 | Chr6 | 11348092 | 11354288 | 1509 |
| MsG0580025725.01 | -6.06167 | 1.45E-15 | 5.85E-13 | Chr5 | 23234952 | 23243295 | 331 |
| novel.2560 | 3.868978 | 1.52E-15 | 6.03E-13 | Chr3 | 86219082 | 86225575 | 3512 |
| novel.1724 | -10.6386 | 1.65E-15 | 6.48E-13 | Chr2 | 10847719 | 10853367 | 5649 |
| novel.5731 | 3.847858 | 1.68E-15 | 6.51E-13 | Chr6 | 40459432 | 40462256 | 2188 |
| MsG0580024933.01 | 5.493413 | 2.23E-15 | 8.53E-13 | Chr5 | 11811036 | 11812472 | 1437 |
| novel.2883 | 4.039853 | 2.34E-15 | 8.84E-13 | Chr3 | 44151393 | 44161057 | 1730 |
| novel.6768 | -5.78424 | 2.47E-15 | 9.20E-13 | Chr6 | 1.1E+08 | 1.1E+08 | 819 |
| MsG0780039104.01 | -6.46462 | 2.60E-15 | 9.59E-13 | Chr7 | 57965841 | 57968952 | 1278 |
| MsG0680030639.01 | 4.623977 | 4.62E-15 | 1.68E-12 | Chr6 | 6495286 | 6496042 | 624 |
| MsG0580027139.01 | 7.265098 | 6.32E-15 | 2.27E-12 | Chr5 | 54269581 | 54270692 | 850 |
| MsG0880045587.01 | -10.3089 | 1.06E-14 | 3.73E-12 | Chr8 | 61390387 | 61393114 | 1089 |
| novel.6191 | 4.445734 | 1.06E-14 | 3.73E-12 | Chr6 | 8053860 | 8058583 | 925 |
| novel.1064 | -4.48527 | 1.09E-14 | 3.79E-12 | Chr1 | 90256048 | 90259270 | 1884 |
| novel.2480 | -3.24197 | 1.11E-14 | 3.80E-12 | Chr3 | 75292532 | 75294904 | 2373 |
| novel.6767 | -4.89781 | 1.12E-14 | 3.80E-12 | Chr6 | 1.1E+08 | 1.1E+08 | 983 |
| novel.6730 | 4.02162 | 1.21E-14 | 4.05E-12 | Chr6 | 1.05E+08 | 1.05E+08 | 1477 |
| MsG0280011107.01 | 2.925083 | 1.33E-14 | 4.43E-12 | Chr2 | 79657582 | 79661887 | 2073 |
| novel.1799 | -10.1737 | 1.36E-14 | 4.48E-12 | Chr2 | 24037107 | 24041634 | 3092 |
| novel.3171 | -5.37176 | 1.56E-14 | 5.06E-12 | Chr3 | 97932027 | 97936387 | 1529 |
| novel.7355 | 9.712695 | 2.23E-14 | 7.19E-12 | Chr7 | 13987504 | 13988274 | 771 |
| novel.12 | 3.074131 | 2.84E-14 | 9.05E-12 | Chr1 | 1777223 | 1819048 | 4616 |
| novel.6183 | -7.1974 | 3.08E-14 | 9.71E-12 | Chr6 | 7545067 | 7547800 | 1972 |
| MsG0480018683.01 | -5.00115 | 3.46E-14 | 1.08E-11 | Chr4 | 8552648 | 8565604 | 1048 |
| novel.1300 | -9.98331 | 3.58E-14 | 1.10E-11 | Chr2 | 22457931 | 22460677 | 2747 |
| MsG0880045816.01 | -9.56182 | 3.63E-14 | 1.10E-11 | Chr8 | 64848994 | 64849488 | 495 |
| novel.6688 | -6.35672 | 3.64E-14 | 1.10E-11 | Chr6 | 1.01E+08 | 1.01E+08 | 2275 |
| MsG0380012991.01 | 8.208885 | 5.10E-14 | 1.53E-11 | Chr3 | 28380970 | 28398030 | 1290 |
| novel.2382 | 9.525176 | 5.28E-14 | 1.57E-11 | Chr3 | 55173891 | 55176780 | 2253 |
| MsG0080047898.01 | 5.237571 | 5.64E-14 | 1.66E-11 | contig13end | 10480 | 14473 | 756 |
| novel.6281 | 4.424111 | 5.96E-14 | 1.74E-11 | Chr6 | 19855664 | 19859722 | 1898 |
| novel.2001 | 2.706515 | 6.06E-14 | 1.75E-11 | Chr2 | 69424700 | 69428286 | 2965 |
| novel.8356 | -6.41838 | 6.75E-14 | 1.93E-11 | Chr8 | 10238957 | 10244095 | 3276 |
| novel.5513 | -2.57575 | 7.58E-14 | 2.15E-11 | Chr6 | 1158118 | 1159219 | 1102 |
| MsG0580029421.01 | 2.899628 | 8.36E-14 | 2.34E-11 | Chr5 | 96785924 | 96789561 | 813 |
| MsG0480018531.01 | -9.59173 | 9.13E-14 | 2.54E-11 | Chr4 | 6210489 | 6220952 | 5786 |
| MsG0480019152.01 | 9.527815 | 9.80E-14 | 2.70E-11 | Chr4 | 15135235 | 15136756 | 708 |
| novel.2756 | 3.475431 | 1.05E-13 | 2.86E-11 | Chr3 | 12430826 | 12434319 | 2043 |
| novel.5317 | -9.20877 | 1.13E-13 | 3.06E-11 | Chr5 | 82484842 | 82487691 | 1663 |
| MsG0580027669.01 | 4.240846 | 1.65E-13 | 4.42E-11 | Chr5 | 65723102 | 65726865 | 1445 |
| novel.8079 | 9.27939 | 1.98E-13 | 5.27E-11 | Chr8 | 55960025 | 55961743 | 1163 |
| novel.4066 | 9.278092 | 2.04E-13 | 5.38E-11 | Chr4 | 65651843 | 65653469 | 843 |
| novel.8513 | -5.48184 | 2.37E-13 | 6.20E-11 | Chr8 | 44743657 | 44746537 | 2030 |
| novel.5284 | 9.302173 | 2.68E-13 | 6.94E-11 | Chr5 | 74838736 | 74840716 | 1693 |
| MsG0680031771.01 | 2.568633 | 2.82E-13 | 7.24E-11 | Chr6 | 26461625 | 26463210 | 609 |
| MsG0680034720.01 | -3.8655 | 3.14E-13 | 7.99E-11 | Chr6 | 93403207 | 93407231 | 3411 |
| novel.6256 | 3.797829 | 3.36E-13 | 8.48E-11 | Chr6 | 16862261 | 16864113 | 1853 |
| novel.7140 | -8.46862 | 3.47E-13 | 8.69E-11 | Chr7 | 75411649 | 75414386 | 1384 |
| MsG0780039096.01 | -4.35632 | 3.87E-13 | 9.62E-11 | Chr7 | 57891139 | 57895254 | 3012 |
| novel.3445 | -3.13567 | 4.05E-13 | 9.98E-11 | Chr4 | 44531973 | 44547112 | 3128 |
| novel.1276 | 3.989125 | 4.14E-13 | 1.01E-10 | Chr2 | 17110202 | 17112032 | 1831 |
| novel.3755 | -9.21272 | 4.44E-13 | 1.08E-10 | Chr4 | 1303710 | 1305237 | 1528 |
| novel.8226 | 5.709936 | 4.68E-13 | 1.13E-10 | Chr8 | 81973982 | 81978965 | 4984 |
| MsG0380012252.01 | -3.60575 | 6.79E-13 | 1.62E-10 | Chr3 | 13347771 | 13354367 | 2553 |
| MsG0380016228.01 | 2.405704 | 6.96E-13 | 1.65E-10 | Chr3 | 80718209 | 80719191 | 899 |
| MsG0580025808.01 | -4.18859 | 8.57E-13 | 2.01E-10 | Chr5 | 24521163 | 24526043 | 873 |
| MsG0780038984.01 | 9.179403 | 9.78E-13 | 2.28E-10 | Chr7 | 56357595 | 56389846 | 5100 |
| MsG0380014405.01 | -9.22228 | 9.87E-13 | 2.28E-10 | Chr3 | 54230316 | 54233102 | 2787 |
| novel.763 | -2.39025 | 1.07E-12 | 2.46E-10 | Chr1 | 24881654 | 24882943 | 472 |
| novel.6260 | 9.039725 | 1.24E-12 | 2.83E-10 | Chr6 | 17072888 | 17078002 | 2852 |
| novel.9042 | -9.50879 | 1.27E-12 | 2.88E-10 | contig451end | 31391 | 32536 | 1146 |
| novel.5871 | -4.91001 | 1.33E-12 | 3.00E-10 | Chr6 | 74525666 | 74551313 | 1783 |
| MsG0880043896.01 | 9.393572 | 1.35E-12 | 3.01E-10 | Chr8 | 32316005 | 32316970 | 966 |
| MsG0880043254.01 | 6.704808 | 1.48E-12 | 3.27E-10 | Chr8 | 21314631 | 21316647 | 267 |
| MsG0480018855.01 | 9.070615 | 1.57E-12 | 3.46E-10 | Chr4 | 10650748 | 10654525 | 1224 |
| MsG0380013886.01 | -6.04421 | 1.58E-12 | 3.46E-10 | Chr3 | 47497666 | 47508267 | 2817 |
| MsG0280006692.01 | -2.60532 | 1.73E-12 | 3.75E-10 | Chr2 | 5057470 | 5060941 | 1481 |
| novel.2486 | -4.60489 | 1.76E-12 | 3.78E-10 | Chr3 | 76219994 | 76223750 | 2789 |
| novel.7979 | -9.03046 | 1.78E-12 | 3.80E-10 | Chr8 | 35681646 | 35683704 | 376 |
| MsG0180001580.01 | 9.115571 | 1.80E-12 | 3.82E-10 | Chr1 | 23640708 | 23642997 | 2154 |
| novel.5828 | -9.53109 | 1.84E-12 | 3.88E-10 | Chr6 | 65005665 | 65008983 | 2066 |
| MsG0680035895.01 | -9.11052 | 1.88E-12 | 3.94E-10 | Chr6 | 1.14E+08 | 1.14E+08 | 8007 |
| novel.2970 | 4.85865 | 2.33E-12 | 4.85E-10 | Chr3 | 66427407 | 66432226 | 4820 |
| novel.3479 | 4.901801 | 3.04E-12 | 6.27E-10 | Chr4 | 52918464 | 52921433 | 1419 |
| novel.6678 | 9.138343 | 3.18E-12 | 6.53E-10 | Chr6 | 99848461 | 99850486 | 1621 |
| novel.8064 | -5.60173 | 3.69E-12 | 7.52E-10 | Chr8 | 52455483 | 52457920 | 1599 |
| novel.6456 | 8.881015 | 3.87E-12 | 7.83E-10 | Chr6 | 51366600 | 51370308 | 1013 |
| novel.7646 | -5.76122 | 4.24E-12 | 8.53E-10 | Chr7 | 75260563 | 75262921 | 1443 |
| novel.1597 | 5.208597 | 4.53E-12 | 9.06E-10 | Chr2 | 79795703 | 79799545 | 1593 |
| novel.6702 | 3.910874 | 5.06E-12 | 1.00E-09 | Chr6 | 1.02E+08 | 1.02E+08 | 737 |
| MsG0180004245.01 | -5.10151 | 5.43E-12 | 1.07E-09 | Chr1 | 75504511 | 75509226 | 630 |
| MsG0280010734.01 | -9.41205 | 5.47E-12 | 1.07E-09 | Chr2 | 74051066 | 74056327 | 5262 |
| MsG0680033510.01 | -8.90481 | 5.87E-12 | 1.14E-09 | Chr6 | 66915140 | 66917593 | 1203 |
| MsG0780039990.01 | 2.313066 | 5.96E-12 | 1.15E-09 | Chr7 | 71596685 | 71600377 | 1364 |
| MsG0480019585.01 | -3.67982 | 5.96E-12 | 1.15E-09 | Chr4 | 22683013 | 22687656 | 2199 |
| MsG0880042901.01 | -4.44706 | 6.60E-12 | 1.26E-09 | Chr8 | 15655616 | 15656443 | 828 |
| novel.7825 | 8.856882 | 6.62E-12 | 1.26E-09 | Chr8 | 6017701 | 6019770 | 671 |
| novel.2336 | 8.913389 | 6.85E-12 | 1.29E-09 | Chr3 | 42718753 | 42721099 | 2062 |
| MsG0680030326.01 | 5.536978 | 7.01E-12 | 1.32E-09 | Chr6 | 620428 | 621780 | 1353 |
| MsG0580026014.01 | -9.16134 | 7.85E-12 | 1.46E-09 | Chr5 | 29098615 | 29100004 | 740 |
| novel.191 | 8.910083 | 8.26E-12 | 1.53E-09 | Chr1 | 26166993 | 26168724 | 659 |
| novel.3869 | 8.965317 | 9.19E-12 | 1.69E-09 | Chr4 | 23072585 | 23074016 | 720 |
| novel.4478 | 8.947553 | 9.94E-12 | 1.82E-09 | Chr5 | 30094567 | 30096670 | 307 |
| novel.3908 | 2.90367 | 1.04E-11 | 1.90E-09 | Chr4 | 32207880 | 32215605 | 2590 |
| novel.2288 | -9.31661 | 1.10E-11 | 1.99E-09 | Chr3 | 29110289 | 29115229 | 1115 |
| MsG0280008210.01 | 3.576093 | 1.11E-11 | 1.99E-09 | Chr2 | 27211068 | 27212010 | 789 |
| MsG0880046107.01 | -6.64289 | 1.14E-11 | 2.04E-09 | Chr8 | 69096638 | 69098233 | 884 |
| MsG0380014844.01 | -5.18755 | 1.35E-11 | 2.40E-09 | Chr3 | 61070640 | 61086113 | 5346 |
| MsG0680035015.01 | -3.37869 | 1.49E-11 | 2.64E-09 | Chr6 | 97771104 | 97780401 | 4998 |
| MsG0580029753.01 | -2.24098 | 1.56E-11 | 2.74E-09 | Chr5 | 1.02E+08 | 1.02E+08 | 1261 |
| novel.4879 | -8.70573 | 1.69E-11 | 2.96E-09 | Chr5 | 1.05E+08 | 1.05E+08 | 1319 |
| MsG0180002088.01 | -3.26604 | 2.10E-11 | 3.63E-09 | Chr1 | 32627446 | 32640631 | 8135 |
| MsG0180003457.01 | 8.677539 | 2.10E-11 | 3.63E-09 | Chr1 | 62525843 | 62526190 | 231 |
| MsG0480018989.01 | 1.605655 | 2.15E-11 | 3.68E-09 | Chr4 | 12888964 | 12893070 | 942 |
| novel.3123 | 2.258601 | 2.17E-11 | 3.70E-09 | Chr3 | 89801980 | 89809191 | 5316 |
| MsG0680030863.01 | 9.158612 | 2.24E-11 | 3.80E-09 | Chr6 | 10612210 | 10612368 | 159 |
| MsG0080048687.01 | -3.83804 | 2.50E-11 | 4.22E-09 | contig400end | 15444 | 19216 | 296 |
| novel.7473 | -8.89045 | 3.06E-11 | 5.14E-09 | Chr7 | 40322131 | 40326944 | 4706 |
| novel.7796 | 4.442763 | 3.12E-11 | 5.20E-09 | Chr8 | 1733604 | 1734562 | 959 |
| MsG0180000927.01 | 8.739349 | 3.18E-11 | 5.27E-09 | Chr1 | 13338244 | 13341290 | 2223 |
| novel.4870 | 8.657179 | 3.28E-11 | 5.40E-09 | Chr5 | 1.04E+08 | 1.04E+08 | 1747 |
| MsG0780040577.01 | 2.401062 | 3.28E-11 | 5.40E-09 | Chr7 | 79439944 | 79440162 | 219 |
| MsG0180001586.01 | 8.699158 | 3.32E-11 | 5.43E-09 | Chr1 | 23749562 | 23752851 | 3042 |
| MsG0280010384.01 | 8.572051 | 3.35E-11 | 5.45E-09 | Chr2 | 68841077 | 68841235 | 159 |
| MsG0680035359.01 | 3.036486 | 4.01E-11 | 6.47E-09 | Chr6 | 1.03E+08 | 1.03E+08 | 2742 |
| novel.7050 | -5.55248 | 4.02E-11 | 6.47E-09 | Chr7 | 59076563 | 59078791 | 1450 |
| novel.7311 | 4.970083 | 4.05E-11 | 6.48E-09 | Chr7 | 2712540 | 2728880 | 1531 |
| novel.6744 | 8.751338 | 4.68E-11 | 7.45E-09 | Chr6 | 1.08E+08 | 1.08E+08 | 4048 |
| MsG0180000878.01 | -2.03992 | 5.51E-11 | 8.73E-09 | Chr1 | 12630323 | 12632150 | 879 |
| novel.7403 | -6.20951 | 6.11E-11 | 9.62E-09 | Chr7 | 21456799 | 21460544 | 3746 |
| MsG0780040959.01 | 8.513629 | 6.59E-11 | 1.03E-08 | Chr7 | 84832188 | 84834591 | 869 |
| novel.4496 | -2.2383 | 7.07E-11 | 1.10E-08 | Chr5 | 34082676 | 34087757 | 2641 |
| MsG0180001395.01 | -3.87915 | 7.59E-11 | 1.18E-08 | Chr1 | 20506526 | 20518899 | 8133 |
| MsG0780037636.01 | -3.34726 | 7.62E-11 | 1.18E-08 | Chr7 | 29859657 | 29865001 | 1248 |
| novel.9125 | -8.84966 | 8.85E-11 | 1.36E-08 | contig503end | 23936 | 25415 | 1375 |
| novel.2567 | 3.095077 | 8.89E-11 | 1.36E-08 | Chr3 | 87147390 | 87153951 | 2716 |
| MsG0280006536.01 | -4.45802 | 9.21E-11 | 1.40E-08 | Chr2 | 3205166 | 3206658 | 564 |
| MsG0680032191.01 | 2.239324 | 9.33E-11 | 1.41E-08 | Chr6 | 35886346 | 35890197 | 546 |
| MsG0180005752.01 | -3.91574 | 9.50E-11 | 1.43E-08 | Chr1 | 95686440 | 95687180 | 741 |
| MsG0680031364.01 | 8.433423 | 9.55E-11 | 1.43E-08 | Chr6 | 19012956 | 19015649 | 799 |
| novel.722 | 8.564273 | 1.01E-10 | 1.51E-08 | Chr1 | 17359236 | 17361382 | 2147 |
| MsG0680031058.01 | 8.462821 | 1.02E-10 | 1.52E-08 | Chr6 | 13420861 | 13422885 | 1371 |
| novel.6749 | -4.11702 | 1.08E-10 | 1.60E-08 | Chr6 | 1.08E+08 | 1.08E+08 | 1919 |
| MsG0480018494.01 | -8.89366 | 1.12E-10 | 1.65E-08 | Chr4 | 5769333 | 5772699 | 98 |
| MsG0280006388.01 | -8.30225 | 1.16E-10 | 1.69E-08 | Chr2 | 1213648 | 1223567 | 1803 |
| novel.2263 | 8.455437 | 1.27E-10 | 1.85E-08 | Chr3 | 22528770 | 22530975 | 1650 |
| MsG0780036697.01 | -3.95202 | 1.32E-10 | 1.91E-08 | Chr7 | 12076523 | 12079811 | 406 |
| novel.6524 | 2.752578 | 1.37E-10 | 1.97E-08 | Chr6 | 67550507 | 67551500 | 664 |
| novel.2602 | -9.09525 | 1.39E-10 | 2.00E-08 | Chr3 | 93990461 | 93994452 | 1369 |
| MsG0280007259.01 | 4.419572 | 1.49E-10 | 2.12E-08 | Chr2 | 12690213 | 12697043 | 1155 |
| novel.7732 | -2.36433 | 1.49E-10 | 2.12E-08 | Chr7 | 88498304 | 88500527 | 1651 |
| novel.7143 | 2.506139 | 1.61E-10 | 2.27E-08 | Chr7 | 75525399 | 75531265 | 3953 |
| novel.2612 | 8.183424 | 1.62E-10 | 2.28E-08 | Chr3 | 95103459 | 95106226 | 1999 |
| novel.6913 | -6.49799 | 1.72E-10 | 2.41E-08 | Chr7 | 26251416 | 26253139 | 461 |
| MsG0180000941.01 | -8.57145 | 1.76E-10 | 2.44E-08 | Chr1 | 13493108 | 13494871 | 1419 |
| MsG0380014813.01 | 4.67882 | 1.76E-10 | 2.44E-08 | Chr3 | 60536528 | 60546953 | 5187 |
| novel.7971 | -4.0562 | 1.81E-10 | 2.50E-08 | Chr8 | 33982210 | 33982524 | 315 |
| MsG0580025514.01 | 7.205331 | 1.94E-10 | 2.67E-08 | Chr5 | 19862143 | 19863207 | 204 |
| MsG0780040954.01 | 8.633926 | 2.01E-10 | 2.75E-08 | Chr7 | 84738212 | 84743529 | 2015 |
| novel.7412 | 5.869711 | 2.13E-10 | 2.91E-08 | Chr7 | 23862143 | 23864844 | 784 |
| MsG0180001098.01 | 8.364009 | 2.21E-10 | 3.00E-08 | Chr1 | 15975994 | 15976685 | 336 |
| novel.2131 | 8.606967 | 2.26E-10 | 3.06E-08 | Chr3 | 1935652 | 1937703 | 669 |
| novel.4848 | 3.78905 | 2.42E-10 | 3.26E-08 | Chr5 | 1.01E+08 | 1.01E+08 | 586 |
| MsG0780038368.01 | -5.47997 | 2.54E-10 | 3.41E-08 | Chr7 | 45229172 | 45252724 | 4785 |
| novel.5382 | -5.49247 | 2.56E-10 | 3.42E-08 | Chr5 | 91960161 | 91964421 | 2066 |
| novel.2491 | 2.272019 | 2.65E-10 | 3.51E-08 | Chr3 | 77442629 | 77445233 | 1251 |
| MsG0780040241.01 | 3.844332 | 2.65E-10 | 3.51E-08 | Chr7 | 74819690 | 74820956 | 909 |
| novel.2383 | 5.647075 | 2.76E-10 | 3.63E-08 | Chr3 | 55178085 | 55179008 | 924 |
| MsG0180000579.01 | -9.22195 | 2.93E-10 | 3.85E-08 | Chr1 | 8074029 | 8075972 | 1944 |
| MsG0880042503.01 | -1.97424 | 2.96E-10 | 3.87E-08 | Chr8 | 9675863 | 9680163 | 564 |
| novel.3058 | 3.798097 | 3.05E-10 | 3.97E-08 | Chr3 | 79752487 | 79753952 | 1249 |
| MsG0680030954.01 | -5.54847 | 3.09E-10 | 4.00E-08 | Chr6 | 12126902 | 12134411 | 5170 |
| MsG0180000772.01 | 5.299859 | 3.10E-10 | 4.00E-08 | Chr1 | 10900800 | 10917684 | 4218 |
| MsG0180003116.01 | 3.959595 | 3.14E-10 | 4.03E-08 | Chr1 | 56848800 | 56852050 | 780 |
| novel.2331 | 3.692404 | 3.16E-10 | 4.04E-08 | Chr3 | 41206874 | 41207986 | 1113 |
| MsG0880045598.01 | 8.401131 | 3.28E-10 | 4.18E-08 | Chr8 | 61565291 | 61577903 | 1956 |
| novel.6680 | 8.453784 | 3.45E-10 | 4.37E-08 | Chr6 | 99880997 | 99886543 | 3144 |
| novel.991 | 1.952998 | 3.50E-10 | 4.41E-08 | Chr1 | 79596970 | 79599916 | 2224 |
| MsG0880044318.01 | -2.41548 | 3.57E-10 | 4.49E-08 | Chr8 | 39914695 | 39916464 | 387 |
| MsG0880044832.01 | -4.50344 | 3.61E-10 | 4.52E-08 | Chr8 | 49890998 | 49894336 | 2124 |
| MsG0080048430.01 | 8.301971 | 3.76E-10 | 4.69E-08 | contig315end | 17815 | 21992 | 3081 |
| MsG0580028095.01 | -8.6534 | 3.79E-10 | 4.71E-08 | Chr5 | 74500091 | 74510991 | 6387 |
| MsG0880046204.01 | 2.57798 | 4.07E-10 | 5.01E-08 | Chr8 | 70494106 | 70501246 | 2836 |
| MsG0880044915.01 | -8.31371 | 4.07E-10 | 5.01E-08 | Chr8 | 51362437 | 51362925 | 489 |
| novel.6077 | -8.48695 | 4.11E-10 | 5.04E-08 | Chr6 | 1.06E+08 | 1.06E+08 | 2511 |
| MsG0580024170.01 | -3.41225 | 4.31E-10 | 5.27E-08 | Chr5 | 1531285 | 1533345 | 1413 |
| novel.4680 | -2.85672 | 4.48E-10 | 5.45E-08 | Chr5 | 74315848 | 74319027 | 2424 |
| MsG0180000757.01 | -8.77435 | 4.92E-10 | 5.96E-08 | Chr1 | 10698670 | 10698939 | 270 |
| MsG0280009830.01 | -8.77287 | 5.10E-10 | 6.14E-08 | Chr2 | 59044418 | 59050066 | 465 |
| novel.4423 | 8.203612 | 5.10E-10 | 6.14E-08 | Chr5 | 22717079 | 22718458 | 1380 |
| novel.7737 | 4.57687 | 5.31E-10 | 6.37E-08 | Chr7 | 89117683 | 89119129 | 641 |
| MsG0680035014.01 | -5.42821 | 5.43E-10 | 6.49E-08 | Chr6 | 97766021 | 97768828 | 759 |
| MsG0880043539.01 | -2.82276 | 5.49E-10 | 6.53E-08 | Chr8 | 26290576 | 26302048 | 3000 |
| novel.2516 | 4.145355 | 5.65E-10 | 6.69E-08 | Chr3 | 81418026 | 81422508 | 2866 |
| MsG0680032758.01 | 1.828711 | 5.74E-10 | 6.77E-08 | Chr6 | 48932203 | 48934218 | 2016 |
| MsG0280011221.01 | -7.51073 | 5.92E-10 | 6.96E-08 | Chr2 | 81144347 | 81148063 | 2393 |
| MsG0180006168.01 | -3.81069 | 6.18E-10 | 7.24E-08 | Chr1 | 1.01E+08 | 1.01E+08 | 1575 |
| novel.7880 | -3.79392 | 6.24E-10 | 7.28E-08 | Chr8 | 16439374 | 16442373 | 2174 |
| MsG0180005607.01 | -3.1037 | 6.34E-10 | 7.37E-08 | Chr1 | 93912030 | 93915270 | 1235 |
| MsG0880043537.01 | -3.12462 | 6.51E-10 | 7.53E-08 | Chr8 | 26273874 | 26277462 | 1673 |
| novel.2167 | 3.515917 | 6.53E-10 | 7.53E-08 | Chr3 | 7933800 | 7961863 | 2519 |
| MsG0280007192.01 | 1.730741 | 7.78E-10 | 8.94E-08 | Chr2 | 11680778 | 11683273 | 1134 |
| novel.6030 | 8.204633 | 7.81E-10 | 8.94E-08 | Chr6 | 1.01E+08 | 1.01E+08 | 521 |
| MsG0380014963.01 | -3.94533 | 7.93E-10 | 9.05E-08 | Chr3 | 63283537 | 63296166 | 5418 |
| novel.2971 | 4.433244 | 7.98E-10 | 9.05E-08 | Chr3 | 66500544 | 66503817 | 3274 |
| novel.1416 | -8.34857 | 7.99E-10 | 9.05E-08 | Chr2 | 45682561 | 45695139 | 1182 |
| novel.2391 | 8.153999 | 8.18E-10 | 9.23E-08 | Chr3 | 57372342 | 57373245 | 786 |
| MsG0180002749.01 | -2.88754 | 8.29E-10 | 9.32E-08 | Chr1 | 44388324 | 44392820 | 3816 |
| MsG0680035476.01 | -2.64858 | 8.82E-10 | 9.87E-08 | Chr6 | 1.06E+08 | 1.06E+08 | 870 |
| MsG0580028438.01 | -4.45058 | 8.94E-10 | 9.97E-08 | Chr5 | 80504949 | 80507004 | 1107 |
| novel.8486 | -3.24483 | 9.20E-10 | 1.02E-07 | Chr8 | 37668015 | 37781870 | 2574 |
| MsG0380014143.01 | 8.114203 | 9.61E-10 | 1.06E-07 | Chr3 | 50330393 | 50330776 | 384 |
| novel.7078 | -3.06058 | 9.72E-10 | 1.07E-07 | Chr7 | 64828800 | 64831359 | 2319 |
| novel.8793 | 7.827559 | 1.01E-09 | 1.10E-07 | contig107end | 60650 | 63478 | 899 |
| MsG0780039796.01 | 8.112838 | 1.01E-09 | 1.10E-07 | Chr7 | 69189151 | 69190260 | 1110 |
| novel.457 | 3.280619 | 1.04E-09 | 1.13E-07 | Chr1 | 83776282 | 83778710 | 1084 |
| MsG0180000415.01 | -3.2087 | 1.14E-09 | 1.24E-07 | Chr1 | 5703921 | 5705665 | 1050 |
| MsG0880043597.01 | -3.90335 | 1.14E-09 | 1.24E-07 | Chr8 | 27473551 | 27475946 | 1704 |
| novel.5508 | -7.19748 | 1.18E-09 | 1.28E-07 | Chr6 | 845469 | 847136 | 1668 |
| MsG0480023006.01 | 1.766884 | 1.23E-09 | 1.33E-07 | Chr4 | 79620525 | 79621844 | 1002 |
| MsG0780039352.01 | 4.831828 | 1.27E-09 | 1.36E-07 | Chr7 | 62555638 | 62560079 | 1602 |
| MsG0180006217.01 | 2.15245 | 1.27E-09 | 1.36E-07 | Chr1 | 1.02E+08 | 1.02E+08 | 384 |
| MsG0580024533.01 | 2.682727 | 1.29E-09 | 1.37E-07 | Chr5 | 6108837 | 6109588 | 525 |
| MsG0380012655.01 | -1.3336 | 1.31E-09 | 1.38E-07 | Chr3 | 21971181 | 21977340 | 1241 |
| MsG0680034764.01 | -3.39758 | 1.31E-09 | 1.38E-07 | Chr6 | 94173776 | 94179606 | 1317 |
| MsG0880044264.01 | 4.856009 | 1.31E-09 | 1.38E-07 | Chr8 | 38935714 | 38945325 | 2742 |
| MsG0180002138.01 | -2.03507 | 1.36E-09 | 1.43E-07 | Chr1 | 33505940 | 33506545 | 606 |
| MsG0380014092.01 | -4.40813 | 1.48E-09 | 1.54E-07 | Chr3 | 49574635 | 49577173 | 1185 |
| novel.1336 | 7.747158 | 1.48E-09 | 1.54E-07 | Chr2 | 29220635 | 29222425 | 634 |
| novel.4806 | -2.64332 | 1.48E-09 | 1.54E-07 | Chr5 | 96381521 | 96385718 | 2211 |
| MsG0780038991.01 | 3.059643 | 1.60E-09 | 1.66E-07 | Chr7 | 56496976 | 56499502 | 478 |
| MsG0780036105.01 | 3.97111 | 1.65E-09 | 1.71E-07 | Chr7 | 2663941 | 2667063 | 2712 |
| MsG0380013740.01 | -3.16184 | 1.73E-09 | 1.78E-07 | Chr3 | 44874967 | 44875631 | 665 |
| MsG0780036095.01 | -5.73176 | 1.74E-09 | 1.78E-07 | Chr7 | 2452362 | 2459891 | 1890 |
| novel.3975 | 8.281905 | 1.87E-09 | 1.91E-07 | Chr4 | 49094436 | 49098718 | 1275 |
| novel.4230 | 8.170255 | 1.87E-09 | 1.91E-07 | Chr4 | 87585761 | 87586474 | 714 |
| novel.7179 | 2.204834 | 1.91E-09 | 1.94E-07 | Chr7 | 79699635 | 79703047 | 1379 |
| novel.7371 | 3.375548 | 2.06E-09 | 2.08E-07 | Chr7 | 16613580 | 16615081 | 625 |
| MsG0380015915.01 | -7.74363 | 2.07E-09 | 2.08E-07 | Chr3 | 76585795 | 76589947 | 1101 |
| MsG0480019743.01 | 4.057085 | 2.11E-09 | 2.13E-07 | Chr4 | 26214520 | 26217224 | 2458 |
| novel.2875 | 8.104928 | 2.48E-09 | 2.49E-07 | Chr3 | 39909791 | 39911460 | 222 |
| novel.6984 | 7.969505 | 2.61E-09 | 2.61E-07 | Chr7 | 42168628 | 42169864 | 1091 |
| novel.8980 | -4.50928 | 2.71E-09 | 2.70E-07 | contig399end | 15145 | 18689 | 2171 |
| MsG0680032093.01 | -2.95719 | 2.76E-09 | 2.74E-07 | Chr6 | 33512109 | 33519109 | 3384 |
| MsG0480018585.01 | 2.23724 | 2.82E-09 | 2.79E-07 | Chr4 | 6957583 | 6960459 | 1647 |
| MsG0880041934.01 | 8.188107 | 2.99E-09 | 2.95E-07 | Chr8 | 1431337 | 1434768 | 1776 |
| MsG0780039923.01 | 7.960072 | 3.00E-09 | 2.95E-07 | Chr7 | 70510481 | 70517558 | 2442 |
| MsG0780036012.01 | -6.81964 | 3.12E-09 | 3.05E-07 | Chr7 | 1310257 | 1313429 | 744 |
| novel.4396 | -8.44333 | 3.14E-09 | 3.06E-07 | Chr5 | 17540842 | 17542174 | 1333 |
| MsG0680035722.01 | 4.231505 | 3.16E-09 | 3.07E-07 | Chr6 | 1.11E+08 | 1.11E+08 | 3675 |
| MsG0780039305.01 | -1.95116 | 3.20E-09 | 3.10E-07 | Chr7 | 61727932 | 61730909 | 1801 |
| MsG0880044218.01 | -8.60664 | 3.22E-09 | 3.11E-07 | Chr8 | 37825099 | 37826079 | 981 |
| MsG0580025210.01 | -1.97038 | 3.26E-09 | 3.14E-07 | Chr5 | 15360114 | 15361391 | 1278 |
| novel.1484 | -3.76227 | 3.27E-09 | 3.14E-07 | Chr2 | 60181156 | 60182907 | 1752 |
| novel.4329 | 1.853156 | 3.31E-09 | 3.17E-07 | Chr5 | 7465635 | 7470694 | 2896 |
| MsG0680033643.01 | 2.755094 | 3.38E-09 | 3.23E-07 | Chr6 | 70147087 | 70150144 | 1527 |
| MsG0480020488.01 | -3.11186 | 3.40E-09 | 3.24E-07 | Chr4 | 42336819 | 42338253 | 1071 |
| novel.5321 | -1.75081 | 3.62E-09 | 3.44E-07 | Chr5 | 82967534 | 82970778 | 2884 |
| novel.6121 | 7.936867 | 3.70E-09 | 3.50E-07 | Chr6 | 1.13E+08 | 1.13E+08 | 1395 |
| MsG0780036791.01 | -4.91076 | 3.77E-09 | 3.55E-07 | Chr7 | 13878443 | 13884334 | 3456 |
| MsG0180004133.01 | -6.48998 | 3.81E-09 | 3.58E-07 | Chr1 | 73802962 | 73807079 | 1689 |
| MsG0880042321.01 | -8.6289 | 3.83E-09 | 3.59E-07 | Chr8 | 7099158 | 7099751 | 453 |
| MsG0180001978.01 | -2.18713 | 3.94E-09 | 3.68E-07 | Chr1 | 30160317 | 30162865 | 636 |
| MsG0680030472.01 | 4.291535 | 4.04E-09 | 3.77E-07 | Chr6 | 3405997 | 3408797 | 486 |
| MsG0180003708.01 | 1.921603 | 4.13E-09 | 3.84E-07 | Chr1 | 67158725 | 67165380 | 1002 |
| MsG0880042995.01 | 2.984081 | 4.33E-09 | 4.01E-07 | Chr8 | 17312511 | 17315851 | 2721 |
| novel.2513 | 1.677573 | 4.42E-09 | 4.08E-07 | Chr3 | 80038946 | 80042206 | 3261 |
| novel.2501 | -4.34768 | 4.49E-09 | 4.13E-07 | Chr3 | 78830012 | 78832510 | 1187 |
| novel.3019 | 8.125607 | 4.77E-09 | 4.38E-07 | Chr3 | 74645346 | 74645864 | 391 |
| novel.907 | -5.72026 | 4.80E-09 | 4.39E-07 | Chr1 | 60546800 | 60547988 | 658 |
| novel.7361 | 6.754267 | 4.88E-09 | 4.45E-07 | Chr7 | 15325224 | 15327127 | 1904 |
| MsG0680031996.01 | 2.611534 | 4.93E-09 | 4.49E-07 | Chr6 | 31621350 | 31624616 | 3267 |
| novel.9205 | -1.5127 | 5.35E-09 | 4.85E-07 | contig547end | 504 | 9977 | 4022 |
| novel.291 | 8.135764 | 5.38E-09 | 4.86E-07 | Chr1 | 55084721 | 55089507 | 1641 |
| novel.9209 | -7.96749 | 5.44E-09 | 4.90E-07 | contig549end | 16136 | 22060 | 2425 |
| novel.1731 | -8.47113 | 5.67E-09 | 5.10E-07 | Chr2 | 11624038 | 11627686 | 743 |
| MsG0180004244.01 | 3.740295 | 5.83E-09 | 5.22E-07 | Chr1 | 75492036 | 75492659 | 624 |
| novel.5289 | -8.24149 | 5.92E-09 | 5.29E-07 | Chr5 | 75768937 | 75773248 | 1706 |
| novel.7424 | 8.136498 | 5.94E-09 | 5.30E-07 | Chr7 | 28179148 | 28180832 | 1541 |
| MsG0580026716.01 | 7.90044 | 6.02E-09 | 5.35E-07 | Chr5 | 42793331 | 42795082 | 714 |
| novel.152 | -3.19923 | 6.06E-09 | 5.37E-07 | Chr1 | 21214518 | 21723908 | 7524 |
| novel.1634 | 4.499884 | 6.27E-09 | 5.54E-07 | Chr2 | 83481977 | 83484969 | 2993 |
| MsG0580029554.01 | 7.974985 | 6.44E-09 | 5.67E-07 | Chr5 | 98465795 | 98477670 | 3918 |
| MsG0880047419.01 | 5.565407 | 6.47E-09 | 5.68E-07 | Chr8 | 86020786 | 86024992 | 870 |
| novel.7715 | 1.661579 | 6.64E-09 | 5.81E-07 | Chr7 | 85198231 | 85200577 | 528 |
| novel.7068 | -8.33387 | 6.82E-09 | 5.95E-07 | Chr7 | 63170482 | 63184581 | 816 |
| MsG0380012947.01 | -2.85674 | 7.01E-09 | 6.10E-07 | Chr3 | 27456646 | 27457047 | 402 |
| novel.3822 | -7.88177 | 7.18E-09 | 6.23E-07 | Chr4 | 14083632 | 14087090 | 1788 |
| novel.142 | -5.35002 | 7.22E-09 | 6.25E-07 | Chr1 | 18628779 | 18632842 | 1855 |
| novel.8779 | -3.70107 | 7.26E-09 | 6.27E-07 | Chr8 | 90425407 | 90427410 | 1039 |
| MsG0180005673.01 | -3.51601 | 7.37E-09 | 6.34E-07 | Chr1 | 94796143 | 94796997 | 855 |
| novel.8018 | 7.868064 | 7.49E-09 | 6.43E-07 | Chr8 | 42992150 | 42997118 | 1890 |
| novel.2284 | 4.700471 | 7.61E-09 | 6.52E-07 | Chr3 | 27701600 | 27710175 | 2835 |
| novel.8422 | -8.15965 | 7.70E-09 | 6.58E-07 | Chr8 | 23769166 | 23772476 | 2408 |
| MsG0380011717.01 | 1.966642 | 7.82E-09 | 6.66E-07 | Chr3 | 3995383 | 4008287 | 1293 |
| MsG0380016160.01 | -2.84187 | 7.94E-09 | 6.73E-07 | Chr3 | 79860909 | 79864890 | 594 |
| novel.5963 | 7.817915 | 7.95E-09 | 6.73E-07 | Chr6 | 90586992 | 90589152 | 882 |
| MsG0080048052.01 | -7.84236 | 8.25E-09 | 6.97E-07 | contig181end | 13573 | 16988 | 1131 |
| MsG0780036014.01 | -2.91766 | 8.65E-09 | 7.28E-07 | Chr7 | 1318655 | 1322183 | 1455 |
| MsG0480020961.01 | -3.23604 | 8.72E-09 | 7.32E-07 | Chr4 | 51246975 | 51257705 | 6223 |
| MsG0380013897.01 | -3.00989 | 8.87E-09 | 7.42E-07 | Chr3 | 47657739 | 47661102 | 1571 |
| MsG0180003688.01 | 1.958414 | 9.07E-09 | 7.57E-07 | Chr1 | 66629757 | 66630167 | 411 |
| MsG0480018434.01 | -4.76967 | 9.10E-09 | 7.58E-07 | Chr4 | 4823307 | 4831174 | 3052 |
| novel.7216 | 6.088766 | 9.81E-09 | 8.15E-07 | Chr7 | 84876048 | 84881984 | 1160 |
| novel.2473 | 4.299937 | 1.00E-08 | 8.29E-07 | Chr3 | 74421840 | 74425480 | 1653 |
| novel.2697 | -7.99456 | 1.02E-08 | 8.39E-07 | Chr3 | 4302217 | 4304576 | 1437 |
| MsG0480018371.01 | 2.558235 | 1.03E-08 | 8.49E-07 | Chr4 | 3950885 | 3952516 | 1018 |
| MsG0880042388.01 | 7.776988 | 1.07E-08 | 8.79E-07 | Chr8 | 7962518 | 7968301 | 1521 |
| novel.1426 | 5.285417 | 1.09E-08 | 8.97E-07 | Chr2 | 48470054 | 48472670 | 1537 |
| novel.8090 | -3.26462 | 1.11E-08 | 9.09E-07 | Chr8 | 57698653 | 57701073 | 2421 |
| MsG0580025535.01 | 7.788727 | 1.14E-08 | 9.29E-07 | Chr5 | 20156870 | 20160578 | 1875 |
| MsG0280009948.01 | -3.84796 | 1.15E-08 | 9.32E-07 | Chr2 | 61541022 | 61549138 | 1811 |
| MsG0280009252.01 | 7.91685 | 1.18E-08 | 9.56E-07 | Chr2 | 48722401 | 48724144 | 1038 |
| novel.376 | 7.813932 | 1.18E-08 | 9.58E-07 | Chr1 | 68029606 | 68031882 | 620 |
| novel.2282 | -7.73572 | 1.20E-08 | 9.71E-07 | Chr3 | 27184500 | 27186814 | 2315 |
| MsG0180005650.01 | -5.55257 | 1.22E-08 | 9.79E-07 | Chr1 | 94486855 | 94492700 | 1416 |
| novel.8190 | 7.79027 | 1.25E-08 | 1.00E-06 | Chr8 | 74824503 | 74825672 | 1170 |
| novel.1485 | 2.20592 | 1.25E-08 | 1.00E-06 | Chr2 | 60283466 | 60286755 | 916 |
| novel.2109 | 7.759799 | 1.25E-08 | 1.00E-06 | Chr2 | 83486906 | 83489053 | 1420 |
| novel.481 | -5.19118 | 1.26E-08 | 1.00E-06 | Chr1 | 85876995 | 85880277 | 2646 |
| MsG0180000980.01 | 2.064138 | 1.31E-08 | 1.04E-06 | Chr1 | 14042475 | 14046458 | 505 |
| novel.5541 | -4.21224 | 1.32E-08 | 1.05E-06 | Chr6 | 5595809 | 5602858 | 5612 |
| novel.6851 | 7.886049 | 1.38E-08 | 1.09E-06 | Chr7 | 15058884 | 15060353 | 986 |
| MsG0280008658.01 | 2.642039 | 1.41E-08 | 1.11E-06 | Chr2 | 35998823 | 35999986 | 1164 |
| novel.2761 | -3.84192 | 1.41E-08 | 1.11E-06 | Chr3 | 13974073 | 13979082 | 3507 |
| novel.3068 | -2.41655 | 1.43E-08 | 1.12E-06 | Chr3 | 80975776 | 80980956 | 5181 |
| MsG0280006300.01 | 3.05243 | 1.44E-08 | 1.13E-06 | Chr2 | 194974 | 197210 | 531 |
| MsG0880047129.01 | -8.11565 | 1.62E-08 | 1.26E-06 | Chr8 | 82391100 | 82397197 | 2931 |
| novel.3261 | -2.24891 | 1.65E-08 | 1.28E-06 | Chr4 | 6730122 | 6737729 | 2350 |
| MsG0380014859.01 | -8.0546 | 1.68E-08 | 1.31E-06 | Chr3 | 61465240 | 61467837 | 1653 |
| MsG0780038118.01 | -3.33306 | 1.77E-08 | 1.37E-06 | Chr7 | 40602906 | 40604054 | 1149 |
| MsG0780038990.01 | 5.687149 | 1.81E-08 | 1.39E-06 | Chr7 | 56489525 | 56489713 | 189 |
| MsG0480018530.01 | -8.00497 | 1.84E-08 | 1.41E-06 | Chr4 | 6195049 | 6200062 | 711 |
| novel.2088 | -6.50596 | 1.93E-08 | 1.49E-06 | Chr2 | 81622465 | 81626167 | 2999 |
| MsG0380013898.01 | -2.45679 | 1.95E-08 | 1.49E-06 | Chr3 | 47706383 | 47710234 | 1637 |
| MsG0680032833.01 | -2.1287 | 1.97E-08 | 1.50E-06 | Chr6 | 50649105 | 50652776 | 2973 |
| MsG0480019065.01 | -2.06366 | 1.97E-08 | 1.50E-06 | Chr4 | 14072610 | 14083566 | 5339 |
| novel.4747 | -3.55743 | 2.02E-08 | 1.53E-06 | Chr5 | 86035759 | 86039086 | 2512 |
| novel.6853 | -5.03644 | 2.04E-08 | 1.55E-06 | Chr7 | 15504209 | 15509523 | 5315 |
| MsG0280011119.01 | 5.479601 | 2.06E-08 | 1.55E-06 | Chr2 | 79767846 | 79768724 | 657 |
| novel.4465 | 7.688961 | 2.07E-08 | 1.56E-06 | Chr5 | 27848815 | 27851767 | 783 |
| MsG0580029312.01 | 2.6809 | 2.09E-08 | 1.57E-06 | Chr5 | 95159510 | 95164869 | 678 |
| MsG0380014361.01 | 1.788943 | 2.12E-08 | 1.59E-06 | Chr3 | 53502105 | 53503884 | 862 |
| novel.5916 | -1.50809 | 2.13E-08 | 1.60E-06 | Chr6 | 81905251 | 81914608 | 1047 |
| MsG0480018927.01 | -3.77458 | 2.15E-08 | 1.61E-06 | Chr4 | 11866277 | 11877572 | 3075 |
| MsG0880043572.01 | 2.28657 | 2.23E-08 | 1.66E-06 | Chr8 | 27008674 | 27011466 | 492 |
| MsG0880044624.01 | 3.741969 | 2.28E-08 | 1.69E-06 | Chr8 | 45871633 | 45873471 | 1263 |
| MsG0480018653.01 | -6.24091 | 2.35E-08 | 1.74E-06 | Chr4 | 8058379 | 8059623 | 507 |
| novel.2950 | -4.26714 | 2.41E-08 | 1.79E-06 | Chr3 | 61090150 | 61095190 | 2247 |
| novel.5055 | -6.57241 | 2.45E-08 | 1.81E-06 | Chr5 | 28490392 | 28493834 | 1062 |
| MsG0280006598.01 | 7.784815 | 2.59E-08 | 1.91E-06 | Chr2 | 3856943 | 3859401 | 1878 |
| MsG0480019308.01 | -4.59104 | 2.65E-08 | 1.94E-06 | Chr4 | 18300473 | 18300760 | 288 |
| MsG0580025992.01 | 1.514081 | 2.68E-08 | 1.97E-06 | Chr5 | 28759291 | 28762815 | 875 |
| MsG0680034725.01 | 1.675574 | 2.93E-08 | 2.14E-06 | Chr6 | 93467241 | 93468590 | 498 |
| MsG0380014090.01 | -8.32091 | 2.93E-08 | 2.14E-06 | Chr3 | 49533453 | 49540361 | 1541 |
| MsG0380015960.01 | -1.88088 | 2.96E-08 | 2.15E-06 | Chr3 | 77253191 | 77255744 | 1629 |
| novel.7008 | -5.85611 | 2.96E-08 | 2.15E-06 | Chr7 | 48190142 | 48191491 | 781 |
| MsG0480022662.01 | 4.064781 | 3.02E-08 | 2.19E-06 | Chr4 | 75222155 | 75225878 | 1062 |
| novel.3095 | 7.634893 | 3.06E-08 | 2.21E-06 | Chr3 | 86399485 | 86402485 | 1366 |
| novel.785 | -7.73715 | 3.09E-08 | 2.22E-06 | Chr1 | 27688044 | 27689957 | 1914 |
| novel.4418 | -7.67137 | 3.13E-08 | 2.25E-06 | Chr5 | 21935582 | 21939477 | 995 |
| novel.2538 | -7.98947 | 3.21E-08 | 2.30E-06 | Chr3 | 84789404 | 84791382 | 1928 |
| novel.9023 | -2.62886 | 3.26E-08 | 2.33E-06 | contig442end | 18902 | 24100 | 4273 |
| MsG0780039121.01 | 2.952004 | 3.36E-08 | 2.40E-06 | Chr7 | 58256209 | 58263813 | 3496 |
| novel.6182 | 7.87213 | 3.37E-08 | 2.40E-06 | Chr6 | 7448432 | 7451056 | 2070 |
| MsG0080049135.01 | 1.705288 | 3.40E-08 | 2.41E-06 | contig91end | 3012 | 3607 | 489 |
| novel.8223 | -2.99048 | 3.67E-08 | 2.60E-06 | Chr8 | 81550561 | 81556627 | 3503 |
| novel.5121 | -6.81897 | 3.77E-08 | 2.67E-06 | Chr5 | 43702461 | 43704897 | 2074 |
| novel.5615 | -11.0089 | 3.82E-08 | 2.70E-06 | Chr6 | 17364158 | 17369555 | 1508 |
| MsG0280007801.01 | 3.489562 | 3.92E-08 | 2.76E-06 | Chr2 | 20753917 | 20756141 | 2105 |
| MsG0180003755.01 | -7.35259 | 3.93E-08 | 2.76E-06 | Chr1 | 67708991 | 67713280 | 639 |
| novel.8530 | 3.835814 | 3.94E-08 | 2.76E-06 | Chr8 | 48000752 | 48002424 | 1045 |
| MsG0780041101.01 | 2.038256 | 4.02E-08 | 2.81E-06 | Chr7 | 86605146 | 86606198 | 612 |
| MsG0380013732.01 | -7.66362 | 4.13E-08 | 2.88E-06 | Chr3 | 44722220 | 44728900 | 1026 |
| novel.42 | -4.28403 | 4.21E-08 | 2.93E-06 | Chr1 | 5782930 | 5785939 | 2342 |
| novel.5581 | -7.72681 | 4.32E-08 | 3.00E-06 | Chr6 | 12200568 | 12205129 | 1366 |
| MsG0880044518.01 | 7.72563 | 4.39E-08 | 3.04E-06 | Chr8 | 43614130 | 43618587 | 960 |
| MsG0480022067.01 | -5.68925 | 4.50E-08 | 3.11E-06 | Chr4 | 67652374 | 67655557 | 2310 |
| novel.3179 | 8.37066 | 4.59E-08 | 3.17E-06 | Chr3 | 99177411 | 99183202 | 2204 |
| novel.8335 | 2.708022 | 4.64E-08 | 3.19E-06 | Chr8 | 7179978 | 7183701 | 764 |
| MsG0180000657.01 | -5.42984 | 4.90E-08 | 3.37E-06 | Chr1 | 9311781 | 9314349 | 1044 |
| novel.8708 | -6.40322 | 4.99E-08 | 3.42E-06 | Chr8 | 79854505 | 79857507 | 2395 |
| MsG0180002793.01 | 7.173087 | 5.00E-08 | 3.42E-06 | Chr1 | 45222684 | 45225023 | 1485 |
| MsG0380013009.01 | -3.7404 | 5.11E-08 | 3.49E-06 | Chr3 | 28635711 | 28636071 | 361 |
| MsG0780037655.01 | 2.017298 | 5.20E-08 | 3.53E-06 | Chr7 | 30366072 | 30372119 | 1196 |
| MsG0580029337.01 | -5.05492 | 5.20E-08 | 3.53E-06 | Chr5 | 95610598 | 95620532 | 3435 |
| novel.4987 | 8.426857 | 5.26E-08 | 3.57E-06 | Chr5 | 13975469 | 13980432 | 1278 |
| novel.5432 | -3.88409 | 5.47E-08 | 3.70E-06 | Chr5 | 99863618 | 99866697 | 1343 |
| MsG0580025806.01 | -7.88871 | 5.58E-08 | 3.77E-06 | Chr5 | 24482553 | 24483488 | 936 |
| MsG0780039677.01 | 4.232037 | 5.60E-08 | 3.77E-06 | Chr7 | 67449432 | 67449755 | 324 |
| MsG0480018327.01 | -3.67986 | 5.63E-08 | 3.78E-06 | Chr4 | 3406949 | 3409188 | 591 |
| MsG0680033602.01 | 2.245187 | 5.64E-08 | 3.78E-06 | Chr6 | 69236500 | 69239377 | 2187 |
| MsG0680032086.01 | -7.74044 | 5.78E-08 | 3.86E-06 | Chr6 | 33406475 | 33407547 | 975 |
| novel.2073 | 5.335518 | 5.87E-08 | 3.92E-06 | Chr2 | 79761338 | 79767466 | 919 |
| MsG0580029296.01 | -2.09926 | 5.99E-08 | 3.99E-06 | Chr5 | 94847462 | 94857200 | 2475 |
| novel.8218 | 1.545254 | 6.07E-08 | 4.03E-06 | Chr8 | 80112697 | 80113380 | 412 |
| MsG0680035114.01 | -3.83067 | 6.10E-08 | 4.04E-06 | Chr6 | 99183554 | 99193870 | 4401 |
| MsG0480023599.01 | -4.6736 | 6.11E-08 | 4.04E-06 | Chr4 | 87481293 | 87481631 | 339 |
| novel.8391 | 1.965877 | 6.16E-08 | 4.07E-06 | Chr8 | 17390072 | 17395214 | 2415 |
| MsG0880047250.01 | -4.10941 | 6.19E-08 | 4.07E-06 | Chr8 | 83785388 | 83791223 | 1608 |
| novel.2176 | -2.31637 | 6.19E-08 | 4.07E-06 | Chr3 | 9049669 | 9054781 | 4571 |
| MsG0680035907.01 | -5.1134 | 6.21E-08 | 4.07E-06 | Chr6 | 1.14E+08 | 1.14E+08 | 639 |
| novel.5044 | -2.07594 | 6.41E-08 | 4.20E-06 | Chr5 | 26796466 | 26802338 | 3037 |
| MsG0880043851.01 | 2.136352 | 6.44E-08 | 4.21E-06 | Chr8 | 31577765 | 31583164 | 4410 |
| MsG0580025867.01 | 1.946556 | 6.75E-08 | 4.40E-06 | Chr5 | 25483504 | 25494630 | 1236 |
| novel.7061 | -7.18008 | 6.80E-08 | 4.40E-06 | Chr7 | 61098395 | 61101178 | 1765 |
| MsG0580030244.01 | -7.81637 | 6.80E-08 | 4.40E-06 | Chr5 | 1.09E+08 | 1.09E+08 | 1131 |
| novel.4100 | -2.50916 | 6.80E-08 | 4.40E-06 | Chr4 | 69079682 | 69084241 | 2135 |
| MsG0880042478.01 | -3.20274 | 7.02E-08 | 4.54E-06 | Chr8 | 9358253 | 9362877 | 3099 |
| MsG0380017536.01 | -2.57015 | 7.23E-08 | 4.66E-06 | Chr3 | 97623642 | 97628844 | 1088 |
| MsG0780038632.01 | 7.055049 | 7.55E-08 | 4.86E-06 | Chr7 | 50181998 | 50187272 | 2154 |
| MsG0480018682.01 | -3.78744 | 7.58E-08 | 4.86E-06 | Chr4 | 8545554 | 8547701 | 1776 |
| MsG0580024823.01 | 2.46503 | 7.83E-08 | 5.02E-06 | Chr5 | 10302020 | 10304622 | 1164 |
| MsG0880043966.01 | 7.224963 | 8.17E-08 | 5.23E-06 | Chr8 | 33522251 | 33522433 | 183 |
| MsG0880046916.01 | -4.71764 | 8.38E-08 | 5.35E-06 | Chr8 | 79819217 | 79825877 | 963 |
| MsG0180001547.01 | -2.38231 | 8.52E-08 | 5.43E-06 | Chr1 | 23055279 | 23056699 | 1332 |
| MsG0880043249.01 | -1.39892 | 8.63E-08 | 5.49E-06 | Chr8 | 21251444 | 21264633 | 2052 |
| novel.830 | -7.73293 | 8.67E-08 | 5.50E-06 | Chr1 | 38018734 | 38020537 | 1804 |
| MsG0680035608.01 | -4.12611 | 8.75E-08 | 5.54E-06 | Chr6 | 1.09E+08 | 1.09E+08 | 658 |
| novel.2536 | -7.88715 | 8.77E-08 | 5.54E-06 | Chr3 | 84351930 | 84354954 | 1960 |
| MsG0380013508.01 | 1.822267 | 9.05E-08 | 5.70E-06 | Chr3 | 39698986 | 39705945 | 1931 |
| novel.9040 | -7.51599 | 9.25E-08 | 5.81E-06 | contig451end | 29553 | 30494 | 664 |
| MsG0880044564.01 | 3.033632 | 9.35E-08 | 5.87E-06 | Chr8 | 44582106 | 44582542 | 345 |
| MsG0480021535.01 | 7.447889 | 9.50E-08 | 5.95E-06 | Chr4 | 59223830 | 59233060 | 1518 |
| novel.2699 | -3.91204 | 9.54E-08 | 5.96E-06 | Chr3 | 5112750 | 5124462 | 804 |
| novel.8816 | 7.157562 | 9.74E-08 | 6.07E-06 | contig152end | 94869 | 95878 | 619 |
| MsG0880042133.01 | -2.34388 | 9.87E-08 | 6.14E-06 | Chr8 | 4182930 | 4183277 | 348 |
| novel.2333 | 2.348527 | 9.90E-08 | 6.15E-06 | Chr3 | 41535660 | 41539755 | 1604 |
| novel.2537 | -7.97058 | 1.04E-07 | 6.43E-06 | Chr3 | 84355623 | 84357972 | 1567 |
| novel.6067 | -7.8371 | 1.06E-07 | 6.59E-06 | Chr6 | 1.05E+08 | 1.05E+08 | 1652 |
| novel.1423 | 3.572656 | 1.08E-07 | 6.69E-06 | Chr2 | 47854137 | 47858593 | 4140 |
| novel.1862 | -6.22722 | 1.11E-07 | 6.82E-06 | Chr2 | 36199167 | 36200506 | 1002 |
| MsG0280008668.01 | -2.50484 | 1.12E-07 | 6.88E-06 | Chr2 | 36091346 | 36094300 | 966 |
| MsG0780041608.01 | -2.5938 | 1.13E-07 | 6.91E-06 | Chr7 | 92980547 | 92983955 | 1143 |
| novel.696 | 3.982533 | 1.16E-07 | 7.11E-06 | Chr1 | 11694457 | 11695969 | 1513 |
| novel.6168 | 7.57535 | 1.19E-07 | 7.25E-06 | Chr6 | 5643627 | 5644844 | 998 |
| MsG0480022413.01 | -1.09115 | 1.19E-07 | 7.25E-06 | Chr4 | 72245059 | 72247479 | 1276 |
| novel.74 | -7.48481 | 1.23E-07 | 7.49E-06 | Chr1 | 9778232 | 9779474 | 811 |
| MsG0380013819.01 | 7.905192 | 1.23E-07 | 7.49E-06 | Chr3 | 46075441 | 46076115 | 675 |
| MsG0380017477.01 | -2.3982 | 1.23E-07 | 7.49E-06 | Chr3 | 96693663 | 96696521 | 714 |
| novel.309 | 2.401637 | 1.28E-07 | 7.76E-06 | Chr1 | 58018610 | 58021595 | 1086 |
| MsG0380017426.01 | 2.66524 | 1.29E-07 | 7.81E-06 | Chr3 | 96171235 | 96172336 | 750 |
| novel.4696 | -7.9368 | 1.30E-07 | 7.82E-06 | Chr5 | 77237151 | 77249547 | 776 |
| MsG0480022039.01 | 1.73424 | 1.32E-07 | 7.96E-06 | Chr4 | 67268508 | 67271317 | 1260 |
| novel.4840 | -4.0776 | 1.34E-07 | 8.04E-06 | Chr5 | 1E+08 | 1E+08 | 2862 |
| novel.6715 | -7.71507 | 1.38E-07 | 8.26E-06 | Chr6 | 1.04E+08 | 1.04E+08 | 886 |
| novel.6893 | 7.642636 | 1.38E-07 | 8.26E-06 | Chr7 | 21886554 | 21888554 | 1691 |
| MsG0180000068.01 | -2.27066 | 1.42E-07 | 8.46E-06 | Chr1 | 1028575 | 1029984 | 1410 |
| MsG0780039296.01 | 7.608278 | 1.42E-07 | 8.46E-06 | Chr7 | 61498757 | 61499041 | 285 |
| MsG0180004262.01 | -3.31282 | 1.45E-07 | 8.62E-06 | Chr1 | 75700375 | 75706294 | 2134 |
| MsG0580025208.01 | -3.48461 | 1.52E-07 | 8.99E-06 | Chr5 | 15339908 | 15341086 | 1179 |
| novel.4787 | -7.59314 | 1.52E-07 | 9.01E-06 | Chr5 | 92529449 | 92532876 | 865 |
| MsG0480022258.01 | -7.61432 | 1.54E-07 | 9.10E-06 | Chr4 | 70152012 | 70157691 | 636 |
| novel.5523 | -2.60322 | 1.57E-07 | 9.27E-06 | Chr6 | 2602789 | 2625651 | 4747 |
| novel.8445 | -8.15354 | 1.60E-07 | 9.45E-06 | Chr8 | 28869800 | 28878682 | 1939 |
| MsG0480022127.01 | -1.41225 | 1.64E-07 | 9.64E-06 | Chr4 | 68576114 | 68584938 | 2349 |
| novel.3692 | -7.66843 | 1.67E-07 | 9.81E-06 | Chr4 | 85714684 | 85716127 | 1201 |
| novel.3231 | 7.11028 | 1.68E-07 | 9.83E-06 | Chr4 | 3139995 | 3143656 | 1540 |
| MsG0880043450.01 | -1.77029 | 1.69E-07 | 9.86E-06 | Chr8 | 24982470 | 24988340 | 2956 |
| MsG0380012567.01 | 1.3915 | 1.71E-07 | 9.97E-06 | Chr3 | 19893776 | 19898942 | 2851 |
| novel.4553 | 5.57715 | 1.76E-07 | 1.02E-05 | Chr5 | 47395346 | 47397529 | 619 |
| novel.7690 | -2.24854 | 1.76E-07 | 1.02E-05 | Chr7 | 82436353 | 82443596 | 5763 |
| MsG0380016056.01 | 1.86143 | 1.77E-07 | 1.02E-05 | Chr3 | 78487086 | 78489907 | 1338 |
| MsG0480018780.01 | -3.09834 | 1.79E-07 | 1.03E-05 | Chr4 | 9675325 | 9678964 | 1464 |
| novel.4060 | 6.424326 | 1.82E-07 | 1.05E-05 | Chr4 | 63961548 | 63962003 | 456 |
| novel.2182 | -7.52437 | 1.83E-07 | 1.06E-05 | Chr3 | 9991736 | 9995710 | 1299 |
| MsG0680035646.01 | 3.952495 | 1.85E-07 | 1.06E-05 | Chr6 | 1.1E+08 | 1.1E+08 | 570 |
| novel.1377 | 5.181012 | 1.85E-07 | 1.06E-05 | Chr2 | 35126525 | 35129218 | 956 |
| MsG0880045709.01 | -3.82485 | 1.89E-07 | 1.09E-05 | Chr8 | 63081420 | 63081944 | 318 |
| novel.8577 | 4.767211 | 1.90E-07 | 1.09E-05 | Chr8 | 54659079 | 54661395 | 1911 |
| MsG0180003128.01 | -3.57084 | 1.91E-07 | 1.09E-05 | Chr1 | 57038003 | 57038725 | 381 |
| MsG0780039894.01 | -2.89619 | 1.91E-07 | 1.09E-05 | Chr7 | 70204958 | 70207630 | 2673 |
| MsG0380012244.01 | -4.07098 | 1.94E-07 | 1.11E-05 | Chr3 | 13234171 | 13236044 | 936 |
| novel.3028 | 4.2663 | 1.97E-07 | 1.12E-05 | Chr3 | 76058934 | 76061370 | 1816 |
| MsG0580024687.01 | -1.33887 | 1.98E-07 | 1.12E-05 | Chr5 | 8147677 | 8153282 | 2118 |
| novel.6459 | -2.69228 | 1.98E-07 | 1.12E-05 | Chr6 | 51868804 | 51872446 | 879 |
| MsG0380014464.01 | 1.934773 | 1.99E-07 | 1.12E-05 | Chr3 | 55210008 | 55211945 | 1644 |
| MsG0780038383.01 | 2.834125 | 2.05E-07 | 1.16E-05 | Chr7 | 45513579 | 45530264 | 700 |
| novel.6488 | -7.0925 | 2.07E-07 | 1.17E-05 | Chr6 | 59173169 | 59175354 | 1292 |
| MsG0280009933.01 | -1.39264 | 2.19E-07 | 1.23E-05 | Chr2 | 61211457 | 61221572 | 3346 |
| novel.54 | -5.42975 | 2.20E-07 | 1.23E-05 | Chr1 | 7266627 | 7276659 | 1628 |
| novel.1160 | 7.719304 | 2.21E-07 | 1.24E-05 | Chr2 | 394734 | 398508 | 795 |
| novel.3525 | -4.86903 | 2.22E-07 | 1.24E-05 | Chr4 | 59797568 | 59800116 | 1874 |
| MsG0880043584.01 | 3.842481 | 2.28E-07 | 1.27E-05 | Chr8 | 27292242 | 27294748 | 714 |
| MsG0180006013.01 | 1.11291 | 2.31E-07 | 1.29E-05 | Chr1 | 98819227 | 98821377 | 551 |
| MsG0380013773.01 | -3.60952 | 2.32E-07 | 1.29E-05 | Chr3 | 45536688 | 45540202 | 2250 |
| MsG0880043195.01 | -2.27946 | 2.33E-07 | 1.29E-05 | Chr8 | 20436408 | 20440565 | 1361 |
| MsG0280010695.01 | 4.038696 | 2.35E-07 | 1.30E-05 | Chr2 | 73481567 | 73485905 | 308 |
| novel.1191 | -4.48455 | 2.43E-07 | 1.34E-05 | Chr2 | 3733939 | 3736067 | 2129 |
| MsG0680034818.01 | 4.704015 | 2.48E-07 | 1.37E-05 | Chr6 | 95000026 | 95004241 | 2316 |
| MsG0780038492.01 | 1.413395 | 2.51E-07 | 1.38E-05 | Chr7 | 47651370 | 47656557 | 1286 |
| MsG0880045288.01 | -2.07463 | 2.52E-07 | 1.39E-05 | Chr8 | 56940239 | 56941871 | 876 |
| MsG0480018677.01 | -8.18837 | 2.53E-07 | 1.39E-05 | Chr4 | 8511170 | 8518715 | 822 |
| MsG0780040259.01 | -1.94198 | 2.65E-07 | 1.45E-05 | Chr7 | 75027930 | 75032934 | 1796 |
| novel.5135 | 7.380128 | 2.68E-07 | 1.47E-05 | Chr5 | 45266111 | 45267447 | 672 |
| novel.1678 | -2.70656 | 2.72E-07 | 1.48E-05 | Chr2 | 4066170 | 4087275 | 2817 |
| novel.3498 | 7.45085 | 2.76E-07 | 1.50E-05 | Chr4 | 56238620 | 56241655 | 551 |
| MsG0280007912.01 | -1.97161 | 2.83E-07 | 1.54E-05 | Chr2 | 22822348 | 22823495 | 474 |
| novel.8293 | -2.91374 | 2.87E-07 | 1.56E-05 | Chr8 | 90374793 | 90379167 | 2972 |
| novel.6608 | -7.38503 | 2.98E-07 | 1.61E-05 | Chr6 | 84765474 | 84770172 | 697 |
| novel.2981 | -3.22327 | 3.04E-07 | 1.65E-05 | Chr3 | 68576060 | 68579990 | 3931 |
| MsG0880044876.01 | 2.152289 | 3.09E-07 | 1.67E-05 | Chr8 | 50538719 | 50543993 | 2676 |
| MsG0380017276.01 | -7.57588 | 3.13E-07 | 1.69E-05 | Chr3 | 94376901 | 94380210 | 825 |
| novel.2702 | 7.886582 | 3.20E-07 | 1.72E-05 | Chr3 | 5415899 | 5418180 | 1789 |
| MsG0480020491.01 | -7.37153 | 3.26E-07 | 1.75E-05 | Chr4 | 42401956 | 42408071 | 1407 |
| MsG0080048732.01 | -2.47451 | 3.30E-07 | 1.77E-05 | contig408end | 187455 | 194243 | 3141 |
| MsG0680033552.01 | 7.427065 | 3.32E-07 | 1.78E-05 | Chr6 | 68020496 | 68028072 | 3741 |
| MsG0480021895.01 | -1.61269 | 3.36E-07 | 1.80E-05 | Chr4 | 65736726 | 65748483 | 2025 |
| MsG0880047599.01 | -8.13506 | 3.38E-07 | 1.80E-05 | Chr8 | 88558224 | 88558718 | 495 |
| MsG0580024209.01 | -2.07623 | 3.39E-07 | 1.80E-05 | Chr5 | 2058681 | 2085629 | 6336 |
| novel.7464 | -2.99837 | 3.39E-07 | 1.80E-05 | Chr7 | 36827050 | 36829171 | 1336 |
| novel.5400 | -5.1928 | 3.46E-07 | 1.84E-05 | Chr5 | 94815806 | 94818487 | 1292 |
| novel.1449 | 7.278321 | 3.47E-07 | 1.84E-05 | Chr2 | 52343233 | 52345405 | 1458 |
| MsG0880044640.01 | -2.65676 | 3.50E-07 | 1.85E-05 | Chr8 | 46103126 | 46106436 | 2337 |
| novel.6035 | -7.25913 | 3.55E-07 | 1.87E-05 | Chr6 | 1.02E+08 | 1.02E+08 | 1014 |
| MsG0680030451.01 | 4.339328 | 3.76E-07 | 1.98E-05 | Chr6 | 3133594 | 3137375 | 2162 |
| MsG0180002100.01 | -3.43365 | 3.85E-07 | 2.02E-05 | Chr1 | 32892562 | 32895039 | 730 |
| novel.5392 | -4.31042 | 3.85E-07 | 2.02E-05 | Chr5 | 93670188 | 93673041 | 1140 |
| novel.4782 | -6.87849 | 3.86E-07 | 2.03E-05 | Chr5 | 91586936 | 91590645 | 781 |
| MsG0580028392.01 | 1.597315 | 3.90E-07 | 2.04E-05 | Chr5 | 79833168 | 79834993 | 1143 |
| novel.7852 | -1.57551 | 3.95E-07 | 2.06E-05 | Chr8 | 10908380 | 10913411 | 2945 |
| MsG0380014449.01 | -3.84046 | 3.99E-07 | 2.08E-05 | Chr3 | 55002297 | 55004102 | 1574 |
| MsG0580025941.01 | -7.96373 | 4.03E-07 | 2.10E-05 | Chr5 | 27640093 | 27657708 | 1743 |
| MsG0580025293.01 | -3.17124 | 4.04E-07 | 2.10E-05 | Chr5 | 16592440 | 16598066 | 1638 |
| MsG0180003671.01 | -1.32819 | 4.10E-07 | 2.13E-05 | Chr1 | 66389051 | 66389461 | 411 |
| novel.4641 | -8.02544 | 4.19E-07 | 2.17E-05 | Chr5 | 66265182 | 66266531 | 1128 |
| novel.1221 | 6.404759 | 4.21E-07 | 2.18E-05 | Chr2 | 8749809 | 8752691 | 777 |
| novel.580 | -4.17379 | 4.23E-07 | 2.18E-05 | Chr1 | 569502 | 574904 | 532 |
| MsG0580024523.01 | 2.191146 | 4.23E-07 | 2.18E-05 | Chr5 | 5992516 | 6000856 | 2988 |
| MsG0880042420.01 | 4.971937 | 4.31E-07 | 2.22E-05 | Chr8 | 8477943 | 8479398 | 816 |
| MsG0780036626.01 | 2.063971 | 4.36E-07 | 2.24E-05 | Chr7 | 10875916 | 10877952 | 1923 |
| novel.3283 | 6.343123 | 4.37E-07 | 2.24E-05 | Chr4 | 10985541 | 10989346 | 3624 |
| MsG0480018369.01 | 3.557019 | 4.52E-07 | 2.31E-05 | Chr4 | 3927792 | 3933931 | 1511 |
| novel.6014 | 3.262209 | 4.58E-07 | 2.34E-05 | Chr6 | 99631912 | 99635783 | 1157 |
| novel.632 | 1.198489 | 4.66E-07 | 2.37E-05 | Chr1 | 4438238 | 4444173 | 5411 |
| novel.7132 | -7.65564 | 4.66E-07 | 2.37E-05 | Chr7 | 74313962 | 74315275 | 1314 |
| MsG0480018562.01 | -4.25375 | 4.71E-07 | 2.39E-05 | Chr4 | 6622356 | 6631813 | 5561 |
| novel.9239 | -3.26288 | 4.73E-07 | 2.40E-05 | contig564end | 12321 | 21558 | 2636 |
| novel.5369 | 7.306174 | 4.75E-07 | 2.41E-05 | Chr5 | 90225893 | 90227727 | 631 |
| novel.208 | -7.35266 | 4.88E-07 | 2.47E-05 | Chr1 | 30177827 | 30180521 | 2695 |
| MsG0180001476.01 | -2.10969 | 4.94E-07 | 2.49E-05 | Chr1 | 21956366 | 21964736 | 6330 |
| MsG0180000810.01 | 5.570094 | 4.94E-07 | 2.49E-05 | Chr1 | 11510604 | 11511764 | 1161 |
| novel.4693 | 7.328263 | 4.98E-07 | 2.51E-05 | Chr5 | 76731977 | 76732449 | 473 |
| MsG0880045634.01 | -2.81311 | 5.06E-07 | 2.54E-05 | Chr8 | 61995166 | 62008785 | 2925 |
| MsG0480019541.01 | 7.265674 | 5.07E-07 | 2.54E-05 | Chr4 | 21874801 | 21877225 | 1525 |
| novel.5007 | -7.9735 | 5.16E-07 | 2.58E-05 | Chr5 | 17152940 | 17154397 | 346 |
| MsG0180004953.01 | 1.415315 | 5.16E-07 | 2.58E-05 | Chr1 | 84910915 | 84913105 | 1187 |
| MsG0180002420.01 | 1.4751 | 5.19E-07 | 2.59E-05 | Chr1 | 38255112 | 38260250 | 2190 |
| MsG0780036817.01 | -2.26635 | 5.28E-07 | 2.63E-05 | Chr7 | 14391028 | 14403581 | 4077 |
| novel.6475 | 7.224618 | 5.29E-07 | 2.63E-05 | Chr6 | 55710322 | 55718765 | 641 |
| MsG0780038378.01 | -1.77978 | 5.30E-07 | 2.63E-05 | Chr7 | 45453751 | 45458088 | 1542 |
| MsG0580028882.01 | -2.56575 | 5.30E-07 | 2.63E-05 | Chr5 | 87992399 | 87993199 | 801 |
| novel.3803 | 4.247575 | 5.36E-07 | 2.66E-05 | Chr4 | 8112689 | 8113883 | 1081 |
| novel.5999 | -7.40404 | 5.41E-07 | 2.68E-05 | Chr6 | 97601244 | 97601955 | 622 |
| MsG0480018592.01 | -1.85798 | 5.48E-07 | 2.70E-05 | Chr4 | 7069659 | 7071925 | 1261 |
| MsG0380013360.01 | 3.421803 | 5.62E-07 | 2.77E-05 | Chr3 | 35892908 | 35897021 | 906 |
| novel.1571 | -6.17101 | 5.73E-07 | 2.82E-05 | Chr2 | 77325947 | 77329618 | 2509 |
| MsG0380012200.01 | 4.879617 | 5.76E-07 | 2.83E-05 | Chr3 | 12625329 | 12629077 | 3096 |
| MsG0580024549.01 | -1.92598 | 5.83E-07 | 2.86E-05 | Chr5 | 6273797 | 6274702 | 906 |
| novel.2464 | 3.877115 | 5.85E-07 | 2.86E-05 | Chr3 | 72320697 | 72324222 | 2145 |
| novel.4713 | -7.22751 | 5.86E-07 | 2.87E-05 | Chr5 | 79945624 | 79949825 | 2544 |
| MsG0880042998.01 | -7.16754 | 5.92E-07 | 2.89E-05 | Chr8 | 17353481 | 17353714 | 234 |
| MsG0680030335.01 | 7.746305 | 5.96E-07 | 2.90E-05 | Chr6 | 781119 | 786819 | 2094 |
| MsG0680034673.01 | 3.910085 | 5.96E-07 | 2.90E-05 | Chr6 | 92607313 | 92608352 | 984 |
| MsG0280007911.01 | -2.10917 | 6.03E-07 | 2.93E-05 | Chr2 | 22807138 | 22813071 | 1304 |
| novel.1890 | 1.823395 | 6.06E-07 | 2.94E-05 | Chr2 | 43635380 | 43637541 | 1346 |
| MsG0280007230.01 | -1.92358 | 6.16E-07 | 2.98E-05 | Chr2 | 12281535 | 12282191 | 657 |
| novel.5617 | -10.7964 | 6.21E-07 | 3.00E-05 | Chr6 | 17667117 | 17667738 | 622 |
| novel.8993 | -6.18784 | 6.24E-07 | 3.01E-05 | contig408end | 238225 | 241328 | 1845 |
| novel.816 | -7.80514 | 6.38E-07 | 3.07E-05 | Chr1 | 35154238 | 35156701 | 567 |
| MsG0280006600.01 | -2.68018 | 6.41E-07 | 3.08E-05 | Chr2 | 3867835 | 3882638 | 3426 |
| MsG0580028316.01 | -1.80395 | 6.42E-07 | 3.08E-05 | Chr5 | 78243669 | 78249445 | 999 |
| MsG0480022703.01 | 7.250115 | 6.47E-07 | 3.10E-05 | Chr4 | 75651221 | 75655812 | 1125 |
| MsG0880044047.01 | 4.499775 | 6.72E-07 | 3.22E-05 | Chr8 | 34750687 | 34751958 | 1272 |
| MsG0880042195.01 | -2.84755 | 6.80E-07 | 3.25E-05 | Chr8 | 5246740 | 5251994 | 3321 |
| novel.8447 | 7.252426 | 6.95E-07 | 3.32E-05 | Chr8 | 28993049 | 28996467 | 756 |
| MsG0380012330.01 | -7.12518 | 6.96E-07 | 3.32E-05 | Chr3 | 15012697 | 15013095 | 399 |
| novel.5214 | 7.640737 | 7.03E-07 | 3.35E-05 | Chr5 | 62812212 | 62819704 | 678 |
| MsG0680032912.01 | 7.193274 | 7.08E-07 | 3.36E-05 | Chr6 | 52755411 | 52759407 | 1605 |
| MsG0180004272.01 | -2.73238 | 7.11E-07 | 3.38E-05 | Chr1 | 75877748 | 75882434 | 865 |
| novel.6936 | 4.676717 | 7.13E-07 | 3.38E-05 | Chr7 | 30001896 | 30006211 | 1879 |
| novel.1927 | -6.03525 | 7.23E-07 | 3.42E-05 | Chr2 | 54636467 | 54638461 | 1239 |
| MsG0680034060.01 | 2.965074 | 7.26E-07 | 3.43E-05 | Chr6 | 79820796 | 79823156 | 447 |
| MsG0580029539.01 | 1.423587 | 7.28E-07 | 3.43E-05 | Chr5 | 98282728 | 98296088 | 1635 |
| novel.3836 | -7.02592 | 7.28E-07 | 3.43E-05 | Chr4 | 17188722 | 17190155 | 1119 |
| novel.8518 | 7.193779 | 7.35E-07 | 3.46E-05 | Chr8 | 46181431 | 46184374 | 1277 |
| novel.3420 | -8.00884 | 7.46E-07 | 3.49E-05 | Chr4 | 38279668 | 38280732 | 1065 |
| novel.4725 | -4.05319 | 7.46E-07 | 3.49E-05 | Chr5 | 82200064 | 82204818 | 1350 |
| MsG0580025378.01 | -7.64519 | 7.47E-07 | 3.49E-05 | Chr5 | 17680830 | 17681594 | 765 |
| MsG0680032516.01 | -1.74577 | 7.50E-07 | 3.51E-05 | Chr6 | 43556089 | 43559407 | 756 |
| MsG0280009533.01 | 6.866888 | 7.70E-07 | 3.59E-05 | Chr2 | 53854601 | 53859189 | 2847 |
| MsG0480023689.01 | -3.46736 | 7.71E-07 | 3.59E-05 | Chr4 | 88602341 | 88604347 | 2007 |
| novel.6726 | -5.62632 | 7.86E-07 | 3.66E-05 | Chr6 | 1.05E+08 | 1.05E+08 | 2206 |
| novel.2343 | -2.98366 | 8.03E-07 | 3.73E-05 | Chr3 | 44670109 | 44670605 | 497 |
| MsG0880044373.01 | -6.88788 | 8.06E-07 | 3.74E-05 | Chr8 | 40966194 | 40975918 | 2481 |
| MsG0180002506.01 | -2.07435 | 8.07E-07 | 3.74E-05 | Chr1 | 39350395 | 39358538 | 2776 |
| MsG0880046415.01 | 1.741985 | 8.26E-07 | 3.82E-05 | Chr8 | 73170419 | 73170844 | 426 |
| novel.9357 | -2.17178 | 8.31E-07 | 3.84E-05 | contig643end | 15898 | 21554 | 2636 |
| MsG0380017349.01 | -1.91969 | 8.38E-07 | 3.86E-05 | Chr3 | 95251115 | 95256386 | 1553 |
| MsG0380011962.01 | 4.246292 | 8.42E-07 | 3.87E-05 | Chr3 | 8507345 | 8509702 | 812 |
| novel.6337 | -7.64333 | 8.57E-07 | 3.94E-05 | Chr6 | 27437234 | 27438284 | 912 |
| novel.2164 | -3.39953 | 8.81E-07 | 4.04E-05 | Chr3 | 7743071 | 7744051 | 981 |
| novel.3083 | 5.210697 | 8.86E-07 | 4.06E-05 | Chr3 | 84287751 | 84290299 | 1179 |
| MsG0880047750.01 | -5.0139 | 8.92E-07 | 4.08E-05 | Chr8 | 90399380 | 90405439 | 2126 |
| MsG0780036926.01 | -6.22324 | 8.94E-07 | 4.09E-05 | Chr7 | 16635166 | 16645880 | 2109 |
| MsG0480018539.01 | 2.762156 | 8.97E-07 | 4.09E-05 | Chr4 | 6323225 | 6349760 | 9552 |
| MsG0380017450.01 | -8.54673 | 9.27E-07 | 4.22E-05 | Chr3 | 96381869 | 96386711 | 2097 |
| MsG0680031580.01 | 7.234526 | 9.30E-07 | 4.23E-05 | Chr6 | 22599573 | 22605147 | 1446 |
| MsG0180002830.01 | -4.17314 | 9.44E-07 | 4.29E-05 | Chr1 | 46142323 | 46146864 | 3063 |
| novel.6021 | 7.188459 | 9.50E-07 | 4.31E-05 | Chr6 | 1E+08 | 1E+08 | 672 |
| novel.8309 | -7.35205 | 9.52E-07 | 4.31E-05 | Chr8 | 2255861 | 2257991 | 2131 |
| MsG0180003346.01 | 7.543863 | 9.59E-07 | 4.34E-05 | Chr1 | 60938875 | 60955567 | 3978 |
| novel.3804 | 7.20325 | 9.67E-07 | 4.37E-05 | Chr4 | 8288351 | 8291525 | 3175 |
| MsG0880045094.01 | 2.451506 | 9.72E-07 | 4.38E-05 | Chr8 | 54118545 | 54122546 | 2255 |
| novel.4148 | -7.79191 | 9.76E-07 | 4.39E-05 | Chr4 | 77260316 | 77261591 | 1179 |
| MsG0780040955.01 | 7.60584 | 9.82E-07 | 4.41E-05 | Chr7 | 84743930 | 84750397 | 3876 |
| MsG0380017436.01 | 1.428147 | 9.89E-07 | 4.44E-05 | Chr3 | 96232992 | 96237904 | 867 |
| MsG0380013726.01 | -2.8791 | 1.01E-06 | 4.52E-05 | Chr3 | 44651333 | 44654991 | 541 |
| MsG0380014508.01 | -2.41684 | 1.01E-06 | 4.53E-05 | Chr3 | 55866758 | 55867425 | 556 |
| MsG0580029792.01 | 1.304397 | 1.02E-06 | 4.56E-05 | Chr5 | 1.02E+08 | 1.02E+08 | 588 |
| MsG0780037656.01 | 3.497108 | 1.02E-06 | 4.56E-05 | Chr7 | 30373752 | 30376463 | 832 |
| MsG0280007779.01 | -1.59577 | 1.05E-06 | 4.66E-05 | Chr2 | 20444259 | 20454205 | 1764 |
| novel.8511 | -7.48993 | 1.06E-06 | 4.74E-05 | Chr8 | 44504197 | 44506997 | 1582 |
| novel.4287 | -6.92123 | 1.09E-06 | 4.84E-05 | Chr5 | 1383602 | 1385015 | 1262 |
| novel.5094 | -3.51025 | 1.09E-06 | 4.86E-05 | Chr5 | 36535095 | 36538673 | 2918 |
| novel.6020 | -3.81793 | 1.11E-06 | 4.89E-05 | Chr6 | 1E+08 | 1E+08 | 1742 |
| MsG0480023343.01 | 4.155905 | 1.13E-06 | 5.01E-05 | Chr4 | 84026367 | 84030241 | 1053 |
| novel.2433 | -4.55888 | 1.16E-06 | 5.14E-05 | Chr3 | 66768003 | 66770487 | 2054 |
| novel.7465 | -7.19364 | 1.18E-06 | 5.22E-05 | Chr7 | 36950909 | 36951968 | 1060 |
| novel.3859 | -7.13424 | 1.20E-06 | 5.30E-05 | Chr4 | 21102164 | 21104894 | 1991 |
| MsG0780036701.01 | -1.84652 | 1.23E-06 | 5.42E-05 | Chr7 | 12106684 | 12109341 | 861 |
| novel.3272 | -2.8824 | 1.28E-06 | 5.61E-05 | Chr4 | 9065400 | 9068900 | 3501 |
| MsG0480018952.01 | -2.48989 | 1.29E-06 | 5.67E-05 | Chr4 | 12283694 | 12286264 | 1726 |
| MsG0380011545.01 | 6.629277 | 1.30E-06 | 5.70E-05 | Chr3 | 1225466 | 1226593 | 1128 |
| MsG0780035970.01 | -1.40471 | 1.33E-06 | 5.82E-05 | Chr7 | 808123 | 811749 | 510 |
| MsG0180004239.01 | 1.188211 | 1.33E-06 | 5.82E-05 | Chr1 | 75400931 | 75407378 | 1895 |
| novel.1922 | -7.11066 | 1.34E-06 | 5.85E-05 | Chr2 | 53962218 | 53963558 | 1189 |
| novel.1142 | -2.05345 | 1.37E-06 | 5.94E-05 | Chr1 | 1.01E+08 | 1.01E+08 | 1056 |
| MsG0580030226.01 | 2.078017 | 1.37E-06 | 5.94E-05 | Chr5 | 1.09E+08 | 1.09E+08 | 924 |
| MsG0880042199.01 | -3.61065 | 1.37E-06 | 5.94E-05 | Chr8 | 5272516 | 5275540 | 1365 |
| MsG0480020541.01 | -6.08038 | 1.38E-06 | 5.96E-05 | Chr4 | 43074675 | 43077248 | 2574 |
| MsG0480018245.01 | -7.26457 | 1.38E-06 | 5.96E-05 | Chr4 | 2117899 | 2120771 | 1161 |
| novel.8694 | 7.073446 | 1.39E-06 | 6.00E-05 | Chr8 | 76181375 | 76182146 | 772 |
| MsG0880043265.01 | -2.95224 | 1.40E-06 | 6.03E-05 | Chr8 | 21731264 | 21739278 | 3855 |
| novel.5511 | -4.38825 | 1.41E-06 | 6.08E-05 | Chr6 | 935545 | 938085 | 2541 |
| MsG0580026777.01 | 1.7001 | 1.45E-06 | 6.23E-05 | Chr5 | 44569594 | 44569908 | 315 |
| MsG0480020471.01 | -2.66807 | 1.47E-06 | 6.30E-05 | Chr4 | 42108878 | 42115774 | 3191 |
| MsG0080048825.01 | -4.42858 | 1.48E-06 | 6.33E-05 | contig468end | 24058 | 24639 | 582 |
| novel.4370 | -5.39961 | 1.49E-06 | 6.39E-05 | Chr5 | 14310483 | 14312640 | 797 |
| MsG0480018440.01 | -2.75648 | 1.50E-06 | 6.40E-05 | Chr4 | 4923716 | 4925842 | 1685 |
| MsG0380016168.01 | 7.651951 | 1.51E-06 | 6.44E-05 | Chr3 | 79940485 | 79942588 | 1236 |
| MsG0780036898.01 | -4.25922 | 1.52E-06 | 6.50E-05 | Chr7 | 15923322 | 15924717 | 777 |
| novel.839 | 2.704338 | 1.53E-06 | 6.51E-05 | Chr1 | 40318323 | 40320788 | 1594 |
| novel.4443 | -3.64453 | 1.54E-06 | 6.55E-05 | Chr5 | 25272540 | 25273716 | 451 |
| novel.8138 | -3.45393 | 1.55E-06 | 6.59E-05 | Chr8 | 64822069 | 64825675 | 3102 |
| MsG0180001488.01 | 1.274368 | 1.56E-06 | 6.63E-05 | Chr1 | 22161607 | 22170910 | 928 |
| MsG0780036126.01 | 2.750414 | 1.59E-06 | 6.74E-05 | Chr7 | 3128961 | 3139308 | 4806 |
| MsG0380016731.01 | 2.071034 | 1.60E-06 | 6.76E-05 | Chr3 | 87165037 | 87171264 | 1899 |
| MsG0480019501.01 | -1.4608 | 1.61E-06 | 6.79E-05 | Chr4 | 21327576 | 21329120 | 219 |
| MsG0380017489.01 | -2.7429 | 1.61E-06 | 6.79E-05 | Chr3 | 96906907 | 96907917 | 1011 |
| MsG0680035610.01 | -1.84004 | 1.62E-06 | 6.84E-05 | Chr6 | 1.09E+08 | 1.09E+08 | 2056 |
| MsG0480020229.01 | -1.28772 | 1.63E-06 | 6.85E-05 | Chr4 | 36937212 | 36941040 | 2943 |
| MsG0780039166.01 | -6.13938 | 1.65E-06 | 6.92E-05 | Chr7 | 58963496 | 58965039 | 795 |
| novel.4516 | 7.066145 | 1.66E-06 | 6.96E-05 | Chr5 | 41193855 | 41195745 | 1614 |
| MsG0880042433.01 | 2.111793 | 1.67E-06 | 6.98E-05 | Chr8 | 8619287 | 8625268 | 2858 |
| novel.3628 | -2.60093 | 1.68E-06 | 7.02E-05 | Chr4 | 77435350 | 77436864 | 1241 |
| novel.7537 | 7.035871 | 1.69E-06 | 7.07E-05 | Chr7 | 58435019 | 58437528 | 1536 |
| MsG0780040208.01 | 1.039378 | 1.71E-06 | 7.13E-05 | Chr7 | 74394507 | 74394917 | 411 |
| MsG0680030333.01 | -2.50275 | 1.72E-06 | 7.17E-05 | Chr6 | 750639 | 761385 | 207 |
| novel.7420 | -4.82872 | 1.72E-06 | 7.17E-05 | Chr7 | 27059332 | 27062227 | 2896 |
| MsG0580024178.01 | -3.26888 | 1.73E-06 | 7.19E-05 | Chr5 | 1596260 | 1598040 | 1428 |
| MsG0480019107.01 | -3.53491 | 1.73E-06 | 7.20E-05 | Chr4 | 14677113 | 14683155 | 4494 |
| MsG0380017256.01 | 6.561299 | 1.78E-06 | 7.40E-05 | Chr3 | 94103061 | 94118324 | 2652 |
| MsG0680035822.01 | -6.67663 | 1.79E-06 | 7.40E-05 | Chr6 | 1.13E+08 | 1.13E+08 | 1083 |
| MsG0680030640.01 | 3.983331 | 1.79E-06 | 7.42E-05 | Chr6 | 6496461 | 6497022 | 369 |
| novel.5486 | 4.359765 | 1.80E-06 | 7.44E-05 | Chr5 | 1.07E+08 | 1.07E+08 | 1153 |
| novel.551 | -5.2924 | 1.83E-06 | 7.53E-05 | Chr1 | 97344952 | 97348200 | 910 |
| MsG0880043085.01 | 4.094339 | 1.85E-06 | 7.60E-05 | Chr8 | 18718308 | 18721561 | 2294 |
| MsG0580024601.01 | -2.5602 | 1.88E-06 | 7.75E-05 | Chr5 | 6778516 | 6781139 | 1152 |
| MsG0580025502.01 | -1.23401 | 1.90E-06 | 7.80E-05 | Chr5 | 19685139 | 19695573 | 4171 |
| novel.4828 | -3.96236 | 1.91E-06 | 7.85E-05 | Chr5 | 99341678 | 99346718 | 1664 |
| MsG0280009050.01 | -7.41026 | 1.95E-06 | 7.99E-05 | Chr2 | 44348802 | 44352549 | 1230 |
| MsG0280006503.01 | 1.998209 | 1.96E-06 | 8.01E-05 | Chr2 | 2837503 | 2844883 | 619 |
| MsG0580028179.01 | -2.42677 | 1.97E-06 | 8.06E-05 | Chr5 | 76212633 | 76215987 | 2224 |
| MsG0880044263.01 | 2.611851 | 2.00E-06 | 8.17E-05 | Chr8 | 38926766 | 38931927 | 2022 |
| novel.828 | 7.443013 | 2.03E-06 | 8.26E-05 | Chr1 | 37640352 | 37642873 | 978 |
| novel.7925 | -1.47971 | 2.04E-06 | 8.30E-05 | Chr8 | 23352932 | 23355245 | 1399 |
| MsG0180000971.01 | 2.012074 | 2.06E-06 | 8.37E-05 | Chr1 | 13888783 | 13890840 | 1936 |
| MsG0180003465.01 | 3.215711 | 2.07E-06 | 8.39E-05 | Chr1 | 62667787 | 62668901 | 876 |
| MsG0680030965.01 | -3.14356 | 2.14E-06 | 8.65E-05 | Chr6 | 12255933 | 12258711 | 2643 |
| MsG0880046600.01 | 1.355345 | 2.14E-06 | 8.65E-05 | Chr8 | 75332071 | 75336432 | 910 |
| MsG0680031279.01 | -6.80399 | 2.18E-06 | 8.80E-05 | Chr6 | 17262435 | 17263190 | 438 |
| MsG0580024320.01 | -1.84218 | 2.18E-06 | 8.80E-05 | Chr5 | 3340511 | 3343973 | 1278 |
| MsG0180000985.01 | -6.28412 | 2.19E-06 | 8.82E-05 | Chr1 | 14082311 | 14085177 | 1032 |
| MsG0580029576.01 | 1.566424 | 2.22E-06 | 8.95E-05 | Chr5 | 98802499 | 98807205 | 1443 |
| MsG0680035158.01 | 7.150602 | 2.25E-06 | 9.07E-05 | Chr6 | 99852588 | 99855121 | 441 |
| novel.6781 | 1.717709 | 2.27E-06 | 9.12E-05 | Chr6 | 1.12E+08 | 1.12E+08 | 1145 |
| MsG0780037661.01 | 1.757572 | 2.31E-06 | 9.25E-05 | Chr7 | 30524960 | 30529183 | 766 |
| MsG0480019159.01 | 6.965886 | 2.31E-06 | 9.27E-05 | Chr4 | 15225608 | 15229582 | 1446 |
| MsG0380012800.01 | -2.24689 | 2.36E-06 | 9.44E-05 | Chr3 | 24776023 | 24776346 | 324 |
| novel.2318 | 5.012183 | 2.36E-06 | 9.44E-05 | Chr3 | 36885937 | 36888216 | 663 |
| MsG0580025807.01 | -3.33361 | 2.42E-06 | 9.65E-05 | Chr5 | 24499071 | 24509647 | 1749 |
| MsG0180000760.01 | -6.49903 | 2.42E-06 | 9.65E-05 | Chr1 | 10737066 | 10737359 | 294 |
| MsG0480020072.01 | 1.952154 | 2.43E-06 | 9.65E-05 | Chr4 | 33385373 | 33390609 | 1525 |
| novel.5116 | -2.89016 | 2.43E-06 | 9.65E-05 | Chr5 | 43106088 | 43109509 | 3341 |
| MsG0680034967.01 | 3.09642 | 2.44E-06 | 9.68E-05 | Chr6 | 97170964 | 97172417 | 1110 |
| MsG0680030607.01 | -1.54915 | 2.44E-06 | 9.68E-05 | Chr6 | 6010840 | 6014811 | 1071 |
| MsG0380015519.01 | -3.71095 | 2.45E-06 | 9.70E-05 | Chr3 | 71318988 | 71321475 | 947 |
| novel.90 | 3.800723 | 2.45E-06 | 9.70E-05 | Chr1 | 11673091 | 11675249 | 1959 |
| novel.2790 | 4.200968 | 2.46E-06 | 9.70E-05 | Chr3 | 22073872 | 22076435 | 1353 |
| novel.5848 | -5.24853 | 2.50E-06 | 9.84E-05 | Chr6 | 69790045 | 69795260 | 1879 |
| MsG0580030147.01 | -7.11372 | 2.52E-06 | 9.91E-05 | Chr5 | 1.07E+08 | 1.07E+08 | 273 |
| MsG0580029769.01 | -3.00337 | 2.54E-06 | 0.0001 | Chr5 | 1.02E+08 | 1.02E+08 | 2561 |
| MsG0080049073.01 | -2.45407 | 2.56E-06 | 0.000101 | contig649end | 4981 | 5943 | 963 |
| novel.2798 | -8.10956 | 2.56E-06 | 0.000101 | Chr3 | 22784213 | 22791104 | 2914 |
| MsG0380014295.01 | -7.01782 | 2.63E-06 | 0.000103 | Chr3 | 52543518 | 52544226 | 615 |
| MsG0280007412.01 | -7.30644 | 2.64E-06 | 0.000103 | Chr2 | 15050989 | 15052177 | 669 |
| MsG0880045744.01 | 6.077815 | 2.66E-06 | 0.000104 | Chr8 | 63666568 | 63666882 | 315 |
| novel.8087 | 1.791391 | 2.72E-06 | 0.000106 | Chr8 | 57505232 | 57505933 | 702 |
| novel.6247 | 6.511849 | 2.72E-06 | 0.000106 | Chr6 | 16156394 | 16157962 | 1569 |
| MsG0680032066.01 | 3.631679 | 2.75E-06 | 0.000107 | Chr6 | 33017209 | 33018459 | 1029 |
| MsG0580027456.01 | 1.790107 | 2.75E-06 | 0.000107 | Chr5 | 61318361 | 61335082 | 1833 |
| novel.8791 | 7.220988 | 2.76E-06 | 0.000107 | contig106end | 24949 | 26944 | 1996 |
| MsG0480020574.01 | -11.4377 | 2.79E-06 | 0.000108 | Chr4 | 43654757 | 43657902 | 846 |
| novel.8780 | -3.47511 | 2.81E-06 | 0.000109 | Chr8 | 90432690 | 90435521 | 1620 |
| novel.1200 | -3.81687 | 2.82E-06 | 0.000109 | Chr2 | 5594136 | 5595815 | 869 |
| MsG0180004434.01 | 1.838809 | 2.83E-06 | 0.000109 | Chr1 | 77895083 | 77895325 | 243 |
| MsG0680035358.01 | 2.037058 | 2.88E-06 | 0.000111 | Chr6 | 1.03E+08 | 1.03E+08 | 2158 |
| novel.7897 | -3.32332 | 2.89E-06 | 0.000111 | Chr8 | 19408025 | 19410771 | 1346 |
| novel.5285 | -7.0614 | 2.93E-06 | 0.000113 | Chr5 | 75099585 | 75101199 | 1362 |
| MsG0380017344.01 | -1.18439 | 2.94E-06 | 0.000113 | Chr3 | 95202636 | 95203884 | 1088 |
| novel.4019 | 3.243207 | 2.95E-06 | 0.000113 | Chr4 | 57213539 | 57219089 | 1539 |
| novel.1032 | 6.043463 | 2.96E-06 | 0.000114 | Chr1 | 86759855 | 86763870 | 1857 |
| MsG0080047938.01 | -3.57305 | 2.98E-06 | 0.000114 | contig152end | 59103 | 61816 | 2049 |
| MsG0480021506.01 | -2.36376 | 2.99E-06 | 0.000114 | Chr4 | 58770571 | 58774029 | 2450 |
| novel.2700 | -7.52193 | 3.00E-06 | 0.000114 | Chr3 | 5113572 | 5125692 | 876 |
| MsG0680034723.01 | -2.75145 | 3.00E-06 | 0.000114 | Chr6 | 93458415 | 93462233 | 3057 |
| MsG0180002209.01 | -1.31044 | 3.01E-06 | 0.000115 | Chr1 | 34921676 | 34931234 | 3162 |
| novel.6901 | -7.03555 | 3.03E-06 | 0.000115 | Chr7 | 23989347 | 23990986 | 632 |
| novel.2215 | -5.72533 | 3.05E-06 | 0.000116 | Chr3 | 15383364 | 15384004 | 479 |
| MsG0180000201.01 | 3.089914 | 3.08E-06 | 0.000117 | Chr1 | 2760862 | 2761116 | 255 |
| MsG0680030667.01 | 1.999072 | 3.11E-06 | 0.000118 | Chr6 | 6919678 | 6927661 | 1602 |
| MsG0780038988.01 | 7.131029 | 3.12E-06 | 0.000118 | Chr7 | 56483727 | 56484026 | 300 |
| novel.2265 | -6.35288 | 3.14E-06 | 0.000119 | Chr3 | 22784428 | 22789318 | 2370 |
| MsG0580030055.01 | 2.095125 | 3.16E-06 | 0.000119 | Chr5 | 1.06E+08 | 1.06E+08 | 2440 |
| MsG0880044566.01 | -2.15138 | 3.19E-06 | 0.00012 | Chr8 | 44747085 | 44751965 | 1356 |
| novel.8609 | -8.3605 | 3.21E-06 | 0.000121 | Chr8 | 62375059 | 62378392 | 2320 |
| MsG0180004561.01 | 1.875381 | 3.21E-06 | 0.000121 | Chr1 | 79599988 | 79600614 | 546 |
| novel.4161 | 3.794295 | 3.30E-06 | 0.000124 | Chr4 | 79023682 | 79029808 | 1203 |
| MsG0280010112.01 | -2.35581 | 3.32E-06 | 0.000125 | Chr2 | 64649517 | 64652790 | 3165 |
| novel.4753 | -3.67148 | 3.34E-06 | 0.000125 | Chr5 | 86545819 | 86548304 | 2486 |
| novel.5789 | 2.263546 | 3.45E-06 | 0.000129 | Chr6 | 52749603 | 52754946 | 4221 |
| novel.8230 | 6.936729 | 3.47E-06 | 0.00013 | Chr8 | 82677966 | 82678521 | 556 |
| novel.6754 | 3.354792 | 3.51E-06 | 0.000131 | Chr6 | 1.08E+08 | 1.08E+08 | 926 |
| novel.2306 | -2.74294 | 3.51E-06 | 0.000131 | Chr3 | 33448143 | 33451467 | 2485 |
| MsG0080048256.01 | 1.954773 | 3.51E-06 | 0.000131 | contig255end | 521 | 3048 | 619 |
| novel.6941 | 4.26557 | 3.52E-06 | 0.000131 | Chr7 | 30589045 | 30590235 | 976 |
| MsG0480019336.01 | 1.635599 | 3.60E-06 | 0.000134 | Chr4 | 18623780 | 18628447 | 1551 |
| novel.5925 | -1.89494 | 3.65E-06 | 0.000136 | Chr6 | 83775770 | 83780992 | 2008 |
| novel.5016 | -2.13976 | 3.67E-06 | 0.000136 | Chr5 | 19803587 | 19807244 | 2113 |
| MsG0180000930.01 | -5.11948 | 3.80E-06 | 0.000141 | Chr1 | 13361767 | 13366310 | 2697 |
| MsG0080048137.01 | 1.221878 | 3.81E-06 | 0.000141 | contig218end | 5952 | 7092 | 276 |
| MsG0380017008.01 | 1.320504 | 3.81E-06 | 0.000141 | Chr3 | 90597355 | 90602593 | 1637 |
| MsG0280010985.01 | -2.02854 | 3.83E-06 | 0.000141 | Chr2 | 77981334 | 77982734 | 1401 |
| novel.4292 | -3.10196 | 3.84E-06 | 0.000142 | Chr5 | 1999366 | 2000706 | 1341 |
| MsG0680032278.01 | -3.47831 | 3.90E-06 | 0.000144 | Chr6 | 37742377 | 37745607 | 3231 |
| MsG0880042073.01 | -2.79461 | 3.98E-06 | 0.000146 | Chr8 | 3390461 | 3398192 | 5686 |
| novel.3510 | -1.84673 | 4.00E-06 | 0.000147 | Chr4 | 57497345 | 57505481 | 983 |
| MsG0080048355.01 | 6.877859 | 4.02E-06 | 0.000148 | contig280end | 16641 | 21783 | 2349 |
| MsG0580028707.01 | -3.23852 | 4.03E-06 | 0.000148 | Chr5 | 85127293 | 85133208 | 3054 |
| MsG0180003243.01 | 2.960168 | 4.04E-06 | 0.000148 | Chr1 | 58967754 | 58971132 | 798 |
| MsG0280007272.01 | 2.308405 | 4.05E-06 | 0.000148 | Chr2 | 12887917 | 12889713 | 978 |
| novel.7287 | 3.522377 | 4.05E-06 | 0.000148 | Chr7 | 94296972 | 94300513 | 1142 |
| MsG0380011742.01 | -4.61728 | 4.06E-06 | 0.000148 | Chr3 | 4374577 | 4377905 | 1506 |
| novel.2148 | 3.95124 | 4.08E-06 | 0.000149 | Chr3 | 5295108 | 5302064 | 1811 |
| novel.7360 | 7.164432 | 4.09E-06 | 0.000149 | Chr7 | 15323487 | 15324006 | 520 |
| novel.734 | 4.374882 | 4.10E-06 | 0.000149 | Chr1 | 19565606 | 19570284 | 2260 |
| MsG0480021016.01 | -4.03468 | 4.14E-06 | 0.00015 | Chr4 | 52125314 | 52125766 | 453 |
| MsG0380013593.01 | 7.722436 | 4.14E-06 | 0.00015 | Chr3 | 41349209 | 41350048 | 840 |
| MsG0480019417.01 | 1.079403 | 4.16E-06 | 0.00015 | Chr4 | 19935485 | 19945677 | 844 |
| novel.3280 | 7.083566 | 4.22E-06 | 0.000153 | Chr4 | 10499132 | 10500494 | 1203 |
| MsG0680034380.01 | 2.332571 | 4.24E-06 | 0.000153 | Chr6 | 86619624 | 86620640 | 1017 |
| MsG0880044282.01 | 5.126491 | 4.24E-06 | 0.000153 | Chr8 | 39386957 | 39388865 | 730 |
| MsG0380016546.01 | -3.45229 | 4.37E-06 | 0.000158 | Chr3 | 84993188 | 84993878 | 363 |
| MsG0580025785.01 | -2.44533 | 4.41E-06 | 0.000159 | Chr5 | 24076558 | 24080470 | 2196 |
| MsG0280007556.01 | 3.80788 | 4.43E-06 | 0.000159 | Chr2 | 17106944 | 17107981 | 1038 |
| novel.2526 | -2.54111 | 4.44E-06 | 0.000159 | Chr3 | 83471058 | 83473814 | 907 |
| novel.2837 | 5.193973 | 4.44E-06 | 0.000159 | Chr3 | 32071317 | 32072374 | 1058 |
| MsG0880042748.01 | -2.81862 | 4.49E-06 | 0.000161 | Chr8 | 13485742 | 13494085 | 1018 |
| MsG0480023981.01 | 2.197423 | 4.49E-06 | 0.000161 | Chr4 | 91961033 | 91961353 | 321 |
| MsG0780041803.01 | 6.852645 | 4.50E-06 | 0.000161 | Chr7 | 95335382 | 95339815 | 1986 |
| MsG0480019217.01 | -4.27135 | 4.54E-06 | 0.000162 | Chr4 | 16317192 | 16320811 | 1963 |
| MsG0580027776.01 | -2.16791 | 4.59E-06 | 0.000164 | Chr5 | 68336233 | 68336943 | 711 |
| novel.4453 | -5.66206 | 4.62E-06 | 0.000165 | Chr5 | 25972405 | 25973215 | 655 |
| MsG0780039087.01 | 3.359991 | 4.63E-06 | 0.000165 | Chr7 | 57755706 | 57762900 | 5654 |
| novel.8465 | -3.63609 | 4.67E-06 | 0.000166 | Chr8 | 31765159 | 31769390 | 3367 |
| MsG0080048756.01 | -6.96094 | 4.78E-06 | 0.00017 | contig424end | 10953 | 12753 | 957 |
| novel.8050 | -2.25102 | 4.79E-06 | 0.00017 | Chr8 | 50941665 | 50945162 | 1730 |
| MsG0580024531.01 | 1.81085 | 4.88E-06 | 0.000173 | Chr5 | 6089444 | 6092955 | 1563 |
| novel.4636 | -2.96454 | 4.97E-06 | 0.000176 | Chr5 | 65313353 | 65318389 | 1673 |
| novel.3842 | -4.62161 | 5.04E-06 | 0.000178 | Chr4 | 17995096 | 17997628 | 1006 |
| MsG0780039506.01 | 6.463781 | 5.14E-06 | 0.000181 | Chr7 | 65016864 | 65017422 | 462 |
| MsG0280007250.01 | -2.58808 | 5.21E-06 | 0.000184 | Chr2 | 12535559 | 12554840 | 1152 |
| novel.7964 | 2.087623 | 5.24E-06 | 0.000185 | Chr8 | 31551732 | 31564372 | 2520 |
| MsG0480021474.01 | -2.39698 | 5.29E-06 | 0.000186 | Chr4 | 58288431 | 58300167 | 1207 |
| MsG0580029482.01 | 7.058687 | 5.31E-06 | 0.000186 | Chr5 | 97585636 | 97586795 | 300 |
| MsG0580028268.01 | -3.41194 | 5.41E-06 | 0.00019 | Chr5 | 77546017 | 77547513 | 1497 |
| novel.3407 | -2.53716 | 5.41E-06 | 0.00019 | Chr4 | 35285766 | 35288538 | 1291 |
| MsG0280008597.01 | -5.48506 | 5.44E-06 | 0.000191 | Chr2 | 34604308 | 34606153 | 1022 |
| MsG0380014383.01 | -1.84588 | 5.45E-06 | 0.000191 | Chr3 | 53914370 | 53922260 | 1842 |
| MsG0180005708.01 | 1.355874 | 5.47E-06 | 0.000191 | Chr1 | 95235789 | 95237182 | 1353 |
| MsG0680031194.01 | -5.59249 | 5.48E-06 | 0.000191 | Chr6 | 15799396 | 15801376 | 312 |
| novel.2032 | 1.450385 | 5.51E-06 | 0.000192 | Chr2 | 74387639 | 74389255 | 1113 |
| MsG0180001100.01 | -1.957 | 5.54E-06 | 0.000193 | Chr1 | 15987120 | 15989464 | 729 |
| novel.68 | 6.943089 | 5.58E-06 | 0.000194 | Chr1 | 8694464 | 8694870 | 407 |
| novel.4162 | 4.153524 | 5.61E-06 | 0.000195 | Chr4 | 79032010 | 79035824 | 808 |
| novel.4672 | -6.78877 | 5.62E-06 | 0.000195 | Chr5 | 72976073 | 72978080 | 1740 |
| novel.3767 | -6.86379 | 5.73E-06 | 0.000198 | Chr4 | 2848162 | 2850153 | 747 |
| MsG0480018784.01 | -2.53063 | 5.73E-06 | 0.000198 | Chr4 | 9714606 | 9719193 | 1911 |
| MsG0180002026.01 | 7.753685 | 5.82E-06 | 0.000201 | Chr1 | 31332696 | 31346663 | 5195 |
| MsG0180004267.01 | -1.91937 | 5.86E-06 | 0.000202 | Chr1 | 75766898 | 75768770 | 711 |
| MsG0280009859.01 | -2.11407 | 5.86E-06 | 0.000202 | Chr2 | 59482251 | 59485931 | 1874 |
| novel.5765 | -7.39253 | 5.88E-06 | 0.000203 | Chr6 | 48979499 | 48980961 | 1463 |
| MsG0680034290.01 | -3.25786 | 6.05E-06 | 0.000208 | Chr6 | 85113715 | 85118410 | 885 |
| MsG0580029376.01 | -1.11872 | 6.08E-06 | 0.000209 | Chr5 | 96093592 | 96110466 | 5234 |
| novel.2272 | -2.43055 | 6.14E-06 | 0.000211 | Chr3 | 24902960 | 24906913 | 1458 |
| MsG0180001188.01 | 4.804163 | 6.16E-06 | 0.000211 | Chr1 | 16977175 | 16979094 | 1920 |
| MsG0480023319.01 | -1.62848 | 6.16E-06 | 0.000211 | Chr4 | 83696629 | 83700527 | 2029 |
| MsG0280006428.01 | -3.23786 | 6.19E-06 | 0.000212 | Chr2 | 1846711 | 1848072 | 1362 |
| MsG0180005686.01 | -1.54275 | 6.20E-06 | 0.000212 | Chr1 | 94916486 | 94920071 | 2331 |
| MsG0580028406.01 | -2.98285 | 6.24E-06 | 0.000213 | Chr5 | 80133390 | 80145099 | 4269 |
| novel.8895 | 5.141783 | 6.30E-06 | 0.000215 | contig264end | 15237 | 16797 | 687 |
| MsG0880042933.01 | -1.99111 | 6.34E-06 | 0.000216 | Chr8 | 16203579 | 16217888 | 2293 |
| MsG0780037026.01 | 2.115414 | 6.43E-06 | 0.000219 | Chr7 | 18267355 | 18271457 | 1455 |
| MsG0480018123.01 | -3.62946 | 6.48E-06 | 0.00022 | Chr4 | 477121 | 481306 | 2025 |
| novel.3998 | -7.1434 | 6.50E-06 | 0.000221 | Chr4 | 54052179 | 54055501 | 1062 |
| MsG0180001581.01 | 7.19106 | 6.53E-06 | 0.000222 | Chr1 | 23653576 | 23656845 | 2799 |
| MsG0880045385.01 | -1.60301 | 6.64E-06 | 0.000225 | Chr8 | 58616953 | 58629046 | 2942 |
| MsG0180004424.01 | -5.68669 | 6.68E-06 | 0.000226 | Chr1 | 77738491 | 77739664 | 912 |
| novel.7991 | 6.963151 | 6.78E-06 | 0.000229 | Chr8 | 38291176 | 38291588 | 285 |
| MsG0180005372.01 | -4.91459 | 6.80E-06 | 0.00023 | Chr1 | 90949871 | 90956038 | 2142 |
| MsG0880043533.01 | -2.3156 | 6.83E-06 | 0.00023 | Chr8 | 26240146 | 26243573 | 378 |
| MsG0080048152.01 | -2.66217 | 6.89E-06 | 0.000232 | contig223end | 17504 | 17893 | 390 |
| MsG0580030247.01 | -5.18204 | 6.91E-06 | 0.000233 | Chr5 | 1.09E+08 | 1.09E+08 | 3280 |
| novel.195 | 6.88861 | 6.93E-06 | 0.000233 | Chr1 | 27693602 | 27694404 | 704 |
| MsG0480023997.01 | -6.67317 | 7.01E-06 | 0.000235 | Chr4 | 92198449 | 92198811 | 363 |
| novel.7076 | -4.00484 | 7.07E-06 | 0.000237 | Chr7 | 64561924 | 64565367 | 2412 |
| MsG0480021906.01 | 1.688387 | 7.10E-06 | 0.000238 | Chr4 | 65864221 | 65864925 | 705 |
| MsG0280007471.01 | -3.18029 | 7.17E-06 | 0.00024 | Chr2 | 15910230 | 15911429 | 1200 |
| MsG0480019106.01 | -2.73489 | 7.24E-06 | 0.000242 | Chr4 | 14664275 | 14670051 | 4257 |
| MsG0680032788.01 | -5.48296 | 7.27E-06 | 0.000243 | Chr6 | 49693117 | 49694691 | 1575 |
| MsG0780036443.01 | 1.816588 | 7.28E-06 | 0.000243 | Chr7 | 8203565 | 8205133 | 613 |
| MsG0880045119.01 | 1.180792 | 7.31E-06 | 0.000244 | Chr8 | 54478580 | 54480991 | 874 |
| MsG0380011547.01 | -6.33633 | 7.34E-06 | 0.000244 | Chr3 | 1251228 | 1251644 | 417 |
| MsG0580030133.01 | -1.33083 | 7.40E-06 | 0.000246 | Chr5 | 1.07E+08 | 1.07E+08 | 1836 |
| novel.5366 | 7.153942 | 7.45E-06 | 0.000247 | Chr5 | 89493998 | 89497386 | 1948 |
| novel.4583 | 1.577524 | 7.48E-06 | 0.000248 | Chr5 | 54686780 | 54689581 | 837 |
| MsG0880043511.01 | -2.51396 | 7.52E-06 | 0.000249 | Chr8 | 25981318 | 25988059 | 1764 |
| MsG0380012987.01 | -2.96199 | 7.58E-06 | 0.000251 | Chr3 | 28347319 | 28348228 | 751 |
| novel.5504 | -5.15545 | 7.61E-06 | 0.000252 | Chr6 | 313358 | 314829 | 1044 |
| MsG0580025279.01 | 2.89154 | 7.68E-06 | 0.000254 | Chr5 | 16387777 | 16395933 | 2005 |
| MsG0580025523.01 | 2.310827 | 7.69E-06 | 0.000254 | Chr5 | 19972941 | 19974330 | 747 |
| novel.2356 | 3.398803 | 7.76E-06 | 0.000256 | Chr3 | 46923092 | 46931759 | 949 |
| novel.6971 | -6.79009 | 7.77E-06 | 0.000256 | Chr7 | 39094769 | 39096641 | 893 |
| MsG0480018609.01 | -1.57358 | 7.79E-06 | 0.000256 | Chr4 | 7342372 | 7355685 | 1859 |
| MsG0580024389.01 | 6.771835 | 7.81E-06 | 0.000257 | Chr5 | 4173577 | 4176904 | 1584 |
| MsG0480018426.01 | 1.636019 | 7.96E-06 | 0.000261 | Chr4 | 4747423 | 4751185 | 3763 |
| novel.8783 | 3.160848 | 7.97E-06 | 0.000261 | Chr8 | 90580596 | 90584658 | 1182 |
| MsG0780036550.01 | -2.92156 | 7.98E-06 | 0.000262 | Chr7 | 9652278 | 9652795 | 444 |
| MsG0380012614.01 | -7.12662 | 8.05E-06 | 0.000263 | Chr3 | 21086517 | 21093445 | 3600 |
| MsG0880047084.01 | -2.96123 | 8.08E-06 | 0.000264 | Chr8 | 81701430 | 81702159 | 477 |
| MsG0780039108.01 | -3.09974 | 8.09E-06 | 0.000264 | Chr7 | 58061054 | 58079119 | 7260 |
| MsG0880046291.01 | 3.309705 | 8.17E-06 | 0.000266 | Chr8 | 71605628 | 71607073 | 477 |
| MsG0580027724.01 | -2.17753 | 8.17E-06 | 0.000266 | Chr5 | 67458081 | 67461086 | 1230 |
| MsG0080047861.01 | -2.33511 | 8.19E-06 | 0.000267 | contig127end | 52705 | 65593 | 2142 |
| MsG0580028529.01 | -3.36351 | 8.21E-06 | 0.000267 | Chr5 | 81888248 | 81889544 | 1212 |
| MsG0280008387.01 | -2.36379 | 8.24E-06 | 0.000268 | Chr2 | 30529740 | 30530429 | 690 |
| MsG0580027661.01 | -3.40196 | 8.29E-06 | 0.000269 | Chr5 | 65394018 | 65396900 | 2883 |
| MsG0480022640.01 | 2.156774 | 8.32E-06 | 0.00027 | Chr4 | 74954924 | 74955193 | 270 |
| novel.814 | 6.76824 | 8.36E-06 | 0.000271 | Chr1 | 34643045 | 34647117 | 1224 |
| MsG0480023546.01 | -1.62156 | 8.46E-06 | 0.000273 | Chr4 | 86725861 | 86729091 | 906 |
| novel.5258 | 2.493005 | 8.50E-06 | 0.000274 | Chr5 | 70749873 | 70754350 | 1985 |
| novel.7328 | 1.902991 | 8.50E-06 | 0.000274 | Chr7 | 8984167 | 8985632 | 1029 |
| novel.3969 | -4.00059 | 8.53E-06 | 0.000275 | Chr4 | 47860070 | 47860861 | 561 |
| MsG0580025154.01 | -3.10358 | 8.62E-06 | 0.000277 | Chr5 | 14527220 | 14538593 | 3039 |
| novel.1870 | -1.50017 | 8.64E-06 | 0.000278 | Chr2 | 37295192 | 37305148 | 4465 |
| novel.2447 | -2.5238 | 8.68E-06 | 0.000279 | Chr3 | 69711800 | 69713591 | 1537 |
| MsG0080047804.01 | -5.16966 | 8.85E-06 | 0.000284 | contig108end | 27427 | 27735 | 309 |
| novel.8137 | -1.69775 | 8.88E-06 | 0.000285 | Chr8 | 64712151 | 64727332 | 2791 |
| MsG0580028247.01 | -3.52818 | 8.91E-06 | 0.000285 | Chr5 | 77230040 | 77230462 | 423 |
| novel.1660 | 2.053017 | 8.91E-06 | 0.000285 | Chr2 | 2185567 | 2187651 | 2085 |
| MsG0380017457.01 | -2.68803 | 8.91E-06 | 0.000285 | Chr3 | 96482492 | 96484899 | 1023 |
| MsG0380016086.01 | -1.78659 | 9.01E-06 | 0.000287 | Chr3 | 78977026 | 78978734 | 1353 |
| MsG0480020696.01 | 5.501037 | 9.03E-06 | 0.000288 | Chr4 | 46461535 | 46464263 | 1497 |
| MsG0480022160.01 | -3.34801 | 9.04E-06 | 0.000288 | Chr4 | 69105162 | 69107887 | 753 |
| MsG0380016317.01 | 1.543797 | 9.07E-06 | 0.000288 | Chr3 | 81918119 | 81919183 | 621 |
| novel.496 | -6.70209 | 9.09E-06 | 0.000289 | Chr1 | 87697568 | 87697882 | 315 |
| MsG0580028990.01 | 6.485708 | 9.16E-06 | 0.000291 | Chr5 | 90031349 | 90035524 | 1103 |
| MsG0180004400.01 | -1.84884 | 9.17E-06 | 0.000291 | Chr1 | 77490697 | 77491248 | 552 |
| MsG0280008172.01 | -1.40954 | 9.21E-06 | 0.000292 | Chr2 | 26731174 | 26735482 | 1446 |
| MsG0280010021.01 | -1.60288 | 9.32E-06 | 0.000295 | Chr2 | 62973240 | 62984916 | 2348 |
| novel.1525 | -5.12816 | 9.41E-06 | 0.000297 | Chr2 | 70223473 | 70227470 | 831 |
| MsG0680032272.01 | 2.526563 | 9.52E-06 | 0.000301 | Chr6 | 37644058 | 37647596 | 3225 |
| MsG0180000436.01 | 2.44214 | 9.58E-06 | 0.000302 | Chr1 | 5996868 | 5999427 | 1665 |
| novel.6170 | -6.86913 | 9.58E-06 | 0.000302 | Chr6 | 5685751 | 5689625 | 1151 |
| novel.4678 | -2.17474 | 9.71E-06 | 0.000305 | Chr5 | 73842044 | 73845649 | 1876 |
| MsG0480018928.01 | 3.76798 | 9.80E-06 | 0.000308 | Chr4 | 11888175 | 11891563 | 945 |
| MsG0780036703.01 | -1.52115 | 9.82E-06 | 0.000308 | Chr7 | 12166873 | 12168189 | 1317 |
| MsG0380017480.01 | -4.86819 | 9.89E-06 | 0.00031 | Chr3 | 96733201 | 96734001 | 537 |
| novel.8698 | 6.768549 | 9.89E-06 | 0.00031 | Chr8 | 76813105 | 76814600 | 1403 |
| MsG0080047960.01 | -3.14491 | 9.95E-06 | 0.000311 | contig152end | 363422 | 370029 | 2413 |
| MsG0380016628.01 | 2.658319 | 9.97E-06 | 0.000312 | Chr3 | 85870591 | 85870935 | 345 |
| novel.6382 | -6.93219 | 9.99E-06 | 0.000312 | Chr6 | 36976956 | 36977671 | 716 |
| MsG0380012860.01 | 1.596324 | 1.01E-05 | 0.000314 | Chr3 | 25736154 | 25739728 | 1479 |
| MsG0680031452.01 | 2.306154 | 1.01E-05 | 0.000315 | Chr6 | 20672096 | 20684199 | 1885 |
| MsG0880045222.01 | -2.57415 | 1.01E-05 | 0.000316 | Chr8 | 56035048 | 56039125 | 1083 |
| MsG0680030985.01 | -3.24414 | 1.02E-05 | 0.000316 | Chr6 | 12532199 | 12536559 | 2007 |
| MsG0180001072.01 | 1.47257 | 1.02E-05 | 0.000316 | Chr1 | 15613325 | 15628687 | 2011 |
| MsG0680034190.01 | 2.308208 | 1.02E-05 | 0.000316 | Chr6 | 83015018 | 83020738 | 713 |
| novel.461 | 1.523011 | 1.02E-05 | 0.000316 | Chr1 | 84808723 | 84812188 | 1331 |
| novel.5163 | -2.4124 | 1.02E-05 | 0.000316 | Chr5 | 51583074 | 51585743 | 1248 |
| novel.6943 | 4.315121 | 1.03E-05 | 0.000317 | Chr7 | 31011314 | 31012025 | 305 |
| novel.2173 | 1.260731 | 1.03E-05 | 0.000317 | Chr3 | 8238694 | 8241259 | 2422 |
| MsG0580029374.01 | 1.201969 | 1.04E-05 | 0.00032 | Chr5 | 96066959 | 96072195 | 811 |
| novel.6906 | -2.62118 | 1.04E-05 | 0.000321 | Chr7 | 25121568 | 25124518 | 1396 |
| MsG0680034079.01 | 7.027379 | 1.05E-05 | 0.000322 | Chr6 | 80141312 | 80143773 | 1563 |
| novel.1936 | 5.581761 | 1.05E-05 | 0.000324 | Chr2 | 56574405 | 56577919 | 1479 |
| MsG0880046413.01 | 1.83164 | 1.05E-05 | 0.000324 | Chr8 | 73151746 | 73162490 | 2379 |
| MsG0180006224.01 | 1.579728 | 1.06E-05 | 0.000327 | Chr1 | 1.02E+08 | 1.02E+08 | 2045 |
| MsG0380011535.01 | -3.63181 | 1.07E-05 | 0.000328 | Chr3 | 1040491 | 1048461 | 4718 |
| novel.8697 | -3.21037 | 1.07E-05 | 0.000329 | Chr8 | 76592537 | 76593624 | 448 |
| novel.4697 | -6.31127 | 1.08E-05 | 0.00033 | Chr5 | 77307432 | 77308679 | 1153 |
| novel.5331 | 4.866656 | 1.08E-05 | 0.000331 | Chr5 | 83606316 | 83606938 | 602 |
| novel.6616 | -3.70666 | 1.08E-05 | 0.000331 | Chr6 | 87728518 | 87733345 | 1670 |
| MsG0580025586.01 | -3.50978 | 1.10E-05 | 0.000335 | Chr5 | 20875197 | 20892613 | 3738 |
| novel.2129 | 6.712139 | 1.10E-05 | 0.000337 | Chr3 | 1468512 | 1468917 | 406 |
| novel.7002 | -6.38346 | 1.11E-05 | 0.000337 | Chr7 | 46799471 | 46802334 | 694 |
| MsG0180001976.01 | 1.19742 | 1.11E-05 | 0.000337 | Chr1 | 30131703 | 30137560 | 526 |
| MsG0780036029.01 | -2.9767 | 1.11E-05 | 0.000338 | Chr7 | 1524404 | 1532396 | 2289 |
| novel.1065 | -3.39657 | 1.13E-05 | 0.000343 | Chr1 | 90259957 | 90265273 | 3816 |
| MsG0580025805.01 | -6.5353 | 1.15E-05 | 0.000348 | Chr5 | 24481716 | 24482195 | 480 |
| MsG0180000823.01 | 4.257728 | 1.15E-05 | 0.000349 | Chr1 | 11675268 | 11676659 | 1392 |
| novel.5142 | -6.05534 | 1.15E-05 | 0.000349 | Chr5 | 47135622 | 47141885 | 2649 |
| novel.7676 | -6.24959 | 1.17E-05 | 0.000353 | Chr7 | 80421447 | 80424480 | 795 |
| MsG0180001944.01 | 2.885367 | 1.18E-05 | 0.000356 | Chr1 | 29699073 | 29700278 | 1206 |
| novel.2945 | -5.67452 | 1.20E-05 | 0.000362 | Chr3 | 60765796 | 60767477 | 1682 |
| MsG0380014552.01 | -2.64238 | 1.20E-05 | 0.000363 | Chr3 | 56426859 | 56438397 | 2502 |
| novel.5805 | -6.39644 | 1.23E-05 | 0.000372 | Chr6 | 58804374 | 58817942 | 1310 |
| novel.5516 | -7.17809 | 1.23E-05 | 0.000372 | Chr6 | 1918029 | 1919134 | 1106 |
| novel.7295 | 2.830784 | 1.26E-05 | 0.000379 | Chr7 | 95351475 | 95353578 | 1164 |
| novel.2196 | 4.06924 | 1.26E-05 | 0.00038 | Chr3 | 12671410 | 12672887 | 649 |
| MsG0180005795.01 | -2.04898 | 1.27E-05 | 0.000381 | Chr1 | 96217710 | 96220646 | 1579 |
| MsG0180000798.01 | 1.381072 | 1.27E-05 | 0.000382 | Chr1 | 11306768 | 11310808 | 904 |
| MsG0780040062.01 | 1.125453 | 1.29E-05 | 0.000386 | Chr7 | 72573108 | 72582964 | 1986 |
| MsG0880045924.01 | -1.93798 | 1.30E-05 | 0.00039 | Chr8 | 66536746 | 66537084 | 339 |
| MsG0780036847.01 | -7.92706 | 1.32E-05 | 0.000395 | Chr7 | 14975822 | 14977321 | 1059 |
| MsG0380014528.01 | -7.13548 | 1.32E-05 | 0.000396 | Chr3 | 56083220 | 56090419 | 3831 |
| novel.7983 | 6.850316 | 1.32E-05 | 0.000396 | Chr8 | 36804148 | 36804649 | 402 |
| MsG0580028850.01 | 2.349166 | 1.33E-05 | 0.000397 | Chr5 | 87553342 | 87555956 | 666 |
| novel.3317 | 7.193787 | 1.34E-05 | 0.000398 | Chr4 | 16450415 | 16454107 | 388 |
| MsG0480018857.01 | -5.67422 | 1.34E-05 | 0.000399 | Chr4 | 10669049 | 10674003 | 1828 |
| novel.1699 | 3.322008 | 1.35E-05 | 0.000401 | Chr2 | 7187252 | 7190237 | 749 |
| MsG0880045747.01 | -2.08874 | 1.35E-05 | 0.000402 | Chr8 | 63691877 | 63696404 | 2449 |
| novel.7914 | -3.01829 | 1.36E-05 | 0.000404 | Chr8 | 21561359 | 21561875 | 435 |
| MsG0480022828.01 | -2.37483 | 1.37E-05 | 0.000405 | Chr4 | 77218797 | 77220964 | 1752 |
| MsG0680035764.01 | -6.63515 | 1.37E-05 | 0.000405 | Chr6 | 1.12E+08 | 1.12E+08 | 376 |
| MsG0380013117.01 | -1.9788 | 1.37E-05 | 0.000406 | Chr3 | 30731590 | 30732303 | 324 |
| novel.5098 | -3.48891 | 1.38E-05 | 0.000409 | Chr5 | 37681601 | 37682715 | 957 |
| novel.8659 | 6.656414 | 1.39E-05 | 0.000412 | Chr8 | 70524623 | 70525455 | 246 |
| novel.6518 | -1.81093 | 1.40E-05 | 0.000412 | Chr6 | 66079450 | 66085228 | 2997 |
| MsG0480022862.01 | -6.47281 | 1.40E-05 | 0.000413 | Chr4 | 77676456 | 77677538 | 1083 |
| MsG0880042436.01 | 1.725806 | 1.40E-05 | 0.000414 | Chr8 | 8678272 | 8678820 | 549 |
| MsG0780036116.01 | -2.51872 | 1.41E-05 | 0.000415 | Chr7 | 2869907 | 2872561 | 2460 |
| MsG0480019785.01 | 1.369095 | 1.41E-05 | 0.000416 | Chr4 | 27107619 | 27115693 | 3304 |
| MsG0680033271.01 | -6.64239 | 1.41E-05 | 0.000416 | Chr6 | 60779407 | 60780894 | 1488 |
| MsG0180002450.01 | 1.163327 | 1.42E-05 | 0.000416 | Chr1 | 38549816 | 38550518 | 397 |
| MsG0780040724.01 | 6.701044 | 1.42E-05 | 0.000418 | Chr7 | 81638876 | 81648731 | 4656 |
| MsG0880043829.01 | -1.18385 | 1.43E-05 | 0.000419 | Chr8 | 31302023 | 31305669 | 595 |
| MsG0280006809.01 | 1.919705 | 1.43E-05 | 0.00042 | Chr2 | 6595493 | 6598544 | 1052 |
| novel.3987 | -3.31233 | 1.44E-05 | 0.000422 | Chr4 | 52404468 | 52405934 | 1336 |
| MsG0880046976.01 | 1.3809 | 1.45E-05 | 0.000424 | Chr8 | 80572541 | 80572981 | 441 |
| novel.6302 | 3.333561 | 1.46E-05 | 0.000427 | Chr6 | 22725510 | 22734291 | 1749 |
| novel.6013 | -2.52667 | 1.47E-05 | 0.000428 | Chr6 | 99376247 | 99379174 | 608 |
| novel.4926 | -5.70986 | 1.48E-05 | 0.000431 | Chr5 | 3653602 | 3655225 | 1353 |
| novel.1723 | -2.92124 | 1.51E-05 | 0.000439 | Chr2 | 10736881 | 10749674 | 3870 |
| novel.5535 | -1.6243 | 1.52E-05 | 0.000441 | Chr6 | 4994324 | 4999079 | 3567 |
| MsG0580025119.01 | -2.21014 | 1.52E-05 | 0.000442 | Chr5 | 14051857 | 14054372 | 1656 |
| MsG0880042070.01 | 1.18761 | 1.52E-05 | 0.000442 | Chr8 | 3357771 | 3362716 | 3084 |
| novel.1115 | -1.73533 | 1.54E-05 | 0.000447 | Chr1 | 96866681 | 96868419 | 858 |
| novel.8244 | 1.363642 | 1.58E-05 | 0.000457 | Chr8 | 84825049 | 84826848 | 1800 |
| MsG0580028708.01 | -5.10674 | 1.58E-05 | 0.000458 | Chr5 | 85146937 | 85150658 | 1488 |
| MsG0880046572.01 | -7.37833 | 1.59E-05 | 0.00046 | Chr8 | 75009809 | 75014052 | 1149 |
| MsG0280010301.01 | 6.938286 | 1.59E-05 | 0.00046 | Chr2 | 67607764 | 67615431 | 1185 |
| novel.3838 | -1.05471 | 1.60E-05 | 0.000461 | Chr4 | 17694083 | 17697552 | 2997 |
| novel.351 | -6.63686 | 1.61E-05 | 0.000463 | Chr1 | 63609991 | 63612664 | 731 |
| MsG0780038496.01 | -1.33006 | 1.62E-05 | 0.000467 | Chr7 | 47684216 | 47684746 | 531 |
| novel.8661 | -2.75247 | 1.64E-05 | 0.000471 | Chr8 | 71070197 | 71075405 | 3250 |
| novel.2011 | 4.090863 | 1.65E-05 | 0.000473 | Chr2 | 70782936 | 70783524 | 516 |
| MsG0780041335.01 | -2.64931 | 1.65E-05 | 0.000473 | Chr7 | 89720192 | 89731653 | 4803 |
| novel.7623 | 6.723558 | 1.65E-05 | 0.000473 | Chr7 | 72229845 | 72231588 | 579 |
| MsG0380013595.01 | 7.145904 | 1.65E-05 | 0.000474 | Chr3 | 41381127 | 41381969 | 843 |
| MsG0880045491.01 | -3.08983 | 1.66E-05 | 0.000475 | Chr8 | 59990755 | 59991642 | 828 |
| novel.7039 | 6.692741 | 1.67E-05 | 0.000478 | Chr7 | 56268066 | 56268489 | 380 |
| novel.6379 | 4.575734 | 1.68E-05 | 0.000479 | Chr6 | 36706229 | 36706992 | 764 |
| MsG0780036820.01 | -1.67105 | 1.68E-05 | 0.000481 | Chr7 | 14432495 | 14434527 | 1161 |
| MsG0880045690.01 | 2.204417 | 1.70E-05 | 0.000484 | Chr8 | 62797392 | 62799663 | 1089 |
| novel.3242 | -1.75636 | 1.70E-05 | 0.000485 | Chr4 | 4865314 | 4869745 | 1020 |
| MsG0280011252.01 | -1.75119 | 1.72E-05 | 0.00049 | Chr2 | 81474974 | 81476944 | 1413 |
| novel.3980 | -6.88926 | 1.74E-05 | 0.000494 | Chr4 | 50563009 | 50584150 | 1472 |
| MsG0880042286.01 | 3.641941 | 1.74E-05 | 0.000494 | Chr8 | 6526522 | 6527593 | 717 |
| MsG0480018485.01 | 1.276285 | 1.76E-05 | 0.000499 | Chr4 | 5607322 | 5613189 | 1335 |
| MsG0680035786.01 | 2.533778 | 1.77E-05 | 0.000501 | Chr6 | 1.12E+08 | 1.12E+08 | 532 |
| MsG0780036517.01 | -1.3016 | 1.77E-05 | 0.000503 | Chr7 | 9190092 | 9205933 | 3470 |
| novel.1676 | -2.1113 | 1.78E-05 | 0.000503 | Chr2 | 3798137 | 3801001 | 1587 |
| MsG0480020074.01 | -6.78701 | 1.78E-05 | 0.000503 | Chr4 | 33433420 | 33437563 | 771 |
| novel.4452 | -6.77064 | 1.78E-05 | 0.000504 | Chr5 | 25970661 | 25972236 | 965 |
| MsG0580025207.01 | 1.46957 | 1.81E-05 | 0.000512 | Chr5 | 15316078 | 15326050 | 3303 |
| novel.2923 | -3.39465 | 1.82E-05 | 0.000513 | Chr3 | 54266727 | 54268772 | 1624 |
| novel.2549 | -2.68051 | 1.86E-05 | 0.000523 | Chr3 | 85412592 | 85415706 | 1436 |
| MsG0280009908.01 | -2.03871 | 1.87E-05 | 0.000526 | Chr2 | 60605789 | 60611000 | 1614 |
| novel.1368 | 6.660502 | 1.87E-05 | 0.000526 | Chr2 | 33870709 | 33871754 | 689 |
| novel.6188 | 1.948326 | 1.89E-05 | 0.000532 | Chr6 | 7937252 | 7940655 | 2462 |
| MsG0280009169.01 | -2.12751 | 1.91E-05 | 0.000536 | Chr2 | 46803226 | 46805600 | 711 |
| MsG0580024303.01 | -6.72136 | 1.91E-05 | 0.000537 | Chr5 | 3177300 | 3177923 | 624 |
| MsG0780041447.01 | -1.0786 | 1.93E-05 | 0.000539 | Chr7 | 90995818 | 90997279 | 773 |
| novel.3354 | -6.06338 | 1.93E-05 | 0.000539 | Chr4 | 24639818 | 24642843 | 2509 |
| MsG0280008336.01 | 6.841644 | 1.94E-05 | 0.000541 | Chr2 | 29538540 | 29544407 | 2772 |
| novel.7477 | -4.51241 | 1.94E-05 | 0.000541 | Chr7 | 41353179 | 41357639 | 1095 |
| novel.1398 | -4.50134 | 1.94E-05 | 0.000541 | Chr2 | 38475411 | 38477000 | 1307 |
| MsG0080048231.01 | -3.45989 | 1.95E-05 | 0.000543 | contig249end | 3781 | 4984 | 1110 |
| MsG0680032286.01 | -7.2058 | 1.95E-05 | 0.000544 | Chr6 | 37805699 | 37812660 | 3327 |
| novel.8398 | 7.004906 | 1.97E-05 | 0.000549 | Chr8 | 19022010 | 19022854 | 641 |
| novel.2678 | -3.30655 | 2.00E-05 | 0.000555 | Chr3 | 976230 | 979248 | 3019 |
| novel.4802 | -4.047 | 2.00E-05 | 0.000555 | Chr5 | 95402147 | 95404631 | 713 |
| MsG0580027088.01 | -2.47109 | 2.00E-05 | 0.000555 | Chr5 | 52928251 | 52933090 | 1245 |
| MsG0680035618.01 | -1.81144 | 2.01E-05 | 0.000558 | Chr6 | 1.09E+08 | 1.09E+08 | 1281 |
| MsG0680034989.01 | -1.97257 | 2.02E-05 | 0.000559 | Chr6 | 97395999 | 97401367 | 3408 |
| MsG0880042068.01 | 2.032896 | 2.02E-05 | 0.00056 | Chr8 | 3326102 | 3334526 | 1797 |
| MsG0780040108.01 | -3.02087 | 2.02E-05 | 0.00056 | Chr7 | 73139529 | 73148265 | 2069 |
| MsG0180000479.01 | -2.94902 | 2.03E-05 | 0.00056 | Chr1 | 6639934 | 6642243 | 1167 |
| novel.1745 | 4.21603 | 2.03E-05 | 0.00056 | Chr2 | 13992009 | 13995743 | 1193 |
| MsG0480018415.01 | 1.092146 | 2.03E-05 | 0.00056 | Chr4 | 4647267 | 4654150 | 826 |
| MsG0180005889.01 | 1.988218 | 2.04E-05 | 0.000562 | Chr1 | 97175452 | 97182551 | 2604 |
| novel.4290 | 1.674418 | 2.05E-05 | 0.000566 | Chr5 | 1891522 | 1892620 | 554 |
| novel.4775 | 6.730168 | 2.08E-05 | 0.000571 | Chr5 | 90618324 | 90619472 | 980 |
| MsG0580025627.01 | -2.18412 | 2.08E-05 | 0.000571 | Chr5 | 21453711 | 21465761 | 3242 |
| MsG0780040676.01 | -1.5779 | 2.08E-05 | 0.000571 | Chr7 | 80984782 | 80994274 | 1584 |
| MsG0280009109.01 | 1.09539 | 2.08E-05 | 0.000571 | Chr2 | 45494526 | 45496327 | 1068 |
| novel.3092 | -3.99625 | 2.10E-05 | 0.000576 | Chr3 | 85540083 | 85541827 | 1584 |
| MsG0680031403.01 | -1.84822 | 2.10E-05 | 0.000576 | Chr6 | 19938566 | 19940828 | 2263 |
| novel.7968 | 3.306021 | 2.10E-05 | 0.000576 | Chr8 | 32365023 | 32367478 | 1414 |
| novel.8355 | 6.603036 | 2.12E-05 | 0.000579 | Chr8 | 10179607 | 10180302 | 696 |
| MsG0180005362.01 | 6.996421 | 2.13E-05 | 0.000581 | Chr1 | 90855527 | 90856545 | 624 |
| MsG0680034729.01 | 2.764284 | 2.14E-05 | 0.000584 | Chr6 | 93530150 | 93532171 | 1436 |
| MsG0480018522.01 | -2.43511 | 2.15E-05 | 0.000587 | Chr4 | 6107925 | 6108503 | 579 |
| MsG0380013729.01 | -2.97025 | 2.16E-05 | 0.000588 | Chr3 | 44694340 | 44698909 | 1245 |
| MsG0280009992.01 | -1.2915 | 2.16E-05 | 0.000588 | Chr2 | 62534412 | 62557766 | 2548 |
| MsG0380015447.01 | -1.55591 | 2.17E-05 | 0.000589 | Chr3 | 70296270 | 70298682 | 1510 |
| MsG0280011429.01 | 4.152234 | 2.17E-05 | 0.000589 | Chr2 | 83812831 | 83823146 | 1791 |
| novel.458 | 1.03959 | 2.18E-05 | 0.00059 | Chr1 | 83816289 | 83819636 | 1034 |
| MsG0580028422.01 | -2.42544 | 2.19E-05 | 0.000593 | Chr5 | 80318890 | 80322412 | 999 |
| novel.9356 | 2.162146 | 2.20E-05 | 0.000595 | contig642end | 22469 | 23848 | 506 |
| MsG0780036125.01 | 3.192678 | 2.20E-05 | 0.000595 | Chr7 | 3116088 | 3126561 | 3582 |
| novel.8378 | -5.60871 | 2.20E-05 | 0.000596 | Chr8 | 15001106 | 15003603 | 1874 |
| MsG0680034255.01 | -3.803 | 2.22E-05 | 0.0006 | Chr6 | 84522721 | 84527831 | 816 |
| novel.388 | -3.87831 | 2.25E-05 | 0.000607 | Chr1 | 69852484 | 69854335 | 604 |
| MsG0780039078.01 | -2.0658 | 2.27E-05 | 0.000612 | Chr7 | 57591745 | 57593160 | 540 |
| MsG0380014527.01 | -7.36922 | 2.28E-05 | 0.000614 | Chr3 | 56058914 | 56063492 | 2688 |
| MsG0680034647.01 | -1.40406 | 2.31E-05 | 0.000623 | Chr6 | 92057184 | 92060962 | 3019 |
| novel.3968 | -4.59269 | 2.33E-05 | 0.000626 | Chr4 | 47855823 | 47859948 | 2699 |
| novel.4980 | 2.432724 | 2.34E-05 | 0.000628 | Chr5 | 12827207 | 12827815 | 609 |
| novel.2787 | -4.50712 | 2.34E-05 | 0.000628 | Chr3 | 20756624 | 20759525 | 881 |
| MsG0180003192.01 | 4.108701 | 2.38E-05 | 0.000639 | Chr1 | 58152964 | 58153272 | 309 |
| MsG0480018729.01 | -3.87135 | 2.38E-05 | 0.000639 | Chr4 | 9058688 | 9061388 | 2214 |
| MsG0880043912.01 | -2.50944 | 2.39E-05 | 0.00064 | Chr8 | 32476989 | 32479251 | 717 |
| MsG0880045135.01 | -3.56934 | 2.49E-05 | 0.000667 | Chr8 | 54686887 | 54691575 | 788 |
| MsG0480019244.01 | 1.678804 | 2.51E-05 | 0.000671 | Chr4 | 16880842 | 16891058 | 3552 |
| MsG0880043345.01 | -3.18471 | 2.51E-05 | 0.000671 | Chr8 | 23410286 | 23417575 | 3782 |
| MsG0480018497.01 | 1.142174 | 2.51E-05 | 0.000671 | Chr4 | 5815441 | 5830092 | 6213 |
| novel.936 | -6.19307 | 2.52E-05 | 0.000671 | Chr1 | 65918240 | 65920753 | 1066 |
| MsG0680031381.01 | -2.22538 | 2.52E-05 | 0.000671 | Chr6 | 19544180 | 19549680 | 1881 |
| MsG0180006226.01 | 1.401334 | 2.53E-05 | 0.000673 | Chr1 | 1.02E+08 | 1.02E+08 | 520 |
| novel.4609 | -3.79729 | 2.55E-05 | 0.000677 | Chr5 | 59048447 | 59050276 | 1346 |
| MsG0580024534.01 | 2.408261 | 2.55E-05 | 0.000678 | Chr5 | 6110527 | 6113993 | 1317 |
| MsG0180001490.01 | -3.76573 | 2.55E-05 | 0.000678 | Chr1 | 22181027 | 22181341 | 315 |
| MsG0580024600.01 | -3.27445 | 2.56E-05 | 0.00068 | Chr5 | 6778127 | 6778498 | 372 |
| MsG0580028287.01 | -2.29176 | 2.57E-05 | 0.000682 | Chr5 | 77830318 | 77833152 | 1464 |
| novel.4296 | -3.82172 | 2.58E-05 | 0.000684 | Chr5 | 2678330 | 2684897 | 390 |
| novel.4259 | 6.663838 | 2.58E-05 | 0.000684 | Chr4 | 90869211 | 90871503 | 336 |
| MsG0480018538.01 | -4.34043 | 2.60E-05 | 0.000688 | Chr4 | 6314629 | 6322141 | 3714 |
| MsG0580027899.01 | -1.74028 | 2.61E-05 | 0.000688 | Chr5 | 70655812 | 70658140 | 1271 |
| MsG0180005401.01 | -6.89262 | 2.62E-05 | 0.000691 | Chr1 | 91412330 | 91416739 | 2509 |
| novel.6161 | 6.543363 | 2.62E-05 | 0.000692 | Chr6 | 4869942 | 4870795 | 854 |
| novel.9198 | 6.575026 | 2.66E-05 | 0.0007 | contig541end | 12595 | 13701 | 1107 |
| MsG0280006894.01 | 1.252035 | 2.68E-05 | 0.000706 | Chr2 | 7712494 | 7715721 | 981 |
| MsG0680031466.01 | 4.730577 | 2.68E-05 | 0.000706 | Chr6 | 20860980 | 20862115 | 696 |
| MsG0880045441.01 | -1.35999 | 2.70E-05 | 0.00071 | Chr8 | 59182388 | 59184400 | 1188 |
| MsG0180005810.01 | 1.256283 | 2.72E-05 | 0.000714 | Chr1 | 96421519 | 96431762 | 2880 |
| novel.8311 | -6.64469 | 2.73E-05 | 0.000714 | Chr8 | 2408774 | 2410448 | 1675 |
| MsG0180006211.01 | 1.409058 | 2.73E-05 | 0.000714 | Chr1 | 1.01E+08 | 1.01E+08 | 593 |
| MsG0480021005.01 | -2.20466 | 2.73E-05 | 0.000714 | Chr4 | 51960330 | 51963788 | 2710 |
| MsG0680033554.01 | -3.69703 | 2.73E-05 | 0.000714 | Chr6 | 68105102 | 68122392 | 1299 |
| MsG0480019238.01 | -4.8714 | 2.73E-05 | 0.000714 | Chr4 | 16717098 | 16721663 | 1766 |
| MsG0180004399.01 | -1.63817 | 2.74E-05 | 0.000715 | Chr1 | 77488708 | 77490384 | 1677 |
| novel.3592 | -4.13862 | 2.77E-05 | 0.000723 | Chr4 | 72773820 | 72775944 | 1276 |
| novel.391 | 2.39501 | 2.77E-05 | 0.000723 | Chr1 | 70403020 | 70407406 | 1470 |
| novel.5785 | 7.497137 | 2.78E-05 | 0.000724 | Chr6 | 52303583 | 52305859 | 2277 |
| MsG0280009648.01 | -1.76579 | 2.79E-05 | 0.000727 | Chr2 | 55767593 | 55768495 | 903 |
| novel.3566 | 6.593567 | 2.80E-05 | 0.000729 | Chr4 | 68281209 | 68282925 | 1136 |
| novel.809 | 1.561138 | 2.81E-05 | 0.000731 | Chr1 | 33291256 | 33295648 | 2720 |
| MsG0480022412.01 | -2.62963 | 2.83E-05 | 0.000735 | Chr4 | 72231406 | 72233147 | 1326 |
| MsG0880042551.01 | -2.24227 | 2.85E-05 | 0.000739 | Chr8 | 10423109 | 10429468 | 442 |
| MsG0480023068.01 | -6.49795 | 2.87E-05 | 0.000743 | Chr4 | 80381028 | 80381207 | 180 |
| MsG0780039186.01 | -1.55371 | 2.89E-05 | 0.000748 | Chr7 | 59335794 | 59339993 | 1814 |
| novel.5273 | 3.097538 | 2.90E-05 | 0.00075 | Chr5 | 73377872 | 73380586 | 1202 |
| novel.1981 | -6.41389 | 2.90E-05 | 0.00075 | Chr2 | 65715647 | 65716855 | 1209 |
| MsG0180001399.01 | 2.007867 | 2.91E-05 | 0.000752 | Chr1 | 20565237 | 20575492 | 5098 |
| novel.4397 | -2.00835 | 2.91E-05 | 0.000752 | Chr5 | 17875369 | 17877597 | 2229 |
| MsG0780037779.01 | 1.708132 | 2.92E-05 | 0.000752 | Chr7 | 33082562 | 33086144 | 876 |
| novel.190 | -1.77426 | 2.92E-05 | 0.000752 | Chr1 | 26136797 | 26139937 | 1731 |
| MsG0380014094.01 | -7.18788 | 2.92E-05 | 0.000752 | Chr3 | 49582829 | 49590798 | 2359 |
| MsG0180001864.01 | 1.841725 | 2.93E-05 | 0.000754 | Chr1 | 28381073 | 28385301 | 1002 |
| novel.4849 | -3.91355 | 2.95E-05 | 0.000757 | Chr5 | 1.01E+08 | 1.01E+08 | 2144 |
| MsG0380014815.01 | -4.45993 | 2.98E-05 | 0.000765 | Chr3 | 60560710 | 60570257 | 2532 |
| MsG0580028184.01 | 1.091741 | 2.99E-05 | 0.000766 | Chr5 | 76266807 | 76271689 | 844 |
| novel.2408 | -4.10592 | 2.99E-05 | 0.000766 | Chr3 | 61139101 | 61140566 | 1466 |
| MsG0780036556.01 | -1.30437 | 2.99E-05 | 0.000766 | Chr7 | 9747734 | 9749181 | 1341 |
| MsG0480021406.01 | -1.14964 | 3.01E-05 | 0.000771 | Chr4 | 57575418 | 57580007 | 2030 |
| MsG0480020971.01 | -2.59842 | 3.07E-05 | 0.000784 | Chr4 | 51347342 | 51351622 | 1191 |
| novel.2238 | 3.020209 | 3.07E-05 | 0.000785 | Chr3 | 18827609 | 18832366 | 529 |
| MsG0780038544.01 | -3.63551 | 3.07E-05 | 0.000785 | Chr7 | 48480090 | 48483240 | 2421 |
| novel.6221 | 2.046732 | 3.08E-05 | 0.000785 | Chr6 | 12439893 | 12447945 | 3380 |
| novel.8326 | 2.367013 | 3.08E-05 | 0.000785 | Chr8 | 5766650 | 5780121 | 3499 |
| MsG0680035769.01 | -2.18617 | 3.08E-05 | 0.000785 | Chr6 | 1.12E+08 | 1.12E+08 | 1167 |
| novel.2960 | 5.411266 | 3.12E-05 | 0.000794 | Chr3 | 62596381 | 62599409 | 844 |
| MsG0680030786.01 | -2.38409 | 3.14E-05 | 0.000797 | Chr6 | 9309536 | 9316609 | 1651 |
| novel.5719 | 2.778179 | 3.15E-05 | 0.000801 | Chr6 | 37218565 | 37219253 | 689 |
| MsG0680031300.01 | -9.72489 | 3.22E-05 | 0.000818 | Chr6 | 17666882 | 17667100 | 219 |
| novel.8886 | -1.42211 | 3.23E-05 | 0.000819 | contig252end | 33970 | 41322 | 1530 |
| MsG0680030692.01 | -2.66502 | 3.26E-05 | 0.000827 | Chr6 | 7277008 | 7280214 | 3207 |
| MsG0680030529.01 | -4.57565 | 3.28E-05 | 0.00083 | Chr6 | 4757190 | 4760342 | 864 |
| MsG0480020628.01 | -2.85141 | 3.31E-05 | 0.000837 | Chr4 | 44957932 | 44959000 | 282 |
| MsG0080048229.01 | 2.25364 | 3.31E-05 | 0.000837 | contig247end | 7457 | 7657 | 171 |
| MsG0580027926.01 | -1.61955 | 3.36E-05 | 0.000848 | Chr5 | 71197887 | 71203620 | 1230 |
| novel.5760 | -2.07145 | 3.38E-05 | 0.000852 | Chr6 | 48427548 | 48439291 | 1712 |
| MsG0380011523.01 | -3.44642 | 3.41E-05 | 0.00086 | Chr3 | 934337 | 940282 | 721 |
| novel.3356 | -6.61002 | 3.47E-05 | 0.000874 | Chr4 | 24882615 | 24883970 | 690 |
| MsG0480018338.01 | -1.92141 | 3.48E-05 | 0.000877 | Chr4 | 3507756 | 3509494 | 531 |
| MsG0480019006.01 | -1.40349 | 3.49E-05 | 0.000878 | Chr4 | 13220573 | 13223820 | 1011 |
| MsG0780037414.01 | -3.02453 | 3.51E-05 | 0.000882 | Chr7 | 25399657 | 25401254 | 1131 |
| MsG0280007370.01 | 1.247341 | 3.51E-05 | 0.000882 | Chr2 | 14636124 | 14638600 | 438 |
| novel.5976 | -6.55283 | 3.53E-05 | 0.000885 | Chr6 | 92925867 | 92931128 | 2597 |
| MsG0880042477.01 | -2.36922 | 3.56E-05 | 0.000892 | Chr8 | 9351781 | 9353617 | 1767 |
| MsG0580029671.01 | 2.645246 | 3.57E-05 | 0.000893 | Chr5 | 1.01E+08 | 1.01E+08 | 540 |
| novel.8517 | -2.55523 | 3.57E-05 | 0.000893 | Chr8 | 45896150 | 45898208 | 1811 |
| novel.3758 | -5.69342 | 3.60E-05 | 0.0009 | Chr4 | 1588504 | 1589793 | 853 |
| MsG0480019037.01 | 2.246815 | 3.61E-05 | 0.000902 | Chr4 | 13632962 | 13636030 | 3015 |
| MsG0180005533.01 | -1.60152 | 3.69E-05 | 0.00092 | Chr1 | 92951697 | 92956676 | 1121 |
| MsG0680035788.01 | -1.95252 | 3.72E-05 | 0.000926 | Chr6 | 1.12E+08 | 1.12E+08 | 270 |
| MsG0780041020.01 | 1.144267 | 3.72E-05 | 0.000927 | Chr7 | 85604151 | 85608641 | 486 |
| MsG0680033196.01 | -6.68795 | 3.73E-05 | 0.000929 | Chr6 | 59169948 | 59170961 | 1014 |
| MsG0780036912.01 | 2.179274 | 3.78E-05 | 0.00094 | Chr7 | 16175374 | 16177165 | 513 |
| MsG0780041281.01 | -4.80144 | 3.80E-05 | 0.000945 | Chr7 | 89138931 | 89144912 | 2169 |
| MsG0780037151.01 | 2.218058 | 3.81E-05 | 0.000945 | Chr7 | 20737184 | 20743342 | 825 |
| MsG0180004594.01 | 2.756284 | 3.83E-05 | 0.000951 | Chr1 | 80107616 | 80114843 | 3108 |
| MsG0380012869.01 | 1.545774 | 3.85E-05 | 0.000953 | Chr3 | 25938220 | 25958534 | 3498 |
| novel.6650 | -6.50812 | 3.86E-05 | 0.000955 | Chr6 | 95209396 | 95214518 | 1002 |
| novel.4515 | -1.80643 | 3.87E-05 | 0.000957 | Chr5 | 40956068 | 40961742 | 1632 |
| MsG0180006065.01 | 6.494263 | 3.90E-05 | 0.000964 | Chr1 | 99612395 | 99624997 | 1431 |
| MsG0280010588.01 | -1.86987 | 3.92E-05 | 0.000967 | Chr2 | 71821029 | 71824141 | 1275 |
| novel.7624 | 3.97564 | 3.94E-05 | 0.000973 | Chr7 | 72482242 | 72486804 | 1014 |
| MsG0580028453.01 | 1.794464 | 3.96E-05 | 0.000976 | Chr5 | 80728166 | 80730545 | 1523 |
| novel.7717 | -2.11771 | 4.00E-05 | 0.000984 | Chr7 | 85421112 | 85425739 | 1254 |
| novel.1866 | -2.59896 | 4.00E-05 | 0.000984 | Chr2 | 36291045 | 36293180 | 1111 |
| novel.7020 | -6.67102 | 4.01E-05 | 0.000985 | Chr7 | 50997508 | 50998937 | 653 |
| MsG0480020453.01 | -2.45336 | 4.04E-05 | 0.000992 | Chr4 | 41761004 | 41768248 | 3920 |
| MsG0880043205.01 | -7.69966 | 4.05E-05 | 0.000993 | Chr8 | 20583565 | 20588292 | 1545 |
| novel.7062 | 1.739802 | 4.06E-05 | 0.000995 | Chr7 | 61465113 | 61467982 | 2707 |
| novel.531 | -6.48465 | 4.07E-05 | 0.000998 | Chr1 | 93999231 | 94000169 | 819 |
| novel.2165 | -2.74389 | 4.10E-05 | 0.001003 | Chr3 | 7744411 | 7745963 | 840 |
| MsG0880044284.01 | 1.837469 | 4.17E-05 | 0.001021 | Chr8 | 39456057 | 39464463 | 3133 |
| MsG0080048397.01 | -2.10509 | 4.22E-05 | 0.001032 | contig2end | 987 | 7443 | 1759 |
| MsG0380014401.01 | -1.62653 | 4.24E-05 | 0.001037 | Chr3 | 54185553 | 54192880 | 4701 |
| MsG0580028452.01 | 1.336029 | 4.30E-05 | 0.00105 | Chr5 | 80717112 | 80720508 | 450 |
| MsG0180003084.01 | -6.43506 | 4.32E-05 | 0.001054 | Chr1 | 56361361 | 56369941 | 768 |
| MsG0280009877.01 | 1.851567 | 4.37E-05 | 0.001064 | Chr2 | 59793451 | 59794180 | 444 |
| MsG0580025305.01 | 6.800974 | 4.37E-05 | 0.001065 | Chr5 | 16744952 | 16745722 | 621 |
| MsG0680030629.01 | 1.961398 | 4.38E-05 | 0.001066 | Chr6 | 6389754 | 6405001 | 1791 |
| MsG0780037803.01 | -2.56705 | 4.39E-05 | 0.001066 | Chr7 | 33444330 | 33454010 | 831 |
| MsG0180002799.01 | 3.298149 | 4.40E-05 | 0.001068 | Chr1 | 45457280 | 45457786 | 507 |
| MsG0780039179.01 | 1.436481 | 4.41E-05 | 0.00107 | Chr7 | 59283454 | 59286288 | 944 |
| MsG0580029038.01 | 1.045099 | 4.43E-05 | 0.001074 | Chr5 | 90784552 | 90786672 | 1182 |
| MsG0880044626.01 | 1.509736 | 4.46E-05 | 0.00108 | Chr8 | 45878527 | 45882268 | 2382 |
| novel.3989 | -1.73516 | 4.46E-05 | 0.00108 | Chr4 | 52485613 | 52487001 | 492 |
| MsG0180004021.01 | 3.300011 | 4.49E-05 | 0.001086 | Chr1 | 71981377 | 71988291 | 2093 |
| novel.8550 | -4.85264 | 4.53E-05 | 0.001094 | Chr8 | 52045401 | 52046481 | 795 |
| novel.8418 | -7.5254 | 4.54E-05 | 0.001095 | Chr8 | 23053435 | 23054229 | 795 |
| MsG0580025883.01 | 1.425399 | 4.54E-05 | 0.001095 | Chr5 | 25903068 | 25914954 | 1260 |
| MsG0780036850.01 | 2.853737 | 4.54E-05 | 0.001095 | Chr7 | 15032731 | 15033180 | 450 |
| MsG0180000549.01 | 1.399683 | 4.55E-05 | 0.001097 | Chr1 | 7693334 | 7697790 | 504 |
| MsG0880042096.01 | -3.62854 | 4.60E-05 | 0.001107 | Chr8 | 3627679 | 3637767 | 1884 |
| novel.8769 | -2.80835 | 4.62E-05 | 0.001112 | Chr8 | 88581071 | 88582075 | 538 |
| MsG0580025583.01 | -5.35551 | 4.65E-05 | 0.001118 | Chr5 | 20824190 | 20825854 | 1589 |
| MsG0880042019.01 | -3.32342 | 4.66E-05 | 0.001119 | Chr8 | 2616121 | 2623550 | 496 |
| MsG0780036121.01 | -2.58322 | 4.66E-05 | 0.001119 | Chr7 | 2980571 | 2992182 | 3300 |
| MsG0880044655.01 | -6.07711 | 4.67E-05 | 0.001119 | Chr8 | 46776266 | 46788991 | 3174 |
| MsG0180001655.01 | 2.087237 | 4.72E-05 | 0.001131 | Chr1 | 24771178 | 24773178 | 2001 |
| MsG0480021998.01 | 1.495531 | 4.72E-05 | 0.001131 | Chr4 | 66945945 | 66946676 | 732 |
| MsG0280006839.01 | 2.980834 | 4.75E-05 | 0.001135 | Chr2 | 6973531 | 6974580 | 591 |
| MsG0780035965.01 | 1.215851 | 4.75E-05 | 0.001135 | Chr7 | 713376 | 719489 | 1592 |
| MsG0280009578.01 | 1.175898 | 4.75E-05 | 0.001136 | Chr2 | 54562837 | 54568101 | 760 |
| MsG0180002286.01 | 1.826424 | 4.80E-05 | 0.001146 | Chr1 | 36272602 | 36283780 | 1626 |
| MsG0280010831.01 | -1.79503 | 4.82E-05 | 0.001151 | Chr2 | 75551631 | 75555275 | 1864 |
| novel.4128 | -4.18545 | 4.89E-05 | 0.001165 | Chr4 | 73439501 | 73441798 | 2298 |
| MsG0780040045.01 | 1.153414 | 4.90E-05 | 0.001166 | Chr7 | 72415764 | 72416300 | 537 |
| novel.4943 | -3.41052 | 4.99E-05 | 0.001187 | Chr5 | 5798287 | 5800208 | 668 |
| novel.4142 | 4.611653 | 4.99E-05 | 0.001187 | Chr4 | 75647072 | 75648031 | 480 |
| MsG0080049100.01 | 1.780163 | 5.07E-05 | 0.001204 | contig6end | 34205 | 34696 | 492 |
| MsG0880042913.01 | 1.322287 | 5.07E-05 | 0.001204 | Chr8 | 15819670 | 15823727 | 1018 |
| MsG0080048726.01 | -2.50632 | 5.11E-05 | 0.001213 | contig408end | 101814 | 109368 | 2892 |
| novel.6590 | 1.299909 | 5.13E-05 | 0.001217 | Chr6 | 81727758 | 81730201 | 1804 |
| novel.3450 | 1.870226 | 5.15E-05 | 0.001219 | Chr4 | 46798979 | 46803184 | 980 |
| novel.6611 | -5.90713 | 5.15E-05 | 0.001219 | Chr6 | 85497269 | 85499637 | 1249 |
| MsG0480021233.01 | -1.11414 | 5.18E-05 | 0.001226 | Chr4 | 55542028 | 55545095 | 2295 |
| MsG0480020963.01 | -2.37736 | 5.20E-05 | 0.001228 | Chr4 | 51264854 | 51270893 | 1574 |
| MsG0880043930.01 | -3.55748 | 5.20E-05 | 0.001228 | Chr8 | 32835642 | 32863606 | 5286 |
| MsG0580030033.01 | 2.517588 | 5.21E-05 | 0.001229 | Chr5 | 1.05E+08 | 1.05E+08 | 1704 |
| MsG0380015373.01 | -1.2879 | 5.21E-05 | 0.001229 | Chr3 | 69193215 | 69197220 | 1590 |
| MsG0480020575.01 | -7.4885 | 5.35E-05 | 0.00126 | Chr4 | 43658258 | 43661201 | 1311 |
| MsG0780037413.01 | -1.91144 | 5.37E-05 | 0.001265 | Chr7 | 25366268 | 25378100 | 1394 |
| novel.7691 | -4.95123 | 5.41E-05 | 0.001274 | Chr7 | 82910235 | 82914100 | 2537 |
| MsG0180005911.01 | -2.66389 | 5.43E-05 | 0.001276 | Chr1 | 97456031 | 97457640 | 275 |
| MsG0580025637.01 | -2.92464 | 5.43E-05 | 0.001276 | Chr5 | 21609587 | 21609927 | 249 |
| MsG0280009427.01 | 2.009357 | 5.45E-05 | 0.00128 | Chr2 | 51743775 | 51744356 | 582 |
| MsG0580028038.01 | 6.748151 | 5.47E-05 | 0.001282 | Chr5 | 73624310 | 73628357 | 1491 |
| MsG0880043911.01 | -5.21659 | 5.51E-05 | 0.001291 | Chr8 | 32473137 | 32476405 | 807 |
| MsG0180003129.01 | -1.98917 | 5.55E-05 | 0.0013 | Chr1 | 57043504 | 57045872 | 588 |
| novel.1883 | -5.82655 | 5.56E-05 | 0.0013 | Chr2 | 39945805 | 39950936 | 703 |
| MsG0180004704.01 | -1.53952 | 5.58E-05 | 0.001306 | Chr1 | 81562133 | 81567701 | 4122 |
| MsG0780041652.01 | -1.34687 | 5.63E-05 | 0.001315 | Chr7 | 93486328 | 93487136 | 621 |
| MsG0680035569.01 | 6.611389 | 5.72E-05 | 0.001335 | Chr6 | 1.09E+08 | 1.09E+08 | 1185 |
| MsG0480021060.01 | -1.01707 | 5.76E-05 | 0.001345 | Chr4 | 52796842 | 52802514 | 1553 |
| novel.1791 | -1.73482 | 5.83E-05 | 0.001359 | Chr2 | 22272311 | 22278582 | 2690 |
| MsG0380015181.01 | 1.916778 | 5.86E-05 | 0.001365 | Chr3 | 66377844 | 66387002 | 1220 |
| novel.5297 | -4.57974 | 5.87E-05 | 0.001366 | Chr5 | 77240789 | 77243417 | 839 |
| MsG0380015352.01 | -1.61118 | 5.89E-05 | 0.00137 | Chr3 | 68908303 | 68911052 | 1088 |
| novel.1161 | -6.28728 | 5.95E-05 | 0.001382 | Chr2 | 455527 | 457718 | 725 |
| MsG0480018285.01 | -1.46704 | 5.98E-05 | 0.001388 | Chr4 | 2782675 | 2786651 | 1204 |
| MsG0180002953.01 | -5.11414 | 5.99E-05 | 0.001388 | Chr1 | 53720732 | 53724911 | 1842 |
| MsG0580025041.01 | -4.59717 | 5.99E-05 | 0.001388 | Chr5 | 13176055 | 13179743 | 2000 |
| novel.946 | -3.49042 | 5.99E-05 | 0.001388 | Chr1 | 67494948 | 67497157 | 1963 |
| MsG0380012363.01 | 2.325185 | 6.04E-05 | 0.001399 | Chr3 | 15468117 | 15470494 | 891 |
| novel.566 | -2.73056 | 6.07E-05 | 0.001405 | Chr1 | 1.01E+08 | 1.01E+08 | 1465 |
| MsG0380012946.01 | 6.926198 | 6.08E-05 | 0.001405 | Chr3 | 27444237 | 27454329 | 1015 |
| novel.909 | 6.456797 | 6.15E-05 | 0.00142 | Chr1 | 60928858 | 60933156 | 1294 |
| MsG0780040612.01 | -2.61255 | 6.16E-05 | 0.001421 | Chr7 | 79986618 | 79987088 | 471 |
| MsG0880045672.01 | -1.13267 | 6.17E-05 | 0.001421 | Chr8 | 62497717 | 62502480 | 2443 |
| novel.4551 | -6.47067 | 6.17E-05 | 0.001421 | Chr5 | 47123561 | 47140044 | 456 |
| MsG0380012412.01 | -10.2052 | 6.18E-05 | 0.001422 | Chr3 | 16079360 | 16080445 | 1086 |
| MsG0480018270.01 | 6.396198 | 6.18E-05 | 0.001422 | Chr4 | 2621924 | 2622535 | 612 |
| novel.5207 | 6.510825 | 6.21E-05 | 0.001428 | Chr5 | 60846135 | 60847203 | 528 |
| MsG0480018867.01 | -3.36709 | 6.21E-05 | 0.001428 | Chr4 | 10791657 | 10796362 | 1904 |
| MsG0880044906.01 | -1.9612 | 6.23E-05 | 0.001431 | Chr8 | 51216179 | 51218966 | 968 |
| MsG0580028956.01 | -5.41631 | 6.25E-05 | 0.001433 | Chr5 | 89563946 | 89564773 | 828 |
| MsG0880045796.01 | -2.87056 | 6.30E-05 | 0.001444 | Chr8 | 64591406 | 64594584 | 786 |
| novel.297 | -6.52887 | 6.32E-05 | 0.001447 | Chr1 | 55404192 | 55412305 | 681 |
| MsG0580024812.01 | -1.26956 | 6.33E-05 | 0.001447 | Chr5 | 10086473 | 10093726 | 2453 |
| novel.5638 | 1.478179 | 6.34E-05 | 0.001449 | Chr6 | 20013737 | 20017791 | 2680 |
| novel.6019 | 2.490741 | 6.39E-05 | 0.001459 | Chr6 | 1E+08 | 1E+08 | 567 |
| MsG0280010055.01 | -3.9998 | 6.39E-05 | 0.001459 | Chr2 | 63555467 | 63556774 | 1308 |
| MsG0380014756.01 | -2.54891 | 6.40E-05 | 0.00146 | Chr3 | 59497797 | 59501007 | 1049 |
| MsG0380015588.01 | 1.139417 | 6.42E-05 | 0.001462 | Chr3 | 72230105 | 72238411 | 1542 |
| novel.9177 | 1.366972 | 6.44E-05 | 0.001466 | contig531end | 10028 | 17474 | 1822 |
| MsG0380012834.01 | 2.114784 | 6.45E-05 | 0.001466 | Chr3 | 25302936 | 25306660 | 3540 |
| MsG0780040506.01 | 6.488368 | 6.45E-05 | 0.001466 | Chr7 | 78490087 | 78492681 | 2466 |
| MsG0480019123.01 | 4.44918 | 6.55E-05 | 0.001488 | Chr4 | 14814159 | 14815268 | 1110 |
| novel.8627 | -1.99502 | 6.57E-05 | 0.001492 | Chr8 | 64892659 | 64895210 | 906 |
| MsG0380017161.01 | -1.39608 | 6.60E-05 | 0.001497 | Chr3 | 92812593 | 92816817 | 1944 |
| MsG0380017469.01 | -1.89536 | 6.62E-05 | 0.0015 | Chr3 | 96625557 | 96628519 | 1528 |
| MsG0480023308.01 | -1.15912 | 6.63E-05 | 0.001502 | Chr4 | 83590820 | 83596677 | 1924 |
| MsG0180001654.01 | 2.791174 | 6.72E-05 | 0.001522 | Chr1 | 24761115 | 24763919 | 2805 |
| MsG0780041377.01 | 6.690452 | 6.73E-05 | 0.001523 | Chr7 | 90219951 | 90236237 | 2517 |
| novel.6996 | 6.596551 | 6.81E-05 | 0.001539 | Chr7 | 46429960 | 46445330 | 611 |
| MsG0580029950.01 | -1.41104 | 6.83E-05 | 0.001541 | Chr5 | 1.04E+08 | 1.04E+08 | 966 |
| novel.8483 | 6.351629 | 6.83E-05 | 0.001541 | Chr8 | 37402927 | 37404085 | 708 |
| MsG0180005999.01 | -1.73166 | 6.84E-05 | 0.001542 | Chr1 | 98636732 | 98638524 | 801 |
| MsG0680034847.01 | -1.79693 | 6.87E-05 | 0.001548 | Chr6 | 95459143 | 95462619 | 2841 |
| novel.8408 | -7.08302 | 6.93E-05 | 0.00156 | Chr8 | 20601667 | 20603452 | 1786 |
| MsG0580025256.01 | 1.434018 | 6.93E-05 | 0.00156 | Chr5 | 16030305 | 16031574 | 699 |
| MsG0680035832.01 | -1.66546 | 6.95E-05 | 0.001563 | Chr6 | 1.13E+08 | 1.13E+08 | 620 |
| novel.1645 | -2.26126 | 6.96E-05 | 0.001563 | Chr2 | 931734 | 937138 | 1899 |
| novel.4188 | 1.170211 | 7.01E-05 | 0.001574 | Chr4 | 82801114 | 82803876 | 986 |
| MsG0880043897.01 | 4.439758 | 7.05E-05 | 0.001583 | Chr8 | 32320156 | 32320629 | 474 |
| MsG0680032680.01 | -3.62201 | 7.11E-05 | 0.001594 | Chr6 | 47499103 | 47502601 | 3399 |
| MsG0180000671.01 | -2.6395 | 7.12E-05 | 0.001594 | Chr1 | 9520504 | 9522097 | 608 |
| novel.4121 | -2.62864 | 7.12E-05 | 0.001595 | Chr4 | 71163124 | 71166124 | 1086 |
| MsG0680030694.01 | 1.723098 | 7.17E-05 | 0.001604 | Chr6 | 7286266 | 7287966 | 1701 |
| novel.8341 | 1.381347 | 7.19E-05 | 0.001607 | Chr8 | 8626257 | 8630495 | 3077 |
| novel.8844 | 2.147274 | 7.24E-05 | 0.001616 | contig178end | 20714 | 24166 | 1388 |
| novel.945 | 6.36594 | 7.24E-05 | 0.001616 | Chr1 | 67201020 | 67206892 | 762 |
| MsG0880043898.01 | -2.1247 | 7.25E-05 | 0.001616 | Chr8 | 32326612 | 32329906 | 888 |
| MsG0580027132.01 | -5.32701 | 7.25E-05 | 0.001617 | Chr5 | 54145493 | 54146248 | 756 |
| novel.6874 | -1.93328 | 7.27E-05 | 0.001619 | Chr7 | 19006908 | 19010033 | 1369 |
| MsG0880042745.01 | 1.90952 | 7.29E-05 | 0.001622 | Chr8 | 13465765 | 13468382 | 1826 |
| MsG0780038493.01 | 1.080569 | 7.29E-05 | 0.001622 | Chr7 | 47657992 | 47664474 | 1802 |
| MsG0180005791.01 | 1.928373 | 7.41E-05 | 0.001647 | Chr1 | 96178085 | 96179660 | 1576 |
| novel.4279 | 6.640461 | 7.44E-05 | 0.001652 | Chr4 | 92562778 | 92563928 | 304 |
| novel.4029 | -4.17935 | 7.48E-05 | 0.001661 | Chr4 | 57965551 | 58020677 | 2961 |
| MsG0780037070.01 | -6.39679 | 7.50E-05 | 0.001663 | Chr7 | 18922737 | 18927252 | 3570 |
| MsG0080047818.01 | -7.10003 | 7.51E-05 | 0.001665 | contig113end | 37545 | 50036 | 1122 |
| MsG0480023417.01 | 2.688952 | 7.55E-05 | 0.001671 | Chr4 | 84950823 | 84951137 | 315 |
| MsG0580025873.01 | -2.45856 | 7.55E-05 | 0.001671 | Chr5 | 25662130 | 25663257 | 1128 |
| MsG0180003992.01 | 2.103322 | 7.68E-05 | 0.001699 | Chr1 | 71478406 | 71479710 | 1305 |
| MsG0180002055.01 | 4.322745 | 7.71E-05 | 0.001703 | Chr1 | 31726935 | 31731017 | 2388 |
| novel.3194 | 6.613852 | 7.75E-05 | 0.001712 | Chr3 | 1E+08 | 1.01E+08 | 917 |
| MsG0280006769.01 | 1.234667 | 7.80E-05 | 0.001721 | Chr2 | 6001869 | 6004232 | 1336 |
| MsG0780038233.01 | 6.339613 | 7.83E-05 | 0.001727 | Chr7 | 42444088 | 42444327 | 240 |
| MsG0480020973.01 | -1.89966 | 7.87E-05 | 0.001733 | Chr4 | 51381606 | 51389094 | 2140 |
| MsG0280008038.01 | -3.76793 | 7.87E-05 | 0.001733 | Chr2 | 24555296 | 24561945 | 3204 |
| MsG0080047873.01 | -2.0961 | 7.90E-05 | 0.001739 | contig131end | 21746 | 24604 | 882 |
| MsG0680032313.01 | -2.74582 | 7.95E-05 | 0.001748 | Chr6 | 38310380 | 38313526 | 2127 |
| novel.6858 | -3.17971 | 7.98E-05 | 0.001753 | Chr7 | 16182536 | 16183647 | 1112 |
| novel.4192 | -6.43348 | 8.04E-05 | 0.001766 | Chr4 | 83786909 | 83789289 | 759 |
| novel.2157 | -2.49597 | 8.08E-05 | 0.001773 | Chr3 | 6335368 | 6337423 | 1355 |
| MsG0380014869.01 | -1.4884 | 8.09E-05 | 0.001773 | Chr3 | 61716673 | 61721090 | 3642 |
| MsG0680032297.01 | 3.389293 | 8.13E-05 | 0.001781 | Chr6 | 37952604 | 37955271 | 309 |
| MsG0480018565.01 | -2.30776 | 8.28E-05 | 0.00181 | Chr4 | 6664576 | 6669890 | 716 |
| MsG0680033739.01 | -5.77444 | 8.32E-05 | 0.001818 | Chr6 | 72446867 | 72456116 | 2148 |
| MsG0880044960.01 | -6.2825 | 8.33E-05 | 0.001821 | Chr8 | 52087947 | 52092251 | 1101 |
| novel.6447 | -6.33437 | 8.36E-05 | 0.001825 | Chr6 | 49316991 | 49320385 | 1466 |
| MsG0680035588.01 | -2.10356 | 8.38E-05 | 0.001829 | Chr6 | 1.09E+08 | 1.09E+08 | 465 |
| MsG0180003193.01 | 3.52612 | 8.44E-05 | 0.00184 | Chr1 | 58186097 | 58186423 | 327 |
| novel.6441 | -3.40178 | 8.47E-05 | 0.001845 | Chr6 | 47974722 | 47976572 | 1468 |
| novel.6462 | 2.00839 | 8.49E-05 | 0.001847 | Chr6 | 52962593 | 52964583 | 1907 |
| novel.6232 | -6.69603 | 8.51E-05 | 0.00185 | Chr6 | 13214831 | 13220466 | 954 |
| novel.540 | 5.665323 | 8.52E-05 | 0.00185 | Chr1 | 96227895 | 96235296 | 1110 |
| MsG0380012867.01 | -2.15449 | 8.57E-05 | 0.00186 | Chr3 | 25927026 | 25929443 | 2280 |
| MsG0480020883.01 | 1.283906 | 8.58E-05 | 0.00186 | Chr4 | 50058632 | 50063214 | 2155 |
| MsG0480018743.01 | -1.98353 | 8.61E-05 | 0.001864 | Chr4 | 9284804 | 9308609 | 2782 |
| MsG0880045427.01 | -1.50677 | 8.61E-05 | 0.001864 | Chr8 | 59013315 | 59018297 | 1777 |
| MsG0680035893.01 | -2.42775 | 8.69E-05 | 0.001881 | Chr6 | 1.14E+08 | 1.14E+08 | 1653 |
| MsG0580026905.01 | 5.891958 | 8.79E-05 | 0.0019 | Chr5 | 47813735 | 47815951 | 2217 |
| MsG0680030777.01 | 2.599 | 8.79E-05 | 0.0019 | Chr6 | 9065660 | 9072345 | 1047 |
| MsG0680035831.01 | -1.2437 | 8.81E-05 | 0.001903 | Chr6 | 1.13E+08 | 1.13E+08 | 2563 |
| MsG0880043271.01 | 3.447424 | 8.85E-05 | 0.00191 | Chr8 | 21825589 | 21829593 | 861 |
| novel.5375 | -6.69801 | 8.86E-05 | 0.001912 | Chr5 | 91237491 | 91238368 | 878 |
| MsG0380013404.01 | -2.83244 | 8.94E-05 | 0.001926 | Chr3 | 36808044 | 36815588 | 5477 |
| MsG0780040681.01 | -1.99775 | 8.95E-05 | 0.001928 | Chr7 | 81089757 | 81097357 | 593 |
| novel.3507 | -2.25577 | 8.96E-05 | 0.001928 | Chr4 | 57492127 | 57497195 | 977 |
| MsG0880043639.01 | 2.241982 | 9.01E-05 | 0.001938 | Chr8 | 28235363 | 28236126 | 300 |
| novel.1158 | -2.00165 | 9.06E-05 | 0.001947 | Chr1 | 1.02E+08 | 1.02E+08 | 3313 |
| MsG0880043286.01 | 4.289566 | 9.09E-05 | 0.001953 | Chr8 | 21985126 | 21985677 | 552 |
| novel.7884 | -4.30391 | 9.10E-05 | 0.001953 | Chr8 | 17354573 | 17356785 | 690 |
| MsG0480019219.01 | 1.63507 | 9.12E-05 | 0.001955 | Chr4 | 16347994 | 16353683 | 1032 |
| MsG0180001063.01 | 1.243772 | 9.16E-05 | 0.001963 | Chr1 | 15415621 | 15420238 | 934 |
| MsG0480019772.01 | -1.07156 | 9.19E-05 | 0.001967 | Chr4 | 26806557 | 26815214 | 2663 |
| MsG0280011026.01 | 2.855655 | 9.20E-05 | 0.00197 | Chr2 | 78570298 | 78572998 | 1056 |
| novel.7030 | 6.938013 | 9.22E-05 | 0.001971 | Chr7 | 53082107 | 53083710 | 1604 |
| novel.3686 | 1.507087 | 9.27E-05 | 0.001981 | Chr4 | 84822305 | 84824749 | 681 |
| MsG0280007567.01 | -3.08625 | 9.30E-05 | 0.001986 | Chr2 | 17366880 | 17374482 | 1053 |
| MsG0880047361.01 | 1.632968 | 9.32E-05 | 0.001987 | Chr8 | 85259040 | 85259699 | 660 |
| MsG0180000520.01 | -3.44175 | 9.41E-05 | 0.002005 | Chr1 | 7345158 | 7346397 | 471 |
| novel.3512 | 2.776563 | 9.43E-05 | 0.002007 | Chr4 | 57872678 | 57877641 | 1981 |
| MsG0180004151.01 | 1.239242 | 9.45E-05 | 0.002011 | Chr1 | 74056997 | 74081223 | 4742 |
| MsG0480018730.01 | 6.356634 | 9.45E-05 | 0.002011 | Chr4 | 9077666 | 9078497 | 756 |
| MsG0780040692.01 | 1.858218 | 9.46E-05 | 0.002011 | Chr7 | 81267378 | 81272189 | 1193 |
| MsG0280006530.01 | 2.076264 | 9.53E-05 | 0.002025 | Chr2 | 3172184 | 3173707 | 1524 |
| MsG0480022105.01 | -2.40727 | 9.56E-05 | 0.00203 | Chr4 | 68295955 | 68299255 | 2832 |
| novel.2317 | -2.12449 | 9.62E-05 | 0.002039 | Chr3 | 36636380 | 36637785 | 868 |
| novel.7883 | -6.3362 | 9.62E-05 | 0.002039 | Chr8 | 17217248 | 17218736 | 652 |
| novel.9116 | 2.214018 | 9.69E-05 | 0.002053 | contig497end | 37 | 3716 | 1074 |
| MsG0880044519.01 | 3.676937 | 9.72E-05 | 0.002057 | Chr8 | 43644081 | 43652701 | 1098 |
| MsG0580025629.01 | -5.44115 | 9.85E-05 | 0.002082 | Chr5 | 21471053 | 21471640 | 588 |
| MsG0480022884.01 | -3.1073 | 9.85E-05 | 0.002082 | Chr4 | 78056071 | 78058661 | 1098 |
| MsG0380015646.01 | 1.258038 | 9.92E-05 | 0.002094 | Chr3 | 73002045 | 73007079 | 1589 |
| novel.1602 | -6.54556 | 9.92E-05 | 0.002094 | Chr2 | 80039564 | 80046342 | 273 |
| novel.2938 | 1.718158 | 9.93E-05 | 0.002094 | Chr3 | 57890185 | 57891109 | 925 |
| MsG0080048553.01 | -3.15484 | 0.0001 | 0.002109 | contig352end | 964 | 6934 | 579 |
| novel.3763 | -6.02762 | 0.0001 | 0.002114 | Chr4 | 2163775 | 2166823 | 606 |
| MsG0580029657.01 | 1.03796 | 0.000101 | 0.002122 | Chr5 | 1E+08 | 1E+08 | 1127 |
| MsG0680032842.01 | -3.0615 | 0.000101 | 0.002122 | Chr6 | 50819585 | 50836650 | 2802 |
| MsG0780036187.01 | 2.132844 | 0.000101 | 0.002123 | Chr7 | 4459215 | 4464465 | 3402 |
| novel.1867 | -2.48223 | 0.000102 | 0.002136 | Chr2 | 36294869 | 36296905 | 937 |
| MsG0480018115.01 | -2.70154 | 0.000103 | 0.002153 | Chr4 | 373808 | 377237 | 528 |
| novel.9280 | 2.29905 | 0.000103 | 0.002153 | contig591end | 18136 | 20769 | 1658 |
| MsG0580027603.01 | -2.96812 | 0.000103 | 0.002163 | Chr5 | 64097880 | 64121112 | 3681 |
| novel.681 | 6.289127 | 0.000104 | 0.002182 | Chr1 | 10277897 | 10279598 | 1477 |
| MsG0180000571.01 | 3.424588 | 0.000105 | 0.002188 | Chr1 | 7987784 | 7988464 | 681 |
| MsG0280006401.01 | 1.729 | 0.000105 | 0.002188 | Chr2 | 1371757 | 1372828 | 466 |
| MsG0780041790.01 | 1.231813 | 0.000105 | 0.002194 | Chr7 | 95180596 | 95180940 | 345 |
| novel.4658 | -6.24397 | 0.000106 | 0.002203 | Chr5 | 70926283 | 70927311 | 728 |
| MsG0480018134.01 | -1.72284 | 0.000106 | 0.002203 | Chr4 | 660221 | 670460 | 2345 |
| MsG0280008098.01 | 1.43069 | 0.000106 | 0.002204 | Chr2 | 25454846 | 25458439 | 1050 |
| novel.719 | 4.312972 | 0.000107 | 0.002221 | Chr1 | 16674873 | 16675529 | 657 |
| MsG0580025403.01 | 1.50776 | 0.000107 | 0.002228 | Chr5 | 18095459 | 18103360 | 3525 |
| MsG0580028469.01 | -1.53767 | 0.000107 | 0.002236 | Chr5 | 80930687 | 80937444 | 3597 |
| MsG0580028160.01 | -1.72066 | 0.000108 | 0.002237 | Chr5 | 75919468 | 75923079 | 2487 |
| MsG0480019056.01 | 2.763742 | 0.000108 | 0.002241 | Chr4 | 13970375 | 13981228 | 3531 |
| MsG0880042650.01 | -1.56775 | 0.000108 | 0.002245 | Chr8 | 11987879 | 11989923 | 1286 |
| novel.4989 | 3.643382 | 0.000108 | 0.002249 | Chr5 | 14190699 | 14191510 | 566 |
| MsG0380014467.01 | -1.93258 | 0.000108 | 0.002249 | Chr3 | 55236250 | 55237260 | 1011 |
| MsG0180005456.01 | -5.78728 | 0.000109 | 0.002249 | Chr1 | 92064893 | 92071183 | 1017 |
| novel.4608 | -6.38911 | 0.000109 | 0.002253 | Chr5 | 59047453 | 59048298 | 302 |
| novel.4881 | 5.748391 | 0.00011 | 0.002267 | Chr5 | 1.06E+08 | 1.06E+08 | 267 |
| MsG0180001414.01 | -2.90352 | 0.00011 | 0.002281 | Chr1 | 20786417 | 20801755 | 6684 |
| MsG0380013366.01 | -2.91245 | 0.000113 | 0.002326 | Chr3 | 35942990 | 35944116 | 705 |
| MsG0880045365.01 | 1.007185 | 0.000113 | 0.002336 | Chr8 | 58182626 | 58188539 | 2158 |
| MsG0880043187.01 | -1.23954 | 0.000113 | 0.002336 | Chr8 | 20314838 | 20319827 | 1087 |
| novel.5653 | 1.65624 | 0.000115 | 0.002365 | Chr6 | 23429053 | 23432305 | 830 |
| novel.267 | -7.14165 | 0.000115 | 0.002366 | Chr1 | 44860626 | 44862032 | 1407 |
| MsG0380018034.01 | 1.040793 | 0.000115 | 0.00237 | Chr3 | 1.04E+08 | 1.04E+08 | 2444 |
| MsG0880044636.01 | -1.32886 | 0.000116 | 0.002378 | Chr8 | 46051511 | 46057130 | 2271 |
| MsG0680032200.01 | 1.626064 | 0.000116 | 0.002382 | Chr6 | 36032719 | 36040757 | 2937 |
| MsG0180004381.01 | -1.22424 | 0.000116 | 0.002382 | Chr1 | 77248420 | 77251450 | 1771 |
| novel.2918 | -5.73818 | 0.000117 | 0.002398 | Chr3 | 52063611 | 52064964 | 373 |
| MsG0480021397.01 | -2.27742 | 0.000117 | 0.002398 | Chr4 | 57386535 | 57388642 | 1056 |
| novel.6467 | -3.71036 | 0.000117 | 0.002404 | Chr6 | 53649357 | 53656306 | 3095 |
| MsG0380012188.01 | -4.61207 | 0.000118 | 0.002414 | Chr3 | 12392836 | 12394443 | 489 |
| novel.1042 | 3.56428 | 0.000119 | 0.002445 | Chr1 | 87207395 | 87210319 | 739 |
| MsG0180003118.01 | -1.74795 | 0.00012 | 0.002457 | Chr1 | 56863598 | 56867493 | 777 |
| MsG0880044615.01 | 5.758098 | 0.00012 | 0.00246 | Chr8 | 45790573 | 45795124 | 1110 |
| novel.6837 | -1.71153 | 0.00012 | 0.002461 | Chr7 | 12146661 | 12150052 | 1706 |
| MsG0580028394.01 | -1.82387 | 0.000121 | 0.002463 | Chr5 | 79916818 | 79927030 | 2495 |
| MsG0380011638.01 | -6.40064 | 0.000121 | 0.002465 | Chr3 | 2882082 | 2887356 | 2676 |
| novel.8063 | -6.25016 | 0.000121 | 0.002465 | Chr8 | 52258265 | 52259091 | 827 |
| novel.7310 | 1.301384 | 0.000121 | 0.002465 | Chr7 | 2111578 | 2114462 | 575 |
| novel.6503 | -4.8289 | 0.000121 | 0.002467 | Chr6 | 62599699 | 62621692 | 1442 |
| novel.2956 | -4.44908 | 0.000122 | 0.002477 | Chr3 | 61661640 | 61664145 | 2506 |
| novel.8696 | 6.731227 | 0.000123 | 0.002491 | Chr8 | 76531979 | 76534253 | 737 |
| MsG0080048216.01 | 2.945235 | 0.000123 | 0.002503 | contig245end | 22402 | 24039 | 649 |
| novel.2733 | -6.21221 | 0.000123 | 0.002503 | Chr3 | 9533969 | 9536841 | 937 |
| MsG0480020253.01 | -3.8768 | 0.000124 | 0.002513 | Chr4 | 37447535 | 37454855 | 1157 |
| novel.3296 | 3.539871 | 0.000124 | 0.002513 | Chr4 | 13708653 | 13713303 | 1145 |
| novel.218 | -6.35514 | 0.000125 | 0.002532 | Chr1 | 34094572 | 34095854 | 1283 |
| MsG0180004270.01 | -3.47779 | 0.000125 | 0.002532 | Chr1 | 75841907 | 75846078 | 1054 |
| MsG0180001466.01 | -2.18277 | 0.000125 | 0.002532 | Chr1 | 21768295 | 21779691 | 7041 |
| MsG0880042443.01 | -1.64132 | 0.000127 | 0.002561 | Chr8 | 8768488 | 8772996 | 3516 |
| novel.7478 | -6.37216 | 0.000127 | 0.002563 | Chr7 | 41354167 | 41354829 | 663 |
| MsG0580024535.01 | 3.310625 | 0.000127 | 0.002571 | Chr5 | 6118582 | 6120774 | 1028 |
| MsG0480022548.01 | 4.193636 | 0.000128 | 0.002578 | Chr4 | 73978919 | 73986324 | 1014 |
| MsG0180003117.01 | -1.79639 | 0.000128 | 0.002583 | Chr1 | 56858366 | 56861810 | 896 |
| MsG0880047151.01 | -4.0325 | 0.000128 | 0.002583 | Chr8 | 82703387 | 82703620 | 234 |
| MsG0780038439.01 | -6.76194 | 0.000128 | 0.002588 | Chr7 | 46870125 | 46871355 | 435 |
| MsG0080048605.01 | -1.81249 | 0.000129 | 0.002588 | contig371end | 1899 | 11497 | 2052 |
| MsG0880043444.01 | 2.655485 | 0.00013 | 0.002607 | Chr8 | 24918523 | 24921803 | 1498 |
| MsG0180002006.01 | -1.44848 | 0.00013 | 0.002615 | Chr1 | 30885377 | 30893909 | 4988 |
| MsG0280010343.01 | 4.108939 | 0.000131 | 0.002624 | Chr2 | 68332435 | 68346701 | 7524 |
| MsG0680034469.01 | 6.364299 | 0.000131 | 0.002624 | Chr6 | 88055038 | 88056687 | 1650 |
| novel.4712 | -2.95109 | 0.000131 | 0.002637 | Chr5 | 79904948 | 79906449 | 1502 |
| MsG0380015069.01 | 4.14603 | 0.000132 | 0.002656 | Chr3 | 64736270 | 64737911 | 962 |
| MsG0380015146.01 | -2.29006 | 0.000133 | 0.002671 | Chr3 | 65877813 | 65879205 | 545 |
| MsG0180001138.01 | 1.095674 | 0.000134 | 0.002687 | Chr1 | 16443266 | 16448339 | 2159 |
| novel.8318 | -6.62894 | 0.000136 | 0.002716 | Chr8 | 3277758 | 3280082 | 848 |
| MsG0780040061.01 | 1.157232 | 0.000136 | 0.002724 | Chr7 | 72566185 | 72567909 | 951 |
| novel.753 | -4.13317 | 0.000137 | 0.002728 | Chr1 | 22992331 | 22995900 | 3570 |
| novel.2358 | 1.797666 | 0.000137 | 0.002728 | Chr3 | 47730425 | 47735037 | 1058 |
| MsG0580025814.01 | -2.71664 | 0.000137 | 0.002728 | Chr5 | 24592553 | 24595526 | 1736 |
| MsG0880045476.01 | -3.33947 | 0.000138 | 0.002751 | Chr8 | 59683788 | 59684258 | 471 |
| MsG0380012895.01 | -6.6848 | 0.000138 | 0.002751 | Chr3 | 26545903 | 26549770 | 3663 |
| novel.1250 | -3.34906 | 0.000139 | 0.002764 | Chr2 | 14569478 | 14571563 | 1030 |
| MsG0780036291.01 | 1.991007 | 0.000139 | 0.002768 | Chr7 | 6072548 | 6073156 | 609 |
| novel.5661 | -3.34905 | 0.000139 | 0.002768 | Chr6 | 24240067 | 24248570 | 1271 |
| MsG0680034206.01 | 1.994669 | 0.00014 | 0.002778 | Chr6 | 83602564 | 83602971 | 408 |
| MsG0280009644.01 | -3.7828 | 0.00014 | 0.002781 | Chr2 | 55747641 | 55754092 | 2004 |
| novel.3585 | -3.16118 | 0.00014 | 0.002782 | Chr4 | 72123747 | 72125092 | 1346 |
| MsG0580025589.01 | 1.226702 | 0.00014 | 0.002782 | Chr5 | 20921765 | 20926914 | 2145 |
| novel.6388 | 3.071911 | 0.000141 | 0.002782 | Chr6 | 38561616 | 38564662 | 1539 |
| MsG0080048717.01 | -2.36299 | 0.000141 | 0.002782 | contig408end | 42503 | 46149 | 3131 |
| MsG0880047158.01 | -3.48322 | 0.000141 | 0.002798 | Chr8 | 82787758 | 82803660 | 3610 |
| novel.1558 | 2.055919 | 0.000142 | 0.002808 | Chr2 | 75718636 | 75721528 | 2373 |
| MsG0180002800.01 | -1.75002 | 0.000142 | 0.002808 | Chr1 | 45458563 | 45467199 | 1725 |
| MsG0680033624.01 | 2.239401 | 0.000142 | 0.00281 | Chr6 | 69709793 | 69720375 | 1410 |
| novel.7246 | 2.786907 | 0.000142 | 0.002811 | Chr7 | 89412966 | 89413503 | 310 |
| MsG0680032009.01 | -2.57845 | 0.000143 | 0.00282 | Chr6 | 31854968 | 31858076 | 1162 |
| novel.7606 | 1.479322 | 0.000143 | 0.002825 | Chr7 | 69512145 | 69516437 | 1298 |
| MsG0780039336.01 | -2.83208 | 0.000145 | 0.002849 | Chr7 | 62183918 | 62185337 | 930 |
| MsG0780037603.01 | 1.325858 | 0.000145 | 0.002851 | Chr7 | 29207450 | 29212126 | 1769 |
| novel.7087 | -3.45407 | 0.000145 | 0.002857 | Chr7 | 66244189 | 66247384 | 1792 |
| MsG0280009858.01 | -2.81114 | 0.000146 | 0.002872 | Chr2 | 59471603 | 59475346 | 1811 |
| MsG0580025841.01 | 3.535927 | 0.000147 | 0.00289 | Chr5 | 24949732 | 24953862 | 3048 |
| novel.128 | 1.529215 | 0.000148 | 0.002898 | Chr1 | 16882776 | 16885944 | 1490 |
| MsG0680032380.01 | -1.39688 | 0.000148 | 0.0029 | Chr6 | 39710946 | 39715132 | 1115 |
| MsG0780036864.01 | -3.36042 | 0.000148 | 0.0029 | Chr7 | 15262038 | 15267605 | 1041 |
| MsG0480018356.01 | 2.268981 | 0.000149 | 0.002906 | Chr4 | 3748334 | 3749053 | 720 |
| novel.5659 | 3.680362 | 0.000149 | 0.002909 | Chr6 | 23935058 | 23944563 | 976 |
| MsG0780036010.01 | -3.83828 | 0.000149 | 0.002914 | Chr7 | 1295611 | 1300839 | 1206 |
| MsG0580025623.01 | -1.5518 | 0.000149 | 0.002918 | Chr5 | 21401148 | 21407888 | 2568 |
| MsG0180000784.01 | 2.631732 | 0.00015 | 0.002928 | Chr1 | 11132370 | 11132582 | 213 |
| MsG0880042616.01 | -6.49931 | 0.00015 | 0.002928 | Chr8 | 11512868 | 11513194 | 327 |
| MsG0080048030.01 | 3.933791 | 0.000151 | 0.002948 | contig177end | 17016 | 23786 | 1190 |
| MsG0380011985.01 | 2.297665 | 0.000152 | 0.002951 | Chr3 | 8835986 | 8841700 | 3117 |
| MsG0480023152.01 | 1.39492 | 0.000152 | 0.002951 | Chr4 | 81502207 | 81507204 | 479 |
| MsG0580024268.01 | 1.468051 | 0.000152 | 0.00296 | Chr5 | 2767218 | 2770093 | 1503 |
| novel.2996 | -7.1662 | 0.000153 | 0.002968 | Chr3 | 70716817 | 70718793 | 1681 |
| novel.5939 | 6.36433 | 0.000153 | 0.002982 | Chr6 | 86042193 | 86043206 | 834 |
| novel.3621 | 6.499721 | 0.000154 | 0.002985 | Chr4 | 77118885 | 77119581 | 697 |
| novel.8075 | 6.397211 | 0.000154 | 0.002986 | Chr8 | 55280968 | 55281849 | 673 |
| novel.9334 | 1.451657 | 0.000154 | 0.002986 | contig628end | 3831 | 8852 | 1703 |
| novel.6807 | 3.982903 | 0.000155 | 0.003001 | Chr7 | 3107498 | 3115416 | 1140 |
| MsG0480018713.01 | -4.1814 | 0.000157 | 0.003032 | Chr4 | 8871718 | 8884930 | 5988 |
| MsG0780038803.01 | 1.735883 | 0.000157 | 0.003044 | Chr7 | 53261550 | 53263217 | 1413 |
| MsG0780036455.01 | 2.368689 | 0.000157 | 0.003045 | Chr7 | 8362215 | 8371677 | 3501 |
| MsG0380013913.01 | -1.60449 | 0.000159 | 0.003066 | Chr3 | 48232892 | 48238758 | 1556 |
| MsG0280008748.01 | -2.2694 | 0.000159 | 0.003069 | Chr2 | 37637069 | 37637761 | 693 |
| novel.5282 | -2.52014 | 0.00016 | 0.003087 | Chr5 | 74758768 | 74764417 | 5650 |
| novel.7982 | 1.840269 | 0.00016 | 0.003092 | Chr8 | 36700592 | 36721790 | 3158 |
| MsG0380014470.01 | -1.87642 | 0.000161 | 0.003102 | Chr3 | 55286407 | 55289782 | 931 |
| MsG0280008675.01 | 2.959312 | 0.000161 | 0.003108 | Chr2 | 36149099 | 36155485 | 1887 |
| novel.7199 | 6.228044 | 0.000161 | 0.003108 | Chr7 | 82906912 | 82908517 | 1501 |
| novel.1847 | 6.311776 | 0.000162 | 0.003115 | Chr2 | 32964765 | 32966082 | 732 |
| MsG0680030904.01 | -3.62043 | 0.000162 | 0.003115 | Chr6 | 11382714 | 11385936 | 516 |
| novel.6384 | 2.998072 | 0.000162 | 0.003121 | Chr6 | 37333789 | 38242392 | 1074 |
| MsG0180004899.01 | -4.13807 | 0.000163 | 0.003122 | Chr1 | 84156329 | 84156628 | 300 |
| MsG0880042763.01 | -4.12899 | 0.000163 | 0.003126 | Chr8 | 13694968 | 13696903 | 324 |
| novel.568 | -2.09326 | 0.000163 | 0.003129 | Chr1 | 1.01E+08 | 1.01E+08 | 2092 |
| MsG0880044545.01 | 1.538298 | 0.000163 | 0.003134 | Chr8 | 44150802 | 44165731 | 3237 |
| MsG0580028029.01 | -3.23307 | 0.000165 | 0.00316 | Chr5 | 73365001 | 73370876 | 1521 |
| MsG0780036812.01 | -3.15077 | 0.000165 | 0.003165 | Chr7 | 14322161 | 14324958 | 2008 |
| MsG0380017520.01 | 1.117833 | 0.000168 | 0.003209 | Chr3 | 97409335 | 97410345 | 1011 |
| MsG0580025303.01 | 6.237575 | 0.000169 | 0.003226 | Chr5 | 16715057 | 16719450 | 1467 |
| MsG0380012185.01 | 1.25168 | 0.000169 | 0.00323 | Chr3 | 12375783 | 12383958 | 2053 |
| novel.748 | -6.48714 | 0.000169 | 0.003232 | Chr1 | 22357881 | 22360290 | 1140 |
| MsG0280010020.01 | -6.84299 | 0.00017 | 0.003246 | Chr2 | 62963344 | 62964532 | 711 |
| MsG0780035990.01 | 1.17015 | 0.00017 | 0.003246 | Chr7 | 1001196 | 1002170 | 975 |
| novel.3622 | 6.28667 | 0.000171 | 0.003254 | Chr4 | 77119720 | 77120533 | 700 |
| novel.7538 | 2.557518 | 0.000172 | 0.003269 | Chr7 | 58601269 | 58602573 | 1274 |
| MsG0580029354.01 | -2.29824 | 0.000173 | 0.003296 | Chr5 | 95908987 | 95911532 | 1068 |
| novel.8619 | 2.399642 | 0.000173 | 0.0033 | Chr8 | 63466998 | 63468267 | 986 |
| MsG0380016655.01 | 2.080537 | 0.000174 | 0.003303 | Chr3 | 86172496 | 86172750 | 255 |
| MsG0380012429.01 | -3.87037 | 0.000174 | 0.003307 | Chr3 | 16409251 | 16415019 | 1822 |
| MsG0880043288.01 | 3.320547 | 0.000175 | 0.003321 | Chr8 | 22004289 | 22004840 | 552 |
| MsG0480018303.01 | 5.637102 | 0.000176 | 0.003336 | Chr4 | 3131876 | 3135380 | 999 |
| novel.1865 | -2.17304 | 0.000176 | 0.003343 | Chr2 | 36275834 | 36279540 | 2715 |
| MsG0280009635.01 | -1.71517 | 0.000176 | 0.003343 | Chr2 | 55668616 | 55678516 | 1642 |
| novel.101 | 2.464592 | 0.000177 | 0.003348 | Chr1 | 12770084 | 12773998 | 2063 |
| novel.1417 | -5.81586 | 0.000177 | 0.003351 | Chr2 | 45905069 | 45907605 | 1395 |
| MsG0380017188.01 | -5.7201 | 0.000177 | 0.003351 | Chr3 | 93146649 | 93147835 | 566 |
| MsG0580025294.01 | -1.87758 | 0.000177 | 0.003353 | Chr5 | 16602914 | 16605498 | 2466 |
| MsG0780039133.01 | 2.094828 | 0.000177 | 0.003355 | Chr7 | 58428831 | 58434569 | 3672 |
| novel.4171 | -6.34465 | 0.000178 | 0.003357 | Chr4 | 80120052 | 80121141 | 1090 |
| MsG0380012254.01 | -4.42913 | 0.000179 | 0.003371 | Chr3 | 13397783 | 13402021 | 2439 |
| novel.5837 | -2.08718 | 0.000179 | 0.003371 | Chr6 | 66876058 | 66881705 | 1702 |
| MsG0480020505.01 | -1.53127 | 0.00018 | 0.003397 | Chr4 | 42535311 | 42536919 | 1506 |
| novel.4314 | 3.050996 | 0.00018 | 0.003399 | Chr5 | 4971365 | 4972995 | 592 |
| MsG0880044266.01 | -2.06006 | 0.000181 | 0.003401 | Chr8 | 38997999 | 38999685 | 468 |
| MsG0380016422.01 | 6.194692 | 0.000181 | 0.00341 | Chr3 | 83229743 | 83241669 | 4410 |
| MsG0480018606.01 | -1.19099 | 0.000182 | 0.003429 | Chr4 | 7281456 | 7287972 | 1905 |
| MsG0280010503.01 | -2.35182 | 0.000183 | 0.003433 | Chr2 | 70644565 | 70660889 | 2089 |
| MsG0680032070.01 | 6.282852 | 0.000184 | 0.003446 | Chr6 | 33052408 | 33058255 | 1380 |
| novel.9368 | -4.3832 | 0.000184 | 0.003451 | contig68end | 15965 | 17834 | 831 |
| MsG0880042391.01 | 1.799293 | 0.000187 | 0.003506 | Chr8 | 7992604 | 7999778 | 2425 |
| MsG0780036695.01 | -2.42814 | 0.000187 | 0.003507 | Chr7 | 12028304 | 12031744 | 1768 |
| MsG0480019447.01 | -1.87444 | 0.000188 | 0.00352 | Chr4 | 20524772 | 20546450 | 4758 |
| novel.8059 | -5.10362 | 0.000188 | 0.003521 | Chr8 | 52024313 | 52025769 | 559 |
| novel.7027 | 6.500497 | 0.000188 | 0.003522 | Chr7 | 52193803 | 52194829 | 814 |
| novel.6706 | -3.20894 | 0.00019 | 0.003549 | Chr6 | 1.03E+08 | 1.03E+08 | 1225 |
| MsG0680035643.01 | -3.25105 | 0.000191 | 0.003569 | Chr6 | 1.1E+08 | 1.1E+08 | 1410 |
| MsG0080048752.01 | -3.81416 | 0.000191 | 0.003573 | contig420end | 15636 | 16954 | 1068 |
| MsG0180001133.01 | -4.87673 | 0.000193 | 0.003606 | Chr1 | 16414903 | 16416426 | 1524 |
| MsG0280010182.01 | 1.554136 | 0.000194 | 0.003614 | Chr2 | 65840486 | 65840952 | 408 |
| MsG0180002092.01 | -4.93617 | 0.000194 | 0.003614 | Chr1 | 32766982 | 32778735 | 6483 |
| MsG0580024590.01 | -3.92488 | 0.000195 | 0.003622 | Chr5 | 6664054 | 6669797 | 588 |
| MsG0780039951.01 | 1.405647 | 0.000195 | 0.003622 | Chr7 | 71017617 | 71021658 | 1652 |
| MsG0880043497.01 | -1.94758 | 0.000196 | 0.003641 | Chr8 | 25661315 | 25661566 | 252 |
| MsG0380013769.01 | -2.76119 | 0.000197 | 0.003668 | Chr3 | 45401974 | 45404774 | 1458 |
| MsG0180001649.01 | 1.772621 | 0.000198 | 0.003678 | Chr1 | 24672106 | 24675155 | 2895 |
| novel.5438 | -6.46268 | 0.000199 | 0.003686 | Chr5 | 1E+08 | 1E+08 | 884 |
| novel.3142 | -4.29778 | 0.000199 | 0.003695 | Chr3 | 93939172 | 93942008 | 1629 |
| MsG0180006199.01 | -1.5209 | 0.000203 | 0.003758 | Chr1 | 1.01E+08 | 1.01E+08 | 2271 |
| MsG0580029432.01 | -1.65364 | 0.000203 | 0.003768 | Chr5 | 96913974 | 96920124 | 2151 |
| MsG0280007861.01 | -6.37444 | 0.000204 | 0.003768 | Chr2 | 21934259 | 21936064 | 1806 |
| MsG0880043844.01 | 6.134642 | 0.000204 | 0.003771 | Chr8 | 31510989 | 31511207 | 219 |
| novel.8918 | 5.681152 | 0.000205 | 0.003787 | contig289end | 17403 | 22782 | 399 |
| MsG0480018936.01 | -2.13241 | 0.000205 | 0.003792 | Chr4 | 11981065 | 11994521 | 6144 |
| novel.1086 | 2.424836 | 0.000207 | 0.003821 | Chr1 | 92973122 | 92974932 | 815 |
| MsG0380016570.01 | 1.830287 | 0.000207 | 0.003823 | Chr3 | 85259525 | 85259824 | 300 |
| MsG0880043158.01 | -1.3527 | 0.000208 | 0.003827 | Chr8 | 19892766 | 19896377 | 935 |
| novel.812 | -5.03311 | 0.000208 | 0.003835 | Chr1 | 34567490 | 34569932 | 2443 |
| MsG0880044315.01 | 1.297364 | 0.000208 | 0.003836 | Chr8 | 39881762 | 39886069 | 1876 |
| MsG0480020442.01 | -2.82663 | 0.00021 | 0.003854 | Chr4 | 41408107 | 41412582 | 1566 |
| novel.366 | -5.61918 | 0.00021 | 0.003854 | Chr1 | 66401475 | 66405114 | 1432 |
| MsG0080049138.01 | 1.366447 | 0.00021 | 0.003867 | contig92end | 10285 | 14892 | 1374 |
| novel.3843 | -3.47744 | 0.000211 | 0.003867 | Chr4 | 18253806 | 18261282 | 1035 |
| MsG0180002525.01 | -3.84773 | 0.000211 | 0.003867 | Chr1 | 39805202 | 39811124 | 1674 |
| MsG0880042645.01 | 2.594099 | 0.000211 | 0.003873 | Chr8 | 11907670 | 11912834 | 3014 |
| MsG0880047550.01 | -3.08579 | 0.000212 | 0.003878 | Chr8 | 87995486 | 87995944 | 459 |
| MsG0280009876.01 | 1.693148 | 0.000212 | 0.003879 | Chr2 | 59791122 | 59793416 | 2295 |
| MsG0180004167.01 | 2.208164 | 0.000213 | 0.003892 | Chr1 | 74330893 | 74334352 | 3042 |
| novel.6522 | -6.25831 | 0.000213 | 0.003896 | Chr6 | 66912284 | 66913243 | 279 |
| MsG0380012005.01 | -1.5385 | 0.000213 | 0.003902 | Chr3 | 9418398 | 9424434 | 1862 |
| MsG0880043652.01 | -6.24726 | 0.000213 | 0.003902 | Chr8 | 28446175 | 28447752 | 1578 |
| novel.5520 | 4.611613 | 0.000214 | 0.003904 | Chr6 | 2152467 | 2155135 | 1224 |
| MsG0480018965.01 | 2.678677 | 0.000215 | 0.003931 | Chr4 | 12555118 | 12556542 | 1425 |
| novel.4858 | -2.27911 | 0.000216 | 0.003931 | Chr5 | 1.02E+08 | 1.02E+08 | 578 |
| novel.5063 | -6.53179 | 0.000216 | 0.003931 | Chr5 | 29926045 | 29927133 | 1089 |
| novel.1041 | -3.0254 | 0.000216 | 0.003931 | Chr1 | 87198832 | 87203735 | 2692 |
| MsG0180002086.01 | -4.04229 | 0.000216 | 0.003935 | Chr1 | 32576761 | 32586303 | 3420 |
| MsG0880042634.01 | -2.10451 | 0.000217 | 0.003956 | Chr8 | 11745118 | 11748939 | 282 |
| MsG0180006222.01 | 1.247264 | 0.000218 | 0.003957 | Chr1 | 1.02E+08 | 1.02E+08 | 1590 |
| MsG0880046767.01 | 1.638822 | 0.000219 | 0.003989 | Chr8 | 77829029 | 77829919 | 429 |
| novel.250 | -5.55179 | 0.000221 | 0.00401 | Chr1 | 38949870 | 38950584 | 641 |
| MsG0380014940.01 | -1.5674 | 0.000221 | 0.00401 | Chr3 | 62812466 | 62816711 | 2034 |
| novel.2191 | -4.57437 | 0.000222 | 0.004023 | Chr3 | 11910001 | 11910354 | 354 |
| novel.3833 | -6.83571 | 0.000222 | 0.004023 | Chr4 | 16838531 | 16846615 | 994 |
| MsG0380013852.01 | -2.28973 | 0.000222 | 0.004028 | Chr3 | 46707389 | 46707604 | 216 |
| MsG0280010738.01 | -1.26119 | 0.000223 | 0.004046 | Chr2 | 74108606 | 74110648 | 2043 |
| MsG0680034990.01 | -2.97607 | 0.000225 | 0.004065 | Chr6 | 97408764 | 97413301 | 3766 |
| novel.7319 | 7.045223 | 0.000225 | 0.00407 | Chr7 | 5405053 | 5406754 | 1084 |
| novel.2489 | -6.17787 | 0.000226 | 0.00409 | Chr3 | 76576317 | 76577558 | 1242 |
| MsG0780040950.01 | 2.701216 | 0.000226 | 0.004093 | Chr7 | 84704015 | 84713058 | 3639 |
| MsG0180005648.01 | -1.4552 | 0.000229 | 0.004133 | Chr1 | 94452520 | 94453497 | 978 |
| MsG0880045022.01 | -1.22495 | 0.00023 | 0.004144 | Chr8 | 52865800 | 52881843 | 2847 |
| novel.1370 | 6.180256 | 0.00023 | 0.004149 | Chr2 | 34283959 | 34285927 | 868 |
| MsG0380016530.01 | -1.53417 | 0.00023 | 0.004156 | Chr3 | 84750091 | 84754153 | 1510 |
| MsG0780040984.01 | 1.505607 | 0.000232 | 0.004182 | Chr7 | 85193397 | 85196727 | 1290 |
| MsG0180002273.01 | 1.92902 | 0.000233 | 0.004194 | Chr1 | 36059054 | 36063197 | 1532 |
| MsG0180006086.01 | 1.683997 | 0.000233 | 0.004194 | Chr1 | 99993106 | 1E+08 | 2312 |
| MsG0780039883.01 | 6.144352 | 0.000233 | 0.004194 | Chr7 | 70078247 | 70078846 | 600 |
| MsG0480018669.01 | -1.29105 | 0.000234 | 0.004198 | Chr4 | 8433023 | 8443876 | 2616 |
| MsG0680030710.01 | -4.07085 | 0.000235 | 0.004215 | Chr6 | 7554979 | 7558328 | 1029 |
| novel.7013 | -2.01429 | 0.000235 | 0.004216 | Chr7 | 49342872 | 49345699 | 1712 |
| MsG0280008749.01 | 6.13409 | 0.000238 | 0.004259 | Chr2 | 37653250 | 37654470 | 1221 |
| MsG0480023341.01 | 2.975841 | 0.000238 | 0.004259 | Chr4 | 84003475 | 84007591 | 1428 |
| MsG0280009299.01 | -1.60715 | 0.000239 | 0.004284 | Chr2 | 49729581 | 49734351 | 1096 |
| MsG0380012007.01 | -2.58625 | 0.000239 | 0.004284 | Chr3 | 9438759 | 9445465 | 2082 |
| novel.4960 | -5.24737 | 0.00024 | 0.004288 | Chr5 | 10284430 | 10288826 | 1213 |
| MsG0280009531.01 | 6.368651 | 0.000241 | 0.0043 | Chr2 | 53852296 | 53853451 | 863 |
| MsG0480021666.01 | 2.253818 | 0.000241 | 0.004311 | Chr4 | 62613168 | 62614581 | 351 |
| novel.7103 | 1.811088 | 0.000242 | 0.004313 | Chr7 | 67916180 | 67919754 | 1413 |
| MsG0380011676.01 | 1.486193 | 0.000242 | 0.004326 | Chr3 | 3362664 | 3362972 | 309 |
| MsG0180005799.01 | 2.785041 | 0.000243 | 0.004328 | Chr1 | 96249075 | 96254198 | 1159 |
| MsG0780040083.01 | -7.06761 | 0.000243 | 0.004334 | Chr7 | 72900457 | 72903350 | 1039 |
| novel.8874 | -6.14224 | 0.000244 | 0.004344 | contig240end | 123783 | 124852 | 875 |
| novel.2327 | -6.1316 | 0.000244 | 0.004344 | Chr3 | 39323020 | 39330321 | 598 |
| MsG0780036454.01 | -6.12331 | 0.000245 | 0.004361 | Chr7 | 8333992 | 8338072 | 3162 |
| MsG0680030696.01 | 2.166518 | 0.000246 | 0.004378 | Chr6 | 7300716 | 7302119 | 1404 |
| novel.6222 | -2.14172 | 0.000247 | 0.00439 | Chr6 | 12483084 | 12487833 | 4140 |
| MsG0480018931.01 | 2.352314 | 0.000247 | 0.004392 | Chr4 | 11910128 | 11912023 | 1602 |
| MsG0380014845.01 | -6.38364 | 0.00025 | 0.004434 | Chr3 | 61102560 | 61120498 | 5424 |
| MsG0480018241.01 | -2.73414 | 0.00025 | 0.004437 | Chr4 | 2077386 | 2085603 | 2535 |
| MsG0780038865.01 | -6.13109 | 0.000251 | 0.00445 | Chr7 | 54348690 | 54348923 | 234 |
| MsG0280006492.01 | -1.15077 | 0.000251 | 0.00445 | Chr2 | 2701549 | 2708545 | 1086 |
| novel.5634 | 4.516708 | 0.000252 | 0.004455 | Chr6 | 19529805 | 19539818 | 674 |
| novel.5330 | -2.20908 | 0.000253 | 0.004464 | Chr5 | 83564983 | 83572843 | 5425 |
| MsG0880046072.01 | -1.34031 | 0.000253 | 0.004464 | Chr8 | 68631572 | 68644742 | 3161 |
| MsG0880046703.01 | 6.095432 | 0.000254 | 0.004481 | Chr8 | 77025400 | 77026210 | 309 |
| novel.3581 | 6.101846 | 0.000254 | 0.004482 | Chr4 | 70769716 | 70770258 | 543 |
| MsG0180001660.01 | -1.80237 | 0.000255 | 0.004497 | Chr1 | 24841052 | 24842947 | 802 |
| MsG0680033197.01 | -6.1139 | 0.000256 | 0.004514 | Chr6 | 59176272 | 59177906 | 1635 |
| novel.2633 | 1.143215 | 0.000257 | 0.004531 | Chr3 | 97394381 | 97399153 | 1963 |
| MsG0780039080.01 | -4.40751 | 0.000258 | 0.004541 | Chr7 | 57606514 | 57609454 | 1995 |
| MsG0480018607.01 | -2.35539 | 0.000259 | 0.004563 | Chr4 | 7301468 | 7306833 | 2122 |
| novel.9085 | 2.499648 | 0.00026 | 0.004572 | contig473end | 26426 | 31424 | 1259 |
| MsG0280010804.01 | -1.85423 | 0.000261 | 0.004594 | Chr2 | 75263812 | 75267407 | 1719 |
| MsG0180003982.01 | -2.97266 | 0.000261 | 0.004594 | Chr1 | 71347133 | 71350683 | 768 |
| MsG0580028913.01 | -1.3945 | 0.000263 | 0.004619 | Chr5 | 88530823 | 88542854 | 1230 |
| novel.7043 | 3.619829 | 0.000263 | 0.004619 | Chr7 | 57521449 | 57524981 | 3414 |
| novel.2569 | -6.32401 | 0.000263 | 0.00462 | Chr3 | 87627204 | 87628033 | 620 |
| novel.8349 | -1.63854 | 0.000264 | 0.004623 | Chr8 | 9702918 | 9709849 | 3140 |
| MsG0480018512.01 | -2.02888 | 0.000266 | 0.004664 | Chr4 | 6008045 | 6008638 | 594 |
| MsG0880042277.01 | -2.2748 | 0.000267 | 0.004668 | Chr8 | 6450221 | 6451384 | 1164 |
| novel.6732 | -5.51652 | 0.000267 | 0.004668 | Chr6 | 1.06E+08 | 1.06E+08 | 2063 |
| MsG0880045440.01 | 1.945233 | 0.000267 | 0.004672 | Chr8 | 59175271 | 59181512 | 1119 |
| MsG0680034075.01 | 6.404772 | 0.000268 | 0.004679 | Chr6 | 80132004 | 80135169 | 1278 |
| MsG0780037802.01 | 6.762708 | 0.000268 | 0.004684 | Chr7 | 33439223 | 33441598 | 603 |
| MsG0880042209.01 | -1.72176 | 0.000268 | 0.004685 | Chr8 | 5387643 | 5388940 | 501 |
| novel.1163 | -1.41311 | 0.00027 | 0.00471 | Chr2 | 1187248 | 1189882 | 2635 |
| novel.630 | 3.941626 | 0.000271 | 0.004723 | Chr1 | 4202284 | 4203559 | 1276 |
| MsG0680030722.01 | -2.91333 | 0.000271 | 0.004727 | Chr6 | 7953069 | 7963903 | 1660 |
| novel.974 | 6.210631 | 0.000272 | 0.004731 | Chr1 | 76952173 | 76952905 | 700 |
| MsG0880046434.01 | -6.11174 | 0.000272 | 0.004739 | Chr8 | 73339292 | 73339687 | 396 |
| MsG0180002332.01 | -3.58838 | 0.000272 | 0.00474 | Chr1 | 36860739 | 36861653 | 915 |
| novel.2698 | -1.0607 | 0.000273 | 0.004753 | Chr3 | 4642014 | 4653609 | 5094 |
| MsG0180004122.01 | -6.92322 | 0.000273 | 0.004753 | Chr1 | 73703796 | 73705538 | 1641 |
| MsG0480022777.01 | 2.747386 | 0.000274 | 0.004753 | Chr4 | 76547930 | 76548157 | 228 |
| MsG0280010413.01 | 2.07528 | 0.000274 | 0.004755 | Chr2 | 69172940 | 69179027 | 3297 |
| MsG0180004978.01 | 1.334877 | 0.000274 | 0.004755 | Chr1 | 85125777 | 85130432 | 4176 |
| novel.3246 | 4.72047 | 0.000276 | 0.004793 | Chr4 | 5194766 | 5201255 | 1394 |
| MsG0780041226.01 | -3.38027 | 0.000277 | 0.004798 | Chr7 | 88457894 | 88458424 | 531 |
| novel.7972 | -3.85015 | 0.000277 | 0.004805 | Chr8 | 34414398 | 34418262 | 1051 |
| MsG0380013854.01 | 1.357514 | 0.000279 | 0.004827 | Chr3 | 46713024 | 46721419 | 2003 |
| MsG0880043601.01 | -1.04233 | 0.000281 | 0.004854 | Chr8 | 27579595 | 27597994 | 4535 |
| MsG0480022573.01 | 4.456914 | 0.000283 | 0.004893 | Chr4 | 74271780 | 74272484 | 705 |
| novel.7510 | -6.38612 | 0.000284 | 0.004902 | Chr7 | 50159683 | 50160750 | 1068 |
| novel.9093 | -1.04308 | 0.000284 | 0.004902 | contig480end | 10045 | 11624 | 1224 |
| novel.1667 | -3.38277 | 0.000284 | 0.004907 | Chr2 | 2628199 | 2630527 | 1335 |
| novel.582 | -2.14478 | 0.000285 | 0.004921 | Chr1 | 657953 | 668235 | 1594 |
| novel.411 | -1.28163 | 0.000286 | 0.004922 | Chr1 | 75749169 | 75752359 | 1720 |
| MsG0280008590.01 | 1.479692 | 0.000286 | 0.004922 | Chr2 | 34426217 | 34429911 | 1035 |
| novel.3205 | -6.15852 | 0.000286 | 0.004927 | Chr3 | 1.02E+08 | 1.02E+08 | 694 |
| MsG0480018403.01 | -1.13686 | 0.000287 | 0.004933 | Chr4 | 4402071 | 4408290 | 701 |
| MsG0680033699.01 | 2.284111 | 0.000287 | 0.004933 | Chr6 | 71865035 | 71873480 | 2051 |
| MsG0580024620.01 | -2.74315 | 0.000288 | 0.004952 | Chr5 | 7064168 | 7064719 | 360 |
| novel.7350 | -1.27074 | 0.000288 | 0.004952 | Chr7 | 12915999 | 12919606 | 2582 |
| MsG0880043299.01 | -1.48045 | 0.000288 | 0.004952 | Chr8 | 22189190 | 22202278 | 3253 |
| MsG0480018963.01 | 2.043159 | 0.000288 | 0.004952 | Chr4 | 12537682 | 12541377 | 1859 |
| novel.8305 | -2.3426 | 0.00029 | 0.004972 | Chr8 | 1852089 | 1856786 | 840 |
| MsG0380015268.01 | -2.11691 | 0.00029 | 0.004976 | Chr3 | 67631024 | 67632165 | 1019 |
| MsG0580024553.01 | -1.01364 | 0.000291 | 0.00498 | Chr5 | 6292663 | 6293555 | 768 |
| novel.7407 | -5.93276 | 0.000291 | 0.00498 | Chr7 | 22074995 | 22077176 | 1006 |
| novel.305 | -3.10154 | 0.000291 | 0.004985 | Chr1 | 56881933 | 56884256 | 2129 |
| novel.3501 | -1.90387 | 0.000292 | 0.004993 | Chr4 | 56869753 | 56878088 | 6242 |
| novel.1141 | -1.25943 | 0.000293 | 0.005005 | Chr1 | 1.01E+08 | 1.01E+08 | 3895 |
| MsG0380012422.01 | 1.970693 | 0.000293 | 0.005007 | Chr3 | 16242223 | 16243136 | 729 |
| novel.3779 | -6.14836 | 0.000294 | 0.005019 | Chr4 | 4193192 | 4194672 | 444 |
| novel.3832 | 1.965676 | 0.000294 | 0.005021 | Chr4 | 16821427 | 16822829 | 1403 |
| MsG0280009826.01 | -2.44759 | 0.000295 | 0.005037 | Chr2 | 58942193 | 58955706 | 5874 |
| MsG0580025539.01 | -6.05398 | 0.000296 | 0.005044 | Chr5 | 20236540 | 20238907 | 1893 |
| novel.2976 | 6.253417 | 0.000296 | 0.005044 | Chr3 | 67444629 | 67446631 | 517 |
| novel.8954 | -6.03085 | 0.000296 | 0.005044 | contig355end | 15489 | 17971 | 887 |
| novel.3279 | 6.070471 | 0.000296 | 0.005044 | Chr4 | 10493223 | 10495725 | 933 |
| MsG0380012192.01 | 2.840406 | 0.000297 | 0.005056 | Chr3 | 12434693 | 12435730 | 1038 |
| novel.1631 | 1.466219 | 0.000299 | 0.005083 | Chr2 | 83102775 | 83106294 | 1958 |
| MsG0580028274.01 | -2.25649 | 0.0003 | 0.00509 | Chr5 | 77675039 | 77700782 | 6239 |
| MsG0480018139.01 | 2.078125 | 0.0003 | 0.00509 | Chr4 | 793900 | 797084 | 1818 |
| MsG0880046417.01 | 1.298553 | 0.0003 | 0.005092 | Chr8 | 73175649 | 73182952 | 1770 |
| novel.6717 | 4.886895 | 0.0003 | 0.005092 | Chr6 | 1.04E+08 | 1.04E+08 | 552 |
| novel.5782 | 1.685706 | 0.000301 | 0.005103 | Chr6 | 51656859 | 51658001 | 699 |
| MsG0680031048.01 | 1.258701 | 0.000301 | 0.005103 | Chr6 | 13315568 | 13329790 | 2838 |
| MsG0680030648.01 | -4.04456 | 0.000302 | 0.005115 | Chr6 | 6612783 | 6618921 | 1856 |
| MsG0280008835.01 | -1.37514 | 0.000302 | 0.005115 | Chr2 | 39785349 | 39797049 | 1953 |
| MsG0880042794.01 | -1.18522 | 0.000303 | 0.005125 | Chr8 | 14131262 | 14134901 | 984 |
| MsG0880045955.01 | -1.24846 | 0.000304 | 0.005146 | Chr8 | 66893202 | 66897395 | 985 |
| MsG0680033250.01 | -1.70817 | 0.000305 | 0.005153 | Chr6 | 60364611 | 60369787 | 1352 |
| novel.1306 | -5.51259 | 0.00031 | 0.005228 | Chr2 | 23572309 | 23573515 | 698 |
| novel.2345 | -2.37871 | 0.000311 | 0.005254 | Chr3 | 44817692 | 44819057 | 1366 |
| MsG0280009769.01 | 6.150057 | 0.000315 | 0.005306 | Chr2 | 57742884 | 57751339 | 3360 |
| novel.943 | 3.39302 | 0.000315 | 0.005307 | Chr1 | 66989595 | 66992940 | 1685 |
| MsG0180001676.01 | -2.54492 | 0.000316 | 0.005316 | Chr1 | 25012612 | 25016460 | 3437 |
| MsG0380017483.01 | -1.56442 | 0.000317 | 0.00533 | Chr3 | 96767428 | 96791445 | 3436 |
| novel.1404 | 1.557905 | 0.000318 | 0.005347 | Chr2 | 39711469 | 39714660 | 1400 |
| MsG0480022064.01 | 2.105869 | 0.000318 | 0.005355 | Chr4 | 67633738 | 67634238 | 501 |
| MsG0280011006.01 | 2.242433 | 0.000321 | 0.005394 | Chr2 | 78242355 | 78249665 | 2227 |
| novel.7627 | -4.26721 | 0.000324 | 0.00544 | Chr7 | 72945416 | 72947887 | 1140 |
| novel.8404 | 2.005307 | 0.000324 | 0.005445 | Chr8 | 19902113 | 19904350 | 2238 |
| MsG0280008254.01 | 1.78871 | 0.000325 | 0.005448 | Chr2 | 28055979 | 28060101 | 1230 |
| MsG0780039992.01 | 1.156172 | 0.000326 | 0.005459 | Chr7 | 71620496 | 71621982 | 558 |
| MsG0380013594.01 | 6.510803 | 0.000328 | 0.005491 | Chr3 | 41374246 | 41375087 | 750 |
| MsG0580025490.01 | -1.63088 | 0.000328 | 0.005492 | Chr5 | 19560728 | 19564828 | 2271 |
| MsG0180005219.01 | 2.358655 | 0.000329 | 0.005503 | Chr1 | 88716133 | 88724011 | 1586 |
| MsG0880045884.01 | 2.085751 | 0.000331 | 0.00554 | Chr8 | 65969500 | 65970861 | 1362 |
| novel.2518 | 2.657645 | 0.000332 | 0.005541 | Chr3 | 82318275 | 82326405 | 1357 |
| MsG0580027454.01 | -1.08517 | 0.000333 | 0.00556 | Chr5 | 61281683 | 61294389 | 4316 |
| MsG0880044022.01 | -2.41911 | 0.000336 | 0.005602 | Chr8 | 34463586 | 34469122 | 486 |
| MsG0180002050.01 | 6.564421 | 0.000338 | 0.00563 | Chr1 | 31678119 | 31684754 | 1425 |
| novel.8146 | -6.04876 | 0.000339 | 0.005644 | Chr8 | 66089381 | 66090562 | 702 |
| MsG0380015520.01 | -3.35312 | 0.00034 | 0.005662 | Chr3 | 71342435 | 71343403 | 969 |
| MsG0780036470.01 | -3.1475 | 0.00034 | 0.005662 | Chr7 | 8605384 | 8609979 | 677 |
| MsG0580029510.01 | 1.589549 | 0.00034 | 0.005662 | Chr5 | 97876007 | 97877038 | 1032 |
| MsG0780040697.01 | -2.23087 | 0.000341 | 0.005665 | Chr7 | 81312834 | 81314145 | 978 |
| MsG0880042143.01 | 1.419919 | 0.000341 | 0.005667 | Chr8 | 4246420 | 4249481 | 1063 |
| MsG0580025558.01 | -1.86118 | 0.000342 | 0.005684 | Chr5 | 20458921 | 20459277 | 357 |
| novel.1627 | -2.88644 | 0.000344 | 0.005712 | Chr2 | 82712022 | 82715261 | 1470 |
| novel.2439 | -2.01641 | 0.000348 | 0.005773 | Chr3 | 68580061 | 68582523 | 2463 |
| MsG0580029198.01 | -2.01643 | 0.000348 | 0.005773 | Chr5 | 93553037 | 93558750 | 998 |
| MsG0180004511.01 | 1.077609 | 0.000349 | 0.005773 | Chr1 | 78995327 | 78997893 | 714 |
| MsG0680032935.01 | 1.159558 | 0.000349 | 0.005773 | Chr6 | 53475811 | 53479435 | 1836 |
| novel.7557 | -6.1643 | 0.000349 | 0.005774 | Chr7 | 63172645 | 63184774 | 302 |
| MsG0380013999.01 | -4.90793 | 0.000349 | 0.005774 | Chr3 | 48616018 | 48616236 | 219 |
| novel.4360 | -3.181 | 0.00035 | 0.005779 | Chr5 | 12132912 | 12137267 | 4266 |
| novel.3428 | -6.01364 | 0.000353 | 0.005835 | Chr4 | 39894391 | 39894784 | 394 |
| MsG0880042411.01 | 1.782987 | 0.000354 | 0.005842 | Chr8 | 8367926 | 8371015 | 767 |
| MsG0080049149.01 | 2.170919 | 0.000355 | 0.005853 | contig95end | 352 | 753 | 402 |
| MsG0380012160.01 | -3.81984 | 0.000357 | 0.005879 | Chr3 | 11816489 | 11818455 | 742 |
| MsG0880045252.01 | 1.955082 | 0.000357 | 0.005887 | Chr8 | 56443320 | 56452568 | 1665 |
| novel.656 | -5.87308 | 0.000359 | 0.005907 | Chr1 | 7320848 | 7322418 | 475 |
| MsG0480021441.01 | 1.708703 | 0.000359 | 0.005908 | Chr4 | 57912814 | 57914547 | 1734 |
| MsG0080048248.01 | 1.671924 | 0.000362 | 0.005946 | contig253end | 16373 | 22691 | 1768 |
| novel.7425 | 6.193797 | 0.000362 | 0.005947 | Chr7 | 28276391 | 28278858 | 1298 |
| MsG0880047380.01 | 1.584517 | 0.000362 | 0.005947 | Chr8 | 85485655 | 85487315 | 372 |
| MsG0780036013.01 | -3.81432 | 0.000364 | 0.00597 | Chr7 | 1317267 | 1318012 | 680 |
| novel.5357 | -1.41621 | 0.000365 | 0.005981 | Chr5 | 88383595 | 88386625 | 1564 |
| MsG0380016773.01 | 2.074755 | 0.000365 | 0.005981 | Chr3 | 87768793 | 87771208 | 612 |
| MsG0180002394.01 | 2.256383 | 0.000368 | 0.006023 | Chr1 | 37926782 | 37927477 | 696 |
| MsG0480018517.01 | -3.44339 | 0.000368 | 0.006023 | Chr4 | 6051611 | 6051937 | 327 |
| MsG0880043648.01 | -2.3381 | 0.000371 | 0.006073 | Chr8 | 28422696 | 28424336 | 579 |
| novel.6239 | 5.160644 | 0.000372 | 0.006084 | Chr6 | 14488371 | 14489201 | 831 |
| MsG0480023066.01 | 3.652056 | 0.000372 | 0.006084 | Chr4 | 80361277 | 80365584 | 1197 |
| MsG0880046320.01 | 1.214191 | 0.000373 | 0.006091 | Chr8 | 71977202 | 71980284 | 1066 |
| novel.1298 | 3.935854 | 0.000375 | 0.006114 | Chr2 | 21617680 | 21619259 | 686 |
| MsG0480018569.01 | -3.75674 | 0.000375 | 0.006114 | Chr4 | 6766065 | 6766721 | 657 |
| MsG0580029353.01 | -2.93258 | 0.000375 | 0.006114 | Chr5 | 95892289 | 95898686 | 1059 |
| MsG0880042934.01 | -1.57021 | 0.000375 | 0.006114 | Chr8 | 16220822 | 16223894 | 1495 |
| novel.1751 | -2.03263 | 0.000375 | 0.006114 | Chr2 | 14830942 | 14835753 | 1961 |
| novel.3642 | 4.867877 | 0.000376 | 0.006117 | Chr4 | 79589694 | 79590569 | 566 |
| MsG0780038863.01 | -6.0533 | 0.000376 | 0.006124 | Chr7 | 54317833 | 54320994 | 480 |
| MsG0380016079.01 | 1.48018 | 0.000378 | 0.006147 | Chr3 | 78822740 | 78825912 | 1165 |
| novel.3275 | -6.1115 | 0.000379 | 0.00616 | Chr4 | 9275203 | 9276073 | 871 |
| novel.791 | 5.438917 | 0.000379 | 0.00616 | Chr1 | 30680816 | 30685461 | 1753 |
| MsG0480019305.01 | 1.300403 | 0.00038 | 0.006165 | Chr4 | 18219867 | 18227119 | 1135 |
| MsG0680032203.01 | -1.31955 | 0.000382 | 0.006192 | Chr6 | 36100420 | 36101812 | 609 |
| novel.8093 | 3.332611 | 0.000383 | 0.006211 | Chr8 | 58362357 | 58364051 | 784 |
| novel.2504 | 6.187153 | 0.000383 | 0.006211 | Chr3 | 78885567 | 78886623 | 870 |
| MsG0480021689.01 | 6.006854 | 0.000389 | 0.0063 | Chr4 | 62961504 | 62967839 | 4233 |
| novel.7787 | 6.136006 | 0.00039 | 0.006321 | Chr7 | 95291495 | 95292594 | 546 |
| MsG0280010414.01 | -1.17404 | 0.000391 | 0.006327 | Chr2 | 69187893 | 69198091 | 4629 |
| MsG0480019426.01 | 2.090289 | 0.000394 | 0.006368 | Chr4 | 20194784 | 20198941 | 1854 |
| MsG0380011889.01 | -2.21138 | 0.000395 | 0.006386 | Chr3 | 7463180 | 7470661 | 2486 |
| MsG0580027890.01 | -1.35298 | 0.000396 | 0.006393 | Chr5 | 70434010 | 70434546 | 537 |
| novel.757 | -3.70802 | 0.000397 | 0.006417 | Chr1 | 23121096 | 23122752 | 1181 |
| MsG0480021927.01 | 1.684392 | 0.000398 | 0.006421 | Chr4 | 66067234 | 66067605 | 240 |
| novel.2911 | 1.790708 | 0.0004 | 0.006444 | Chr3 | 50346795 | 50350821 | 1206 |
| MsG0880043287.01 | 2.526833 | 0.0004 | 0.006444 | Chr8 | 22000136 | 22000696 | 561 |
| novel.2694 | 6.213697 | 0.000402 | 0.006473 | Chr3 | 3682237 | 3684412 | 481 |
| MsG0180001887.01 | -2.43006 | 0.000402 | 0.006475 | Chr1 | 28804214 | 28806589 | 96 |
| MsG0380013549.01 | 1.581497 | 0.000404 | 0.006499 | Chr3 | 40520289 | 40524009 | 1467 |
| novel.2909 | -3.70218 | 0.000404 | 0.006505 | Chr3 | 50005163 | 50007033 | 500 |
| novel.5226 | -2.7262 | 0.000406 | 0.006522 | Chr5 | 65916174 | 65927589 | 9780 |
| MsG0480020499.01 | -1.95462 | 0.000407 | 0.006532 | Chr4 | 42499930 | 42502335 | 1428 |
| novel.845 | 3.150907 | 0.000408 | 0.006546 | Chr1 | 43137885 | 43140602 | 1094 |
| MsG0480021180.01 | 1.042333 | 0.000408 | 0.006552 | Chr4 | 54775887 | 54776114 | 228 |
| MsG0780036469.01 | -1.74413 | 0.000414 | 0.006647 | Chr7 | 8602475 | 8604905 | 1366 |
| MsG0380017169.01 | -2.57096 | 0.000415 | 0.006658 | Chr3 | 92929697 | 92936180 | 1041 |
| novel.9107 | -5.50778 | 0.000417 | 0.00669 | contig488end | 1994 | 3143 | 997 |
| MsG0880047584.01 | 1.014074 | 0.000418 | 0.006702 | Chr8 | 88328173 | 88330158 | 1234 |
| MsG0280010989.01 | 2.413882 | 0.00042 | 0.006715 | Chr2 | 78047794 | 78049557 | 1764 |
| MsG0880043854.01 | 2.5185 | 0.00042 | 0.006715 | Chr8 | 31635854 | 31653705 | 6168 |
| novel.4168 | 1.407772 | 0.000421 | 0.006723 | Chr4 | 79849489 | 79853179 | 894 |
| MsG0680035725.01 | 3.188336 | 0.000421 | 0.006723 | Chr6 | 1.11E+08 | 1.11E+08 | 3859 |
| MsG0480020009.01 | 3.180465 | 0.000422 | 0.006746 | Chr4 | 31870172 | 31870585 | 414 |
| MsG0180000574.01 | -5.23272 | 0.000424 | 0.006764 | Chr1 | 8023810 | 8024819 | 924 |
| MsG0580024822.01 | 2.139003 | 0.000424 | 0.006772 | Chr5 | 10296390 | 10298936 | 1148 |
| MsG0280007075.01 | -1.88616 | 0.000425 | 0.00678 | Chr2 | 10232027 | 10246564 | 1075 |
| novel.2339 | 3.186238 | 0.000425 | 0.00678 | Chr3 | 43568418 | 43570367 | 1950 |
| MsG0780040112.01 | 1.810957 | 0.000426 | 0.006783 | Chr7 | 73176017 | 73176823 | 807 |
| MsG0880047160.01 | -4.15043 | 0.000427 | 0.006804 | Chr8 | 82820819 | 82821814 | 426 |
| MsG0480021019.01 | 1.221534 | 0.000428 | 0.006806 | Chr4 | 52169556 | 52171763 | 2208 |
| novel.4799 | -4.85081 | 0.000428 | 0.006809 | Chr5 | 94927268 | 94929035 | 1671 |
| novel.192 | 6.103341 | 0.000428 | 0.00681 | Chr1 | 26676340 | 26677272 | 847 |
| MsG0180002184.01 | 1.361959 | 0.00043 | 0.006832 | Chr1 | 34394603 | 34405280 | 1700 |
| novel.8998 | -3.4686 | 0.000434 | 0.0069 | contig420end | 440 | 3398 | 806 |
| MsG0280008219.01 | -2.90874 | 0.000436 | 0.006916 | Chr2 | 27338507 | 27339216 | 471 |
| MsG0580028692.01 | -6.10049 | 0.000437 | 0.006941 | Chr5 | 84851726 | 84855633 | 2805 |
| MsG0480020305.01 | -6.11768 | 0.000438 | 0.006944 | Chr4 | 38580507 | 38589921 | 2424 |
| novel.3857 | -5.9763 | 0.000439 | 0.006963 | Chr4 | 20877432 | 20885723 | 371 |
| MsG0780036863.01 | -1.9692 | 0.000441 | 0.006987 | Chr7 | 15212445 | 15218389 | 989 |
| MsG0580025693.01 | 2.046318 | 0.000443 | 0.007008 | Chr5 | 22859926 | 22867064 | 1512 |
| MsG0280006581.01 | -1.94097 | 0.000443 | 0.007008 | Chr2 | 3675248 | 3679450 | 2769 |
| MsG0580026056.01 | -2.85643 | 0.000443 | 0.007014 | Chr5 | 29915687 | 29917239 | 1488 |
| MsG0580027410.01 | -2.38726 | 0.000444 | 0.007016 | Chr5 | 60021255 | 60022502 | 1248 |
| MsG0680031141.01 | 1.458246 | 0.000444 | 0.007016 | Chr6 | 15052112 | 15054226 | 1938 |
| MsG0580028612.01 | 6.258491 | 0.000444 | 0.007016 | Chr5 | 83397145 | 83397813 | 669 |
| novel.2926 | 1.037388 | 0.000446 | 0.007042 | Chr3 | 55611517 | 55615695 | 1758 |
| novel.8658 | 6.00247 | 0.000448 | 0.007061 | Chr8 | 70443817 | 70447180 | 1579 |
| MsG0680031027.01 | 4.818833 | 0.000448 | 0.007064 | Chr6 | 13009484 | 13009932 | 207 |
| novel.8056 | -1.88773 | 0.00045 | 0.007085 | Chr8 | 51181742 | 51183624 | 1663 |
| MsG0680035157.01 | -2.18103 | 0.00045 | 0.007091 | Chr6 | 99839350 | 99845740 | 2157 |
| novel.6552 | 5.97952 | 0.000451 | 0.007095 | Chr6 | 72401138 | 72403169 | 595 |
| MsG0180004332.01 | 4.239327 | 0.000452 | 0.007106 | Chr1 | 76485995 | 76490408 | 750 |
| MsG0280007936.01 | 1.134176 | 0.000452 | 0.007106 | Chr2 | 23268162 | 23270486 | 986 |
| novel.1715 | -5.67004 | 0.000456 | 0.007159 | Chr2 | 8474023 | 8479630 | 2205 |
| novel.5636 | -6.21276 | 0.000457 | 0.007174 | Chr6 | 19954950 | 19956790 | 521 |
| MsG0580027344.01 | -1.51108 | 0.000458 | 0.007182 | Chr5 | 58772222 | 58775375 | 391 |
| MsG0080047795.01 | 6.332984 | 0.000459 | 0.007194 | contig105end | 8525 | 12197 | 777 |
| MsG0180004311.01 | 1.084768 | 0.000459 | 0.007196 | Chr1 | 76343321 | 76344166 | 444 |
| MsG0680032370.01 | 6.117622 | 0.00046 | 0.007201 | Chr6 | 39408080 | 39411980 | 1770 |
| MsG0780041623.01 | 6.428193 | 0.000461 | 0.007225 | Chr7 | 93177983 | 93183662 | 738 |
| MsG0480019129.01 | -3.64427 | 0.000463 | 0.007244 | Chr4 | 14852580 | 14859627 | 978 |
| novel.4016 | -3.1936 | 0.000466 | 0.007298 | Chr4 | 57145563 | 57159964 | 3114 |
| MsG0480019311.01 | -2.91586 | 0.000468 | 0.007322 | Chr4 | 18337178 | 18337462 | 285 |
| novel.8859 | -6.18477 | 0.000469 | 0.007323 | contig219end | 13299 | 21607 | 607 |
| MsG0280007337.01 | 1.723492 | 0.000469 | 0.007323 | Chr2 | 14063160 | 14065961 | 747 |
| novel.6836 | -3.37971 | 0.000469 | 0.007328 | Chr7 | 12138679 | 12143503 | 3115 |
| novel.5766 | -6.32075 | 0.000471 | 0.00735 | Chr6 | 48981103 | 48981990 | 784 |
| MsG0780041758.01 | -5.12347 | 0.000471 | 0.007353 | Chr7 | 94792650 | 94793348 | 699 |
| MsG0280006674.01 | 2.030688 | 0.000472 | 0.007353 | Chr2 | 4797565 | 4815878 | 2213 |
| novel.2051 | -6.20322 | 0.000473 | 0.007364 | Chr2 | 76361235 | 76362541 | 593 |
| MsG0180004295.01 | 1.596466 | 0.000474 | 0.007379 | Chr1 | 76195672 | 76196795 | 1017 |
| MsG0880046344.01 | 2.081728 | 0.000475 | 0.007388 | Chr8 | 72237185 | 72241852 | 2568 |
| MsG0680035721.01 | 2.170524 | 0.000475 | 0.007399 | Chr6 | 1.11E+08 | 1.11E+08 | 4389 |
| MsG0480021006.01 | -4.10736 | 0.000477 | 0.007417 | Chr4 | 51986332 | 51990301 | 586 |
| MsG0480020962.01 | -2.42475 | 0.000478 | 0.007438 | Chr4 | 51258251 | 51262087 | 1648 |
| MsG0680032308.01 | 2.002946 | 0.000479 | 0.007449 | Chr6 | 38161558 | 38168177 | 1209 |
| novel.6673 | 6.482395 | 0.000481 | 0.007464 | Chr6 | 98985301 | 98987141 | 1478 |
| MsG0480023189.01 | -1.19976 | 0.000481 | 0.007465 | Chr4 | 81997757 | 82000541 | 929 |
| MsG0480020960.01 | 1.40692 | 0.000483 | 0.007497 | Chr4 | 51219271 | 51221507 | 1221 |
| MsG0580024168.01 | -4.18126 | 0.000484 | 0.007508 | Chr5 | 1522029 | 1523789 | 1079 |
| MsG0280009959.01 | 2.343167 | 0.000485 | 0.007515 | Chr2 | 61839368 | 61846374 | 1800 |
| novel.5003 | -1.35211 | 0.000485 | 0.007515 | Chr5 | 16502834 | 16506426 | 3593 |
| MsG0680034609.01 | -2.76651 | 0.000486 | 0.007522 | Chr6 | 91223520 | 91229442 | 887 |
| MsG0480019644.01 | 1.329158 | 0.000487 | 0.007522 | Chr4 | 23978165 | 23980914 | 699 |
| MsG0580027113.01 | 2.567731 | 0.000487 | 0.007522 | Chr5 | 53843926 | 53851407 | 1564 |
| MsG0280010347.01 | -1.18139 | 0.000487 | 0.007522 | Chr2 | 68397303 | 68400837 | 2180 |
| MsG0780036515.01 | -2.05015 | 0.000488 | 0.007533 | Chr7 | 9165412 | 9167939 | 1380 |
| novel.2112 | -5.95524 | 0.000488 | 0.007536 | Chr2 | 83751267 | 83753420 | 845 |
| novel.8399 | -3.54733 | 0.00049 | 0.007559 | Chr8 | 19070969 | 19073985 | 1431 |
| MsG0780039559.01 | -3.49944 | 0.000491 | 0.007572 | Chr7 | 65833832 | 65846288 | 1552 |
| novel.7144 | -6.27171 | 0.000491 | 0.007576 | Chr7 | 75603361 | 75603985 | 602 |
| novel.6071 | 5.379749 | 0.000492 | 0.007583 | Chr6 | 1.06E+08 | 1.06E+08 | 538 |
| MsG0580028101.01 | 1.239627 | 0.000492 | 0.007583 | Chr5 | 74650821 | 74652839 | 2019 |
| MsG0280009757.01 | 1.013039 | 0.000496 | 0.007639 | Chr2 | 57657566 | 57659278 | 1024 |
| MsG0680035010.01 | -6.7227 | 0.000497 | 0.007641 | Chr6 | 97741797 | 97748747 | 3244 |
| MsG0280009394.01 | 2.011893 | 0.000497 | 0.007641 | Chr2 | 51284797 | 51285042 | 246 |
| MsG0780037593.01 | -4.76943 | 0.000498 | 0.007647 | Chr7 | 29011631 | 29012380 | 750 |
| novel.8277 | 1.050759 | 0.000499 | 0.007664 | Chr8 | 88939229 | 88949568 | 4393 |
| novel.5337 | -3.93779 | 0.000503 | 0.007714 | Chr5 | 85221483 | 85230910 | 1113 |
| MsG0580025726.01 | 1.421606 | 0.000504 | 0.007731 | Chr5 | 23246586 | 23253440 | 966 |
| novel.4094 | 5.400563 | 0.000505 | 0.007736 | Chr4 | 68721018 | 68722486 | 908 |
| MsG0180000237.01 | -2.43647 | 0.000505 | 0.007737 | Chr1 | 3258864 | 3261228 | 1096 |
| MsG0780040173.01 | -2.1349 | 0.000508 | 0.007773 | Chr7 | 73914904 | 73921359 | 495 |
| MsG0680030756.01 | -1.55259 | 0.000508 | 0.007773 | Chr6 | 8682718 | 8683515 | 798 |
| MsG0880043736.01 | -5.94406 | 0.000508 | 0.007773 | Chr8 | 29833993 | 29840739 | 403 |
| MsG0280008332.01 | 2.53933 | 0.000508 | 0.007773 | Chr2 | 29465734 | 29473165 | 4338 |
| novel.8492 | 3.23837 | 0.000514 | 0.007855 | Chr8 | 38948238 | 39468553 | 1833 |
| MsG0880045324.01 | 1.442652 | 0.000518 | 0.007906 | Chr8 | 57508400 | 57511081 | 783 |
| MsG0380016589.01 | -1.2615 | 0.000519 | 0.007916 | Chr3 | 85408205 | 85412510 | 1373 |
| novel.6016 | 5.930206 | 0.00052 | 0.007932 | Chr6 | 99879565 | 99880664 | 603 |
| MsG0580027228.01 | 1.674382 | 0.000522 | 0.007958 | Chr5 | 56675776 | 56676870 | 1095 |
| MsG0780036064.01 | -1.57892 | 0.000523 | 0.007965 | Chr7 | 1997758 | 2005176 | 744 |
| MsG0480021756.01 | -1.49135 | 0.000524 | 0.007985 | Chr4 | 63916129 | 63921127 | 1778 |
| MsG0380016704.01 | -1.33992 | 0.000525 | 0.007996 | Chr3 | 86799061 | 86803535 | 1026 |
| MsG0480022025.01 | -1.88781 | 0.000525 | 0.007996 | Chr4 | 67135969 | 67138264 | 1914 |
| MsG0180004017.01 | 1.00544 | 0.000526 | 0.008004 | Chr1 | 71908810 | 71918749 | 1584 |
| MsG0280009953.01 | -1.37487 | 0.000527 | 0.008009 | Chr2 | 61652710 | 61681147 | 6066 |
| novel.1079 | -4.09236 | 0.000528 | 0.008017 | Chr1 | 91780053 | 91781150 | 1098 |
| MsG0380015663.01 | 4.131006 | 0.000529 | 0.008034 | Chr3 | 73280475 | 73280699 | 225 |
| MsG0580029893.01 | 1.360354 | 0.00053 | 0.008044 | Chr5 | 1.04E+08 | 1.04E+08 | 677 |
| MsG0280008403.01 | 6.008838 | 0.000532 | 0.008074 | Chr2 | 30720482 | 30721039 | 558 |
| MsG0580029430.01 | -1.84439 | 0.000533 | 0.008078 | Chr5 | 96898181 | 96902793 | 1628 |
| MsG0380014402.01 | -2.9779 | 0.000533 | 0.008082 | Chr3 | 54194716 | 54194940 | 225 |
| MsG0480021667.01 | 1.857886 | 0.000534 | 0.008093 | Chr4 | 62637087 | 62638010 | 679 |
| novel.3319 | -5.64974 | 0.000538 | 0.008141 | Chr4 | 16959240 | 16961015 | 828 |
| novel.73 | -5.96152 | 0.000538 | 0.008144 | Chr1 | 9769198 | 9776511 | 330 |
| novel.4683 | -5.57518 | 0.000538 | 0.008144 | Chr5 | 74611580 | 74616777 | 2193 |
| MsG0480020536.01 | -3.68648 | 0.000539 | 0.008144 | Chr4 | 43011700 | 43016354 | 239 |
| novel.8604 | -1.18678 | 0.000539 | 0.008144 | Chr8 | 60459324 | 60465739 | 2360 |
| MsG0880043013.01 | -5.41288 | 0.00054 | 0.008151 | Chr8 | 17675693 | 17681139 | 1992 |
| MsG0780036715.01 | -1.88244 | 0.000541 | 0.00817 | Chr7 | 12377576 | 12381018 | 2814 |
| novel.713 | -2.62428 | 0.000542 | 0.008171 | Chr1 | 15769421 | 15775810 | 1225 |
| novel.2467 | 5.970956 | 0.000542 | 0.008174 | Chr3 | 72844280 | 72845893 | 725 |
| MsG0880043252.01 | -2.69218 | 0.000545 | 0.008207 | Chr8 | 21289355 | 21290882 | 606 |
| MsG0380016357.01 | -2.31997 | 0.000545 | 0.008207 | Chr3 | 82427332 | 82428056 | 561 |
| MsG0280008954.01 | 2.293612 | 0.000548 | 0.008236 | Chr2 | 42216750 | 42218455 | 1147 |
| MsG0880047445.01 | -1.20028 | 0.000548 | 0.008236 | Chr8 | 86408610 | 86421750 | 3667 |
| MsG0180005089.01 | -6.07185 | 0.000551 | 0.008275 | Chr1 | 86807043 | 86811423 | 529 |
| novel.8269 | -6.01597 | 0.000551 | 0.008278 | Chr8 | 87980239 | 87981666 | 554 |
| novel.6165 | -3.81025 | 0.000554 | 0.008315 | Chr6 | 5018350 | 5023302 | 1431 |
| MsG0180001409.01 | -3.52677 | 0.000554 | 0.008315 | Chr1 | 20742297 | 20746119 | 3018 |
| MsG0880042932.01 | 1.211224 | 0.000555 | 0.008322 | Chr8 | 16202211 | 16203469 | 621 |
| MsG0880043170.01 | 1.263978 | 0.000556 | 0.008322 | Chr8 | 20054948 | 20056824 | 321 |
| MsG0380011504.01 | 1.419509 | 0.000556 | 0.008322 | Chr3 | 611425 | 617141 | 2597 |
| MsG0680032923.01 | 2.902064 | 0.000556 | 0.008322 | Chr6 | 53078362 | 53080279 | 883 |
| MsG0580028424.01 | 1.440681 | 0.000556 | 0.008322 | Chr5 | 80333574 | 80344226 | 720 |
| MsG0680034419.01 | -1.34871 | 0.000558 | 0.008344 | Chr6 | 87323096 | 87332203 | 738 |
| MsG0680031143.01 | 4.019609 | 0.000561 | 0.00839 | Chr6 | 15060355 | 15067464 | 1410 |
| MsG0880041862.01 | -2.87782 | 0.000562 | 0.008392 | Chr8 | 387614 | 389143 | 804 |
| MsG0180003191.01 | 3.764986 | 0.000564 | 0.008414 | Chr1 | 58148403 | 58151774 | 1788 |
| novel.2894 | -2.65663 | 0.000565 | 0.008436 | Chr3 | 46936888 | 46938742 | 1582 |
| novel.1362 | -2.75702 | 0.000566 | 0.008438 | Chr2 | 32353828 | 32354441 | 614 |
| novel.760 | -3.30142 | 0.000567 | 0.008448 | Chr1 | 24670404 | 24671407 | 915 |
| MsG0480018302.01 | 1.588036 | 0.000569 | 0.008482 | Chr4 | 3124061 | 3129474 | 1858 |
| MsG0280009517.01 | -1.26678 | 0.000572 | 0.008515 | Chr2 | 53471117 | 53473972 | 1077 |
| novel.8310 | -3.2559 | 0.000575 | 0.008548 | Chr8 | 2405227 | 2406932 | 425 |
| MsG0880042801.01 | 1.788591 | 0.000577 | 0.008573 | Chr8 | 14230880 | 14231379 | 390 |
| MsG0780039370.01 | 2.071323 | 0.000577 | 0.008573 | Chr7 | 62921927 | 62923273 | 612 |
| MsG0580026875.01 | 5.410453 | 0.000577 | 0.008576 | Chr5 | 47056583 | 47058918 | 918 |
| MsG0180000759.01 | -1.79991 | 0.000578 | 0.008577 | Chr1 | 10732849 | 10733460 | 612 |
| MsG0680035360.01 | 2.185909 | 0.000578 | 0.008583 | Chr6 | 1.03E+08 | 1.03E+08 | 2213 |
| MsG0780037642.01 | 2.456453 | 0.000579 | 0.008588 | Chr7 | 29947213 | 29949363 | 792 |
| MsG0480019197.01 | -2.64379 | 0.000581 | 0.008605 | Chr4 | 15943442 | 15946045 | 2604 |
| MsG0380013908.01 | -1.31765 | 0.000581 | 0.008605 | Chr3 | 48138656 | 48140098 | 1443 |
| novel.2074 | 3.771335 | 0.000581 | 0.008612 | Chr2 | 79852140 | 79852875 | 649 |
| novel.5354 | -2.71395 | 0.000582 | 0.008613 | Chr5 | 87706770 | 87709794 | 996 |
| MsG0380014702.01 | -1.11279 | 0.000583 | 0.008627 | Chr3 | 58666322 | 58669976 | 1913 |
| novel.7 | 1.285156 | 0.000584 | 0.008634 | Chr1 | 891971 | 893448 | 1039 |
| MsG0580027613.01 | -1.2495 | 0.000584 | 0.008639 | Chr5 | 64324616 | 64337404 | 2570 |
| MsG0380014457.01 | 1.369396 | 0.000587 | 0.008671 | Chr3 | 55077302 | 55083479 | 1653 |
| MsG0880042677.01 | -2.35006 | 0.000587 | 0.008676 | Chr8 | 12477623 | 12478321 | 699 |
| MsG0180005366.01 | 1.674911 | 0.000589 | 0.008695 | Chr1 | 90873534 | 90875318 | 748 |
| novel.6572 | -6.14796 | 0.00059 | 0.008702 | Chr6 | 75603751 | 75611272 | 1315 |
| MsG0580029325.01 | -1.44224 | 0.000591 | 0.008716 | Chr5 | 95286929 | 95287571 | 522 |
| novel.6852 | -3.46503 | 0.000593 | 0.008739 | Chr7 | 15255973 | 15258991 | 3019 |
| MsG0780036630.01 | -1.22045 | 0.000599 | 0.008831 | Chr7 | 10943509 | 10949453 | 3195 |
| MsG0580030111.01 | -4.82474 | 0.0006 | 0.008838 | Chr5 | 1.07E+08 | 1.07E+08 | 1218 |
| novel.4836 | -4.30836 | 0.000603 | 0.008871 | Chr5 | 99812347 | 99812868 | 522 |
| MsG0680032896.01 | 2.017415 | 0.000604 | 0.00889 | Chr6 | 52339312 | 52340440 | 543 |
| MsG0280010654.01 | -2.73622 | 0.000605 | 0.008898 | Chr2 | 73004171 | 73005586 | 1416 |
| MsG0380015432.01 | -3.30739 | 0.000606 | 0.008909 | Chr3 | 70174088 | 70179455 | 1546 |
| MsG0380013735.01 | -1.58452 | 0.000616 | 0.009044 | Chr3 | 44784850 | 44793817 | 418 |
| MsG0580029926.01 | -2.44391 | 0.000616 | 0.009044 | Chr5 | 1.04E+08 | 1.04E+08 | 1071 |
| MsG0380013739.01 | -1.0179 | 0.000616 | 0.009044 | Chr3 | 44872531 | 44873637 | 1107 |
| MsG0480024029.01 | -5.3451 | 0.000617 | 0.009053 | Chr4 | 92706564 | 92706994 | 348 |
| MsG0880043806.01 | -1.53196 | 0.000618 | 0.009063 | Chr8 | 30983698 | 30986289 | 1380 |
| MsG0880045100.01 | 1.736429 | 0.000618 | 0.009063 | Chr8 | 54202258 | 54212318 | 2898 |
| MsG0380012525.01 | 2.061129 | 0.000623 | 0.009134 | Chr3 | 19166357 | 19172423 | 903 |
| MsG0680031056.01 | -1.47696 | 0.000626 | 0.009162 | Chr6 | 13404501 | 13409499 | 1892 |
| MsG0680031254.01 | 2.835385 | 0.000627 | 0.009172 | Chr6 | 16866292 | 16867329 | 1038 |
| MsG0380016833.01 | -1.92846 | 0.000627 | 0.009174 | Chr3 | 88444970 | 88447978 | 1545 |
| MsG0780041383.01 | -2.59765 | 0.00063 | 0.009208 | Chr7 | 90322449 | 90329031 | 2417 |
| MsG0580024218.01 | -2.78408 | 0.00063 | 0.009208 | Chr5 | 2153360 | 2153967 | 399 |
| MsG0680031253.01 | -2.37708 | 0.000632 | 0.009225 | Chr6 | 16848781 | 16857722 | 1431 |
| novel.5783 | -4.7095 | 0.000636 | 0.009275 | Chr6 | 51842454 | 51843256 | 777 |
| novel.6005 | -1.91569 | 0.000636 | 0.009278 | Chr6 | 98403205 | 98406757 | 3553 |
| MsG0580029772.01 | 2.093555 | 0.000638 | 0.009308 | Chr5 | 1.02E+08 | 1.02E+08 | 627 |
| novel.3837 | -4.53527 | 0.000643 | 0.009378 | Chr4 | 17682562 | 17684465 | 1313 |
| MsG0480023675.01 | 1.943029 | 0.000644 | 0.00938 | Chr4 | 88444318 | 88446238 | 1692 |
| MsG0880047441.01 | -3.31564 | 0.000648 | 0.00943 | Chr8 | 86334973 | 86337760 | 1320 |
| MsG0480021254.01 | -2.1601 | 0.000649 | 0.009437 | Chr4 | 55940798 | 55954485 | 1584 |
| novel.6765 | -2.84842 | 0.000649 | 0.009437 | Chr6 | 1.1E+08 | 1.1E+08 | 1294 |
| MsG0880045912.01 | 4.003113 | 0.00065 | 0.009453 | Chr8 | 66309143 | 66315889 | 1304 |
| MsG0480018155.01 | -4.16927 | 0.000656 | 0.009526 | Chr4 | 961870 | 968360 | 1800 |
| MsG0780040231.01 | 2.680255 | 0.000659 | 0.009566 | Chr7 | 74674846 | 74675256 | 411 |
| MsG0580025069.01 | -3.78024 | 0.00066 | 0.009582 | Chr5 | 13520616 | 13521094 | 307 |
| MsG0880046300.01 | 1.889242 | 0.000663 | 0.009608 | Chr8 | 71662096 | 71662992 | 897 |
| novel.4253 | 5.985114 | 0.000663 | 0.009608 | Chr4 | 90310826 | 90311673 | 766 |
| MsG0480020869.01 | -1.13873 | 0.000667 | 0.009665 | Chr4 | 49880956 | 49887631 | 2480 |
| novel.5559 | -4.15215 | 0.000671 | 0.009708 | Chr6 | 6861808 | 6863719 | 1821 |
| novel.2111 | 1.225592 | 0.000674 | 0.009755 | Chr2 | 83648260 | 83649190 | 805 |
| MsG0680031781.01 | 5.954146 | 0.000675 | 0.009755 | Chr6 | 26603225 | 26614193 | 1170 |
| MsG0780039323.01 | -4.00503 | 0.000678 | 0.009794 | Chr7 | 62049428 | 62051405 | 1536 |
| MsG0280007997.01 | 2.955098 | 0.000683 | 0.009866 | Chr2 | 24107094 | 24107879 | 786 |
| novel.6786 | 1.496427 | 0.000684 | 0.009872 | Chr6 | 1.14E+08 | 1.14E+08 | 800 |
| MsG0880046668.01 | -3.30522 | 0.000684 | 0.009872 | Chr8 | 76422207 | 76423535 | 1329 |
| MsG0380015170.01 | -5.34169 | 0.000686 | 0.00989 | Chr3 | 66174370 | 66178168 | 1146 |
| novel.595 | -2.59114 | 0.000687 | 0.009892 | Chr1 | 1531724 | 1536706 | 3189 |
| MsG0780035991.01 | 1.040655 | 0.000688 | 0.009905 | Chr7 | 1025690 | 1040948 | 2440 |
| novel.2907 | 5.293958 | 0.000689 | 0.009917 | Chr3 | 49428223 | 49433779 | 661 |
| novel.2715 | 6.05933 | 0.000691 | 0.009942 | Chr3 | 7802197 | 7803749 | 537 |
| MsG0480022310.01 | -1.24584 | 0.000691 | 0.009942 | Chr4 | 70997123 | 70998819 | 1385 |
| MsG0280007323.01 | 1.29261 | 0.000697 | 0.010004 | Chr2 | 13605187 | 13605774 | 588 |
| MsG0280006437.01 | -5.43477 | 0.000697 | 0.010006 | Chr2 | 1939478 | 1943318 | 1305 |
| novel.8595 | -4.15037 | 0.000701 | 0.010055 | Chr8 | 57693514 | 57698111 | 1797 |
| MsG0780039338.01 | -4.41047 | 0.000701 | 0.010057 | Chr7 | 62196679 | 62198172 | 1194 |
| novel.664 | 1.134968 | 0.000702 | 0.010067 | Chr1 | 8878191 | 8886212 | 1104 |
| MsG0580024628.01 | -1.15619 | 0.000704 | 0.010084 | Chr5 | 7392479 | 7396348 | 2326 |
| novel.133 | -6.60036 | 0.000705 | 0.010097 | Chr1 | 17370037 | 17371290 | 495 |
| MsG0180000246.01 | 1.404909 | 0.000706 | 0.010099 | Chr1 | 3352425 | 3353012 | 588 |
| MsG0280008456.01 | 2.063142 | 0.000706 | 0.010099 | Chr2 | 32201557 | 32206394 | 3175 |
| MsG0380016148.01 | 1.136371 | 0.000707 | 0.010107 | Chr3 | 79636708 | 79644794 | 978 |
| MsG0180006148.01 | -2.68773 | 0.000707 | 0.010107 | Chr1 | 1.01E+08 | 1.01E+08 | 930 |
| novel.8560 | -5.97132 | 0.000708 | 0.010108 | Chr8 | 52612648 | 52613155 | 441 |
| MsG0780039513.01 | -3.5966 | 0.000708 | 0.010108 | Chr7 | 65073261 | 65081334 | 1785 |
| MsG0680034881.01 | -1.40409 | 0.000708 | 0.010108 | Chr6 | 96190137 | 96190994 | 858 |
| MsG0680031140.01 | 1.779517 | 0.000712 | 0.010155 | Chr6 | 15039916 | 15044324 | 1788 |
| novel.2780 | -4.5373 | 0.000712 | 0.010155 | Chr3 | 19137122 | 19147573 | 4727 |
| MsG0380016232.01 | -1.1061 | 0.000715 | 0.010186 | Chr3 | 80750095 | 80754282 | 1239 |
| MsG0280011404.01 | -1.15624 | 0.000715 | 0.010186 | Chr2 | 83512254 | 83514701 | 2448 |
| novel.6664 | -4.17705 | 0.000722 | 0.010277 | Chr6 | 97474437 | 97477989 | 879 |
| novel.3671 | -4.9767 | 0.000723 | 0.010277 | Chr4 | 83456833 | 83458212 | 1175 |
| novel.1629 | -2.89548 | 0.000724 | 0.010288 | Chr2 | 83042486 | 83045551 | 1233 |
| MsG0880043638.01 | 1.04297 | 0.000725 | 0.010294 | Chr8 | 28224995 | 28233753 | 3029 |
| novel.8011 | -4.75655 | 0.000726 | 0.010311 | Chr8 | 40737004 | 40738228 | 1225 |
| MsG0580028352.01 | -1.63604 | 0.000728 | 0.010336 | Chr5 | 78933146 | 78939445 | 1719 |
| MsG0680034721.01 | -1.40868 | 0.00073 | 0.01035 | Chr6 | 93410786 | 93423410 | 2818 |
| novel.1657 | -2.29263 | 0.000731 | 0.010365 | Chr2 | 1855553 | 1860595 | 2808 |
| MsG0880045054.01 | -1.68591 | 0.000733 | 0.010391 | Chr8 | 53486579 | 53492738 | 2369 |
| novel.6249 | -3.25454 | 0.000737 | 0.010427 | Chr6 | 16293193 | 16296964 | 3772 |
| MsG0680032165.01 | 1.529803 | 0.000739 | 0.010462 | Chr6 | 35414233 | 35441967 | 3332 |
| MsG0780039272.01 | 1.027345 | 0.00074 | 0.010472 | Chr7 | 61113430 | 61120078 | 1231 |
| MsG0280006430.01 | 2.490384 | 0.000742 | 0.010491 | Chr2 | 1853264 | 1853479 | 216 |
| novel.5208 | 6.038668 | 0.000745 | 0.010523 | Chr5 | 60878651 | 60880998 | 707 |
| MsG0680035325.01 | -1.78411 | 0.000747 | 0.010547 | Chr6 | 1.02E+08 | 1.02E+08 | 2388 |
| novel.8529 | 4.326619 | 0.000747 | 0.01055 | Chr8 | 47909633 | 47911514 | 1565 |
| MsG0080048106.01 | 1.509818 | 0.000751 | 0.010598 | contig20end | 4930 | 9646 | 3239 |
| novel.7882 | -3.49639 | 0.000753 | 0.010612 | Chr8 | 17008204 | 17009469 | 1027 |
| novel.3792 | -5.53718 | 0.000753 | 0.010612 | Chr4 | 6371977 | 6374026 | 1847 |
| MsG0380012960.01 | -1.46476 | 0.000753 | 0.010612 | Chr3 | 27725035 | 27728766 | 3732 |
| novel.2211 | -3.17842 | 0.000756 | 0.010648 | Chr3 | 14711974 | 14716994 | 2831 |
| novel.6066 | -5.75599 | 0.000757 | 0.010661 | Chr6 | 1.05E+08 | 1.05E+08 | 843 |
| MsG0280008093.01 | -5.34698 | 0.000759 | 0.010674 | Chr2 | 25400971 | 25401528 | 558 |
| MsG0680035570.01 | -2.42118 | 0.000759 | 0.010679 | Chr6 | 1.09E+08 | 1.09E+08 | 345 |
| MsG0780039420.01 | -4.82755 | 0.00076 | 0.010688 | Chr7 | 63828254 | 63832790 | 1221 |
| MsG0480018922.01 | 2.204062 | 0.000763 | 0.010715 | Chr4 | 11793374 | 11794719 | 399 |
| MsG0380014453.01 | -4.97408 | 0.000764 | 0.010725 | Chr3 | 55039137 | 55039673 | 537 |
| MsG0780039592.01 | -4.98889 | 0.000765 | 0.010736 | Chr7 | 66415731 | 66418983 | 1572 |
| novel.3426 | 5.921837 | 0.000767 | 0.010758 | Chr4 | 39264317 | 39265148 | 507 |
| MsG0480018593.01 | -3.41668 | 0.00077 | 0.010806 | Chr4 | 7086196 | 7094776 | 2811 |
| novel.848 | 4.800687 | 0.000773 | 0.010825 | Chr1 | 43723536 | 43727425 | 1033 |
| novel.762 | -2.17917 | 0.000776 | 0.010873 | Chr1 | 24806669 | 24812030 | 2001 |
| MsG0380016078.01 | 1.169311 | 0.000779 | 0.010897 | Chr3 | 78817955 | 78818786 | 585 |
| MsG0880042389.01 | 2.142873 | 0.000784 | 0.010972 | Chr8 | 7982079 | 7982648 | 570 |
| novel.9027 | -2.46719 | 0.000785 | 0.010972 | contig445end | 1 | 4684 | 4367 |
| novel.1762 | 3.690259 | 0.000787 | 0.010991 | Chr2 | 16705662 | 16707172 | 1284 |
| MsG0480022702.01 | -2.56697 | 0.000792 | 0.011059 | Chr4 | 75625903 | 75627846 | 1068 |
| novel.7566 | -3.10287 | 0.000793 | 0.011059 | Chr7 | 64531919 | 64537052 | 2211 |
| MsG0180001690.01 | -1.64819 | 0.000793 | 0.011068 | Chr1 | 25362968 | 25363180 | 213 |
| novel.8284 | -6.24631 | 0.000795 | 0.011083 | Chr8 | 89777714 | 89778766 | 971 |
| novel.4821 | -5.3896 | 0.000797 | 0.011112 | Chr5 | 98132236 | 98151461 | 1592 |
| MsG0480018311.01 | -1.59146 | 0.000798 | 0.01112 | Chr4 | 3192071 | 3193499 | 411 |
| MsG0480021865.01 | 6.281804 | 0.000799 | 0.01113 | Chr4 | 65339991 | 65340380 | 390 |
| novel.3760 | -2.93487 | 0.000801 | 0.011144 | Chr4 | 1794102 | 1795174 | 955 |
| MsG0380014624.01 | -1.05339 | 0.000806 | 0.011211 | Chr3 | 57443700 | 57448330 | 1706 |
| novel.8405 | 1.071298 | 0.000809 | 0.011231 | Chr8 | 20071955 | 20073714 | 965 |
| MsG0580028947.01 | 6.214062 | 0.000809 | 0.011231 | Chr5 | 89390100 | 89393839 | 1581 |
| MsG0380018009.01 | -1.31025 | 0.000809 | 0.011231 | Chr3 | 1.03E+08 | 1.03E+08 | 465 |
| MsG0380014144.01 | 1.527716 | 0.000811 | 0.011249 | Chr3 | 50332952 | 50336084 | 2265 |
| MsG0680034952.01 | -5.3789 | 0.000811 | 0.011249 | Chr6 | 97043881 | 97046067 | 1377 |
| novel.4917 | 1.894815 | 0.000812 | 0.011249 | Chr5 | 1604849 | 1606614 | 1766 |
| MsG0180003949.01 | -3.38708 | 0.000812 | 0.011249 | Chr1 | 70778392 | 70785753 | 578 |
| MsG0380015277.01 | 1.864224 | 0.000812 | 0.011253 | Chr3 | 67773308 | 67777916 | 750 |
| MsG0180001278.01 | 5.924323 | 0.000818 | 0.011321 | Chr1 | 18688045 | 18688767 | 723 |
| MsG0680031676.01 | 2.682373 | 0.000818 | 0.011324 | Chr6 | 24489893 | 24495706 | 3403 |
| novel.4945 | 4.704909 | 0.000819 | 0.011325 | Chr5 | 6326444 | 6327784 | 412 |
| novel.3335 | 3.120171 | 0.00082 | 0.011325 | Chr4 | 20403874 | 20407988 | 1765 |
| novel.7621 | -6.23192 | 0.00082 | 0.011325 | Chr7 | 71748017 | 71749752 | 667 |
| novel.118 | -1.47534 | 0.00082 | 0.01133 | Chr1 | 15333418 | 15336559 | 2227 |
| MsG0880043658.01 | -4.45404 | 0.000821 | 0.011338 | Chr8 | 28574751 | 28577779 | 804 |
| MsG0780039079.01 | -5.97552 | 0.000822 | 0.011339 | Chr7 | 57594012 | 57595849 | 974 |
| novel.7421 | -6.07051 | 0.000826 | 0.011397 | Chr7 | 27104734 | 27107195 | 1941 |
| MsG0780041047.01 | -1.64242 | 0.000827 | 0.011397 | Chr7 | 85837987 | 85839768 | 1782 |
| MsG0680031998.01 | 1.293754 | 0.000829 | 0.011428 | Chr6 | 31654134 | 31663760 | 3450 |
| MsG0880047128.01 | -2.69845 | 0.000831 | 0.011444 | Chr8 | 82371157 | 82377524 | 2961 |
| MsG0680034243.01 | 4.677102 | 0.000832 | 0.011455 | Chr6 | 84292905 | 84308288 | 3534 |
| MsG0680032013.01 | 1.199827 | 0.000833 | 0.011455 | Chr6 | 31934476 | 31937578 | 988 |
| MsG0080048998.01 | 2.6639 | 0.000834 | 0.011471 | contig5end | 479 | 882 | 404 |
| MsG0180000389.01 | 2.019576 | 0.000835 | 0.011474 | Chr1 | 5334548 | 5336373 | 932 |
| MsG0680030399.01 | 2.452711 | 0.000836 | 0.011478 | Chr6 | 1679383 | 1689712 | 1419 |
| MsG0380012013.01 | -2.75286 | 0.000837 | 0.01149 | Chr3 | 9499412 | 9501154 | 1210 |
| MsG0880043548.01 | -1.99892 | 0.000838 | 0.011496 | Chr8 | 26438358 | 26442136 | 986 |
| MsG0180002918.01 | -2.46807 | 0.00084 | 0.011515 | Chr1 | 52956123 | 52957496 | 1374 |
| novel.959 | -1.35003 | 0.000841 | 0.011525 | Chr1 | 72047598 | 72053760 | 1599 |
| novel.3228 | -6.38315 | 0.000843 | 0.011545 | Chr4 | 2610208 | 2614016 | 1766 |
| MsG0480020896.01 | -1.3786 | 0.000843 | 0.011545 | Chr4 | 50315600 | 50317317 | 624 |
| novel.6039 | -1.29203 | 0.000845 | 0.01156 | Chr6 | 1.03E+08 | 1.03E+08 | 2263 |
| MsG0580029385.01 | 1.141258 | 0.000847 | 0.011585 | Chr5 | 96244333 | 96244713 | 381 |
| MsG0580029412.01 | 1.317028 | 0.000848 | 0.011589 | Chr5 | 96680831 | 96697732 | 3876 |
| MsG0580026706.01 | 6.030351 | 0.000848 | 0.011589 | Chr5 | 42660460 | 42662033 | 1173 |
| MsG0180001126.01 | -1.07786 | 0.00085 | 0.011607 | Chr1 | 16325713 | 16330663 | 2475 |
| novel.9176 | -1.78069 | 0.000851 | 0.01162 | contig530end | 5634 | 14244 | 2831 |
| novel.4898 | 1.792143 | 0.000852 | 0.011628 | Chr5 | 1.08E+08 | 1.08E+08 | 2070 |
| novel.911 | 1.866652 | 0.000854 | 0.011652 | Chr1 | 61395943 | 61397965 | 741 |
| novel.555 | -1.14026 | 0.000857 | 0.011688 | Chr1 | 98178857 | 98180412 | 1556 |
| MsG0880043587.01 | -5.84087 | 0.00086 | 0.011716 | Chr8 | 27344346 | 27345878 | 750 |
| MsG0580030029.01 | 1.868719 | 0.000863 | 0.011743 | Chr5 | 1.05E+08 | 1.05E+08 | 900 |
| novel.1994 | -2.30625 | 0.000863 | 0.011749 | Chr2 | 69202847 | 69205497 | 2089 |
| MsG0380017151.01 | -1.21057 | 0.000869 | 0.011823 | Chr3 | 92673448 | 92677584 | 1848 |
| novel.3365 | -4.09323 | 0.00087 | 0.01183 | Chr4 | 25746820 | 25747816 | 916 |
| MsG0480022568.01 | 1.141973 | 0.000872 | 0.011851 | Chr4 | 74199143 | 74202159 | 1055 |
| MsG0480020690.01 | -1.16133 | 0.000875 | 0.011876 | Chr4 | 46272778 | 46276119 | 1025 |
| novel.7617 | 1.807369 | 0.000877 | 0.011907 | Chr7 | 71340183 | 71343735 | 2638 |
| MsG0380012270.01 | -2.26863 | 0.000878 | 0.011912 | Chr3 | 13853094 | 13858952 | 2439 |
| MsG0880044003.01 | -1.93429 | 0.000889 | 0.012052 | Chr8 | 34206055 | 34211750 | 1068 |
| MsG0680035118.01 | -1.68784 | 0.000891 | 0.012067 | Chr6 | 99210841 | 99221885 | 5013 |
| novel.1100 | -5.36048 | 0.000901 | 0.012197 | Chr1 | 94424099 | 94424851 | 647 |
| MsG0880045275.01 | -1.76022 | 0.000901 | 0.012197 | Chr8 | 56762966 | 56767164 | 768 |
| MsG0580028091.01 | -6.20464 | 0.000904 | 0.012231 | Chr5 | 74448960 | 74450384 | 1425 |
| MsG0680030585.01 | -6.03464 | 0.000905 | 0.012244 | Chr6 | 5679958 | 5681397 | 1440 |
| MsG0380015985.01 | -2.04704 | 0.000907 | 0.012263 | Chr3 | 77530506 | 77533135 | 891 |
| MsG0180000650.01 | -2.22307 | 0.000908 | 0.012269 | Chr1 | 9217686 | 9217976 | 291 |
| MsG0480019434.01 | 2.247433 | 0.00091 | 0.012284 | Chr4 | 20289062 | 20296002 | 389 |
| MsG0580030164.01 | -2.35998 | 0.000911 | 0.012286 | Chr5 | 1.08E+08 | 1.08E+08 | 780 |
| MsG0780041692.01 | 2.083608 | 0.000912 | 0.012303 | Chr7 | 93991090 | 93994506 | 1137 |
| MsG0680030656.01 | -2.13777 | 0.000918 | 0.012374 | Chr6 | 6678544 | 6686445 | 3838 |
| novel.3491 | -5.78069 | 0.000918 | 0.012374 | Chr4 | 55587458 | 55589133 | 1002 |
| MsG0380015310.01 | 2.300368 | 0.000923 | 0.012436 | Chr3 | 68271075 | 68273027 | 1101 |
| MsG0680035362.01 | 2.187393 | 0.000926 | 0.012461 | Chr6 | 1.03E+08 | 1.03E+08 | 1779 |
| MsG0380011744.01 | -1.0744 | 0.000932 | 0.01254 | Chr3 | 4386171 | 4394862 | 1425 |
| novel.7736 | 1.193329 | 0.000933 | 0.012544 | Chr7 | 88874940 | 88876754 | 1815 |
| novel.8953 | -3.28752 | 0.000934 | 0.012553 | contig354end | 9026 | 17816 | 694 |
| novel.2625 | 1.263986 | 0.000936 | 0.012567 | Chr3 | 96254783 | 96260431 | 3002 |
| MsG0380017954.01 | 2.610541 | 0.000937 | 0.01257 | Chr3 | 1.03E+08 | 1.03E+08 | 660 |
| novel.5791 | -2.64995 | 0.000938 | 0.012581 | Chr6 | 52841424 | 52842552 | 1129 |
| novel.7860 | 5.860614 | 0.000941 | 0.012616 | Chr8 | 11598158 | 11599352 | 823 |
| novel.8393 | 1.574647 | 0.000941 | 0.01262 | Chr8 | 18009228 | 18011028 | 785 |
| novel.6557 | -5.83694 | 0.000948 | 0.012692 | Chr6 | 72666859 | 72670055 | 655 |
| novel.8112 | -6.0627 | 0.000948 | 0.012692 | Chr8 | 61490092 | 61490823 | 732 |
| novel.5776 | -2.13896 | 0.00095 | 0.012709 | Chr6 | 50655702 | 50656959 | 1258 |
| MsG0180001474.01 | -1.55291 | 0.000953 | 0.012742 | Chr1 | 21915994 | 21930539 | 2266 |
| novel.6707 | -3.07237 | 0.000959 | 0.012819 | Chr6 | 1.03E+08 | 1.03E+08 | 1686 |
| MsG0480023299.01 | -1.99477 | 0.000959 | 0.012819 | Chr4 | 83499012 | 83512408 | 2061 |
| novel.7006 | 1.441403 | 0.00096 | 0.012821 | Chr7 | 47910411 | 47914000 | 1629 |
| MsG0380013770.01 | 2.32313 | 0.000968 | 0.012919 | Chr3 | 45432874 | 45438022 | 2454 |
| novel.6063 | 6.014285 | 0.000971 | 0.012952 | Chr6 | 1.05E+08 | 1.05E+08 | 805 |
| MsG0380012034.01 | 1.994226 | 0.000974 | 0.012986 | Chr3 | 9869191 | 9872020 | 2679 |
| MsG0680033539.01 | 2.143786 | 0.000979 | 0.013037 | Chr6 | 67653980 | 67654522 | 543 |
| novel.4067 | 4.562218 | 0.000979 | 0.013037 | Chr4 | 65653540 | 65655815 | 557 |
| MsG0480022153.01 | 5.877572 | 0.000979 | 0.013037 | Chr4 | 69003312 | 69005774 | 1230 |
| MsG0280007915.01 | -1.03502 | 0.000981 | 0.013058 | Chr2 | 22856694 | 22858271 | 474 |
| novel.6679 | 3.404873 | 0.000982 | 0.013058 | Chr6 | 99850730 | 99851582 | 443 |
| MsG0380014761.01 | 2.452001 | 0.000999 | 0.013279 | Chr3 | 59546933 | 59548553 | 857 |
| MsG0480019427.01 | 2.950011 | 0.001001 | 0.013295 | Chr4 | 20202020 | 20203474 | 1455 |
| MsG0880042417.01 | 1.913442 | 0.001002 | 0.013295 | Chr8 | 8436015 | 8438474 | 2460 |
| novel.3215 | -2.77037 | 0.001002 | 0.013295 | Chr3 | 1.04E+08 | 1.04E+08 | 1495 |
| MsG0280007121.01 | -2.91925 | 0.001008 | 0.013367 | Chr2 | 10921036 | 10932177 | 1350 |
| MsG0880046359.01 | 1.020427 | 0.001011 | 0.013399 | Chr8 | 72393340 | 72394803 | 881 |
| novel.5726 | -2.70164 | 0.001014 | 0.013431 | Chr6 | 39147004 | 39147865 | 698 |
| MsG0380015769.01 | 5.867923 | 0.001016 | 0.013458 | Chr3 | 74844405 | 74845149 | 624 |
| novel.7229 | -2.73526 | 0.001025 | 0.013563 | Chr7 | 87394155 | 87395500 | 366 |
| MsG0380015615.01 | 2.195925 | 0.001027 | 0.013586 | Chr3 | 72548917 | 72549561 | 645 |
| MsG0480023127.01 | -1.14937 | 0.00103 | 0.013614 | Chr4 | 81177606 | 81180436 | 612 |
| novel.8840 | -4.41202 | 0.001035 | 0.013674 | contig175end | 11938 | 14860 | 1701 |
| MsG0680032274.01 | 2.27814 | 0.001036 | 0.013679 | Chr6 | 37677668 | 37684349 | 4278 |
| MsG0580025639.01 | -2.40097 | 0.001041 | 0.013739 | Chr5 | 21621397 | 21630476 | 2228 |
| novel.2786 | 2.647934 | 0.001042 | 0.013745 | Chr3 | 20686155 | 20690842 | 936 |
| novel.3124 | -6.0012 | 0.001046 | 0.013794 | Chr3 | 90571750 | 90573089 | 1121 |
| MsG0680031251.01 | -1.77648 | 0.001047 | 0.013799 | Chr6 | 16833374 | 16837513 | 2004 |
| MsG0680032306.01 | -1.5082 | 0.001048 | 0.013805 | Chr6 | 38152397 | 38154937 | 2541 |
| MsG0880047326.01 | 1.094557 | 0.001053 | 0.013866 | Chr8 | 84711062 | 84711632 | 348 |
| MsG0480022693.01 | 1.263823 | 0.001057 | 0.013912 | Chr4 | 75550630 | 75551428 | 488 |
| MsG0680034677.01 | -1.95239 | 0.001057 | 0.013912 | Chr6 | 92693198 | 92701426 | 1571 |
| novel.7547 | 1.676298 | 0.001062 | 0.013972 | Chr7 | 60713968 | 60715315 | 1348 |
| novel.1837 | 5.856307 | 0.001062 | 0.013972 | Chr2 | 31269684 | 31276887 | 618 |
| MsG0580028248.01 | -3.43248 | 0.001064 | 0.013992 | Chr5 | 77254395 | 77263196 | 1281 |
| novel.67 | 2.069086 | 0.001065 | 0.013993 | Chr1 | 8551927 | 8561007 | 1627 |
| MsG0480021066.01 | -6.04434 | 0.001074 | 0.0141 | Chr4 | 52954150 | 52954488 | 339 |
| MsG0380014948.01 | -1.05384 | 0.001076 | 0.014129 | Chr3 | 62913004 | 62917273 | 1794 |
| novel.8276 | -2.15621 | 0.001083 | 0.0142 | Chr8 | 88931458 | 88935073 | 1824 |
| MsG0880043788.01 | -1.11401 | 0.001083 | 0.0142 | Chr8 | 30689434 | 30694950 | 995 |
| MsG0080048405.01 | -1.28532 | 0.001084 | 0.0142 | contig304end | 6567 | 10240 | 1126 |
| novel.7965 | -2.15402 | 0.001084 | 0.014201 | Chr8 | 31772232 | 31781486 | 2151 |
| MsG0180001148.01 | -1.24559 | 0.001089 | 0.014253 | Chr1 | 16579972 | 16581603 | 1221 |
| MsG0580025609.01 | 1.552906 | 0.001095 | 0.014329 | Chr5 | 21208168 | 21209979 | 1089 |
| novel.189 | 2.440066 | 0.001097 | 0.014355 | Chr1 | 25949256 | 25960362 | 749 |
| MsG0680032065.01 | 3.043463 | 0.001101 | 0.014393 | Chr6 | 33004706 | 33011091 | 1296 |
| MsG0780038937.01 | -4.49551 | 0.001108 | 0.014475 | Chr7 | 55562214 | 55566368 | 1533 |
| MsG0780037371.01 | -2.1491 | 0.001108 | 0.014475 | Chr7 | 24502494 | 24506474 | 2460 |
| novel.4718 | 5.755354 | 0.001126 | 0.014702 | Chr5 | 80806211 | 80806763 | 459 |
| novel.5935 | -5.73879 | 0.001126 | 0.014705 | Chr6 | 85497269 | 85500548 | 357 |
| MsG0380014362.01 | 5.016866 | 0.001128 | 0.014718 | Chr3 | 53505681 | 53506772 | 504 |
| novel.5179 | -1.55136 | 0.00113 | 0.014732 | Chr5 | 55130019 | 55136188 | 4168 |
| MsG0180000932.01 | -5.53087 | 0.00113 | 0.014732 | Chr1 | 13379024 | 13379728 | 705 |
| MsG0680034479.01 | 3.502405 | 0.001131 | 0.014742 | Chr6 | 88295362 | 88296900 | 1539 |
| novel.7731 | 5.884828 | 0.001132 | 0.014743 | Chr7 | 88206767 | 88207218 | 355 |
| MsG0480021504.01 | 5.845867 | 0.001134 | 0.014755 | Chr4 | 58734045 | 58743199 | 1341 |
| MsG0380016349.01 | -1.11506 | 0.001139 | 0.014824 | Chr3 | 82296906 | 82300540 | 2038 |
| MsG0480019019.01 | -1.01078 | 0.00114 | 0.014829 | Chr4 | 13386088 | 13386821 | 619 |
| MsG0880047195.01 | -6.09459 | 0.001151 | 0.014961 | Chr8 | 83183787 | 83186030 | 1308 |
| MsG0480022905.01 | -1.14084 | 0.001152 | 0.014961 | Chr4 | 78338272 | 78343608 | 2332 |
| MsG0480018493.01 | -1.84883 | 0.001152 | 0.014961 | Chr4 | 5761135 | 5764130 | 1725 |
| MsG0380016571.01 | 1.459911 | 0.001154 | 0.014987 | Chr3 | 85259928 | 85260263 | 336 |
| MsG0880042914.01 | -2.06773 | 0.001158 | 0.01503 | Chr8 | 15834982 | 15835926 | 945 |
| MsG0680030964.01 | -1.08291 | 0.001159 | 0.015036 | Chr6 | 12242904 | 12243902 | 999 |
| MsG0780036024.01 | -2.10402 | 0.001162 | 0.015061 | Chr7 | 1462391 | 1468729 | 3531 |
| MsG0780040952.01 | 1.271784 | 0.001165 | 0.015098 | Chr7 | 84727359 | 84730666 | 1388 |
| novel.9363 | 1.211011 | 0.001168 | 0.015122 | contig649end | 2261 | 3618 | 826 |
| novel.2223 | 5.82087 | 0.00117 | 0.01514 | Chr3 | 16237307 | 16237623 | 317 |
| novel.6299 | 1.066747 | 0.00117 | 0.01514 | Chr6 | 22551934 | 22570667 | 4239 |
| MsG0080049021.01 | -2.1479 | 0.001179 | 0.01523 | contig614end | 31 | 2698 | 1254 |
| MsG0480018334.01 | -3.00986 | 0.001179 | 0.01523 | Chr4 | 3480006 | 3482300 | 888 |
| MsG0380011674.01 | -1.83196 | 0.001179 | 0.01523 | Chr3 | 3341789 | 3344886 | 1623 |
| novel.4462 | -6.13476 | 0.00118 | 0.015234 | Chr5 | 27216899 | 27218400 | 716 |
| MsG0180001977.01 | 1.528497 | 0.00118 | 0.015234 | Chr1 | 30150147 | 30158471 | 1749 |
| novel.9062 | -4.43495 | 0.001182 | 0.015251 | contig463end | 5573 | 8949 | 653 |
| novel.453 | 1.550324 | 0.001183 | 0.015251 | Chr1 | 82790964 | 82793018 | 2055 |
| novel.6850 | -1.75408 | 0.001184 | 0.015255 | Chr7 | 14765314 | 14770360 | 3912 |
| MsG0580024260.01 | -2.52159 | 0.001184 | 0.015255 | Chr5 | 2695386 | 2696365 | 927 |
| MsG0880046521.01 | 1.805795 | 0.001186 | 0.015278 | Chr8 | 74327029 | 74330873 | 1104 |
| MsG0480021125.01 | -2.41593 | 0.001192 | 0.015343 | Chr4 | 53884206 | 53896322 | 3054 |
| MsG0680033260.01 | 5.980707 | 0.001193 | 0.015348 | Chr6 | 60601364 | 60604798 | 1176 |
| MsG0480019337.01 | 1.951211 | 0.001193 | 0.015348 | Chr4 | 18629656 | 18629916 | 261 |
| novel.8450 | -6.30647 | 0.001198 | 0.015394 | Chr8 | 29140538 | 29142096 | 656 |
| MsG0180001967.01 | 1.388031 | 0.001199 | 0.0154 | Chr1 | 30004068 | 30009515 | 943 |
| novel.6074 | 1.578199 | 0.0012 | 0.0154 | Chr6 | 1.06E+08 | 1.06E+08 | 823 |
| novel.1840 | -2.5627 | 0.0012 | 0.0154 | Chr2 | 31558652 | 31561734 | 2455 |
| MsG0180001276.01 | -2.74686 | 0.001202 | 0.015417 | Chr1 | 18665572 | 18671020 | 375 |
| MsG0680031135.01 | -5.81504 | 0.001205 | 0.015447 | Chr6 | 15013286 | 15014125 | 714 |
| novel.7417 | -2.31804 | 0.001214 | 0.015563 | Chr7 | 25392033 | 25398698 | 4030 |
| novel.7438 | -3.27368 | 0.001215 | 0.015563 | Chr7 | 32517969 | 32518789 | 718 |
| MsG0480021336.01 | -5.23194 | 0.001237 | 0.015828 | Chr4 | 56866588 | 56866869 | 282 |
| MsG0880042862.01 | 2.211815 | 0.001237 | 0.015828 | Chr8 | 15023069 | 15025699 | 1545 |
| novel.5473 | -2.59781 | 0.001239 | 0.01585 | Chr5 | 1.05E+08 | 1.05E+08 | 1460 |
| novel.7813 | 5.770704 | 0.00124 | 0.015853 | Chr8 | 3468674 | 3470281 | 979 |
| novel.4477 | -1.50014 | 0.001244 | 0.015897 | Chr5 | 30087781 | 30090542 | 1544 |
| novel.847 | -5.75174 | 0.001244 | 0.015897 | Chr1 | 43446959 | 43448559 | 644 |
| MsG0880046149.01 | -1.61853 | 0.001245 | 0.015901 | Chr8 | 69864735 | 69865641 | 672 |
| MsG0780037052.01 | -2.20267 | 0.00125 | 0.015952 | Chr7 | 18600816 | 18605431 | 1701 |
| MsG0380017905.01 | 5.825179 | 0.001252 | 0.015973 | Chr3 | 1.02E+08 | 1.02E+08 | 1071 |
| MsG0880047188.01 | -1.66092 | 0.001253 | 0.015978 | Chr8 | 83105754 | 83106287 | 534 |
| MsG0180003697.01 | 1.0225 | 0.001258 | 0.016029 | Chr1 | 66822045 | 66826847 | 1230 |
| novel.1954 | -5.78581 | 0.001259 | 0.016029 | Chr2 | 61472943 | 61483474 | 266 |
| novel.5457 | -5.78581 | 0.001259 | 0.016029 | Chr5 | 1.03E+08 | 1.03E+08 | 511 |
| novel.7274 | -6.41152 | 0.001269 | 0.016147 | Chr7 | 93039931 | 93040707 | 777 |
| MsG0180002395.01 | 5.888293 | 0.001272 | 0.016182 | Chr1 | 37929256 | 37934127 | 1326 |
| novel.3826 | -8.56429 | 0.001273 | 0.016182 | Chr4 | 15621509 | 15625839 | 898 |
| MsG0180000255.01 | -2.43653 | 0.001276 | 0.016207 | Chr1 | 3434853 | 3435128 | 276 |
| MsG0880044360.01 | 1.001454 | 0.001276 | 0.016207 | Chr8 | 40550623 | 40561315 | 1539 |
| MsG0480022997.01 | 4.571968 | 0.001277 | 0.016215 | Chr4 | 79540238 | 79540830 | 483 |
| novel.5194 | -1.95571 | 0.001282 | 0.016265 | Chr5 | 58158027 | 58162587 | 3114 |
| MsG0180000733.01 | 1.555476 | 0.001284 | 0.016291 | Chr1 | 10396489 | 10399691 | 946 |
| MsG0480019325.01 | -1.88654 | 0.001285 | 0.016292 | Chr4 | 18518396 | 18519116 | 721 |
| MsG0180002212.01 | -3.74246 | 0.001287 | 0.016314 | Chr1 | 34942365 | 34945124 | 2760 |
| MsG0180004678.01 | -6.26888 | 0.001289 | 0.016324 | Chr1 | 81133253 | 81134866 | 633 |
| MsG0580025316.01 | -4.97062 | 0.001289 | 0.016324 | Chr5 | 16878547 | 16879923 | 1377 |
| MsG0680030857.01 | -1.56713 | 0.001295 | 0.016398 | Chr6 | 10570834 | 10574027 | 1789 |
| MsG0280011177.01 | -1.26392 | 0.001299 | 0.016441 | Chr2 | 80573948 | 80578200 | 1314 |
| MsG0480023211.01 | 1.132085 | 0.001301 | 0.016456 | Chr4 | 82210785 | 82215337 | 1297 |
| MsG0880046830.01 | -1.15921 | 0.001304 | 0.016487 | Chr8 | 78842424 | 78845252 | 1524 |
| MsG0680035160.01 | 1.935289 | 0.001304 | 0.016487 | Chr6 | 99863235 | 99871065 | 5208 |
| MsG0680030970.01 | -5.94216 | 0.00132 | 0.016674 | Chr6 | 12364432 | 12365358 | 504 |
| MsG0480018767.01 | -1.45597 | 0.001325 | 0.016724 | Chr4 | 9556357 | 9559243 | 1780 |
| MsG0580024829.01 | 1.854055 | 0.001325 | 0.016724 | Chr5 | 10433229 | 10433678 | 450 |
| MsG0380015488.01 | 1.87839 | 0.001326 | 0.016724 | Chr3 | 70926319 | 70927869 | 948 |
| MsG0480021561.01 | 1.572522 | 0.001326 | 0.016724 | Chr4 | 59844999 | 59846597 | 940 |
| novel.158 | -1.53701 | 0.001328 | 0.016737 | Chr1 | 22178352 | 22180915 | 2511 |
| MsG0380014710.01 | 3.544386 | 0.001329 | 0.016745 | Chr3 | 58777865 | 58781970 | 2026 |
| novel.6120 | -6.89785 | 0.001337 | 0.016832 | Chr6 | 1.13E+08 | 1.13E+08 | 1208 |
| novel.8543 | 2.072393 | 0.001339 | 0.016855 | Chr8 | 50929724 | 50932374 | 2371 |
| MsG0380014461.01 | 5.818193 | 0.001343 | 0.016894 | Chr3 | 55143035 | 55143604 | 570 |
| MsG0880042278.01 | -1.7861 | 0.00135 | 0.016975 | Chr8 | 6456971 | 6458401 | 1431 |
| novel.6162 | 5.77345 | 0.001351 | 0.016981 | Chr6 | 4870984 | 4872038 | 622 |
| MsG0580025247.01 | -1.75085 | 0.00136 | 0.017074 | Chr5 | 15805385 | 15812661 | 975 |
| novel.7362 | 5.768418 | 0.001361 | 0.017074 | Chr7 | 15447805 | 15450652 | 1775 |
| MsG0180002780.01 | 5.704143 | 0.001361 | 0.017074 | Chr1 | 45090944 | 45092599 | 1656 |
| novel.4996 | -2.49807 | 0.001362 | 0.017074 | Chr5 | 15308502 | 15313575 | 1482 |
| novel.6929 | 2.847401 | 0.001367 | 0.017132 | Chr7 | 29510121 | 29511462 | 553 |
| MsG0180003710.01 | 1.76958 | 0.001369 | 0.017152 | Chr1 | 67185846 | 67191956 | 1245 |
| novel.219 | -4.46955 | 0.00137 | 0.01716 | Chr1 | 34098264 | 34101945 | 2434 |
| MsG0580025453.01 | 2.172803 | 0.001371 | 0.017162 | Chr5 | 18905391 | 18905993 | 603 |
| MsG0580029684.01 | 1.038813 | 0.001376 | 0.017224 | Chr5 | 1.01E+08 | 1.01E+08 | 909 |
| MsG0580028701.01 | -2.28823 | 0.001377 | 0.017228 | Chr5 | 85037311 | 85039828 | 1026 |
| MsG0380017189.01 | -2.52198 | 0.001378 | 0.017228 | Chr3 | 93148720 | 93152515 | 947 |
| MsG0080049010.01 | 1.780656 | 0.00138 | 0.017244 | contig605end | 10906 | 11764 | 699 |
| MsG0380014087.01 | -5.85936 | 0.001382 | 0.01727 | Chr3 | 49368660 | 49374662 | 795 |
| MsG0680031997.01 | 1.560783 | 0.001385 | 0.017293 | Chr6 | 31640177 | 31642778 | 2585 |
| novel.2997 | -6.34234 | 0.001392 | 0.017374 | Chr3 | 70719729 | 70721329 | 781 |
| MsG0880045134.01 | 5.114211 | 0.001394 | 0.017399 | Chr8 | 54662751 | 54664061 | 612 |
| MsG0180000883.01 | -1.77002 | 0.001396 | 0.017402 | Chr1 | 12693061 | 12700885 | 1006 |
| MsG0480023482.01 | -2.39446 | 0.001396 | 0.017402 | Chr4 | 85808155 | 85821282 | 2781 |
| MsG0780039663.01 | -3.59078 | 0.001396 | 0.017402 | Chr7 | 67357565 | 67358601 | 765 |
| novel.135 | 5.7298 | 0.001405 | 0.017499 | Chr1 | 17451856 | 17452490 | 635 |
| MsG0280008761.01 | -2.31136 | 0.001406 | 0.017507 | Chr2 | 37954939 | 37958189 | 831 |
| MsG0380012207.01 | -2.28689 | 0.001406 | 0.017507 | Chr3 | 12699531 | 12703358 | 3828 |
| MsG0680033993.01 | 2.034331 | 0.001407 | 0.017507 | Chr6 | 78247863 | 78251014 | 2463 |
| MsG0480020829.01 | 1.529774 | 0.001408 | 0.017507 | Chr4 | 49252746 | 49253336 | 306 |
| MsG0480020910.01 | -2.78668 | 0.001409 | 0.017516 | Chr4 | 50451088 | 50457870 | 846 |
| MsG0680032563.01 | 4.256617 | 0.001415 | 0.017589 | Chr6 | 44894987 | 44900212 | 1087 |
| novel.8607 | -5.75938 | 0.001419 | 0.017628 | Chr8 | 61489859 | 61491902 | 688 |
| MsG0380012321.01 | 1.41188 | 0.001423 | 0.017667 | Chr3 | 14846367 | 14853532 | 4362 |
| MsG0580025410.01 | -2.15934 | 0.001429 | 0.017729 | Chr5 | 18206705 | 18210275 | 534 |
| novel.7845 | -2.64741 | 0.001431 | 0.017747 | Chr8 | 9341711 | 9344104 | 650 |
| MsG0480023639.01 | -2.0397 | 0.001434 | 0.017781 | Chr4 | 87985503 | 87988684 | 1645 |
| MsG0380012875.01 | -2.31591 | 0.001443 | 0.017889 | Chr3 | 26166171 | 26168758 | 1359 |
| novel.7529 | -1.18598 | 0.001454 | 0.018018 | Chr7 | 55473869 | 55474616 | 631 |
| MsG0680030812.01 | 5.716298 | 0.001456 | 0.018027 | Chr6 | 9795797 | 9796781 | 915 |
| MsG0480021443.01 | 2.689918 | 0.001459 | 0.018059 | Chr4 | 57923087 | 57925863 | 2586 |
| novel.2605 | -3.20379 | 0.00146 | 0.01806 | Chr3 | 94363305 | 94365366 | 846 |
| novel.2563 | -6.82636 | 0.001464 | 0.018104 | Chr3 | 86407691 | 86409442 | 1404 |
| MsG0280011274.01 | 1.651529 | 0.00147 | 0.018166 | Chr2 | 81819835 | 81830289 | 4752 |
| MsG0280007950.01 | 1.408754 | 0.001472 | 0.018188 | Chr2 | 23570279 | 23571142 | 864 |
| MsG0780037846.01 | 2.096135 | 0.001473 | 0.018188 | Chr7 | 34396640 | 34400322 | 1842 |
| MsG0880042085.01 | -1.85201 | 0.001474 | 0.018188 | Chr8 | 3527216 | 3529693 | 2478 |
| MsG0780036750.01 | -5.74623 | 0.001474 | 0.018188 | Chr7 | 13157087 | 13157317 | 231 |
| novel.2803 | 3.54965 | 0.001475 | 0.01819 | Chr3 | 24028016 | 24030506 | 1685 |
| MsG0780039113.01 | -1.96172 | 0.001485 | 0.018296 | Chr7 | 58139120 | 58145956 | 3502 |
| MsG0780036881.01 | 1.41071 | 0.001485 | 0.018296 | Chr7 | 15611893 | 15617488 | 1819 |
| novel.262 | 6.088689 | 0.001486 | 0.01831 | Chr1 | 44095069 | 44109232 | 611 |
| MsG0880047240.01 | -1.39011 | 0.00149 | 0.018347 | Chr8 | 83720979 | 83724914 | 1287 |
| MsG0180000024.01 | -4.27832 | 0.001492 | 0.01836 | Chr1 | 456456 | 457159 | 618 |
| MsG0180003471.01 | -4.72666 | 0.001493 | 0.01836 | Chr1 | 62758121 | 62758582 | 462 |
| novel.6667 | -2.10448 | 0.001495 | 0.018373 | Chr6 | 97634531 | 97635673 | 698 |
| MsG0680031120.01 | -2.04571 | 0.001497 | 0.018391 | Chr6 | 14774568 | 14775643 | 984 |
| MsG0380017731.01 | -3.68181 | 0.001501 | 0.018433 | Chr3 | 1E+08 | 1E+08 | 2680 |
| MsG0680031136.01 | -5.69944 | 0.001502 | 0.018436 | Chr6 | 15014276 | 15015262 | 987 |
| MsG0880046194.01 | -1.02102 | 0.001504 | 0.018456 | Chr8 | 70387192 | 70388771 | 903 |
| novel.8687 | -5.8902 | 0.001508 | 0.018498 | Chr8 | 74390599 | 74392863 | 1333 |
| MsG0180006099.01 | 1.763286 | 0.00151 | 0.018518 | Chr1 | 1E+08 | 1E+08 | 401 |
| MsG0880042289.01 | 1.803043 | 0.001513 | 0.018539 | Chr8 | 6554956 | 6558901 | 2022 |
| MsG0580028630.01 | 2.380494 | 0.001513 | 0.018539 | Chr5 | 83806969 | 83808487 | 1449 |
| MsG0880043364.01 | -1.52025 | 0.001518 | 0.018592 | Chr8 | 23678559 | 23685474 | 3191 |
| MsG0280006644.01 | 1.358912 | 0.001518 | 0.018592 | Chr2 | 4432569 | 4436433 | 1192 |
| MsG0680035706.01 | -2.15528 | 0.001519 | 0.018592 | Chr6 | 1.11E+08 | 1.11E+08 | 1885 |
| novel.5922 | -1.43854 | 0.001526 | 0.018662 | Chr6 | 82932204 | 82938141 | 1900 |
| MsG0180003345.01 | 6.135745 | 0.001526 | 0.018662 | Chr1 | 60906831 | 60908890 | 1494 |
| MsG0880044436.01 | 1.04647 | 0.00153 | 0.018703 | Chr8 | 42024899 | 42026810 | 1175 |
| MsG0280006497.01 | -3.59134 | 0.001531 | 0.018714 | Chr2 | 2752318 | 2759090 | 1269 |
| MsG0880046260.01 | -2.86047 | 0.001534 | 0.018735 | Chr8 | 71189587 | 71192056 | 1500 |
| novel.6516 | 1.982252 | 0.001541 | 0.018797 | Chr6 | 65778501 | 65779004 | 504 |
| novel.5043 | -5.66551 | 0.001541 | 0.018797 | Chr5 | 26631060 | 26631510 | 400 |
| MsG0580027688.01 | -6.82076 | 0.001541 | 0.018797 | Chr5 | 66160138 | 66161472 | 1335 |
| MsG0280009515.01 | 2.549531 | 0.001551 | 0.018905 | Chr2 | 53454345 | 53459313 | 2475 |
| MsG0680032095.01 | -2.5514 | 0.001552 | 0.018906 | Chr6 | 33562001 | 33566796 | 402 |
| MsG0780039203.01 | -5.81202 | 0.001553 | 0.01892 | Chr7 | 59715737 | 59717423 | 1026 |
| MsG0080048230.01 | 1.073186 | 0.001555 | 0.018928 | contig247end | 14125 | 16324 | 1365 |
| MsG0280010299.01 | 1.273569 | 0.001556 | 0.018934 | Chr2 | 67574662 | 67576824 | 642 |
| MsG0780036068.01 | -2.10905 | 0.001561 | 0.01899 | Chr7 | 2059309 | 2060204 | 519 |
| MsG0680035716.01 | -1.35271 | 0.001562 | 0.018999 | Chr6 | 1.11E+08 | 1.11E+08 | 3246 |
| MsG0480021362.01 | -1.28588 | 0.001563 | 0.019001 | Chr4 | 57087078 | 57092833 | 1598 |
| MsG0580024177.01 | -2.47463 | 0.001568 | 0.01905 | Chr5 | 1591675 | 1592840 | 948 |
| MsG0280011222.01 | -1.17359 | 0.001574 | 0.019112 | Chr2 | 81149691 | 81152446 | 1465 |
| novel.3774 | -2.27129 | 0.001586 | 0.019236 | Chr4 | 3835004 | 3837763 | 2580 |
| MsG0380011483.01 | -5.25398 | 0.001586 | 0.019237 | Chr3 | 76094 | 76651 | 558 |
| MsG0680030627.01 | -1.4 | 0.00159 | 0.019267 | Chr6 | 6368155 | 6372149 | 825 |
| novel.2409 | -3.13796 | 0.001592 | 0.019284 | Chr3 | 61425126 | 61430431 | 1760 |
| MsG0580026480.01 | -6.83553 | 0.001593 | 0.019284 | Chr5 | 38257604 | 38260744 | 2013 |
| MsG0180001124.01 | -4.53588 | 0.001593 | 0.019284 | Chr1 | 16316652 | 16319392 | 2212 |
| MsG0480019256.01 | -5.83604 | 0.001599 | 0.019343 | Chr4 | 17225240 | 17231240 | 1149 |
| MsG0480021260.01 | -1.17498 | 0.001609 | 0.019459 | Chr4 | 56055783 | 56058144 | 1042 |
| novel.4673 | 2.054685 | 0.00161 | 0.019469 | Chr5 | 72979363 | 72989490 | 1497 |
| MsG0380014892.01 | 2.076891 | 0.001614 | 0.019509 | Chr3 | 62121830 | 62124223 | 2394 |
| MsG0480018388.01 | -2.08539 | 0.001616 | 0.019518 | Chr4 | 4229282 | 4231192 | 930 |
| MsG0480023250.01 | 1.427204 | 0.00162 | 0.019565 | Chr4 | 82651086 | 82651772 | 687 |
| MsG0080048842.01 | 1.385295 | 0.001627 | 0.019632 | contig478end | 20043 | 23296 | 1194 |
| novel.7962 | 1.907406 | 0.001628 | 0.019647 | Chr8 | 30789635 | 30791185 | 1551 |
| MsG0680032762.01 | 1.018998 | 0.001632 | 0.019672 | Chr6 | 49009396 | 49012116 | 1258 |
| novel.6833 | -5.71583 | 0.001636 | 0.019713 | Chr7 | 11534516 | 11535639 | 703 |
| MsG0580024596.01 | 1.497704 | 0.001646 | 0.019816 | Chr5 | 6726729 | 6729920 | 1232 |
| novel.6184 | -4.26863 | 0.001649 | 0.019843 | Chr6 | 7676662 | 7677233 | 548 |
| MsG0580024272.01 | 5.773554 | 0.001653 | 0.019878 | Chr5 | 2806775 | 2809683 | 1957 |
| novel.4878 | 5.771765 | 0.001655 | 0.019901 | Chr5 | 1.05E+08 | 1.05E+08 | 658 |
| novel.8789 | 3.985067 | 0.001656 | 0.019907 | contig105end | 16519 | 18925 | 328 |
| novel.7523 | -1.73988 | 0.001658 | 0.019915 | Chr7 | 54264916 | 54280912 | 2507 |
| novel.7596 | -2.15091 | 0.00166 | 0.019931 | Chr7 | 67648410 | 67654405 | 3127 |
| novel.4774 | -1.74859 | 0.001662 | 0.019946 | Chr5 | 90433932 | 90437424 | 1650 |
| novel.1330 | 3.190943 | 0.001671 | 0.020044 | Chr2 | 28042811 | 28045519 | 855 |
| MsG0680035045.01 | 1.526143 | 0.001672 | 0.02005 | Chr6 | 98164788 | 98170092 | 1233 |
| novel.1908 | -6.0327 | 0.001679 | 0.020119 | Chr2 | 50908870 | 50909240 | 371 |
| novel.1130 | 1.363063 | 0.001679 | 0.020119 | Chr1 | 99287585 | 99290310 | 1011 |
| MsG0680032109.01 | -3.13046 | 0.001683 | 0.02016 | Chr6 | 33907839 | 33910652 | 2061 |
| MsG0780041130.01 | -5.84269 | 0.001686 | 0.020182 | Chr7 | 86916779 | 86925469 | 2220 |
| MsG0680033402.01 | -5.78738 | 0.001693 | 0.020253 | Chr6 | 64094662 | 64098008 | 1158 |
| novel.3817 | -1.26883 | 0.001694 | 0.020264 | Chr4 | 13730248 | 13733524 | 2959 |
| MsG0180004121.01 | -2.96374 | 0.0017 | 0.020322 | Chr1 | 73694349 | 73703595 | 2198 |
| novel.7663 | -3.89174 | 0.001703 | 0.020349 | Chr7 | 78299414 | 78302954 | 1199 |
| novel.3707 | 1.407306 | 0.001709 | 0.020412 | Chr4 | 87678965 | 87684242 | 2497 |
| MsG0280006832.01 | -1.28897 | 0.001709 | 0.020412 | Chr2 | 6859743 | 6861626 | 920 |
| novel.6848 | -5.08806 | 0.001713 | 0.02044 | Chr7 | 14412205 | 14416811 | 469 |
| novel.3645 | -2.06736 | 0.001713 | 0.02044 | Chr4 | 79781398 | 79786670 | 1224 |
| MsG0580028413.01 | 1.651817 | 0.001714 | 0.02044 | Chr5 | 80237340 | 80242080 | 778 |
| MsG0080047803.01 | -3.52067 | 0.001714 | 0.02044 | contig108end | 18858 | 20540 | 907 |
| MsG0580024233.01 | 3.928686 | 0.001716 | 0.020459 | Chr5 | 2405571 | 2411435 | 1902 |
| MsG0280009691.01 | -1.8914 | 0.001718 | 0.020462 | Chr2 | 56570329 | 56571132 | 804 |
| MsG0880046839.01 | -1.15745 | 0.001718 | 0.020462 | Chr8 | 78966892 | 78967209 | 318 |
| novel.6273 | 1.763866 | 0.001719 | 0.020462 | Chr6 | 19067738 | 19068680 | 815 |
| MsG0480018950.01 | 1.33064 | 0.001722 | 0.020497 | Chr4 | 12232768 | 12249494 | 6884 |
| MsG0880045888.01 | 1.494385 | 0.001726 | 0.020526 | Chr8 | 66009845 | 66011191 | 1347 |
| MsG0480023453.01 | 1.117935 | 0.001726 | 0.020526 | Chr4 | 85333066 | 85333755 | 690 |
| MsG0280011165.01 | -2.55809 | 0.001734 | 0.020608 | Chr2 | 80409183 | 80412871 | 1970 |
| MsG0080047817.01 | -6.76082 | 0.001735 | 0.02062 | contig113end | 30734 | 31213 | 480 |
| MsG0180003728.01 | 1.618178 | 0.001739 | 0.020656 | Chr1 | 67360376 | 67362329 | 1860 |
| novel.6956 | 2.357513 | 0.001744 | 0.020701 | Chr7 | 35571721 | 35572620 | 900 |
| MsG0780036481.01 | -1.28363 | 0.001749 | 0.020746 | Chr7 | 8718366 | 8718910 | 450 |
| novel.9283 | -4.07003 | 0.001753 | 0.020786 | contig592end | 22804 | 24413 | 1266 |
| MsG0180000955.01 | 1.76796 | 0.001757 | 0.020831 | Chr1 | 13631620 | 13633133 | 1514 |
| novel.8509 | 1.78337 | 0.00176 | 0.020851 | Chr8 | 44167883 | 44176376 | 1893 |
| novel.6684 | 5.962247 | 0.00176 | 0.020851 | Chr6 | 1E+08 | 1E+08 | 712 |
| MsG0580027825.01 | 1.144864 | 0.001763 | 0.020876 | Chr5 | 69178207 | 69183335 | 597 |
| MsG0380014412.01 | -5.90194 | 0.001766 | 0.020908 | Chr3 | 54352326 | 54363306 | 786 |
| MsG0880044861.01 | -3.29693 | 0.001773 | 0.020974 | Chr8 | 50335393 | 50343834 | 927 |
| MsG0780038504.01 | -3.50599 | 0.001775 | 0.020992 | Chr7 | 47760041 | 47763606 | 1037 |
| MsG0380012173.01 | 1.614275 | 0.001778 | 0.021019 | Chr3 | 12156096 | 12156350 | 255 |
| MsG0680032607.01 | 1.815707 | 0.001782 | 0.021063 | Chr6 | 45877178 | 45877465 | 288 |
| MsG0880045803.01 | -1.10702 | 0.001784 | 0.021078 | Chr8 | 64689990 | 64699061 | 2921 |
| MsG0280009888.01 | 1.744896 | 0.00179 | 0.021134 | Chr2 | 60146342 | 60153241 | 1192 |
| novel.106 | -3.34843 | 0.001793 | 0.021152 | Chr1 | 13654951 | 13656682 | 1064 |
| MsG0480021940.01 | 1.228739 | 0.001793 | 0.021152 | Chr4 | 66191310 | 66196743 | 2389 |
| novel.550 | -5.70514 | 0.001794 | 0.021152 | Chr1 | 97351062 | 97352776 | 835 |
| MsG0280007347.01 | -4.55754 | 0.001794 | 0.021152 | Chr2 | 14224785 | 14231149 | 2145 |
| novel.7865 | 4.228614 | 0.001799 | 0.021196 | Chr8 | 12075105 | 12075384 | 240 |
| MsG0380011642.01 | -2.08941 | 0.0018 | 0.021196 | Chr3 | 2932137 | 2934677 | 1102 |
| MsG0880043412.01 | -1.94379 | 0.001802 | 0.021214 | Chr8 | 24420793 | 24423392 | 429 |
| MsG0880046920.01 | -2.37638 | 0.001809 | 0.021286 | Chr8 | 79896383 | 79905094 | 906 |
| novel.5431 | -3.86827 | 0.001815 | 0.021346 | Chr5 | 99845806 | 99847181 | 1022 |
| novel.2217 | -5.82362 | 0.00183 | 0.02151 | Chr3 | 15758408 | 15759623 | 728 |
| MsG0580027430.01 | 6.045135 | 0.001832 | 0.021515 | Chr5 | 60513940 | 60523930 | 2733 |
| MsG0680035117.01 | -2.36392 | 0.001832 | 0.021515 | Chr6 | 99208858 | 99209100 | 243 |
| novel.8689 | -6.79747 | 0.001838 | 0.021576 | Chr8 | 75373622 | 75374284 | 663 |
| MsG0680032003.01 | -1.07205 | 0.001842 | 0.021605 | Chr6 | 31757657 | 31760827 | 3171 |
| novel.4661 | -2.37757 | 0.001846 | 0.021653 | Chr5 | 71752993 | 71753924 | 400 |
| MsG0480019218.01 | -1.52849 | 0.001849 | 0.021671 | Chr4 | 16346507 | 16347004 | 498 |
| MsG0280008053.01 | -6.03864 | 0.00185 | 0.021682 | Chr2 | 24866736 | 24870287 | 1902 |
| MsG0580028552.01 | -1.13678 | 0.001857 | 0.021746 | Chr5 | 82185895 | 82187574 | 1680 |
| MsG0180000822.01 | 1.884253 | 0.001857 | 0.021748 | Chr1 | 11666867 | 11669775 | 1115 |
| novel.5936 | -4.99678 | 0.001864 | 0.021804 | Chr6 | 85544926 | 85553282 | 1242 |
| MsG0580025682.01 | -2.36158 | 0.001864 | 0.021804 | Chr5 | 22587487 | 22601365 | 3114 |
| MsG0780041606.01 | -1.17747 | 0.001865 | 0.021804 | Chr7 | 92970473 | 92972894 | 1154 |
| MsG0480018676.01 | -1.40589 | 0.00187 | 0.02185 | Chr4 | 8504546 | 8508754 | 1448 |
| MsG0380017223.01 | -3.2919 | 0.001886 | 0.021998 | Chr3 | 93743474 | 93747208 | 1079 |
| novel.4936 | -5.48367 | 0.001887 | 0.022007 | Chr5 | 5361434 | 5363887 | 883 |
| MsG0180000725.01 | -5.05254 | 0.001889 | 0.022014 | Chr1 | 10299519 | 10305110 | 1800 |
| MsG0580028899.01 | -2.38668 | 0.00189 | 0.022025 | Chr5 | 88284097 | 88288739 | 1711 |
| novel.7248 | -4.77417 | 0.001891 | 0.022028 | Chr7 | 89428561 | 89429339 | 402 |
| MsG0280009745.01 | -5.77291 | 0.001896 | 0.022053 | Chr2 | 57455858 | 57458430 | 1234 |
| novel.2344 | -1.53024 | 0.001899 | 0.022084 | Chr3 | 44691077 | 44693240 | 795 |
| MsG0180003713.01 | -2.03715 | 0.001915 | 0.022258 | Chr1 | 67224994 | 67237316 | 1944 |
| MsG0380016620.01 | 1.166109 | 0.001916 | 0.022265 | Chr3 | 85807942 | 85810631 | 741 |
| MsG0280007934.01 | -3.79461 | 0.00192 | 0.0223 | Chr2 | 23247364 | 23247960 | 597 |
| MsG0580025219.01 | -1.59944 | 0.001924 | 0.022333 | Chr5 | 15409885 | 15413934 | 1182 |
| MsG0180002949.01 | -5.64489 | 0.001924 | 0.022333 | Chr1 | 53669647 | 53674352 | 1056 |
| MsG0780037244.01 | -4.98588 | 0.00193 | 0.02239 | Chr7 | 22379126 | 22380204 | 685 |
| novel.9170 | -1.06774 | 0.001931 | 0.022396 | contig527end | 4482 | 9141 | 2061 |
| MsG0380017484.01 | -4.28429 | 0.001933 | 0.022415 | Chr3 | 96794059 | 96796673 | 1739 |
| novel.7776 | 1.143056 | 0.001935 | 0.02243 | Chr7 | 93925578 | 93928402 | 1940 |
| MsG0380014593.01 | -1.39009 | 0.001936 | 0.022432 | Chr3 | 56910303 | 56914432 | 3812 |
| MsG0580025224.01 | -3.42463 | 0.001939 | 0.022455 | Chr5 | 15464852 | 15467503 | 1332 |
| MsG0180001538.01 | 1.598552 | 0.001942 | 0.022482 | Chr1 | 22931130 | 22934002 | 1200 |
| novel.4054 | -5.10357 | 0.00196 | 0.022668 | Chr4 | 63186361 | 63187056 | 696 |
| novel.8650 | -1.1802 | 0.00196 | 0.022668 | Chr8 | 69065522 | 69068107 | 1118 |
| MsG0780041826.01 | 1.444798 | 0.00196 | 0.022668 | Chr7 | 95595849 | 95597823 | 1542 |
| MsG0680034927.01 | -3.37011 | 0.001966 | 0.022728 | Chr6 | 96728130 | 96728747 | 618 |
| novel.636 | 2.028114 | 0.001974 | 0.022803 | Chr1 | 4946574 | 4948905 | 1806 |
| MsG0180003359.01 | 1.270457 | 0.001982 | 0.02289 | Chr1 | 61064256 | 61065209 | 954 |
| MsG0180000590.01 | -3.06166 | 0.001985 | 0.022917 | Chr1 | 8385017 | 8385517 | 501 |
| MsG0880044177.01 | -5.73111 | 0.001988 | 0.022933 | Chr8 | 37050187 | 37050695 | 330 |
| novel.4483 | -5.73111 | 0.001988 | 0.022933 | Chr5 | 31370864 | 31372918 | 490 |
| MsG0180002897.01 | -1.54437 | 0.002001 | 0.023067 | Chr1 | 52321399 | 52325510 | 2343 |
| MsG0180004941.01 | -2.14127 | 0.002002 | 0.023073 | Chr1 | 84712640 | 84713614 | 321 |
| MsG0880042996.01 | -6.77959 | 0.002003 | 0.023073 | Chr8 | 17327157 | 17328333 | 810 |
| MsG0280010391.01 | 1.086743 | 0.002007 | 0.023114 | Chr2 | 68961887 | 68967391 | 1948 |
| novel.8991 | -2.31907 | 0.002009 | 0.02312 | contig408end | 195682 | 196652 | 568 |
| novel.7025 | -5.70113 | 0.00201 | 0.02313 | Chr7 | 51743113 | 51748928 | 616 |
| MsG0680033187.01 | -2.40903 | 0.002014 | 0.023163 | Chr6 | 58987937 | 58990251 | 450 |
| MsG0380014894.01 | 2.532767 | 0.002025 | 0.023285 | Chr3 | 62138846 | 62141227 | 2382 |
| MsG0880043869.01 | -4.64662 | 0.002029 | 0.023319 | Chr8 | 31887060 | 31890186 | 945 |
| novel.1156 | -6.13002 | 0.00203 | 0.023319 | Chr1 | 1.02E+08 | 1.02E+08 | 1761 |
| novel.146 | 1.413145 | 0.002041 | 0.023438 | Chr1 | 18775056 | 18782528 | 1398 |
| MsG0580027652.01 | -1.11294 | 0.002044 | 0.023465 | Chr5 | 65245227 | 65258124 | 667 |
| novel.5989 | 4.350676 | 0.002047 | 0.02349 | Chr6 | 95671476 | 95673338 | 723 |
| MsG0180005424.01 | 2.12515 | 0.002048 | 0.023492 | Chr1 | 91690563 | 91694891 | 337 |
| MsG0680033443.01 | -5.72697 | 0.002049 | 0.023501 | Chr6 | 65108176 | 65113875 | 1737 |
| MsG0680032356.01 | 3.221144 | 0.002053 | 0.023538 | Chr6 | 39227485 | 39231412 | 294 |
| MsG0080047802.01 | -3.79751 | 0.002054 | 0.023541 | contig108end | 9812 | 14952 | 1498 |
| MsG0580025980.01 | -5.78641 | 0.002055 | 0.023544 | Chr5 | 28546913 | 28554026 | 2348 |
| MsG0880046650.01 | -1.0044 | 0.002058 | 0.023559 | Chr8 | 76149895 | 76156383 | 2121 |
| novel.9190 | -1.35299 | 0.002066 | 0.023637 | contig538end | 4983 | 9300 | 2136 |
| MsG0180004150.01 | -2.23022 | 0.002067 | 0.02364 | Chr1 | 74038044 | 74050479 | 2613 |
| MsG0780035960.01 | -2.55046 | 0.002068 | 0.023646 | Chr7 | 620929 | 627218 | 2804 |
| MsG0280008255.01 | 2.271542 | 0.002077 | 0.023733 | Chr2 | 28065966 | 28069305 | 1113 |
| MsG0380017065.01 | -2.57893 | 0.002082 | 0.023773 | Chr3 | 91531165 | 91533107 | 579 |
| novel.7565 | -1.39737 | 0.002086 | 0.023814 | Chr7 | 64316861 | 64320151 | 1691 |
| MsG0680032831.01 | -1.57305 | 0.002093 | 0.023879 | Chr6 | 50621399 | 50625068 | 3396 |
| novel.537 | 5.837151 | 0.002097 | 0.023915 | Chr1 | 95631118 | 95635217 | 764 |
| novel.3722 | -4.84784 | 0.002106 | 0.024001 | Chr4 | 88724761 | 88725522 | 762 |
| MsG0880042110.01 | 5.019244 | 0.002109 | 0.024033 | Chr8 | 3820515 | 3821573 | 1059 |
| novel.6566 | -2.67919 | 0.00211 | 0.024036 | Chr6 | 74696632 | 74702991 | 1079 |
| MsG0880045965.01 | -2.1487 | 0.002112 | 0.024052 | Chr8 | 67017637 | 67019737 | 555 |
| MsG0280007750.01 | -2.79146 | 0.002116 | 0.024083 | Chr2 | 20012695 | 20013453 | 759 |
| novel.2544 | 1.012817 | 0.002117 | 0.024083 | Chr3 | 84898772 | 84902290 | 903 |
| novel.4316 | -1.44677 | 0.002123 | 0.024143 | Chr5 | 5577188 | 5582209 | 1013 |
| MsG0480023281.01 | 1.02111 | 0.002124 | 0.024144 | Chr4 | 83160691 | 83164079 | 1368 |
| MsG0780040558.01 | 1.24843 | 0.002125 | 0.02415 | Chr7 | 79179023 | 79184234 | 2089 |
| MsG0580024756.01 | 4.175093 | 0.002128 | 0.024173 | Chr5 | 9288955 | 9293235 | 800 |
| novel.1154 | -4.22822 | 0.002129 | 0.024173 | Chr1 | 1.02E+08 | 1.02E+08 | 462 |
| novel.4880 | -4.20242 | 0.002143 | 0.024324 | Chr5 | 1.06E+08 | 1.06E+08 | 1257 |
| MsG0780037194.01 | -1.75387 | 0.002145 | 0.024338 | Chr7 | 21547275 | 21558582 | 1068 |
| novel.5302 | -2.20427 | 0.002151 | 0.024404 | Chr5 | 78949263 | 78953620 | 744 |
| MsG0080048113.01 | 2.934073 | 0.002156 | 0.024445 | contig211end | 282 | 2349 | 682 |
| MsG0280010482.01 | 2.391507 | 0.00216 | 0.024489 | Chr2 | 70350337 | 70355797 | 1014 |
| novel.3903 | -5.9696 | 0.002173 | 0.024623 | Chr4 | 30885695 | 30887384 | 1082 |
| MsG0780040005.01 | -2.18209 | 0.002178 | 0.024671 | Chr7 | 71843328 | 71846047 | 1622 |
| MsG0480019201.01 | -2.41531 | 0.002184 | 0.024735 | Chr4 | 16037040 | 16041035 | 1281 |
| MsG0580025359.01 | -1.70639 | 0.002188 | 0.024759 | Chr5 | 17440732 | 17443742 | 1158 |
| MsG0880042800.01 | 1.530937 | 0.002189 | 0.024759 | Chr8 | 14224454 | 14230026 | 987 |
| novel.6385 | 5.670527 | 0.002201 | 0.024881 | Chr6 | 37640632 | 37642869 | 527 |
| MsG0180006085.01 | -1.40336 | 0.002205 | 0.024913 | Chr1 | 99976695 | 99988588 | 2124 |
| MsG0680032254.01 | 1.518748 | 0.002208 | 0.024928 | Chr6 | 37407309 | 37419210 | 3630 |
| MsG0380015434.01 | -1.34605 | 0.002208 | 0.024928 | Chr3 | 70194524 | 70203537 | 1669 |
| novel.4877 | -1.20871 | 0.00221 | 0.024941 | Chr5 | 1.05E+08 | 1.05E+08 | 1416 |
| MsG0880046355.01 | -5.75811 | 0.002214 | 0.024974 | Chr8 | 72323438 | 72325705 | 1179 |
| novel.6633 | 6.379878 | 0.002216 | 0.024988 | Chr6 | 89775299 | 89775969 | 431 |
| MsG0780036011.01 | -5.4303 | 0.002217 | 0.024988 | Chr7 | 1309660 | 1309854 | 195 |
| MsG0580024408.01 | -2.78889 | 0.002217 | 0.024988 | Chr5 | 4450142 | 4450839 | 606 |
| MsG0780040260.01 | -1.8256 | 0.002226 | 0.025064 | Chr7 | 75034920 | 75035342 | 423 |
| MsG0680030996.01 | -2.35305 | 0.002226 | 0.025064 | Chr6 | 12662425 | 12665538 | 1746 |
| novel.685 | 1.676812 | 0.002228 | 0.025078 | Chr1 | 10919010 | 10923625 | 1024 |
| MsG0180005150.01 | -3.18879 | 0.00223 | 0.025094 | Chr1 | 87635855 | 87636400 | 450 |
| MsG0480022359.01 | -1.06964 | 0.002232 | 0.025101 | Chr4 | 71556440 | 71556907 | 369 |
| MsG0780040850.01 | -5.92347 | 0.002232 | 0.025101 | Chr7 | 83318871 | 83319774 | 729 |
| MsG0180000471.01 | -2.61785 | 0.002235 | 0.025125 | Chr1 | 6517585 | 6518019 | 435 |
| MsG0480018484.01 | -1.46698 | 0.002239 | 0.025157 | Chr4 | 5594718 | 5604797 | 2388 |
| MsG0580027789.01 | -1.23534 | 0.00224 | 0.025163 | Chr5 | 68487914 | 68490275 | 2172 |
| MsG0380012176.01 | -1.32263 | 0.002242 | 0.025168 | Chr3 | 12206488 | 12206928 | 441 |
| novel.3812 | 3.19245 | 0.002242 | 0.025168 | Chr4 | 11582158 | 11584432 | 931 |
| MsG0180001183.01 | -1.89884 | 0.002246 | 0.025203 | Chr1 | 16945465 | 16950577 | 2115 |
| novel.5536 | -3.69212 | 0.002248 | 0.025207 | Chr6 | 5017784 | 5023562 | 1274 |
| novel.501 | 2.000231 | 0.002249 | 0.025214 | Chr1 | 89399026 | 89399676 | 447 |
| novel.8544 | 2.34799 | 0.002255 | 0.025263 | Chr8 | 50989381 | 50997903 | 2792 |
| novel.6887 | -5.42482 | 0.002255 | 0.025263 | Chr7 | 21059008 | 21059838 | 831 |
| novel.7970 | -5.58989 | 0.002256 | 0.025265 | Chr8 | 33760972 | 33761938 | 889 |
| MsG0680032846.01 | 1.849993 | 0.002259 | 0.025287 | Chr6 | 50982026 | 50986107 | 942 |
| MsG0380015676.01 | 1.758693 | 0.002261 | 0.025304 | Chr3 | 73508216 | 73530639 | 2640 |
| MsG0180003120.01 | -4.62716 | 0.002264 | 0.025328 | Chr1 | 56874717 | 56877129 | 1056 |
| MsG0880045711.01 | -6.05679 | 0.002268 | 0.025354 | Chr8 | 63107714 | 63107932 | 219 |
| MsG0880046621.01 | -6.05679 | 0.002268 | 0.025354 | Chr8 | 75773896 | 75775447 | 891 |
| MsG0880042475.01 | -1.9542 | 0.002275 | 0.025398 | Chr8 | 9322629 | 9334581 | 2932 |
| MsG0480022301.01 | 1.114214 | 0.002275 | 0.025398 | Chr4 | 70865188 | 70870043 | 47 |
| novel.2779 | 1.582109 | 0.002276 | 0.025398 | Chr3 | 18834232 | 18837343 | 1404 |
| MsG0280008438.01 | 2.594361 | 0.002277 | 0.025398 | Chr2 | 31896447 | 31897055 | 609 |
| novel.2351 | -2.31109 | 0.002282 | 0.025443 | Chr3 | 45603791 | 45606503 | 1321 |
| MsG0580025590.01 | -5.85045 | 0.002291 | 0.025526 | Chr5 | 20943573 | 20949156 | 660 |
| novel.8719 | 1.338181 | 0.002307 | 0.025699 | Chr8 | 81088220 | 81093390 | 2239 |
| MsG0780036694.01 | -3.00071 | 0.002311 | 0.025703 | Chr7 | 12016100 | 12019408 | 1701 |
| novel.1214 | 1.618047 | 0.002311 | 0.025703 | Chr2 | 7427578 | 7429721 | 2144 |
| MsG0380013300.01 | -1.23722 | 0.002311 | 0.025703 | Chr3 | 34676904 | 34680365 | 1732 |
| MsG0880043460.01 | -2.39476 | 0.002312 | 0.025703 | Chr8 | 25167669 | 25168295 | 627 |
| MsG0680032413.01 | -1.26281 | 0.002314 | 0.025711 | Chr6 | 40580621 | 40587729 | 751 |
| MsG0380013481.01 | 1.21898 | 0.002317 | 0.025739 | Chr3 | 39084237 | 39087593 | 1127 |
| MsG0380012082.01 | -5.63968 | 0.002325 | 0.025814 | Chr3 | 10666356 | 10671394 | 2937 |
| MsG0180001522.01 | 1.813044 | 0.002328 | 0.025829 | Chr1 | 22626180 | 22628242 | 807 |
| MsG0280011237.01 | -5.77902 | 0.00233 | 0.025837 | Chr2 | 81326906 | 81328397 | 1107 |
| MsG0780039224.01 | 1.048622 | 0.002331 | 0.025841 | Chr7 | 60136421 | 60140705 | 2029 |
| novel.5236 | 5.581746 | 0.002339 | 0.025907 | Chr5 | 68301123 | 68301786 | 664 |
| MsG0480019592.01 | 1.153357 | 0.00234 | 0.025907 | Chr4 | 22870654 | 22872864 | 1064 |
| novel.649 | -2.75833 | 0.00234 | 0.025907 | Chr1 | 6935963 | 6942326 | 1209 |
| novel.5524 | -2.83027 | 0.002341 | 0.025907 | Chr6 | 2981647 | 2982561 | 546 |
| MsG0180002686.01 | -2.2768 | 0.002343 | 0.025926 | Chr1 | 43142248 | 43146382 | 741 |
| MsG0480020709.01 | 3.226694 | 0.002355 | 0.026036 | Chr4 | 46763021 | 46765242 | 1473 |
| MsG0180004278.01 | -1.6017 | 0.002359 | 0.026077 | Chr1 | 75936482 | 75937167 | 477 |
| MsG0380015770.01 | 1.439369 | 0.002361 | 0.026086 | Chr3 | 74850694 | 74854348 | 1424 |
| MsG0380016178.01 | 1.059279 | 0.002368 | 0.026153 | Chr3 | 80011922 | 80012843 | 712 |
| MsG0480018982.01 | -1.02278 | 0.002376 | 0.026233 | Chr4 | 12844671 | 12847787 | 979 |
| MsG0280007577.01 | 4.98997 | 0.002377 | 0.026233 | Chr2 | 17506994 | 17517668 | 2352 |
| MsG0480018204.01 | -5.58329 | 0.00238 | 0.026254 | Chr4 | 1531695 | 1536424 | 2680 |
| novel.1528 | 2.53762 | 0.002384 | 0.026279 | Chr2 | 70337542 | 70349680 | 1040 |
| novel.5968 | 2.913476 | 0.002392 | 0.026356 | Chr6 | 91131983 | 91136400 | 2513 |
| MsG0280010957.01 | -1.81256 | 0.002396 | 0.026384 | Chr2 | 77615424 | 77619190 | 1662 |
| novel.4522 | -5.01138 | 0.002415 | 0.026582 | Chr5 | 43152439 | 43155942 | 420 |
| MsG0880045654.01 | -1.75191 | 0.002418 | 0.026604 | Chr8 | 62214256 | 62214573 | 318 |
| MsG0180000929.01 | -3.93539 | 0.002431 | 0.026734 | Chr1 | 13357851 | 13360726 | 1833 |
| novel.3965 | 4.206386 | 0.002432 | 0.026735 | Chr4 | 47443240 | 47445287 | 1613 |
| MsG0480020764.01 | -2.78226 | 0.002434 | 0.026735 | Chr4 | 47930574 | 47931635 | 729 |
| MsG0780036391.01 | 1.095417 | 0.002438 | 0.026759 | Chr7 | 7452053 | 7466474 | 4758 |
| MsG0280007036.01 | -1.69085 | 0.002438 | 0.026759 | Chr2 | 9728982 | 9735437 | 2652 |
| novel.6886 | -3.64601 | 0.002444 | 0.026817 | Chr7 | 20942037 | 20945512 | 1417 |
| MsG0180000652.01 | -1.18721 | 0.002451 | 0.026886 | Chr1 | 9227771 | 9229794 | 921 |
| MsG0180005047.01 | 1.02017 | 0.002455 | 0.026923 | Chr1 | 86166948 | 86171596 | 1863 |
| novel.7338 | -1.83875 | 0.00246 | 0.026966 | Chr7 | 10796618 | 10807762 | 1578 |
| MsG0180006026.01 | -3.13169 | 0.002465 | 0.02701 | Chr1 | 99015103 | 99016884 | 1290 |
| MsG0480022680.01 | -1.68593 | 0.002468 | 0.027038 | Chr4 | 75414343 | 75414888 | 546 |
| MsG0380013128.01 | -2.56559 | 0.002472 | 0.027063 | Chr3 | 30933916 | 30936289 | 757 |
| MsG0480022028.01 | 5.687016 | 0.002473 | 0.027071 | Chr4 | 67163807 | 67164274 | 468 |
| novel.8925 | 5.614525 | 0.002474 | 0.027071 | contig297end | 34558 | 35413 | 640 |
| MsG0880042911.01 | 1.18513 | 0.002489 | 0.027217 | Chr8 | 15807776 | 15810568 | 1020 |
| novel.3385 | -5.99185 | 0.002491 | 0.027223 | Chr4 | 29253369 | 29254475 | 976 |
| MsG0880043346.01 | -4.06578 | 0.002498 | 0.027294 | Chr8 | 23422800 | 23425799 | 2952 |
| MsG0380017246.01 | -3.29069 | 0.002502 | 0.027329 | Chr3 | 93997821 | 93999457 | 1389 |
| novel.5827 | 2.026563 | 0.002506 | 0.027359 | Chr6 | 64649621 | 64659166 | 1555 |
| MsG0180000360.01 | -1.43318 | 0.002507 | 0.027367 | Chr1 | 4864786 | 4869231 | 2692 |
| MsG0780040358.01 | -1.70794 | 0.002511 | 0.027398 | Chr7 | 76390209 | 76396847 | 2319 |
| novel.1006 | 1.260672 | 0.002513 | 0.02741 | Chr1 | 82635621 | 82639460 | 1463 |
| MsG0180000577.01 | 1.002724 | 0.002519 | 0.027462 | Chr1 | 8040671 | 8045064 | 1833 |
| novel.3253 | -5.55064 | 0.00252 | 0.027464 | Chr4 | 5811233 | 5811714 | 482 |
| MsG0580025789.01 | 1.49195 | 0.002521 | 0.027468 | Chr5 | 24162804 | 24164012 | 615 |
| MsG0380012255.01 | -2.98317 | 0.002526 | 0.027502 | Chr3 | 13439351 | 13442957 | 2457 |
| novel.7724 | 2.343782 | 0.002527 | 0.027502 | Chr7 | 87106407 | 87112599 | 2694 |
| MsG0480019038.01 | -1.9196 | 0.002528 | 0.027503 | Chr4 | 13641687 | 13647286 | 720 |
| MsG0580027692.01 | -5.7322 | 0.00253 | 0.027507 | Chr5 | 66261541 | 66263590 | 1680 |
| MsG0480023656.01 | -1.83793 | 0.00253 | 0.027507 | Chr4 | 88144802 | 88146211 | 1410 |
| MsG0680030731.01 | 1.017071 | 0.002531 | 0.027507 | Chr6 | 8184271 | 8191709 | 1260 |
| MsG0880044983.01 | -1.71922 | 0.002544 | 0.027614 | Chr8 | 52409853 | 52416183 | 1318 |
| MsG0880044106.01 | -2.25752 | 0.00255 | 0.027657 | Chr8 | 35636636 | 35641269 | 1227 |
| MsG0280009860.01 | -4.18649 | 0.00255 | 0.027657 | Chr2 | 59489631 | 59493762 | 1985 |
| MsG0580027767.01 | -1.02169 | 0.002552 | 0.027661 | Chr5 | 68250626 | 68251570 | 945 |
| MsG0780036061.01 | 1.030705 | 0.002565 | 0.027785 | Chr7 | 1968433 | 1969059 | 627 |
| novel.3967 | 2.678556 | 0.002565 | 0.027785 | Chr4 | 47764712 | 47766561 | 1293 |
| novel.141 | -4.27232 | 0.002566 | 0.027787 | Chr1 | 18613461 | 18615630 | 986 |
| MsG0380013810.01 | -1.93234 | 0.002567 | 0.02779 | Chr3 | 46007590 | 46009815 | 2226 |
| MsG0780038193.01 | 1.783589 | 0.002569 | 0.027802 | Chr7 | 41898642 | 41917037 | 2613 |
| MsG0180001726.01 | -2.81377 | 0.002574 | 0.027844 | Chr1 | 26089245 | 26092713 | 957 |
| MsG0480018473.01 | -1.58209 | 0.002575 | 0.027844 | Chr4 | 5474173 | 5479844 | 1026 |
| MsG0880045095.01 | 1.325743 | 0.002579 | 0.027882 | Chr8 | 54139428 | 54142257 | 1661 |
| MsG0280007246.01 | -1.25251 | 0.002588 | 0.027956 | Chr2 | 12476113 | 12488609 | 2353 |
| novel.5228 | 2.543978 | 0.002592 | 0.027986 | Chr5 | 66674106 | 66680206 | 3365 |
| MsG0180001492.01 | -2.91238 | 0.002592 | 0.027986 | Chr1 | 22197079 | 22206836 | 1493 |
| novel.1502 | -1.42189 | 0.002596 | 0.028008 | Chr2 | 64789864 | 64821025 | 1498 |
| novel.1930 | -2.34339 | 0.002596 | 0.028008 | Chr2 | 54842097 | 54846670 | 4574 |
| novel.9320 | -6.1196 | 0.0026 | 0.028027 | contig61end | 21813 | 26711 | 951 |
| MsG0780036514.01 | -1.95574 | 0.002601 | 0.028031 | Chr7 | 9121567 | 9124214 | 1453 |
| novel.294 | -3.79327 | 0.002609 | 0.028099 | Chr1 | 55247536 | 55251163 | 1625 |
| MsG0280010587.01 | -1.70465 | 0.002609 | 0.028099 | Chr2 | 71809645 | 71814024 | 2236 |
| novel.2887 | 1.241396 | 0.002613 | 0.028131 | Chr3 | 45449679 | 45456225 | 2749 |
| novel.6772 | -5.65478 | 0.002622 | 0.028219 | Chr6 | 1.1E+08 | 1.1E+08 | 688 |
| MsG0380014690.01 | -4.53585 | 0.002625 | 0.028227 | Chr3 | 58533871 | 58542064 | 1353 |
| MsG0580029146.01 | -1.15335 | 0.002626 | 0.028227 | Chr5 | 92889182 | 92895332 | 2816 |
| MsG0380014125.01 | -2.07644 | 0.002644 | 0.028403 | Chr3 | 50157554 | 50161136 | 552 |
| MsG0480021367.01 | -2.15965 | 0.002668 | 0.028653 | Chr4 | 57117244 | 57143166 | 1481 |
| novel.4729 | -1.18577 | 0.002672 | 0.028686 | Chr5 | 82826704 | 82846106 | 2696 |
| MsG0580030070.01 | -1.1377 | 0.002688 | 0.028849 | Chr5 | 1.06E+08 | 1.06E+08 | 1374 |
| MsG0380014890.01 | 1.675383 | 0.0027 | 0.028956 | Chr3 | 62100726 | 62105850 | 1570 |
| novel.4811 | -5.82224 | 0.002703 | 0.028969 | Chr5 | 97104044 | 97107163 | 898 |
| MsG0880044764.01 | -2.06361 | 0.002703 | 0.028969 | Chr8 | 48778775 | 48781391 | 1191 |
| novel.6570 | -1.54745 | 0.002706 | 0.028983 | Chr6 | 75369826 | 75374268 | 2094 |
| MsG0280010676.01 | -1.95748 | 0.002707 | 0.028991 | Chr2 | 73206697 | 73206969 | 273 |
| MsG0880042425.01 | -2.47836 | 0.002709 | 0.028998 | Chr8 | 8511950 | 8520449 | 2514 |
| novel.2521 | 1.52563 | 0.002713 | 0.029029 | Chr3 | 82953130 | 82954023 | 894 |
| novel.2969 | 1.51892 | 0.002721 | 0.029095 | Chr3 | 66343842 | 66346027 | 1727 |
| MsG0180000661.01 | -5.54524 | 0.002724 | 0.029115 | Chr1 | 9387830 | 9389472 | 540 |
| MsG0480018433.01 | -3.06242 | 0.002728 | 0.029143 | Chr4 | 4813230 | 4821428 | 4127 |
| MsG0780036661.01 | -1.24679 | 0.002729 | 0.029143 | Chr7 | 11493770 | 11499668 | 1741 |
| MsG0280009854.01 | -2.84194 | 0.002729 | 0.029143 | Chr2 | 59428424 | 59434319 | 1620 |
| novel.7536 | 3.563119 | 0.002742 | 0.029266 | Chr7 | 58129116 | 58129594 | 414 |
| MsG0280007316.01 | 4.119913 | 0.002747 | 0.029317 | Chr2 | 13502533 | 13518160 | 2895 |
| novel.1392 | -2.45732 | 0.002763 | 0.029461 | Chr2 | 37640097 | 37643545 | 3449 |
| MsG0580025521.01 | 1.6284 | 0.002765 | 0.029469 | Chr5 | 19954972 | 19958090 | 1163 |
| novel.1917 | -1.52505 | 0.002768 | 0.029491 | Chr2 | 51924747 | 51925717 | 442 |
| novel.6692 | -5.4016 | 0.002769 | 0.029491 | Chr6 | 1.02E+08 | 1.02E+08 | 626 |
| MsG0880043447.01 | -4.6022 | 0.00277 | 0.029491 | Chr8 | 24961874 | 24964485 | 1041 |
| MsG0180001867.01 | 1.096217 | 0.002776 | 0.029534 | Chr1 | 28412384 | 28412620 | 237 |
| novel.646 | 1.764811 | 0.002776 | 0.029534 | Chr1 | 6792157 | 6792856 | 345 |
| MsG0880042877.01 | -2.43234 | 0.002785 | 0.029606 | Chr8 | 15295872 | 15299039 | 900 |
| MsG0780041056.01 | -1.24925 | 0.002796 | 0.02971 | Chr7 | 85938087 | 85938733 | 454 |
| novel.9101 | -1.36349 | 0.002797 | 0.02971 | contig484end | 9812 | 15314 | 3357 |
| MsG0080048247.01 | -4.11457 | 0.002798 | 0.029712 | contig252end | 20076 | 23710 | 1196 |
| novel.1915 | 1.165775 | 0.002803 | 0.029752 | Chr2 | 51728003 | 51730991 | 1251 |
| MsG0780040311.01 | -2.61752 | 0.002816 | 0.029873 | Chr7 | 75791132 | 75797509 | 4572 |
| MsG0480018222.01 | -2.13946 | 0.00282 | 0.029903 | Chr4 | 1769277 | 1769660 | 384 |
| MsG0280008459.01 | 5.654052 | 0.002822 | 0.029917 | Chr2 | 32235526 | 32237534 | 1788 |
| MsG0380012838.01 | 5.529153 | 0.002825 | 0.02992 | Chr3 | 25472681 | 25473196 | 516 |
| MsG0880042683.01 | -1.8078 | 0.002825 | 0.02992 | Chr8 | 12600578 | 12605016 | 594 |
| MsG0180001594.01 | 1.578798 | 0.002832 | 0.029975 | Chr1 | 23863989 | 23868435 | 3273 |
| novel.5505 | -4.25656 | 0.002833 | 0.029981 | Chr6 | 641498 | 642515 | 696 |
| novel.3505 | -2.93391 | 0.002838 | 0.030017 | Chr4 | 57191008 | 57193640 | 908 |
| MsG0580025833.01 | -1.73256 | 0.002844 | 0.030071 | Chr5 | 24864402 | 24865562 | 1161 |
| novel.6683 | 2.171236 | 0.002845 | 0.030071 | Chr6 | 1E+08 | 1E+08 | 384 |
| MsG0280008747.01 | -2.09729 | 0.00285 | 0.030118 | Chr2 | 37618333 | 37625190 | 3504 |
| novel.984 | -1.52375 | 0.002856 | 0.030166 | Chr1 | 78319655 | 78325217 | 1248 |
| novel.5235 | 5.651181 | 0.002862 | 0.030216 | Chr5 | 68299935 | 68301113 | 1052 |
| MsG0480020318.01 | 1.673219 | 0.002864 | 0.030216 | Chr4 | 38830657 | 38834869 | 1198 |
| MsG0780041680.01 | 2.590764 | 0.002865 | 0.030216 | Chr7 | 93845883 | 93856416 | 1914 |
| novel.1074 | -2.51005 | 0.002865 | 0.030216 | Chr1 | 91072691 | 91075316 | 1801 |
| MsG0580028671.01 | -1.09643 | 0.002875 | 0.030297 | Chr5 | 84577982 | 84578749 | 768 |
| MsG0180005229.01 | 1.047026 | 0.002876 | 0.030301 | Chr1 | 88878369 | 88884038 | 1425 |
| novel.620 | 2.919867 | 0.002879 | 0.030323 | Chr1 | 3436800 | 3443169 | 3017 |
| novel.4359 | -1.01055 | 0.002883 | 0.030352 | Chr5 | 11851852 | 11862800 | 10949 |
| MsG0480019232.01 | -3.97369 | 0.002885 | 0.030361 | Chr4 | 16657224 | 16666989 | 1391 |
| MsG0780036978.01 | 1.197621 | 0.002896 | 0.030468 | Chr7 | 17448241 | 17451895 | 744 |
| MsG0280010622.01 | -2.21183 | 0.002903 | 0.030517 | Chr2 | 72427012 | 72427318 | 307 |
| novel.7801 | -1.49031 | 0.002909 | 0.030571 | Chr8 | 2087257 | 2089736 | 1376 |
| novel.1105 | 2.07092 | 0.002913 | 0.030604 | Chr1 | 95757167 | 95758676 | 981 |
| MsG0580026281.01 | -2.64038 | 0.002918 | 0.030644 | Chr5 | 33916087 | 33919544 | 934 |
| novel.1734 | -3.07713 | 0.002923 | 0.030689 | Chr2 | 12397405 | 12399829 | 1409 |
| novel.1812 | 5.499467 | 0.002925 | 0.030698 | Chr2 | 26264411 | 26265620 | 535 |
| novel.3978 | 1.97878 | 0.002932 | 0.030761 | Chr4 | 49864971 | 49869014 | 3596 |
| MsG0880042130.01 | -1.90809 | 0.002939 | 0.030816 | Chr8 | 4169596 | 4172096 | 1215 |
| MsG0380013312.01 | 1.567116 | 0.002939 | 0.030816 | Chr3 | 34937766 | 34941593 | 2816 |
| MsG0580024705.01 | -1.89476 | 0.002951 | 0.030889 | Chr5 | 8526342 | 8531526 | 943 |
| MsG0680031239.01 | -2.86719 | 0.002952 | 0.030889 | Chr6 | 16525331 | 16527023 | 707 |
| novel.8362 | -4.99032 | 0.002952 | 0.030889 | Chr8 | 10975234 | 10975681 | 448 |
| MsG0180000653.01 | 1.535962 | 0.00296 | 0.030951 | Chr1 | 9262762 | 9264791 | 1551 |
| MsG0880045341.01 | -1.95665 | 0.002971 | 0.031051 | Chr8 | 57797590 | 57805099 | 2409 |
| novel.669 | -1.11122 | 0.002974 | 0.031076 | Chr1 | 9837798 | 9838777 | 980 |
| novel.6519 | 5.561092 | 0.002977 | 0.031097 | Chr6 | 66607874 | 66609535 | 515 |
| MsG0480021867.01 | 1.762451 | 0.002983 | 0.031144 | Chr4 | 65361041 | 65363730 | 1506 |
| novel.3352 | -4.77514 | 0.002995 | 0.031257 | Chr4 | 24574197 | 24576360 | 1125 |
| MsG0680034890.01 | -2.87399 | 0.002996 | 0.031257 | Chr6 | 96299744 | 96301098 | 740 |
| MsG0780036097.01 | 2.530957 | 0.002997 | 0.031259 | Chr7 | 2505453 | 2514474 | 1247 |
| novel.2877 | -4.45725 | 0.003 | 0.031267 | Chr3 | 40125942 | 40128583 | 581 |
| novel.2920 | -3.30906 | 0.003003 | 0.031287 | Chr3 | 52161556 | 52164032 | 815 |
| novel.4876 | -1.71581 | 0.003005 | 0.031305 | Chr5 | 1.05E+08 | 1.05E+08 | 1598 |
| MsG0880047717.01 | 1.101297 | 0.003011 | 0.031357 | Chr8 | 89959877 | 89961499 | 1623 |
| MsG0480023521.01 | -2.06206 | 0.003016 | 0.031397 | Chr4 | 86434769 | 86439671 | 1410 |
| novel.7434 | 2.440747 | 0.003025 | 0.031472 | Chr7 | 31605754 | 31614975 | 1532 |
| MsG0380012400.01 | -2.70893 | 0.003026 | 0.031473 | Chr3 | 16008610 | 16010662 | 1446 |
| novel.6964 | -5.98511 | 0.003032 | 0.031531 | Chr7 | 37192111 | 37192917 | 585 |
| MsG0680031468.01 | 4.513335 | 0.003034 | 0.031533 | Chr6 | 20872569 | 20873659 | 597 |
| MsG0480020388.01 | -3.68425 | 0.003035 | 0.031533 | Chr4 | 40267708 | 40272013 | 1545 |
| MsG0680032726.01 | -1.9556 | 0.003036 | 0.031533 | Chr6 | 48294314 | 48296777 | 1818 |
| MsG0180004155.01 | 1.15117 | 0.003046 | 0.031624 | Chr1 | 74122990 | 74127616 | 951 |
| MsG0880046603.01 | 1.452343 | 0.003046 | 0.031624 | Chr8 | 75383249 | 75386964 | 1676 |
| novel.886 | 1.048921 | 0.003061 | 0.031757 | Chr1 | 55801020 | 55806735 | 674 |
| MsG0380014941.01 | 1.731361 | 0.003067 | 0.031809 | Chr3 | 62824359 | 62825054 | 696 |
| MsG0580028370.01 | 6.709552 | 0.003085 | 0.03195 | Chr5 | 79243943 | 79244326 | 384 |
| novel.1596 | 5.636281 | 0.003085 | 0.03195 | Chr2 | 79761771 | 79767468 | 427 |
| novel.1912 | 1.038537 | 0.00309 | 0.031988 | Chr2 | 51287344 | 51289237 | 866 |
| MsG0680030979.01 | -2.79726 | 0.0031 | 0.032079 | Chr6 | 12406903 | 12411217 | 3081 |
| MsG0480020450.01 | 1.907796 | 0.003105 | 0.032118 | Chr4 | 41714540 | 41718381 | 3195 |
| MsG0680030989.01 | 1.24763 | 0.003113 | 0.032195 | Chr6 | 12581705 | 12587209 | 3321 |
| MsG0380015693.01 | -1.99041 | 0.003122 | 0.032273 | Chr3 | 73808819 | 73813705 | 1969 |
| novel.4656 | -3.67861 | 0.003144 | 0.032458 | Chr5 | 70486590 | 70490210 | 1054 |
| MsG0480018353.01 | 1.284202 | 0.003147 | 0.032476 | Chr4 | 3695134 | 3698049 | 525 |
| novel.7614 | 3.185428 | 0.003155 | 0.032539 | Chr7 | 70612631 | 70613608 | 978 |
| MsG0580027236.01 | -1.47596 | 0.003156 | 0.032539 | Chr5 | 56828058 | 56831153 | 1657 |
| MsG0180001775.01 | -2.57408 | 0.003156 | 0.032539 | Chr1 | 26745048 | 26751946 | 1152 |
| novel.557 | -3.58487 | 0.003167 | 0.032636 | Chr1 | 98518727 | 98520118 | 1257 |
| novel.1777 | 2.263192 | 0.00318 | 0.032736 | Chr2 | 18802710 | 18806356 | 3647 |
| MsG0280007866.01 | 1.286264 | 0.003182 | 0.032736 | Chr2 | 22031040 | 22035623 | 1020 |
| MsG0280009909.01 | -1.05219 | 0.003182 | 0.032736 | Chr2 | 60620434 | 60625723 | 2370 |
| novel.3282 | -5.51863 | 0.003182 | 0.032736 | Chr4 | 10783684 | 10785120 | 1437 |
| novel.5966 | -2.29533 | 0.003189 | 0.032796 | Chr6 | 91083366 | 91085504 | 1205 |
| MsG0880045155.01 | -1.79844 | 0.003191 | 0.032807 | Chr8 | 55009166 | 55009553 | 303 |
| novel.4427 | 4.381713 | 0.003193 | 0.032814 | Chr5 | 23717123 | 23718114 | 554 |
| MsG0580025512.01 | -2.2276 | 0.003197 | 0.032846 | Chr5 | 19849419 | 19850549 | 1131 |
| novel.3035 | -4.0318 | 0.003198 | 0.032846 | Chr3 | 77466585 | 77478344 | 364 |
| MsG0480020084.01 | 4.195965 | 0.003207 | 0.032919 | Chr4 | 33690486 | 33690860 | 375 |
| MsG0480018116.01 | -2.81786 | 0.003211 | 0.032947 | Chr4 | 378657 | 380680 | 1308 |
| MsG0280010720.01 | -2.55397 | 0.003227 | 0.033105 | Chr2 | 73882717 | 73884834 | 1007 |
| novel.6220 | -5.2065 | 0.003235 | 0.033173 | Chr6 | 12378565 | 12379964 | 1400 |
| novel.7866 | -11.5029 | 0.003238 | 0.033192 | Chr8 | 12213657 | 12216206 | 1791 |
| MsG0580025174.01 | 5.601216 | 0.003239 | 0.033192 | Chr5 | 14747223 | 14748095 | 873 |
| MsG0680034842.01 | -2.80927 | 0.003246 | 0.033254 | Chr6 | 95411195 | 95418499 | 2844 |
| MsG0180001724.01 | -3.45682 | 0.003248 | 0.033268 | Chr1 | 26071057 | 26071290 | 234 |
| MsG0780036862.01 | -3.68559 | 0.003254 | 0.033302 | Chr7 | 15198981 | 15203152 | 850 |
| MsG0580029001.01 | 1.404655 | 0.003271 | 0.03347 | Chr5 | 90134994 | 90139314 | 1567 |
| MsG0880041833.01 | 1.354193 | 0.003277 | 0.033502 | Chr8 | 10319 | 10846 | 528 |
| novel.4666 | -2.88153 | 0.003284 | 0.033569 | Chr5 | 72388228 | 72391468 | 1571 |
| MsG0280009201.01 | 5.519564 | 0.003291 | 0.033628 | Chr2 | 47602337 | 47604378 | 858 |
| novel.47 | -1.47281 | 0.003298 | 0.033684 | Chr1 | 6023558 | 6027779 | 2213 |
| MsG0880046203.01 | -1.06134 | 0.0033 | 0.033698 | Chr8 | 70500884 | 70502849 | 1236 |
| novel.7563 | 3.297039 | 0.003307 | 0.033748 | Chr7 | 64045322 | 64047933 | 626 |
| novel.6210 | -5.86769 | 0.003307 | 0.033748 | Chr6 | 11043859 | 11045400 | 1318 |
| MsG0380012211.01 | 2.652179 | 0.003315 | 0.033811 | Chr3 | 12765267 | 12773685 | 2757 |
| novel.5608 | -5.48161 | 0.003318 | 0.033825 | Chr6 | 17040946 | 17043962 | 2927 |
| MsG0780039243.01 | 1.019237 | 0.003319 | 0.033825 | Chr7 | 60561759 | 60563122 | 675 |
| MsG0180005996.01 | -2.07252 | 0.003319 | 0.033825 | Chr1 | 98610109 | 98611770 | 1662 |
| MsG0880044341.01 | -1.59069 | 0.003322 | 0.033839 | Chr8 | 40313884 | 40317882 | 1458 |
| novel.8542 | -6.52574 | 0.003327 | 0.033876 | Chr8 | 50854351 | 50857709 | 728 |
| novel.8943 | 5.509948 | 0.003329 | 0.033891 | contig338end | 31365 | 32138 | 678 |
| novel.3007 | -3.88986 | 0.003352 | 0.034103 | Chr3 | 72573863 | 72584365 | 2789 |
| MsG0380017819.01 | -1.41439 | 0.003362 | 0.03419 | Chr3 | 1.01E+08 | 1.01E+08 | 843 |
| MsG0080048243.01 | -3.80404 | 0.00337 | 0.034266 | contig252end | 2028 | 5207 | 498 |
| novel.1897 | -5.10918 | 0.003375 | 0.034281 | Chr2 | 45975263 | 45976075 | 454 |
| MsG0680034233.01 | -1.13232 | 0.003375 | 0.034281 | Chr6 | 84183182 | 84187969 | 1236 |
| novel.5898 | 2.151035 | 0.003383 | 0.034349 | Chr6 | 78311703 | 78314968 | 809 |
| MsG0880042200.01 | -2.65077 | 0.00339 | 0.034401 | Chr8 | 5275586 | 5276792 | 1089 |
| MsG0180004164.01 | 1.189406 | 0.00339 | 0.034401 | Chr1 | 74281802 | 74287578 | 2025 |
| MsG0580029725.01 | -4.34764 | 0.003395 | 0.034435 | Chr5 | 1.01E+08 | 1.01E+08 | 2937 |
| MsG0880045327.01 | 1.187448 | 0.003397 | 0.034443 | Chr8 | 57529354 | 57529973 | 255 |
| MsG0680032613.01 | 5.676653 | 0.003405 | 0.034505 | Chr6 | 45970913 | 45974665 | 1446 |
| MsG0580025893.01 | -3.42899 | 0.003406 | 0.034505 | Chr5 | 26280453 | 26281798 | 739 |
| MsG0180004879.01 | -1.54347 | 0.003411 | 0.034543 | Chr1 | 83906674 | 83908092 | 1053 |
| MsG0280011270.01 | -1.32664 | 0.003418 | 0.034598 | Chr2 | 81771798 | 81772388 | 591 |
| MsG0780036792.01 | 1.0463 | 0.00343 | 0.034706 | Chr7 | 13921221 | 13924747 | 942 |
| novel.2240 | -6.48626 | 0.003437 | 0.034754 | Chr3 | 18908505 | 18909895 | 1391 |
| MsG0380015369.01 | -1.48874 | 0.003443 | 0.034796 | Chr3 | 69139413 | 69143347 | 2111 |
| MsG0580029455.01 | -5.54332 | 0.003446 | 0.034816 | Chr5 | 97133356 | 97136181 | 399 |
| MsG0180004517.01 | -5.36534 | 0.003455 | 0.034885 | Chr1 | 79056182 | 79061015 | 756 |
| MsG0580027191.01 | -1.71745 | 0.003457 | 0.03489 | Chr5 | 55893038 | 55895689 | 1767 |
| novel.5088 | -3.30572 | 0.003469 | 0.034997 | Chr5 | 34349669 | 34351921 | 1949 |
| MsG0780037292.01 | -1.89788 | 0.003472 | 0.034997 | Chr7 | 23208228 | 23210111 | 822 |
| novel.2510 | 1.375637 | 0.003473 | 0.034997 | Chr3 | 79429585 | 79430841 | 794 |
| MsG0480023386.01 | 1.003496 | 0.003473 | 0.034997 | Chr4 | 84501312 | 84503454 | 898 |
| novel.126 | -1.64401 | 0.003476 | 0.03501 | Chr1 | 16421765 | 16426625 | 2720 |
| novel.7503 | -5.61083 | 0.003477 | 0.03501 | Chr7 | 47885149 | 47886665 | 401 |
| MsG0380012322.01 | -2.98357 | 0.003487 | 0.035105 | Chr3 | 14865704 | 14873378 | 4533 |
| novel.4503 | 3.148612 | 0.003496 | 0.035176 | Chr5 | 36320388 | 36322445 | 1227 |
| MsG0580028895.01 | 1.065884 | 0.003502 | 0.035226 | Chr5 | 88235312 | 88244323 | 1605 |
| novel.286 | -1.80055 | 0.003503 | 0.035231 | Chr1 | 53065399 | 53067040 | 1336 |
| novel.1987 | -2.59818 | 0.003518 | 0.035355 | Chr2 | 66737893 | 66741601 | 2263 |
| MsG0580028840.01 | -1.84847 | 0.00352 | 0.035365 | Chr5 | 87356140 | 87366545 | 2847 |
| MsG0880042762.01 | -2.42494 | 0.003525 | 0.035394 | Chr8 | 13687300 | 13690067 | 1409 |
| novel.3844 | 2.888069 | 0.003526 | 0.035394 | Chr4 | 18268186 | 18269778 | 1276 |
| novel.5817 | -5.44911 | 0.003527 | 0.035394 | Chr6 | 62024341 | 62025817 | 764 |
| MsG0180002376.01 | 2.456487 | 0.00354 | 0.0355 | Chr1 | 37653612 | 37655738 | 2127 |
| MsG0880044578.01 | -3.65639 | 0.00354 | 0.0355 | Chr8 | 44924085 | 44924321 | 237 |
| MsG0780036078.01 | 1.108006 | 0.003542 | 0.0355 | Chr7 | 2126734 | 2132147 | 1754 |
| novel.1578 | 2.791775 | 0.003542 | 0.0355 | Chr2 | 77972622 | 77975465 | 2601 |
| MsG0880043280.01 | 1.680216 | 0.003544 | 0.035516 | Chr8 | 21914696 | 21915232 | 537 |
| MsG0580026876.01 | -6.81894 | 0.003547 | 0.035525 | Chr5 | 47089939 | 47090598 | 660 |
| novel.2935 | -2.65007 | 0.00356 | 0.035622 | Chr3 | 57338861 | 57344157 | 1321 |
| MsG0680034110.01 | -3.74536 | 0.003589 | 0.035902 | Chr6 | 80770178 | 80772275 | 546 |
| MsG0480019381.01 | -2.7025 | 0.003591 | 0.035912 | Chr4 | 19321957 | 19328007 | 1473 |
| MsG0680035265.01 | -1.96679 | 0.003594 | 0.035919 | Chr6 | 1.01E+08 | 1.01E+08 | 1581 |
| novel.7973 | 2.838237 | 0.003596 | 0.035919 | Chr8 | 34887402 | 34889893 | 910 |
| novel.8905 | -2.23457 | 0.003596 | 0.035919 | contig271end | 131970 | 133739 | 1164 |
| novel.3128 | -5.49341 | 0.003605 | 0.035974 | Chr3 | 91197720 | 91198863 | 692 |
| novel.4005 | -4.46244 | 0.003606 | 0.035974 | Chr4 | 55318478 | 55320165 | 1085 |
| novel.199 | -5.64967 | 0.003606 | 0.035974 | Chr1 | 28147361 | 28148143 | 683 |
| MsG0380015681.01 | -1.49388 | 0.003607 | 0.035974 | Chr3 | 73685145 | 73693671 | 651 |
| MsG0180003264.01 | -1.29128 | 0.003608 | 0.035974 | Chr1 | 59465701 | 59471808 | 1839 |
| MsG0580025366.01 | -1.36443 | 0.003609 | 0.035978 | Chr5 | 17538769 | 17539876 | 922 |
| MsG0480022951.01 | 5.644043 | 0.00362 | 0.036075 | Chr4 | 78969924 | 78977581 | 1941 |
| MsG0580029467.01 | 2.297803 | 0.003642 | 0.036225 | Chr5 | 97307216 | 97310263 | 1762 |
| novel.2292 | 2.71217 | 0.003643 | 0.036225 | Chr3 | 29977858 | 29978909 | 1052 |
| MsG0480018101.01 | -3.00671 | 0.003644 | 0.036225 | Chr4 | 255260 | 260301 | 1726 |
| novel.9086 | 1.719604 | 0.00365 | 0.036254 | contig473end | 34127 | 36968 | 923 |
| MsG0880043292.01 | -1.62616 | 0.003654 | 0.036283 | Chr8 | 22120127 | 22124749 | 1296 |
| novel.320 | -4.39933 | 0.003657 | 0.036298 | Chr1 | 60093341 | 60094199 | 542 |
| novel.8487 | -2.11254 | 0.003671 | 0.036411 | Chr8 | 37670898 | 37673175 | 906 |
| MsG0680030990.01 | 1.464724 | 0.003672 | 0.036412 | Chr6 | 12599742 | 12609930 | 4668 |
| MsG0680035715.01 | -1.64949 | 0.003674 | 0.036423 | Chr6 | 1.11E+08 | 1.11E+08 | 1398 |
| MsG0780036635.01 | -1.37182 | 0.003681 | 0.036462 | Chr7 | 10991051 | 10999032 | 3699 |
| novel.1286 | -1.93204 | 0.003691 | 0.036543 | Chr2 | 19350004 | 19352816 | 2732 |
| novel.8883 | -1.08798 | 0.003694 | 0.036559 | contig246end | 117530 | 122687 | 2468 |
| novel.827 | -2.05703 | 0.003701 | 0.036617 | Chr1 | 36956485 | 36958368 | 1642 |
| novel.7016 | -2.31314 | 0.003706 | 0.036648 | Chr7 | 49737791 | 49744303 | 6433 |
| novel.8025 | -4.06415 | 0.003707 | 0.036649 | Chr8 | 44957058 | 44958617 | 820 |
| MsG0080048672.01 | -2.46588 | 0.00371 | 0.036673 | contig399end | 13289 | 13692 | 372 |
| novel.289 | -3.01464 | 0.003721 | 0.036728 | Chr1 | 54189184 | 54192803 | 1482 |
| novel.8140 | -5.83605 | 0.003722 | 0.036728 | Chr8 | 64887246 | 64889412 | 722 |
| MsG0280010668.01 | -2.60654 | 0.003722 | 0.036728 | Chr2 | 73141020 | 73141277 | 258 |
| MsG0780037808.01 | 1.372266 | 0.003724 | 0.036732 | Chr7 | 33551791 | 33553167 | 1377 |
| MsG0780041265.01 | -3.33161 | 0.003729 | 0.036773 | Chr7 | 88934300 | 88934611 | 312 |
| MsG0580028720.01 | 1.842061 | 0.003731 | 0.036782 | Chr5 | 85327714 | 85333333 | 1128 |
| MsG0480021011.01 | -1.45556 | 0.003734 | 0.036798 | Chr4 | 52030238 | 52049466 | 7860 |
| MsG0680035458.01 | 1.232645 | 0.003739 | 0.036818 | Chr6 | 1.06E+08 | 1.06E+08 | 1056 |
| novel.8872 | 3.917254 | 0.00374 | 0.036818 | contig238end | 22254 | 24077 | 1588 |
| novel.7822 | -3.63271 | 0.003741 | 0.036818 | Chr8 | 5290572 | 5294095 | 790 |
| novel.6916 | 5.465748 | 0.003761 | 0.036974 | Chr7 | 26658235 | 26658745 | 511 |
| MsG0180004435.01 | 1.518282 | 0.003761 | 0.036974 | Chr1 | 77895420 | 77896636 | 756 |
| MsG0780037249.01 | 6.055638 | 0.003766 | 0.037011 | Chr7 | 22448329 | 22449604 | 876 |
| MsG0780036611.01 | 1.436474 | 0.003776 | 0.037096 | Chr7 | 10592720 | 10599972 | 1765 |
| novel.7066 | 5.460216 | 0.003786 | 0.037175 | Chr7 | 62395093 | 62395557 | 369 |
| MsG0780040785.01 | -6.53039 | 0.00379 | 0.037188 | Chr7 | 82410949 | 82419317 | 4097 |
| MsG0680035825.01 | -3.61094 | 0.00379 | 0.037188 | Chr6 | 1.13E+08 | 1.13E+08 | 816 |
| novel.8688 | 4.485965 | 0.003802 | 0.037285 | Chr8 | 74836207 | 74837657 | 781 |
| MsG0780041533.01 | 1.058845 | 0.003809 | 0.037344 | Chr7 | 92099509 | 92099829 | 321 |
| MsG0780037700.01 | -2.46824 | 0.003821 | 0.037426 | Chr7 | 31457787 | 31459378 | 1011 |
| novel.4631 | 3.52239 | 0.003822 | 0.037428 | Chr5 | 64035820 | 64039911 | 1509 |
| novel.247 | 1.019588 | 0.003829 | 0.037478 | Chr1 | 38417022 | 38418830 | 718 |
| MsG0280007312.01 | -1.17025 | 0.00383 | 0.037478 | Chr2 | 13463480 | 13470697 | 2072 |
| MsG0280007862.01 | -3.94307 | 0.00384 | 0.037557 | Chr2 | 21939863 | 21941653 | 1791 |
| MsG0580026061.01 | -1.09456 | 0.00385 | 0.037644 | Chr5 | 30044935 | 30047586 | 1406 |
| novel.3915 | 1.94691 | 0.003859 | 0.037713 | Chr4 | 33342752 | 33342969 | 218 |
| MsG0680030336.01 | -3.63214 | 0.003863 | 0.037738 | Chr6 | 790329 | 792768 | 945 |
| novel.6926 | 2.282592 | 0.003871 | 0.037793 | Chr7 | 28979216 | 28983239 | 929 |
| MsG0480021141.01 | -1.17848 | 0.003871 | 0.037793 | Chr4 | 54237159 | 54249936 | 1608 |
| MsG0180001806.01 | 1.736676 | 0.003874 | 0.037812 | Chr1 | 27334227 | 27335207 | 981 |
| MsG0180000331.01 | -1.65465 | 0.003876 | 0.037812 | Chr1 | 4532837 | 4534875 | 331 |
| MsG0580025323.01 | 1.426312 | 0.003883 | 0.037867 | Chr5 | 16939463 | 16939720 | 258 |
| MsG0080047911.01 | -1.91288 | 0.003885 | 0.037871 | contig142end | 53153 | 59393 | 2139 |
| novel.1811 | 3.690584 | 0.003901 | 0.038002 | Chr2 | 26231705 | 26256876 | 989 |
| MsG0580027471.01 | 3.333508 | 0.003906 | 0.038041 | Chr5 | 61702529 | 61706454 | 1285 |
| MsG0180001066.01 | -2.1545 | 0.003914 | 0.038102 | Chr1 | 15444787 | 15449557 | 532 |
| MsG0680034234.01 | -1.38425 | 0.003915 | 0.038102 | Chr6 | 84190754 | 84193945 | 511 |
| novel.3071 | 3.243745 | 0.003926 | 0.038203 | Chr3 | 81588700 | 81594761 | 2026 |
| novel.7571 | 1.292127 | 0.003928 | 0.038203 | Chr7 | 65270732 | 65275795 | 4422 |
| MsG0780036272.01 | 1.779127 | 0.00393 | 0.03821 | Chr7 | 5758402 | 5765444 | 3955 |
| MsG0580027986.01 | -6.44527 | 0.003931 | 0.03821 | Chr5 | 72459178 | 72459561 | 384 |
| MsG0480021753.01 | -1.76849 | 0.003938 | 0.038266 | Chr4 | 63880621 | 63884805 | 2045 |
| MsG0180003730.01 | -1.72099 | 0.003942 | 0.038268 | Chr1 | 67382213 | 67386144 | 1402 |
| novel.8474 | 3.308309 | 0.003942 | 0.038268 | Chr8 | 34400336 | 34402816 | 724 |
| novel.4685 | -6.49775 | 0.003942 | 0.038268 | Chr5 | 74774901 | 74780889 | 2048 |
| novel.4610 | -2.11721 | 0.003948 | 0.038295 | Chr5 | 59223905 | 59225624 | 1445 |
| MsG0780036896.01 | -3.11535 | 0.003954 | 0.038342 | Chr7 | 15868506 | 15871392 | 615 |
| MsG0580024862.01 | 1.244869 | 0.003964 | 0.038423 | Chr5 | 10960399 | 10963271 | 886 |
| novel.5107 | 1.489428 | 0.003971 | 0.038462 | Chr5 | 38890418 | 38892232 | 1138 |
| MsG0880046299.01 | 1.620608 | 0.003972 | 0.038462 | Chr8 | 71661204 | 71662063 | 780 |
| MsG0480019339.01 | -2.31213 | 0.003979 | 0.038514 | Chr4 | 18636570 | 18643338 | 2913 |
| MsG0280010812.01 | 3.126862 | 0.003994 | 0.038652 | Chr2 | 75359555 | 75360073 | 519 |
| novel.7164 | -2.21446 | 0.004006 | 0.038752 | Chr7 | 77777282 | 77779845 | 1669 |
| novel.4439 | -1.60055 | 0.004012 | 0.038792 | Chr5 | 24620987 | 24623073 | 1284 |
| MsG0680032234.01 | 2.251714 | 0.004019 | 0.038843 | Chr6 | 37063230 | 37073882 | 1428 |
| MsG0780040308.01 | -3.39073 | 0.004028 | 0.038906 | Chr7 | 75759372 | 75761047 | 948 |
| MsG0280011079.01 | -1.32604 | 0.004028 | 0.038906 | Chr2 | 79204667 | 79206543 | 1253 |
| MsG0580024259.01 | 1.710758 | 0.004029 | 0.038906 | Chr5 | 2670301 | 2673011 | 1203 |
| MsG0180004703.01 | -1.18559 | 0.004031 | 0.038908 | Chr1 | 81556703 | 81561127 | 743 |
| MsG0480021189.01 | 2.027177 | 0.004033 | 0.038921 | Chr4 | 54884784 | 54885134 | 351 |
| novel.5490 | 1.028166 | 0.004037 | 0.038943 | Chr5 | 1.08E+08 | 1.08E+08 | 1012 |
| MsG0880046262.01 | 5.421243 | 0.004041 | 0.038973 | Chr8 | 71246696 | 71249031 | 621 |
| MsG0180000655.01 | 1.397155 | 0.004043 | 0.038973 | Chr1 | 9272688 | 9275184 | 735 |
| novel.4436 | 1.484965 | 0.004056 | 0.039086 | Chr5 | 24272098 | 24272798 | 346 |
| MsG0780038104.01 | -5.40369 | 0.004075 | 0.039262 | Chr7 | 40236104 | 40237001 | 756 |
| MsG0680035868.01 | -2.43008 | 0.00408 | 0.039281 | Chr6 | 1.14E+08 | 1.14E+08 | 1092 |
| novel.5972 | 3.381044 | 0.004081 | 0.039284 | Chr6 | 91945654 | 91950360 | 794 |
| MsG0280006490.01 | -1.02427 | 0.004086 | 0.039314 | Chr2 | 2682848 | 2689027 | 1023 |
| MsG0880047762.01 | 5.506263 | 0.004097 | 0.039408 | Chr8 | 90502781 | 90523816 | 4728 |
| MsG0380014180.01 | -4.28827 | 0.004109 | 0.039516 | Chr3 | 50946972 | 50950483 | 1181 |
| novel.1102 | 5.573366 | 0.00412 | 0.039607 | Chr1 | 95499661 | 95500340 | 614 |
| MsG0580028526.01 | 1.048833 | 0.004144 | 0.039827 | Chr5 | 81872286 | 81876857 | 3282 |
| novel.313 | 1.093397 | 0.00415 | 0.039857 | Chr1 | 58894660 | 58898000 | 1162 |
| MsG0380016192.01 | -1.43542 | 0.004151 | 0.039857 | Chr3 | 80204273 | 80205489 | 656 |
| MsG0480022309.01 | -1.89363 | 0.004154 | 0.039857 | Chr4 | 70980208 | 70982494 | 2033 |
| novel.3956 | -2.45755 | 0.004154 | 0.039857 | Chr4 | 44885167 | 44887639 | 1348 |
| novel.8541 | -3.75405 | 0.004155 | 0.039857 | Chr8 | 50723952 | 50733071 | 1569 |
| MsG0380012844.01 | 1.273155 | 0.004156 | 0.039857 | Chr3 | 25557743 | 25567238 | 5745 |
| novel.668 | -2.2672 | 0.004167 | 0.039926 | Chr1 | 9616626 | 9618193 | 882 |
| MsG0180000526.01 | -1.54659 | 0.004168 | 0.039926 | Chr1 | 7425287 | 7426630 | 756 |
| MsG0180005755.01 | -1.33286 | 0.004171 | 0.039947 | Chr1 | 95723328 | 95728285 | 1483 |
| MsG0680034516.01 | 2.826372 | 0.004175 | 0.039954 | Chr6 | 89466165 | 89466936 | 387 |
| novel.8704 | 5.413257 | 0.004176 | 0.039954 | Chr8 | 78490253 | 78492193 | 541 |
| MsG0380013359.01 | 5.412118 | 0.004182 | 0.040001 | Chr3 | 35891950 | 35892651 | 375 |
| MsG0280010814.01 | 1.20158 | 0.004191 | 0.040067 | Chr2 | 75370416 | 75373530 | 1506 |
| novel.6196 | -6.46814 | 0.004192 | 0.040067 | Chr6 | 8963509 | 8965644 | 802 |
| MsG0880046552.01 | 3.404034 | 0.004194 | 0.040067 | Chr8 | 74778742 | 74783570 | 631 |
| novel.5947 | 1.634764 | 0.004195 | 0.040067 | Chr6 | 88360864 | 88365189 | 2135 |
| MsG0880042109.01 | -1.27103 | 0.004197 | 0.040067 | Chr8 | 3813377 | 3819212 | 2385 |
| MsG0280010645.01 | -1.09978 | 0.004198 | 0.040067 | Chr2 | 72775929 | 72778415 | 2487 |
| MsG0780036338.01 | -1.65915 | 0.004198 | 0.040067 | Chr7 | 6654820 | 6656607 | 1788 |
| MsG0680030581.01 | 5.48556 | 0.004205 | 0.040119 | Chr6 | 5639400 | 5643196 | 1137 |
| novel.4363 | -1.33597 | 0.004207 | 0.040119 | Chr5 | 12353306 | 12357935 | 3494 |
| novel.6368 | -1.87691 | 0.004209 | 0.040119 | Chr6 | 33875984 | 33877707 | 1657 |
| MsG0380014378.01 | -6.41064 | 0.00421 | 0.040119 | Chr3 | 53902433 | 53902852 | 420 |
| MsG0880044386.01 | 1.349948 | 0.004213 | 0.040134 | Chr8 | 41154719 | 41161047 | 2518 |
| novel.7505 | 1.11055 | 0.004214 | 0.040134 | Chr7 | 47969291 | 47974916 | 2310 |
| novel.5412 | 2.401075 | 0.004218 | 0.04016 | Chr5 | 97054586 | 97058042 | 1242 |
| MsG0780039263.01 | -1.04991 | 0.004225 | 0.040199 | Chr7 | 60948467 | 60954778 | 3806 |
| MsG0880042613.01 | -3.13918 | 0.004232 | 0.04025 | Chr8 | 11463273 | 11469220 | 4052 |
| MsG0680031559.01 | -2.20129 | 0.004236 | 0.040275 | Chr6 | 22299356 | 22306570 | 1378 |
| MsG0580025813.01 | -1.32991 | 0.004243 | 0.04033 | Chr5 | 24567153 | 24577268 | 1527 |
| novel.2031 | 1.436852 | 0.004245 | 0.040341 | Chr2 | 74385755 | 74387260 | 1506 |
| novel.3075 | -4.52198 | 0.004247 | 0.040349 | Chr3 | 82752471 | 82753708 | 1007 |
| MsG0180004694.01 | -1.72831 | 0.004252 | 0.040369 | Chr1 | 81317490 | 81335163 | 3765 |
| MsG0180004817.01 | 2.031158 | 0.004252 | 0.040369 | Chr1 | 83173163 | 83173720 | 558 |
| novel.8113 | -5.21038 | 0.004254 | 0.040379 | Chr8 | 61634372 | 61635395 | 391 |
| MsG0780037943.01 | -1.07772 | 0.004258 | 0.040398 | Chr7 | 36708108 | 36710631 | 1071 |
| MsG0480020082.01 | -3.7599 | 0.00426 | 0.040406 | Chr4 | 33679026 | 33681764 | 771 |
| novel.5840 | -5.43058 | 0.004264 | 0.040429 | Chr6 | 68258977 | 68260581 | 642 |
| MsG0380015975.01 | -4.50939 | 0.004266 | 0.040429 | Chr3 | 77402831 | 77404842 | 1332 |
| MsG0480022611.01 | -1.72158 | 0.004266 | 0.040429 | Chr4 | 74642690 | 74646806 | 1979 |
| novel.8654 | 4.404449 | 0.004269 | 0.040438 | Chr8 | 69694556 | 69696097 | 817 |
| novel.4593 | 3.176599 | 0.00427 | 0.040438 | Chr5 | 55684435 | 55690945 | 523 |
| MsG0780039572.01 | -2.54131 | 0.004274 | 0.040463 | Chr7 | 66035895 | 66038399 | 699 |
| MsG0280006991.01 | -1.92333 | 0.00428 | 0.040492 | Chr2 | 9088828 | 9101513 | 1218 |
| novel.6597 | -5.02138 | 0.00428 | 0.040492 | Chr6 | 82446294 | 82447774 | 445 |
| novel.1275 | 1.666306 | 0.004305 | 0.040719 | Chr2 | 17105771 | 17106408 | 555 |
| MsG0680031840.01 | 1.482744 | 0.004308 | 0.040719 | Chr6 | 27838183 | 27839625 | 1443 |
| MsG0780036976.01 | 1.808374 | 0.004308 | 0.040719 | Chr7 | 17429009 | 17430237 | 552 |
| MsG0480023041.01 | 1.409879 | 0.004309 | 0.040722 | Chr4 | 80087084 | 80088136 | 702 |
| MsG0180005509.01 | -2.4683 | 0.004329 | 0.040875 | Chr1 | 92682305 | 92685157 | 1124 |
| MsG0180004404.01 | 1.24199 | 0.00433 | 0.040875 | Chr1 | 77527585 | 77531491 | 1076 |
| novel.5151 | -4.36535 | 0.00433 | 0.040875 | Chr5 | 48087209 | 48097500 | 7846 |
| MsG0480021771.01 | -2.03648 | 0.004331 | 0.040875 | Chr4 | 64096932 | 64110377 | 942 |
| novel.468 | 3.271226 | 0.004344 | 0.040982 | Chr1 | 84981056 | 84982132 | 1077 |
| MsG0580027618.01 | -1.07717 | 0.004368 | 0.041177 | Chr5 | 64447836 | 64452484 | 1779 |
| MsG0480021719.01 | -1.89139 | 0.004368 | 0.041177 | Chr4 | 63423132 | 63428386 | 1672 |
| MsG0780040375.01 | -1.36066 | 0.004387 | 0.041338 | Chr7 | 76714966 | 76721336 | 1576 |
| MsG0380015050.01 | -2.43792 | 0.00439 | 0.041355 | Chr3 | 64518075 | 64518703 | 549 |
| MsG0780039081.01 | 1.683638 | 0.004401 | 0.041432 | Chr7 | 57610369 | 57621052 | 4908 |
| MsG0280011328.01 | 4.265256 | 0.004411 | 0.041505 | Chr2 | 82458957 | 82459322 | 366 |
| novel.9174 | 4.559745 | 0.004417 | 0.041545 | contig529end | 22716 | 25054 | 1592 |
| MsG0580024171.01 | -2.67669 | 0.004445 | 0.041802 | Chr5 | 1533793 | 1535762 | 1330 |
| novel.1864 | 4.935486 | 0.004452 | 0.041847 | Chr2 | 36256313 | 36259521 | 774 |
| MsG0880046399.01 | -1.27521 | 0.004467 | 0.041977 | Chr8 | 72872452 | 72882325 | 2733 |
| MsG0380012194.01 | -1.3796 | 0.004479 | 0.042066 | Chr3 | 12453070 | 12461190 | 3732 |
| MsG0280009054.01 | 1.297277 | 0.00448 | 0.042067 | Chr2 | 44401882 | 44402205 | 324 |
| MsG0480021109.01 | 1.047093 | 0.004486 | 0.042089 | Chr4 | 53605613 | 53607693 | 1691 |
| novel.9305 | -3.28901 | 0.004486 | 0.042089 | contig611end | 18560 | 21939 | 1441 |
| novel.4302 | -5.95618 | 0.004493 | 0.042144 | Chr5 | 3020652 | 3022110 | 1121 |
| novel.3853 | -3.24817 | 0.004495 | 0.042147 | Chr4 | 19976801 | 19978241 | 332 |
| MsG0280006557.01 | 5.446743 | 0.004497 | 0.042157 | Chr2 | 3410650 | 3418347 | 1842 |
| MsG0880046449.01 | 1.064428 | 0.00452 | 0.042362 | Chr8 | 73541908 | 73546566 | 912 |
| novel.7453 | -2.02961 | 0.004524 | 0.042374 | Chr7 | 35058564 | 35064477 | 2056 |
| MsG0380017686.01 | -2.43734 | 0.004528 | 0.042396 | Chr3 | 99543977 | 99548813 | 2181 |
| novel.5030 | -1.2608 | 0.004534 | 0.042437 | Chr5 | 23401243 | 23406471 | 2119 |
| MsG0780037917.01 | -1.31233 | 0.004538 | 0.042449 | Chr7 | 36350617 | 36355097 | 1008 |
| novel.5075 | -1.90893 | 0.004546 | 0.042513 | Chr5 | 31928917 | 31932562 | 1692 |
| novel.2126 | 1.768649 | 0.004561 | 0.042642 | Chr3 | 1177795 | 1186408 | 1370 |
| MsG0580029466.01 | -3.51472 | 0.004565 | 0.042667 | Chr5 | 97303785 | 97306826 | 3042 |
| MsG0380014168.01 | -2.49864 | 0.00458 | 0.042795 | Chr3 | 50805251 | 50807780 | 1449 |
| MsG0880043128.01 | -1.97155 | 0.004602 | 0.042982 | Chr8 | 19416468 | 19422800 | 486 |
| MsG0480020241.01 | -2.48461 | 0.004612 | 0.043064 | Chr4 | 37263667 | 37268874 | 3145 |
| novel.7626 | -1.67277 | 0.004621 | 0.043125 | Chr7 | 72719570 | 72723121 | 3450 |
| MsG0680034699.01 | 1.689978 | 0.004641 | 0.04329 | Chr6 | 93048844 | 93054057 | 2818 |
| MsG0180001281.01 | 3.019499 | 0.004643 | 0.043302 | Chr1 | 18736226 | 18738558 | 1464 |
| MsG0780036970.01 | -1.73988 | 0.00466 | 0.043446 | Chr7 | 17346548 | 17347994 | 1122 |
| novel.5017 | -1.19501 | 0.00467 | 0.043523 | Chr5 | 20522943 | 20528427 | 1062 |
| MsG0780041687.01 | 1.253077 | 0.004679 | 0.043599 | Chr7 | 93947517 | 93955653 | 2428 |
| novel.6997 | 4.535431 | 0.004686 | 0.043651 | Chr7 | 46430718 | 46446126 | 587 |
| MsG0480018435.01 | -1.06411 | 0.004691 | 0.043679 | Chr4 | 4851472 | 4860560 | 4744 |
| novel.8072 | -1.4743 | 0.004704 | 0.043788 | Chr8 | 54612676 | 54614082 | 1323 |
| MsG0480022815.01 | -1.55814 | 0.004708 | 0.043807 | Chr4 | 77012163 | 77017153 | 1821 |
| MsG0580024656.01 | 1.154878 | 0.00471 | 0.043807 | Chr5 | 7798015 | 7798959 | 945 |
| MsG0580028271.01 | -5.57334 | 0.004711 | 0.043807 | Chr5 | 77606771 | 77611476 | 1100 |
| MsG0380013724.01 | 3.197348 | 0.004712 | 0.043807 | Chr3 | 44610992 | 44621090 | 2148 |
| novel.8937 | 1.031478 | 0.004713 | 0.043807 | contig327end | 11093 | 19440 | 1615 |
| MsG0180005871.01 | 1.212772 | 0.00473 | 0.043925 | Chr1 | 97049005 | 97049969 | 906 |
| novel.1977 | 1.666853 | 0.00475 | 0.044046 | Chr2 | 65631566 | 65632311 | 638 |
| MsG0580024148.01 | 1.031931 | 0.00475 | 0.044046 | Chr5 | 1341677 | 1352041 | 3044 |
| novel.1565 | 4.743127 | 0.004752 | 0.044046 | Chr2 | 76861781 | 76864337 | 920 |
| MsG0580025977.01 | -1.92483 | 0.004768 | 0.044175 | Chr5 | 28460851 | 28462821 | 837 |
| MsG0180000942.01 | -1.51642 | 0.004769 | 0.044175 | Chr1 | 13496285 | 13500754 | 1179 |
| MsG0680030883.01 | -1.13891 | 0.004771 | 0.044187 | Chr6 | 11100203 | 11102696 | 972 |
| MsG0480023124.01 | -1.25125 | 0.004775 | 0.044212 | Chr4 | 81139764 | 81140114 | 351 |
| MsG0480022705.01 | -3.11194 | 0.004789 | 0.044326 | Chr4 | 75684029 | 75685765 | 1683 |
| MsG0280008673.01 | -1.64173 | 0.004808 | 0.044471 | Chr2 | 36128657 | 36132509 | 1644 |
| MsG0580025218.01 | -1.02054 | 0.004811 | 0.044487 | Chr5 | 15407886 | 15409774 | 1773 |
| novel.4524 | -5.12818 | 0.004822 | 0.044573 | Chr5 | 43752162 | 43789898 | 424 |
| novel.3097 | -5.85283 | 0.004835 | 0.04468 | Chr3 | 86605669 | 86607222 | 659 |
| MsG0480022311.01 | -1.2345 | 0.004837 | 0.044683 | Chr4 | 71001478 | 71003798 | 2038 |
| MsG0880046515.01 | 1.243528 | 0.004844 | 0.044735 | Chr8 | 74262999 | 74265511 | 1321 |
| novel.9296 | -1.62769 | 0.004849 | 0.044773 | contig606end | 7214 | 9253 | 1531 |
| novel.5162 | 5.525906 | 0.004859 | 0.044847 | Chr5 | 51571413 | 51573320 | 586 |
| MsG0680031848.01 | 1.211525 | 0.004873 | 0.044937 | Chr6 | 28129823 | 28132719 | 2079 |
| MsG0680030542.01 | -1.54128 | 0.004874 | 0.044937 | Chr6 | 4968998 | 4970808 | 1251 |
| novel.5938 | 1.04815 | 0.004874 | 0.044937 | Chr6 | 85985491 | 85988031 | 2541 |
| MsG0880046330.01 | 1.873543 | 0.004896 | 0.045111 | Chr8 | 72088753 | 72089388 | 636 |
| MsG0380015988.01 | 2.274891 | 0.004902 | 0.045151 | Chr3 | 77578250 | 77582097 | 2845 |
| MsG0880045416.01 | -1.60158 | 0.004909 | 0.045171 | Chr8 | 58913469 | 58919234 | 1711 |
| MsG0580024346.01 | -1.26685 | 0.004909 | 0.045171 | Chr5 | 3634714 | 3651055 | 797 |
| MsG0180003092.01 | -4.10805 | 0.00491 | 0.045171 | Chr1 | 56431632 | 56435267 | 1899 |
| novel.7063 | -5.49704 | 0.004916 | 0.045213 | Chr7 | 61517140 | 61517436 | 297 |
| MsG0580025638.01 | -2.33257 | 0.004918 | 0.04522 | Chr5 | 21614258 | 21615067 | 810 |
| MsG0480023011.01 | -1.05083 | 0.004935 | 0.045317 | Chr4 | 79662285 | 79676240 | 1854 |
| novel.7152 | -1.82773 | 0.004935 | 0.045317 | Chr7 | 76591859 | 76594362 | 1387 |
| MsG0680035842.01 | -4.72141 | 0.004956 | 0.04549 | Chr6 | 1.13E+08 | 1.13E+08 | 840 |
| MsG0580025869.01 | -1.08033 | 0.004957 | 0.04549 | Chr5 | 25544149 | 25545126 | 978 |
| MsG0480023177.01 | -5.43134 | 0.00496 | 0.045506 | Chr4 | 81831745 | 81832508 | 490 |
| novel.6704 | -2.71291 | 0.004969 | 0.045574 | Chr6 | 1.03E+08 | 1.03E+08 | 780 |
| novel.6869 | -2.78675 | 0.004988 | 0.04572 | Chr7 | 18487018 | 18488852 | 1242 |
| MsG0880045422.01 | -6.37145 | 0.004996 | 0.045782 | Chr8 | 58976001 | 58976411 | 411 |
| MsG0380016629.01 | 2.701036 | 0.00501 | 0.04588 | Chr3 | 85875881 | 85882262 | 2187 |
| novel.4649 | 2.653348 | 0.005011 | 0.045882 | Chr5 | 68615347 | 68616717 | 911 |
| MsG0480022878.01 | 2.144244 | 0.005018 | 0.045914 | Chr4 | 77965554 | 77972215 | 1947 |
| MsG0880046349.01 | 1.195827 | 0.005028 | 0.045988 | Chr8 | 72288545 | 72289003 | 459 |
| MsG0680031640.01 | -2.10283 | 0.00503 | 0.045995 | Chr6 | 23802912 | 23815497 | 1797 |
| novel.5177 | 3.76687 | 0.005035 | 0.046025 | Chr5 | 54906675 | 54907393 | 719 |
| novel.1537 | -5.56429 | 0.005047 | 0.0461 | Chr2 | 71374204 | 71374643 | 347 |
| novel.1192 | -2.33334 | 0.005047 | 0.0461 | Chr2 | 3887736 | 3900843 | 1350 |
| MsG0680035203.01 | -1.49438 | 0.005056 | 0.046162 | Chr6 | 1.01E+08 | 1.01E+08 | 4668 |
| MsG0080048057.01 | -1.62371 | 0.005057 | 0.046162 | contig184end | 26156 | 26776 | 489 |
| MsG0480023761.01 | -1.20657 | 0.005066 | 0.046227 | Chr4 | 89514773 | 89516509 | 803 |
| MsG0780040352.01 | -1.06813 | 0.005067 | 0.046227 | Chr7 | 76329634 | 76355783 | 5428 |
| MsG0280009638.01 | 5.354002 | 0.005081 | 0.046341 | Chr2 | 55699476 | 55700827 | 942 |
| MsG0780038807.01 | -1.58485 | 0.005088 | 0.046393 | Chr7 | 53321320 | 53323492 | 886 |
| MsG0180001988.01 | -2.19577 | 0.005093 | 0.046407 | Chr1 | 30384576 | 30392007 | 2232 |
| MsG0880047159.01 | -5.55626 | 0.005103 | 0.046484 | Chr8 | 82807885 | 82808927 | 930 |
| MsG0380015356.01 | 1.322176 | 0.005106 | 0.046502 | Chr3 | 68961904 | 68966191 | 738 |
| MsG0380014882.01 | -1.43905 | 0.00511 | 0.046517 | Chr3 | 61925829 | 61928882 | 971 |
| MsG0280009453.01 | -3.0573 | 0.005123 | 0.04658 | Chr2 | 52329824 | 52330345 | 522 |
| MsG0380012168.01 | -1.99583 | 0.00513 | 0.046622 | Chr3 | 12038642 | 12038995 | 354 |
| novel.8047 | -3.7533 | 0.00513 | 0.046622 | Chr8 | 50327805 | 50329208 | 807 |
| MsG0480019126.01 | -1.09874 | 0.005137 | 0.04667 | Chr4 | 14833426 | 14838758 | 1321 |
| novel.1990 | -3.71783 | 0.005139 | 0.046672 | Chr2 | 67995547 | 67996536 | 950 |
| novel.6044 | 2.344683 | 0.005152 | 0.04678 | Chr6 | 1.03E+08 | 1.03E+08 | 3745 |
| MsG0480018807.01 | 1.371805 | 0.005155 | 0.046794 | Chr4 | 10151631 | 10153571 | 965 |
| novel.4374 | -5.31985 | 0.005162 | 0.046841 | Chr5 | 14523677 | 14525262 | 668 |
| novel.4217 | 2.649066 | 0.005178 | 0.046955 | Chr4 | 85574515 | 85576219 | 979 |
| MsG0580029130.01 | -1.69472 | 0.005179 | 0.046955 | Chr5 | 92741973 | 92742206 | 234 |
| novel.8109 | 1.163437 | 0.005199 | 0.047122 | Chr8 | 61191675 | 61196166 | 963 |
| MsG0880045895.01 | 1.649699 | 0.005209 | 0.047195 | Chr8 | 66092648 | 66093040 | 393 |
| MsG0880044572.01 | -1.96515 | 0.005214 | 0.047215 | Chr8 | 44840989 | 44842706 | 1635 |
| MsG0280007479.01 | 1.065704 | 0.005234 | 0.047379 | Chr2 | 16014231 | 16014593 | 363 |
| MsG0380016272.01 | -1.42182 | 0.005244 | 0.047442 | Chr3 | 81310738 | 81319255 | 8436 |
| novel.5243 | -2.15293 | 0.005244 | 0.047442 | Chr5 | 69188029 | 69189991 | 1202 |
| MsG0380016060.01 | -1.48106 | 0.005255 | 0.047509 | Chr3 | 78513545 | 78514291 | 747 |
| MsG0880045543.01 | -1.23943 | 0.005256 | 0.047509 | Chr8 | 60800828 | 60815611 | 2058 |
| novel.6156 | 5.44774 | 0.00526 | 0.047528 | Chr6 | 3639607 | 3640370 | 650 |
| MsG0580027343.01 | 1.072063 | 0.005265 | 0.047565 | Chr5 | 58763847 | 58768232 | 1514 |
| novel.1080 | -3.32606 | 0.005268 | 0.047574 | Chr1 | 92049149 | 92051801 | 1181 |
| novel.5813 | -2.26511 | 0.005285 | 0.047685 | Chr6 | 61070047 | 61077605 | 3092 |
| novel.252 | -5.36593 | 0.005288 | 0.0477 | Chr1 | 40198807 | 40199852 | 608 |
| MsG0180000969.01 | 1.160638 | 0.005306 | 0.047847 | Chr1 | 13869514 | 13870503 | 990 |
| MsG0080047939.01 | 1.725865 | 0.005308 | 0.047848 | contig152end | 65919 | 70759 | 573 |
| MsG0780037452.01 | 2.261194 | 0.005312 | 0.047863 | Chr7 | 26147521 | 26151339 | 1918 |
| MsG0580025853.01 | 2.342843 | 0.005321 | 0.047926 | Chr5 | 25172629 | 25183340 | 5850 |
| MsG0380017877.01 | -1.70382 | 0.005327 | 0.047955 | Chr3 | 1.02E+08 | 1.02E+08 | 2356 |
| novel.6509 | -4.81494 | 0.005336 | 0.04799 | Chr6 | 64195757 | 64201762 | 646 |
| novel.8888 | -2.71187 | 0.00536 | 0.048178 | contig253end | 48921 | 52334 | 3414 |
| MsG0280009565.01 | 1.023316 | 0.005368 | 0.048208 | Chr2 | 54413001 | 54414370 | 495 |
| MsG0580029013.01 | 1.935216 | 0.005404 | 0.048496 | Chr5 | 90319347 | 90327568 | 4551 |
| MsG0280008007.01 | 1.143225 | 0.005405 | 0.048496 | Chr2 | 24221064 | 24222011 | 948 |
| novel.7671 | 5.378927 | 0.005412 | 0.048545 | Chr7 | 79348675 | 79349885 | 332 |
| MsG0180000778.01 | 1.3437 | 0.005417 | 0.048573 | Chr1 | 11028428 | 11028988 | 561 |
| novel.6919 | 3.629284 | 0.005426 | 0.048647 | Chr7 | 27323603 | 27326009 | 825 |
| MsG0780041157.01 | 1.790821 | 0.005434 | 0.048683 | Chr7 | 87251839 | 87252852 | 1014 |
| MsG0480019649.01 | -1.40091 | 0.005444 | 0.04876 | Chr4 | 24044619 | 24044903 | 285 |
| novel.4492 | 1.424516 | 0.005454 | 0.048839 | Chr5 | 34030314 | 34031771 | 1304 |
| MsG0880044035.01 | 1.86517 | 0.005458 | 0.04884 | Chr8 | 34615403 | 34621036 | 1066 |
| MsG0580025259.01 | 1.257739 | 0.005459 | 0.04884 | Chr5 | 16068021 | 16069262 | 1242 |
| novel.2751 | -2.37028 | 0.005474 | 0.048963 | Chr3 | 11741822 | 11743572 | 1000 |
| novel.6728 | 3.178462 | 0.005506 | 0.049229 | Chr6 | 1.05E+08 | 1.05E+08 | 1034 |
| MsG0280009700.01 | -2.42342 | 0.005523 | 0.049358 | Chr2 | 56808255 | 56819937 | 2487 |
| MsG0080048957.01 | 4.204844 | 0.005533 | 0.049416 | contig567end | 20599 | 21446 | 773 |
| MsG0180001683.01 | 4.687226 | 0.005555 | 0.049579 | Chr1 | 25119073 | 25121874 | 1203 |
| novel.8803 | -1.52467 | 0.005564 | 0.049645 | contig125end | 8386 | 9788 | 1403 |
| novel.8552 | -4.78504 | 0.00557 | 0.049687 | Chr8 | 52067128 | 52074998 | 476 |
| MsG0680031692.01 | -1.26968 | 0.005578 | 0.04973 | Chr6 | 24687621 | 24689575 | 1223 |
| MsG0580030046.01 | -1.47719 | 0.005585 | 0.049775 | Chr5 | 1.06E+08 | 1.06E+08 | 1098 |
| novel.5696 | 2.671778 | 0.005587 | 0.049784 | Chr6 | 31625322 | 31627450 | 1392 |
| novel.204 | -4.68157 | 0.005594 | 0.04981 | Chr1 | 28667890 | 28668593 | 624 |
| MsG0280008216.01 | 3.486331 | 0.005594 | 0.04981 | Chr2 | 27319836 | 27323252 | 1986 |
| MsG0180005086.01 | 1.118185 | 0.005595 | 0.04981 | Chr1 | 86773796 | 86779105 | 877 |
| novel.728 | -6.28903 | 0.005599 | 0.049824 | Chr1 | 17771954 | 17772955 | 743 |
| MsG0380016137.01 | -1.70586 | 0.005607 | 0.049889 | Chr3 | 79504693 | 79506513 | 685 |
| MsG0380015158.01 | -5.44977 | 0.005611 | 0.049905 | Chr3 | 65965869 | 65966432 | 564 |
| MsG0380015667.01 | 3.168184 | 0.005618 | 0.049951 | Chr3 | 73326376 | 73335354 | 999 |
| MsG0580030245.01 | -5.63284 | 0.005619 | 0.049953 | Chr5 | 1.09E+08 | 1.09E+08 | 1113 |
