# Supplementary material for: Overexpression of an NF-YC2 gene confers alkali tolerance to transgenic alfalfa (Medicago sativa L.)
Source: Front Plant Sci. 2022 Aug 5;13:960160. doi: 10.3389/fpls.2022.960160 (PMC9389336; doi:10.3389/fpls.2022.960160)
Supplement: Supplementary file 4 [file Table_4.docx]

| **Supplementary** **Table 4. Differently-expressed genes between transgenic alfalfa plants and WT under alkali stress conditions.** | | | | | | | |
| --- | --- | --- | --- | --- | --- | --- | --- |
| gene_id | log2FoldChange | pvalue | padj | gene_chr | gene_start | gene_end | gene_length |
| novel.5617 | -8.06025 | 6.93E-42 | 2.06E-37 | Chr6 | 17667117 | 17667738 | 622 |
| MsG0280008038.01 | -6.63324 | 4.99E-35 | 7.40E-31 | Chr2 | 24555296 | 24561945 | 3204 |
| novel.4776 | -6.86126 | 3.13E-32 | 3.10E-28 | Chr5 | 91036930 | 91045032 | 6203 |
| novel.2381 | 6.344865 | 5.81E-29 | 4.31E-25 | Chr3 | 55139168 | 55140887 | 1720 |
| MsG0480021203.01 | -6.93415 | 9.02E-29 | 5.35E-25 | Chr4 | 55098713 | 55104905 | 2572 |
| MsG0580028882.01 | -4.71781 | 1.74E-28 | 8.62E-25 | Chr5 | 87992399 | 87993199 | 801 |
| novel.1419 | 14.02208 | 4.51E-28 | 1.91E-24 | Chr2 | 46351607 | 46352702 | 1096 |
| MsG0680032109.01 | -8.18716 | 1.22E-26 | 4.52E-23 | Chr6 | 33907839 | 33910652 | 2061 |
| novel.8934 | 12.89436 | 1.63E-24 | 5.39E-21 | contig315end | 23509 | 26204 | 725 |
| MsG0680035721.01 | 7.603278 | 6.23E-24 | 1.85E-20 | Chr6 | 1.11E+08 | 1.11E+08 | 4389 |
| MsG0180003592.01 | 8.234811 | 1.75E-22 | 4.71E-19 | Chr1 | 64910824 | 64916751 | 2044 |
| MsG0280010535.01 | -6.08122 | 2.15E-22 | 5.33E-19 | Chr2 | 71130479 | 71131441 | 963 |
| MsG0880045491.01 | -6.38141 | 6.05E-22 | 1.38E-18 | Chr8 | 59990755 | 59991642 | 828 |
| MsG0380015003.01 | -7.89929 | 4.28E-21 | 8.55E-18 | Chr3 | 63779636 | 63790311 | 2288 |
| MsG0880042185.01 | -12.1451 | 4.32E-21 | 8.55E-18 | Chr8 | 5088182 | 5089281 | 948 |
| novel.2268 | 5.245318 | 1.15E-20 | 2.13E-17 | Chr3 | 23285856 | 23294564 | 4802 |
| MsG0480020539.01 | 6.685584 | 1.51E-20 | 2.64E-17 | Chr4 | 43035369 | 43045234 | 2066 |
| MsG0880045407.01 | -11.5972 | 1.97E-20 | 3.24E-17 | Chr8 | 58844578 | 58847727 | 1616 |
| novel.6432 | 6.219752 | 2.70E-20 | 4.22E-17 | Chr6 | 45846048 | 45851532 | 1784 |
| MsG0480020574.01 | -6.98405 | 3.22E-20 | 4.78E-17 | Chr4 | 43654757 | 43657902 | 846 |
| MsG0480023665.01 | -7.5322 | 3.59E-20 | 5.07E-17 | Chr4 | 88318984 | 88320348 | 1365 |
| novel.6928 | -8.35541 | 5.41E-20 | 7.30E-17 | Chr7 | 29167611 | 29169431 | 1821 |
| MsG0880042079.01 | 6.431251 | 8.24E-20 | 1.06E-16 | Chr8 | 3471109 | 3481432 | 4710 |
| novel.2754 | 6.349464 | 1.07E-19 | 1.33E-16 | Chr3 | 12210588 | 12220083 | 670 |
| novel.4476 | 11.30436 | 1.42E-19 | 1.69E-16 | Chr5 | 29900412 | 29910396 | 6901 |
| MsG0680031300.01 | -11.2442 | 1.76E-19 | 2.01E-16 | Chr6 | 17666882 | 17667100 | 219 |
| MsG0380015103.01 | 3.767995 | 9.93E-19 | 1.09E-15 | Chr3 | 65331148 | 65333323 | 919 |
| novel.3180 | 11.88754 | 1.49E-18 | 1.58E-15 | Chr3 | 99398050 | 99400218 | 2169 |
| MsG0780040610.01 | -9.25609 | 2.52E-18 | 2.58E-15 | Chr7 | 79921410 | 79935960 | 948 |
| MsG0480021561.01 | 4.572351 | 3.28E-18 | 3.24E-15 | Chr4 | 59844999 | 59846597 | 940 |
| MsG0880044221.01 | -11.1772 | 3.44E-18 | 3.30E-15 | Chr8 | 37869045 | 37870424 | 1380 |
| novel.8343 | 5.747967 | 4.18E-18 | 3.87E-15 | Chr8 | 8695657 | 8700437 | 1104 |
| MsG0280010547.01 | 11.66873 | 5.16E-18 | 4.64E-15 | Chr2 | 71282192 | 71284832 | 993 |
| MsG0580029554.01 | 11.35898 | 8.95E-18 | 7.81E-15 | Chr5 | 98465795 | 98477670 | 3918 |
| MsG0880042492.01 | -11.8914 | 9.42E-18 | 7.99E-15 | Chr8 | 9519747 | 9524484 | 2340 |
| novel.8335 | 5.136391 | 1.33E-17 | 1.09E-14 | Chr8 | 7179978 | 7183701 | 764 |
| MsG0080048216.01 | 6.260271 | 1.60E-17 | 1.28E-14 | contig245end | 22402 | 24039 | 649 |
| MsG0080047938.01 | -6.40053 | 1.95E-17 | 1.53E-14 | contig152end | 59103 | 61816 | 2049 |
| novel.2967 | 5.941088 | 2.20E-17 | 1.68E-14 | Chr3 | 65550220 | 65551657 | 745 |
| MsG0580029732.01 | 4.371413 | 2.96E-17 | 2.20E-14 | Chr5 | 1.01E+08 | 1.01E+08 | 1381 |
| MsG0480019219.01 | 6.027303 | 3.08E-17 | 2.23E-14 | Chr4 | 16347994 | 16353683 | 1032 |
| MsG0180005378.01 | 11.09146 | 4.63E-17 | 3.27E-14 | Chr1 | 91017485 | 91019805 | 1643 |
| MsG0880045994.01 | 3.529287 | 5.00E-17 | 3.42E-14 | Chr8 | 67372775 | 67378220 | 1009 |
| novel.5039 | 5.701425 | 5.07E-17 | 3.42E-14 | Chr5 | 25553619 | 25557156 | 3166 |
| MsG0680035786.01 | 5.661306 | 1.32E-16 | 8.73E-14 | Chr6 | 1.12E+08 | 1.12E+08 | 532 |
| MsG0880045055.01 | -3.73867 | 1.42E-16 | 9.00E-14 | Chr8 | 53493186 | 53494699 | 738 |
| novel.7577 | -5.70252 | 1.43E-16 | 9.00E-14 | Chr7 | 65593890 | 65601504 | 3184 |
| novel.2088 | -10.4018 | 1.53E-16 | 9.47E-14 | Chr2 | 81622465 | 81626167 | 2999 |
| novel.457 | 3.891982 | 1.75E-16 | 1.06E-13 | Chr1 | 83776282 | 83778710 | 1084 |
| novel.2331 | 11.35195 | 2.88E-16 | 1.71E-13 | Chr3 | 41206874 | 41207986 | 1113 |
| MsG0880045492.01 | -5.65213 | 3.04E-16 | 1.77E-13 | Chr8 | 59991660 | 59992115 | 456 |
| novel.2220 | -10.3709 | 3.28E-16 | 1.87E-13 | Chr3 | 15986852 | 16006849 | 5728 |
| MsG0480019193.01 | -5.89977 | 3.41E-16 | 1.88E-13 | Chr4 | 15791187 | 15798458 | 1161 |
| MsG0380013598.01 | 5.342498 | 3.41E-16 | 1.88E-13 | Chr3 | 41423445 | 41428939 | 1729 |
| MsG0780037661.01 | 3.659863 | 4.24E-16 | 2.29E-13 | Chr7 | 30524960 | 30529183 | 766 |
| novel.908 | -4.63011 | 5.40E-16 | 2.86E-13 | Chr1 | 60892856 | 60897047 | 3000 |
| novel.3352 | -6.73326 | 5.55E-16 | 2.89E-13 | Chr4 | 24574197 | 24576360 | 1125 |
| novel.7971 | -4.70899 | 5.66E-16 | 2.90E-13 | Chr8 | 33982210 | 33982524 | 315 |
| novel.7866 | -10.9422 | 6.42E-16 | 3.23E-13 | Chr8 | 12213657 | 12216206 | 1791 |
| MsG0480021407.01 | 10.95636 | 1.19E-15 | 5.90E-13 | Chr4 | 57583426 | 57584153 | 637 |
| MsG0180001124.01 | -5.3924 | 1.32E-15 | 6.42E-13 | Chr1 | 16316652 | 16319392 | 2212 |
| MsG0480018819.01 | -6.62867 | 1.48E-15 | 7.11E-13 | Chr4 | 10248746 | 10256282 | 3309 |
| novel.8231 | 10.22019 | 1.78E-15 | 8.39E-13 | Chr8 | 82685940 | 82689072 | 1765 |
| MsG0780036265.01 | 3.510024 | 2.98E-15 | 1.38E-12 | Chr7 | 5617727 | 5621877 | 2300 |
| novel.4575 | -10.0346 | 3.26E-15 | 1.49E-12 | Chr5 | 53389919 | 53402642 | 5830 |
| MsG0280010654.01 | -4.04515 | 4.03E-15 | 1.81E-12 | Chr2 | 73004171 | 73005586 | 1416 |
| novel.664 | 10.134 | 4.53E-15 | 2.01E-12 | Chr1 | 8878191 | 8886212 | 1104 |
| novel.5226 | -4.86445 | 4.63E-15 | 2.02E-12 | Chr5 | 65916174 | 65927589 | 9780 |
| MsG0280007420.01 | -7.15119 | 6.05E-15 | 2.60E-12 | Chr2 | 15169850 | 15170130 | 281 |
| novel.7757 | 3.56836 | 7.20E-15 | 3.05E-12 | Chr7 | 91771445 | 91774212 | 2768 |
| MsG0180003243.01 | 4.409063 | 7.58E-15 | 3.17E-12 | Chr1 | 58967754 | 58971132 | 798 |
| MsG0580025301.01 | 10.03923 | 9.53E-15 | 3.90E-12 | Chr5 | 16680894 | 16683322 | 1530 |
| MsG0580029669.01 | 10.55592 | 9.58E-15 | 3.90E-12 | Chr5 | 1.01E+08 | 1.01E+08 | 792 |
| MsG0680033953.01 | -4.15114 | 1.04E-14 | 4.16E-12 | Chr6 | 77294863 | 77295852 | 990 |
| MsG0280008566.01 | 9.92693 | 1.09E-14 | 4.30E-12 | Chr2 | 34066973 | 34074669 | 2434 |
| MsG0380014999.01 | -6.79028 | 1.11E-14 | 4.32E-12 | Chr3 | 63749183 | 63751026 | 1014 |
| MsG0880043781.01 | -5.25393 | 1.13E-14 | 4.34E-12 | Chr8 | 30510835 | 30513267 | 2118 |
| novel.6585 | -10.0494 | 1.14E-14 | 4.34E-12 | Chr6 | 80027362 | 80031236 | 2516 |
| novel.3144 | 10.76197 | 1.25E-14 | 4.70E-12 | Chr3 | 94769845 | 94771327 | 690 |
| MsG0480019191.01 | -5.09251 | 1.37E-14 | 5.08E-12 | Chr4 | 15764454 | 15770404 | 1233 |
| novel.5238 | 10.33781 | 1.93E-14 | 7.06E-12 | Chr5 | 68368149 | 68379681 | 2102 |
| MsG0380012869.01 | 2.989937 | 2.08E-14 | 7.52E-12 | Chr3 | 25938220 | 25958534 | 3498 |
| MsG0680031853.01 | -5.51641 | 2.22E-14 | 7.95E-12 | Chr6 | 28222949 | 28228779 | 1191 |
| novel.5731 | 10.18799 | 2.33E-14 | 8.24E-12 | Chr6 | 40459432 | 40462256 | 2188 |
| MsG0180006051.01 | -7.71009 | 2.49E-14 | 8.70E-12 | Chr1 | 99397147 | 99401169 | 915 |
| novel.5284 | 10.55235 | 2.71E-14 | 9.34E-12 | Chr5 | 74838736 | 74840716 | 1693 |
| MsG0180005083.01 | 10.42799 | 2.83E-14 | 9.65E-12 | Chr1 | 86734827 | 86740674 | 1569 |
| novel.536 | -4.26779 | 2.96E-14 | 9.99E-12 | Chr1 | 95603895 | 95610266 | 3224 |
| novel.4244 | -5.06955 | 3.27E-14 | 1.09E-11 | Chr4 | 88918716 | 88923620 | 1476 |
| MsG0480019226.01 | -3.21134 | 3.30E-14 | 1.09E-11 | Chr4 | 16547671 | 16549593 | 1741 |
| novel.6712 | -9.65846 | 3.75E-14 | 1.22E-11 | Chr6 | 1.04E+08 | 1.04E+08 | 1450 |
| MsG0480019192.01 | -4.83993 | 3.88E-14 | 1.25E-11 | Chr4 | 15781315 | 15787264 | 843 |
| novel.8609 | -10.1423 | 4.84E-14 | 1.54E-11 | Chr8 | 62375059 | 62378392 | 2320 |
| MsG0580025976.01 | 9.684703 | 4.87E-14 | 1.54E-11 | Chr5 | 28445748 | 28445969 | 222 |
| novel.1158 | 6.144152 | 5.36E-14 | 1.68E-11 | Chr1 | 1.02E+08 | 1.02E+08 | 3313 |
| novel.228 | 8.76097 | 6.01E-14 | 1.86E-11 | Chr1 | 35604955 | 35607365 | 2411 |
| MsG0080048743.01 | 5.530986 | 6.18E-14 | 1.89E-11 | contig412end | 2254 | 6979 | 729 |
| novel.8632 | -5.09786 | 6.50E-14 | 1.97E-11 | Chr8 | 65774642 | 65776546 | 1905 |
| MsG0380014508.01 | -3.16426 | 7.65E-14 | 2.29E-11 | Chr3 | 55866758 | 55867425 | 556 |
| novel.2801 | -9.55854 | 7.75E-14 | 2.30E-11 | Chr3 | 23701490 | 23706316 | 2658 |
| MsG0880047145.01 | -9.96104 | 8.36E-14 | 2.46E-11 | Chr8 | 82623803 | 82624731 | 249 |
| novel.9107 | -7.01627 | 9.52E-14 | 2.77E-11 | contig488end | 1994 | 3143 | 997 |
| novel.2883 | 5.249089 | 1.02E-13 | 2.93E-11 | Chr3 | 44151393 | 44161057 | 1730 |
| MsG0480018241.01 | 7.181672 | 1.10E-13 | 3.13E-11 | Chr4 | 2077386 | 2085603 | 2535 |
| MsG0780041594.01 | -7.96054 | 1.28E-13 | 3.62E-11 | Chr7 | 92831213 | 92834155 | 939 |
| novel.6586 | 10.08369 | 1.47E-13 | 4.13E-11 | Chr6 | 80144635 | 80147746 | 1138 |
| novel.8793 | 9.536869 | 1.66E-13 | 4.62E-11 | contig107end | 60650 | 63478 | 899 |
| novel.655 | -9.57833 | 1.79E-13 | 4.92E-11 | Chr1 | 7318753 | 7320671 | 1083 |
| novel.7795 | -4.43858 | 1.87E-13 | 5.09E-11 | Chr8 | 1161489 | 1165756 | 1331 |
| novel.5905 | 9.783281 | 1.95E-13 | 5.25E-11 | Chr6 | 80424582 | 80428634 | 3430 |
| MsG0780039513.01 | -5.44528 | 2.09E-13 | 5.58E-11 | Chr7 | 65073261 | 65081334 | 1785 |
| MsG0480020497.01 | -5.92346 | 2.15E-13 | 5.69E-11 | Chr4 | 42475239 | 42476099 | 861 |
| MsG0380017122.01 | -4.83075 | 2.62E-13 | 6.87E-11 | Chr3 | 92357180 | 92358957 | 1266 |
| MsG0680030683.01 | -4.48327 | 3.01E-13 | 7.84E-11 | Chr6 | 7156241 | 7162886 | 1977 |
| novel.2211 | -6.91544 | 3.25E-13 | 8.40E-11 | Chr3 | 14711974 | 14716994 | 2831 |
| MsG0680032857.01 | 4.699066 | 3.77E-13 | 9.64E-11 | Chr6 | 51291577 | 51297557 | 937 |
| novel.1485 | 4.948358 | 3.97E-13 | 1.01E-10 | Chr2 | 60283466 | 60286755 | 916 |
| MsG0280009948.01 | -4.17806 | 4.51E-13 | 1.13E-10 | Chr2 | 61541022 | 61549138 | 1811 |
| novel.152 | -4.24435 | 4.74E-13 | 1.18E-10 | Chr1 | 21214518 | 21723908 | 7524 |
| MsG0380014998.01 | -6.16156 | 5.07E-13 | 1.25E-10 | Chr3 | 63747995 | 63748537 | 543 |
| MsG0880045924.01 | -6.21813 | 5.24E-13 | 1.29E-10 | Chr8 | 66536746 | 66537084 | 339 |
| novel.2852 | 9.699842 | 5.60E-13 | 1.36E-10 | Chr3 | 33911676 | 33913833 | 1645 |
| MsG0680035608.01 | -6.01722 | 5.84E-13 | 1.41E-10 | Chr6 | 1.09E+08 | 1.09E+08 | 658 |
| novel.8316 | -5.77307 | 5.91E-13 | 1.41E-10 | Chr8 | 3104839 | 3105924 | 474 |
| MsG0180003346.01 | 10.21054 | 6.23E-13 | 1.48E-10 | Chr1 | 60938875 | 60955567 | 3978 |
| MsG0880042911.01 | 3.277663 | 6.67E-13 | 1.57E-10 | Chr8 | 15807776 | 15810568 | 1020 |
| novel.7012 | 5.116457 | 7.04E-13 | 1.65E-10 | Chr7 | 49219836 | 49223147 | 1474 |
| novel.2875 | 9.298808 | 7.11E-13 | 1.65E-10 | Chr3 | 39909791 | 39911460 | 222 |
| novel.7355 | 9.318514 | 7.72E-13 | 1.78E-10 | Chr7 | 13987504 | 13988274 | 771 |
| novel.8771 | -9.8967 | 9.39E-13 | 2.14E-10 | Chr8 | 88664465 | 88668678 | 4214 |
| novel.2996 | -9.11074 | 1.16E-12 | 2.58E-10 | Chr3 | 70716817 | 70718793 | 1681 |
| novel.8272 | -9.6975 | 1.17E-12 | 2.58E-10 | Chr8 | 88509070 | 88511408 | 2339 |
| novel.7512 | 4.969689 | 1.17E-12 | 2.58E-10 | Chr7 | 50365481 | 50367131 | 709 |
| MsG0480018371.01 | 3.715091 | 1.17E-12 | 2.58E-10 | Chr4 | 3950885 | 3952516 | 1018 |
| novel.4444 | -7.20106 | 1.18E-12 | 2.58E-10 | Chr5 | 25324982 | 25326302 | 1116 |
| novel.4879 | -5.28721 | 1.19E-12 | 2.61E-10 | Chr5 | 1.05E+08 | 1.05E+08 | 1319 |
| MsG0180005662.01 | -9.59573 | 1.24E-12 | 2.69E-10 | Chr1 | 94634694 | 94643885 | 1320 |
| MsG0880045816.01 | -9.47921 | 1.31E-12 | 2.81E-10 | Chr8 | 64848994 | 64849488 | 495 |
| novel.8022 | 4.686661 | 1.37E-12 | 2.91E-10 | Chr8 | 44197935 | 44198217 | 263 |
| MsG0480018522.01 | -5.56387 | 1.37E-12 | 2.91E-10 | Chr4 | 6107925 | 6108503 | 579 |
| novel.4631 | 6.564347 | 1.54E-12 | 3.23E-10 | Chr5 | 64035820 | 64039911 | 1509 |
| MsG0780040577.01 | 2.521989 | 1.62E-12 | 3.38E-10 | Chr7 | 79439944 | 79440162 | 219 |
| novel.6085 | -3.09538 | 1.70E-12 | 3.53E-10 | Chr6 | 1.07E+08 | 1.07E+08 | 3008 |
| MsG0480019195.01 | -4.37438 | 1.74E-12 | 3.59E-10 | Chr4 | 15815989 | 15816489 | 501 |
| MsG0780036090.01 | -2.60775 | 1.76E-12 | 3.61E-10 | Chr7 | 2314089 | 2318450 | 974 |
| novel.6077 | -6.10426 | 1.92E-12 | 3.90E-10 | Chr6 | 1.06E+08 | 1.06E+08 | 2511 |
| novel.1042 | 9.482507 | 2.01E-12 | 4.05E-10 | Chr1 | 87207395 | 87210319 | 739 |
| MsG0880045912.01 | 8.127748 | 2.08E-12 | 4.18E-10 | Chr8 | 66309143 | 66315889 | 1304 |
| novel.1065 | 7.140598 | 2.16E-12 | 4.31E-10 | Chr1 | 90259957 | 90265273 | 3816 |
| novel.2702 | 7.242166 | 2.18E-12 | 4.31E-10 | Chr3 | 5415899 | 5418180 | 1789 |
| MsG0280007321.01 | 3.026133 | 2.22E-12 | 4.37E-10 | Chr2 | 13559373 | 13573685 | 2112 |
| MsG0280008255.01 | 9.133782 | 2.35E-12 | 4.57E-10 | Chr2 | 28065966 | 28069305 | 1113 |
| novel.8445 | -9.26762 | 2.36E-12 | 4.57E-10 | Chr8 | 28869800 | 28878682 | 1939 |
| MsG0880045399.01 | -9.35874 | 2.53E-12 | 4.87E-10 | Chr8 | 58763126 | 58763689 | 564 |
| novel.584 | -4.92556 | 2.56E-12 | 4.90E-10 | Chr1 | 668563 | 670611 | 526 |
| novel.5382 | -5.19769 | 2.58E-12 | 4.92E-10 | Chr5 | 91960161 | 91964421 | 2066 |
| MsG0380013725.01 | -4.91708 | 2.61E-12 | 4.92E-10 | Chr3 | 44647588 | 44648367 | 780 |
| novel.5982 | 10.11085 | 2.62E-12 | 4.92E-10 | Chr6 | 94355321 | 94359952 | 2464 |
| MsG0680033624.01 | 7.178444 | 2.68E-12 | 5.00E-10 | Chr6 | 69709793 | 69720375 | 1410 |
| MsG0880045408.01 | -9.23436 | 2.77E-12 | 5.13E-10 | Chr8 | 58852092 | 58852655 | 564 |
| novel.5045 | -5.07771 | 2.85E-12 | 5.25E-10 | Chr5 | 26869392 | 26874131 | 2969 |
| novel.3283 | 8.513608 | 2.88E-12 | 5.28E-10 | Chr4 | 10985541 | 10989346 | 3624 |
| novel.2480 | -3.75421 | 2.93E-12 | 5.34E-10 | Chr3 | 75292532 | 75294904 | 2373 |
| novel.2735 | -9.05451 | 2.96E-12 | 5.36E-10 | Chr3 | 10105123 | 10109626 | 4504 |
| MsG0580029179.01 | 6.211097 | 3.49E-12 | 6.27E-10 | Chr5 | 93323941 | 93330908 | 3378 |
| MsG0480019585.01 | -3.26558 | 3.85E-12 | 6.87E-10 | Chr4 | 22683013 | 22687656 | 2199 |
| MsG0580026122.01 | 9.242485 | 3.87E-12 | 6.87E-10 | Chr5 | 31052625 | 31061263 | 582 |
| MsG0880044706.01 | -4.30229 | 4.05E-12 | 7.15E-10 | Chr8 | 47981568 | 47986305 | 2562 |
| novel.705 | 4.279449 | 4.21E-12 | 7.39E-10 | Chr1 | 12949975 | 12971935 | 2064 |
| novel.1716 | -3.88614 | 4.71E-12 | 8.23E-10 | Chr2 | 8645091 | 8648726 | 3060 |
| novel.133 | -8.87779 | 5.01E-12 | 8.69E-10 | Chr1 | 17370037 | 17371290 | 495 |
| MsG0880045651.01 | 9.371237 | 5.13E-12 | 8.85E-10 | Chr8 | 62178961 | 62182585 | 2011 |
| MsG0880044283.01 | 7.966057 | 5.17E-12 | 8.87E-10 | Chr8 | 39391996 | 39406469 | 2775 |
| novel.6366 | 9.059538 | 5.27E-12 | 8.99E-10 | Chr6 | 33520298 | 33531838 | 2720 |
| MsG0180000941.01 | -9.08357 | 6.29E-12 | 1.06E-09 | Chr1 | 13493108 | 13494871 | 1419 |
| MsG0680033014.01 | -6.75148 | 6.29E-12 | 1.06E-09 | Chr6 | 54784001 | 54784381 | 381 |
| MsG0380015000.01 | -4.25802 | 6.32E-12 | 1.06E-09 | Chr3 | 63755764 | 63763547 | 4895 |
| MsG0480023751.01 | 8.934252 | 6.44E-12 | 1.07E-09 | Chr4 | 89385695 | 89387287 | 650 |
| novel.144 | 9.347095 | 6.56E-12 | 1.09E-09 | Chr1 | 18691816 | 18693366 | 1551 |
| novel.6117 | 9.245308 | 6.81E-12 | 1.12E-09 | Chr6 | 1.12E+08 | 1.12E+08 | 891 |
| novel.2910 | 8.844041 | 7.59E-12 | 1.24E-09 | Chr3 | 50310195 | 50321260 | 895 |
| MsG0680032788.01 | -5.18012 | 7.68E-12 | 1.25E-09 | Chr6 | 49693117 | 49694691 | 1575 |
| novel.3826 | -5.72924 | 7.69E-12 | 1.25E-09 | Chr4 | 15621509 | 15625839 | 898 |
| MsG0280009888.01 | 4.518635 | 8.22E-12 | 1.32E-09 | Chr2 | 60146342 | 60153241 | 1192 |
| MsG0580024842.01 | 4.9534 | 8.25E-12 | 1.32E-09 | Chr5 | 10628714 | 10632858 | 3934 |
| MsG0880046764.01 | 2.754978 | 8.28E-12 | 1.32E-09 | Chr8 | 77808324 | 77810346 | 701 |
| MsG0880043648.01 | -6.3141 | 1.01E-11 | 1.60E-09 | Chr8 | 28422696 | 28424336 | 579 |
| MsG0780040674.01 | -6.85264 | 1.06E-11 | 1.68E-09 | Chr7 | 80951598 | 80952538 | 729 |
| MsG0680030975.01 | 3.139969 | 1.15E-11 | 1.81E-09 | Chr6 | 12381394 | 12391875 | 4203 |
| MsG0680035822.01 | -9.35149 | 1.20E-11 | 1.88E-09 | Chr6 | 1.13E+08 | 1.13E+08 | 1083 |
| MsG0580028268.01 | 10.20432 | 1.29E-11 | 2.01E-09 | Chr5 | 77546017 | 77547513 | 1497 |
| MsG0580026422.01 | -9.2599 | 1.33E-11 | 2.05E-09 | Chr5 | 36982587 | 36982967 | 381 |
| MsG0780036187.01 | 9.078415 | 1.33E-11 | 2.05E-09 | Chr7 | 4459215 | 4464465 | 3402 |
| MsG0280006870.01 | 5.089186 | 1.37E-11 | 2.10E-09 | Chr2 | 7417055 | 7419183 | 735 |
| MsG0880044324.01 | -9.04787 | 1.43E-11 | 2.17E-09 | Chr8 | 39979892 | 39980624 | 549 |
| MsG0780037656.01 | 3.44472 | 1.51E-11 | 2.28E-09 | Chr7 | 30373752 | 30376463 | 832 |
| MsG0880043597.01 | -8.84163 | 1.52E-11 | 2.29E-09 | Chr8 | 27473551 | 27475946 | 1704 |
| MsG0280007421.01 | -6.91801 | 1.57E-11 | 2.36E-09 | Chr2 | 15176666 | 15176941 | 276 |
| MsG0180004941.01 | -5.20039 | 1.63E-11 | 2.43E-09 | Chr1 | 84712640 | 84713614 | 321 |
| novel.750 | 4.916961 | 1.73E-11 | 2.57E-09 | Chr1 | 22514464 | 22516970 | 1290 |
| novel.4607 | -5.30255 | 1.76E-11 | 2.60E-09 | Chr5 | 58801362 | 58804277 | 2472 |
| MsG0880042913.01 | 2.656655 | 1.85E-11 | 2.72E-09 | Chr8 | 15819670 | 15823727 | 1018 |
| novel.2382 | 9.643451 | 1.91E-11 | 2.79E-09 | Chr3 | 55173891 | 55176780 | 2253 |
| MsG0880046767.01 | 5.557552 | 2.25E-11 | 3.25E-09 | Chr8 | 77829029 | 77829919 | 429 |
| MsG0580025399.01 | -3.10668 | 2.26E-11 | 3.25E-09 | Chr5 | 18044590 | 18048456 | 3059 |
| MsG0780040959.01 | 8.866656 | 2.27E-11 | 3.25E-09 | Chr7 | 84832188 | 84834591 | 869 |
| MsG0280007359.01 | -4.21855 | 2.27E-11 | 3.25E-09 | Chr2 | 14481114 | 14484359 | 2715 |
| novel.2489 | -5.2725 | 2.40E-11 | 3.41E-09 | Chr3 | 76576317 | 76577558 | 1242 |
| MsG0280010301.01 | 10.11873 | 2.40E-11 | 3.41E-09 | Chr2 | 67607764 | 67615431 | 1185 |
| novel.6578 | 8.812806 | 2.43E-11 | 3.43E-09 | Chr6 | 77606596 | 77609345 | 2176 |
| MsG0580025807.01 | -2.89769 | 2.71E-11 | 3.82E-09 | Chr5 | 24499071 | 24509647 | 1749 |
| novel.5091 | 9.346674 | 2.78E-11 | 3.89E-09 | Chr5 | 36117359 | 36120797 | 2074 |
| MsG0480019427.01 | 6.316296 | 2.79E-11 | 3.89E-09 | Chr4 | 20202020 | 20203474 | 1455 |
| novel.8233 | -9.43479 | 2.91E-11 | 4.04E-09 | Chr8 | 82987270 | 82988617 | 1177 |
| novel.8346 | -9.34151 | 2.99E-11 | 4.13E-09 | Chr8 | 9050616 | 9051268 | 482 |
| novel.7884 | -7.86963 | 3.09E-11 | 4.24E-09 | Chr8 | 17354573 | 17356785 | 690 |
| MsG0880046204.01 | 5.764947 | 3.15E-11 | 4.31E-09 | Chr8 | 70494106 | 70501246 | 2836 |
| novel.7412 | 9.049755 | 3.29E-11 | 4.48E-09 | Chr7 | 23862143 | 23864844 | 784 |
| MsG0880044564.01 | 6.095177 | 3.60E-11 | 4.88E-09 | Chr8 | 44582106 | 44582542 | 345 |
| MsG0380014625.01 | -6.64079 | 3.79E-11 | 5.11E-09 | Chr3 | 57449851 | 57452581 | 1523 |
| novel.2560 | 3.410587 | 3.84E-11 | 5.15E-09 | Chr3 | 86219082 | 86225575 | 3512 |
| MsG0680035496.01 | -4.5038 | 4.06E-11 | 5.43E-09 | Chr6 | 1.07E+08 | 1.07E+08 | 1929 |
| novel.2047 | -8.8992 | 4.10E-11 | 5.45E-09 | Chr2 | 76002598 | 76003568 | 806 |
| MsG0780040083.01 | -8.48254 | 4.12E-11 | 5.46E-09 | Chr7 | 72900457 | 72903350 | 1039 |
| MsG0380013999.01 | -9.66531 | 4.37E-11 | 5.76E-09 | Chr3 | 48616018 | 48616236 | 219 |
| MsG0480019152.01 | 8.718485 | 4.48E-11 | 5.88E-09 | Chr4 | 15135235 | 15136756 | 708 |
| novel.6097 | -4.61997 | 4.51E-11 | 5.89E-09 | Chr6 | 1.08E+08 | 1.08E+08 | 2836 |
| MsG0480022947.01 | 5.126453 | 4.56E-11 | 5.94E-09 | Chr4 | 78951727 | 78954311 | 1029 |
| MsG0480019107.01 | -5.65421 | 5.24E-11 | 6.77E-09 | Chr4 | 14677113 | 14683155 | 4494 |
| novel.8021 | -3.99112 | 5.24E-11 | 6.77E-09 | Chr8 | 43457736 | 43459908 | 638 |
| MsG0580025804.01 | -3.64982 | 5.32E-11 | 6.82E-09 | Chr5 | 24473981 | 24478124 | 1509 |
| MsG0680035010.01 | -8.72874 | 5.33E-11 | 6.82E-09 | Chr6 | 97741797 | 97748747 | 3244 |
| MsG0580025521.01 | 8.676529 | 5.44E-11 | 6.92E-09 | Chr5 | 19954972 | 19958090 | 1163 |
| novel.5317 | -8.62536 | 5.73E-11 | 7.27E-09 | Chr5 | 82484842 | 82487691 | 1663 |
| MsG0480019065.01 | -2.66992 | 5.86E-11 | 7.39E-09 | Chr4 | 14072610 | 14083566 | 5339 |
| MsG0780036188.01 | 8.677887 | 5.88E-11 | 7.39E-09 | Chr7 | 4466132 | 4479025 | 4488 |
| novel.649 | -4.67312 | 6.01E-11 | 7.52E-09 | Chr1 | 6935963 | 6942326 | 1209 |
| MsG0680035448.01 | 5.618699 | 6.05E-11 | 7.54E-09 | Chr6 | 1.06E+08 | 1.06E+08 | 924 |
| novel.8971 | -8.64655 | 6.22E-11 | 7.73E-09 | contig384end | 20830 | 24501 | 3672 |
| MsG0580030145.01 | -3.69825 | 6.31E-11 | 7.80E-09 | Chr5 | 1.07E+08 | 1.07E+08 | 1401 |
| novel.1952 | 8.994177 | 6.66E-11 | 8.20E-09 | Chr2 | 61370722 | 61376785 | 2335 |
| MsG0680034531.01 | -8.73321 | 6.85E-11 | 8.39E-09 | Chr6 | 89868964 | 89874096 | 834 |
| novel.6786 | 3.09978 | 6.88E-11 | 8.41E-09 | Chr6 | 1.14E+08 | 1.14E+08 | 800 |
| MsG0780036854.01 | -5.14339 | 7.12E-11 | 8.66E-09 | Chr7 | 15115192 | 15124156 | 1935 |
| MsG0080048889.01 | 3.155846 | 7.23E-11 | 8.76E-09 | contig515end | 3956 | 6104 | 2063 |
| novel.7562 | -8.80838 | 7.48E-11 | 9.03E-09 | Chr7 | 64003049 | 64005434 | 1120 |
| MsG0280006439.01 | -5.67132 | 8.69E-11 | 1.04E-08 | Chr2 | 1958535 | 1961750 | 1257 |
| MsG0280007814.01 | -2.65331 | 8.97E-11 | 1.07E-08 | Chr2 | 21089921 | 21097101 | 702 |
| MsG0680032003.01 | -3.07947 | 9.06E-11 | 1.08E-08 | Chr6 | 31757657 | 31760827 | 3171 |
| MsG0280006784.01 | 8.75793 | 9.30E-11 | 1.10E-08 | Chr2 | 6171995 | 6177914 | 742 |
| MsG0780038860.01 | -3.90602 | 9.66E-11 | 1.14E-08 | Chr7 | 54235672 | 54235932 | 261 |
| MsG0380014378.01 | -8.57685 | 9.71E-11 | 1.14E-08 | Chr3 | 53902433 | 53902852 | 420 |
| novel.3525 | -8.96131 | 9.98E-11 | 1.17E-08 | Chr4 | 59797568 | 59800116 | 1874 |
| MsG0280009648.01 | -2.51619 | 1.02E-10 | 1.19E-08 | Chr2 | 55767593 | 55768495 | 903 |
| MsG0680034699.01 | 3.277496 | 1.12E-10 | 1.30E-08 | Chr6 | 93048844 | 93054057 | 2818 |
| novel.4478 | 9.317336 | 1.18E-10 | 1.37E-08 | Chr5 | 30094567 | 30096670 | 307 |
| MsG0880044037.01 | -3.76643 | 1.20E-10 | 1.39E-08 | Chr8 | 34650614 | 34653100 | 1456 |
| MsG0780036443.01 | 4.815201 | 1.22E-10 | 1.40E-08 | Chr7 | 8203565 | 8205133 | 613 |
| novel.1314 | 6.396818 | 1.22E-10 | 1.40E-08 | Chr2 | 24703099 | 24705440 | 1598 |
| novel.7114 | 3.4631 | 1.25E-10 | 1.42E-08 | Chr7 | 70636319 | 70645438 | 2663 |
| novel.8714 | -8.51848 | 1.26E-10 | 1.43E-08 | Chr8 | 80760665 | 80764013 | 1389 |
| MsG0380011840.01 | 3.362972 | 1.34E-10 | 1.52E-08 | Chr3 | 6169156 | 6183938 | 2897 |
| MsG0580025514.01 | 8.705863 | 1.43E-10 | 1.61E-08 | Chr5 | 19862143 | 19863207 | 204 |
| novel.8079 | 8.618161 | 1.43E-10 | 1.61E-08 | Chr8 | 55960025 | 55961743 | 1163 |
| MsG0880041996.01 | -2.02665 | 1.47E-10 | 1.64E-08 | Chr8 | 2288214 | 2292808 | 2907 |
| MsG0880043085.01 | 8.409732 | 1.48E-10 | 1.66E-08 | Chr8 | 18718308 | 18721561 | 2294 |
| MsG0680035895.01 | -8.50479 | 1.65E-10 | 1.83E-08 | Chr6 | 1.14E+08 | 1.14E+08 | 8007 |
| novel.5055 | -8.58225 | 1.67E-10 | 1.85E-08 | Chr5 | 28490392 | 28493834 | 1062 |
| MsG0080048354.01 | 8.937276 | 1.70E-10 | 1.87E-08 | contig280end | 13765 | 14130 | 366 |
| MsG0780039119.01 | -5.35598 | 1.94E-10 | 2.13E-08 | Chr7 | 58238619 | 58246197 | 3199 |
| MsG0680030863.01 | 8.888899 | 2.13E-10 | 2.33E-08 | Chr6 | 10612210 | 10612368 | 159 |
| MsG0780036562.01 | -4.23898 | 2.14E-10 | 2.34E-08 | Chr7 | 9845428 | 9846870 | 1443 |
| novel.6365 | -5.20577 | 2.16E-10 | 2.35E-08 | Chr6 | 33408919 | 33410206 | 1261 |
| novel.2490 | -8.83519 | 2.28E-10 | 2.47E-08 | Chr3 | 77198640 | 77201395 | 2615 |
| MsG0280008198.01 | 3.196595 | 2.30E-10 | 2.49E-08 | Chr2 | 27065403 | 27078052 | 1545 |
| MsG0680035454.01 | -3.1554 | 2.32E-10 | 2.50E-08 | Chr6 | 1.06E+08 | 1.06E+08 | 1911 |
| MsG0680030639.01 | 5.752807 | 2.51E-10 | 2.69E-08 | Chr6 | 6495286 | 6496042 | 624 |
| novel.309 | 6.642722 | 2.55E-10 | 2.72E-08 | Chr1 | 58018610 | 58021595 | 1086 |
| novel.6456 | 8.448637 | 2.57E-10 | 2.74E-08 | Chr6 | 51366600 | 51370308 | 1013 |
| novel.5635 | 8.436212 | 2.74E-10 | 2.91E-08 | Chr6 | 19929493 | 19932249 | 1420 |
| novel.4867 | 8.9697 | 2.84E-10 | 3.00E-08 | Chr5 | 1.04E+08 | 1.04E+08 | 4417 |
| MsG0880045419.01 | -9.0425 | 2.88E-10 | 3.03E-08 | Chr8 | 58956947 | 58958054 | 123 |
| MsG0380011703.01 | -3.76926 | 3.04E-10 | 3.19E-08 | Chr3 | 3791683 | 3802347 | 1758 |
| MsG0680030953.01 | -8.39351 | 3.16E-10 | 3.30E-08 | Chr6 | 12116468 | 12123235 | 5296 |
| novel.2200 | 3.782879 | 3.20E-10 | 3.33E-08 | Chr3 | 13169336 | 13171598 | 689 |
| MsG0880046271.01 | -6.44155 | 3.28E-10 | 3.40E-08 | Chr8 | 71367054 | 71367665 | 612 |
| novel.2941 | 8.558488 | 3.31E-10 | 3.42E-08 | Chr3 | 58546248 | 58548887 | 2050 |
| novel.8816 | 8.46199 | 3.48E-10 | 3.59E-08 | contig152end | 94869 | 95878 | 619 |
| novel.6191 | 8.49586 | 3.86E-10 | 3.96E-08 | Chr6 | 8053860 | 8058583 | 925 |
| novel.3869 | 8.383758 | 4.14E-10 | 4.24E-08 | Chr4 | 23072585 | 23074016 | 720 |
| novel.3822 | -8.35315 | 4.20E-10 | 4.28E-08 | Chr4 | 14083632 | 14087090 | 1788 |
| novel.3830 | 8.211129 | 4.30E-10 | 4.37E-08 | Chr4 | 16582565 | 16584853 | 1305 |
| novel.4999 | 4.354686 | 4.52E-10 | 4.58E-08 | Chr5 | 15655702 | 15660613 | 2341 |
| novel.8947 | 5.055464 | 4.55E-10 | 4.59E-08 | contig341end | 19576 | 23195 | 1636 |
| MsG0780036847.01 | -8.96384 | 4.71E-10 | 4.73E-08 | Chr7 | 14975822 | 14977321 | 1059 |
| MsG0880045965.01 | -2.92104 | 4.88E-10 | 4.90E-08 | Chr8 | 67017637 | 67019737 | 555 |
| MsG0680032093.01 | -4.3214 | 5.05E-10 | 5.04E-08 | Chr6 | 33512109 | 33519109 | 3384 |
| MsG0380015797.01 | 3.624286 | 5.06E-10 | 5.04E-08 | Chr3 | 75134420 | 75138008 | 642 |
| novel.1336 | 8.397187 | 5.11E-10 | 5.07E-08 | Chr2 | 29220635 | 29222425 | 634 |
| novel.1715 | -5.55849 | 5.37E-10 | 5.31E-08 | Chr2 | 8474023 | 8479630 | 2205 |
| MsG0680031771.01 | 2.874803 | 5.39E-10 | 5.31E-08 | Chr6 | 26461625 | 26463210 | 609 |
| MsG0380013593.01 | 9.230821 | 5.57E-10 | 5.47E-08 | Chr3 | 41349209 | 41350048 | 840 |
| novel.963 | 8.523154 | 6.01E-10 | 5.88E-08 | Chr1 | 72998862 | 73002661 | 1364 |
| novel.1894 | 9.441606 | 6.03E-10 | 5.88E-08 | Chr2 | 44170351 | 44176806 | 2667 |
| MsG0780038585.01 | 3.055752 | 6.06E-10 | 5.90E-08 | Chr7 | 49247199 | 49248969 | 688 |
| MsG0580026917.01 | -5.16889 | 6.09E-10 | 5.91E-08 | Chr5 | 48085675 | 48087183 | 1509 |
| MsG0680032009.01 | 8.796451 | 6.60E-10 | 6.38E-08 | Chr6 | 31854968 | 31858076 | 1162 |
| novel.8834 | 8.596719 | 6.70E-10 | 6.46E-08 | contig172end | 5698 | 6859 | 1162 |
| novel.7371 | 5.353008 | 6.79E-10 | 6.52E-08 | Chr7 | 16613580 | 16615081 | 625 |
| novel.3420 | -8.29685 | 7.42E-10 | 7.10E-08 | Chr4 | 38279668 | 38280732 | 1065 |
| MsG0180003113.01 | 4.487781 | 7.49E-10 | 7.15E-08 | Chr1 | 56811967 | 56819492 | 587 |
| MsG0580027864.01 | -4.67121 | 7.55E-10 | 7.18E-08 | Chr5 | 69866539 | 69867488 | 729 |
| MsG0580027139.01 | 8.64205 | 7.71E-10 | 7.31E-08 | Chr5 | 54269581 | 54270692 | 850 |
| MsG0780041790.01 | 2.279458 | 7.81E-10 | 7.38E-08 | Chr7 | 95180596 | 95180940 | 345 |
| MsG0280008042.01 | -2.31348 | 7.86E-10 | 7.41E-08 | Chr2 | 24674177 | 24681762 | 931 |
| novel.909 | 9.599975 | 7.95E-10 | 7.47E-08 | Chr1 | 60928858 | 60933156 | 1294 |
| novel.4815 | 8.392352 | 8.23E-10 | 7.70E-08 | Chr5 | 97920806 | 97924022 | 3217 |
| novel.806 | -8.445 | 8.42E-10 | 7.86E-08 | Chr1 | 32799260 | 32800204 | 945 |
| MsG0080047963.01 | 7.229355 | 8.61E-10 | 8.01E-08 | contig152end | 411542 | 421148 | 870 |
| MsG0580029437.01 | -8.37899 | 8.78E-10 | 8.15E-08 | Chr5 | 96983830 | 96992625 | 1932 |
| MsG0480020925.01 | -8.03932 | 9.43E-10 | 8.72E-08 | Chr4 | 50631395 | 50631667 | 273 |
| MsG0180000980.01 | 4.718816 | 9.47E-10 | 8.73E-08 | Chr1 | 14042475 | 14046458 | 505 |
| MsG0380017824.01 | -8.8711 | 9.56E-10 | 8.79E-08 | Chr3 | 1.01E+08 | 1.01E+08 | 735 |
| MsG0580025812.01 | -3.40927 | 9.91E-10 | 9.07E-08 | Chr5 | 24554437 | 24561296 | 1997 |
| MsG0180004122.01 | -11.9919 | 1.01E-09 | 9.20E-08 | Chr1 | 73703796 | 73705538 | 1641 |
| novel.4418 | -8.2367 | 1.06E-09 | 9.64E-08 | Chr5 | 21935582 | 21939477 | 995 |
| novel.6218 | 2.316183 | 1.07E-09 | 9.68E-08 | Chr6 | 12176642 | 12184998 | 4238 |
| MsG0080048756.01 | -8.21598 | 1.12E-09 | 1.01E-07 | contig424end | 10953 | 12753 | 957 |
| novel.7037 | 8.505177 | 1.12E-09 | 1.01E-07 | Chr7 | 55743682 | 55745178 | 1497 |
| novel.6239 | 8.256057 | 1.13E-09 | 1.01E-07 | Chr6 | 14488371 | 14489201 | 831 |
| novel.4848 | 8.202365 | 1.14E-09 | 1.02E-07 | Chr5 | 1.01E+08 | 1.01E+08 | 586 |
| novel.7874 | 8.489701 | 1.14E-09 | 1.02E-07 | Chr8 | 14281648 | 14283800 | 1461 |
| MsG0380012033.01 | -3.35579 | 1.17E-09 | 1.04E-07 | Chr3 | 9845066 | 9851439 | 3120 |
| MsG0780038461.01 | 6.215329 | 1.19E-09 | 1.06E-07 | Chr7 | 47150266 | 47154188 | 987 |
| novel.2333 | 3.698095 | 1.22E-09 | 1.08E-07 | Chr3 | 41535660 | 41539755 | 1604 |
| MsG0780039104.01 | -5.11267 | 1.22E-09 | 1.08E-07 | Chr7 | 57965841 | 57968952 | 1278 |
| MsG0380011657.01 | -2.82697 | 1.30E-09 | 1.14E-07 | Chr3 | 3108093 | 3110266 | 1533 |
| MsG0780040430.01 | -5.14529 | 1.30E-09 | 1.14E-07 | Chr7 | 77417650 | 77418856 | 858 |
| MsG0380015680.01 | 4.580563 | 1.33E-09 | 1.16E-07 | Chr3 | 73659767 | 73666257 | 1933 |
| MsG0080048027.01 | -4.41161 | 1.36E-09 | 1.19E-07 | contig175end | 9564 | 10114 | 447 |
| novel.132 | -8.61117 | 1.53E-09 | 1.33E-07 | Chr1 | 17367473 | 17369226 | 714 |
| novel.5703 | 8.727168 | 1.54E-09 | 1.33E-07 | Chr6 | 33033084 | 33034564 | 577 |
| MsG0280007686.01 | -2.56437 | 1.54E-09 | 1.33E-07 | Chr2 | 19198463 | 19201765 | 729 |
| novel.6425 | 8.585253 | 1.54E-09 | 1.33E-07 | Chr6 | 44958555 | 44960051 | 658 |
| MsG0580029893.01 | 2.648269 | 1.61E-09 | 1.39E-07 | Chr5 | 1.04E+08 | 1.04E+08 | 677 |
| MsG0180001414.01 | -4.67823 | 1.63E-09 | 1.40E-07 | Chr1 | 20786417 | 20801755 | 6684 |
| novel.5785 | 9.089227 | 1.68E-09 | 1.44E-07 | Chr6 | 52303583 | 52305859 | 2277 |
| MsG0180001148.01 | -4.11054 | 1.70E-09 | 1.45E-07 | Chr1 | 16579972 | 16581603 | 1221 |
| MsG0380016576.01 | 2.525022 | 1.70E-09 | 1.45E-07 | Chr3 | 85323387 | 85326567 | 1285 |
| novel.7808 | 8.141635 | 1.74E-09 | 1.47E-07 | Chr8 | 3125280 | 3129316 | 1167 |
| novel.3682 | 4.325153 | 1.79E-09 | 1.51E-07 | Chr4 | 84646406 | 84647186 | 678 |
| novel.3179 | 8.900603 | 1.80E-09 | 1.51E-07 | Chr3 | 99177411 | 99183202 | 2204 |
| novel.6229 | 8.552067 | 1.91E-09 | 1.60E-07 | Chr6 | 12890281 | 12892578 | 1541 |
| MsG0780039370.01 | 8.207249 | 1.92E-09 | 1.60E-07 | Chr7 | 62921927 | 62923273 | 612 |
| MsG0880044521.01 | 2.785987 | 1.92E-09 | 1.60E-07 | Chr8 | 43687317 | 43693649 | 918 |
| novel.8991 | -5.1425 | 1.93E-09 | 1.61E-07 | contig408end | 195682 | 196652 | 568 |
| MsG0780037601.01 | 4.347589 | 1.98E-09 | 1.64E-07 | Chr7 | 29108954 | 29132298 | 8694 |
| MsG0480018302.01 | 2.947028 | 1.99E-09 | 1.65E-07 | Chr4 | 3124061 | 3129474 | 1858 |
| novel.7016 | 8.628093 | 2.06E-09 | 1.70E-07 | Chr7 | 49737791 | 49744303 | 6433 |
| novel.2513 | 2.441319 | 2.27E-09 | 1.87E-07 | Chr3 | 80038946 | 80042206 | 3261 |
| novel.6688 | -8.73199 | 2.34E-09 | 1.92E-07 | Chr6 | 1.01E+08 | 1.01E+08 | 2275 |
| MsG0880045358.01 | 3.762312 | 2.41E-09 | 1.98E-07 | Chr8 | 58075399 | 58075867 | 318 |
| MsG0480019285.01 | 2.784325 | 2.51E-09 | 2.05E-07 | Chr4 | 17750068 | 17750448 | 381 |
| novel.710 | -8.13813 | 2.55E-09 | 2.08E-07 | Chr1 | 15127370 | 15129147 | 413 |
| MsG0680030335.01 | 8.714839 | 2.60E-09 | 2.11E-07 | Chr6 | 781119 | 786819 | 2094 |
| MsG0680030969.01 | 8.118935 | 2.74E-09 | 2.22E-07 | Chr6 | 12341097 | 12346458 | 695 |
| MsG0780036105.01 | 3.744006 | 2.92E-09 | 2.36E-07 | Chr7 | 2663941 | 2667063 | 2712 |
| MsG0280010553.01 | 8.43096 | 3.07E-09 | 2.48E-07 | Chr2 | 71387028 | 71389311 | 1726 |
| novel.880 | 3.227557 | 3.09E-09 | 2.49E-07 | Chr1 | 54592270 | 54593284 | 660 |
| MsG0880047201.01 | -6.2016 | 3.10E-09 | 2.49E-07 | Chr8 | 83230704 | 83231161 | 366 |
| MsG0380015237.01 | 3.140702 | 3.11E-09 | 2.49E-07 | Chr3 | 67199269 | 67200071 | 663 |
| MsG0080048387.01 | -4.464 | 3.13E-09 | 2.50E-07 | contig297end | 10841 | 14796 | 786 |
| MsG0580026162.01 | -4.74989 | 3.19E-09 | 2.54E-07 | Chr5 | 31600349 | 31608680 | 5058 |
| MsG0880046771.01 | 5.483701 | 3.20E-09 | 2.54E-07 | Chr8 | 77872715 | 77875153 | 2439 |
| MsG0280007327.01 | 4.777542 | 3.23E-09 | 2.56E-07 | Chr2 | 13671164 | 13672165 | 662 |
| novel.7921 | 3.619049 | 3.39E-09 | 2.68E-07 | Chr8 | 22726724 | 22728111 | 1271 |
| novel.4174 | 8.547149 | 3.44E-09 | 2.71E-07 | Chr4 | 80850198 | 80852113 | 785 |
| novel.1620 | -8.06874 | 3.54E-09 | 2.78E-07 | Chr2 | 82312517 | 82317521 | 1257 |
| novel.3587 | -2.68523 | 3.55E-09 | 2.78E-07 | Chr4 | 72257918 | 72261444 | 2754 |
| MsG0480021782.01 | -4.18799 | 3.56E-09 | 2.78E-07 | Chr4 | 64212578 | 64213478 | 747 |
| MsG0580024737.01 | -5.03733 | 3.68E-09 | 2.86E-07 | Chr5 | 9002988 | 9008894 | 1779 |
| novel.3104 | -3.8814 | 3.68E-09 | 2.86E-07 | Chr3 | 87583856 | 87586138 | 1136 |
| MsG0880047419.01 | 6.176977 | 3.82E-09 | 2.96E-07 | Chr8 | 86020786 | 86024992 | 870 |
| MsG0380013595.01 | 8.485749 | 3.83E-09 | 2.96E-07 | Chr3 | 41381127 | 41381969 | 843 |
| MsG0580026718.01 | 3.838309 | 3.87E-09 | 2.98E-07 | Chr5 | 42800882 | 42803587 | 859 |
| novel.8408 | -8.49702 | 3.90E-09 | 3.00E-07 | Chr8 | 20601667 | 20603452 | 1786 |
| MsG0680032686.01 | 6.014275 | 3.94E-09 | 3.02E-07 | Chr6 | 47610561 | 47611106 | 546 |
| MsG0680032451.01 | -2.7528 | 3.95E-09 | 3.02E-07 | Chr6 | 41583472 | 41590098 | 1709 |
| MsG0780040961.01 | 8.632946 | 4.08E-09 | 3.10E-07 | Chr7 | 84858368 | 84869795 | 6321 |
| novel.8276 | -5.9718 | 4.08E-09 | 3.10E-07 | Chr8 | 88931458 | 88935073 | 1824 |
| MsG0180003142.01 | 3.63635 | 4.14E-09 | 3.14E-07 | Chr1 | 57212956 | 57215130 | 906 |
| MsG0780038803.01 | 2.842808 | 4.17E-09 | 3.15E-07 | Chr7 | 53261550 | 53263217 | 1413 |
| novel.4066 | 8.434868 | 4.28E-09 | 3.24E-07 | Chr4 | 65651843 | 65653469 | 843 |
| novel.4019 | 7.98947 | 4.43E-09 | 3.34E-07 | Chr4 | 57213539 | 57219089 | 1539 |
| novel.2814 | 4.309492 | 4.53E-09 | 3.40E-07 | Chr3 | 26483440 | 26488498 | 1614 |
| MsG0880042611.01 | 4.901317 | 4.62E-09 | 3.46E-07 | Chr8 | 11458785 | 11459330 | 546 |
| MsG0580025873.01 | -5.23372 | 4.67E-09 | 3.49E-07 | Chr5 | 25662130 | 25663257 | 1128 |
| MsG0880044596.01 | -8.38655 | 4.68E-09 | 3.49E-07 | Chr8 | 45444121 | 45448427 | 1673 |
| MsG0780041377.01 | 6.90124 | 4.71E-09 | 3.50E-07 | Chr7 | 90219951 | 90236237 | 2517 |
| novel.3456 | -3.7475 | 4.73E-09 | 3.51E-07 | Chr4 | 47827998 | 47831098 | 3101 |
| MsG0580025523.01 | 2.842305 | 4.75E-09 | 3.51E-07 | Chr5 | 19972941 | 19974330 | 747 |
| MsG0380014813.01 | 5.623157 | 4.85E-09 | 3.58E-07 | Chr3 | 60536528 | 60546953 | 5187 |
| MsG0580028720.01 | 8.0321 | 4.91E-09 | 3.61E-07 | Chr5 | 85327714 | 85333333 | 1128 |
| novel.2997 | -7.97756 | 4.93E-09 | 3.62E-07 | Chr3 | 70719729 | 70721329 | 781 |
| MsG0280009636.01 | -5.33625 | 4.97E-09 | 3.64E-07 | Chr2 | 55685200 | 55688627 | 1907 |
| MsG0780036634.01 | -4.88895 | 5.14E-09 | 3.76E-07 | Chr7 | 10982883 | 10985711 | 1053 |
| MsG0680032895.01 | -4.30515 | 5.20E-09 | 3.80E-07 | Chr6 | 52327284 | 52334431 | 2376 |
| MsG0380011656.01 | -3.90575 | 5.50E-09 | 4.00E-07 | Chr3 | 3096680 | 3103616 | 1947 |
| MsG0580029891.01 | 7.965687 | 5.51E-09 | 4.00E-07 | Chr5 | 1.04E+08 | 1.04E+08 | 312 |
| MsG0580028829.01 | 4.084101 | 6.15E-09 | 4.45E-07 | Chr5 | 87121396 | 87126600 | 3168 |
| novel.6120 | -8.04087 | 6.17E-09 | 4.46E-07 | Chr6 | 1.13E+08 | 1.13E+08 | 1208 |
| MsG0580024933.01 | 7.936881 | 6.20E-09 | 4.47E-07 | Chr5 | 11811036 | 11812472 | 1437 |
| MsG0880045796.01 | -4.81804 | 6.25E-09 | 4.49E-07 | Chr8 | 64591406 | 64594584 | 786 |
| novel.213 | 3.238711 | 6.37E-09 | 4.56E-07 | Chr1 | 31426196 | 31426735 | 480 |
| MsG0180004312.01 | 3.139318 | 6.38E-09 | 4.56E-07 | Chr1 | 76344717 | 76347268 | 1207 |
| MsG0180003161.01 | 7.981771 | 6.88E-09 | 4.91E-07 | Chr1 | 57781458 | 57781754 | 297 |
| novel.1447 | 7.899968 | 7.03E-09 | 5.01E-07 | Chr2 | 52055955 | 52058548 | 707 |
| novel.7179 | 2.501368 | 7.09E-09 | 5.03E-07 | Chr7 | 79699635 | 79703047 | 1379 |
| novel.2338 | 8.040265 | 7.18E-09 | 5.08E-07 | Chr3 | 43358687 | 43360318 | 351 |
| novel.6505 | 3.242891 | 7.22E-09 | 5.10E-07 | Chr6 | 62849888 | 62853481 | 1121 |
| novel.4501 | -5.93003 | 7.37E-09 | 5.20E-07 | Chr5 | 35780267 | 35783488 | 767 |
| MsG0380011870.01 | -8.0253 | 7.43E-09 | 5.23E-07 | Chr3 | 6680960 | 6685929 | 1647 |
| MsG0180005650.01 | -5.05776 | 7.73E-09 | 5.42E-07 | Chr1 | 94486855 | 94492700 | 1416 |
| novel.1732 | 8.170789 | 7.80E-09 | 5.46E-07 | Chr2 | 11749528 | 11752791 | 2614 |
| MsG0280010863.01 | 8.234348 | 7.83E-09 | 5.47E-07 | Chr2 | 76043351 | 76048888 | 1347 |
| novel.1558 | 7.88905 | 7.92E-09 | 5.52E-07 | Chr2 | 75718636 | 75721528 | 2373 |
| MsG0180001208.01 | 4.523808 | 8.05E-09 | 5.59E-07 | Chr1 | 17402944 | 17407714 | 1686 |
| novel.3222 | 2.081541 | 8.09E-09 | 5.61E-07 | Chr4 | 828248 | 831310 | 1839 |
| novel.991 | 1.905111 | 8.25E-09 | 5.70E-07 | Chr1 | 79596970 | 79599916 | 2224 |
| novel.8831 | -8.20201 | 8.27E-09 | 5.71E-07 | contig168end | 5536 | 6542 | 754 |
| MsG0580025377.01 | 3.577306 | 8.31E-09 | 5.72E-07 | Chr5 | 17670788 | 17675345 | 1249 |
| MsG0780041225.01 | -4.00103 | 8.36E-09 | 5.74E-07 | Chr7 | 88450576 | 88453085 | 450 |
| MsG0580026281.01 | -2.72908 | 8.61E-09 | 5.89E-07 | Chr5 | 33916087 | 33919544 | 934 |
| MsG0680030823.01 | -7.97179 | 8.62E-09 | 5.89E-07 | Chr6 | 9953477 | 9954281 | 543 |
| MsG0780038984.01 | 8.093871 | 8.63E-09 | 5.89E-07 | Chr7 | 56357595 | 56389846 | 5100 |
| MsG0880043721.01 | -6.90326 | 8.98E-09 | 6.11E-07 | Chr8 | 29618874 | 29625806 | 3571 |
| MsG0380014464.01 | 2.126907 | 8.99E-09 | 6.11E-07 | Chr3 | 55210008 | 55211945 | 1644 |
| novel.1959 | 5.780925 | 9.04E-09 | 6.13E-07 | Chr2 | 62372140 | 62373276 | 835 |
| MsG0680034206.01 | 7.878115 | 9.63E-09 | 6.50E-07 | Chr6 | 83602564 | 83602971 | 408 |
| MsG0180004088.01 | 4.177835 | 9.64E-09 | 6.50E-07 | Chr1 | 73119655 | 73130002 | 3439 |
| novel.5527 | 5.681738 | 9.77E-09 | 6.57E-07 | Chr6 | 3655973 | 3658976 | 1205 |
| novel.1597 | 6.690758 | 1.01E-08 | 6.75E-07 | Chr2 | 79795703 | 79799545 | 1593 |
| novel.8599 | -8.19837 | 1.01E-08 | 6.75E-07 | Chr8 | 58790388 | 58797444 | 3328 |
| MsG0280007911.01 | -3.71335 | 1.01E-08 | 6.75E-07 | Chr2 | 22807138 | 22813071 | 1304 |
| novel.5836 | -3.98598 | 1.02E-08 | 6.82E-07 | Chr6 | 66628395 | 66633069 | 2120 |
| MsG0180000574.01 | -5.84077 | 1.05E-08 | 6.97E-07 | Chr1 | 8023810 | 8024819 | 924 |
| novel.178 | -4.86685 | 1.06E-08 | 7.01E-07 | Chr1 | 25369928 | 25373655 | 502 |
| novel.8736 | 7.917941 | 1.09E-08 | 7.23E-07 | Chr8 | 83538676 | 83542462 | 1237 |
| MsG0680033567.01 | 5.280157 | 1.11E-08 | 7.31E-07 | Chr6 | 68434691 | 68440587 | 2582 |
| MsG0480018894.01 | 3.810156 | 1.18E-08 | 7.80E-07 | Chr4 | 11246140 | 11249947 | 298 |
| novel.2001 | 2.725459 | 1.19E-08 | 7.81E-07 | Chr2 | 69424700 | 69428286 | 2965 |
| novel.8542 | -8.27262 | 1.20E-08 | 7.88E-07 | Chr8 | 50854351 | 50857709 | 728 |
| MsG0180005988.01 | 2.855742 | 1.22E-08 | 7.96E-07 | Chr1 | 98542597 | 98548035 | 995 |
| novel.5848 | -7.87478 | 1.22E-08 | 8.01E-07 | Chr6 | 69790045 | 69795260 | 1879 |
| MsG0180005841.01 | -2.78817 | 1.24E-08 | 8.10E-07 | Chr1 | 96780764 | 96786651 | 1741 |
| MsG0880045422.01 | -7.9698 | 1.26E-08 | 8.21E-07 | Chr8 | 58976001 | 58976411 | 411 |
| novel.7013 | 7.006375 | 1.32E-08 | 8.58E-07 | Chr7 | 49342872 | 49345699 | 1712 |
| MsG0380017450.01 | -4.9302 | 1.39E-08 | 9.00E-07 | Chr3 | 96381869 | 96386711 | 2097 |
| novel.7825 | 7.936835 | 1.41E-08 | 9.10E-07 | Chr8 | 6017701 | 6019770 | 671 |
| MsG0880042836.01 | -3.09081 | 1.42E-08 | 9.18E-07 | Chr8 | 14695680 | 14711688 | 5405 |
| MsG0880045928.01 | -2.31247 | 1.44E-08 | 9.24E-07 | Chr8 | 66601495 | 66605843 | 2229 |
| novel.219 | -2.62666 | 1.44E-08 | 9.24E-07 | Chr1 | 34098264 | 34101945 | 2434 |
| MsG0280007394.01 | -5.46625 | 1.47E-08 | 9.44E-07 | Chr2 | 14881124 | 14883737 | 702 |
| novel.4844 | 5.104485 | 1.52E-08 | 9.75E-07 | Chr5 | 1.01E+08 | 1.01E+08 | 4003 |
| MsG0380012197.01 | 8.04623 | 1.54E-08 | 9.83E-07 | Chr3 | 12558969 | 12563498 | 4530 |
| MsG0180003697.01 | 2.914738 | 1.54E-08 | 9.83E-07 | Chr1 | 66822045 | 66826847 | 1230 |
| MsG0280007125.01 | 3.524166 | 1.57E-08 | 9.98E-07 | Chr2 | 10975004 | 10975729 | 726 |
| novel.3965 | 8.508231 | 1.60E-08 | 1.02E-06 | Chr4 | 47443240 | 47445287 | 1613 |
| novel.2545 | 2.879019 | 1.62E-08 | 1.03E-06 | Chr3 | 85024004 | 85028487 | 3411 |
| MsG0480022813.01 | -3.03068 | 1.66E-08 | 1.05E-06 | Chr4 | 76994451 | 76994966 | 516 |
| novel.1982 | -4.90144 | 1.79E-08 | 1.13E-06 | Chr2 | 66099722 | 66100974 | 778 |
| novel.4313 | 6.9666 | 1.82E-08 | 1.14E-06 | Chr5 | 4677223 | 4680079 | 1330 |
| MsG0180000733.01 | 8.128088 | 1.86E-08 | 1.17E-06 | Chr1 | 10396489 | 10399691 | 946 |
| MsG0580024551.01 | -5.68589 | 1.94E-08 | 1.22E-06 | Chr5 | 6283157 | 6285565 | 2409 |
| MsG0780038571.01 | -3.35192 | 1.98E-08 | 1.24E-06 | Chr7 | 48985038 | 48987928 | 1131 |
| MsG0180003539.01 | -3.01195 | 1.98E-08 | 1.24E-06 | Chr1 | 63919298 | 63923113 | 3174 |
| MsG0280006552.01 | -3.26247 | 1.99E-08 | 1.24E-06 | Chr2 | 3379485 | 3379976 | 492 |
| MsG0880043068.01 | -1.82685 | 2.03E-08 | 1.26E-06 | Chr8 | 18401263 | 18404011 | 402 |
| MsG0880043966.01 | 7.84006 | 2.14E-08 | 1.33E-06 | Chr8 | 33522251 | 33522433 | 183 |
| MsG0180004942.01 | -6.40478 | 2.16E-08 | 1.34E-06 | Chr1 | 84718735 | 84731584 | 399 |
| MsG0880042085.01 | -3.92821 | 2.21E-08 | 1.37E-06 | Chr8 | 3527216 | 3529693 | 2478 |
| MsG0580028701.01 | -4.42348 | 2.29E-08 | 1.41E-06 | Chr5 | 85037311 | 85039828 | 1026 |
| MsG0280009909.01 | -2.51665 | 2.30E-08 | 1.42E-06 | Chr2 | 60620434 | 60625723 | 2370 |
| novel.6678 | 7.727447 | 2.37E-08 | 1.45E-06 | Chr6 | 99848461 | 99850486 | 1621 |
| novel.9112 | -4.98149 | 2.39E-08 | 1.46E-06 | contig493end | 3529 | 5080 | 704 |
| novel.7136 | 4.866134 | 2.45E-08 | 1.49E-06 | Chr7 | 75265757 | 75268872 | 953 |
| novel.5052 | 7.757086 | 2.48E-08 | 1.51E-06 | Chr5 | 28273431 | 28274345 | 680 |
| novel.4284 | -8.24945 | 2.50E-08 | 1.52E-06 | Chr5 | 971662 | 972045 | 384 |
| novel.9334 | 2.202415 | 2.55E-08 | 1.55E-06 | contig628end | 3831 | 8852 | 1703 |
| novel.791 | 8.329019 | 2.57E-08 | 1.56E-06 | Chr1 | 30680816 | 30685461 | 1753 |
| novel.4997 | 7.748443 | 2.57E-08 | 1.56E-06 | Chr5 | 15414345 | 15426096 | 379 |
| novel.4140 | 7.717574 | 2.60E-08 | 1.57E-06 | Chr4 | 75398930 | 75404805 | 593 |
| MsG0780037930.01 | -8.05224 | 2.60E-08 | 1.57E-06 | Chr7 | 36529882 | 36534102 | 1062 |
| MsG0380012895.01 | -7.62198 | 2.64E-08 | 1.59E-06 | Chr3 | 26545903 | 26549770 | 3663 |
| novel.6241 | 3.884218 | 2.73E-08 | 1.64E-06 | Chr6 | 14516835 | 14517716 | 882 |
| MsG0180000901.01 | -8.14887 | 2.75E-08 | 1.64E-06 | Chr1 | 12995186 | 12996945 | 1296 |
| MsG0580029157.01 | 7.697931 | 2.77E-08 | 1.65E-06 | Chr5 | 93019632 | 93023771 | 545 |
| MsG0180005673.01 | -3.21147 | 2.87E-08 | 1.71E-06 | Chr1 | 94796143 | 94796997 | 855 |
| MsG0380013312.01 | 6.30194 | 2.88E-08 | 1.72E-06 | Chr3 | 34937766 | 34941593 | 2816 |
| MsG0780039572.01 | -8.55685 | 2.91E-08 | 1.72E-06 | Chr7 | 66035895 | 66038399 | 699 |
| MsG0480019804.01 | 5.480702 | 2.93E-08 | 1.74E-06 | Chr4 | 27579443 | 27581132 | 1242 |
| MsG0380012991.01 | 8.370002 | 2.96E-08 | 1.75E-06 | Chr3 | 28380970 | 28398030 | 1290 |
| MsG0580026480.01 | -7.88942 | 3.04E-08 | 1.80E-06 | Chr5 | 38257604 | 38260744 | 2013 |
| MsG0280010799.01 | 2.236111 | 3.07E-08 | 1.80E-06 | Chr2 | 75200455 | 75206813 | 543 |
| novel.3207 | 7.990273 | 3.07E-08 | 1.81E-06 | Chr3 | 1.02E+08 | 1.02E+08 | 1520 |
| novel.6281 | 5.842085 | 3.09E-08 | 1.81E-06 | Chr6 | 19855664 | 19859722 | 1898 |
| MsG0780035965.01 | 2.820981 | 3.11E-08 | 1.82E-06 | Chr7 | 713376 | 719489 | 1592 |
| novel.2780 | -4.37592 | 3.31E-08 | 1.93E-06 | Chr3 | 19137122 | 19147573 | 4727 |
| novel.6937 | 5.974808 | 3.39E-08 | 1.98E-06 | Chr7 | 30006324 | 30014184 | 1566 |
| novel.5335 | -5.71692 | 3.43E-08 | 1.99E-06 | Chr5 | 84938320 | 84942105 | 2109 |
| novel.1081 | -8.01962 | 3.43E-08 | 1.99E-06 | Chr1 | 92093623 | 92098798 | 512 |
| novel.7407 | -4.325 | 3.44E-08 | 1.99E-06 | Chr7 | 22074995 | 22077176 | 1006 |
| MsG0480019756.01 | -5.84676 | 3.46E-08 | 2.00E-06 | Chr4 | 26439816 | 26448188 | 2019 |
| novel.8265 | -4.45658 | 3.58E-08 | 2.07E-06 | Chr8 | 87338896 | 87342529 | 3244 |
| MsG0480022413.01 | -2.64624 | 3.60E-08 | 2.07E-06 | Chr4 | 72245059 | 72247479 | 1276 |
| MsG0480021066.01 | -7.82295 | 3.65E-08 | 2.10E-06 | Chr4 | 52954150 | 52954488 | 339 |
| novel.7390 | 8.205164 | 3.66E-08 | 2.10E-06 | Chr7 | 18319198 | 18320428 | 475 |
| MsG0480022311.01 | -4.48825 | 3.72E-08 | 2.13E-06 | Chr4 | 71001478 | 71003798 | 2038 |
| MsG0580026196.01 | 7.892736 | 3.74E-08 | 2.14E-06 | Chr5 | 32125317 | 32127566 | 1161 |
| MsG0680035643.01 | -8.21779 | 3.84E-08 | 2.19E-06 | Chr6 | 1.1E+08 | 1.1E+08 | 1410 |
| MsG0380012252.01 | -4.51836 | 3.84E-08 | 2.19E-06 | Chr3 | 13347771 | 13354367 | 2553 |
| MsG0180001580.01 | 8.074001 | 3.85E-08 | 2.19E-06 | Chr1 | 23640708 | 23642997 | 2154 |
| MsG0280010686.01 | 3.664944 | 3.87E-08 | 2.19E-06 | Chr2 | 73343776 | 73345574 | 900 |
| novel.3975 | 8.019397 | 3.90E-08 | 2.21E-06 | Chr4 | 49094436 | 49098718 | 1275 |
| MsG0480018780.01 | -4.44748 | 3.97E-08 | 2.24E-06 | Chr4 | 9675325 | 9678964 | 1464 |
| novel.8292 | -3.39606 | 4.07E-08 | 2.29E-06 | Chr8 | 90357378 | 90360563 | 1271 |
| MsG0580024955.01 | -2.03712 | 4.07E-08 | 2.29E-06 | Chr5 | 12094283 | 12099978 | 2196 |
| novel.8604 | -2.37799 | 4.17E-08 | 2.35E-06 | Chr8 | 60459324 | 60465739 | 2360 |
| novel.291 | 8.121901 | 4.21E-08 | 2.36E-06 | Chr1 | 55084721 | 55089507 | 1641 |
| novel.8872 | 7.650991 | 4.27E-08 | 2.39E-06 | contig238end | 22254 | 24077 | 1588 |
| MsG0880043941.01 | 4.029461 | 4.29E-08 | 2.40E-06 | Chr8 | 33086734 | 33089735 | 681 |
| novel.8631 | -7.384 | 4.32E-08 | 2.41E-06 | Chr8 | 65763754 | 65766088 | 902 |
| MsG0480019105.01 | -3.81182 | 4.33E-08 | 2.41E-06 | Chr4 | 14661383 | 14663058 | 1343 |
| MsG0280011135.01 | -4.65921 | 4.36E-08 | 2.42E-06 | Chr2 | 80029153 | 80030813 | 855 |
| MsG0580025814.01 | -2.86807 | 4.39E-08 | 2.44E-06 | Chr5 | 24592553 | 24595526 | 1736 |
| MsG0280009954.01 | -5.51101 | 4.47E-08 | 2.47E-06 | Chr2 | 61721118 | 61729621 | 3117 |
| novel.2612 | 7.623152 | 4.75E-08 | 2.62E-06 | Chr3 | 95103459 | 95106226 | 1999 |
| novel.7151 | 8.558396 | 4.81E-08 | 2.65E-06 | Chr7 | 76518633 | 76522767 | 1186 |
| novel.8138 | -4.14021 | 4.82E-08 | 2.66E-06 | Chr8 | 64822069 | 64825675 | 3102 |
| novel.785 | -7.8369 | 5.04E-08 | 2.77E-06 | Chr1 | 27688044 | 27689957 | 1914 |
| MsG0380016128.01 | -5.73795 | 5.07E-08 | 2.78E-06 | Chr3 | 79411449 | 79412172 | 589 |
| novel.5298 | -5.44625 | 5.12E-08 | 2.80E-06 | Chr5 | 77233073 | 77234764 | 1022 |
| MsG0480022310.01 | -4.38306 | 5.22E-08 | 2.85E-06 | Chr4 | 70997123 | 70998819 | 1385 |
| MsG0880044218.01 | -7.61953 | 5.32E-08 | 2.90E-06 | Chr8 | 37825099 | 37826079 | 981 |
| MsG0180001590.01 | -3.55149 | 5.34E-08 | 2.91E-06 | Chr1 | 23779008 | 23795471 | 6204 |
| MsG0880047655.01 | -2.68766 | 5.54E-08 | 3.01E-06 | Chr8 | 89269277 | 89274559 | 1936 |
| MsG0580025992.01 | 1.906935 | 5.61E-08 | 3.04E-06 | Chr5 | 28759291 | 28762815 | 875 |
| novel.5978 | -6.6454 | 5.68E-08 | 3.07E-06 | Chr6 | 93347619 | 93348870 | 1167 |
| MsG0380016704.01 | -2.53813 | 5.79E-08 | 3.13E-06 | Chr3 | 86799061 | 86803535 | 1026 |
| novel.1157 | 2.714185 | 5.90E-08 | 3.18E-06 | Chr1 | 1.02E+08 | 1.02E+08 | 1110 |
| MsG0780036216.01 | 8.703053 | 6.04E-08 | 3.25E-06 | Chr7 | 4781663 | 4783850 | 1371 |
| MsG0480022884.01 | -3.40556 | 6.11E-08 | 3.29E-06 | Chr4 | 78056071 | 78058661 | 1098 |
| MsG0180001676.01 | -3.42646 | 6.26E-08 | 3.36E-06 | Chr1 | 25012612 | 25016460 | 3437 |
| MsG0380013594.01 | 7.234644 | 6.27E-08 | 3.36E-06 | Chr3 | 41374246 | 41375087 | 750 |
| novel.5257 | 4.512455 | 6.34E-08 | 3.39E-06 | Chr5 | 70711836 | 70724345 | 2004 |
| novel.6055 | 7.278308 | 6.66E-08 | 3.56E-06 | Chr6 | 1.04E+08 | 1.04E+08 | 967 |
| novel.7199 | 8.149674 | 6.68E-08 | 3.56E-06 | Chr7 | 82906912 | 82908517 | 1501 |
| novel.2240 | -8.01784 | 6.80E-08 | 3.62E-06 | Chr3 | 18908505 | 18909895 | 1391 |
| MsG0380014405.01 | -7.90474 | 6.91E-08 | 3.67E-06 | Chr3 | 54230316 | 54233102 | 2787 |
| MsG0780037779.01 | 3.271064 | 6.99E-08 | 3.71E-06 | Chr7 | 33082562 | 33086144 | 876 |
| MsG0380011651.01 | -1.98206 | 7.10E-08 | 3.75E-06 | Chr3 | 3029374 | 3035369 | 1818 |
| MsG0880045441.01 | -3.53992 | 7.10E-08 | 3.75E-06 | Chr8 | 59182388 | 59184400 | 1188 |
| novel.7030 | 8.1352 | 7.17E-08 | 3.78E-06 | Chr7 | 53082107 | 53083710 | 1604 |
| MsG0180005150.01 | -5.15114 | 7.18E-08 | 3.78E-06 | Chr1 | 87635855 | 87636400 | 450 |
| MsG0380013041.01 | 6.384809 | 7.43E-08 | 3.90E-06 | Chr3 | 29197832 | 29204027 | 1521 |
| novel.9280 | 4.97756 | 7.47E-08 | 3.91E-06 | contig591end | 18136 | 20769 | 1658 |
| novel.8242 | -7.63086 | 7.55E-08 | 3.95E-06 | Chr8 | 84570504 | 84573637 | 2108 |
| MsG0880046908.01 | -2.72614 | 7.59E-08 | 3.96E-06 | Chr8 | 79773489 | 79779765 | 1689 |
| MsG0580029482.01 | 7.851325 | 7.73E-08 | 4.03E-06 | Chr5 | 97585636 | 97586795 | 300 |
| MsG0380015047.01 | 7.701492 | 7.76E-08 | 4.04E-06 | Chr3 | 64496707 | 64497252 | 546 |
| novel.3672 | 2.027177 | 7.97E-08 | 4.14E-06 | Chr4 | 83811670 | 83839394 | 7204 |
| MsG0880045690.01 | 2.318348 | 8.21E-08 | 4.26E-06 | Chr8 | 62797392 | 62799663 | 1089 |
| novel.3498 | 7.564275 | 8.38E-08 | 4.34E-06 | Chr4 | 56238620 | 56241655 | 551 |
| novel.2032 | 2.340556 | 8.39E-08 | 4.34E-06 | Chr2 | 74387639 | 74389255 | 1113 |
| MsG0380016748.01 | -3.38398 | 8.40E-08 | 4.34E-06 | Chr3 | 87362275 | 87364194 | 1430 |
| novel.1244 | -8.38704 | 8.46E-08 | 4.35E-06 | Chr2 | 13643972 | 13644553 | 475 |
| novel.1691 | -2.87716 | 8.46E-08 | 4.35E-06 | Chr2 | 6395593 | 6398080 | 2297 |
| MsG0780039296.01 | 7.511536 | 8.50E-08 | 4.36E-06 | Chr7 | 61498757 | 61499041 | 285 |
| MsG0480023796.01 | 3.807397 | 8.51E-08 | 4.36E-06 | Chr4 | 89917300 | 89917945 | 531 |
| novel.5422 | -7.75987 | 8.53E-08 | 4.36E-06 | Chr5 | 98972622 | 98974234 | 802 |
| MsG0780040412.01 | 4.925832 | 8.55E-08 | 4.37E-06 | Chr7 | 77144591 | 77148026 | 1350 |
| novel.4741 | 7.218478 | 8.64E-08 | 4.41E-06 | Chr5 | 85175121 | 85176421 | 1091 |
| novel.191 | 7.959779 | 8.69E-08 | 4.42E-06 | Chr1 | 26166993 | 26168724 | 659 |
| MsG0380017076.01 | -3.12724 | 8.77E-08 | 4.46E-06 | Chr3 | 91668434 | 91669342 | 909 |
| novel.1862 | -5.51423 | 8.79E-08 | 4.46E-06 | Chr2 | 36199167 | 36200506 | 1002 |
| MsG0180005683.01 | -2.77201 | 8.96E-08 | 4.54E-06 | Chr1 | 94873763 | 94886275 | 4836 |
| MsG0580024089.01 | 3.397089 | 9.05E-08 | 4.57E-06 | Chr5 | 682234 | 689037 | 2103 |
| novel.7911 | -7.542 | 9.06E-08 | 4.57E-06 | Chr8 | 20816403 | 20817681 | 769 |
| novel.6019 | 7.899036 | 9.09E-08 | 4.58E-06 | Chr6 | 1E+08 | 1E+08 | 567 |
| MsG0680032241.01 | 7.155046 | 9.52E-08 | 4.79E-06 | Chr6 | 37186090 | 37186611 | 339 |
| novel.1765 | 4.439907 | 9.77E-08 | 4.90E-06 | Chr2 | 17079889 | 17080969 | 654 |
| novel.4828 | -6.68587 | 9.77E-08 | 4.90E-06 | Chr5 | 99341678 | 99346718 | 1664 |
| MsG0880041900.01 | 3.05085 | 9.82E-08 | 4.91E-06 | Chr8 | 903977 | 905914 | 1482 |
| MsG0880043205.01 | -7.25289 | 1.00E-07 | 5.00E-06 | Chr8 | 20583565 | 20588292 | 1545 |
| MsG0180004678.01 | -7.60834 | 1.02E-07 | 5.07E-06 | Chr1 | 81133253 | 81134866 | 633 |
| novel.1794 | -7.51577 | 1.02E-07 | 5.07E-06 | Chr2 | 22954928 | 22957850 | 1557 |
| MsG0680034190.01 | 3.124292 | 1.03E-07 | 5.10E-06 | Chr6 | 83015018 | 83020738 | 713 |
| MsG0180002100.01 | 8.040846 | 1.04E-07 | 5.17E-06 | Chr1 | 32892562 | 32895039 | 730 |
| novel.4613 | 6.352484 | 1.04E-07 | 5.17E-06 | Chr5 | 59882420 | 59885107 | 1266 |
| novel.5007 | -7.82586 | 1.05E-07 | 5.20E-06 | Chr5 | 17152940 | 17154397 | 346 |
| MsG0180005156.01 | 3.668191 | 1.06E-07 | 5.23E-06 | Chr1 | 87769974 | 87775928 | 2442 |
| novel.1307 | -8.13748 | 1.06E-07 | 5.24E-06 | Chr2 | 23762460 | 23763543 | 1084 |
| MsG0180003710.01 | 3.67055 | 1.07E-07 | 5.26E-06 | Chr1 | 67185846 | 67191956 | 1245 |
| novel.9087 | 8.224007 | 1.07E-07 | 5.26E-06 | contig474end | 24456 | 26194 | 531 |
| MsG0580026008.01 | -4.92615 | 1.07E-07 | 5.26E-06 | Chr5 | 29012889 | 29013563 | 675 |
| novel.7283 | 8.2237 | 1.07E-07 | 5.26E-06 | Chr7 | 94146759 | 94148748 | 1017 |
| novel.2318 | 7.746307 | 1.09E-07 | 5.34E-06 | Chr3 | 36885937 | 36888216 | 663 |
| novel.1634 | 2.619828 | 1.13E-07 | 5.49E-06 | Chr2 | 83481977 | 83484969 | 2993 |
| MsG0880043896.01 | 8.519134 | 1.13E-07 | 5.49E-06 | Chr8 | 32316005 | 32316970 | 966 |
| novel.8577 | 6.411807 | 1.14E-07 | 5.52E-06 | Chr8 | 54659079 | 54661395 | 1911 |
| MsG0680032305.01 | -3.31372 | 1.14E-07 | 5.52E-06 | Chr6 | 38132468 | 38135905 | 3438 |
| MsG0580024216.01 | 6.604475 | 1.16E-07 | 5.63E-06 | Chr5 | 2133817 | 2137882 | 1110 |
| MsG0780040942.01 | -3.57084 | 1.19E-07 | 5.75E-06 | Chr7 | 84578247 | 84581524 | 2619 |
| MsG0380014362.01 | 2.623854 | 1.19E-07 | 5.76E-06 | Chr3 | 53505681 | 53506772 | 504 |
| novel.6447 | -7.63809 | 1.20E-07 | 5.78E-06 | Chr6 | 49316991 | 49320385 | 1466 |
| MsG0680033022.01 | -3.22384 | 1.23E-07 | 5.93E-06 | Chr6 | 55004976 | 55005305 | 330 |
| novel.1423 | 8.271705 | 1.26E-07 | 6.08E-06 | Chr2 | 47854137 | 47858593 | 4140 |
| MsG0880047320.01 | -5.19584 | 1.28E-07 | 6.13E-06 | Chr8 | 84610445 | 84622271 | 2475 |
| MsG0080047962.01 | 3.62009 | 1.30E-07 | 6.21E-06 | contig152end | 388942 | 403227 | 3417 |
| MsG0480019106.01 | -3.48625 | 1.31E-07 | 6.27E-06 | Chr4 | 14664275 | 14670051 | 4257 |
| MsG0880042996.01 | -7.52465 | 1.33E-07 | 6.36E-06 | Chr8 | 17327157 | 17328333 | 810 |
| MsG0280008026.01 | 7.592337 | 1.34E-07 | 6.40E-06 | Chr2 | 24389580 | 24390764 | 1185 |
| MsG0680031444.01 | 3.362108 | 1.37E-07 | 6.53E-06 | Chr6 | 20495918 | 20496724 | 807 |
| MsG0380015369.01 | -3.30221 | 1.41E-07 | 6.68E-06 | Chr3 | 69139413 | 69143347 | 2111 |
| MsG0680031058.01 | 7.646299 | 1.41E-07 | 6.68E-06 | Chr6 | 13420861 | 13422885 | 1371 |
| novel.126 | -4.68257 | 1.42E-07 | 6.74E-06 | Chr1 | 16421765 | 16426625 | 2720 |
| MsG0780041029.01 | 4.216371 | 1.45E-07 | 6.85E-06 | Chr7 | 85668183 | 85669617 | 453 |
| MsG0580025808.01 | -2.76281 | 1.50E-07 | 7.11E-06 | Chr5 | 24521163 | 24526043 | 873 |
| novel.5625 | -3.57801 | 1.55E-07 | 7.30E-06 | Chr6 | 18364865 | 18368476 | 3455 |
| MsG0180003207.01 | -7.65024 | 1.55E-07 | 7.30E-06 | Chr1 | 58350958 | 58357619 | 1611 |
| MsG0780036180.01 | 7.507687 | 1.55E-07 | 7.30E-06 | Chr7 | 4351355 | 4353650 | 1152 |
| novel.2176 | -4.13825 | 1.55E-07 | 7.30E-06 | Chr3 | 9049669 | 9054781 | 4571 |
| MsG0580027937.01 | -3.66327 | 1.57E-07 | 7.37E-06 | Chr5 | 71495638 | 71500186 | 516 |
| novel.8167 | 7.595127 | 1.58E-07 | 7.38E-06 | Chr8 | 71135463 | 71140038 | 1692 |
| MsG0180003755.01 | -7.15963 | 1.65E-07 | 7.73E-06 | Chr1 | 67708991 | 67713280 | 639 |
| MsG0680031116.01 | -5.47811 | 1.66E-07 | 7.77E-06 | Chr6 | 14716177 | 14720444 | 1376 |
| MsG0580029421.01 | 3.140802 | 1.68E-07 | 7.84E-06 | Chr5 | 96785924 | 96789561 | 813 |
| novel.6255 | 7.825625 | 1.71E-07 | 7.96E-06 | Chr6 | 16860262 | 16861639 | 1208 |
| MsG0580025985.01 | 2.307035 | 1.76E-07 | 8.18E-06 | Chr5 | 28693155 | 28695783 | 1680 |
| MsG0280008172.01 | -3.33576 | 1.78E-07 | 8.24E-06 | Chr2 | 26731174 | 26735482 | 1446 |
| MsG0580030181.01 | -3.60444 | 1.78E-07 | 8.26E-06 | Chr5 | 1.08E+08 | 1.08E+08 | 1611 |
| novel.4544 | 5.154618 | 1.81E-07 | 8.35E-06 | Chr5 | 46124164 | 46127406 | 3243 |
| novel.4661 | -3.51753 | 1.81E-07 | 8.36E-06 | Chr5 | 71752993 | 71753924 | 400 |
| MsG0480022153.01 | 7.832455 | 1.82E-07 | 8.41E-06 | Chr4 | 69003312 | 69005774 | 1230 |
| novel.1185 | -5.1602 | 1.85E-07 | 8.52E-06 | Chr2 | 3217675 | 3222668 | 950 |
| MsG0880046819.01 | -3.61897 | 1.91E-07 | 8.77E-06 | Chr8 | 78711789 | 78712160 | 372 |
| novel.8993 | -3.77839 | 1.92E-07 | 8.81E-06 | contig408end | 238225 | 241328 | 1845 |
| MsG0480021666.01 | 2.989032 | 1.96E-07 | 8.97E-06 | Chr4 | 62613168 | 62614581 | 351 |
| MsG0380017161.01 | -1.82621 | 1.99E-07 | 9.12E-06 | Chr3 | 92812593 | 92816817 | 1944 |
| MsG0680031458.01 | 3.145314 | 2.01E-07 | 9.20E-06 | Chr6 | 20745993 | 20750098 | 1064 |
| MsG0780039473.01 | -4.21316 | 2.07E-07 | 9.42E-06 | Chr7 | 64600139 | 64600642 | 504 |
| MsG0780037917.01 | -2.86462 | 2.10E-07 | 9.56E-06 | Chr7 | 36350617 | 36355097 | 1008 |
| MsG0280006983.01 | -2.68805 | 2.10E-07 | 9.56E-06 | Chr2 | 8976785 | 8985962 | 3799 |
| MsG0580026557.01 | 7.479758 | 2.15E-07 | 9.75E-06 | Chr5 | 39460608 | 39462083 | 930 |
| novel.212 | -7.40289 | 2.15E-07 | 9.75E-06 | Chr1 | 30696869 | 30697994 | 574 |
| novel.8199 | 6.406178 | 2.15E-07 | 9.75E-06 | Chr8 | 76828785 | 76831268 | 974 |
| MsG0380011717.01 | 2.199203 | 2.16E-07 | 9.76E-06 | Chr3 | 3995383 | 4008287 | 1293 |
| MsG0580029296.01 | -3.7417 | 2.16E-07 | 9.76E-06 | Chr5 | 94847462 | 94857200 | 2475 |
| MsG0480023819.01 | -1.98979 | 2.17E-07 | 9.78E-06 | Chr4 | 90179007 | 90182659 | 1801 |
| novel.1869 | -8.04909 | 2.19E-07 | 9.85E-06 | Chr2 | 37281442 | 37282121 | 680 |
| MsG0780036791.01 | -5.30266 | 2.19E-07 | 9.85E-06 | Chr7 | 13878443 | 13884334 | 3456 |
| novel.4791 | -7.61014 | 2.21E-07 | 9.87E-06 | Chr5 | 93351373 | 93352104 | 515 |
| novel.586 | -7.61119 | 2.21E-07 | 9.87E-06 | Chr1 | 774784 | 777370 | 1414 |
| novel.8687 | -6.06675 | 2.30E-07 | 1.03E-05 | Chr8 | 74390599 | 74392863 | 1333 |
| novel.728 | -7.39554 | 2.31E-07 | 1.03E-05 | Chr1 | 17771954 | 17772955 | 743 |
| MsG0180000660.01 | 7.929515 | 2.32E-07 | 1.03E-05 | Chr1 | 9376930 | 9377639 | 417 |
| MsG0280011392.01 | -8.1031 | 2.33E-07 | 1.03E-05 | Chr2 | 83400280 | 83401929 | 1206 |
| MsG0780038359.01 | -2.2202 | 2.59E-07 | 1.15E-05 | Chr7 | 44997212 | 45002951 | 1236 |
| novel.4100 | -3.14196 | 2.61E-07 | 1.16E-05 | Chr4 | 69079682 | 69084241 | 2135 |
| novel.1139 | 7.834881 | 2.64E-07 | 1.17E-05 | Chr1 | 1E+08 | 1E+08 | 2437 |
| novel.4583 | 4.195391 | 2.68E-07 | 1.18E-05 | Chr5 | 54686780 | 54689581 | 837 |
| MsG0280010384.01 | 7.604525 | 2.70E-07 | 1.19E-05 | Chr2 | 68841077 | 68841235 | 159 |
| MsG0680031364.01 | 5.642723 | 2.71E-07 | 1.19E-05 | Chr6 | 19012956 | 19015649 | 799 |
| novel.5641 | 7.018286 | 2.72E-07 | 1.20E-05 | Chr6 | 20935524 | 20947753 | 2238 |
| MsG0780041216.01 | 3.248263 | 2.72E-07 | 1.20E-05 | Chr7 | 88346312 | 88346536 | 225 |
| MsG0380012396.01 | -5.29244 | 2.73E-07 | 1.20E-05 | Chr3 | 15952851 | 15960215 | 780 |
| MsG0880046399.01 | -2.32904 | 2.73E-07 | 1.20E-05 | Chr8 | 72872452 | 72882325 | 2733 |
| MsG0280009297.01 | 7.587661 | 2.74E-07 | 1.20E-05 | Chr2 | 49681427 | 49683620 | 891 |
| novel.6467 | -4.28044 | 2.75E-07 | 1.20E-05 | Chr6 | 53649357 | 53656306 | 3095 |
| MsG0480018369.01 | 4.491099 | 2.75E-07 | 1.20E-05 | Chr4 | 3927792 | 3933931 | 1511 |
| novel.8634 | -7.38281 | 2.76E-07 | 1.20E-05 | Chr8 | 66205547 | 66209870 | 946 |
| MsG0780040954.01 | 7.421633 | 2.82E-07 | 1.23E-05 | Chr7 | 84738212 | 84743529 | 2015 |
| MsG0780038990.01 | 7.336752 | 2.87E-07 | 1.25E-05 | Chr7 | 56489525 | 56489713 | 189 |
| MsG0780037943.01 | -3.42567 | 2.92E-07 | 1.27E-05 | Chr7 | 36708108 | 36710631 | 1071 |
| novel.8670 | -4.67488 | 2.96E-07 | 1.28E-05 | Chr8 | 72185409 | 72186503 | 809 |
| novel.4231 | -4.34963 | 2.96E-07 | 1.28E-05 | Chr4 | 87659845 | 87661984 | 2140 |
| MsG0780038471.01 | 3.748702 | 2.96E-07 | 1.28E-05 | Chr7 | 47271322 | 47275569 | 1929 |
| MsG0180004308.01 | 7.414811 | 2.97E-07 | 1.28E-05 | Chr1 | 76327638 | 76330956 | 1098 |
| novel.7848 | -4.61295 | 3.04E-07 | 1.31E-05 | Chr8 | 10236106 | 10236576 | 471 |
| novel.5419 | -7.3857 | 3.07E-07 | 1.32E-05 | Chr5 | 97988490 | 97989581 | 732 |
| MsG0580024986.01 | -4.72628 | 3.07E-07 | 1.32E-05 | Chr5 | 12554639 | 12557522 | 1198 |
| MsG0880045054.01 | -2.31396 | 3.11E-07 | 1.33E-05 | Chr8 | 53486579 | 53492738 | 2369 |
| novel.4443 | -4.89179 | 3.12E-07 | 1.33E-05 | Chr5 | 25272540 | 25273716 | 451 |
| novel.6065 | -2.49835 | 3.20E-07 | 1.37E-05 | Chr6 | 1.05E+08 | 1.05E+08 | 2111 |
| novel.8942 | 7.127347 | 3.24E-07 | 1.38E-05 | contig336end | 17612 | 29045 | 1983 |
| novel.580 | -3.97516 | 3.28E-07 | 1.40E-05 | Chr1 | 569502 | 574904 | 532 |
| MsG0880047755.01 | -4.44738 | 3.29E-07 | 1.40E-05 | Chr8 | 90436431 | 90440543 | 1230 |
| novel.6260 | 6.302495 | 3.30E-07 | 1.40E-05 | Chr6 | 17072888 | 17078002 | 2852 |
| MsG0380016086.01 | -5.03304 | 3.41E-07 | 1.45E-05 | Chr3 | 78977026 | 78978734 | 1353 |
| MsG0480019238.01 | -7.62202 | 3.51E-07 | 1.49E-05 | Chr4 | 16717098 | 16721663 | 1766 |
| MsG0180001998.01 | 3.44741 | 3.54E-07 | 1.50E-05 | Chr1 | 30730734 | 30735744 | 1182 |
| MsG0380017905.01 | 7.789391 | 3.57E-07 | 1.51E-05 | Chr3 | 1.02E+08 | 1.02E+08 | 1071 |
| novel.198 | -4.14741 | 3.65E-07 | 1.54E-05 | Chr1 | 28113836 | 28117268 | 1140 |
| novel.9108 | -4.59976 | 3.68E-07 | 1.55E-05 | contig488end | 1 | 1730 | 1730 |
| novel.1161 | -4.00652 | 3.81E-07 | 1.60E-05 | Chr2 | 455527 | 457718 | 725 |
| MsG0380015484.01 | -2.54505 | 3.84E-07 | 1.62E-05 | Chr3 | 70858911 | 70862344 | 1325 |
| novel.67 | 3.830137 | 3.92E-07 | 1.65E-05 | Chr1 | 8551927 | 8561007 | 1627 |
| MsG0780038836.01 | -2.55354 | 3.95E-07 | 1.66E-05 | Chr7 | 53775729 | 53777768 | 2040 |
| novel.5283 | -7.53979 | 3.97E-07 | 1.66E-05 | Chr5 | 74773865 | 74795660 | 1322 |
| novel.6168 | 7.562251 | 4.05E-07 | 1.69E-05 | Chr6 | 5643627 | 5644844 | 998 |
| novel.6848 | -8.00924 | 4.06E-07 | 1.70E-05 | Chr7 | 14412205 | 14416811 | 469 |
| MsG0380011579.01 | 4.996819 | 4.31E-07 | 1.80E-05 | Chr3 | 1733885 | 1747724 | 2543 |
| novel.477 | 3.212393 | 4.33E-07 | 1.80E-05 | Chr1 | 85638566 | 85642736 | 3230 |
| novel.7050 | -7.24822 | 4.35E-07 | 1.81E-05 | Chr7 | 59076563 | 59078791 | 1450 |
| MsG0080047802.01 | -3.88292 | 4.36E-07 | 1.81E-05 | contig108end | 9812 | 14952 | 1498 |
| MsG0480021839.01 | 2.647633 | 4.39E-07 | 1.82E-05 | Chr4 | 65005169 | 65008408 | 1779 |
| novel.2358 | 2.056378 | 4.39E-07 | 1.82E-05 | Chr3 | 47730425 | 47735037 | 1058 |
| MsG0780040612.01 | -4.26988 | 4.48E-07 | 1.85E-05 | Chr7 | 79986618 | 79987088 | 471 |
| novel.4851 | -7.30339 | 4.50E-07 | 1.86E-05 | Chr5 | 1.02E+08 | 1.02E+08 | 525 |
| novel.3649 | 7.440823 | 4.53E-07 | 1.87E-05 | Chr4 | 80281137 | 80284666 | 1446 |
| MsG0580029180.01 | -4.86275 | 4.60E-07 | 1.89E-05 | Chr5 | 93335012 | 93339444 | 1614 |
| MsG0780041153.01 | 5.424982 | 4.60E-07 | 1.89E-05 | Chr7 | 87223787 | 87226817 | 873 |
| MsG0880042780.01 | 2.24337 | 4.63E-07 | 1.90E-05 | Chr8 | 13947829 | 13950360 | 1132 |
| novel.6738 | -5.38634 | 4.63E-07 | 1.90E-05 | Chr6 | 1.07E+08 | 1.07E+08 | 3106 |
| MsG0780036850.01 | 3.831706 | 4.69E-07 | 1.92E-05 | Chr7 | 15032731 | 15033180 | 450 |
| MsG0180005263.01 | -4.65407 | 4.69E-07 | 1.92E-05 | Chr1 | 89309048 | 89309344 | 297 |
| MsG0380013599.01 | 6.87944 | 4.70E-07 | 1.92E-05 | Chr3 | 41455551 | 41456126 | 576 |
| MsG0480018389.01 | -8.35189 | 4.81E-07 | 1.96E-05 | Chr4 | 4236404 | 4236658 | 255 |
| novel.936 | -5.71891 | 4.83E-07 | 1.97E-05 | Chr1 | 65918240 | 65920753 | 1066 |
| novel.7425 | 8.300931 | 4.91E-07 | 2.00E-05 | Chr7 | 28276391 | 28278858 | 1298 |
| MsG0680031451.01 | -3.11557 | 4.93E-07 | 2.00E-05 | Chr6 | 20644990 | 20645895 | 906 |
| MsG0180003210.01 | -5.36833 | 4.93E-07 | 2.00E-05 | Chr1 | 58403357 | 58404755 | 1260 |
| novel.4463 | -7.31762 | 4.97E-07 | 2.01E-05 | Chr5 | 27218665 | 27220131 | 1077 |
| novel.6516 | 5.175294 | 5.04E-07 | 2.04E-05 | Chr6 | 65778501 | 65779004 | 504 |
| novel.5889 | 4.634507 | 5.05E-07 | 2.04E-05 | Chr6 | 76475677 | 76477162 | 676 |
| MsG0580024990.01 | -5.20698 | 5.14E-07 | 2.07E-05 | Chr5 | 12578743 | 12580545 | 348 |
| MsG0580026845.01 | -4.23311 | 5.21E-07 | 2.09E-05 | Chr5 | 46317402 | 46320611 | 2448 |
| MsG0280009815.01 | -3.62167 | 5.21E-07 | 2.09E-05 | Chr2 | 58609490 | 58618874 | 4314 |
| MsG0880045765.01 | -2.20578 | 5.21E-07 | 2.09E-05 | Chr8 | 64013091 | 64013363 | 273 |
| MsG0880044817.01 | 4.502479 | 5.23E-07 | 2.10E-05 | Chr8 | 49610322 | 49614766 | 1560 |
| novel.2148 | 3.816584 | 5.52E-07 | 2.21E-05 | Chr3 | 5295108 | 5302064 | 1811 |
| MsG0880042846.01 | 7.389047 | 5.63E-07 | 2.25E-05 | Chr8 | 14832554 | 14833375 | 822 |
| novel.7216 | 4.965049 | 5.73E-07 | 2.29E-05 | Chr7 | 84876048 | 84881984 | 1160 |
| MsG0680031010.01 | 4.966572 | 5.79E-07 | 2.31E-05 | Chr6 | 12822451 | 12825398 | 426 |
| MsG0180003204.01 | -5.66824 | 5.82E-07 | 2.32E-05 | Chr1 | 58325608 | 58331313 | 1815 |
| MsG0480021208.01 | -4.27396 | 5.93E-07 | 2.36E-05 | Chr4 | 55198004 | 55200352 | 2349 |
| novel.8223 | -2.78477 | 5.97E-07 | 2.37E-05 | Chr8 | 81550561 | 81556627 | 3503 |
| MsG0780041752.01 | 7.398226 | 6.09E-07 | 2.42E-05 | Chr7 | 94730080 | 94738117 | 1551 |
| MsG0580027875.01 | -2.81573 | 6.17E-07 | 2.44E-05 | Chr5 | 70148609 | 70149556 | 948 |
| MsG0480023842.01 | 3.093412 | 6.20E-07 | 2.45E-05 | Chr4 | 90393484 | 90393894 | 411 |
| MsG0780040214.01 | 2.412146 | 6.23E-07 | 2.46E-05 | Chr7 | 74475361 | 74480708 | 386 |
| MsG0580024919.01 | -7.25413 | 6.28E-07 | 2.48E-05 | Chr5 | 11663991 | 11664308 | 318 |
| novel.8018 | 7.301974 | 6.29E-07 | 2.48E-05 | Chr8 | 42992150 | 42997118 | 1890 |
| MsG0580028850.01 | 7.503499 | 6.35E-07 | 2.50E-05 | Chr5 | 87553342 | 87555956 | 666 |
| MsG0380011726.01 | -2.1185 | 6.48E-07 | 2.55E-05 | Chr3 | 4161789 | 4166086 | 1956 |
| MsG0880047269.01 | 5.314736 | 6.62E-07 | 2.60E-05 | Chr8 | 83990024 | 83990602 | 579 |
| MsG0180000658.01 | -4.94109 | 6.65E-07 | 2.61E-05 | Chr1 | 9349941 | 9352493 | 708 |
| MsG0780038991.01 | 3.348685 | 6.74E-07 | 2.64E-05 | Chr7 | 56496976 | 56499502 | 478 |
| MsG0280010695.01 | 5.371643 | 6.75E-07 | 2.64E-05 | Chr2 | 73481567 | 73485905 | 308 |
| novel.8696 | 7.210614 | 6.80E-07 | 2.65E-05 | Chr8 | 76531979 | 76534253 | 737 |
| novel.4296 | -7.73778 | 6.81E-07 | 2.66E-05 | Chr5 | 2678330 | 2684897 | 390 |
| novel.8095 | -7.32882 | 6.86E-07 | 2.67E-05 | Chr8 | 58746752 | 58747596 | 845 |
| novel.6784 | -3.51666 | 6.88E-07 | 2.68E-05 | Chr6 | 1.13E+08 | 1.13E+08 | 837 |
| MsG0180001098.01 | 7.582759 | 6.92E-07 | 2.69E-05 | Chr1 | 15975994 | 15976685 | 336 |
| novel.8457 | -7.42489 | 7.03E-07 | 2.73E-05 | Chr8 | 30554791 | 30557908 | 1253 |
| MsG0880042877.01 | -3.02589 | 7.03E-07 | 2.73E-05 | Chr8 | 15295872 | 15299039 | 900 |
| MsG0880042995.01 | 4.308614 | 7.08E-07 | 2.74E-05 | Chr8 | 17312511 | 17315851 | 2721 |
| novel.2567 | 2.905704 | 7.10E-07 | 2.74E-05 | Chr3 | 87147390 | 87153951 | 2716 |
| novel.3689 | 7.578992 | 7.14E-07 | 2.76E-05 | Chr4 | 85100200 | 85101276 | 984 |
| MsG0480019541.01 | 7.191552 | 7.15E-07 | 2.76E-05 | Chr4 | 21874801 | 21877225 | 1525 |
| MsG0180000070.01 | -7.99987 | 7.27E-07 | 2.80E-05 | Chr1 | 1043529 | 1044479 | 951 |
| MsG0380014624.01 | -3.21333 | 7.30E-07 | 2.81E-05 | Chr3 | 57443700 | 57448330 | 1706 |
| novel.2131 | 7.415331 | 7.37E-07 | 2.83E-05 | Chr3 | 1935652 | 1937703 | 669 |
| novel.6569 | 7.259773 | 7.42E-07 | 2.84E-05 | Chr6 | 75191234 | 75193783 | 1743 |
| novel.2083 | -2.19143 | 7.57E-07 | 2.90E-05 | Chr2 | 80770379 | 80773364 | 1754 |
| MsG0780038632.01 | 7.183549 | 7.62E-07 | 2.91E-05 | Chr7 | 50181998 | 50187272 | 2154 |
| MsG0380012788.01 | 2.287747 | 7.71E-07 | 2.94E-05 | Chr3 | 24513936 | 24523680 | 4335 |
| MsG0480020786.01 | -3.85188 | 7.76E-07 | 2.96E-05 | Chr4 | 48261276 | 48265750 | 750 |
| MsG0880047263.01 | 4.342329 | 7.76E-07 | 2.96E-05 | Chr8 | 83939272 | 83941831 | 1230 |
| MsG0880045544.01 | -3.4759 | 7.79E-07 | 2.96E-05 | Chr8 | 60819569 | 60820138 | 570 |
| novel.7532 | -4.02441 | 7.92E-07 | 3.01E-05 | Chr7 | 55888003 | 55893222 | 2391 |
| MsG0180004765.01 | -3.84127 | 7.94E-07 | 3.01E-05 | Chr1 | 82448317 | 82448634 | 318 |
| novel.5719 | 5.191025 | 8.20E-07 | 3.11E-05 | Chr6 | 37218565 | 37219253 | 689 |
| novel.6801 | -2.4354 | 8.25E-07 | 3.12E-05 | Chr7 | 835947 | 842965 | 4989 |
| MsG0880042436.01 | 2.547056 | 8.30E-07 | 3.14E-05 | Chr8 | 8678272 | 8678820 | 549 |
| MsG0280010312.01 | 7.992262 | 8.42E-07 | 3.18E-05 | Chr2 | 67796618 | 67810145 | 2391 |
| novel.6336 | 7.758138 | 8.50E-07 | 3.20E-05 | Chr6 | 27268632 | 27269220 | 478 |
| MsG0680033510.01 | -7.26588 | 8.51E-07 | 3.20E-05 | Chr6 | 66915140 | 66917593 | 1203 |
| MsG0380012438.01 | -2.61063 | 8.58E-07 | 3.23E-05 | Chr3 | 16553670 | 16557110 | 3441 |
| MsG0580029376.01 | -1.46242 | 8.61E-07 | 3.23E-05 | Chr5 | 96093592 | 96110466 | 5234 |
| novel.579 | 2.14309 | 8.72E-07 | 3.27E-05 | Chr1 | 451213 | 455797 | 1955 |
| MsG0180004424.01 | -5.12517 | 8.80E-07 | 3.30E-05 | Chr1 | 77738491 | 77739664 | 912 |
| novel.1525 | -3.75466 | 9.01E-07 | 3.37E-05 | Chr2 | 70223473 | 70227470 | 831 |
| novel.1426 | 7.386025 | 9.05E-07 | 3.38E-05 | Chr2 | 48470054 | 48472670 | 1537 |
| novel.1880 | 7.799874 | 9.36E-07 | 3.49E-05 | Chr2 | 38958171 | 38958765 | 595 |
| MsG0680035348.01 | 2.240846 | 9.38E-07 | 3.50E-05 | Chr6 | 1.03E+08 | 1.03E+08 | 678 |
| novel.4783 | 4.180601 | 9.45E-07 | 3.52E-05 | Chr5 | 91664158 | 91666882 | 2140 |
| novel.5019 | -3.02079 | 9.72E-07 | 3.61E-05 | Chr5 | 20814084 | 20816373 | 498 |
| MsG0780036658.01 | -7.25317 | 9.78E-07 | 3.63E-05 | Chr7 | 11473854 | 11477384 | 186 |
| MsG0780037655.01 | 3.483801 | 9.80E-07 | 3.63E-05 | Chr7 | 30366072 | 30372119 | 1196 |
| MsG0080048036.01 | -3.46675 | 9.81E-07 | 3.64E-05 | contig179end | 11718 | 16018 | 1851 |
| MsG0680033852.01 | 2.467419 | 9.92E-07 | 3.67E-05 | Chr6 | 74809923 | 74810525 | 603 |
| novel.8980 | -3.37616 | 1.01E-06 | 3.74E-05 | contig399end | 15145 | 18689 | 2171 |
| MsG0480021254.01 | -2.58491 | 1.02E-06 | 3.77E-05 | Chr4 | 55940798 | 55954485 | 1584 |
| novel.1191 | 7.475901 | 1.03E-06 | 3.79E-05 | Chr2 | 3733939 | 3736067 | 2129 |
| novel.6614 | -7.46834 | 1.03E-06 | 3.81E-05 | Chr6 | 87193468 | 87202471 | 682 |
| MsG0180002026.01 | 5.980027 | 1.04E-06 | 3.81E-05 | Chr1 | 31332696 | 31346663 | 5195 |
| novel.2000 | 3.921505 | 1.04E-06 | 3.82E-05 | Chr2 | 69413838 | 69416191 | 1652 |
| novel.6680 | 7.336071 | 1.04E-06 | 3.82E-05 | Chr6 | 99880997 | 99886543 | 3144 |
| novel.7448 | 2.642775 | 1.07E-06 | 3.92E-05 | Chr7 | 33981678 | 33987107 | 1951 |
| MsG0880047435.01 | 1.922118 | 1.08E-06 | 3.96E-05 | Chr8 | 86277541 | 86280531 | 2202 |
| MsG0180001400.01 | -7.24058 | 1.08E-06 | 3.96E-05 | Chr1 | 20576517 | 20577934 | 1026 |
| MsG0680030397.01 | 1.860809 | 1.10E-06 | 4.03E-05 | Chr6 | 1662771 | 1676272 | 571 |
| novel.161 | 3.930469 | 1.15E-06 | 4.19E-05 | Chr1 | 23162723 | 23177285 | 2201 |
| novel.1414 | 3.401739 | 1.16E-06 | 4.21E-05 | Chr2 | 45244330 | 45252774 | 774 |
| MsG0280007997.01 | 6.779518 | 1.22E-06 | 4.42E-05 | Chr2 | 24107094 | 24107879 | 786 |
| MsG0480019738.01 | -3.27281 | 1.23E-06 | 4.46E-05 | Chr4 | 26179775 | 26184468 | 2412 |
| novel.8689 | -7.54926 | 1.23E-06 | 4.47E-05 | Chr8 | 75373622 | 75374284 | 663 |
| novel.341 | -6.29682 | 1.24E-06 | 4.48E-05 | Chr1 | 62739945 | 62741941 | 1034 |
| novel.4711 | 4.170987 | 1.26E-06 | 4.55E-05 | Chr5 | 79648076 | 79649265 | 965 |
| novel.3058 | 7.303182 | 1.28E-06 | 4.60E-05 | Chr3 | 79752487 | 79753952 | 1249 |
| MsG0380016020.01 | -3.12521 | 1.28E-06 | 4.60E-05 | Chr3 | 77999495 | 78000049 | 555 |
| novel.7303 | -7.19653 | 1.28E-06 | 4.60E-05 | Chr7 | 697464 | 698559 | 665 |
| MsG0180001433.01 | -7.26211 | 1.30E-06 | 4.68E-05 | Chr1 | 21064911 | 21074219 | 5799 |
| novel.6606 | 3.91964 | 1.30E-06 | 4.68E-05 | Chr6 | 84375297 | 84379379 | 3054 |
| MsG0180003130.01 | -4.34569 | 1.32E-06 | 4.76E-05 | Chr1 | 57067297 | 57071194 | 1287 |
| novel.7506 | -4.75221 | 1.33E-06 | 4.76E-05 | Chr7 | 48787141 | 48796283 | 374 |
| novel.892 | 3.432539 | 1.33E-06 | 4.76E-05 | Chr1 | 56753550 | 56756670 | 2559 |
| MsG0380015663.01 | 7.085741 | 1.34E-06 | 4.81E-05 | Chr3 | 73280475 | 73280699 | 225 |
| novel.4652 | -7.20507 | 1.35E-06 | 4.83E-05 | Chr5 | 69301712 | 69303035 | 627 |
| MsG0180001072.01 | 2.209914 | 1.35E-06 | 4.83E-05 | Chr1 | 15613325 | 15628687 | 2011 |
| novel.5366 | 7.729018 | 1.37E-06 | 4.89E-05 | Chr5 | 89493998 | 89497386 | 1948 |
| novel.3640 | -8.06254 | 1.38E-06 | 4.92E-05 | Chr4 | 79251315 | 79253414 | 2100 |
| MsG0480018855.01 | 7.700315 | 1.39E-06 | 4.94E-05 | Chr4 | 10650748 | 10654525 | 1224 |
| MsG0080048430.01 | 7.080259 | 1.39E-06 | 4.94E-05 | contig315end | 17815 | 21992 | 3081 |
| MsG0480018904.01 | -3.30558 | 1.40E-06 | 4.98E-05 | Chr4 | 11405614 | 11409053 | 970 |
| MsG0680034649.01 | -4.82049 | 1.41E-06 | 5.01E-05 | Chr6 | 92123720 | 92130117 | 1122 |
| novel.8976 | -7.16311 | 1.42E-06 | 5.03E-05 | contig389end | 5055 | 5784 | 730 |
| novel.2306 | 6.830004 | 1.42E-06 | 5.03E-05 | Chr3 | 33448143 | 33451467 | 2485 |
| novel.903 | 4.222327 | 1.43E-06 | 5.04E-05 | Chr1 | 60268064 | 60269742 | 1127 |
| novel.7796 | 4.434082 | 1.44E-06 | 5.08E-05 | Chr8 | 1733604 | 1734562 | 959 |
| MsG0280008882.01 | 2.040765 | 1.45E-06 | 5.12E-05 | Chr2 | 40835901 | 40842239 | 1532 |
| novel.4733 | 6.842014 | 1.45E-06 | 5.12E-05 | Chr5 | 83364259 | 83368410 | 2631 |
| MsG0380013726.01 | -2.62215 | 1.46E-06 | 5.14E-05 | Chr3 | 44651333 | 44654991 | 541 |
| MsG0280007635.01 | -3.45051 | 1.48E-06 | 5.20E-05 | Chr2 | 18371077 | 18372275 | 780 |
| MsG0380016100.01 | 7.329228 | 1.48E-06 | 5.21E-05 | Chr3 | 79135258 | 79138314 | 1677 |
| MsG0180001573.01 | 2.124289 | 1.51E-06 | 5.28E-05 | Chr1 | 23492362 | 23496638 | 2378 |
| novel.5870 | 7.844131 | 1.51E-06 | 5.30E-05 | Chr6 | 74492835 | 74496037 | 2784 |
| MsG0480018949.01 | -3.86306 | 1.57E-06 | 5.49E-05 | Chr4 | 12205487 | 12223573 | 7350 |
| MsG0080048957.01 | 5.664841 | 1.57E-06 | 5.49E-05 | contig567end | 20599 | 21446 | 773 |
| MsG0880045598.01 | 7.650936 | 1.57E-06 | 5.49E-05 | Chr8 | 61565291 | 61577903 | 1956 |
| MsG0580028416.01 | -2.9711 | 1.59E-06 | 5.55E-05 | Chr5 | 80274336 | 80277061 | 1339 |
| novel.4769 | -2.87115 | 1.60E-06 | 5.57E-05 | Chr5 | 90190073 | 90192760 | 1729 |
| novel.5307 | -2.63346 | 1.63E-06 | 5.66E-05 | Chr5 | 79852731 | 79856758 | 2644 |
| novel.1032 | 10.3077 | 1.65E-06 | 5.73E-05 | Chr1 | 86759855 | 86763870 | 1857 |
| MsG0380012629.01 | -4.06636 | 1.66E-06 | 5.74E-05 | Chr3 | 21520589 | 21520831 | 243 |
| MsG0180005160.01 | 4.022077 | 1.67E-06 | 5.78E-05 | Chr1 | 87805476 | 87808730 | 1222 |
| MsG0780040413.01 | 3.962647 | 1.71E-06 | 5.93E-05 | Chr7 | 77152078 | 77157370 | 1203 |
| novel.3130 | 7.027786 | 1.74E-06 | 6.01E-05 | Chr3 | 91629532 | 91632772 | 634 |
| novel.7560 | 6.788197 | 1.76E-06 | 6.07E-05 | Chr7 | 63743428 | 63745038 | 695 |
| MsG0680030667.01 | 3.388359 | 1.78E-06 | 6.12E-05 | Chr6 | 6919678 | 6927661 | 1602 |
| MsG0880045387.01 | -1.98678 | 1.78E-06 | 6.12E-05 | Chr8 | 58647442 | 58649327 | 621 |
| MsG0180003345.01 | 7.797892 | 1.78E-06 | 6.14E-05 | Chr1 | 60906831 | 60908890 | 1494 |
| novel.3447 | 4.626298 | 1.79E-06 | 6.15E-05 | Chr4 | 46014971 | 46017998 | 1423 |
| MsG0780040062.01 | 1.611936 | 1.80E-06 | 6.16E-05 | Chr7 | 72573108 | 72582964 | 1986 |
| MsG0880047240.01 | -2.25343 | 1.80E-06 | 6.16E-05 | Chr8 | 83720979 | 83724914 | 1287 |
| MsG0480022298.01 | 3.872711 | 1.87E-06 | 6.39E-05 | Chr4 | 70826081 | 70829347 | 1766 |
| MsG0380016076.01 | 2.872124 | 1.88E-06 | 6.42E-05 | Chr3 | 78793646 | 78794508 | 687 |
| MsG0180005752.01 | -3.65914 | 1.93E-06 | 6.58E-05 | Chr1 | 95686440 | 95687180 | 741 |
| novel.4725 | -3.73525 | 1.95E-06 | 6.65E-05 | Chr5 | 82200064 | 82204818 | 1350 |
| novel.177 | -7.13549 | 1.95E-06 | 6.65E-05 | Chr1 | 25316720 | 25319383 | 759 |
| MsG0080048825.01 | -3.78214 | 1.96E-06 | 6.67E-05 | contig468end | 24058 | 24639 | 582 |
| MsG0680035460.01 | -3.12596 | 1.97E-06 | 6.68E-05 | Chr6 | 1.06E+08 | 1.06E+08 | 1476 |
| novel.8147 | -3.8477 | 1.97E-06 | 6.70E-05 | Chr8 | 66577556 | 66578330 | 775 |
| novel.2785 | -7.41984 | 1.99E-06 | 6.76E-05 | Chr3 | 19799961 | 19807641 | 932 |
| MsG0680033683.01 | -2.53035 | 2.01E-06 | 6.79E-05 | Chr6 | 71117502 | 71119410 | 552 |
| novel.1212 | 2.622059 | 2.01E-06 | 6.79E-05 | Chr2 | 7306495 | 7309200 | 967 |
| MsG0880042391.01 | 2.798098 | 2.02E-06 | 6.84E-05 | Chr8 | 7992604 | 7999778 | 2425 |
| novel.4731 | 7.044095 | 2.03E-06 | 6.84E-05 | Chr5 | 83240584 | 83243584 | 3001 |
| MsG0780040426.01 | -3.44371 | 2.08E-06 | 7.00E-05 | Chr7 | 77384313 | 77386229 | 660 |
| novel.5049 | -7.67924 | 2.09E-06 | 7.05E-05 | Chr5 | 28235855 | 28243698 | 666 |
| MsG0780037478.01 | -2.3272 | 2.10E-06 | 7.06E-05 | Chr7 | 26678091 | 26682305 | 889 |
| MsG0180003457.01 | 7.369914 | 2.10E-06 | 7.06E-05 | Chr1 | 62525843 | 62526190 | 231 |
| MsG0380014094.01 | -4.25313 | 2.12E-06 | 7.13E-05 | Chr3 | 49582829 | 49590798 | 2359 |
| MsG0780038324.01 | 3.646444 | 2.13E-06 | 7.14E-05 | Chr7 | 44258425 | 44266118 | 2811 |
| novel.1449 | 7.053915 | 2.15E-06 | 7.20E-05 | Chr2 | 52343233 | 52345405 | 1458 |
| MsG0180000936.01 | -3.1152 | 2.15E-06 | 7.20E-05 | Chr1 | 13419266 | 13427644 | 2022 |
| MsG0580026146.01 | -7.10671 | 2.16E-06 | 7.23E-05 | Chr5 | 31352538 | 31355564 | 1956 |
| novel.6121 | 7.331166 | 2.16E-06 | 7.23E-05 | Chr6 | 1.13E+08 | 1.13E+08 | 1395 |
| novel.4425 | 3.964811 | 2.20E-06 | 7.32E-05 | Chr5 | 22926411 | 22929536 | 1396 |
| novel.748 | -7.49321 | 2.24E-06 | 7.46E-05 | Chr1 | 22357881 | 22360290 | 1140 |
| novel.917 | 7.49003 | 2.25E-06 | 7.47E-05 | Chr1 | 62694521 | 62696395 | 934 |
| MsG0180003115.01 | 2.116465 | 2.26E-06 | 7.50E-05 | Chr1 | 56840543 | 56843869 | 910 |
| novel.8567 | 4.514403 | 2.26E-06 | 7.50E-05 | Chr8 | 54230781 | 54247351 | 687 |
| MsG0180004332.01 | 7.220752 | 2.26E-06 | 7.50E-05 | Chr1 | 76485995 | 76490408 | 750 |
| MsG0480018890.01 | -2.77627 | 2.28E-06 | 7.55E-05 | Chr4 | 11185824 | 11191237 | 1912 |
| novel.4757 | 7.337638 | 2.32E-06 | 7.66E-05 | Chr5 | 87431288 | 87433460 | 1227 |
| MsG0480018206.01 | 2.473755 | 2.35E-06 | 7.78E-05 | Chr4 | 1550788 | 1555846 | 1200 |
| MsG0480021102.01 | 3.311975 | 2.37E-06 | 7.83E-05 | Chr4 | 53481983 | 53487526 | 1110 |
| novel.195 | 7.010399 | 2.38E-06 | 7.84E-05 | Chr1 | 27693602 | 27694404 | 704 |
| MsG0580027789.01 | -2.49163 | 2.38E-06 | 7.85E-05 | Chr5 | 68487914 | 68490275 | 2172 |
| MsG0380015633.01 | 2.632671 | 2.39E-06 | 7.87E-05 | Chr3 | 72771574 | 72772728 | 717 |
| MsG0180004594.01 | 2.174842 | 2.40E-06 | 7.88E-05 | Chr1 | 80107616 | 80114843 | 3108 |
| MsG0280006590.01 | -2.83095 | 2.40E-06 | 7.88E-05 | Chr2 | 3783696 | 3783857 | 162 |
| MsG0280006819.01 | 4.908097 | 2.41E-06 | 7.90E-05 | Chr2 | 6666645 | 6667780 | 554 |
| MsG0180001656.01 | -1.90493 | 2.42E-06 | 7.92E-05 | Chr1 | 24800490 | 24803863 | 3084 |
| novel.4724 | -3.93068 | 2.47E-06 | 8.08E-05 | Chr5 | 82076482 | 82077410 | 929 |
| MsG0180003817.01 | 2.58081 | 2.47E-06 | 8.09E-05 | Chr1 | 68663797 | 68667671 | 1150 |
| MsG0880043254.01 | 5.464918 | 2.49E-06 | 8.13E-05 | Chr8 | 21314631 | 21316647 | 267 |
| novel.4870 | 7.358013 | 2.50E-06 | 8.15E-05 | Chr5 | 1.04E+08 | 1.04E+08 | 1747 |
| novel.7061 | -6.63969 | 2.53E-06 | 8.24E-05 | Chr7 | 61098395 | 61101178 | 1765 |
| MsG0380015957.01 | 2.780712 | 2.56E-06 | 8.32E-05 | Chr3 | 77206076 | 77229704 | 10197 |
| novel.119 | 3.752834 | 2.57E-06 | 8.36E-05 | Chr1 | 15681188 | 15683482 | 1106 |
| MsG0780039682.01 | 4.150766 | 2.60E-06 | 8.43E-05 | Chr7 | 67506313 | 67506501 | 189 |
| novel.878 | 7.32268 | 2.63E-06 | 8.53E-05 | Chr1 | 53813723 | 53814244 | 256 |
| MsG0580024784.01 | -1.81403 | 2.64E-06 | 8.55E-05 | Chr5 | 9669105 | 9670100 | 996 |
| MsG0680031466.01 | 4.495068 | 2.67E-06 | 8.65E-05 | Chr6 | 20860980 | 20862115 | 696 |
| novel.4407 | 4.947025 | 2.67E-06 | 8.65E-05 | Chr5 | 20686594 | 20689171 | 2123 |
| MsG0680030982.01 | 4.083737 | 2.69E-06 | 8.66E-05 | Chr6 | 12505932 | 12508979 | 948 |
| MsG0580027808.01 | -4.8935 | 2.69E-06 | 8.66E-05 | Chr5 | 68864454 | 68865233 | 780 |
| MsG0280007764.01 | -2.28095 | 2.73E-06 | 8.79E-05 | Chr2 | 20228850 | 20231118 | 1192 |
| MsG0880045416.01 | -3.33035 | 2.79E-06 | 8.96E-05 | Chr8 | 58913469 | 58919234 | 1711 |
| novel.8353 | 7.914735 | 2.79E-06 | 8.96E-05 | Chr8 | 9824137 | 9825351 | 924 |
| MsG0880043271.01 | 2.891448 | 2.79E-06 | 8.96E-05 | Chr8 | 21825589 | 21829593 | 861 |
| novel.4344 | -3.21088 | 2.81E-06 | 9.01E-05 | Chr5 | 9655577 | 9659624 | 1592 |
| novel.3686 | 2.307216 | 2.82E-06 | 9.04E-05 | Chr4 | 84822305 | 84824749 | 681 |
| novel.2750 | -3.35894 | 2.82E-06 | 9.04E-05 | Chr3 | 11729569 | 11737188 | 2530 |
| MsG0680033015.01 | -4.54084 | 2.84E-06 | 9.07E-05 | Chr6 | 54795628 | 54801684 | 1389 |
| novel.1631 | 2.410494 | 2.86E-06 | 9.14E-05 | Chr2 | 83102775 | 83106294 | 1958 |
| MsG0380017648.01 | 3.710609 | 2.87E-06 | 9.16E-05 | Chr3 | 99046725 | 99051078 | 1198 |
| MsG0880045351.01 | 4.248451 | 2.88E-06 | 9.17E-05 | Chr8 | 57937049 | 57938244 | 462 |
| MsG0680035724.01 | 8.170024 | 2.88E-06 | 9.18E-05 | Chr6 | 1.11E+08 | 1.11E+08 | 609 |
| MsG0280006388.01 | -6.58599 | 2.89E-06 | 9.18E-05 | Chr2 | 1213648 | 1223567 | 1803 |
| novel.7961 | 7.00103 | 2.90E-06 | 9.20E-05 | Chr8 | 30649238 | 30650664 | 1218 |
| MsG0180002793.01 | 7.333818 | 2.90E-06 | 9.21E-05 | Chr1 | 45222684 | 45225023 | 1485 |
| novel.2697 | -6.9641 | 2.92E-06 | 9.25E-05 | Chr3 | 4302217 | 4304576 | 1437 |
| novel.7691 | -2.91046 | 2.94E-06 | 9.30E-05 | Chr7 | 82910235 | 82914100 | 2537 |
| MsG0280009735.01 | 7.017226 | 2.97E-06 | 9.39E-05 | Chr2 | 57322482 | 57322769 | 288 |
| MsG0880043454.01 | 1.841882 | 2.97E-06 | 9.40E-05 | Chr8 | 25047509 | 25061079 | 5040 |
| MsG0080047898.01 | 6.886419 | 3.01E-06 | 9.49E-05 | contig13end | 10480 | 14473 | 756 |
| novel.1221 | 7.442959 | 3.06E-06 | 9.66E-05 | Chr2 | 8749809 | 8752691 | 777 |
| MsG0180003708.01 | 2.081479 | 3.10E-06 | 9.77E-05 | Chr1 | 67158725 | 67165380 | 1002 |
| novel.6807 | 4.693429 | 3.11E-06 | 9.80E-05 | Chr7 | 3107498 | 3115416 | 1140 |
| MsG0480019232.01 | -7.21264 | 3.14E-06 | 9.89E-05 | Chr4 | 16657224 | 16666989 | 1391 |
| MsG0580029673.01 | -2.97765 | 3.15E-06 | 9.89E-05 | Chr5 | 1.01E+08 | 1.01E+08 | 1167 |
| MsG0280009743.01 | -5.9031 | 3.18E-06 | 9.96E-05 | Chr2 | 57445965 | 57447044 | 1080 |
| novel.2167 | 3.654244 | 3.18E-06 | 9.96E-05 | Chr3 | 7933800 | 7961863 | 2519 |
| MsG0580030133.01 | -3.4411 | 3.20E-06 | 0.0001 | Chr5 | 1.07E+08 | 1.07E+08 | 1836 |
| MsG0280010882.01 | 2.996666 | 3.26E-06 | 0.000102 | Chr2 | 76321135 | 76326932 | 1018 |
| novel.2516 | 4.004726 | 3.27E-06 | 0.000102 | Chr3 | 81418026 | 81422508 | 2866 |
| novel.7287 | 7.701908 | 3.31E-06 | 0.000103 | Chr7 | 94296972 | 94300513 | 1142 |
| MsG0580024562.01 | -7.06491 | 3.34E-06 | 0.000104 | Chr5 | 6367368 | 6368579 | 1212 |
| MsG0480018883.01 | 2.372204 | 3.38E-06 | 0.000105 | Chr4 | 11056592 | 11062409 | 2265 |
| novel.4987 | 6.993772 | 3.43E-06 | 0.000107 | Chr5 | 13975469 | 13980432 | 1278 |
| MsG0480022431.01 | 4.686932 | 3.48E-06 | 0.000108 | Chr4 | 72505364 | 72508035 | 1542 |
| novel.2311 | -3.81192 | 3.51E-06 | 0.000109 | Chr3 | 35633438 | 35639759 | 1095 |
| MsG0780040785.01 | -7.11604 | 3.54E-06 | 0.00011 | Chr7 | 82410949 | 82419317 | 4097 |
| novel.4462 | -7.1642 | 3.55E-06 | 0.00011 | Chr5 | 27216899 | 27218400 | 716 |
| novel.6259 | 1.95672 | 3.58E-06 | 0.000111 | Chr6 | 17044329 | 17047942 | 2090 |
| MsG0480023468.01 | 7.005716 | 3.61E-06 | 0.000112 | Chr4 | 85616960 | 85618259 | 537 |
| MsG0280010055.01 | -5.35169 | 3.63E-06 | 0.000112 | Chr2 | 63555467 | 63556774 | 1308 |
| MsG0380015514.01 | -3.00161 | 3.78E-06 | 0.000117 | Chr3 | 71281944 | 71283919 | 1056 |
| MsG0180001752.01 | -3.96991 | 3.81E-06 | 0.000117 | Chr1 | 26417450 | 26422230 | 1746 |
| novel.3288 | 3.2058 | 3.82E-06 | 0.000117 | Chr4 | 12460015 | 12465897 | 4731 |
| MsG0480022989.01 | 2.300897 | 3.82E-06 | 0.000117 | Chr4 | 79475534 | 79476919 | 1386 |
| MsG0180001402.01 | -3.29757 | 3.82E-06 | 0.000117 | Chr1 | 20597148 | 20610812 | 7800 |
| novel.2945 | -7.00045 | 3.88E-06 | 0.000119 | Chr3 | 60765796 | 60767477 | 1682 |
| novel.1258 | -7.34785 | 3.91E-06 | 0.00012 | Chr2 | 15177286 | 15178220 | 833 |
| MsG0480022039.01 | 1.724727 | 3.92E-06 | 0.00012 | Chr4 | 67268508 | 67271317 | 1260 |
| MsG0380012957.01 | -6.98352 | 3.94E-06 | 0.00012 | Chr3 | 27649405 | 27652403 | 1362 |
| MsG0780041226.01 | -4.37255 | 3.95E-06 | 0.000121 | Chr7 | 88457894 | 88458424 | 531 |
| MsG0380014376.01 | -4.26119 | 3.98E-06 | 0.000121 | Chr3 | 53811527 | 53814271 | 2745 |
| MsG0680034771.01 | -3.53854 | 3.99E-06 | 0.000122 | Chr6 | 94269747 | 94277000 | 1683 |
| MsG0380012948.01 | -7.69205 | 4.01E-06 | 0.000122 | Chr3 | 27475621 | 27476094 | 474 |
| novel.305 | -4.73092 | 4.01E-06 | 0.000122 | Chr1 | 56881933 | 56884256 | 2129 |
| MsG0580025157.01 | -2.38406 | 4.05E-06 | 0.000123 | Chr5 | 14606517 | 14610710 | 1605 |
| novel.7246 | 7.373568 | 4.16E-06 | 0.000126 | Chr7 | 89412966 | 89413503 | 310 |
| MsG0880042070.01 | 1.454961 | 4.20E-06 | 0.000127 | Chr8 | 3357771 | 3362716 | 3084 |
| MsG0380016168.01 | 7.179239 | 4.21E-06 | 0.000128 | Chr3 | 79940485 | 79942588 | 1236 |
| novel.6633 | 7.335952 | 4.24E-06 | 0.000128 | Chr6 | 89775299 | 89775969 | 431 |
| MsG0580029006.01 | -3.09329 | 4.25E-06 | 0.000128 | Chr5 | 90199487 | 90200134 | 408 |
| novel.7991 | 6.45805 | 4.26E-06 | 0.000129 | Chr8 | 38291176 | 38291588 | 285 |
| MsG0880044640.01 | -2.76367 | 4.27E-06 | 0.000129 | Chr8 | 46103126 | 46106436 | 2337 |
| MsG0380012005.01 | -1.93052 | 4.28E-06 | 0.000129 | Chr3 | 9418398 | 9424434 | 1862 |
| novel.2786 | 6.543811 | 4.29E-06 | 0.000129 | Chr3 | 20686155 | 20690842 | 936 |
| MsG0480021350.01 | 6.545577 | 4.29E-06 | 0.000129 | Chr4 | 56940167 | 56941044 | 717 |
| MsG0380013128.01 | -5.01015 | 4.33E-06 | 0.00013 | Chr3 | 30933916 | 30936289 | 757 |
| MsG0380016416.01 | -7.61597 | 4.35E-06 | 0.000131 | Chr3 | 83147891 | 83156537 | 4602 |
| MsG0380012422.01 | 4.311418 | 4.38E-06 | 0.000131 | Chr3 | 16242223 | 16243136 | 729 |
| novel.6762 | -2.35944 | 4.43E-06 | 0.000133 | Chr6 | 1.1E+08 | 1.1E+08 | 1501 |
| novel.2391 | 5.138042 | 4.47E-06 | 0.000134 | Chr3 | 57372342 | 57373245 | 786 |
| novel.8688 | 4.455742 | 4.51E-06 | 0.000135 | Chr8 | 74836207 | 74837657 | 781 |
| MsG0280009942.01 | -6.07712 | 4.54E-06 | 0.000136 | Chr2 | 61391955 | 61394744 | 1491 |
| novel.2923 | -3.76771 | 4.57E-06 | 0.000136 | Chr3 | 54266727 | 54268772 | 1624 |
| MsG0680035770.01 | -2.60308 | 4.60E-06 | 0.000137 | Chr6 | 1.12E+08 | 1.12E+08 | 801 |
| MsG0580027687.01 | -6.90055 | 4.63E-06 | 0.000138 | Chr5 | 66148582 | 66156040 | 2253 |
| MsG0580024079.01 | -2.80179 | 4.63E-06 | 0.000138 | Chr5 | 564185 | 566380 | 849 |
| MsG0180003213.01 | -4.43453 | 4.69E-06 | 0.000139 | Chr1 | 58490602 | 58491965 | 1260 |
| novel.3915 | 10.1229 | 4.70E-06 | 0.00014 | Chr4 | 33342752 | 33342969 | 218 |
| MsG0580026516.01 | -2.24126 | 4.70E-06 | 0.00014 | Chr5 | 38754038 | 38755088 | 633 |
| MsG0580030135.01 | -3.24793 | 4.74E-06 | 0.000141 | Chr5 | 1.07E+08 | 1.07E+08 | 1243 |
| MsG0180001533.01 | -3.99477 | 4.78E-06 | 0.000141 | Chr1 | 22827909 | 22842513 | 3288 |
| MsG0180001724.01 | -3.50226 | 4.81E-06 | 0.000142 | Chr1 | 26071057 | 26071290 | 234 |
| MsG0480020090.01 | 2.322783 | 4.82E-06 | 0.000143 | Chr4 | 33844238 | 33847616 | 1668 |
| MsG0580025279.01 | 2.203693 | 4.87E-06 | 0.000144 | Chr5 | 16387777 | 16395933 | 2005 |
| novel.367 | 5.93443 | 4.87E-06 | 0.000144 | Chr1 | 66439298 | 66441773 | 2476 |
| MsG0780039607.01 | -4.08841 | 4.89E-06 | 0.000144 | Chr7 | 66658017 | 66658520 | 504 |
| MsG0780040755.01 | -3.50595 | 5.01E-06 | 0.000147 | Chr7 | 81990704 | 82016855 | 2421 |
| MsG0280010021.01 | -1.96485 | 5.02E-06 | 0.000148 | Chr2 | 62973240 | 62984916 | 2348 |
| novel.3996 | 5.254752 | 5.05E-06 | 0.000148 | Chr4 | 53672323 | 53674977 | 1153 |
| MsG0580025683.01 | -2.2766 | 5.06E-06 | 0.000149 | Chr5 | 22602190 | 22605024 | 1371 |
| novel.6242 | -6.41493 | 5.08E-06 | 0.000149 | Chr6 | 14821447 | 14839924 | 343 |
| MsG0180003938.01 | 1.551832 | 5.13E-06 | 0.00015 | Chr1 | 70600279 | 70609536 | 2018 |
| MsG0080048653.01 | 6.94864 | 5.17E-06 | 0.000151 | contig392end | 22707 | 24162 | 1156 |
| MsG0180001988.01 | -2.46609 | 5.25E-06 | 0.000153 | Chr1 | 30384576 | 30392007 | 2232 |
| novel.6714 | -7.33065 | 5.25E-06 | 0.000153 | Chr6 | 1.04E+08 | 1.04E+08 | 382 |
| MsG0180004976.01 | 3.536553 | 5.31E-06 | 0.000155 | Chr1 | 85106242 | 85109722 | 2475 |
| MsG0280007643.01 | 3.19782 | 5.33E-06 | 0.000155 | Chr2 | 18470044 | 18471849 | 584 |
| MsG0380015416.01 | -3.8841 | 5.37E-06 | 0.000157 | Chr3 | 69875772 | 69881924 | 1053 |
| novel.3479 | 5.927406 | 5.39E-06 | 0.000157 | Chr4 | 52918464 | 52921433 | 1419 |
| novel.6673 | 7.108516 | 5.43E-06 | 0.000158 | Chr6 | 98985301 | 98987141 | 1478 |
| MsG0280010715.01 | -1.70158 | 5.43E-06 | 0.000158 | Chr2 | 73799805 | 73810596 | 2189 |
| novel.902 | 6.165632 | 5.52E-06 | 0.00016 | Chr1 | 60064770 | 60065933 | 922 |
| novel.5699 | -5.31636 | 5.56E-06 | 0.000161 | Chr6 | 32312527 | 32314406 | 883 |
| MsG0780039885.01 | -2.63138 | 5.59E-06 | 0.000162 | Chr7 | 70092861 | 70098140 | 1856 |
| MsG0380013908.01 | -3.51359 | 5.62E-06 | 0.000163 | Chr3 | 48138656 | 48140098 | 1443 |
| MsG0080047995.01 | 7.018134 | 5.63E-06 | 0.000163 | contig163end | 13270 | 14356 | 813 |
| MsG0280009960.01 | -2.79436 | 5.69E-06 | 0.000164 | Chr2 | 61864637 | 61873263 | 3304 |
| MsG0480022309.01 | -3.78181 | 5.79E-06 | 0.000167 | Chr4 | 70980208 | 70982494 | 2033 |
| MsG0880046332.01 | -7.62539 | 5.80E-06 | 0.000167 | Chr8 | 72111940 | 72112941 | 1002 |
| MsG0480019055.01 | -2.01996 | 5.83E-06 | 0.000168 | Chr4 | 13965488 | 13968955 | 1803 |
| MsG0680035610.01 | -2.34627 | 5.88E-06 | 0.000169 | Chr6 | 1.09E+08 | 1.09E+08 | 2056 |
| MsG0480019799.01 | 4.455772 | 5.90E-06 | 0.000169 | Chr4 | 27506284 | 27509903 | 3222 |
| MsG0780038922.01 | -3.0643 | 5.92E-06 | 0.00017 | Chr7 | 55389194 | 55390687 | 678 |
| MsG0680032542.01 | -6.97005 | 5.98E-06 | 0.000172 | Chr6 | 44205531 | 44206894 | 1081 |
| MsG0780040032.01 | -6.84973 | 6.00E-06 | 0.000172 | Chr7 | 72221843 | 72228415 | 732 |
| novel.4423 | 7.115005 | 6.04E-06 | 0.000173 | Chr5 | 22717079 | 22718458 | 1380 |
| MsG0280007869.01 | -4.93934 | 6.10E-06 | 0.000174 | Chr2 | 22061651 | 22065100 | 1388 |
| MsG0080048355.01 | 6.829233 | 6.11E-06 | 0.000175 | contig280end | 16641 | 21783 | 2349 |
| MsG0480020067.01 | 2.41468 | 6.24E-06 | 0.000178 | Chr4 | 33277544 | 33281817 | 1216 |
| novel.2050 | 1.816521 | 6.25E-06 | 0.000178 | Chr2 | 76181430 | 76187461 | 2464 |
| MsG0480019312.01 | -4.16126 | 6.26E-06 | 0.000178 | Chr4 | 18338092 | 18338583 | 492 |
| novel.123 | 4.01517 | 6.29E-06 | 0.000179 | Chr1 | 16299922 | 16300370 | 449 |
| MsG0380014168.01 | 5.740142 | 6.31E-06 | 0.000179 | Chr3 | 50805251 | 50807780 | 1449 |
| MsG0180004053.01 | -3.50543 | 6.32E-06 | 0.00018 | Chr1 | 72523965 | 72527724 | 2305 |
| novel.5977 | 2.58596 | 6.35E-06 | 0.00018 | Chr6 | 93130390 | 93137788 | 2362 |
| novel.7537 | 7.001782 | 6.43E-06 | 0.000182 | Chr7 | 58435019 | 58437528 | 1536 |
| novel.316 | 6.371173 | 6.44E-06 | 0.000182 | Chr1 | 59699255 | 59702575 | 2079 |
| MsG0680031368.01 | 1.846786 | 6.46E-06 | 0.000183 | Chr6 | 19095632 | 19098756 | 941 |
| MsG0880044994.01 | 5.555147 | 6.48E-06 | 0.000183 | Chr8 | 52543467 | 52549606 | 3353 |
| novel.3665 | -4.25987 | 6.54E-06 | 0.000185 | Chr4 | 83188171 | 83188917 | 747 |
| MsG0780039272.01 | 1.792347 | 6.55E-06 | 0.000185 | Chr7 | 61113430 | 61120078 | 1231 |
| MsG0780037249.01 | 6.937628 | 6.60E-06 | 0.000186 | Chr7 | 22448329 | 22449604 | 876 |
| MsG0180000333.01 | 6.379595 | 6.63E-06 | 0.000187 | Chr1 | 4547724 | 4548041 | 318 |
| MsG0880045744.01 | 4.484864 | 6.64E-06 | 0.000187 | Chr8 | 63666568 | 63666882 | 315 |
| MsG0880042478.01 | -7.52811 | 6.64E-06 | 0.000187 | Chr8 | 9358253 | 9362877 | 3099 |
| novel.6822 | -4.35006 | 6.81E-06 | 0.000191 | Chr7 | 8699321 | 8701915 | 1285 |
| novel.4636 | -3.09138 | 6.83E-06 | 0.000192 | Chr5 | 65313353 | 65318389 | 1673 |
| novel.2971 | 4.491449 | 6.87E-06 | 0.000193 | Chr3 | 66500544 | 66503817 | 3274 |
| novel.2970 | 5.949872 | 6.88E-06 | 0.000193 | Chr3 | 66427407 | 66432226 | 4820 |
| novel.8113 | -7.01641 | 6.91E-06 | 0.000193 | Chr8 | 61634372 | 61635395 | 391 |
| MsG0780041281.01 | -4.63753 | 7.05E-06 | 0.000197 | Chr7 | 89138931 | 89144912 | 2169 |
| MsG0380012835.01 | -2.15792 | 7.05E-06 | 0.000197 | Chr3 | 25313947 | 25320764 | 1273 |
| MsG0480019256.01 | -5.88243 | 7.07E-06 | 0.000197 | Chr4 | 17225240 | 17231240 | 1149 |
| novel.1793 | 7.341205 | 7.13E-06 | 0.000199 | Chr2 | 22938357 | 22942646 | 731 |
| MsG0380015738.01 | 1.511139 | 7.15E-06 | 0.000199 | Chr3 | 74471025 | 74475313 | 912 |
| MsG0480020818.01 | 4.654121 | 7.17E-06 | 0.000199 | Chr4 | 48834127 | 48835002 | 876 |
| MsG0280009716.01 | 1.87286 | 7.18E-06 | 0.000199 | Chr2 | 57060131 | 57061342 | 777 |
| MsG0480021644.01 | 2.925393 | 7.30E-06 | 0.000203 | Chr4 | 61186641 | 61189408 | 1518 |
| MsG0580029435.01 | -6.85508 | 7.32E-06 | 0.000203 | Chr5 | 96951254 | 96955258 | 1698 |
| novel.5124 | -6.83232 | 7.35E-06 | 0.000204 | Chr5 | 44143479 | 44144481 | 827 |
| MsG0180001586.01 | 7.172648 | 7.35E-06 | 0.000204 | Chr1 | 23749562 | 23752851 | 3042 |
| novel.4630 | 3.466751 | 7.37E-06 | 0.000204 | Chr5 | 63777779 | 63782901 | 1890 |
| MsG0280006430.01 | 4.839261 | 7.38E-06 | 0.000204 | Chr2 | 1853264 | 1853479 | 216 |
| MsG0380013875.01 | -4.47059 | 7.38E-06 | 0.000204 | Chr3 | 47156969 | 47161145 | 1337 |
| MsG0680030915.01 | -3.54377 | 7.38E-06 | 0.000204 | Chr6 | 11487917 | 11489698 | 1458 |
| MsG0880043345.01 | -3.66068 | 7.39E-06 | 0.000204 | Chr8 | 23410286 | 23417575 | 3782 |
| MsG0880045880.01 | -2.23452 | 7.41E-06 | 0.000204 | Chr8 | 65917805 | 65927989 | 2020 |
| MsG0680031212.01 | 7.50058 | 7.50E-06 | 0.000206 | Chr6 | 16088704 | 16090001 | 1017 |
| novel.8393 | 2.544928 | 7.50E-06 | 0.000206 | Chr8 | 18009228 | 18011028 | 785 |
| novel.532 | -2.81268 | 7.53E-06 | 0.000207 | Chr1 | 94171256 | 94172702 | 1447 |
| novel.5273 | 4.764154 | 7.54E-06 | 0.000207 | Chr5 | 73377872 | 73380586 | 1202 |
| novel.2930 | 7.314982 | 7.56E-06 | 0.000207 | Chr3 | 56563350 | 56564056 | 707 |
| MsG0480020883.01 | 2.592581 | 7.57E-06 | 0.000207 | Chr4 | 50058632 | 50063214 | 2155 |
| MsG0180006126.01 | -3.62893 | 7.76E-06 | 0.000212 | Chr1 | 1E+08 | 1E+08 | 925 |
| MsG0680030290.01 | 2.125895 | 7.80E-06 | 0.000213 | Chr6 | 149705 | 150106 | 402 |
| MsG0380014509.01 | -2.42412 | 7.84E-06 | 0.000214 | Chr3 | 55876854 | 55877330 | 477 |
| novel.5575 | -5.19404 | 7.86E-06 | 0.000214 | Chr6 | 11032701 | 11033686 | 986 |
| MsG0680031452.01 | 3.663595 | 7.90E-06 | 0.000215 | Chr6 | 20672096 | 20684199 | 1885 |
| novel.1657 | -1.99057 | 7.91E-06 | 0.000215 | Chr2 | 1855553 | 1860595 | 2808 |
| MsG0180002649.01 | 4.916412 | 7.98E-06 | 0.000217 | Chr1 | 42365392 | 42372267 | 3465 |
| MsG0280009621.01 | -3.00087 | 7.99E-06 | 0.000217 | Chr2 | 55454756 | 55459247 | 1650 |
| MsG0480020488.01 | -3.55885 | 7.99E-06 | 0.000217 | Chr4 | 42336819 | 42338253 | 1071 |
| MsG0780039098.01 | 7.840959 | 8.08E-06 | 0.000219 | Chr7 | 57914539 | 57918550 | 864 |
| novel.7331 | -6.93229 | 8.10E-06 | 0.000219 | Chr7 | 10372501 | 10375958 | 1571 |
| MsG0580028788.01 | 4.55932 | 8.11E-06 | 0.000219 | Chr5 | 86466724 | 86470429 | 2812 |
| MsG0580028821.01 | 5.905543 | 8.17E-06 | 0.000221 | Chr5 | 86985505 | 86990169 | 3075 |
| MsG0580024394.01 | 2.050123 | 8.27E-06 | 0.000224 | Chr5 | 4240804 | 4252178 | 2583 |
| MsG0580026905.01 | 7.842927 | 8.31E-06 | 0.000224 | Chr5 | 47813735 | 47815951 | 2217 |
| novel.3190 | -6.94172 | 8.38E-06 | 0.000226 | Chr3 | 1E+08 | 1E+08 | 562 |
| MsG0280010892.01 | 4.624294 | 8.41E-06 | 0.000227 | Chr2 | 76460629 | 76461149 | 408 |
| MsG0280007868.01 | -2.31128 | 8.52E-06 | 0.000229 | Chr2 | 22042556 | 22043593 | 1038 |
| MsG0780040431.01 | -7.68762 | 8.53E-06 | 0.000229 | Chr7 | 77420020 | 77423982 | 1196 |
| novel.5289 | -6.95599 | 8.55E-06 | 0.00023 | Chr5 | 75768937 | 75773248 | 1706 |
| MsG0580029599.01 | 4.607521 | 8.58E-06 | 0.00023 | Chr5 | 99198915 | 99201257 | 441 |
| MsG0180000782.01 | -5.67259 | 8.59E-06 | 0.00023 | Chr1 | 11073896 | 11077783 | 807 |
| novel.5027 | 6.978414 | 8.76E-06 | 0.000235 | Chr5 | 22631037 | 22632660 | 752 |
| novel.8103 | -3.21909 | 8.78E-06 | 0.000235 | Chr8 | 59665994 | 59667927 | 1693 |
| MsG0480023114.01 | 4.699572 | 8.86E-06 | 0.000237 | Chr4 | 81090252 | 81090470 | 219 |
| novel.6392 | 3.436373 | 8.94E-06 | 0.000239 | Chr6 | 39536623 | 39539611 | 2751 |
| MsG0280006826.01 | -3.48178 | 8.96E-06 | 0.000239 | Chr2 | 6800215 | 6801977 | 1011 |
| novel.596 | -3.78558 | 9.00E-06 | 0.00024 | Chr1 | 1993097 | 1994866 | 1185 |
| MsG0180001403.01 | -6.91662 | 9.02E-06 | 0.000241 | Chr1 | 20614682 | 20618065 | 1359 |
| MsG0580027344.01 | -2.72494 | 9.06E-06 | 0.000241 | Chr5 | 58772222 | 58775375 | 391 |
| MsG0880044877.01 | -2.55422 | 9.08E-06 | 0.000242 | Chr8 | 50634110 | 50644383 | 2010 |
| novel.6810 | -4.3903 | 9.11E-06 | 0.000242 | Chr7 | 4800035 | 4801023 | 795 |
| MsG0280011406.01 | -2.38409 | 9.11E-06 | 0.000242 | Chr2 | 83542943 | 83545218 | 1019 |
| MsG0180000965.01 | -3.80435 | 9.17E-06 | 0.000243 | Chr1 | 13844403 | 13846189 | 1692 |
| MsG0380016365.01 | -7.11162 | 9.20E-06 | 0.000244 | Chr3 | 82524668 | 82525021 | 354 |
| MsG0180001591.01 | -2.41887 | 9.27E-06 | 0.000246 | Chr1 | 23844028 | 23846786 | 2094 |
| MsG0480023544.01 | 2.911819 | 9.38E-06 | 0.000248 | Chr4 | 86706845 | 86708881 | 618 |
| MsG0880042294.01 | 1.845559 | 9.40E-06 | 0.000249 | Chr8 | 6641969 | 6647371 | 2490 |
| MsG0280010723.01 | 1.87898 | 9.46E-06 | 0.00025 | Chr2 | 73910920 | 73915076 | 1650 |
| MsG0580027955.01 | -3.36881 | 9.52E-06 | 0.000251 | Chr5 | 71760800 | 71761403 | 207 |
| MsG0480020595.01 | -3.08349 | 9.55E-06 | 0.000252 | Chr4 | 44303785 | 44304975 | 1191 |
| novel.6330 | -4.55015 | 9.68E-06 | 0.000255 | Chr6 | 26201007 | 26202911 | 959 |
| MsG0380014133.01 | -5.6519 | 9.73E-06 | 0.000256 | Chr3 | 50233254 | 50235472 | 720 |
| MsG0480022378.01 | 2.913413 | 9.76E-06 | 0.000257 | Chr4 | 71788553 | 71791464 | 873 |
| MsG0480023519.01 | 2.615029 | 9.77E-06 | 0.000257 | Chr4 | 86415063 | 86416331 | 1269 |
| MsG0180005450.01 | 4.040492 | 9.79E-06 | 0.000257 | Chr1 | 91989108 | 91993000 | 2335 |
| MsG0880042998.01 | -4.64393 | 9.81E-06 | 0.000257 | Chr8 | 17353481 | 17353714 | 234 |
| MsG0780038930.01 | -3.89334 | 9.84E-06 | 0.000258 | Chr7 | 55496339 | 55497916 | 675 |
| MsG0680031695.01 | -6.13398 | 9.84E-06 | 0.000258 | Chr6 | 24780040 | 24781096 | 513 |
| novel.3694 | 1.69665 | 9.95E-06 | 0.00026 | Chr4 | 85934309 | 85944207 | 2454 |
| MsG0480019197.01 | 7.564096 | 1.01E-05 | 0.000263 | Chr4 | 15943442 | 15946045 | 2604 |
| MsG0380013732.01 | -6.83921 | 1.01E-05 | 0.000263 | Chr3 | 44722220 | 44728900 | 1026 |
| novel.479 | 7.618378 | 1.01E-05 | 0.000265 | Chr1 | 85838544 | 85839799 | 479 |
| MsG0880046916.01 | -3.73335 | 1.02E-05 | 0.000265 | Chr8 | 79819217 | 79825877 | 963 |
| MsG0080047804.01 | -5.89642 | 1.02E-05 | 0.000265 | contig108end | 27427 | 27735 | 309 |
| MsG0480022412.01 | -4.288 | 1.03E-05 | 0.000267 | Chr4 | 72231406 | 72233147 | 1326 |
| novel.2960 | 6.773086 | 1.04E-05 | 0.000271 | Chr3 | 62596381 | 62599409 | 844 |
| novel.6018 | -2.84552 | 1.04E-05 | 0.000271 | Chr6 | 1E+08 | 1E+08 | 3050 |
| MsG0680030746.01 | -1.6612 | 1.04E-05 | 0.000271 | Chr6 | 8483450 | 8493797 | 4394 |
| novel.4678 | -2.33383 | 1.04E-05 | 0.000271 | Chr5 | 73842044 | 73845649 | 1876 |
| MsG0580026050.01 | 2.029115 | 1.04E-05 | 0.000271 | Chr5 | 29854061 | 29860967 | 857 |
| MsG0180005020.01 | -6.96737 | 1.05E-05 | 0.000272 | Chr1 | 85732657 | 85746629 | 1125 |
| MsG0780041290.01 | -4.02123 | 1.05E-05 | 0.000272 | Chr7 | 89227842 | 89228198 | 357 |
| MsG0180006217.01 | 1.816541 | 1.06E-05 | 0.000274 | Chr1 | 1.02E+08 | 1.02E+08 | 384 |
| MsG0680035603.01 | -2.41417 | 1.06E-05 | 0.000274 | Chr6 | 1.09E+08 | 1.09E+08 | 327 |
| MsG0680034240.01 | 5.586898 | 1.07E-05 | 0.000276 | Chr6 | 84241540 | 84242202 | 663 |
| novel.2720 | -4.19056 | 1.08E-05 | 0.000278 | Chr3 | 8202034 | 8207816 | 2035 |
| novel.5369 | 6.80501 | 1.09E-05 | 0.000282 | Chr5 | 90225893 | 90227727 | 631 |
| MsG0180003527.01 | 1.491364 | 1.10E-05 | 0.000283 | Chr1 | 63674617 | 63676270 | 342 |
| novel.6437 | -6.7812 | 1.10E-05 | 0.000283 | Chr6 | 47238111 | 47241871 | 597 |
| MsG0280008218.01 | -7.43162 | 1.11E-05 | 0.000285 | Chr2 | 27330494 | 27331191 | 489 |
| MsG0180004744.01 | -3.51968 | 1.11E-05 | 0.000286 | Chr1 | 82038118 | 82042706 | 1461 |
| novel.211 | -6.87717 | 1.11E-05 | 0.000286 | Chr1 | 30694699 | 30696850 | 840 |
| MsG0780037938.01 | -6.89835 | 1.11E-05 | 0.000286 | Chr7 | 36577816 | 36580524 | 1944 |
| MsG0280006598.01 | 7.199821 | 1.12E-05 | 0.000286 | Chr2 | 3856943 | 3859401 | 1878 |
| MsG0680030992.01 | 1.951567 | 1.13E-05 | 0.000288 | Chr6 | 12623176 | 12623687 | 512 |
| novel.2336 | 6.774423 | 1.13E-05 | 0.000288 | Chr3 | 42718753 | 42721099 | 2062 |
| MsG0180005076.01 | -2.23182 | 1.13E-05 | 0.000289 | Chr1 | 86562917 | 86563597 | 681 |
| MsG0580029429.01 | -2.06301 | 1.14E-05 | 0.00029 | Chr5 | 96893010 | 96896755 | 1830 |
| novel.3738 | 6.892683 | 1.14E-05 | 0.00029 | Chr4 | 91181364 | 91182468 | 620 |
| novel.6767 | -2.26619 | 1.14E-05 | 0.000291 | Chr6 | 1.1E+08 | 1.1E+08 | 983 |
| MsG0780039591.01 | -2.50164 | 1.14E-05 | 0.000291 | Chr7 | 66393721 | 66399370 | 2894 |
| MsG0780041761.01 | -1.50839 | 1.16E-05 | 0.000294 | Chr7 | 94828496 | 94833267 | 1617 |
| novel.7913 | 6.843793 | 1.16E-05 | 0.000294 | Chr8 | 21364323 | 21368562 | 1141 |
| MsG0480022738.01 | 4.047151 | 1.17E-05 | 0.000297 | Chr4 | 76183272 | 76184675 | 1404 |
| MsG0780039097.01 | 7.131694 | 1.17E-05 | 0.000297 | Chr7 | 57904656 | 57909973 | 1518 |
| MsG0380013784.01 | 3.247104 | 1.18E-05 | 0.000299 | Chr3 | 45652867 | 45655203 | 2337 |
| MsG0380017538.01 | -7.39116 | 1.18E-05 | 0.000299 | Chr3 | 97632900 | 97633697 | 540 |
| MsG0780038807.01 | -2.96605 | 1.19E-05 | 0.000301 | Chr7 | 53321320 | 53323492 | 886 |
| MsG0280009515.01 | 4.654997 | 1.20E-05 | 0.000303 | Chr2 | 53454345 | 53459313 | 2475 |
| novel.2491 | 2.107465 | 1.21E-05 | 0.000304 | Chr3 | 77442629 | 77445233 | 1251 |
| MsG0280008675.01 | 3.908197 | 1.21E-05 | 0.000306 | Chr2 | 36149099 | 36155485 | 1887 |
| MsG0680031429.01 | -4.27339 | 1.21E-05 | 0.000306 | Chr6 | 20338399 | 20343848 | 1368 |
| MsG0280006429.01 | 4.779486 | 1.22E-05 | 0.000307 | Chr2 | 1850243 | 1851661 | 1419 |
| MsG0580025303.01 | 7.245326 | 1.23E-05 | 0.000309 | Chr5 | 16715057 | 16719450 | 1467 |
| novel.6118 | -3.17793 | 1.26E-05 | 0.000317 | Chr6 | 1.12E+08 | 1.12E+08 | 442 |
| novel.1706 | 2.27131 | 1.27E-05 | 0.000318 | Chr2 | 7692560 | 7694219 | 1660 |
| MsG0380011844.01 | 2.823599 | 1.27E-05 | 0.000319 | Chr3 | 6270342 | 6276159 | 676 |
| MsG0080047789.01 | 7.403533 | 1.28E-05 | 0.00032 | contig102end | 22983 | 27033 | 1032 |
| novel.2992 | -2.03814 | 1.28E-05 | 0.00032 | Chr3 | 69868526 | 69870223 | 1698 |
| MsG0480018934.01 | 4.451939 | 1.28E-05 | 0.000321 | Chr4 | 11947565 | 11957070 | 5664 |
| MsG0680031108.01 | 2.421117 | 1.31E-05 | 0.000328 | Chr6 | 14560739 | 14565715 | 2012 |
| MsG0680030543.01 | -2.47681 | 1.31E-05 | 0.000328 | Chr6 | 4999251 | 5002151 | 1937 |
| MsG0380017394.01 | 3.358801 | 1.32E-05 | 0.000328 | Chr3 | 95767046 | 95767681 | 636 |
| novel.3842 | -4.90423 | 1.32E-05 | 0.000328 | Chr4 | 17995096 | 17997628 | 1006 |
| novel.3244 | 6.817743 | 1.32E-05 | 0.000328 | Chr4 | 4894366 | 4897682 | 1892 |
| MsG0880044915.01 | -6.71405 | 1.32E-05 | 0.00033 | Chr8 | 51362437 | 51362925 | 489 |
| MsG0580024355.01 | -7.21186 | 1.35E-05 | 0.000335 | Chr5 | 3794125 | 3798899 | 3618 |
| MsG0780041556.01 | -2.94117 | 1.35E-05 | 0.000335 | Chr7 | 92366789 | 92376425 | 4394 |
| novel.5128 | 7.153167 | 1.35E-05 | 0.000336 | Chr5 | 44597053 | 44602502 | 476 |
| novel.4737 | -1.92137 | 1.36E-05 | 0.000337 | Chr5 | 84413347 | 84418934 | 936 |
| novel.6247 | 6.775699 | 1.36E-05 | 0.000339 | Chr6 | 16156394 | 16157962 | 1569 |
| novel.7624 | 6.759848 | 1.37E-05 | 0.000339 | Chr7 | 72482242 | 72486804 | 1014 |
| novel.6851 | 7.266681 | 1.38E-05 | 0.000341 | Chr7 | 15058884 | 15060353 | 986 |
| novel.656 | -6.88316 | 1.38E-05 | 0.000342 | Chr1 | 7320848 | 7322418 | 475 |
| MsG0580024756.01 | 6.900178 | 1.38E-05 | 0.000342 | Chr5 | 9288955 | 9293235 | 800 |
| novel.3642 | 6.791293 | 1.39E-05 | 0.000343 | Chr4 | 79589694 | 79590569 | 566 |
| MsG0180005697.01 | -3.08371 | 1.39E-05 | 0.000343 | Chr1 | 95124793 | 95131231 | 1488 |
| MsG0080048752.01 | -2.70517 | 1.40E-05 | 0.000347 | contig420end | 15636 | 16954 | 1068 |
| MsG0780038118.01 | -3.25943 | 1.42E-05 | 0.00035 | Chr7 | 40602906 | 40604054 | 1149 |
| novel.5747 | -7.22162 | 1.43E-05 | 0.000353 | Chr6 | 44679075 | 44680577 | 1421 |
| novel.1112 | -5.48235 | 1.44E-05 | 0.000353 | Chr1 | 96832123 | 96833117 | 846 |
| novel.879 | 7.578222 | 1.45E-05 | 0.000355 | Chr1 | 53892205 | 53893496 | 746 |
| novel.8064 | -3.18126 | 1.46E-05 | 0.000358 | Chr8 | 52455483 | 52457920 | 1599 |
| novel.5391 | -6.84972 | 1.46E-05 | 0.000358 | Chr5 | 93562203 | 93562750 | 548 |
| MsG0480023115.01 | 4.10772 | 1.46E-05 | 0.000358 | Chr4 | 81090814 | 81091137 | 324 |
| MsG0580026848.01 | -2.85794 | 1.47E-05 | 0.00036 | Chr5 | 46358697 | 46364178 | 1002 |
| MsG0680032751.01 | 4.086976 | 1.48E-05 | 0.000361 | Chr6 | 48583915 | 48587341 | 2748 |
| MsG0680032249.01 | -3.65683 | 1.48E-05 | 0.000361 | Chr6 | 37345886 | 37348655 | 2532 |
| MsG0380012244.01 | -4.53214 | 1.52E-05 | 0.000371 | Chr3 | 13234171 | 13236044 | 936 |
| MsG0580024232.01 | -2.76802 | 1.54E-05 | 0.000375 | Chr5 | 2402713 | 2403774 | 926 |
| MsG0480021719.01 | 3.840258 | 1.54E-05 | 0.000375 | Chr4 | 63423132 | 63428386 | 1672 |
| MsG0680031840.01 | 2.935579 | 1.54E-05 | 0.000375 | Chr6 | 27838183 | 27839625 | 1443 |
| MsG0380017681.01 | -5.56002 | 1.55E-05 | 0.000378 | Chr3 | 99500120 | 99500755 | 636 |
| MsG0780036515.01 | -4.95604 | 1.59E-05 | 0.000387 | Chr7 | 9165412 | 9167939 | 1380 |
| MsG0380017397.01 | 5.144391 | 1.59E-05 | 0.000387 | Chr3 | 95805282 | 95805929 | 648 |
| MsG0380016614.01 | 2.247027 | 1.61E-05 | 0.00039 | Chr3 | 85737610 | 85741206 | 687 |
| novel.5796 | 3.626429 | 1.61E-05 | 0.000391 | Chr6 | 53554541 | 53560820 | 1800 |
| novel.5424 | -6.68231 | 1.61E-05 | 0.000391 | Chr5 | 99308625 | 99313845 | 678 |
| MsG0780040005.01 | -4.16936 | 1.62E-05 | 0.000393 | Chr7 | 71843328 | 71846047 | 1622 |
| MsG0680031042.01 | 2.405366 | 1.63E-05 | 0.000395 | Chr6 | 13200830 | 13204503 | 749 |
| MsG0480019171.01 | -2.91945 | 1.64E-05 | 0.000397 | Chr4 | 15395201 | 15400024 | 637 |
| novel.2802 | -6.66239 | 1.65E-05 | 0.000399 | Chr3 | 23752217 | 23763970 | 1133 |
| MsG0680033447.01 | -2.17915 | 1.65E-05 | 0.000399 | Chr6 | 65446429 | 65449112 | 2100 |
| MsG0780040242.01 | -1.64467 | 1.65E-05 | 0.000399 | Chr7 | 74823862 | 74836989 | 1936 |
| MsG0880045677.01 | -4.30087 | 1.66E-05 | 0.0004 | Chr8 | 62625539 | 62627153 | 390 |
| MsG0880043350.01 | 1.955177 | 1.67E-05 | 0.000402 | Chr8 | 23486629 | 23486928 | 219 |
| MsG0580026460.01 | -2.62056 | 1.68E-05 | 0.000404 | Chr5 | 37867007 | 37868093 | 712 |
| MsG0280007866.01 | 3.038935 | 1.68E-05 | 0.000404 | Chr2 | 22031040 | 22035623 | 1020 |
| novel.1214 | 6.747103 | 1.68E-05 | 0.000405 | Chr2 | 7427578 | 7429721 | 2144 |
| MsG0880046273.01 | -6.70334 | 1.68E-05 | 0.000405 | Chr8 | 71373427 | 71374177 | 654 |
| MsG0580029871.01 | 2.466821 | 1.69E-05 | 0.000405 | Chr5 | 1.03E+08 | 1.03E+08 | 1450 |
| MsG0280006664.01 | 1.640972 | 1.69E-05 | 0.000405 | Chr2 | 4665731 | 4666171 | 441 |
| MsG0680035788.01 | -2.85491 | 1.69E-05 | 0.000406 | Chr6 | 1.12E+08 | 1.12E+08 | 270 |
| MsG0080048635.01 | -4.75672 | 1.71E-05 | 0.000411 | contig382end | 7689 | 9026 | 1338 |
| novel.8075 | 6.902204 | 1.73E-05 | 0.000413 | Chr8 | 55280968 | 55281849 | 673 |
| MsG0180000649.01 | -4.14168 | 1.73E-05 | 0.000413 | Chr1 | 9201153 | 9206968 | 1311 |
| MsG0480018570.01 | -6.90117 | 1.74E-05 | 0.000415 | Chr4 | 6775567 | 6778985 | 2331 |
| MsG0580029500.01 | -2.27483 | 1.74E-05 | 0.000415 | Chr5 | 97780143 | 97800185 | 5343 |
| novel.2731 | -3.49807 | 1.75E-05 | 0.000418 | Chr3 | 9431423 | 9433850 | 1044 |
| novel.1629 | -2.76185 | 1.76E-05 | 0.000418 | Chr2 | 83042486 | 83045551 | 1233 |
| MsG0880044047.01 | 4.096433 | 1.76E-05 | 0.00042 | Chr8 | 34750687 | 34751958 | 1272 |
| MsG0480022607.01 | -2.93173 | 1.78E-05 | 0.000423 | Chr4 | 74604422 | 74606503 | 2082 |
| MsG0380011962.01 | 2.382917 | 1.78E-05 | 0.000424 | Chr3 | 8507345 | 8509702 | 812 |
| MsG0780039966.01 | -2.53378 | 1.80E-05 | 0.000428 | Chr7 | 71182724 | 71196941 | 5535 |
| novel.6196 | -6.64581 | 1.81E-05 | 0.000429 | Chr6 | 8963509 | 8965644 | 802 |
| MsG0280008658.01 | 3.210719 | 1.82E-05 | 0.000432 | Chr2 | 35998823 | 35999986 | 1164 |
| novel.478 | -3.9975 | 1.85E-05 | 0.000439 | Chr1 | 85816021 | 85816958 | 581 |
| novel.336 | 6.610584 | 1.86E-05 | 0.000441 | Chr1 | 61904089 | 61905225 | 765 |
| MsG0080047994.01 | 1.690811 | 1.87E-05 | 0.000442 | contig163end | 1052 | 2380 | 795 |
| novel.4360 | 7.107648 | 1.89E-05 | 0.000447 | Chr5 | 12132912 | 12137267 | 4266 |
| MsG0680034239.01 | 3.810252 | 1.90E-05 | 0.000448 | Chr6 | 84237662 | 84238312 | 651 |
| novel.3231 | 6.75914 | 1.90E-05 | 0.000448 | Chr4 | 3139995 | 3143656 | 1540 |
| MsG0480018158.01 | -6.76954 | 1.90E-05 | 0.000449 | Chr4 | 997994 | 1005278 | 2664 |
| MsG0180000556.01 | 2.341091 | 1.92E-05 | 0.000451 | Chr1 | 7755694 | 7782470 | 4995 |
| MsG0180005199.01 | -1.46841 | 1.92E-05 | 0.000452 | Chr1 | 88374752 | 88379061 | 1540 |
| MsG0880045339.01 | 4.262174 | 1.92E-05 | 0.000453 | Chr8 | 57766196 | 57768480 | 1845 |
| novel.2016 | 6.658586 | 1.93E-05 | 0.000454 | Chr2 | 71292770 | 71295371 | 1151 |
| novel.3296 | 4.725931 | 1.93E-05 | 0.000454 | Chr4 | 13708653 | 13713303 | 1145 |
| MsG0880046449.01 | 2.02243 | 1.95E-05 | 0.000459 | Chr8 | 73541908 | 73546566 | 912 |
| novel.3445 | -2.36125 | 1.96E-05 | 0.000459 | Chr4 | 44531973 | 44547112 | 3128 |
| novel.8486 | 2.984764 | 1.96E-05 | 0.00046 | Chr8 | 37668015 | 37781870 | 2574 |
| novel.7845 | 3.852609 | 1.97E-05 | 0.000461 | Chr8 | 9341711 | 9344104 | 650 |
| novel.812 | -5.79231 | 1.97E-05 | 0.000461 | Chr1 | 34567490 | 34569932 | 2443 |
| novel.3486 | 2.169044 | 1.97E-05 | 0.000461 | Chr4 | 54852220 | 54853126 | 691 |
| MsG0580026903.01 | -2.74216 | 1.97E-05 | 0.000461 | Chr5 | 47801366 | 47801996 | 453 |
| MsG0380011639.01 | -2.42584 | 1.97E-05 | 0.000461 | Chr3 | 2916857 | 2921411 | 2576 |
| MsG0280010793.01 | -2.99923 | 1.97E-05 | 0.000461 | Chr2 | 75159641 | 75160730 | 971 |
| MsG0680030559.01 | -3.32427 | 1.98E-05 | 0.000461 | Chr6 | 5287814 | 5289199 | 1386 |
| MsG0180001183.01 | -2.05686 | 1.98E-05 | 0.000461 | Chr1 | 16945465 | 16950577 | 2115 |
| MsG0380014955.01 | -2.44096 | 1.99E-05 | 0.000464 | Chr3 | 63088639 | 63104118 | 6429 |
| novel.1870 | -1.79723 | 1.99E-05 | 0.000464 | Chr2 | 37295192 | 37305148 | 4465 |
| MsG0480019311.01 | -3.94006 | 2.01E-05 | 0.000467 | Chr4 | 18337178 | 18337462 | 285 |
| novel.7880 | 3.70118 | 2.01E-05 | 0.000467 | Chr8 | 16439374 | 16442373 | 2174 |
| MsG0880042454.01 | 7.003574 | 2.02E-05 | 0.000469 | Chr8 | 9085192 | 9085641 | 450 |
| MsG0680033985.01 | -4.69871 | 2.02E-05 | 0.000469 | Chr6 | 78105631 | 78114332 | 2430 |
| novel.2464 | 4.602158 | 2.03E-05 | 0.000469 | Chr3 | 72320697 | 72324222 | 2145 |
| MsG0180005688.01 | 6.772843 | 2.04E-05 | 0.000473 | Chr1 | 94934667 | 94936171 | 909 |
| MsG0880047020.01 | -7.13868 | 2.06E-05 | 0.000477 | Chr8 | 81111327 | 81114360 | 1071 |
| novel.7171 | 2.210644 | 2.06E-05 | 0.000477 | Chr7 | 78288993 | 78296131 | 3062 |
| MsG0480023322.01 | -1.75024 | 2.06E-05 | 0.000477 | Chr4 | 83716248 | 83718598 | 914 |
| novel.2026 | -3.08423 | 2.07E-05 | 0.000478 | Chr2 | 73123064 | 73125359 | 420 |
| MsG0780039681.01 | 5.042989 | 2.07E-05 | 0.000478 | Chr7 | 67491913 | 67492257 | 345 |
| MsG0680035300.01 | -5.47132 | 2.08E-05 | 0.00048 | Chr6 | 1.02E+08 | 1.02E+08 | 1032 |
| MsG0880042056.01 | 7.271689 | 2.10E-05 | 0.000484 | Chr8 | 3234965 | 3239383 | 858 |
| MsG0480020816.01 | 5.635477 | 2.11E-05 | 0.000485 | Chr4 | 48819910 | 48820137 | 228 |
| novel.9089 | 1.66808 | 2.11E-05 | 0.000486 | contig476end | 15725 | 19217 | 1545 |
| MsG0780040259.01 | -2.31086 | 2.12E-05 | 0.000487 | Chr7 | 75027930 | 75032934 | 1796 |
| MsG0880042411.01 | 2.38653 | 2.15E-05 | 0.000493 | Chr8 | 8367926 | 8371015 | 767 |
| novel.6997 | 6.595668 | 2.17E-05 | 0.000498 | Chr7 | 46430718 | 46446126 | 587 |
| novel.4060 | 5.715669 | 2.18E-05 | 0.000499 | Chr4 | 63961548 | 63962003 | 456 |
| MsG0680034772.01 | -4.13414 | 2.18E-05 | 0.000499 | Chr6 | 94277982 | 94278606 | 234 |
| novel.7045 | 6.865236 | 2.18E-05 | 0.000499 | Chr7 | 57842031 | 57844519 | 2377 |
| MsG0580028237.01 | -4.48089 | 2.19E-05 | 0.000501 | Chr5 | 77121063 | 77121833 | 771 |
| MsG0480023003.01 | -4.01451 | 2.19E-05 | 0.000501 | Chr4 | 79599672 | 79604891 | 3237 |
| MsG0480020445.01 | -3.11613 | 2.20E-05 | 0.000502 | Chr4 | 41618723 | 41625959 | 1056 |
| MsG0880044985.01 | 1.566942 | 2.21E-05 | 0.000504 | Chr8 | 52453094 | 52455480 | 720 |
| MsG0580025787.01 | 2.847453 | 2.21E-05 | 0.000504 | Chr5 | 24144084 | 24150279 | 375 |
| MsG0780037642.01 | 5.127646 | 2.22E-05 | 0.000506 | Chr7 | 29947213 | 29949363 | 792 |
| MsG0380015101.01 | 3.689244 | 2.23E-05 | 0.000508 | Chr3 | 65233289 | 65237966 | 1155 |
| MsG0080048210.01 | -3.47367 | 2.28E-05 | 0.000518 | contig244end | 3474 | 6230 | 1344 |
| MsG0580029430.01 | -2.30139 | 2.29E-05 | 0.000519 | Chr5 | 96898181 | 96902793 | 1628 |
| novel.6509 | -4.4232 | 2.29E-05 | 0.000519 | Chr6 | 64195757 | 64201762 | 646 |
| MsG0380016721.01 | -3.30292 | 2.29E-05 | 0.00052 | Chr3 | 87045770 | 87047691 | 1270 |
| novel.6984 | 6.612764 | 2.30E-05 | 0.000521 | Chr7 | 42168628 | 42169864 | 1091 |
| MsG0680032280.01 | -4.36735 | 2.30E-05 | 0.000521 | Chr6 | 37753640 | 37755238 | 1599 |
| novel.4821 | -4.91785 | 2.30E-05 | 0.000521 | Chr5 | 98132236 | 98151461 | 1592 |
| MsG0380011742.01 | -3.19097 | 2.31E-05 | 0.000522 | Chr3 | 4374577 | 4377905 | 1506 |
| MsG0180005434.01 | -3.09749 | 2.33E-05 | 0.000526 | Chr1 | 91838516 | 91840139 | 1054 |
| novel.2829 | 3.754894 | 2.34E-05 | 0.000529 | Chr3 | 31268453 | 31270032 | 798 |
| MsG0680035085.01 | 7.226765 | 2.36E-05 | 0.000533 | Chr6 | 98627665 | 98630265 | 1359 |
| MsG0380017336.01 | -3.39815 | 2.37E-05 | 0.000535 | Chr3 | 95082914 | 95086497 | 1990 |
| MsG0580029003.01 | -2.88946 | 2.38E-05 | 0.000535 | Chr5 | 90155213 | 90155848 | 435 |
| MsG0480018591.01 | -5.28498 | 2.38E-05 | 0.000535 | Chr4 | 7057238 | 7061041 | 828 |
| MsG0680030735.01 | 6.844139 | 2.39E-05 | 0.000538 | Chr6 | 8261254 | 8275946 | 741 |
| novel.5679 | 4.625665 | 2.40E-05 | 0.000539 | Chr6 | 27431444 | 27435077 | 2015 |
| MsG0480023116.01 | 4.316902 | 2.40E-05 | 0.000539 | Chr4 | 81093509 | 81100939 | 1506 |
| MsG0580027941.01 | -4.87576 | 2.41E-05 | 0.00054 | Chr5 | 71557416 | 71558198 | 783 |
| MsG0080048255.01 | 2.517977 | 2.41E-05 | 0.000541 | contig254end | 24561 | 26978 | 1035 |
| MsG0580029657.01 | 2.558259 | 2.42E-05 | 0.000542 | Chr5 | 1E+08 | 1E+08 | 1127 |
| MsG0180001391.01 | -3.8904 | 2.42E-05 | 0.000542 | Chr1 | 20394464 | 20398288 | 1420 |
| MsG0880046714.01 | -1.737 | 2.43E-05 | 0.000543 | Chr8 | 77255267 | 77255608 | 342 |
| MsG0480021646.01 | -2.64325 | 2.43E-05 | 0.000544 | Chr4 | 61231801 | 61235105 | 1506 |
| MsG0080048687.01 | -2.01261 | 2.45E-05 | 0.000548 | contig400end | 15444 | 19216 | 296 |
| MsG0180005648.01 | -2.81126 | 2.46E-05 | 0.000548 | Chr1 | 94452520 | 94453497 | 978 |
| novel.5274 | -5.03225 | 2.46E-05 | 0.000549 | Chr5 | 73553807 | 73555577 | 1276 |
| MsG0380018034.01 | 1.656449 | 2.47E-05 | 0.000549 | Chr3 | 1.04E+08 | 1.04E+08 | 2444 |
| novel.472 | 5.522056 | 2.47E-05 | 0.000549 | Chr1 | 85317053 | 85326933 | 775 |
| MsG0280010768.01 | 2.334025 | 2.47E-05 | 0.000549 | Chr2 | 74840293 | 74847070 | 1242 |
| novel.4210 | 6.68968 | 2.48E-05 | 0.000553 | Chr4 | 84647665 | 84648945 | 881 |
| novel.7646 | -2.1212 | 2.50E-05 | 0.000556 | Chr7 | 75260563 | 75262921 | 1443 |
| MsG0880042495.01 | -3.51732 | 2.50E-05 | 0.000556 | Chr8 | 9550288 | 9553701 | 989 |
| novel.5678 | -6.599 | 2.51E-05 | 0.000557 | Chr6 | 27277014 | 27279922 | 2909 |
| MsG0380017169.01 | 5.26107 | 2.52E-05 | 0.000559 | Chr3 | 92929697 | 92936180 | 1041 |
| novel.5740 | -2.74756 | 2.53E-05 | 0.00056 | Chr6 | 43109494 | 43113006 | 1208 |
| MsG0880041847.01 | 1.657617 | 2.53E-05 | 0.00056 | Chr8 | 173917 | 177434 | 1048 |
| novel.500 | 5.094779 | 2.54E-05 | 0.000561 | Chr1 | 89110462 | 89111636 | 441 |
| MsG0780039574.01 | -3.27662 | 2.56E-05 | 0.000566 | Chr7 | 66052102 | 66063194 | 1816 |
| novel.4912 | -6.15618 | 2.57E-05 | 0.000569 | Chr5 | 585360 | 593333 | 913 |
| novel.1516 | 2.011395 | 2.58E-05 | 0.00057 | Chr2 | 67201140 | 67204914 | 1910 |
| MsG0180003377.01 | 2.269313 | 2.59E-05 | 0.000572 | Chr1 | 61378730 | 61390901 | 3085 |
| MsG0880044007.01 | -3.64183 | 2.60E-05 | 0.000573 | Chr8 | 34247949 | 34251367 | 1945 |
| MsG0280009945.01 | -2.30975 | 2.60E-05 | 0.000573 | Chr2 | 61450484 | 61460514 | 1626 |
| MsG0180002746.01 | 6.650246 | 2.60E-05 | 0.000573 | Chr1 | 44353047 | 44357742 | 1296 |
| novel.3246 | 6.73382 | 2.60E-05 | 0.000573 | Chr4 | 5194766 | 5201255 | 1394 |
| MsG0480022223.01 | 1.756975 | 2.61E-05 | 0.000573 | Chr4 | 69839498 | 69841433 | 781 |
| MsG0880046412.01 | 1.635301 | 2.62E-05 | 0.000574 | Chr8 | 73113790 | 73116255 | 1648 |
| MsG0380016228.01 | 1.697598 | 2.62E-05 | 0.000574 | Chr3 | 80718209 | 80719191 | 899 |
| novel.6570 | 4.874562 | 2.62E-05 | 0.000575 | Chr6 | 75369826 | 75374268 | 2094 |
| novel.503 | 1.385963 | 2.63E-05 | 0.000577 | Chr1 | 90189442 | 90191249 | 1644 |
| novel.1330 | 4.392523 | 2.64E-05 | 0.000579 | Chr2 | 28042811 | 28045519 | 855 |
| MsG0280007916.01 | -3.04606 | 2.65E-05 | 0.000579 | Chr2 | 22878041 | 22878971 | 474 |
| novel.9198 | 6.752712 | 2.65E-05 | 0.00058 | contig541end | 12595 | 13701 | 1107 |
| novel.4465 | 6.939216 | 2.65E-05 | 0.00058 | Chr5 | 27848815 | 27851767 | 783 |
| MsG0480021200.01 | 1.616876 | 2.67E-05 | 0.000582 | Chr4 | 55083140 | 55086334 | 1131 |
| MsG0480022308.01 | -5.32317 | 2.67E-05 | 0.000583 | Chr4 | 70967429 | 70974018 | 3089 |
| novel.2563 | -6.68908 | 2.68E-05 | 0.000584 | Chr3 | 86407691 | 86409442 | 1404 |
| novel.7857 | -4.40393 | 2.68E-05 | 0.000584 | Chr8 | 11526550 | 11534127 | 4618 |
| MsG0780036703.01 | -2.10819 | 2.68E-05 | 0.000584 | Chr7 | 12166873 | 12168189 | 1317 |
| novel.57 | -3.6874 | 2.68E-05 | 0.000584 | Chr1 | 7411259 | 7414093 | 1527 |
| MsG0080048405.01 | -1.65746 | 2.69E-05 | 0.000585 | contig304end | 6567 | 10240 | 1126 |
| MsG0280006589.01 | -2.02671 | 2.69E-05 | 0.000585 | Chr2 | 3754567 | 3758504 | 2415 |
| MsG0480018607.01 | -2.17228 | 2.71E-05 | 0.000587 | Chr4 | 7301468 | 7306833 | 2122 |
| MsG0280008334.01 | -2.57821 | 2.71E-05 | 0.000588 | Chr2 | 29477755 | 29485130 | 3796 |
| novel.3235 | -3.81405 | 2.71E-05 | 0.000588 | Chr4 | 4175342 | 4180677 | 1889 |
| MsG0580024865.01 | 6.676992 | 2.71E-05 | 0.000588 | Chr5 | 10991080 | 10991577 | 498 |
| MsG0480021288.01 | 1.485198 | 2.73E-05 | 0.000591 | Chr4 | 56355288 | 56359907 | 1979 |
| MsG0480022125.01 | -4.21276 | 2.74E-05 | 0.000591 | Chr4 | 68566741 | 68567370 | 630 |
| MsG0380013178.01 | 1.974997 | 2.74E-05 | 0.000591 | Chr3 | 32133574 | 32139047 | 427 |
| MsG0780039336.01 | -2.58342 | 2.77E-05 | 0.000597 | Chr7 | 62183918 | 62185337 | 930 |
| novel.3302 | 3.354423 | 2.77E-05 | 0.000599 | Chr4 | 14141763 | 14143545 | 1370 |
| MsG0280008747.01 | -3.52732 | 2.78E-05 | 0.000599 | Chr2 | 37618333 | 37625190 | 3504 |
| novel.4685 | -6.69343 | 2.79E-05 | 0.000601 | Chr5 | 74774901 | 74780889 | 2048 |
| MsG0480022507.01 | -3.10385 | 2.79E-05 | 0.000601 | Chr4 | 73399142 | 73400728 | 1587 |
| novel.5162 | 6.969689 | 2.79E-05 | 0.000601 | Chr5 | 51571413 | 51573320 | 586 |
| MsG0380015637.01 | -2.0286 | 2.80E-05 | 0.000601 | Chr3 | 72860708 | 72864380 | 2079 |
| MsG0180006010.01 | -9.65002 | 2.80E-05 | 0.000601 | Chr1 | 98775756 | 98779191 | 1383 |
| novel.2308 | -6.68457 | 2.80E-05 | 0.000602 | Chr3 | 34701120 | 34706987 | 1206 |
| MsG0180002381.01 | -4.51199 | 2.81E-05 | 0.000603 | Chr1 | 37707923 | 37714656 | 2089 |
| novel.2896 | -6.58016 | 2.82E-05 | 0.000605 | Chr3 | 47911363 | 47911984 | 622 |
| MsG0380016773.01 | 3.683771 | 2.83E-05 | 0.000605 | Chr3 | 87768793 | 87771208 | 612 |
| novel.7970 | -6.67161 | 2.83E-05 | 0.000605 | Chr8 | 33760972 | 33761938 | 889 |
| MsG0480023383.01 | -3.72673 | 2.85E-05 | 0.00061 | Chr4 | 84485824 | 84486661 | 442 |
| novel.6730 | 4.089153 | 2.86E-05 | 0.000611 | Chr6 | 1.05E+08 | 1.05E+08 | 1477 |
| novel.1376 | -5.4906 | 2.86E-05 | 0.000612 | Chr2 | 35094271 | 35102805 | 1672 |
| novel.898 | 3.656273 | 2.88E-05 | 0.000615 | Chr1 | 58878040 | 58881024 | 1725 |
| MsG0380016627.01 | -2.29274 | 2.88E-05 | 0.000615 | Chr3 | 85864532 | 85865542 | 657 |
| MsG0280008066.01 | 6.400171 | 2.88E-05 | 0.000615 | Chr2 | 24980793 | 24984678 | 3687 |
| MsG0580030147.01 | -7.11011 | 2.89E-05 | 0.000615 | Chr5 | 1.07E+08 | 1.07E+08 | 273 |
| novel.3280 | 6.764155 | 2.90E-05 | 0.000617 | Chr4 | 10499132 | 10500494 | 1203 |
| MsG0580029712.01 | 3.172091 | 2.91E-05 | 0.000619 | Chr5 | 1.01E+08 | 1.01E+08 | 4148 |
| MsG0480022680.01 | -4.09504 | 2.93E-05 | 0.000622 | Chr4 | 75414343 | 75414888 | 546 |
| novel.294 | -6.57655 | 2.93E-05 | 0.000623 | Chr1 | 55247536 | 55251163 | 1625 |
| MsG0580024390.01 | 1.857469 | 2.94E-05 | 0.000623 | Chr5 | 4189805 | 4197475 | 1824 |
| MsG0480022878.01 | 2.920122 | 2.97E-05 | 0.000631 | Chr4 | 77965554 | 77972215 | 1947 |
| MsG0580027876.01 | -2.69524 | 2.98E-05 | 0.000631 | Chr5 | 70155303 | 70156253 | 951 |
| novel.3774 | -2.56431 | 2.99E-05 | 0.000633 | Chr4 | 3835004 | 3837763 | 2580 |
| novel.828 | 7.054987 | 3.00E-05 | 0.000636 | Chr1 | 37640352 | 37642873 | 978 |
| MsG0480021756.01 | -3.95096 | 3.05E-05 | 0.000645 | Chr4 | 63916129 | 63921127 | 1778 |
| MsG0580024549.01 | -2.04839 | 3.06E-05 | 0.000646 | Chr5 | 6273797 | 6274702 | 906 |
| novel.6492 | 6.618106 | 3.06E-05 | 0.000646 | Chr6 | 60265428 | 60268887 | 1301 |
| MsG0380011535.01 | -4.27639 | 3.06E-05 | 0.000646 | Chr3 | 1040491 | 1048461 | 4718 |
| novel.7310 | 1.88321 | 3.06E-05 | 0.000646 | Chr7 | 2111578 | 2114462 | 575 |
| novel.5779 | 2.706392 | 3.09E-05 | 0.000651 | Chr6 | 51149916 | 51152096 | 657 |
| novel.7925 | -1.96784 | 3.09E-05 | 0.000652 | Chr8 | 23352932 | 23355245 | 1399 |
| MsG0180000927.01 | 5.53468 | 3.11E-05 | 0.000655 | Chr1 | 13338244 | 13341290 | 2223 |
| MsG0780037924.01 | -6.20364 | 3.14E-05 | 0.000659 | Chr7 | 36449385 | 36455236 | 2892 |
| MsG0880046519.01 | 2.444212 | 3.14E-05 | 0.000661 | Chr8 | 74311079 | 74314925 | 1104 |
| MsG0480019313.01 | -1.96553 | 3.15E-05 | 0.000661 | Chr4 | 18350355 | 18353224 | 2680 |
| novel.5156 | 7.049562 | 3.16E-05 | 0.000663 | Chr5 | 50239632 | 50241856 | 1088 |
| MsG0380013414.01 | 5.043873 | 3.18E-05 | 0.000667 | Chr3 | 36903748 | 36905160 | 1413 |
| MsG0880043344.01 | -3.35571 | 3.21E-05 | 0.000672 | Chr8 | 23397498 | 23400056 | 1383 |
| MsG0380012614.01 | -6.46448 | 3.23E-05 | 0.000677 | Chr3 | 21086517 | 21093445 | 3600 |
| novel.8378 | -4.6446 | 3.26E-05 | 0.000683 | Chr8 | 15001106 | 15003603 | 1874 |
| MsG0680031472.01 | 2.645432 | 3.28E-05 | 0.000687 | Chr6 | 20912086 | 20919619 | 2553 |
| MsG0380012315.01 | 2.83361 | 3.29E-05 | 0.000688 | Chr3 | 14765491 | 14765817 | 327 |
| novel.5301 | -6.70441 | 3.33E-05 | 0.000695 | Chr5 | 78709230 | 78710089 | 860 |
| MsG0780037665.01 | -2.63702 | 3.35E-05 | 0.000698 | Chr7 | 30586586 | 30587863 | 279 |
| MsG0180000437.01 | -3.83099 | 3.35E-05 | 0.000698 | Chr1 | 6003828 | 6006044 | 1656 |
| novel.4329 | 2.31917 | 3.36E-05 | 0.000701 | Chr5 | 7465635 | 7470694 | 2896 |
| novel.1404 | 2.63415 | 3.38E-05 | 0.000704 | Chr2 | 39711469 | 39714660 | 1400 |
| MsG0680035568.01 | 2.599346 | 3.41E-05 | 0.000709 | Chr6 | 1.09E+08 | 1.09E+08 | 1296 |
| MsG0580025405.01 | -3.14253 | 3.41E-05 | 0.000709 | Chr5 | 18135260 | 18135718 | 459 |
| novel.232 | -6.63163 | 3.41E-05 | 0.000709 | Chr1 | 36357692 | 36359754 | 1550 |
| novel.8886 | -1.95903 | 3.41E-05 | 0.000709 | contig252end | 33970 | 41322 | 1530 |
| MsG0780038801.01 | 3.118339 | 3.43E-05 | 0.000711 | Chr7 | 53248333 | 53250068 | 1233 |
| MsG0880042591.01 | -3.56899 | 3.43E-05 | 0.000712 | Chr8 | 11092009 | 11095983 | 1992 |
| novel.5365 | 7.039196 | 3.44E-05 | 0.000713 | Chr5 | 89430347 | 89432580 | 1601 |
| novel.9344 | 3.110279 | 3.45E-05 | 0.000714 | contig635end | 5069 | 6775 | 1551 |
| novel.573 | 6.606883 | 3.48E-05 | 0.00072 | Chr1 | 1.02E+08 | 1.02E+08 | 598 |
| novel.3987 | -2.51619 | 3.49E-05 | 0.000722 | Chr4 | 52404468 | 52405934 | 1336 |
| MsG0380012044.01 | -3.29582 | 3.56E-05 | 0.000736 | Chr3 | 10115297 | 10120949 | 1179 |
| MsG0880042186.01 | 6.767763 | 3.58E-05 | 0.000739 | Chr8 | 5092976 | 5094434 | 396 |
| MsG0880047706.01 | 6.507777 | 3.59E-05 | 0.00074 | Chr8 | 89830159 | 89831785 | 1272 |
| novel.7222 | 6.104475 | 3.60E-05 | 0.000743 | Chr7 | 85308737 | 85315750 | 4555 |
| MsG0180004856.01 | -2.11805 | 3.64E-05 | 0.000749 | Chr1 | 83662425 | 83664957 | 1503 |
| novel.4427 | 6.528219 | 3.64E-05 | 0.00075 | Chr5 | 23717123 | 23718114 | 554 |
| MsG0580027688.01 | -6.73723 | 3.64E-05 | 0.00075 | Chr5 | 66160138 | 66161472 | 1335 |
| MsG0880047581.01 | 4.053226 | 3.65E-05 | 0.00075 | Chr8 | 88289886 | 88291841 | 1956 |
| MsG0180001740.01 | -4.02306 | 3.66E-05 | 0.00075 | Chr1 | 26275926 | 26280721 | 1644 |
| MsG0180002341.01 | -6.49429 | 3.66E-05 | 0.00075 | Chr1 | 37094846 | 37099927 | 2025 |
| novel.5120 | -6.65181 | 3.66E-05 | 0.00075 | Chr5 | 43374601 | 43378840 | 1169 |
| novel.7403 | -4.60002 | 3.66E-05 | 0.00075 | Chr7 | 21456799 | 21460544 | 3746 |
| novel.376 | 6.484606 | 3.69E-05 | 0.000757 | Chr1 | 68029606 | 68031882 | 620 |
| MsG0280007687.01 | -2.56803 | 3.72E-05 | 0.000761 | Chr2 | 19211559 | 19215755 | 1253 |
| MsG0780040943.01 | -2.80713 | 3.72E-05 | 0.000761 | Chr7 | 84613071 | 84616157 | 2556 |
| novel.445 | -3.55356 | 3.75E-05 | 0.000768 | Chr1 | 82168761 | 82172093 | 1627 |
| MsG0380012316.01 | 4.972528 | 3.77E-05 | 0.000771 | Chr3 | 14782086 | 14782313 | 228 |
| MsG0580024209.01 | -2.1354 | 3.78E-05 | 0.000771 | Chr5 | 2058681 | 2085629 | 6336 |
| MsG0580025210.01 | -1.76205 | 3.80E-05 | 0.000775 | Chr5 | 15360114 | 15361391 | 1278 |
| MsG0780041047.01 | -2.25249 | 3.80E-05 | 0.000776 | Chr7 | 85837987 | 85839768 | 1782 |
| MsG0880043450.01 | -1.99682 | 3.82E-05 | 0.000778 | Chr8 | 24982470 | 24988340 | 2956 |
| MsG0780037442.01 | 2.680578 | 3.82E-05 | 0.000778 | Chr7 | 26020911 | 26021204 | 294 |
| MsG0380013796.01 | -2.62319 | 3.82E-05 | 0.000778 | Chr3 | 45794187 | 45796490 | 2304 |
| MsG0680031814.01 | -2.39901 | 3.82E-05 | 0.000778 | Chr6 | 27186305 | 27193485 | 2084 |
| MsG0280010216.01 | -2.52699 | 3.83E-05 | 0.000779 | Chr2 | 66309125 | 66312039 | 2275 |
| MsG0380011545.01 | 5.37684 | 3.84E-05 | 0.00078 | Chr3 | 1225466 | 1226593 | 1128 |
| novel.4699 | -2.61141 | 3.85E-05 | 0.000781 | Chr5 | 77667438 | 77668789 | 1232 |
| novel.5153 | -3.53448 | 3.86E-05 | 0.000783 | Chr5 | 48341001 | 48341427 | 328 |
| MsG0680035494.01 | -2.50587 | 3.86E-05 | 0.000783 | Chr6 | 1.07E+08 | 1.07E+08 | 2468 |
| novel.7529 | -3.06175 | 3.89E-05 | 0.000787 | Chr7 | 55473869 | 55474616 | 631 |
| novel.736 | 5.608033 | 3.90E-05 | 0.00079 | Chr1 | 19594653 | 19595689 | 594 |
| MsG0180005996.01 | -4.05067 | 3.91E-05 | 0.000791 | Chr1 | 98610109 | 98611770 | 1662 |
| novel.1400 | 6.568714 | 3.92E-05 | 0.000793 | Chr2 | 38855675 | 38856562 | 427 |
| MsG0480018847.01 | -6.65966 | 3.93E-05 | 0.000793 | Chr4 | 10566015 | 10566620 | 606 |
| novel.2909 | -4.53655 | 3.94E-05 | 0.000795 | Chr3 | 50005163 | 50007033 | 500 |
| MsG0880045314.01 | -5.50608 | 3.95E-05 | 0.000798 | Chr8 | 57382053 | 57392002 | 1482 |
| novel.6248 | -4.33607 | 3.96E-05 | 0.000798 | Chr6 | 16262109 | 16263653 | 1545 |
| MsG0880043942.01 | 4.725348 | 4.02E-05 | 0.000809 | Chr8 | 33093803 | 33102407 | 2898 |
| novel.3927 | 7.193716 | 4.03E-05 | 0.000812 | Chr4 | 36545914 | 36547891 | 717 |
| novel.8087 | 2.278766 | 4.06E-05 | 0.000818 | Chr8 | 57505232 | 57505933 | 702 |
| MsG0680033129.01 | -3.9866 | 4.07E-05 | 0.000818 | Chr6 | 57539150 | 57560971 | 3505 |
| novel.6744 | 6.633382 | 4.12E-05 | 0.000828 | Chr6 | 1.08E+08 | 1.08E+08 | 4048 |
| novel.6124 | -2.64386 | 4.14E-05 | 0.000832 | Chr6 | 1.13E+08 | 1.13E+08 | 1147 |
| novel.1556 | 5.54575 | 4.16E-05 | 0.000833 | Chr2 | 75337539 | 75338639 | 499 |
| novel.7326 | -6.7266 | 4.16E-05 | 0.000833 | Chr7 | 8620998 | 8624093 | 672 |
| novel.6753 | -5.00458 | 4.16E-05 | 0.000833 | Chr6 | 1.08E+08 | 1.08E+08 | 1283 |
| MsG0480023134.01 | -9.45778 | 4.16E-05 | 0.000833 | Chr4 | 81236490 | 81239538 | 855 |
| MsG0680034980.01 | -4.97412 | 4.21E-05 | 0.000842 | Chr6 | 97297854 | 97298495 | 642 |
| novel.1821 | 6.926596 | 4.22E-05 | 0.000844 | Chr2 | 26717656 | 26719150 | 1244 |
| novel.4926 | -5.13501 | 4.23E-05 | 0.000846 | Chr5 | 3653602 | 3655225 | 1353 |
| novel.6732 | -6.46399 | 4.25E-05 | 0.000849 | Chr6 | 1.06E+08 | 1.06E+08 | 2063 |
| MsG0680032101.01 | -2.86948 | 4.27E-05 | 0.000853 | Chr6 | 33660972 | 33670438 | 3672 |
| MsG0780038923.01 | -4.257 | 4.29E-05 | 0.000855 | Chr7 | 55399250 | 55400531 | 681 |
| MsG0880042538.01 | -4.35047 | 4.32E-05 | 0.000862 | Chr8 | 10226241 | 10227369 | 837 |
| MsG0580024080.01 | -2.36849 | 4.37E-05 | 0.000871 | Chr5 | 566891 | 567520 | 630 |
| MsG0580027986.01 | -6.7519 | 4.39E-05 | 0.000873 | Chr5 | 72459178 | 72459561 | 384 |
| MsG0780040844.01 | 1.923528 | 4.40E-05 | 0.000874 | Chr7 | 83246979 | 83247290 | 312 |
| MsG0180005501.01 | -6.4795 | 4.42E-05 | 0.000879 | Chr1 | 92561616 | 92576667 | 5979 |
| MsG0780038467.01 | 6.43708 | 4.44E-05 | 0.000882 | Chr7 | 47187952 | 47190908 | 711 |
| MsG0180000832.01 | -2.15725 | 4.44E-05 | 0.000882 | Chr1 | 11838318 | 11852324 | 4036 |
| MsG0080048171.01 | -7.28377 | 4.45E-05 | 0.000882 | contig235end | 13115 | 14810 | 1094 |
| MsG0380016975.01 | 3.282853 | 4.45E-05 | 0.000883 | Chr3 | 90206261 | 90217285 | 1356 |
| novel.2343 | -2.81129 | 4.47E-05 | 0.000886 | Chr3 | 44670109 | 44670605 | 497 |
| MsG0180002212.01 | -4.72722 | 4.47E-05 | 0.000886 | Chr1 | 34942365 | 34945124 | 2760 |
| novel.452 | 2.318846 | 4.48E-05 | 0.000887 | Chr1 | 82662704 | 82665566 | 1611 |
| novel.6030 | 6.47738 | 4.50E-05 | 0.000889 | Chr6 | 1.01E+08 | 1.01E+08 | 521 |
| MsG0180003116.01 | 2.801517 | 4.57E-05 | 0.000903 | Chr1 | 56848800 | 56852050 | 780 |
| MsG0280006747.01 | 1.642125 | 4.61E-05 | 0.000911 | Chr2 | 5780629 | 5781864 | 916 |
| MsG0380012122.01 | 2.950871 | 4.62E-05 | 0.000912 | Chr3 | 11165232 | 11171181 | 670 |
| MsG0280009968.01 | -2.14177 | 4.63E-05 | 0.000913 | Chr2 | 62018555 | 62022834 | 1479 |
| MsG0780040427.01 | -2.72754 | 4.66E-05 | 0.000917 | Chr7 | 77387086 | 77389847 | 969 |
| MsG0380016033.01 | -2.60075 | 4.73E-05 | 0.000932 | Chr3 | 78168448 | 78170991 | 2474 |
| MsG0080048295.01 | 6.492395 | 4.74E-05 | 0.000934 | contig266end | 34772 | 41182 | 1644 |
| novel.6998 | 2.033852 | 4.85E-05 | 0.000953 | Chr7 | 46456042 | 46461832 | 3607 |
| novel.945 | 6.410139 | 4.89E-05 | 0.000962 | Chr1 | 67201020 | 67206892 | 762 |
| novel.7897 | -2.38006 | 4.92E-05 | 0.000966 | Chr8 | 19408025 | 19410771 | 1346 |
| MsG0180003252.01 | -3.41991 | 4.92E-05 | 0.000966 | Chr1 | 59136635 | 59137510 | 876 |
| MsG0880046262.01 | 6.627149 | 4.93E-05 | 0.000968 | Chr8 | 71246696 | 71249031 | 621 |
| MsG0680030899.01 | -1.76612 | 4.96E-05 | 0.000972 | Chr6 | 11287091 | 11288843 | 1317 |
| novel.2074 | 5.422295 | 4.98E-05 | 0.000976 | Chr2 | 79852140 | 79852875 | 649 |
| MsG0880043539.01 | -3.00664 | 4.98E-05 | 0.000976 | Chr8 | 26290576 | 26302048 | 3000 |
| MsG0780036239.01 | 2.925252 | 5.01E-05 | 0.00098 | Chr7 | 5079303 | 5087622 | 1229 |
| MsG0280010511.01 | -2.84053 | 5.03E-05 | 0.000983 | Chr2 | 70773770 | 70779538 | 1362 |
| novel.3840 | -6.67718 | 5.04E-05 | 0.000984 | Chr4 | 17823477 | 17824599 | 1123 |
| MsG0480021787.01 | -3.44186 | 5.05E-05 | 0.000986 | Chr4 | 64261891 | 64262322 | 432 |
| MsG0280009252.01 | 6.602253 | 5.06E-05 | 0.000987 | Chr2 | 48722401 | 48724144 | 1038 |
| MsG0380014690.01 | -6.84339 | 5.07E-05 | 0.000988 | Chr3 | 58533871 | 58542064 | 1353 |
| novel.274 | -3.40559 | 5.08E-05 | 0.000989 | Chr1 | 46490678 | 46493337 | 555 |
| MsG0580026183.01 | -2.81353 | 5.16E-05 | 0.001005 | Chr5 | 31952271 | 31952960 | 690 |
| novel.1841 | -3.34548 | 5.21E-05 | 0.001014 | Chr2 | 31663423 | 31667553 | 2061 |
| novel.6787 | 4.177199 | 5.22E-05 | 0.001016 | Chr6 | 1.14E+08 | 1.14E+08 | 952 |
| MsG0480020487.01 | -2.22168 | 5.28E-05 | 0.001027 | Chr4 | 42330886 | 42331683 | 798 |
| novel.3083 | 4.188961 | 5.35E-05 | 0.00104 | Chr3 | 84287751 | 84290299 | 1179 |
| novel.6865 | 6.460865 | 5.36E-05 | 0.00104 | Chr7 | 17970934 | 17972427 | 1275 |
| novel.5010 | -6.55968 | 5.38E-05 | 0.001044 | Chr5 | 17488418 | 17490797 | 1232 |
| MsG0880043087.01 | 2.909241 | 5.43E-05 | 0.001053 | Chr8 | 18801655 | 18804984 | 3195 |
| MsG0180001123.01 | -2.89835 | 5.46E-05 | 0.001057 | Chr1 | 16303573 | 16315112 | 3324 |
| MsG0180000192.01 | 5.962117 | 5.50E-05 | 0.001064 | Chr1 | 2656375 | 2660807 | 1783 |
| novel.1238 | -2.24032 | 5.51E-05 | 0.001066 | Chr2 | 12472459 | 12473512 | 1054 |
| novel.6891 | -6.5056 | 5.56E-05 | 0.001075 | Chr7 | 21410677 | 21412864 | 694 |
| MsG0480019007.01 | 1.243794 | 5.63E-05 | 0.001088 | Chr4 | 13230791 | 13235726 | 1079 |
| MsG0480023113.01 | 4.109755 | 5.65E-05 | 0.001092 | Chr4 | 81087409 | 81088287 | 879 |
| MsG0480022910.01 | 2.056179 | 5.67E-05 | 0.001095 | Chr4 | 78428793 | 78433544 | 452 |
| novel.3843 | -3.94104 | 5.69E-05 | 0.001097 | Chr4 | 18253806 | 18261282 | 1035 |
| MsG0780040188.01 | -1.87417 | 5.70E-05 | 0.001099 | Chr7 | 74112725 | 74120178 | 3402 |
| novel.5299 | 6.882674 | 5.74E-05 | 0.001106 | Chr5 | 77415524 | 77417104 | 737 |
| novel.1256 | -4.9271 | 5.77E-05 | 0.001111 | Chr2 | 14878627 | 14880968 | 525 |
| MsG0880046072.01 | -2.05885 | 5.84E-05 | 0.001123 | Chr8 | 68631572 | 68644742 | 3161 |
| MsG0180001492.01 | -2.57551 | 5.89E-05 | 0.001132 | Chr1 | 22197079 | 22206836 | 1493 |
| novel.6515 | -6.47163 | 5.92E-05 | 0.001137 | Chr6 | 65692534 | 65694995 | 1243 |
| MsG0580024219.01 | -3.75095 | 5.94E-05 | 0.001141 | Chr5 | 2157874 | 2158635 | 762 |
| MsG0480021499.01 | -1.73812 | 5.98E-05 | 0.001145 | Chr4 | 58625965 | 58636840 | 1656 |
| MsG0580025969.01 | -1.85507 | 5.98E-05 | 0.001145 | Chr5 | 28340242 | 28345109 | 1932 |
| novel.6221 | 3.00028 | 5.98E-05 | 0.001145 | Chr6 | 12439893 | 12447945 | 3380 |
| novel.462 | 5.256686 | 5.99E-05 | 0.001146 | Chr1 | 84886072 | 84886915 | 695 |
| MsG0680035014.01 | -2.9927 | 6.02E-05 | 0.001151 | Chr6 | 97766021 | 97768828 | 759 |
| MsG0180000810.01 | 6.827868 | 6.07E-05 | 0.00116 | Chr1 | 11510604 | 11511764 | 1161 |
| MsG0280007959.01 | 2.466398 | 6.07E-05 | 0.001161 | Chr2 | 23729905 | 23739405 | 891 |
| MsG0380015496.01 | 1.37972 | 6.08E-05 | 0.001161 | Chr3 | 70993119 | 70995276 | 1236 |
| MsG0580029773.01 | 3.923647 | 6.11E-05 | 0.001167 | Chr5 | 1.02E+08 | 1.02E+08 | 783 |
| novel.7982 | 1.619485 | 6.15E-05 | 0.001174 | Chr8 | 36700592 | 36721790 | 3158 |
| MsG0080048211.01 | 1.967469 | 6.18E-05 | 0.001177 | contig244end | 26452 | 29658 | 825 |
| novel.6462 | 1.561056 | 6.18E-05 | 0.001177 | Chr6 | 52962593 | 52964583 | 1907 |
| MsG0180004291.01 | 1.520642 | 6.19E-05 | 0.001178 | Chr1 | 76160936 | 76166898 | 1303 |
| MsG0280010016.01 | 1.42186 | 6.19E-05 | 0.001178 | Chr2 | 62859263 | 62864922 | 1561 |
| MsG0180005913.01 | -1.69775 | 6.22E-05 | 0.001183 | Chr1 | 97470099 | 97473214 | 1409 |
| novel.1398 | -4.07472 | 6.26E-05 | 0.00119 | Chr2 | 38475411 | 38477000 | 1307 |
| novel.1910 | -2.79053 | 6.29E-05 | 0.001194 | Chr2 | 51143325 | 51147873 | 1925 |
| MsG0580024450.01 | -2.73537 | 6.34E-05 | 0.001202 | Chr5 | 5088821 | 5095077 | 2766 |
| MsG0880043898.01 | -2.13257 | 6.35E-05 | 0.001204 | Chr8 | 32326612 | 32329906 | 888 |
| novel.3817 | -2.57813 | 6.37E-05 | 0.001206 | Chr4 | 13730248 | 13733524 | 2959 |
| novel.6164 | -1.68543 | 6.37E-05 | 0.001207 | Chr6 | 4983235 | 4991427 | 4108 |
| MsG0480019552.01 | -1.51933 | 6.38E-05 | 0.001207 | Chr4 | 22026880 | 22032158 | 1186 |
| MsG0880042072.01 | -2.45116 | 6.39E-05 | 0.001209 | Chr8 | 3381162 | 3389171 | 3291 |
| MsG0380016317.01 | 1.420705 | 6.41E-05 | 0.001211 | Chr3 | 81918119 | 81919183 | 621 |
| novel.5117 | -3.50902 | 6.48E-05 | 0.001225 | Chr5 | 43232640 | 43236602 | 1411 |
| novel.6743 | -2.34048 | 6.51E-05 | 0.001228 | Chr6 | 1.07E+08 | 1.07E+08 | 2260 |
| MsG0480023107.01 | -3.41396 | 6.51E-05 | 0.001228 | Chr4 | 81045041 | 81049568 | 823 |
| MsG0780040061.01 | 1.327782 | 6.56E-05 | 0.001236 | Chr7 | 72566185 | 72567909 | 951 |
| MsG0080048203.01 | -3.13103 | 6.59E-05 | 0.001242 | contig240end | 280877 | 286019 | 2625 |
| novel.877 | 6.629585 | 6.60E-05 | 0.001242 | Chr1 | 53650359 | 53651725 | 833 |
| MsG0880042633.01 | 4.336484 | 6.62E-05 | 0.001246 | Chr8 | 11737824 | 11742869 | 2897 |
| novel.2907 | 6.466444 | 6.64E-05 | 0.001249 | Chr3 | 49428223 | 49433779 | 661 |
| novel.5095 | 4.048618 | 6.69E-05 | 0.001258 | Chr5 | 36553857 | 36586782 | 1903 |
| MsG0680035565.01 | 5.282697 | 6.72E-05 | 0.001263 | Chr6 | 1.09E+08 | 1.09E+08 | 591 |
| MsG0880045859.01 | 3.230418 | 6.74E-05 | 0.001265 | Chr8 | 65550802 | 65551182 | 381 |
| MsG0880045489.01 | -3.34757 | 6.82E-05 | 0.001279 | Chr8 | 59961279 | 59976265 | 1854 |
| novel.6250 | 4.363369 | 6.87E-05 | 0.001286 | Chr6 | 16320193 | 16321194 | 389 |
| MsG0480019038.01 | -2.47363 | 6.87E-05 | 0.001286 | Chr4 | 13641687 | 13647286 | 720 |
| novel.4637 | 6.377702 | 6.87E-05 | 0.001286 | Chr5 | 65378475 | 65383510 | 1143 |
| MsG0480020817.01 | 5.359371 | 6.90E-05 | 0.00129 | Chr4 | 48822115 | 48822366 | 252 |
| MsG0480020718.01 | 3.855345 | 6.95E-05 | 0.001299 | Chr4 | 46927349 | 46936064 | 1018 |
| MsG0580029683.01 | 6.622113 | 6.95E-05 | 0.001299 | Chr5 | 1.01E+08 | 1.01E+08 | 498 |
| MsG0380012800.01 | -2.09909 | 6.97E-05 | 0.001302 | Chr3 | 24776023 | 24776346 | 324 |
| MsG0380017593.01 | 5.919967 | 6.99E-05 | 0.001304 | Chr3 | 98264408 | 98267208 | 409 |
| MsG0480023306.01 | -1.71921 | 7.00E-05 | 0.001305 | Chr4 | 83567963 | 83576525 | 4005 |
| MsG0880042503.01 | -1.35833 | 7.00E-05 | 0.001305 | Chr8 | 9675863 | 9680163 | 564 |
| MsG0380016101.01 | 7.266229 | 7.01E-05 | 0.001306 | Chr3 | 79140275 | 79146494 | 4567 |
| MsG0780040806.01 | -6.62915 | 7.02E-05 | 0.001306 | Chr7 | 82728240 | 82735525 | 3771 |
| novel.5414 | 6.357382 | 7.02E-05 | 0.001307 | Chr5 | 97588481 | 97589873 | 1181 |
| MsG0680034470.01 | 2.226264 | 7.04E-05 | 0.001308 | Chr6 | 88060512 | 88071878 | 1812 |
| novel.4123 | -6.36667 | 7.05E-05 | 0.001311 | Chr4 | 71356789 | 71357500 | 558 |
| novel.6670 | -6.45231 | 7.09E-05 | 0.001317 | Chr6 | 98796160 | 98797088 | 840 |
| MsG0180000456.01 | 2.033851 | 7.10E-05 | 0.001318 | Chr1 | 6328274 | 6328447 | 174 |
| MsG0580028603.01 | -2.47506 | 7.18E-05 | 0.001332 | Chr5 | 83201388 | 83205546 | 1823 |
| novel.8530 | 3.192459 | 7.22E-05 | 0.001339 | Chr8 | 48000752 | 48002424 | 1045 |
| MsG0880044192.01 | -6.85038 | 7.26E-05 | 0.001344 | Chr8 | 37344613 | 37345338 | 726 |
| novel.1678 | -1.94243 | 7.26E-05 | 0.001345 | Chr2 | 4066170 | 4087275 | 2817 |
| MsG0680033006.01 | 2.76851 | 7.27E-05 | 0.001345 | Chr6 | 54639218 | 54640337 | 477 |
| MsG0480018222.01 | -3.12216 | 7.31E-05 | 0.00135 | Chr4 | 1769277 | 1769660 | 384 |
| MsG0880047150.01 | 1.38617 | 7.31E-05 | 0.00135 | Chr8 | 82701114 | 82702711 | 1315 |
| MsG0580026426.01 | -5.24596 | 7.31E-05 | 0.00135 | Chr5 | 37069202 | 37069895 | 369 |
| MsG0180000887.01 | -3.80876 | 7.32E-05 | 0.00135 | Chr1 | 12774880 | 12779079 | 1440 |
| novel.921 | 7.112095 | 7.46E-05 | 0.001376 | Chr1 | 63742036 | 63745499 | 2721 |
| MsG0480021614.01 | 5.937051 | 7.47E-05 | 0.001377 | Chr4 | 60751822 | 60755276 | 1727 |
| novel.4237 | 2.363039 | 7.48E-05 | 0.001377 | Chr4 | 87860525 | 87866035 | 1549 |
| MsG0580028432.01 | -4.00517 | 7.48E-05 | 0.001377 | Chr5 | 80437588 | 80443048 | 1557 |
| MsG0680032100.01 | -2.8733 | 7.52E-05 | 0.001384 | Chr6 | 33649518 | 33652838 | 3042 |
| novel.3375 | -6.85181 | 7.54E-05 | 0.001386 | Chr4 | 26706003 | 26707821 | 926 |
| MsG0780036582.01 | -1.8252 | 7.56E-05 | 0.001389 | Chr7 | 10084458 | 10086287 | 1069 |
| MsG0380015515.01 | -3.62447 | 7.57E-05 | 0.00139 | Chr3 | 71284973 | 71287467 | 1074 |
| MsG0780040313.01 | -1.47311 | 7.58E-05 | 0.00139 | Chr7 | 75810709 | 75824310 | 4272 |
| MsG0280009824.01 | -3.65748 | 7.61E-05 | 0.001397 | Chr2 | 58939289 | 58941068 | 1065 |
| MsG0780037414.01 | -2.71436 | 7.63E-05 | 0.001399 | Chr7 | 25399657 | 25401254 | 1131 |
| MsG0380015111.01 | 1.640702 | 7.66E-05 | 0.001402 | Chr3 | 65372227 | 65377923 | 1547 |
| novel.6587 | 6.720536 | 7.69E-05 | 0.001408 | Chr6 | 80871518 | 80873627 | 919 |
| MsG0280006414.01 | -7.14003 | 7.70E-05 | 0.001408 | Chr2 | 1650395 | 1653400 | 918 |
| novel.2751 | -3.92554 | 7.70E-05 | 0.001408 | Chr3 | 11741822 | 11743572 | 1000 |
| novel.809 | 2.349763 | 7.74E-05 | 0.001414 | Chr1 | 33291256 | 33295648 | 2720 |
| novel.1561 | -6.36072 | 7.77E-05 | 0.001419 | Chr2 | 76470845 | 76471423 | 579 |
| MsG0280006520.01 | 1.319408 | 7.78E-05 | 0.00142 | Chr2 | 3039847 | 3044359 | 2531 |
| novel.7957 | -2.79055 | 7.79E-05 | 0.001421 | Chr8 | 30394432 | 30395536 | 1105 |
| novel.1286 | -3.64499 | 7.80E-05 | 0.001422 | Chr2 | 19350004 | 19352816 | 2732 |
| MsG0580025338.01 | -2.46097 | 7.81E-05 | 0.001423 | Chr5 | 17146533 | 17149159 | 996 |
| novel.1647 | -3.57473 | 7.83E-05 | 0.001426 | Chr2 | 1203533 | 1205351 | 522 |
| MsG0180002184.01 | 1.64017 | 7.84E-05 | 0.001427 | Chr1 | 34394603 | 34405280 | 1700 |
| MsG0680031550.01 | -3.37156 | 7.86E-05 | 0.001429 | Chr6 | 22133780 | 22142588 | 900 |
| MsG0580024320.01 | -1.99819 | 7.91E-05 | 0.001438 | Chr5 | 3340511 | 3343973 | 1278 |
| MsG0280009666.01 | 1.5022 | 7.96E-05 | 0.001445 | Chr2 | 55952348 | 55956289 | 2932 |
| novel.3040 | -2.94013 | 7.99E-05 | 0.00145 | Chr3 | 78340097 | 78341445 | 1269 |
| MsG0680032473.01 | -3.04787 | 8.01E-05 | 0.001453 | Chr6 | 42258781 | 42279077 | 2892 |
| MsG0180002504.01 | 2.240554 | 8.02E-05 | 0.001454 | Chr1 | 39338996 | 39341416 | 934 |
| MsG0280006468.01 | 4.037078 | 8.10E-05 | 0.001467 | Chr2 | 2353632 | 2357963 | 759 |
| novel.4655 | 3.679772 | 8.13E-05 | 0.001472 | Chr5 | 70478180 | 70486288 | 1438 |
| novel.6220 | -4.22457 | 8.17E-05 | 0.001478 | Chr6 | 12378565 | 12379964 | 1400 |
| MsG0880044416.01 | 3.333229 | 8.21E-05 | 0.001484 | Chr8 | 41719605 | 41721451 | 293 |
| MsG0780040563.01 | -2.02187 | 8.24E-05 | 0.001489 | Chr7 | 79228622 | 79229647 | 1026 |
| novel.1099 | -6.52797 | 8.24E-05 | 0.001489 | Chr1 | 94084922 | 94086926 | 2005 |
| MsG0880045538.01 | -2.99233 | 8.26E-05 | 0.00149 | Chr8 | 60717421 | 60722415 | 3195 |
| MsG0180005235.01 | 1.458725 | 8.26E-05 | 0.00149 | Chr1 | 88927110 | 88928553 | 1347 |
| MsG0580025547.01 | 5.152715 | 8.27E-05 | 0.001492 | Chr5 | 20319091 | 20319582 | 492 |
| MsG0880043915.01 | 5.837836 | 8.31E-05 | 0.001497 | Chr8 | 32493105 | 32494892 | 957 |
| MsG0780036060.01 | -2.44041 | 8.32E-05 | 0.001498 | Chr7 | 1952822 | 1954450 | 1354 |
| MsG0780040746.01 | -6.61391 | 8.32E-05 | 0.001498 | Chr7 | 81873109 | 81882728 | 5097 |
| MsG0280008290.01 | 3.086632 | 8.34E-05 | 0.001501 | Chr2 | 28728368 | 28731507 | 1612 |
| MsG0580028538.01 | -3.04919 | 8.39E-05 | 0.001508 | Chr5 | 81995178 | 81996020 | 843 |
| MsG0480023073.01 | 2.212583 | 8.45E-05 | 0.001519 | Chr4 | 80437465 | 80440048 | 1443 |
| MsG0680032019.01 | -1.49964 | 8.58E-05 | 0.00154 | Chr6 | 32060938 | 32062204 | 938 |
| novel.7027 | 5.593705 | 8.58E-05 | 0.00154 | Chr7 | 52193803 | 52194829 | 814 |
| MsG0880045119.01 | 1.2491 | 8.61E-05 | 0.001545 | Chr8 | 54478580 | 54480991 | 874 |
| MsG0080048507.01 | -2.29938 | 8.87E-05 | 0.00159 | contig339end | 14633 | 18450 | 3324 |
| MsG0580025867.01 | 1.934027 | 8.92E-05 | 0.001597 | Chr5 | 25483504 | 25494630 | 1236 |
| novel.6133 | 6.577716 | 8.94E-05 | 0.0016 | Chr6 | 1.14E+08 | 1.14E+08 | 653 |
| MsG0780036533.01 | -6.38366 | 8.99E-05 | 0.001607 | Chr7 | 9424037 | 9428739 | 1659 |
| novel.8994 | -2.33977 | 8.99E-05 | 0.001607 | contig412end | 9847 | 11171 | 861 |
| MsG0880045409.01 | -6.60419 | 9.01E-05 | 0.001609 | Chr8 | 58853318 | 58866619 | 1225 |
| MsG0380016546.01 | -2.70295 | 9.02E-05 | 0.001611 | Chr3 | 84993188 | 84993878 | 363 |
| MsG0180005421.01 | 3.052902 | 9.14E-05 | 0.001632 | Chr1 | 91645857 | 91646843 | 840 |
| MsG0880042713.01 | -3.30172 | 9.18E-05 | 0.001637 | Chr8 | 13031150 | 13035312 | 3228 |
| novel.4128 | -4.04138 | 9.27E-05 | 0.001652 | Chr4 | 73439501 | 73441798 | 2298 |
| novel.6705 | -3.39832 | 9.31E-05 | 0.001657 | Chr6 | 1.03E+08 | 1.03E+08 | 1426 |
| novel.6298 | -4.12084 | 9.31E-05 | 0.001657 | Chr6 | 22278292 | 22282449 | 2155 |
| novel.2803 | 5.296095 | 9.37E-05 | 0.001666 | Chr3 | 24028016 | 24030506 | 1685 |
| MsG0680031654.01 | 1.413879 | 9.44E-05 | 0.001678 | Chr6 | 24024693 | 24039396 | 2424 |
| MsG0380016486.01 | 5.788048 | 9.45E-05 | 0.001679 | Chr3 | 84106558 | 84108241 | 1044 |
| MsG0380012654.01 | -6.47489 | 9.60E-05 | 0.001704 | Chr3 | 21954456 | 21962319 | 1434 |
| MsG0480020428.01 | -5.26018 | 9.64E-05 | 0.00171 | Chr4 | 41168040 | 41169281 | 1242 |
| MsG0180005391.01 | 2.442362 | 9.65E-05 | 0.001711 | Chr1 | 91215404 | 91217666 | 1324 |
| MsG0580025323.01 | 3.120887 | 9.67E-05 | 0.001715 | Chr5 | 16939463 | 16939720 | 258 |
| novel.249 | 2.740296 | 9.76E-05 | 0.001729 | Chr1 | 38948131 | 38949829 | 1527 |
| MsG0880044518.01 | 6.538728 | 9.79E-05 | 0.001733 | Chr8 | 43614130 | 43618587 | 960 |
| MsG0680034720.01 | -3.20716 | 9.87E-05 | 0.001747 | Chr6 | 93403207 | 93407231 | 3411 |
| novel.678 | -1.84183 | 9.88E-05 | 0.001747 | Chr1 | 10240053 | 10243249 | 1218 |
| MsG0280006411.01 | -1.72278 | 9.88E-05 | 0.001747 | Chr2 | 1586225 | 1587213 | 540 |
| MsG0180003035.01 | 6.220847 | 9.91E-05 | 0.00175 | Chr1 | 55364452 | 55365865 | 405 |
| novel.7486 | 6.709788 | 9.93E-05 | 0.001752 | Chr7 | 44204164 | 44206465 | 531 |
| MsG0680035514.01 | -1.52485 | 9.94E-05 | 0.001754 | Chr6 | 1.07E+08 | 1.07E+08 | 1869 |
| MsG0380012834.01 | 2.986862 | 9.98E-05 | 0.00176 | Chr3 | 25302936 | 25306660 | 3540 |
| novel.3019 | 5.995448 | 9.99E-05 | 0.001761 | Chr3 | 74645346 | 74645864 | 391 |
| novel.8128 | -2.50482 | 0.0001 | 0.001764 | Chr8 | 62919951 | 62922763 | 2732 |
| MsG0380012412.01 | -7.13605 | 0.0001 | 0.001766 | Chr3 | 16079360 | 16080445 | 1086 |
| MsG0280006815.01 | 1.768732 | 0.000101 | 0.001769 | Chr2 | 6638875 | 6640481 | 987 |
| novel.1187 | -2.32837 | 0.000101 | 0.001769 | Chr2 | 3391242 | 3391553 | 312 |
| MsG0480021617.01 | -6.50695 | 0.000101 | 0.001774 | Chr4 | 60832723 | 60835509 | 2787 |
| MsG0480021550.01 | 5.936644 | 0.000101 | 0.001774 | Chr4 | 59520663 | 59522800 | 1733 |
| MsG0380016255.01 | -1.97863 | 0.000101 | 0.001774 | Chr3 | 81094170 | 81104157 | 2142 |
| novel.4505 | -4.36656 | 0.000102 | 0.001795 | Chr5 | 36554421 | 36563100 | 1790 |
| novel.2957 | -4.8145 | 0.000103 | 0.001801 | Chr3 | 61711260 | 61712134 | 875 |
| novel.4152 | 5.835682 | 0.000103 | 0.00181 | Chr4 | 78013450 | 78016896 | 1778 |
| novel.3857 | -6.47639 | 0.000103 | 0.001812 | Chr4 | 20877432 | 20885723 | 371 |
| MsG0880042368.01 | -1.46715 | 0.000104 | 0.001812 | Chr8 | 7678145 | 7689624 | 4892 |
| MsG0780038542.01 | 2.177318 | 0.000104 | 0.001814 | Chr7 | 48428455 | 48466752 | 13218 |
| novel.1878 | -5.78236 | 0.000104 | 0.001816 | Chr2 | 38682872 | 38684232 | 797 |
| MsG0280009462.01 | -2.44073 | 0.000105 | 0.00183 | Chr2 | 52387645 | 52393497 | 1485 |
| novel.848 | 3.677238 | 0.000105 | 0.001832 | Chr1 | 43723536 | 43727425 | 1033 |
| MsG0880044972.01 | -3.01272 | 0.000105 | 0.001834 | Chr8 | 52283286 | 52284380 | 1095 |
| MsG0880047773.01 | 5.292838 | 0.000105 | 0.001837 | Chr8 | 90642126 | 90644079 | 1504 |
| MsG0180003971.01 | 2.063953 | 0.000105 | 0.001837 | Chr1 | 71189220 | 71196018 | 1371 |
| MsG0480022307.01 | 1.756212 | 0.000106 | 0.001846 | Chr4 | 70966682 | 70967114 | 433 |
| novel.6338 | -7.10138 | 0.000106 | 0.001847 | Chr6 | 27442206 | 27442977 | 772 |
| MsG0280006704.01 | -2.99345 | 0.000107 | 0.001855 | Chr2 | 5180013 | 5182037 | 969 |
| MsG0080049061.01 | 1.733318 | 0.000107 | 0.001859 | contig641end | 10494 | 14971 | 802 |
| MsG0880042856.01 | -2.35134 | 0.000107 | 0.001859 | Chr8 | 14938244 | 14955285 | 3239 |
| MsG0280008805.01 | -2.98715 | 0.000107 | 0.001859 | Chr2 | 38998260 | 39004444 | 3147 |
| MsG0280007545.01 | 2.005134 | 0.000107 | 0.001865 | Chr2 | 16970232 | 16973048 | 1176 |
| novel.2415 | -6.3952 | 0.000108 | 0.001866 | Chr3 | 62707716 | 62709051 | 762 |
| MsG0680032165.01 | 2.429445 | 0.000109 | 0.001894 | Chr6 | 35414233 | 35441967 | 3332 |
| novel.3267 | -2.54875 | 0.00011 | 0.001898 | Chr4 | 7123948 | 7125428 | 1185 |
| MsG0780036754.01 | -2.0612 | 0.00011 | 0.001898 | Chr7 | 13226065 | 13228784 | 1132 |
| MsG0380013860.01 | 1.893857 | 0.00011 | 0.001898 | Chr3 | 46775577 | 46781526 | 1715 |
| MsG0580025594.01 | 6.423147 | 0.000111 | 0.001913 | Chr5 | 20995008 | 21001316 | 2349 |
| MsG0680035646.01 | 1.876605 | 0.000111 | 0.001919 | Chr6 | 1.1E+08 | 1.1E+08 | 570 |
| MsG0480021958.01 | -2.67791 | 0.000111 | 0.001919 | Chr4 | 66417075 | 66427077 | 1552 |
| MsG0580028434.01 | -2.14776 | 0.000112 | 0.001928 | Chr5 | 80468017 | 80470134 | 2118 |
| MsG0080048302.01 | -4.05449 | 0.000112 | 0.00194 | contig26end | 27194 | 27874 | 681 |
| novel.2029 | 4.706102 | 0.000113 | 0.00194 | Chr2 | 73639151 | 73643860 | 2410 |
| MsG0480019403.01 | 6.214892 | 0.000113 | 0.00194 | Chr4 | 19686033 | 19686675 | 425 |
| MsG0480023343.01 | 3.709569 | 0.000113 | 0.001952 | Chr4 | 84026367 | 84030241 | 1053 |
| novel.6475 | 6.308299 | 0.000114 | 0.001959 | Chr6 | 55710322 | 55718765 | 641 |
| MsG0780039243.01 | 1.726585 | 0.000114 | 0.001959 | Chr7 | 60561759 | 60563122 | 675 |
| MsG0480018521.01 | -3.97782 | 0.000115 | 0.001975 | Chr4 | 6096921 | 6097496 | 576 |
| novel.7899 | 7.062029 | 0.000115 | 0.001975 | Chr8 | 19473047 | 19475772 | 1289 |
| MsG0480022736.01 | -2.61602 | 0.000115 | 0.001976 | Chr4 | 76133765 | 76144667 | 4362 |
| MsG0780036127.01 | -2.58936 | 0.000116 | 0.001997 | Chr7 | 3142859 | 3147994 | 3510 |
| novel.2856 | -6.26985 | 0.000117 | 0.002004 | Chr3 | 34701120 | 34706985 | 1205 |
| MsG0780038383.01 | 2.042258 | 0.000117 | 0.002004 | Chr7 | 45513579 | 45530264 | 700 |
| novel.8521 | -5.20305 | 0.000117 | 0.002004 | Chr8 | 46347567 | 46353968 | 1387 |
| MsG0280011304.01 | -1.50288 | 0.000118 | 0.002021 | Chr2 | 82171911 | 82173160 | 276 |
| MsG0380015667.01 | 6.583569 | 0.000118 | 0.002022 | Chr3 | 73326376 | 73335354 | 999 |
| MsG0680030903.01 | 2.660484 | 0.000119 | 0.002044 | Chr6 | 11348092 | 11354288 | 1509 |
| novel.6067 | -6.00064 | 0.00012 | 0.002059 | Chr6 | 1.05E+08 | 1.05E+08 | 1652 |
| MsG0880045709.01 | -6.0811 | 0.000121 | 0.002063 | Chr8 | 63081420 | 63081944 | 318 |
| novel.600 | -6.47921 | 0.000121 | 0.002068 | Chr1 | 2373281 | 2374543 | 311 |
| novel.4115 | -6.4019 | 0.000122 | 0.002074 | Chr4 | 70364957 | 70365776 | 820 |
| MsG0380014314.01 | 4.66517 | 0.000122 | 0.002081 | Chr3 | 52840373 | 52844978 | 1168 |
| MsG0680030720.01 | -1.49456 | 0.000122 | 0.002081 | Chr6 | 7932479 | 7936749 | 834 |
| MsG0880045030.01 | -1.68783 | 0.000122 | 0.002082 | Chr8 | 52983813 | 52988855 | 1837 |
| MsG0080048726.01 | -4.40367 | 0.000122 | 0.002085 | contig408end | 101814 | 109368 | 2892 |
| MsG0380015920.01 | -2.12514 | 0.000123 | 0.002097 | Chr3 | 76633816 | 76641455 | 4212 |
| MsG0780039422.01 | 1.755426 | 0.000123 | 0.002099 | Chr7 | 63858227 | 63863066 | 737 |
| MsG0880043823.01 | -4.21996 | 0.000124 | 0.002102 | Chr8 | 31223380 | 31223835 | 456 |
| MsG0880042660.01 | -2.49186 | 0.000125 | 0.002119 | Chr8 | 12089970 | 12092036 | 2067 |
| MsG0280010397.01 | 6.477611 | 0.000125 | 0.002123 | Chr2 | 69005763 | 69006608 | 846 |
| MsG0180001864.01 | 2.033377 | 0.000125 | 0.002123 | Chr1 | 28381073 | 28385301 | 1002 |
| MsG0580028967.01 | 6.065203 | 0.000125 | 0.002127 | Chr5 | 89705136 | 89705336 | 201 |
| MsG0580028786.01 | 3.49283 | 0.000127 | 0.002143 | Chr5 | 86397533 | 86410602 | 3324 |
| MsG0280006360.01 | -2.82969 | 0.000127 | 0.002143 | Chr2 | 880301 | 883624 | 2160 |
| MsG0580029805.01 | -2.72823 | 0.000127 | 0.002156 | Chr5 | 1.02E+08 | 1.02E+08 | 1949 |
| MsG0580030012.01 | 3.382435 | 0.000128 | 0.002163 | Chr5 | 1.05E+08 | 1.05E+08 | 3617 |
| MsG0580025875.01 | 1.66085 | 0.000129 | 0.002183 | Chr5 | 25665044 | 25692286 | 6042 |
| MsG0680034705.01 | -5.46632 | 0.000129 | 0.002188 | Chr6 | 93154468 | 93161694 | 1050 |
| MsG0680030946.01 | -5.82379 | 0.00013 | 0.002196 | Chr6 | 11996436 | 12004263 | 3321 |
| MsG0180004278.01 | -3.14634 | 0.00013 | 0.002196 | Chr1 | 75936482 | 75937167 | 477 |
| MsG0580028990.01 | 6.243094 | 0.00013 | 0.002197 | Chr5 | 90031349 | 90035524 | 1103 |
| MsG0880045672.01 | -1.68693 | 0.00013 | 0.002198 | Chr8 | 62497717 | 62502480 | 2443 |
| MsG0380017386.01 | -2.98055 | 0.000131 | 0.002206 | Chr3 | 95694213 | 95696322 | 1305 |
| MsG0880047718.01 | 6.262223 | 0.000132 | 0.002216 | Chr8 | 89971199 | 89981134 | 1089 |
| MsG0780036002.01 | -5.24233 | 0.000132 | 0.002224 | Chr7 | 1155852 | 1163731 | 1006 |
| MsG0780038825.01 | -2.476 | 0.000133 | 0.002244 | Chr7 | 53637657 | 53647408 | 2649 |
| novel.5940 | 2.286687 | 0.000134 | 0.002246 | Chr6 | 86567381 | 86575603 | 1400 |
| MsG0880042676.01 | 3.374347 | 0.000134 | 0.002246 | Chr8 | 12431109 | 12431870 | 762 |
| novel.1541 | 1.906096 | 0.000134 | 0.002246 | Chr2 | 72726178 | 72732789 | 2703 |
| MsG0380017366.01 | -2.07559 | 0.000134 | 0.002246 | Chr3 | 95364422 | 95368145 | 1052 |
| MsG0680035722.01 | 3.405692 | 0.000134 | 0.002246 | Chr6 | 1.11E+08 | 1.11E+08 | 3675 |
| MsG0880045774.01 | -6.45224 | 0.000134 | 0.002251 | Chr8 | 64196089 | 64197565 | 603 |
| novel.3381 | -1.65762 | 0.000135 | 0.002254 | Chr4 | 28111166 | 28115310 | 914 |
| MsG0380014456.01 | -3.48275 | 0.000135 | 0.00226 | Chr3 | 55068336 | 55070084 | 1569 |
| novel.8480 | -3.33436 | 0.000136 | 0.002271 | Chr8 | 36243643 | 36249113 | 5471 |
| novel.2109 | 6.406849 | 0.000136 | 0.002271 | Chr2 | 83486906 | 83489053 | 1420 |
| MsG0880046093.01 | -6.48385 | 0.000136 | 0.002277 | Chr8 | 68944702 | 68945172 | 471 |
| MsG0280007262.01 | -4.77667 | 0.000137 | 0.002284 | Chr2 | 12735608 | 12736408 | 354 |
| novel.4517 | 6.242397 | 0.000137 | 0.002291 | Chr5 | 42733449 | 42736361 | 620 |
| novel.8235 | -6.6165 | 0.000138 | 0.002309 | Chr8 | 83119618 | 83119937 | 320 |
| MsG0280008749.01 | 4.257799 | 0.000139 | 0.002313 | Chr2 | 37653250 | 37654470 | 1221 |
| MsG0880045029.01 | -1.50365 | 0.000139 | 0.002315 | Chr8 | 52928409 | 52931349 | 1956 |
| MsG0580029758.01 | -2.20138 | 0.00014 | 0.002334 | Chr5 | 1.02E+08 | 1.02E+08 | 1462 |
| novel.9027 | -3.59325 | 0.00014 | 0.002334 | contig445end | 1 | 4684 | 4367 |
| MsG0080049004.01 | 1.252809 | 0.00014 | 0.002334 | contig602end | 10001 | 11721 | 1721 |
| MsG0880044008.01 | 2.368244 | 0.000141 | 0.002342 | Chr8 | 34254373 | 34256142 | 1770 |
| MsG0880042641.01 | 7.09254 | 0.000141 | 0.002344 | Chr8 | 11831655 | 11836307 | 2421 |
| MsG0780039087.01 | 3.683145 | 0.000141 | 0.002348 | Chr7 | 57755706 | 57762900 | 5654 |
| novel.6590 | 2.155684 | 0.000142 | 0.00235 | Chr6 | 81727758 | 81730201 | 1804 |
| MsG0280007259.01 | 2.427525 | 0.000142 | 0.00235 | Chr2 | 12690213 | 12697043 | 1155 |
| MsG0280006317.01 | -5.75024 | 0.000142 | 0.00235 | Chr2 | 399657 | 400343 | 549 |
| MsG0780041623.01 | 6.480609 | 0.000143 | 0.002373 | Chr7 | 93177983 | 93183662 | 738 |
| novel.7337 | -5.86902 | 0.000143 | 0.002373 | Chr7 | 10783632 | 10788340 | 982 |
| MsG0780037374.01 | 7.216474 | 0.000144 | 0.00238 | Chr7 | 24570004 | 24570348 | 345 |
| novel.4858 | -3.28772 | 0.000144 | 0.00238 | Chr5 | 1.02E+08 | 1.02E+08 | 578 |
| MsG0680032923.01 | 6.197792 | 0.000144 | 0.002385 | Chr6 | 53078362 | 53080279 | 883 |
| MsG0180000651.01 | -5.18687 | 0.000144 | 0.002388 | Chr1 | 9218579 | 9221264 | 711 |
| MsG0880047417.01 | 2.897291 | 0.000145 | 0.002397 | Chr8 | 85977641 | 85978374 | 448 |
| MsG0880042238.01 | -4.74534 | 0.000145 | 0.002398 | Chr8 | 5934476 | 5935906 | 1431 |
| MsG0880046691.01 | 6.285555 | 0.000145 | 0.002398 | Chr8 | 76820744 | 76821976 | 1233 |
| MsG0880042068.01 | 3.433554 | 0.000145 | 0.0024 | Chr8 | 3326102 | 3334526 | 1797 |
| MsG0280007751.01 | -3.02572 | 0.000146 | 0.002401 | Chr2 | 20043853 | 20046233 | 1524 |
| novel.2251 | -4.42823 | 0.000146 | 0.002403 | Chr3 | 21624648 | 21625213 | 566 |
| MsG0780039166.01 | 6.44904 | 0.000147 | 0.00242 | Chr7 | 58963496 | 58965039 | 795 |
| MsG0480018184.01 | -1.54599 | 0.000147 | 0.00242 | Chr4 | 1292282 | 1301576 | 4056 |
| novel.2473 | 6.566244 | 0.000147 | 0.00242 | Chr3 | 74421840 | 74425480 | 1653 |
| MsG0880043126.01 | -2.63458 | 0.000149 | 0.002447 | Chr8 | 19365287 | 19370709 | 1768 |
| MsG0480023631.01 | 2.483141 | 0.000149 | 0.002449 | Chr4 | 87923243 | 87930145 | 1320 |
| MsG0180004752.01 | -2.14279 | 0.000149 | 0.002451 | Chr1 | 82204442 | 82210603 | 4077 |
| MsG0580025403.01 | -2.47324 | 0.000149 | 0.002452 | Chr5 | 18095459 | 18103360 | 3525 |
| novel.1250 | -6.4817 | 0.00015 | 0.002453 | Chr2 | 14569478 | 14571563 | 1030 |
| MsG0480022662.01 | 4.276748 | 0.00015 | 0.002455 | Chr4 | 75222155 | 75225878 | 1062 |
| MsG0480018426.01 | 1.8284 | 0.00015 | 0.002455 | Chr4 | 4747423 | 4751185 | 3763 |
| MsG0180000772.01 | 4.818224 | 0.00015 | 0.002455 | Chr1 | 10900800 | 10917684 | 4218 |
| MsG0780041514.01 | 3.999768 | 0.00015 | 0.002456 | Chr7 | 91873112 | 91875631 | 960 |
| MsG0580030167.01 | 6.338042 | 0.00015 | 0.002457 | Chr5 | 1.08E+08 | 1.08E+08 | 189 |
| MsG0180003420.01 | 4.202709 | 0.00015 | 0.00246 | Chr1 | 62091072 | 62096754 | 1900 |
| novel.5136 | 4.521002 | 0.000151 | 0.002472 | Chr5 | 45592681 | 45602487 | 1786 |
| MsG0380014143.01 | 6.387082 | 0.000152 | 0.00249 | Chr3 | 50330393 | 50330776 | 384 |
| novel.7571 | 6.846267 | 0.000155 | 0.002522 | Chr7 | 65270732 | 65275795 | 4422 |
| MsG0580027803.01 | 2.246809 | 0.000155 | 0.002523 | Chr5 | 68775366 | 68777528 | 1272 |
| novel.3164 | 2.6763 | 0.000155 | 0.002532 | Chr3 | 97710720 | 97712462 | 489 |
| novel.6537 | 6.592344 | 0.000156 | 0.002544 | Chr6 | 69720944 | 69721617 | 592 |
| novel.2278 | -3.32667 | 0.000156 | 0.002546 | Chr3 | 26098970 | 26100542 | 1573 |
| MsG0580024408.01 | -4.12342 | 0.000156 | 0.002547 | Chr5 | 4450142 | 4450839 | 606 |
| novel.4480 | -6.24874 | 0.000157 | 0.002547 | Chr5 | 30718550 | 30720506 | 916 |
| MsG0280010295.01 | -3.4781 | 0.000157 | 0.002549 | Chr2 | 67534029 | 67538150 | 2686 |
| MsG0280009667.01 | -2.23199 | 0.000157 | 0.00255 | Chr2 | 55956953 | 55962354 | 2580 |
| novel.5520 | 3.993489 | 0.000158 | 0.002559 | Chr6 | 2152467 | 2155135 | 1224 |
| MsG0280007501.01 | 1.649093 | 0.000158 | 0.002572 | Chr2 | 16349428 | 16352426 | 2676 |
| MsG0180001133.01 | -5.49292 | 0.000159 | 0.002583 | Chr1 | 16414903 | 16416426 | 1524 |
| MsG0680030945.01 | -6.25758 | 0.000159 | 0.002583 | Chr6 | 11993374 | 11996338 | 2670 |
| MsG0180001487.01 | -4.20646 | 0.00016 | 0.002588 | Chr1 | 22151808 | 22152941 | 1134 |
| MsG0380017988.01 | -2.20202 | 0.000161 | 0.002602 | Chr3 | 1.03E+08 | 1.03E+08 | 1662 |
| MsG0480021558.01 | 1.612524 | 0.000161 | 0.002606 | Chr4 | 59786097 | 59792146 | 2146 |
| novel.1680 | -1.66847 | 0.000162 | 0.002619 | Chr2 | 4470370 | 4475438 | 1989 |
| novel.7294 | -2.39134 | 0.000163 | 0.00263 | Chr7 | 95274413 | 95276406 | 1244 |
| MsG0080047993.01 | -1.89867 | 0.000163 | 0.002638 | contig162end | 27237 | 31558 | 2643 |
| novel.6658 | 2.681551 | 0.000165 | 0.002666 | Chr6 | 96693727 | 96694605 | 879 |
| MsG0680031545.01 | 2.646005 | 0.000168 | 0.002706 | Chr6 | 22035770 | 22041487 | 1974 |
| novel.1787 | -6.24679 | 0.000168 | 0.002713 | Chr2 | 21223037 | 21224492 | 716 |
| MsG0380014485.01 | -2.92038 | 0.000168 | 0.002717 | Chr3 | 55522187 | 55527855 | 996 |
| novel.2011 | 4.307531 | 0.000169 | 0.002724 | Chr2 | 70782936 | 70783524 | 516 |
| MsG0380016708.01 | -2.39233 | 0.000169 | 0.002726 | Chr3 | 86884684 | 86887174 | 1289 |
| MsG0180000295.01 | -2.96718 | 0.000169 | 0.002726 | Chr1 | 4095973 | 4096661 | 381 |
| MsG0580024866.01 | -2.26663 | 0.00017 | 0.002729 | Chr5 | 10996093 | 11013544 | 2193 |
| MsG0780041515.01 | -1.15831 | 0.00017 | 0.002734 | Chr7 | 91904524 | 91914275 | 2249 |
| novel.839 | 2.024425 | 0.00017 | 0.002735 | Chr1 | 40318323 | 40320788 | 1594 |
| novel.1272 | -3.30588 | 0.00017 | 0.002735 | Chr2 | 16820078 | 16820830 | 637 |
| MsG0180002444.01 | 3.964661 | 0.00017 | 0.002737 | Chr1 | 38471073 | 38473638 | 675 |
| MsG0180003484.01 | -7.2057 | 0.000171 | 0.002747 | Chr1 | 63106882 | 63112984 | 852 |
| MsG0880042008.01 | -1.65262 | 0.000171 | 0.002747 | Chr8 | 2393621 | 2396759 | 862 |
| MsG0180005692.01 | -2.3425 | 0.000172 | 0.00275 | Chr1 | 94985863 | 94991591 | 2268 |
| MsG0280008227.01 | 1.407449 | 0.000172 | 0.002753 | Chr2 | 27457750 | 27460573 | 816 |
| MsG0480021730.01 | 6.864445 | 0.000174 | 0.00278 | Chr4 | 63665345 | 63666231 | 711 |
| novel.1925 | -1.88349 | 0.000174 | 0.002786 | Chr2 | 54258249 | 54260074 | 1826 |
| novel.5925 | -2.14353 | 0.000175 | 0.002805 | Chr6 | 83775770 | 83780992 | 2008 |
| novel.6486 | -2.05465 | 0.000176 | 0.002809 | Chr6 | 59084501 | 59086032 | 926 |
| novel.324 | 4.287902 | 0.000177 | 0.002823 | Chr1 | 60520310 | 60523069 | 1633 |
| MsG0580026716.01 | 6.361929 | 0.000177 | 0.002833 | Chr5 | 42793331 | 42795082 | 714 |
| MsG0280009745.01 | -6.2612 | 0.000178 | 0.002836 | Chr2 | 57455858 | 57458430 | 1234 |
| MsG0880042727.01 | -1.97865 | 0.000178 | 0.002844 | Chr8 | 13251789 | 13263456 | 1527 |
| novel.6183 | -3.02501 | 0.000179 | 0.002853 | Chr6 | 7545067 | 7547800 | 1972 |
| MsG0680033552.01 | 6.289289 | 0.000179 | 0.002853 | Chr6 | 68020496 | 68028072 | 3741 |
| MsG0380017065.01 | -2.96275 | 0.000179 | 0.002858 | Chr3 | 91531165 | 91533107 | 579 |
| MsG0780038077.01 | 2.418628 | 0.00018 | 0.002868 | Chr7 | 39608516 | 39609641 | 753 |
| novel.5729 | -1.97452 | 0.000183 | 0.002906 | Chr6 | 40212073 | 40215524 | 1346 |
| novel.8332 | 1.553319 | 0.000183 | 0.002907 | Chr8 | 6564474 | 6567548 | 765 |
| MsG0180005893.01 | -3.88757 | 0.000183 | 0.002909 | Chr1 | 97220278 | 97221775 | 1179 |
| MsG0480020963.01 | -2.14469 | 0.000183 | 0.002909 | Chr4 | 51264854 | 51270893 | 1574 |
| MsG0380012099.01 | -3.50464 | 0.000184 | 0.002914 | Chr3 | 10898076 | 10899476 | 1401 |
| novel.4260 | -3.00487 | 0.000185 | 0.002929 | Chr4 | 90878325 | 90878865 | 316 |
| MsG0380016745.01 | 1.468625 | 0.000185 | 0.00294 | Chr3 | 87338418 | 87343610 | 660 |
| novel.4924 | -4.61935 | 0.000186 | 0.002943 | Chr5 | 2678899 | 2685809 | 907 |
| MsG0180000447.01 | 3.842689 | 0.000187 | 0.002964 | Chr1 | 6177460 | 6185124 | 1359 |
| novel.3824 | 2.649767 | 0.000188 | 0.002972 | Chr4 | 14966673 | 14968711 | 1545 |
| MsG0580024072.01 | -6.35335 | 0.000189 | 0.002987 | Chr5 | 470812 | 471330 | 519 |
| novel.1592 | 1.867186 | 0.00019 | 0.003001 | Chr2 | 79378727 | 79381097 | 985 |
| MsG0180003462.01 | -3.17825 | 0.00019 | 0.003001 | Chr1 | 62641407 | 62642767 | 962 |
| MsG0480019426.01 | 3.294757 | 0.00019 | 0.003007 | Chr4 | 20194784 | 20198941 | 1854 |
| novel.8008 | 2.632048 | 0.000191 | 0.003013 | Chr8 | 40708028 | 40719722 | 1882 |
| novel.543 | -6.65515 | 0.000191 | 0.003019 | Chr1 | 96403821 | 96404718 | 402 |
| novel.2144 | 6.644721 | 0.000191 | 0.00302 | Chr3 | 4441829 | 4443429 | 966 |
| MsG0780039956.01 | 1.865744 | 0.000192 | 0.003035 | Chr7 | 71077690 | 71082836 | 1662 |
| MsG0280006498.01 | -3.4998 | 0.000193 | 0.003037 | Chr2 | 2759127 | 2764943 | 2779 |
| novel.6014 | 2.477213 | 0.000193 | 0.003037 | Chr6 | 99631912 | 99635783 | 1157 |
| MsG0380012161.01 | -1.62253 | 0.000193 | 0.003045 | Chr3 | 11819787 | 11835362 | 5279 |
| novel.5897 | -3.83546 | 0.000194 | 0.003045 | Chr6 | 78308144 | 78309337 | 899 |
| novel.8777 | -6.18536 | 0.000194 | 0.003045 | Chr8 | 90025676 | 90027830 | 1542 |
| MsG0380014425.01 | -4.8822 | 0.000195 | 0.003059 | Chr3 | 54633608 | 54635417 | 1440 |
| MsG0180000294.01 | -5.31932 | 0.000195 | 0.003071 | Chr1 | 4079836 | 4082894 | 1850 |
| MsG0180002935.01 | -4.45344 | 0.000196 | 0.003072 | Chr1 | 53376050 | 53384122 | 1548 |
| MsG0680032234.01 | 3.260706 | 0.000196 | 0.003072 | Chr6 | 37063230 | 37073882 | 1428 |
| MsG0880045877.01 | 2.998876 | 0.000196 | 0.003072 | Chr8 | 65879677 | 65880687 | 1011 |
| MsG0680035203.01 | -2.69622 | 0.000197 | 0.003082 | Chr6 | 1.01E+08 | 1.01E+08 | 4668 |
| MsG0880043298.01 | 2.543453 | 0.000197 | 0.003094 | Chr8 | 22181016 | 22181234 | 219 |
| MsG0780037857.01 | -3.34494 | 0.000198 | 0.003097 | Chr7 | 34787944 | 34789001 | 708 |
| novel.7787 | 6.202997 | 0.000198 | 0.003099 | Chr7 | 95291495 | 95292594 | 546 |
| MsG0380015351.01 | 6.274264 | 0.000199 | 0.003108 | Chr3 | 68904377 | 68906578 | 885 |
| MsG0180000304.01 | -3.5668 | 0.000199 | 0.003108 | Chr1 | 4204089 | 4206522 | 1417 |
| novel.9209 | -6.38226 | 0.000199 | 0.003111 | contig549end | 16136 | 22060 | 2425 |
| novel.2953 | -6.30614 | 0.0002 | 0.003129 | Chr3 | 61381042 | 61387672 | 682 |
| MsG0580028722.01 | -1.6289 | 0.0002 | 0.003129 | Chr5 | 85354016 | 85356609 | 570 |
| MsG0880044386.01 | 2.056436 | 0.000201 | 0.003132 | Chr8 | 41154719 | 41161047 | 2518 |
| MsG0580026935.01 | 2.172888 | 0.000201 | 0.003135 | Chr5 | 48873887 | 48906017 | 3066 |
| MsG0880046345.01 | -4.85316 | 0.000201 | 0.003135 | Chr8 | 72258351 | 72259115 | 765 |
| MsG0180000153.01 | -2.47357 | 0.000201 | 0.003135 | Chr1 | 2141202 | 2142854 | 1653 |
| MsG0680034952.01 | -6.30173 | 0.000201 | 0.003135 | Chr6 | 97043881 | 97046067 | 1377 |
| MsG0480019194.01 | -3.48336 | 0.000203 | 0.003157 | Chr4 | 15802328 | 15811698 | 1740 |
| MsG0480021781.01 | -2.51357 | 0.000203 | 0.003159 | Chr4 | 64200281 | 64200724 | 444 |
| novel.384 | 7.014932 | 0.000203 | 0.003159 | Chr1 | 68832076 | 68832608 | 453 |
| MsG0880047067.01 | -2.65423 | 0.000204 | 0.003163 | Chr8 | 81523095 | 81523940 | 846 |
| novel.3756 | -6.40899 | 0.000204 | 0.003163 | Chr4 | 1393635 | 1394256 | 538 |
| MsG0780036750.01 | -6.28913 | 0.000204 | 0.003164 | Chr7 | 13157087 | 13157317 | 231 |
| MsG0680035458.01 | -2.20134 | 0.000204 | 0.003167 | Chr6 | 1.06E+08 | 1.06E+08 | 1056 |
| novel.3629 | 1.575036 | 0.000204 | 0.003169 | Chr4 | 77852460 | 77852810 | 323 |
| MsG0380016265.01 | 2.704904 | 0.000205 | 0.003181 | Chr3 | 81227327 | 81227776 | 450 |
| MsG0280007237.01 | -6.33441 | 0.000206 | 0.003186 | Chr2 | 12390804 | 12391082 | 279 |
| novel.6960 | -2.4888 | 0.000207 | 0.003202 | Chr7 | 36347357 | 36349975 | 461 |
| novel.8885 | -1.93705 | 0.000207 | 0.003208 | contig252end | 29055 | 32307 | 996 |
| novel.3497 | -6.4018 | 0.000208 | 0.003214 | Chr4 | 56149602 | 56153051 | 590 |
| novel.4878 | 6.13592 | 0.000208 | 0.003216 | Chr5 | 1.05E+08 | 1.05E+08 | 658 |
| MsG0380012782.01 | 1.716512 | 0.000208 | 0.003216 | Chr3 | 24465634 | 24470975 | 1471 |
| MsG0680032026.01 | -2.32869 | 0.00021 | 0.003243 | Chr6 | 32182215 | 32183059 | 845 |
| MsG0580028563.01 | 3.283155 | 0.00021 | 0.003244 | Chr5 | 82454205 | 82456039 | 1434 |
| MsG0180004266.01 | -4.42253 | 0.00021 | 0.003244 | Chr1 | 75762510 | 75763820 | 1311 |
| MsG0880045826.01 | -3.68055 | 0.000211 | 0.003246 | Chr8 | 65018073 | 65021522 | 1171 |
| MsG0480022160.01 | -4.56006 | 0.000211 | 0.003248 | Chr4 | 69105162 | 69107887 | 753 |
| MsG0780036013.01 | -3.75441 | 0.000211 | 0.00325 | Chr7 | 1317267 | 1318012 | 680 |
| MsG0380013404.01 | -3.18707 | 0.000211 | 0.003253 | Chr3 | 36808044 | 36815588 | 5477 |
| novel.5420 | 5.007919 | 0.000212 | 0.003259 | Chr5 | 98483152 | 98487801 | 405 |
| MsG0480020856.01 | -5.1376 | 0.000214 | 0.003283 | Chr4 | 49706963 | 49713198 | 611 |
| novel.6958 | 6.349995 | 0.000214 | 0.003288 | Chr7 | 35856249 | 35856809 | 399 |
| MsG0280011373.01 | 6.03127 | 0.000214 | 0.003288 | Chr2 | 83195617 | 83197445 | 972 |
| MsG0580029613.01 | -1.30114 | 0.000214 | 0.003289 | Chr5 | 99435884 | 99440597 | 1126 |
| novel.3243 | 6.957945 | 0.000216 | 0.003308 | Chr4 | 4873838 | 4876292 | 792 |
| MsG0780038305.01 | 2.182732 | 0.000216 | 0.003311 | Chr7 | 43850775 | 43852923 | 2061 |
| novel.5140 | -3.07509 | 0.000217 | 0.003325 | Chr5 | 46758632 | 46764330 | 5699 |
| MsG0680031826.01 | 1.579064 | 0.000218 | 0.003347 | Chr6 | 27480527 | 27485632 | 1474 |
| MsG0880045115.01 | 1.59459 | 0.000219 | 0.003353 | Chr8 | 54371356 | 54371637 | 282 |
| novel.3605 | 3.441831 | 0.00022 | 0.003371 | Chr4 | 74329969 | 74334385 | 2160 |
| MsG0680034290.01 | -3.24928 | 0.00022 | 0.003371 | Chr6 | 85113715 | 85118410 | 885 |
| MsG0680032536.01 | -2.12322 | 0.000221 | 0.00338 | Chr6 | 44112503 | 44125446 | 2146 |
| MsG0780041810.01 | -2.11008 | 0.000221 | 0.003383 | Chr7 | 95388427 | 95390694 | 1348 |
| MsG0580029198.01 | -2.79269 | 0.000223 | 0.003404 | Chr5 | 93553037 | 93558750 | 998 |
| MsG0380018001.01 | 1.462164 | 0.000223 | 0.003406 | Chr3 | 1.03E+08 | 1.03E+08 | 716 |
| novel.2694 | 6.274276 | 0.000223 | 0.003406 | Chr3 | 3682237 | 3684412 | 481 |
| MsG0480021682.01 | 1.687432 | 0.000223 | 0.003406 | Chr4 | 62913590 | 62916934 | 934 |
| MsG0880043078.01 | 1.912326 | 0.000223 | 0.003406 | Chr8 | 18622887 | 18628673 | 1652 |
| novel.3957 | -2.38758 | 0.000224 | 0.003408 | Chr4 | 45756532 | 45802890 | 7660 |
| MsG0680030529.01 | -3.57919 | 0.000226 | 0.003435 | Chr6 | 4757190 | 4760342 | 864 |
| novel.4392 | -4.20904 | 0.000227 | 0.003454 | Chr5 | 17169414 | 17170656 | 678 |
| MsG0180002800.01 | -1.86926 | 0.000228 | 0.003471 | Chr1 | 45458563 | 45467199 | 1725 |
| MsG0480018318.01 | -1.54235 | 0.000229 | 0.003475 | Chr4 | 3269605 | 3273463 | 1000 |
| novel.2076 | 6.755151 | 0.000229 | 0.003475 | Chr2 | 80168266 | 80170036 | 1131 |
| novel.2379 | -6.17916 | 0.000229 | 0.003479 | Chr3 | 53822040 | 53823281 | 1148 |
| MsG0480020229.01 | -1.99937 | 0.00023 | 0.003488 | Chr4 | 36937212 | 36941040 | 2943 |
| novel.8205 | 6.35169 | 0.00023 | 0.003488 | Chr8 | 77489328 | 77490472 | 839 |
| MsG0880046106.01 | 6.398457 | 0.00023 | 0.003493 | Chr8 | 69094020 | 69095086 | 618 |
| MsG0880042301.01 | -1.70147 | 0.000231 | 0.003494 | Chr8 | 6767725 | 6775501 | 2004 |
| novel.96 | 6.251671 | 0.000231 | 0.003498 | Chr1 | 12525005 | 12525904 | 687 |
| MsG0280006923.01 | 2.780369 | 0.000232 | 0.003507 | Chr2 | 8015514 | 8015690 | 177 |
| MsG0680035741.01 | -6.67895 | 0.000232 | 0.003511 | Chr6 | 1.11E+08 | 1.11E+08 | 1134 |
| MsG0680034233.01 | -2.40085 | 0.000232 | 0.003512 | Chr6 | 84183182 | 84187969 | 1236 |
| novel.3951 | 4.612638 | 0.000233 | 0.003521 | Chr4 | 43565626 | 43568371 | 1201 |
| MsG0680031141.01 | 2.224321 | 0.000233 | 0.003521 | Chr6 | 15052112 | 15054226 | 1938 |
| MsG0680030615.01 | 3.602284 | 0.000235 | 0.003547 | Chr6 | 6104845 | 6109758 | 2371 |
| MsG0780035991.01 | 1.653364 | 0.000235 | 0.003547 | Chr7 | 1025690 | 1040948 | 2440 |
| MsG0680034729.01 | 3.070464 | 0.000235 | 0.003547 | Chr6 | 93530150 | 93532171 | 1436 |
| MsG0880042515.01 | -1.95602 | 0.000237 | 0.003576 | Chr8 | 9888103 | 9891014 | 1658 |
| MsG0580027474.01 | -3.07828 | 0.000238 | 0.003594 | Chr5 | 61745926 | 61754402 | 2073 |
| MsG0680031843.01 | -2.26871 | 0.000239 | 0.003596 | Chr6 | 27905782 | 27911929 | 3016 |
| novel.1912 | 1.552013 | 0.000239 | 0.003598 | Chr2 | 51287344 | 51289237 | 866 |
| novel.3467 | -2.49727 | 0.00024 | 0.00361 | Chr4 | 49837454 | 49838603 | 1150 |
| MsG0580027918.01 | -4.48944 | 0.000241 | 0.003619 | Chr5 | 70956701 | 70969811 | 2610 |
| novel.6766 | -3.14007 | 0.000241 | 0.003621 | Chr6 | 1.1E+08 | 1.1E+08 | 373 |
| MsG0880043277.01 | -3.33974 | 0.000241 | 0.003623 | Chr8 | 21880693 | 21890902 | 1750 |
| novel.3410 | -2.32612 | 0.000242 | 0.003635 | Chr4 | 36542994 | 36552431 | 2992 |
| MsG0480018851.01 | -2.38616 | 0.000242 | 0.003635 | Chr4 | 10589167 | 10593064 | 1713 |
| MsG0480022069.01 | -2.02969 | 0.000242 | 0.003635 | Chr4 | 67691852 | 67695114 | 2130 |
| MsG0380016671.01 | 1.306471 | 0.000243 | 0.003639 | Chr3 | 86430071 | 86431018 | 716 |
| MsG0680031140.01 | 2.446121 | 0.000244 | 0.003654 | Chr6 | 15039916 | 15044324 | 1788 |
| MsG0780037459.01 | -1.59235 | 0.000244 | 0.003657 | Chr7 | 26279310 | 26298108 | 4335 |
| MsG0880042693.01 | -2.49535 | 0.000245 | 0.003668 | Chr8 | 12673908 | 12676353 | 951 |
| MsG0580028567.01 | -6.17939 | 0.000245 | 0.003673 | Chr5 | 82503823 | 82505670 | 1231 |
| MsG0480019111.01 | 5.605354 | 0.000247 | 0.003698 | Chr4 | 14720844 | 14721971 | 1128 |
| MsG0380015137.01 | 3.692979 | 0.000247 | 0.003699 | Chr3 | 65744728 | 65746113 | 1386 |
| MsG0480022025.01 | -3.49281 | 0.000248 | 0.003699 | Chr4 | 67135969 | 67138264 | 1914 |
| MsG0480023858.01 | 2.333596 | 0.000248 | 0.003699 | Chr4 | 90562342 | 90585195 | 2205 |
| MsG0580026412.01 | 6.148784 | 0.000248 | 0.003709 | Chr5 | 36723875 | 36724306 | 432 |
| MsG0380014941.01 | 2.659773 | 0.000249 | 0.003717 | Chr3 | 62824359 | 62825054 | 696 |
| novel.5196 | -6.26896 | 0.000249 | 0.003717 | Chr5 | 58357859 | 58359449 | 530 |
| MsG0180004091.01 | 6.206624 | 0.000249 | 0.003718 | Chr1 | 73186648 | 73187704 | 1057 |
| MsG0480023998.01 | 1.771935 | 0.00025 | 0.003718 | Chr4 | 92206452 | 92209938 | 2086 |
| MsG0780036014.01 | -1.89768 | 0.000252 | 0.003747 | Chr7 | 1318655 | 1322183 | 1455 |
| novel.8123 | 2.513412 | 0.000255 | 0.003791 | Chr8 | 61753864 | 61756601 | 1327 |
| MsG0380017686.01 | -3.15037 | 0.000256 | 0.003808 | Chr3 | 99543977 | 99548813 | 2181 |
| MsG0680035769.01 | -2.47645 | 0.000256 | 0.003808 | Chr6 | 1.12E+08 | 1.12E+08 | 1167 |
| novel.3239 | 5.562273 | 0.000256 | 0.003811 | Chr4 | 4716977 | 4719525 | 1190 |
| MsG0480023369.01 | 6.380208 | 0.000257 | 0.00382 | Chr4 | 84266590 | 84269451 | 1344 |
| MsG0180001129.01 | -4.23092 | 0.000257 | 0.00382 | Chr1 | 16360838 | 16363921 | 934 |
| novel.2662 | -6.18674 | 0.000258 | 0.00382 | Chr3 | 1.03E+08 | 1.03E+08 | 749 |
| novel.7361 | 6.518043 | 0.000258 | 0.00382 | Chr7 | 15325224 | 15327127 | 1904 |
| MsG0880045858.01 | 3.833654 | 0.000258 | 0.003823 | Chr8 | 65549780 | 65550067 | 288 |
| MsG0880044659.01 | -2.35991 | 0.000258 | 0.00383 | Chr8 | 46806772 | 46807119 | 348 |
| MsG0480021926.01 | 1.598772 | 0.000259 | 0.003843 | Chr4 | 66062502 | 66065204 | 1461 |
| MsG0680031480.01 | 6.681346 | 0.000261 | 0.003856 | Chr6 | 21040839 | 21051292 | 1461 |
| MsG0080048731.01 | -3.27074 | 0.000261 | 0.003856 | contig408end | 161170 | 172004 | 3078 |
| MsG0380015326.01 | -3.47235 | 0.000261 | 0.003856 | Chr3 | 68471649 | 68483621 | 1599 |
| MsG0580027788.01 | -1.7979 | 0.000261 | 0.003857 | Chr5 | 68485208 | 68485483 | 276 |
| MsG0480020392.01 | -5.14958 | 0.000262 | 0.003872 | Chr4 | 40390106 | 40390871 | 645 |
| MsG0680030791.01 | 2.07339 | 0.000262 | 0.003872 | Chr6 | 9442055 | 9446740 | 1159 |
| MsG0380016668.01 | 1.275393 | 0.000264 | 0.003891 | Chr3 | 86412751 | 86414988 | 1171 |
| novel.3425 | 6.612709 | 0.000265 | 0.003908 | Chr4 | 39173966 | 39181813 | 674 |
| MsG0580024421.01 | 1.696162 | 0.000266 | 0.003922 | Chr5 | 4722694 | 4732859 | 2919 |
| MsG0580026160.01 | -1.14111 | 0.000267 | 0.003941 | Chr5 | 31552430 | 31578105 | 4091 |
| MsG0480018141.01 | 2.360294 | 0.000268 | 0.003943 | Chr4 | 813788 | 816637 | 889 |
| novel.7083 | -2.37133 | 0.000268 | 0.003952 | Chr7 | 65581169 | 65583986 | 1101 |
| novel.2586 | -6.41608 | 0.000269 | 0.003953 | Chr3 | 90873658 | 90875089 | 1432 |
| MsG0880047286.01 | 1.342341 | 0.000269 | 0.003953 | Chr8 | 84183737 | 84193442 | 3276 |
| novel.722 | 6.689975 | 0.000269 | 0.003956 | Chr1 | 17359236 | 17361382 | 2147 |
| novel.6061 | 6.09643 | 0.000271 | 0.003981 | Chr6 | 1.05E+08 | 1.05E+08 | 497 |
| novel.7236 | 1.629832 | 0.000271 | 0.003981 | Chr7 | 87658702 | 87665578 | 3296 |
| MsG0580029987.01 | 1.988282 | 0.000272 | 0.003988 | Chr5 | 1.05E+08 | 1.05E+08 | 2457 |
| MsG0480018217.01 | -2.37292 | 0.000272 | 0.003991 | Chr4 | 1735725 | 1740217 | 1781 |
| MsG0380012002.01 | -1.88313 | 0.000274 | 0.004016 | Chr3 | 9300392 | 9314023 | 5515 |
| MsG0880043588.01 | -2.27826 | 0.000275 | 0.004025 | Chr8 | 27351411 | 27353067 | 915 |
| novel.6300 | 6.34443 | 0.000275 | 0.004029 | Chr6 | 22539742 | 22541300 | 936 |
| MsG0780040185.01 | -3.06204 | 0.000276 | 0.004036 | Chr7 | 74071227 | 74072093 | 867 |
| MsG0480021012.01 | -1.65017 | 0.000277 | 0.004053 | Chr4 | 52050160 | 52052586 | 1185 |
| novel.3282 | -5.90381 | 0.000277 | 0.004055 | Chr4 | 10783684 | 10785120 | 1437 |
| MsG0580028947.01 | 5.306975 | 0.000278 | 0.004057 | Chr5 | 89390100 | 89393839 | 1581 |
| MsG0180003711.01 | 2.623216 | 0.00028 | 0.004091 | Chr1 | 67210716 | 67211126 | 411 |
| novel.1868 | -2.08735 | 0.000281 | 0.0041 | Chr2 | 36933377 | 36939284 | 2233 |
| MsG0580028377.01 | -2.3354 | 0.000281 | 0.004109 | Chr5 | 79388534 | 79389484 | 951 |
| novel.5596 | 6.359991 | 0.000283 | 0.004135 | Chr6 | 15264855 | 15265197 | 343 |
| novel.6236 | 2.494375 | 0.000284 | 0.004139 | Chr6 | 13410568 | 13411041 | 360 |
| novel.7341 | 6.296168 | 0.000284 | 0.004142 | Chr7 | 11300264 | 11301296 | 410 |
| MsG0280008595.01 | -2.3565 | 0.000284 | 0.004142 | Chr2 | 34576553 | 34592070 | 2537 |
| MsG0880044832.01 | -3.4134 | 0.000285 | 0.004152 | Chr8 | 49890998 | 49894336 | 2124 |
| MsG0380013854.01 | 1.73291 | 0.000285 | 0.004152 | Chr3 | 46713024 | 46721419 | 2003 |
| MsG0480022319.01 | -2.45388 | 0.000285 | 0.004153 | Chr4 | 71079494 | 71083867 | 1803 |
| MsG0380013945.01 | -2.22728 | 0.000287 | 0.00417 | Chr3 | 48485755 | 48486438 | 684 |
| MsG0780041786.01 | -1.4574 | 0.000287 | 0.004175 | Chr7 | 95122561 | 95122911 | 351 |
| MsG0580028808.01 | 3.152809 | 0.000288 | 0.00418 | Chr5 | 86776807 | 86780436 | 1176 |
| MsG0580024804.01 | 1.511445 | 0.000289 | 0.004189 | Chr5 | 9939563 | 9944515 | 822 |
| novel.1097 | -6.12509 | 0.000289 | 0.004189 | Chr1 | 93788730 | 93791068 | 2339 |
| MsG0480020783.01 | -1.62143 | 0.000291 | 0.004223 | Chr4 | 48228212 | 48236934 | 1079 |
| novel.4845 | 6.176194 | 0.000292 | 0.004228 | Chr5 | 1.01E+08 | 1.01E+08 | 2221 |
| MsG0780040805.01 | -6.36809 | 0.000292 | 0.004236 | Chr7 | 82727222 | 82728013 | 593 |
| MsG0580025726.01 | 2.135492 | 0.000293 | 0.004238 | Chr5 | 23246586 | 23253440 | 966 |
| novel.8038 | -6.08154 | 0.000293 | 0.004243 | Chr8 | 48361942 | 48363073 | 702 |
| MsG0580024616.01 | -2.12747 | 0.000293 | 0.004243 | Chr5 | 7036775 | 7038408 | 765 |
| MsG0180005270.01 | 2.818554 | 0.000295 | 0.004266 | Chr1 | 89408755 | 89409123 | 369 |
| MsG0480020739.01 | -2.52951 | 0.000295 | 0.004271 | Chr4 | 47470402 | 47471817 | 1053 |
| MsG0780041317.01 | -2.90805 | 0.000297 | 0.004293 | Chr7 | 89514625 | 89517563 | 1698 |
| MsG0380015382.01 | 1.894571 | 0.000298 | 0.004299 | Chr3 | 69340394 | 69344624 | 1911 |
| MsG0880042762.01 | 3.597043 | 0.000299 | 0.004313 | Chr8 | 13687300 | 13690067 | 1409 |
| novel.8376 | 6.097256 | 0.000299 | 0.004316 | Chr8 | 13645415 | 13646510 | 842 |
| MsG0180002006.01 | -1.37545 | 0.000299 | 0.004318 | Chr1 | 30885377 | 30893909 | 4988 |
| MsG0380013339.01 | -2.01329 | 0.000302 | 0.004355 | Chr3 | 35534269 | 35540021 | 1221 |
| MsG0680033463.01 | -6.15082 | 0.000303 | 0.004368 | Chr6 | 65831198 | 65833216 | 447 |
| MsG0380016068.01 | -3.70103 | 0.000304 | 0.004372 | Chr3 | 78633460 | 78636004 | 1032 |
| novel.3065 | -2.55631 | 0.000304 | 0.004377 | Chr3 | 80614522 | 80615019 | 226 |
| MsG0480023884.01 | -2.89724 | 0.000304 | 0.004378 | Chr4 | 90873363 | 90878317 | 1895 |
| novel.3752 | -2.83704 | 0.000305 | 0.004382 | Chr4 | 922782 | 925925 | 2139 |
| MsG0380012429.01 | -3.92209 | 0.000305 | 0.004385 | Chr3 | 16409251 | 16415019 | 1822 |
| MsG0880047188.01 | 2.945013 | 0.000306 | 0.004388 | Chr8 | 83105754 | 83106287 | 534 |
| MsG0480018215.01 | -4.03734 | 0.000307 | 0.004408 | Chr4 | 1711220 | 1717180 | 1781 |
| MsG0180004950.01 | 4.507656 | 0.000308 | 0.004419 | Chr1 | 84827621 | 84835339 | 1606 |
| MsG0780041648.01 | -2.31659 | 0.000308 | 0.00442 | Chr7 | 93436555 | 93439912 | 1619 |
| MsG0680031852.01 | -3.63476 | 0.00031 | 0.004443 | Chr6 | 28204853 | 28209146 | 1737 |
| MsG0680035015.01 | -3.21053 | 0.00031 | 0.004445 | Chr6 | 97771104 | 97780401 | 4998 |
| novel.6320 | 2.654916 | 0.000311 | 0.004449 | Chr6 | 24849866 | 24852650 | 885 |
| MsG0780038861.01 | 1.340189 | 0.000312 | 0.004464 | Chr7 | 54241415 | 54244266 | 762 |
| MsG0180004090.01 | 4.249407 | 0.000314 | 0.004488 | Chr1 | 73175781 | 73180964 | 2268 |
| MsG0180003580.01 | -3.70913 | 0.000314 | 0.004488 | Chr1 | 64647797 | 64659691 | 4008 |
| MsG0280007216.01 | -2.54912 | 0.000314 | 0.004488 | Chr2 | 12094866 | 12103374 | 2463 |
| novel.7965 | 3.788812 | 0.000315 | 0.004501 | Chr8 | 31772232 | 31781486 | 2151 |
| novel.6600 | -5.70961 | 0.000316 | 0.004513 | Chr6 | 82809091 | 82810322 | 1085 |
| MsG0780037050.01 | 6.167049 | 0.000316 | 0.004513 | Chr7 | 18585080 | 18587435 | 1648 |
| novel.5615 | -9.18238 | 0.000317 | 0.004525 | Chr6 | 17364158 | 17369555 | 1508 |
| MsG0180000571.01 | 2.583418 | 0.000318 | 0.00453 | Chr1 | 7987784 | 7988464 | 681 |
| MsG0880045803.01 | -1.78055 | 0.000319 | 0.004542 | Chr8 | 64689990 | 64699061 | 2921 |
| novel.1588 | 1.697472 | 0.000319 | 0.004543 | Chr2 | 79068883 | 79071152 | 752 |
| novel.6528 | 5.447188 | 0.000319 | 0.004546 | Chr6 | 68057620 | 68060945 | 1251 |
| MsG0780037292.01 | -3.59632 | 0.00032 | 0.004548 | Chr7 | 23208228 | 23210111 | 822 |
| novel.1524 | -5.06213 | 0.00032 | 0.004554 | Chr2 | 70219126 | 70222040 | 1015 |
| MsG0480021690.01 | 1.874254 | 0.00032 | 0.004557 | Chr4 | 62980587 | 62981573 | 987 |
| novel.1200 | -4.33016 | 0.000324 | 0.004601 | Chr2 | 5594136 | 5595815 | 869 |
| MsG0180001522.01 | 2.516046 | 0.000324 | 0.004603 | Chr1 | 22626180 | 22628242 | 807 |
| MsG0280010891.01 | 3.970969 | 0.000325 | 0.004608 | Chr2 | 76456526 | 76457149 | 624 |
| MsG0580025102.01 | -1.69912 | 0.000326 | 0.004627 | Chr5 | 13866991 | 13867491 | 501 |
| novel.9023 | -3.01167 | 0.000327 | 0.004639 | contig442end | 18902 | 24100 | 4273 |
| MsG0480018585.01 | 1.680339 | 0.000327 | 0.004639 | Chr4 | 6957583 | 6960459 | 1647 |
| MsG0180005255.01 | 1.837134 | 0.000328 | 0.004641 | Chr1 | 89235209 | 89235928 | 720 |
| MsG0680030286.01 | 1.667017 | 0.000328 | 0.004641 | Chr6 | 43082 | 47445 | 1250 |
| MsG0580029001.01 | 2.608977 | 0.000329 | 0.004654 | Chr5 | 90134994 | 90139314 | 1567 |
| MsG0180005037.01 | -2.00179 | 0.000329 | 0.004655 | Chr1 | 86052768 | 86054512 | 618 |
| MsG0780038368.01 | 6.229916 | 0.000329 | 0.004655 | Chr7 | 45229172 | 45252724 | 4785 |
| MsG0280011309.01 | -2.13184 | 0.00033 | 0.004665 | Chr2 | 82201553 | 82208617 | 1032 |
| MsG0380016178.01 | 1.587614 | 0.00033 | 0.004665 | Chr3 | 80011922 | 80012843 | 712 |
| novel.6964 | -6.1999 | 0.000331 | 0.00467 | Chr7 | 37192111 | 37192917 | 585 |
| novel.8190 | 6.017792 | 0.000331 | 0.00467 | Chr8 | 74824503 | 74825672 | 1170 |
| novel.4713 | -4.58307 | 0.000332 | 0.00468 | Chr5 | 79945624 | 79949825 | 2544 |
| MsG0580025693.01 | 3.243722 | 0.000332 | 0.00468 | Chr5 | 22859926 | 22867064 | 1512 |
| novel.7152 | -3.20586 | 0.000333 | 0.00469 | Chr7 | 76591859 | 76594362 | 1387 |
| MsG0580027938.01 | -2.82051 | 0.000334 | 0.004707 | Chr5 | 71512762 | 71520056 | 3537 |
| MsG0880047326.01 | 1.846755 | 0.000334 | 0.004707 | Chr8 | 84711062 | 84711632 | 348 |
| MsG0580027309.01 | -2.3454 | 0.000335 | 0.004714 | Chr5 | 58186741 | 58191801 | 447 |
| novel.8494 | -4.8638 | 0.000335 | 0.004714 | Chr8 | 39263140 | 39264064 | 837 |
| MsG0280011336.01 | -1.43861 | 0.000335 | 0.004717 | Chr2 | 82570767 | 82603950 | 5772 |
| MsG0380014461.01 | 6.128248 | 0.000336 | 0.004726 | Chr3 | 55143035 | 55143604 | 570 |
| novel.598 | 1.622906 | 0.000337 | 0.004737 | Chr1 | 2203198 | 2205284 | 942 |
| MsG0180006249.01 | 1.287336 | 0.000338 | 0.004743 | Chr1 | 1.02E+08 | 1.02E+08 | 1469 |
| MsG0180002724.01 | 5.565137 | 0.000338 | 0.004743 | Chr1 | 43872517 | 43873759 | 1155 |
| MsG0580024389.01 | 6.046844 | 0.00034 | 0.00477 | Chr5 | 4173577 | 4176904 | 1584 |
| MsG0280008456.01 | 2.638897 | 0.000342 | 0.00479 | Chr2 | 32201557 | 32206394 | 3175 |
| MsG0580028109.01 | 3.515562 | 0.000342 | 0.004794 | Chr5 | 74700468 | 74700881 | 414 |
| novel.7907 | 6.291554 | 0.000343 | 0.0048 | Chr8 | 20286616 | 20288166 | 623 |
| MsG0480023377.01 | -1.43145 | 0.000343 | 0.0048 | Chr4 | 84346434 | 84352354 | 338 |
| novel.4744 | -2.82782 | 0.000343 | 0.004805 | Chr5 | 85437166 | 85439801 | 2002 |
| novel.5144 | 4.147689 | 0.000346 | 0.004847 | Chr5 | 47247489 | 47251033 | 1344 |
| novel.3803 | 5.874921 | 0.000348 | 0.004869 | Chr4 | 8112689 | 8113883 | 1081 |
| MsG0280011299.01 | 1.446404 | 0.000349 | 0.004875 | Chr2 | 82136204 | 82136680 | 477 |
| MsG0180006211.01 | 1.497426 | 0.00035 | 0.004892 | Chr1 | 1.01E+08 | 1.01E+08 | 593 |
| MsG0280010152.01 | 1.987021 | 0.000351 | 0.004901 | Chr2 | 65369043 | 65373301 | 1940 |
| MsG0080048267.01 | 1.637448 | 0.000351 | 0.004906 | contig259end | 7541 | 13841 | 1096 |
| MsG0180005784.01 | -1.46824 | 0.000352 | 0.004912 | Chr1 | 96119699 | 96121432 | 815 |
| MsG0280008597.01 | -4.05853 | 0.000353 | 0.004917 | Chr2 | 34604308 | 34606153 | 1022 |
| MsG0580025574.01 | -5.04579 | 0.000353 | 0.004917 | Chr5 | 20729088 | 20733243 | 1271 |
| MsG0280006553.01 | -2.17067 | 0.000355 | 0.004948 | Chr2 | 3386068 | 3386547 | 480 |
| novel.6929 | 2.150334 | 0.000356 | 0.004962 | Chr7 | 29510121 | 29511462 | 553 |
| MsG0380012007.01 | -3.47906 | 0.000357 | 0.004964 | Chr3 | 9438759 | 9445465 | 2082 |
| novel.3856 | 2.705142 | 0.000357 | 0.004964 | Chr4 | 20165061 | 20170732 | 1711 |
| MsG0280009908.01 | -1.85229 | 0.000357 | 0.004964 | Chr2 | 60605789 | 60611000 | 1614 |
| MsG0380012270.01 | -3.21724 | 0.000358 | 0.004968 | Chr3 | 13853094 | 13858952 | 2439 |
| MsG0780036661.01 | -2.28391 | 0.000358 | 0.004968 | Chr7 | 11493770 | 11499668 | 1741 |
| MsG0580025865.01 | 1.500228 | 0.000359 | 0.004987 | Chr5 | 25406510 | 25422335 | 2550 |
| novel.5981 | -1.66876 | 0.00036 | 0.004992 | Chr6 | 94236426 | 94251069 | 4160 |
| MsG0780039794.01 | 3.46289 | 0.00036 | 0.004992 | Chr7 | 69168350 | 69169919 | 738 |
| MsG0280006557.01 | 6.211338 | 0.00036 | 0.004995 | Chr2 | 3410650 | 3418347 | 1842 |
| MsG0880042976.01 | -2.92949 | 0.000362 | 0.005014 | Chr8 | 16930430 | 16931139 | 276 |
| novel.90 | 2.784161 | 0.000362 | 0.005017 | Chr1 | 11673091 | 11675249 | 1959 |
| MsG0280008219.01 | -3.21883 | 0.000364 | 0.005037 | Chr2 | 27338507 | 27339216 | 471 |
| MsG0280008210.01 | 2.310002 | 0.000364 | 0.005041 | Chr2 | 27211068 | 27212010 | 789 |
| MsG0380017965.01 | 5.764797 | 0.000365 | 0.005046 | Chr3 | 1.03E+08 | 1.03E+08 | 1509 |
| novel.7243 | -2.62243 | 0.000365 | 0.005046 | Chr7 | 88877909 | 88878532 | 522 |
| novel.8226 | 3.279702 | 0.000367 | 0.005068 | Chr8 | 81973982 | 81978965 | 4984 |
| novel.5781 | -6.42275 | 0.000367 | 0.005068 | Chr6 | 51266612 | 51267604 | 743 |
| MsG0880045272.01 | -2.73157 | 0.000367 | 0.005068 | Chr8 | 56757904 | 56758314 | 411 |
| MsG0680031952.01 | -2.30326 | 0.000368 | 0.005084 | Chr6 | 30548849 | 30556433 | 1723 |
| novel.1061 | 6.31523 | 0.000368 | 0.005084 | Chr1 | 89976077 | 89978136 | 903 |
| novel.1154 | -5.47126 | 0.000369 | 0.005089 | Chr1 | 1.02E+08 | 1.02E+08 | 462 |
| MsG0580028013.01 | 1.735651 | 0.000369 | 0.005089 | Chr5 | 73161136 | 73164443 | 1199 |
| MsG0180001413.01 | -3.13209 | 0.000371 | 0.005112 | Chr1 | 20769085 | 20774371 | 3933 |
| MsG0880047361.01 | 1.382081 | 0.000372 | 0.005121 | Chr8 | 85259040 | 85259699 | 660 |
| MsG0280008748.01 | -2.53607 | 0.000372 | 0.005121 | Chr2 | 37637069 | 37637761 | 693 |
| MsG0480019644.01 | 2.108364 | 0.000372 | 0.005121 | Chr4 | 23978165 | 23980914 | 699 |
| MsG0680034721.01 | -1.61842 | 0.000373 | 0.00513 | Chr6 | 93410786 | 93423410 | 2818 |
| MsG0480019159.01 | 6.184808 | 0.000374 | 0.005144 | Chr4 | 15225608 | 15229582 | 1446 |
| MsG0580025657.01 | -1.28394 | 0.000375 | 0.005154 | Chr5 | 21978515 | 21983604 | 1638 |
| MsG0580026080.01 | -5.27888 | 0.000375 | 0.005155 | Chr5 | 30317043 | 30319701 | 885 |
| MsG0080048243.01 | -3.81953 | 0.000377 | 0.005173 | contig252end | 2028 | 5207 | 498 |
| MsG0680031718.01 | 2.034601 | 0.000377 | 0.005176 | Chr6 | 25250755 | 25258515 | 1674 |
| MsG0780039576.01 | -3.03564 | 0.000379 | 0.005197 | Chr7 | 66077620 | 66079786 | 1225 |
| novel.221 | -2.71696 | 0.00038 | 0.005197 | Chr1 | 34421188 | 34424261 | 2726 |
| MsG0680035157.01 | -3.25992 | 0.00038 | 0.005197 | Chr6 | 99839350 | 99845740 | 2157 |
| MsG0380014781.01 | -2.75119 | 0.00038 | 0.005197 | Chr3 | 59872990 | 59880572 | 5471 |
| novel.2843 | 6.069378 | 0.00038 | 0.005197 | Chr3 | 33274936 | 33275873 | 670 |
| novel.136 | 5.648036 | 0.00038 | 0.005197 | Chr1 | 17518015 | 17520995 | 1160 |
| novel.3073 | -6.60635 | 0.00038 | 0.005198 | Chr3 | 81812532 | 81812886 | 355 |
| MsG0480019314.01 | -1.635 | 0.000381 | 0.005213 | Chr4 | 18355291 | 18355593 | 303 |
| novel.3095 | 6.043587 | 0.000382 | 0.005213 | Chr3 | 86399485 | 86402485 | 1366 |
| MsG0480020969.01 | 2.523401 | 0.000384 | 0.00525 | Chr4 | 51332035 | 51332481 | 447 |
| MsG0680031204.01 | 1.656315 | 0.000385 | 0.00526 | Chr6 | 16026760 | 16030739 | 903 |
| MsG0880042199.01 | 3.680801 | 0.000385 | 0.00526 | Chr8 | 5272516 | 5275540 | 1365 |
| MsG0380017436.01 | 1.329537 | 0.000386 | 0.005264 | Chr3 | 96232992 | 96237904 | 867 |
| novel.7527 | 1.832062 | 0.000386 | 0.005269 | Chr7 | 54689189 | 54690622 | 660 |
| MsG0380013769.01 | -2.08242 | 0.000387 | 0.005273 | Chr3 | 45401974 | 45404774 | 1458 |
| novel.8562 | -2.48526 | 0.000388 | 0.00528 | Chr8 | 53154040 | 53155162 | 1123 |
| novel.8044 | 6.031436 | 0.000388 | 0.005283 | Chr8 | 49651246 | 49654104 | 511 |
| novel.6094 | -1.32021 | 0.000389 | 0.005289 | Chr6 | 1.08E+08 | 1.08E+08 | 3335 |
| MsG0680035049.01 | -6.46311 | 0.00039 | 0.005307 | Chr6 | 98211506 | 98211799 | 294 |
| novel.6683 | 6.19856 | 0.000391 | 0.005313 | Chr6 | 1E+08 | 1E+08 | 384 |
| MsG0780041508.01 | 1.291861 | 0.000391 | 0.005316 | Chr7 | 91843751 | 91845448 | 975 |
| novel.2349 | 1.815754 | 0.000392 | 0.00532 | Chr3 | 45308580 | 45311480 | 1626 |
| novel.686 | -5.69167 | 0.000392 | 0.00532 | Chr1 | 10955351 | 10956143 | 793 |
| MsG0480023900.01 | -2.34682 | 0.000393 | 0.005331 | Chr4 | 91048766 | 91051957 | 1702 |
| MsG0880044387.01 | 1.713049 | 0.000393 | 0.005333 | Chr8 | 41175886 | 41176336 | 451 |
| MsG0280011193.01 | -7.12955 | 0.000395 | 0.005352 | Chr2 | 80806533 | 80808637 | 981 |
| MsG0880042984.01 | -6.27734 | 0.000396 | 0.005362 | Chr8 | 17053108 | 17055671 | 1191 |
| novel.1883 | -3.85241 | 0.000396 | 0.005367 | Chr2 | 39945805 | 39950936 | 703 |
| novel.4693 | 6.921917 | 0.000397 | 0.005369 | Chr5 | 76731977 | 76732449 | 473 |
| novel.3326 | -2.59523 | 0.000397 | 0.005376 | Chr4 | 19071502 | 19075941 | 889 |
| MsG0180001100.01 | -1.39858 | 0.000397 | 0.005376 | Chr1 | 15987120 | 15989464 | 729 |
| MsG0180001978.01 | -1.8601 | 0.000399 | 0.005394 | Chr1 | 30160317 | 30162865 | 636 |
| novel.8063 | -6.19278 | 0.000401 | 0.005423 | Chr8 | 52258265 | 52259091 | 827 |
| novel.7723 | 6.572063 | 0.000403 | 0.005438 | Chr7 | 86760953 | 86764603 | 769 |
| novel.1983 | 5.662959 | 0.000403 | 0.005442 | Chr2 | 66374235 | 66375099 | 865 |
| MsG0580024926.01 | -4.33381 | 0.000403 | 0.005442 | Chr5 | 11729218 | 11730345 | 1128 |
| MsG0680035227.01 | -5.99797 | 0.000404 | 0.005456 | Chr6 | 1.01E+08 | 1.01E+08 | 2226 |
| MsG0680030555.01 | -1.24616 | 0.000405 | 0.005459 | Chr6 | 5254447 | 5254958 | 512 |
| MsG0780037250.01 | 5.542126 | 0.000407 | 0.00548 | Chr7 | 22452779 | 22453165 | 387 |
| MsG0180004419.01 | 1.764796 | 0.000407 | 0.005485 | Chr1 | 77665816 | 77668979 | 998 |
| MsG0480023072.01 | -2.07501 | 0.000408 | 0.005496 | Chr4 | 80428514 | 80435726 | 2352 |
| novel.190 | 1.68404 | 0.000409 | 0.005507 | Chr1 | 26136797 | 26139937 | 1731 |
| MsG0580025610.01 | 1.712708 | 0.00041 | 0.005514 | Chr5 | 21212458 | 21227344 | 1075 |
| MsG0780036517.01 | -1.2143 | 0.000411 | 0.005533 | Chr7 | 9190092 | 9205933 | 3470 |
| novel.5601 | 2.419023 | 0.000412 | 0.00554 | Chr6 | 15954467 | 15957464 | 912 |
| MsG0780039510.01 | 1.899743 | 0.000414 | 0.005557 | Chr7 | 65054995 | 65055306 | 312 |
| novel.7570 | -2.26182 | 0.000417 | 0.005603 | Chr7 | 64948394 | 64954313 | 5920 |
| MsG0380017749.01 | 1.752642 | 0.000418 | 0.005607 | Chr3 | 1E+08 | 1E+08 | 1212 |
| MsG0180004059.01 | -5.66629 | 0.000418 | 0.005609 | Chr1 | 72640039 | 72640536 | 498 |
| novel.7359 | -7.01758 | 0.000418 | 0.005609 | Chr7 | 14973933 | 14975792 | 1811 |
| MsG0780037551.01 | 6.334646 | 0.000419 | 0.005611 | Chr7 | 28259172 | 28260440 | 1100 |
| novel.8918 | 6.219729 | 0.000419 | 0.005617 | contig289end | 17403 | 22782 | 399 |
| novel.2474 | -4.05794 | 0.00042 | 0.005619 | Chr3 | 74537975 | 74551477 | 1281 |
| MsG0680035154.01 | -2.25104 | 0.00042 | 0.005625 | Chr6 | 99798329 | 99807453 | 4122 |
| novel.2230 | 6.378743 | 0.00042 | 0.005625 | Chr3 | 17739007 | 17747678 | 368 |
| MsG0880042314.01 | 2.214802 | 0.000421 | 0.005632 | Chr8 | 6981302 | 6985567 | 2831 |
| novel.1779 | -6.11508 | 0.000422 | 0.005638 | Chr2 | 19054716 | 19063188 | 577 |
| MsG0580025569.01 | 2.013918 | 0.000422 | 0.00564 | Chr5 | 20639551 | 20644221 | 1509 |
| MsG0480020815.01 | 4.520215 | 0.000423 | 0.005651 | Chr4 | 48819268 | 48819717 | 450 |
| novel.8852 | 6.791043 | 0.000423 | 0.005651 | contig199end | 42434 | 44964 | 1122 |
| MsG0280006300.01 | 2.54887 | 0.000423 | 0.005651 | Chr2 | 194974 | 197210 | 531 |
| MsG0780039796.01 | 6.208685 | 0.000425 | 0.005664 | Chr7 | 69189151 | 69190260 | 1110 |
| novel.1160 | 6.21618 | 0.000426 | 0.005681 | Chr2 | 394734 | 398508 | 795 |
| MsG0880044282.01 | 4.940826 | 0.000428 | 0.005703 | Chr8 | 39386957 | 39388865 | 730 |
| novel.2714 | -6.06211 | 0.000428 | 0.005707 | Chr3 | 7776566 | 7781827 | 717 |
| novel.5971 | -2.6991 | 0.00043 | 0.005722 | Chr6 | 91416608 | 91418360 | 218 |
| novel.8550 | -3.36929 | 0.00043 | 0.005722 | Chr8 | 52045401 | 52046481 | 795 |
| MsG0380014983.01 | -1.83884 | 0.000431 | 0.00573 | Chr3 | 63563281 | 63566640 | 1686 |
| MsG0580029305.01 | 5.472765 | 0.000432 | 0.005741 | Chr5 | 95049694 | 95053674 | 994 |
| novel.3205 | -5.49645 | 0.000434 | 0.005764 | Chr3 | 1.02E+08 | 1.02E+08 | 694 |
| novel.7085 | 6.446275 | 0.000434 | 0.005769 | Chr7 | 65996986 | 66001219 | 972 |
| novel.630 | 5.534244 | 0.000435 | 0.005778 | Chr1 | 4202284 | 4203559 | 1276 |
| MsG0080047837.01 | 2.042034 | 0.000435 | 0.005778 | contig125end | 10644 | 16946 | 1132 |
| novel.3628 | -2.1752 | 0.000436 | 0.005791 | Chr4 | 77435350 | 77436864 | 1241 |
| MsG0680034881.01 | -1.98281 | 0.000437 | 0.005801 | Chr6 | 96190137 | 96190994 | 858 |
| novel.3351 | -4.23853 | 0.000442 | 0.005859 | Chr4 | 24523122 | 24524060 | 681 |
| MsG0380014679.01 | 2.825291 | 0.000442 | 0.005859 | Chr3 | 58335755 | 58335994 | 240 |
| MsG0380011660.01 | -1.85407 | 0.000445 | 0.005894 | Chr3 | 3128544 | 3134081 | 1632 |
| MsG0680035339.01 | -1.32823 | 0.000446 | 0.005903 | Chr6 | 1.03E+08 | 1.03E+08 | 1341 |
| MsG0580030060.01 | -1.58637 | 0.000448 | 0.005929 | Chr5 | 1.06E+08 | 1.06E+08 | 737 |
| MsG0380012561.01 | 2.710681 | 0.000449 | 0.00593 | Chr3 | 19811206 | 19814702 | 792 |
| MsG0080049010.01 | 3.165393 | 0.000449 | 0.00593 | contig605end | 10906 | 11764 | 699 |
| MsG0280011053.01 | -3.61547 | 0.00045 | 0.005946 | Chr2 | 78918380 | 78921416 | 806 |
| MsG0480022429.01 | 2.039057 | 0.000452 | 0.005971 | Chr4 | 72499073 | 72499858 | 786 |
| MsG0780036277.01 | -1.88657 | 0.000453 | 0.005985 | Chr7 | 5852683 | 5858925 | 3720 |
| MsG0080048000.01 | -1.12917 | 0.000455 | 0.005996 | contig165end | 8918 | 15265 | 3045 |
| novel.5743 | -5.54316 | 0.000455 | 0.005996 | Chr6 | 44019572 | 44020619 | 398 |
| novel.8247 | 1.413551 | 0.000457 | 0.006025 | Chr8 | 85106195 | 85111313 | 1114 |
| novel.7379 | -6.2857 | 0.000458 | 0.006041 | Chr7 | 17098419 | 17099604 | 638 |
| novel.6249 | -3.10817 | 0.000459 | 0.006047 | Chr6 | 16293193 | 16296964 | 3772 |
| MsG0380013179.01 | 2.840271 | 0.000461 | 0.006069 | Chr3 | 32143890 | 32150002 | 988 |
| MsG0180000784.01 | 2.400305 | 0.000463 | 0.006094 | Chr1 | 11132370 | 11132582 | 213 |
| novel.1298 | 5.954942 | 0.000463 | 0.006094 | Chr2 | 21617680 | 21619259 | 686 |
| MsG0080048214.01 | 5.928259 | 0.000463 | 0.006094 | contig245end | 6650 | 16143 | 336 |
| novel.9084 | -1.98596 | 0.000466 | 0.006131 | contig473end | 20664 | 23207 | 1254 |
| novel.747 | -3.32101 | 0.000468 | 0.006145 | Chr1 | 22232851 | 22235307 | 1916 |
| MsG0680030352.01 | 1.364277 | 0.000469 | 0.00616 | Chr6 | 975798 | 985457 | 750 |
| MsG0680031731.01 | 2.45042 | 0.00047 | 0.006163 | Chr6 | 25490860 | 25493984 | 528 |
| novel.621 | -5.50041 | 0.00047 | 0.00617 | Chr1 | 3447047 | 3447869 | 540 |
| MsG0280006503.01 | 1.125055 | 0.000471 | 0.006171 | Chr2 | 2837503 | 2844883 | 619 |
| novel.8163 | 2.961492 | 0.000471 | 0.006178 | Chr8 | 70570808 | 70572036 | 1229 |
| MsG0580029335.01 | 1.435033 | 0.000473 | 0.0062 | Chr5 | 95591981 | 95594574 | 739 |
| MsG0380016050.01 | 1.366012 | 0.000475 | 0.006215 | Chr3 | 78410174 | 78413932 | 674 |
| MsG0680030648.01 | 3.812903 | 0.000476 | 0.006228 | Chr6 | 6612783 | 6618921 | 1856 |
| MsG0180000888.01 | -4.22812 | 0.000476 | 0.006235 | Chr1 | 12781413 | 12782858 | 696 |
| MsG0480021876.01 | 1.180145 | 0.000478 | 0.006251 | Chr4 | 65465619 | 65470991 | 1320 |
| MsG0180004694.01 | 3.047126 | 0.000478 | 0.006253 | Chr1 | 81317490 | 81335163 | 3765 |
| MsG0580029312.01 | 3.091819 | 0.000479 | 0.006254 | Chr5 | 95159510 | 95164869 | 678 |
| novel.8116 | -1.09507 | 0.00048 | 0.006266 | Chr8 | 61669307 | 61673446 | 1925 |
| MsG0580027183.01 | 2.902361 | 0.00048 | 0.006266 | Chr5 | 55716606 | 55718773 | 558 |
| MsG0880044625.01 | 3.598103 | 0.000481 | 0.006277 | Chr8 | 45873550 | 45875299 | 1179 |
| MsG0280008344.01 | -2.09414 | 0.000483 | 0.0063 | Chr2 | 29734647 | 29748397 | 3281 |
| MsG0880046418.01 | -1.27539 | 0.000483 | 0.0063 | Chr8 | 73203908 | 73207169 | 1361 |
| MsG0380015404.01 | -1.4759 | 0.000485 | 0.00632 | Chr3 | 69653873 | 69654805 | 933 |
| MsG0580029539.01 | 1.407202 | 0.000486 | 0.006332 | Chr5 | 98282728 | 98296088 | 1635 |
| MsG0880045895.01 | 2.244246 | 0.000488 | 0.006351 | Chr8 | 66092648 | 66093040 | 393 |
| MsG0580025564.01 | 2.120731 | 0.000489 | 0.006364 | Chr5 | 20559663 | 20561676 | 602 |
| novel.2521 | -2.28825 | 0.000489 | 0.006364 | Chr3 | 82953130 | 82954023 | 894 |
| MsG0580024665.01 | 3.818895 | 0.000493 | 0.006406 | Chr5 | 7884403 | 7891287 | 1167 |
| novel.6070 | 6.222707 | 0.000493 | 0.006414 | Chr6 | 1.05E+08 | 1.05E+08 | 891 |
| MsG0480021792.01 | -3.63721 | 0.000494 | 0.006414 | Chr4 | 64323596 | 64325832 | 777 |
| novel.1721 | 6.131372 | 0.000497 | 0.00645 | Chr2 | 10297165 | 10297885 | 721 |
| MsG0180004953.01 | 1.475237 | 0.000497 | 0.006454 | Chr1 | 84910915 | 84913105 | 1187 |
| MsG0880045973.01 | -1.23262 | 0.000497 | 0.006454 | Chr8 | 67071692 | 67074790 | 1273 |
| MsG0480023246.01 | -2.5291 | 0.000499 | 0.006475 | Chr4 | 82612406 | 82612651 | 246 |
| MsG0780040837.01 | 6.483941 | 0.0005 | 0.00648 | Chr7 | 83208648 | 83210115 | 393 |
| MsG0380017847.01 | -5.95333 | 0.000501 | 0.006496 | Chr3 | 1.01E+08 | 1.01E+08 | 1227 |
| MsG0880044142.01 | 1.445138 | 0.000503 | 0.006511 | Chr8 | 36342970 | 36356942 | 3663 |
| MsG0880043364.01 | -1.73735 | 0.000504 | 0.00653 | Chr8 | 23678559 | 23685474 | 3191 |
| MsG0380016077.01 | 6.460162 | 0.000505 | 0.00654 | Chr3 | 78807258 | 78808508 | 1251 |
| MsG0380015537.01 | -4.01482 | 0.000507 | 0.006561 | Chr3 | 71558329 | 71559018 | 690 |
| MsG0280009977.01 | 6.656651 | 0.000508 | 0.006566 | Chr2 | 62217402 | 62217710 | 309 |
| MsG0780035963.01 | 6.150213 | 0.00051 | 0.00659 | Chr7 | 682760 | 683263 | 339 |
| MsG0880042242.01 | 3.478559 | 0.00051 | 0.006591 | Chr8 | 5969377 | 5976760 | 579 |
| novel.1507 | 2.373144 | 0.000513 | 0.006625 | Chr2 | 65916833 | 65919153 | 874 |
| novel.3742 | 4.911394 | 0.000514 | 0.006633 | Chr4 | 91936769 | 91938992 | 1147 |
| novel.3364 | 5.948021 | 0.000514 | 0.006634 | Chr4 | 25681988 | 25683951 | 629 |
| MsG0280010998.01 | -1.30619 | 0.000515 | 0.006643 | Chr2 | 78135663 | 78138694 | 1732 |
| MsG0580027983.01 | -2.418 | 0.000515 | 0.006645 | Chr5 | 72364313 | 72367251 | 2401 |
| MsG0580025404.01 | -3.82818 | 0.000516 | 0.006646 | Chr5 | 18133050 | 18133460 | 411 |
| novel.6868 | 4.22759 | 0.000519 | 0.006682 | Chr7 | 18444263 | 18446491 | 888 |
| novel.5830 | 6.301487 | 0.000519 | 0.006682 | Chr6 | 65579146 | 65582703 | 896 |
| MsG0780040405.01 | -2.0787 | 0.000521 | 0.006709 | Chr7 | 77052049 | 77056259 | 3045 |
| novel.8708 | -3.64881 | 0.000522 | 0.006709 | Chr8 | 79854505 | 79857507 | 2395 |
| MsG0680030990.01 | 2.888207 | 0.000522 | 0.006713 | Chr6 | 12599742 | 12609930 | 4668 |
| novel.7367 | -6.01317 | 0.000522 | 0.006715 | Chr7 | 15750315 | 15752580 | 1287 |
| MsG0680030867.01 | -1.98514 | 0.000524 | 0.006726 | Chr6 | 10670638 | 10672679 | 402 |
| novel.187 | 5.944451 | 0.000524 | 0.006729 | Chr1 | 25934784 | 25964374 | 1687 |
| novel.5278 | 6.515623 | 0.000525 | 0.006735 | Chr5 | 73804350 | 73805414 | 1065 |
| MsG0680034786.01 | -2.75927 | 0.000526 | 0.006743 | Chr6 | 94428972 | 94438962 | 2592 |
| MsG0580028529.01 | -3.00089 | 0.000526 | 0.006743 | Chr5 | 81888248 | 81889544 | 1212 |
| novel.3776 | -2.7878 | 0.000527 | 0.006758 | Chr4 | 4084778 | 4087624 | 526 |
| MsG0280006321.01 | 3.94449 | 0.000528 | 0.006761 | Chr2 | 431620 | 432225 | 606 |
| MsG0480024023.01 | -4.46579 | 0.000529 | 0.006772 | Chr4 | 92471492 | 92479869 | 1347 |
| MsG0580028958.01 | 1.477437 | 0.000529 | 0.006772 | Chr5 | 89581161 | 89582850 | 755 |
| MsG0580027097.01 | -1.69225 | 0.000529 | 0.006775 | Chr5 | 53031288 | 53035219 | 1684 |
| novel.680 | -3.0444 | 0.000531 | 0.006786 | Chr1 | 10265699 | 10268636 | 2740 |
| MsG0780041643.01 | -3.93801 | 0.000531 | 0.006786 | Chr7 | 93393543 | 93396092 | 450 |
| MsG0880043200.01 | 4.419545 | 0.000531 | 0.006786 | Chr8 | 20509322 | 20512347 | 1020 |
| novel.7852 | -1.72323 | 0.000531 | 0.006786 | Chr8 | 10908380 | 10913411 | 2945 |
| MsG0580028606.01 | 2.562097 | 0.000531 | 0.006786 | Chr5 | 83272291 | 83279210 | 550 |
| MsG0780039553.01 | -2.68003 | 0.000532 | 0.006794 | Chr7 | 65743535 | 65754371 | 1605 |
| novel.5958 | -6.06338 | 0.000534 | 0.006805 | Chr6 | 89750512 | 89751286 | 775 |
| novel.8698 | 6.024098 | 0.000534 | 0.006805 | Chr8 | 76813105 | 76814600 | 1403 |
| MsG0780040016.01 | -1.49521 | 0.000536 | 0.006837 | Chr7 | 72043327 | 72069096 | 5074 |
| MsG0280011347.01 | 2.245549 | 0.000539 | 0.006862 | Chr2 | 82775968 | 82777373 | 867 |
| MsG0480018925.01 | -2.41347 | 0.00054 | 0.006879 | Chr4 | 11850524 | 11854485 | 1308 |
| novel.620 | 1.958891 | 0.000542 | 0.006901 | Chr1 | 3436800 | 3443169 | 3017 |
| MsG0380016869.01 | -1.21431 | 0.000544 | 0.006925 | Chr3 | 88801886 | 88814650 | 2471 |
| MsG0780040472.01 | 2.130005 | 0.000545 | 0.006929 | Chr7 | 78087492 | 78089950 | 1078 |
| MsG0380012089.01 | -1.79024 | 0.000545 | 0.006933 | Chr3 | 10771908 | 10773084 | 573 |
| MsG0580028295.01 | -6.34287 | 0.000547 | 0.006948 | Chr5 | 77905542 | 77920063 | 1296 |
| MsG0880043265.01 | -3.33338 | 0.000547 | 0.006952 | Chr8 | 21731264 | 21739278 | 3855 |
| novel.139 | -5.96353 | 0.000548 | 0.006965 | Chr1 | 18231420 | 18232480 | 849 |
| MsG0080048702.01 | -2.37695 | 0.00055 | 0.006978 | contig405end | 11464 | 13157 | 1105 |
| MsG0480018519.01 | -2.35069 | 0.000555 | 0.007042 | Chr4 | 6078057 | 6078653 | 597 |
| novel.208 | -6.21813 | 0.000556 | 0.007045 | Chr1 | 30177827 | 30180521 | 2695 |
| novel.9176 | -2.32115 | 0.000556 | 0.007047 | contig530end | 5634 | 14244 | 2831 |
| novel.1651 | -4.1082 | 0.000556 | 0.007047 | Chr2 | 1629750 | 1638767 | 1376 |
| MsG0380016682.01 | -2.13908 | 0.000558 | 0.007074 | Chr3 | 86533861 | 86534693 | 657 |
| novel.437 | -1.71058 | 0.00056 | 0.007095 | Chr1 | 80626188 | 80631803 | 2324 |
| MsG0380013687.01 | 1.788559 | 0.000561 | 0.007096 | Chr3 | 43572214 | 43574566 | 1932 |
| MsG0380017846.01 | -5.96695 | 0.000561 | 0.007097 | Chr3 | 1.01E+08 | 1.01E+08 | 948 |
| MsG0280011368.01 | -3.48875 | 0.000562 | 0.00711 | Chr2 | 83093582 | 83097917 | 2013 |
| MsG0880042634.01 | -1.98891 | 0.000563 | 0.00711 | Chr8 | 11745118 | 11748939 | 282 |
| MsG0380012312.01 | -7.48166 | 0.000563 | 0.007119 | Chr3 | 14717168 | 14722996 | 4350 |
| MsG0480019045.01 | 2.270104 | 0.000564 | 0.007119 | Chr4 | 13746806 | 13748132 | 1059 |
| MsG0280010637.01 | -2.93362 | 0.000571 | 0.007208 | Chr2 | 72663375 | 72668638 | 660 |
| MsG0680035778.01 | -2.15273 | 0.000572 | 0.007211 | Chr6 | 1.12E+08 | 1.12E+08 | 2991 |
| MsG0180001969.01 | -2.71357 | 0.000572 | 0.007214 | Chr1 | 30058880 | 30061930 | 3051 |
| novel.1919 | 5.885678 | 0.000577 | 0.007274 | Chr2 | 53034916 | 53040906 | 364 |
| MsG0580028789.01 | -3.07733 | 0.000578 | 0.007288 | Chr5 | 86483109 | 86486860 | 2913 |
| MsG0780040871.01 | -1.77585 | 0.000579 | 0.007288 | Chr7 | 83695577 | 83698825 | 1838 |
| MsG0080048348.01 | -3.82862 | 0.000582 | 0.007325 | contig27end | 35727 | 40369 | 924 |
| MsG0880042771.01 | -1.65068 | 0.000583 | 0.007341 | Chr8 | 13822343 | 13826776 | 1017 |
| MsG0180000562.01 | -2.62206 | 0.000584 | 0.007347 | Chr1 | 7906140 | 7916374 | 3819 |
| MsG0180003996.01 | -1.40853 | 0.000588 | 0.00739 | Chr1 | 71533158 | 71544775 | 2431 |
| MsG0280010681.01 | 2.783876 | 0.000588 | 0.007396 | Chr2 | 73269846 | 73277339 | 331 |
| novel.8261 | -1.42222 | 0.000589 | 0.007406 | Chr8 | 86896264 | 86900354 | 2344 |
| novel.4366 | -1.90368 | 0.00059 | 0.007407 | Chr5 | 12528668 | 12530917 | 1669 |
| MsG0380017079.01 | -3.39373 | 0.00059 | 0.007411 | Chr3 | 91704377 | 91705693 | 1317 |
| MsG0680034495.01 | -1.78483 | 0.000591 | 0.007414 | Chr6 | 88542659 | 88549825 | 2202 |
| novel.7898 | 1.178998 | 0.000593 | 0.007437 | Chr8 | 19437180 | 19442089 | 1990 |
| novel.6841 | -4.45123 | 0.000596 | 0.007471 | Chr7 | 13308331 | 13311910 | 1173 |
| MsG0580025152.01 | 1.82186 | 0.000597 | 0.007484 | Chr5 | 14496998 | 14499354 | 1130 |
| MsG0580024218.01 | -5.18756 | 0.000597 | 0.007484 | Chr5 | 2153360 | 2153967 | 399 |
| novel.7544 | 3.393379 | 0.0006 | 0.007516 | Chr7 | 60350133 | 60352194 | 1917 |
| MsG0180000201.01 | 3.088741 | 0.000605 | 0.007568 | Chr1 | 2760862 | 2761116 | 255 |
| MsG0180004333.01 | 2.552496 | 0.000609 | 0.007617 | Chr1 | 76521687 | 76524944 | 1190 |
| novel.222 | 6.314389 | 0.00061 | 0.007626 | Chr1 | 34515624 | 34517076 | 767 |
| MsG0380011882.01 | -2.75646 | 0.00061 | 0.007628 | Chr3 | 7325444 | 7332682 | 1353 |
| MsG0580025490.01 | -1.38496 | 0.000611 | 0.00763 | Chr5 | 19560728 | 19564828 | 2271 |
| novel.4927 | -2.57705 | 0.000611 | 0.007636 | Chr5 | 3932352 | 3934283 | 1129 |
| MsG0680035762.01 | 2.088995 | 0.000612 | 0.007636 | Chr6 | 1.12E+08 | 1.12E+08 | 351 |
| novel.5782 | 1.646134 | 0.000612 | 0.007643 | Chr6 | 51656859 | 51658001 | 699 |
| MsG0380013913.01 | -1.53305 | 0.000614 | 0.007659 | Chr3 | 48232892 | 48238758 | 1556 |
| novel.2005 | -2.03987 | 0.000615 | 0.007675 | Chr2 | 69732756 | 69736556 | 1733 |
| MsG0480018962.01 | 1.342564 | 0.000616 | 0.007679 | Chr4 | 12529520 | 12535027 | 1415 |
| MsG0780039837.01 | -1.72845 | 0.000617 | 0.007686 | Chr7 | 69592943 | 69595600 | 1343 |
| MsG0780036010.01 | -5.77417 | 0.000617 | 0.007689 | Chr7 | 1295611 | 1300839 | 1206 |
| MsG0280010989.01 | 2.615553 | 0.000619 | 0.007704 | Chr2 | 78047794 | 78049557 | 1764 |
| MsG0780037714.01 | 6.746967 | 0.00062 | 0.007709 | Chr7 | 31826453 | 31837592 | 2535 |
| MsG0580025447.01 | -1.24252 | 0.000621 | 0.007727 | Chr5 | 18744511 | 18747419 | 1044 |
| MsG0180000273.01 | -1.4414 | 0.000621 | 0.007727 | Chr1 | 3619093 | 3626196 | 2018 |
| novel.1945 | 1.614481 | 0.000622 | 0.007736 | Chr2 | 59103359 | 59109755 | 1897 |
| novel.8350 | 5.964228 | 0.000625 | 0.007759 | Chr8 | 9724595 | 9725258 | 664 |
| MsG0880042276.01 | -2.44303 | 0.000625 | 0.007764 | Chr8 | 6405519 | 6410196 | 1246 |
| MsG0380015746.01 | -2.07642 | 0.000626 | 0.007764 | Chr3 | 74525287 | 74529682 | 2817 |
| MsG0780037463.01 | 5.34066 | 0.000626 | 0.007765 | Chr7 | 26399529 | 26400479 | 519 |
| MsG0280010438.01 | -1.69511 | 0.000626 | 0.007768 | Chr2 | 69600081 | 69602503 | 1351 |
| novel.3734 | 1.973486 | 0.000629 | 0.007792 | Chr4 | 90585526 | 90587224 | 739 |
| MsG0580024787.01 | 3.009729 | 0.00063 | 0.007799 | Chr5 | 9695648 | 9698183 | 1366 |
| MsG0580029385.01 | 1.567083 | 0.00063 | 0.007799 | Chr5 | 96244333 | 96244713 | 381 |
| novel.4551 | -4.87388 | 0.00063 | 0.007799 | Chr5 | 47123561 | 47140044 | 456 |
| MsG0380016127.01 | -3.0469 | 0.000631 | 0.007807 | Chr3 | 79410715 | 79411419 | 705 |
| novel.9283 | -3.01761 | 0.000633 | 0.007828 | contig592end | 22804 | 24413 | 1266 |
| MsG0380013831.01 | -2.00395 | 0.000633 | 0.00783 | Chr3 | 46281908 | 46284214 | 2307 |
| novel.365 | 6.343596 | 0.000634 | 0.007841 | Chr1 | 66157865 | 66159681 | 930 |
| MsG0480022129.01 | 2.007101 | 0.000636 | 0.007854 | Chr4 | 68609334 | 68611611 | 946 |
| MsG0780036621.01 | 6.060893 | 0.000638 | 0.007882 | Chr7 | 10794062 | 10794661 | 600 |
| MsG0180005790.01 | -1.63876 | 0.000639 | 0.007889 | Chr1 | 96166817 | 96175922 | 3061 |
| MsG0280010676.01 | -1.75859 | 0.000641 | 0.007912 | Chr2 | 73206697 | 73206969 | 273 |
| MsG0480020829.01 | 1.973812 | 0.000641 | 0.007912 | Chr4 | 49252746 | 49253336 | 306 |
| novel.3507 | -3.37065 | 0.000643 | 0.007932 | Chr4 | 57492127 | 57497195 | 977 |
| MsG0180005911.01 | -3.15053 | 0.000645 | 0.007951 | Chr1 | 97456031 | 97457640 | 275 |
| MsG0480022940.01 | 4.435084 | 0.000645 | 0.007951 | Chr4 | 78834191 | 78839010 | 2484 |
| novel.4324 | 1.901393 | 0.000646 | 0.007957 | Chr5 | 6453389 | 6454890 | 1502 |
| novel.7335 | -1.84289 | 0.000646 | 0.007957 | Chr7 | 10554863 | 10564431 | 2582 |
| MsG0280010237.01 | -1.06531 | 0.000647 | 0.007964 | Chr2 | 66499617 | 66513910 | 1872 |
| MsG0480022493.01 | 1.140706 | 0.000649 | 0.007976 | Chr4 | 73203674 | 73205978 | 626 |
| MsG0780036210.01 | -2.44398 | 0.000649 | 0.007977 | Chr7 | 4740830 | 4748548 | 1925 |
| novel.5916 | -1.20613 | 0.000651 | 0.008001 | Chr6 | 81905251 | 81914608 | 1047 |
| MsG0280011465.01 | -1.5852 | 0.000651 | 0.008003 | Chr2 | 84380731 | 84384444 | 1200 |
| MsG0780037190.01 | 1.477454 | 0.000652 | 0.008004 | Chr7 | 21503688 | 21505735 | 1600 |
| MsG0880047695.01 | -1.52102 | 0.000657 | 0.008058 | Chr8 | 89687311 | 89689767 | 644 |
| MsG0280007962.01 | -5.89846 | 0.000657 | 0.00806 | Chr2 | 23770886 | 23772058 | 1173 |
| MsG0080048816.01 | 1.58954 | 0.000659 | 0.008077 | contig462end | 5946 | 6296 | 351 |
| MsG0080048057.01 | -2.47674 | 0.000659 | 0.008077 | contig184end | 26156 | 26776 | 489 |
| MsG0880042712.01 | -1.85121 | 0.000659 | 0.008077 | Chr8 | 13027823 | 13030209 | 894 |
| MsG0480022809.01 | 1.183649 | 0.000663 | 0.008119 | Chr4 | 76945144 | 76947575 | 2355 |
| MsG0180001128.01 | -1.97966 | 0.000664 | 0.008122 | Chr1 | 16348234 | 16349011 | 483 |
| MsG0180003695.01 | 1.80719 | 0.000664 | 0.008122 | Chr1 | 66781688 | 66784267 | 1955 |
| MsG0680034060.01 | 6.365293 | 0.000665 | 0.008128 | Chr6 | 79820796 | 79823156 | 447 |
| MsG0180001917.01 | 2.608215 | 0.000665 | 0.008128 | Chr1 | 29327275 | 29328827 | 735 |
| MsG0780039506.01 | 3.290516 | 0.000666 | 0.008143 | Chr7 | 65016864 | 65017422 | 462 |
| novel.5493 | -5.989 | 0.000668 | 0.008163 | Chr5 | 1.08E+08 | 1.08E+08 | 674 |
| MsG0580024050.01 | -1.5704 | 0.000669 | 0.008169 | Chr5 | 274834 | 285780 | 3487 |
| MsG0580027882.01 | 3.71747 | 0.00067 | 0.008174 | Chr5 | 70272690 | 70275351 | 606 |
| MsG0180001876.01 | -5.50444 | 0.00067 | 0.008179 | Chr1 | 28617654 | 28618061 | 408 |
| novel.3123 | 1.644771 | 0.000676 | 0.008242 | Chr3 | 89801980 | 89809191 | 5316 |
| MsG0380015339.01 | -1.322 | 0.000676 | 0.008247 | Chr3 | 68661232 | 68666401 | 2662 |
| MsG0280009011.01 | -2.19304 | 0.00068 | 0.008293 | Chr2 | 43436889 | 43452263 | 2127 |
| MsG0780036881.01 | 1.138686 | 0.000681 | 0.008301 | Chr7 | 15611893 | 15617488 | 1819 |
| MsG0480018207.01 | 1.585888 | 0.000684 | 0.008327 | Chr4 | 1558635 | 1562459 | 604 |
| MsG0280010039.01 | 1.391724 | 0.000685 | 0.00834 | Chr2 | 63360707 | 63364439 | 794 |
| MsG0680034028.01 | -2.37451 | 0.000686 | 0.008353 | Chr6 | 78907436 | 78927971 | 2151 |
| MsG0680030608.01 | 1.262643 | 0.000688 | 0.008368 | Chr6 | 6016630 | 6019286 | 982 |
| novel.3785 | 6.240622 | 0.000689 | 0.008377 | Chr4 | 5315060 | 5316898 | 1299 |
| MsG0680033607.01 | -6.2958 | 0.00069 | 0.008381 | Chr6 | 69425686 | 69426969 | 1284 |
| novel.1845 | 4.964091 | 0.000697 | 0.008468 | Chr2 | 32262664 | 32263143 | 315 |
| MsG0280006996.01 | 1.320794 | 0.000699 | 0.008486 | Chr2 | 9202548 | 9209731 | 2201 |
| MsG0380011528.01 | 6.148095 | 0.000699 | 0.008487 | Chr3 | 970317 | 974129 | 1410 |
| MsG0180001836.01 | -5.98672 | 0.0007 | 0.008495 | Chr1 | 27972000 | 27973324 | 630 |
| MsG0380016083.01 | -5.86672 | 0.000701 | 0.008504 | Chr3 | 78909736 | 78909945 | 210 |
| MsG0880043214.01 | -3.25229 | 0.000702 | 0.008507 | Chr8 | 20729526 | 20730660 | 540 |
| MsG0380012860.01 | 1.353505 | 0.000702 | 0.008509 | Chr3 | 25736154 | 25739728 | 1479 |
| novel.2154 | 6.295645 | 0.000704 | 0.008533 | Chr3 | 5919587 | 5922029 | 682 |
| MsG0380015917.01 | -5.31057 | 0.000707 | 0.008556 | Chr3 | 76602151 | 76604811 | 816 |
| MsG0380013481.01 | 1.684714 | 0.000707 | 0.008557 | Chr3 | 39084237 | 39087593 | 1127 |
| MsG0580027103.01 | 1.356093 | 0.000709 | 0.008575 | Chr5 | 53226438 | 53240057 | 1356 |
| MsG0180005041.01 | -2.03955 | 0.000711 | 0.008594 | Chr1 | 86113899 | 86120278 | 303 |
| MsG0780040738.01 | -3.24802 | 0.000711 | 0.008594 | Chr7 | 81809331 | 81810941 | 1611 |
| MsG0580027408.01 | -1.53892 | 0.000713 | 0.008607 | Chr5 | 59989155 | 59990111 | 957 |
| novel.819 | 6.09622 | 0.000713 | 0.008607 | Chr1 | 36158342 | 36159355 | 825 |
| MsG0580029754.01 | 1.123195 | 0.000713 | 0.008609 | Chr5 | 1.02E+08 | 1.02E+08 | 798 |
| novel.5194 | -3.1357 | 0.000714 | 0.008615 | Chr5 | 58158027 | 58162587 | 3114 |
| novel.5783 | -5.94158 | 0.000715 | 0.00862 | Chr6 | 51842454 | 51843256 | 777 |
| novel.8059 | -4.36029 | 0.000715 | 0.008626 | Chr8 | 52024313 | 52025769 | 559 |
| MsG0380012253.01 | -2.98375 | 0.000718 | 0.008655 | Chr3 | 13371906 | 13380029 | 3981 |
| MsG0380014453.01 | -2.50289 | 0.000718 | 0.008656 | Chr3 | 55039137 | 55039673 | 537 |
| MsG0380017703.01 | 1.759599 | 0.000719 | 0.008656 | Chr3 | 99768002 | 99768316 | 315 |
| novel.5753 | -1.66966 | 0.000719 | 0.008659 | Chr6 | 46304344 | 46305769 | 1131 |
| MsG0180005366.01 | 1.438194 | 0.00072 | 0.008668 | Chr1 | 90873534 | 90875318 | 748 |
| novel.1288 | 2.486855 | 0.000721 | 0.008668 | Chr2 | 19602151 | 19606826 | 1678 |
| novel.3811 | 6.236424 | 0.000721 | 0.008668 | Chr4 | 11580626 | 11582007 | 819 |
| novel.2373 | -5.86424 | 0.000722 | 0.008677 | Chr3 | 52831063 | 52833113 | 397 |
| MsG0180001538.01 | 2.265657 | 0.000722 | 0.008677 | Chr1 | 22931130 | 22934002 | 1200 |
| MsG0780041509.01 | 2.064328 | 0.000725 | 0.008711 | Chr7 | 91848471 | 91853290 | 1248 |
| novel.2129 | 5.903826 | 0.000729 | 0.008746 | Chr3 | 1468512 | 1468917 | 406 |
| MsG0680035382.01 | -3.13047 | 0.000729 | 0.00875 | Chr6 | 1.04E+08 | 1.04E+08 | 781 |
| MsG0180001475.01 | -2.54016 | 0.000732 | 0.008785 | Chr1 | 21952860 | 21954506 | 1647 |
| MsG0680030771.01 | 1.85372 | 0.000734 | 0.008802 | Chr6 | 8895118 | 8904440 | 2849 |
| novel.72 | 6.067672 | 0.000735 | 0.008813 | Chr1 | 9493816 | 9495581 | 457 |
| novel.5151 | -2.63166 | 0.000739 | 0.008852 | Chr5 | 48087209 | 48097500 | 7846 |
| novel.9171 | 1.238235 | 0.000741 | 0.00887 | contig528end | 16429 | 24119 | 2006 |
| MsG0180000044.01 | 4.015241 | 0.000741 | 0.008875 | Chr1 | 723031 | 728584 | 2553 |
| novel.1660 | 2.191968 | 0.000742 | 0.008879 | Chr2 | 2185567 | 2187651 | 2085 |
| MsG0880043897.01 | 6.345303 | 0.000743 | 0.00889 | Chr8 | 32320156 | 32320629 | 474 |
| MsG0580028987.01 | 6.228965 | 0.000744 | 0.00889 | Chr5 | 89952725 | 89954888 | 801 |
| MsG0480019417.01 | 1.313352 | 0.000744 | 0.008894 | Chr4 | 19935485 | 19945677 | 844 |
| MsG0380017516.01 | 1.249933 | 0.000746 | 0.008911 | Chr3 | 97296110 | 97298777 | 1355 |
| MsG0880042043.01 | 1.862907 | 0.000746 | 0.008914 | Chr8 | 3056227 | 3061048 | 1503 |
| MsG0880046599.01 | 4.65413 | 0.000747 | 0.008914 | Chr8 | 75329580 | 75331388 | 501 |
| MsG0680030285.01 | 1.529482 | 0.000749 | 0.00894 | Chr6 | 33851 | 42934 | 998 |
| MsG0680030777.01 | 3.590318 | 0.000749 | 0.00894 | Chr6 | 9065660 | 9072345 | 1047 |
| MsG0480022398.01 | -2.07791 | 0.00075 | 0.008945 | Chr4 | 72035706 | 72038570 | 393 |
| MsG0780041426.01 | 1.802173 | 0.000754 | 0.008984 | Chr7 | 90745151 | 90746632 | 831 |
| MsG0480023920.01 | -1.68459 | 0.000754 | 0.008986 | Chr4 | 91265911 | 91268567 | 964 |
| MsG0380011504.01 | 2.762238 | 0.000755 | 0.008986 | Chr3 | 611425 | 617141 | 2597 |
| MsG0180000242.01 | 1.272165 | 0.000758 | 0.009028 | Chr1 | 3299549 | 3308842 | 1462 |
| MsG0880045503.01 | 2.557938 | 0.000762 | 0.009065 | Chr8 | 60174639 | 60178205 | 1572 |
| novel.3743 | 5.14629 | 0.000764 | 0.00909 | Chr4 | 92219994 | 92221713 | 1279 |
| novel.1610 | 3.212119 | 0.00077 | 0.009154 | Chr2 | 80579468 | 80583110 | 871 |
| MsG0580025356.01 | -3.28622 | 0.000771 | 0.009159 | Chr5 | 17388799 | 17389572 | 774 |
| MsG0580027343.01 | 1.576802 | 0.000771 | 0.009159 | Chr5 | 58763847 | 58768232 | 1514 |
| MsG0580028492.01 | -1.64662 | 0.000771 | 0.009159 | Chr5 | 81385081 | 81387154 | 1386 |
| MsG0480018211.01 | -1.97419 | 0.000774 | 0.009189 | Chr4 | 1659613 | 1661624 | 513 |
| MsG0480021094.01 | 1.493232 | 0.000775 | 0.009198 | Chr4 | 53358694 | 53362722 | 1627 |
| MsG0280009645.01 | -3.88268 | 0.000777 | 0.009211 | Chr2 | 55753793 | 55760589 | 2026 |
| novel.5694 | -2.17196 | 0.000777 | 0.009213 | Chr6 | 30653540 | 30654754 | 1215 |
| MsG0180000930.01 | -2.39551 | 0.000777 | 0.009213 | Chr1 | 13361767 | 13366310 | 2697 |
| MsG0280010245.01 | 2.203565 | 0.000783 | 0.009271 | Chr2 | 66629993 | 66633112 | 969 |
| MsG0480018531.01 | -5.87689 | 0.000786 | 0.009306 | Chr4 | 6210489 | 6220952 | 5786 |
| novel.5706 | 6.196338 | 0.000786 | 0.009306 | Chr6 | 33520412 | 33521375 | 937 |
| MsG0480021657.01 | 5.849748 | 0.000786 | 0.009306 | Chr4 | 62241604 | 62241858 | 255 |
| MsG0680032013.01 | 1.534802 | 0.000787 | 0.00931 | Chr6 | 31934476 | 31937578 | 988 |
| MsG0680030354.01 | -1.96341 | 0.000788 | 0.009312 | Chr6 | 1009056 | 1014144 | 2866 |
| MsG0780037919.01 | 3.865396 | 0.000789 | 0.009322 | Chr7 | 36372001 | 36374627 | 483 |
| MsG0780036873.01 | -4.26285 | 0.000791 | 0.009348 | Chr7 | 15450699 | 15454404 | 2412 |
| MsG0380014313.01 | -2.97438 | 0.000792 | 0.009348 | Chr3 | 52818801 | 52830939 | 4323 |
| MsG0580028316.01 | -1.7097 | 0.000792 | 0.009354 | Chr5 | 78243669 | 78249445 | 999 |
| MsG0880046225.01 | -1.77356 | 0.000796 | 0.009391 | Chr8 | 70771261 | 70776814 | 2038 |
| novel.3464 | 3.054778 | 0.000797 | 0.009403 | Chr4 | 48886974 | 48894890 | 530 |
| MsG0880042843.01 | -1.68327 | 0.000807 | 0.009509 | Chr8 | 14775641 | 14776033 | 393 |
| MsG0580025570.01 | 1.34846 | 0.00081 | 0.009546 | Chr5 | 20669792 | 20678426 | 3898 |
| MsG0880046686.01 | 6.061425 | 0.00081 | 0.009546 | Chr8 | 76765061 | 76771761 | 1814 |
| MsG0780039963.01 | -2.0264 | 0.000811 | 0.009546 | Chr7 | 71155766 | 71165757 | 3678 |
| MsG0480022252.01 | 1.72969 | 0.000811 | 0.009546 | Chr4 | 70119887 | 70123935 | 921 |
| MsG0680031054.01 | 6.75135 | 0.000812 | 0.009553 | Chr6 | 13377314 | 13381112 | 1326 |
| novel.4516 | 5.831307 | 0.000813 | 0.009563 | Chr5 | 41193855 | 41195745 | 1614 |
| novel.2885 | -3.08896 | 0.000814 | 0.009566 | Chr3 | 45112772 | 45113688 | 917 |
| MsG0880046751.01 | 2.682059 | 0.000814 | 0.009566 | Chr8 | 77653899 | 77654885 | 987 |
| MsG0380014144.01 | 1.398295 | 0.000815 | 0.009568 | Chr3 | 50332952 | 50336084 | 2265 |
| MsG0680031859.01 | 5.991563 | 0.000815 | 0.009568 | Chr6 | 28342361 | 28342852 | 492 |
| MsG0680032357.01 | 2.849029 | 0.000815 | 0.009568 | Chr6 | 39231463 | 39234531 | 675 |
| MsG0180005272.01 | -1.42835 | 0.000822 | 0.009644 | Chr1 | 89437370 | 89441569 | 1925 |
| MsG0780036424.01 | 6.115201 | 0.000824 | 0.009667 | Chr7 | 7956106 | 7958535 | 1310 |
| MsG0380016063.01 | -1.4582 | 0.000824 | 0.009667 | Chr3 | 78593347 | 78598513 | 1473 |
| MsG0380014778.01 | -1.7044 | 0.000827 | 0.009689 | Chr3 | 59837600 | 59844058 | 652 |
| MsG0180001138.01 | 1.539132 | 0.000828 | 0.009704 | Chr1 | 16443266 | 16448339 | 2159 |
| MsG0380015158.01 | -5.92071 | 0.000831 | 0.009731 | Chr3 | 65965869 | 65966432 | 564 |
| MsG0680030437.01 | 4.836322 | 0.000831 | 0.009731 | Chr6 | 2796937 | 2797875 | 939 |
| MsG0680035493.01 | -2.09772 | 0.000834 | 0.00976 | Chr6 | 1.07E+08 | 1.07E+08 | 1797 |
| novel.5168 | -2.44875 | 0.000834 | 0.00976 | Chr5 | 52897098 | 52901520 | 1139 |
| MsG0880047199.01 | 1.338893 | 0.000835 | 0.009764 | Chr8 | 83209591 | 83211303 | 773 |
| MsG0380017214.01 | 3.604824 | 0.000838 | 0.009795 | Chr3 | 93393210 | 93398810 | 454 |
| MsG0280008779.01 | -2.0751 | 0.000839 | 0.009804 | Chr2 | 38419925 | 38423047 | 3123 |
| novel.7096 | 4.83032 | 0.000841 | 0.009822 | Chr7 | 67507929 | 67508257 | 329 |
| MsG0880042087.01 | -4.53894 | 0.000841 | 0.009822 | Chr8 | 3533959 | 3535560 | 504 |
| MsG0180003084.01 | -5.9236 | 0.000842 | 0.009825 | Chr1 | 56361361 | 56369941 | 768 |
| MsG0780040840.01 | -4.6455 | 0.000843 | 0.009839 | Chr7 | 83228667 | 83235506 | 3066 |
| MsG0580030148.01 | 6.173784 | 0.000845 | 0.009855 | Chr5 | 1.07E+08 | 1.07E+08 | 546 |
| MsG0780041475.01 | -2.46962 | 0.000845 | 0.009855 | Chr7 | 91380097 | 91380495 | 399 |
| MsG0580029576.01 | 1.64176 | 0.000846 | 0.009856 | Chr5 | 98802499 | 98807205 | 1443 |
| MsG0480018773.01 | -2.44347 | 0.000847 | 0.009856 | Chr4 | 9621131 | 9630283 | 3708 |
| novel.3278 | 1.891457 | 0.000847 | 0.009856 | Chr4 | 10459779 | 10463764 | 1861 |
| MsG0880042086.01 | -1.60914 | 0.000854 | 0.009943 | Chr8 | 3530983 | 3532299 | 1317 |
| novel.74 | -5.82569 | 0.000855 | 0.009945 | Chr1 | 9778232 | 9779474 | 811 |
| novel.2263 | 6.12777 | 0.000856 | 0.009949 | Chr3 | 22528770 | 22530975 | 1650 |
| novel.293 | -4.64668 | 0.000856 | 0.00995 | Chr1 | 55232741 | 55235283 | 1553 |
| MsG0680034101.01 | -2.56175 | 0.000861 | 0.010007 | Chr6 | 80518352 | 80519605 | 1254 |
| MsG0680034483.01 | 5.555659 | 0.000862 | 0.010016 | Chr6 | 88368037 | 88369503 | 694 |
| MsG0180004609.01 | 1.742808 | 0.000863 | 0.01002 | Chr1 | 80299533 | 80302028 | 923 |
| novel.8032 | 3.727875 | 0.000865 | 0.010034 | Chr8 | 47725827 | 47730642 | 598 |
| MsG0780036567.01 | -2.98446 | 0.000865 | 0.010034 | Chr7 | 9908468 | 9909058 | 312 |
| MsG0380014710.01 | 2.647835 | 0.000865 | 0.010034 | Chr3 | 58777865 | 58781970 | 2026 |
| novel.4511 | -3.34509 | 0.000866 | 0.010035 | Chr5 | 38009773 | 38014243 | 1662 |
| novel.6704 | -5.86855 | 0.000866 | 0.010035 | Chr6 | 1.03E+08 | 1.03E+08 | 780 |
| novel.3795 | 2.679522 | 0.000868 | 0.010047 | Chr4 | 6673978 | 6674937 | 730 |
| novel.1198 | 3.249276 | 0.000868 | 0.010047 | Chr2 | 5232650 | 5237825 | 1652 |
| novel.7498 | -1.49051 | 0.000868 | 0.010049 | Chr7 | 47509172 | 47515284 | 4013 |
| MsG0880042610.01 | -5.83173 | 0.000869 | 0.01005 | Chr8 | 11440902 | 11448381 | 4542 |
| MsG0480022050.01 | 1.978356 | 0.000869 | 0.010055 | Chr4 | 67399925 | 67417082 | 3129 |
| MsG0780040428.01 | 1.686696 | 0.000871 | 0.010069 | Chr7 | 77393567 | 77398057 | 1560 |
| MsG0480021765.01 | -1.29332 | 0.000873 | 0.01009 | Chr4 | 63999849 | 64010098 | 4288 |
| novel.8513 | -3.06287 | 0.000874 | 0.010097 | Chr8 | 44743657 | 44746537 | 2030 |
| MsG0180004193.01 | -2.09488 | 0.000875 | 0.010106 | Chr1 | 74700645 | 74702533 | 1544 |
| MsG0180005137.01 | 2.026545 | 0.000876 | 0.010114 | Chr1 | 87469586 | 87472335 | 801 |
| MsG0280006550.01 | -2.85837 | 0.000877 | 0.010114 | Chr2 | 3359484 | 3359972 | 489 |
| MsG0880042656.01 | -2.07916 | 0.000879 | 0.010139 | Chr8 | 12027180 | 12029395 | 1509 |
| novel.6388 | 2.425009 | 0.000881 | 0.010153 | Chr6 | 38561616 | 38564662 | 1539 |
| MsG0380017944.01 | 3.96228 | 0.000882 | 0.010161 | Chr3 | 1.03E+08 | 1.03E+08 | 1073 |
| MsG0880043601.01 | -1.47305 | 0.000882 | 0.010161 | Chr8 | 27579595 | 27597994 | 4535 |
| MsG0380015908.01 | 2.72559 | 0.000882 | 0.010161 | Chr3 | 76528563 | 76529159 | 597 |
| MsG0480020804.01 | -1.31147 | 0.000883 | 0.010166 | Chr4 | 48631111 | 48651069 | 2895 |
| novel.2756 | 3.294715 | 0.000884 | 0.010167 | Chr3 | 12430826 | 12434319 | 2043 |
| novel.8334 | 1.445686 | 0.000884 | 0.010172 | Chr8 | 7113630 | 7114580 | 576 |
| MsG0480023799.01 | -3.7531 | 0.000885 | 0.010172 | Chr4 | 89939615 | 89941283 | 1125 |
| novel.5969 | 2.103008 | 0.00089 | 0.010225 | Chr6 | 91179642 | 91184729 | 1923 |
| novel.6950 | 1.898455 | 0.000894 | 0.010268 | Chr7 | 34387026 | 34402970 | 2641 |
| novel.6088 | 1.77474 | 0.000899 | 0.010322 | Chr6 | 1.08E+08 | 1.08E+08 | 4839 |
| MsG0480019217.01 | -7.05562 | 0.000899 | 0.010322 | Chr4 | 16317192 | 16320811 | 1963 |
| MsG0480020675.01 | 2.116273 | 0.000899 | 0.010322 | Chr4 | 45881057 | 45886598 | 323 |
| MsG0180005754.01 | -2.07814 | 0.0009 | 0.010329 | Chr1 | 95703079 | 95718483 | 3888 |
| novel.5214 | 3.391189 | 0.000905 | 0.010381 | Chr5 | 62812212 | 62819704 | 678 |
| novel.1395 | -5.44974 | 0.000911 | 0.010443 | Chr2 | 38313935 | 38316726 | 680 |
| novel.2280 | 5.827545 | 0.000912 | 0.010443 | Chr3 | 26864087 | 26865284 | 1198 |
| MsG0580024443.01 | 2.929041 | 0.000912 | 0.010443 | Chr5 | 5059428 | 5061696 | 1112 |
| MsG0080048367.01 | 2.766386 | 0.000916 | 0.010492 | contig288end | 4682 | 10239 | 1768 |
| MsG0480023068.01 | -4.13882 | 0.000917 | 0.010497 | Chr4 | 80381028 | 80381207 | 180 |
| MsG0580025174.01 | 5.919468 | 0.000918 | 0.010501 | Chr5 | 14747223 | 14748095 | 873 |
| MsG0580025224.01 | -3.39652 | 0.000923 | 0.010551 | Chr5 | 15464852 | 15467503 | 1332 |
| novel.4837 | 6.36961 | 0.000923 | 0.010556 | Chr5 | 99838035 | 99840468 | 769 |
| novel.5098 | -3.96619 | 0.000924 | 0.010563 | Chr5 | 37681601 | 37682715 | 957 |
| MsG0880045410.01 | -5.83337 | 0.000925 | 0.010564 | Chr8 | 58860832 | 58861404 | 573 |
| MsG0580024531.01 | 2.007822 | 0.000925 | 0.010567 | Chr5 | 6089444 | 6092955 | 1563 |
| MsG0880047259.01 | 1.448373 | 0.000926 | 0.010567 | Chr8 | 83877644 | 83884462 | 4188 |
| novel.7364 | -1.96623 | 0.000926 | 0.01057 | Chr7 | 15474982 | 15477023 | 1023 |
| novel.1377 | 5.379306 | 0.000927 | 0.010574 | Chr2 | 35126525 | 35129218 | 956 |
| novel.6331 | -1.91178 | 0.000928 | 0.010576 | Chr6 | 26203186 | 26214832 | 3006 |
| MsG0080047989.01 | 1.485286 | 0.000931 | 0.010605 | contig161end | 19875 | 24075 | 1500 |
| MsG0780039709.01 | -5.81776 | 0.000931 | 0.010605 | Chr7 | 67907165 | 67907902 | 738 |
| novel.4866 | 3.617951 | 0.000932 | 0.010619 | Chr5 | 1.04E+08 | 1.04E+08 | 695 |
| novel.3631 | 2.613641 | 0.000933 | 0.01062 | Chr4 | 77953538 | 77955392 | 975 |
| MsG0380015227.01 | -1.92705 | 0.000936 | 0.010654 | Chr3 | 67073139 | 67075163 | 831 |
| novel.895 | -1.75429 | 0.000939 | 0.010683 | Chr1 | 57634274 | 57637789 | 2437 |
| MsG0480023898.01 | -2.3943 | 0.00094 | 0.010683 | Chr4 | 91023544 | 91026671 | 1558 |
| MsG0180004466.01 | 1.197488 | 0.000943 | 0.010717 | Chr1 | 78437490 | 78448227 | 2733 |
| novel.6916 | 5.247278 | 0.000949 | 0.010776 | Chr7 | 26658235 | 26658745 | 511 |
| novel.1205 | -4.90854 | 0.000949 | 0.010776 | Chr2 | 6513939 | 6516755 | 622 |
| novel.311 | -3.8328 | 0.00095 | 0.010776 | Chr1 | 58389772 | 58390260 | 489 |
| novel.3031 | -6.22576 | 0.00095 | 0.010776 | Chr3 | 76612955 | 76614509 | 378 |
| MsG0480022833.01 | -2.96912 | 0.000951 | 0.010786 | Chr4 | 77293014 | 77297036 | 2059 |
| novel.4847 | -6.08499 | 0.000955 | 0.010823 | Chr5 | 1.01E+08 | 1.01E+08 | 396 |
| MsG0680032243.01 | -3.39334 | 0.000955 | 0.010823 | Chr6 | 37227131 | 37227469 | 339 |
| novel.4308 | 1.455336 | 0.000955 | 0.010824 | Chr5 | 4232316 | 4237787 | 1143 |
| MsG0880042336.01 | -1.5817 | 0.000956 | 0.010826 | Chr8 | 7227566 | 7237498 | 3730 |
| MsG0180005787.01 | -1.24603 | 0.000956 | 0.010828 | Chr1 | 96142856 | 96144380 | 710 |
| novel.3662 | -3.24906 | 0.000958 | 0.010841 | Chr4 | 82560161 | 82567283 | 1774 |
| novel.6627 | 6.097637 | 0.000959 | 0.010853 | Chr6 | 88960515 | 88964213 | 1005 |
| MsG0580025739.01 | -1.9744 | 0.000965 | 0.010909 | Chr5 | 23458179 | 23461430 | 1553 |
| MsG0680034700.01 | 1.923961 | 0.000969 | 0.010954 | Chr6 | 93069429 | 93075085 | 3508 |
| MsG0580029502.01 | 1.069174 | 0.000971 | 0.010969 | Chr5 | 97813386 | 97822483 | 2477 |
| MsG0480022117.01 | -3.26986 | 0.000974 | 0.011 | Chr4 | 68428341 | 68428715 | 375 |
| MsG0480021797.01 | -5.29201 | 0.000976 | 0.011025 | Chr4 | 64415715 | 64418710 | 1476 |
| MsG0580028407.01 | -2.58245 | 0.000977 | 0.011032 | Chr5 | 80145656 | 80152040 | 1444 |
| MsG0380013792.01 | -2.47749 | 0.000978 | 0.011038 | Chr3 | 45734181 | 45736643 | 2250 |
| MsG0880043653.01 | -1.3502 | 0.00098 | 0.011053 | Chr8 | 28454378 | 28459590 | 915 |
| MsG0380013794.01 | -2.49892 | 0.00098 | 0.011053 | Chr3 | 45747213 | 45752659 | 1065 |
| novel.8861 | -5.40546 | 0.000981 | 0.011053 | contig222end | 8351 | 9062 | 712 |
| novel.6863 | 3.835871 | 0.000981 | 0.011053 | Chr7 | 17787333 | 17787861 | 508 |
| MsG0580025176.01 | 6.03972 | 0.000981 | 0.011054 | Chr5 | 14770038 | 14770955 | 918 |
| MsG0380014982.01 | -1.84751 | 0.000982 | 0.011054 | Chr3 | 63559787 | 63560125 | 339 |
| novel.1852 | 1.318705 | 0.000985 | 0.011083 | Chr2 | 34684932 | 34686326 | 647 |
| MsG0480021049.01 | 1.282529 | 0.000986 | 0.011097 | Chr4 | 52666787 | 52667233 | 447 |
| MsG0480019564.01 | 5.893945 | 0.000988 | 0.011106 | Chr4 | 22288263 | 22288778 | 516 |
| MsG0480018480.01 | 3.793704 | 0.000988 | 0.011106 | Chr4 | 5564367 | 5564708 | 342 |
| novel.1750 | 1.951134 | 0.000988 | 0.011106 | Chr2 | 14203034 | 14210484 | 1533 |
| MsG0580028095.01 | -6.20925 | 0.000989 | 0.011109 | Chr5 | 74500091 | 74510991 | 6387 |
| novel.5844 | -5.31044 | 0.00099 | 0.011118 | Chr6 | 68837889 | 68841005 | 1641 |
| MsG0180004805.01 | -1.48755 | 0.000991 | 0.011118 | Chr1 | 82974275 | 82979657 | 2064 |
| MsG0580026097.01 | 5.997332 | 0.000992 | 0.011127 | Chr5 | 30742004 | 30742725 | 609 |
| MsG0180005229.01 | 1.725213 | 0.000996 | 0.01117 | Chr1 | 88878369 | 88884038 | 1425 |
| MsG0680031041.01 | 1.322199 | 0.000996 | 0.01117 | Chr6 | 13183913 | 13189255 | 1766 |
| MsG0480022043.01 | 2.348373 | 0.000997 | 0.011176 | Chr4 | 67291335 | 67292012 | 678 |
| MsG0680033431.01 | -6.41916 | 0.000999 | 0.01119 | Chr6 | 64752918 | 64754991 | 1224 |
| novel.3760 | -2.37519 | 0.001 | 0.011201 | Chr4 | 1794102 | 1795174 | 955 |
| MsG0180006187.01 | -2.08411 | 0.001004 | 0.011235 | Chr1 | 1.01E+08 | 1.01E+08 | 195 |
| novel.3590 | 5.755729 | 0.001005 | 0.011246 | Chr4 | 72404640 | 72409346 | 593 |
| MsG0080048410.01 | -2.0755 | 0.00101 | 0.0113 | contig304end | 43745 | 44104 | 360 |
| MsG0180000267.01 | -2.83949 | 0.001011 | 0.01131 | Chr1 | 3529188 | 3529385 | 198 |
| novel.6593 | 5.771412 | 0.001012 | 0.011313 | Chr6 | 82223758 | 82226564 | 1180 |
| novel.5282 | -2.82204 | 0.001015 | 0.011339 | Chr5 | 74758768 | 74764417 | 5650 |
| novel.730 | -5.8724 | 0.001018 | 0.011373 | Chr1 | 18704435 | 18705238 | 512 |
| MsG0780038444.01 | -1.57024 | 0.001021 | 0.011389 | Chr7 | 46966978 | 46977705 | 912 |
| MsG0480022590.01 | -1.88422 | 0.001021 | 0.011389 | Chr4 | 74430972 | 74438183 | 654 |
| MsG0280007915.01 | -1.46313 | 0.001021 | 0.011389 | Chr2 | 22856694 | 22858271 | 474 |
| MsG0780041140.01 | 1.987619 | 0.001021 | 0.011389 | Chr7 | 87074661 | 87075305 | 645 |
| MsG0780040592.01 | 3.762274 | 0.001024 | 0.011418 | Chr7 | 79631705 | 79640141 | 504 |
| MsG0180005991.01 | -1.13868 | 0.001025 | 0.011418 | Chr1 | 98571226 | 98575710 | 2311 |
| MsG0180006047.01 | -1.94518 | 0.001025 | 0.011421 | Chr1 | 99354806 | 99357866 | 1419 |
| MsG0780038755.01 | -1.39849 | 0.001031 | 0.011479 | Chr7 | 52614375 | 52620273 | 1988 |
| MsG0480023162.01 | 1.551499 | 0.001033 | 0.011497 | Chr4 | 81623819 | 81624055 | 237 |
| MsG0880044042.01 | -2.11135 | 0.001034 | 0.011502 | Chr8 | 34701029 | 34705030 | 783 |
| novel.481 | -3.90419 | 0.001034 | 0.011502 | Chr1 | 85876995 | 85880277 | 2646 |
| MsG0280009781.01 | 1.997664 | 0.001035 | 0.01151 | Chr2 | 57867654 | 57868721 | 1068 |
| MsG0480021752.01 | 2.443638 | 0.001036 | 0.01151 | Chr4 | 63871173 | 63879402 | 1356 |
| novel.6608 | -5.81801 | 0.001036 | 0.01151 | Chr6 | 84765474 | 84770172 | 697 |
| MsG0880042932.01 | 1.442484 | 0.001036 | 0.01151 | Chr8 | 16202211 | 16203469 | 621 |
| MsG0180001804.01 | -2.35966 | 0.001038 | 0.011525 | Chr1 | 27250971 | 27251624 | 654 |
| MsG0180000834.01 | 2.395742 | 0.001039 | 0.011535 | Chr1 | 11853314 | 11857095 | 1005 |
| novel.1564 | -1.69398 | 0.001041 | 0.011545 | Chr2 | 76740011 | 76743837 | 1909 |
| novel.1322 | 2.996998 | 0.001043 | 0.011563 | Chr2 | 26716728 | 26717560 | 745 |
| MsG0380016204.01 | 1.271579 | 0.001045 | 0.011573 | Chr3 | 80414691 | 80419469 | 1517 |
| MsG0580025789.01 | 2.363716 | 0.001045 | 0.011573 | Chr5 | 24162804 | 24164012 | 615 |
| MsG0580025858.01 | -2.84806 | 0.001045 | 0.011573 | Chr5 | 25240926 | 25242740 | 1641 |
| novel.8984 | -6.26295 | 0.001045 | 0.011573 | contig405end | 17736 | 21518 | 513 |
| MsG0180000819.01 | -1.15176 | 0.001045 | 0.011573 | Chr1 | 11623684 | 11625805 | 1417 |
| novel.897 | 5.743854 | 0.001052 | 0.011637 | Chr1 | 57967673 | 57968875 | 1203 |
| MsG0280011079.01 | -1.75909 | 0.001053 | 0.011644 | Chr2 | 79204667 | 79206543 | 1253 |
| novel.7424 | 5.850058 | 0.001056 | 0.011673 | Chr7 | 28179148 | 28180832 | 1541 |
| MsG0180004635.01 | -3.57423 | 0.001056 | 0.011673 | Chr1 | 80534541 | 80545094 | 3045 |
| MsG0480021159.01 | 1.035314 | 0.001057 | 0.011673 | Chr4 | 54534666 | 54538644 | 822 |
| MsG0380014166.01 | -1.08634 | 0.001057 | 0.011673 | Chr3 | 50746898 | 50755849 | 1403 |
| MsG0880046428.01 | 1.914037 | 0.001061 | 0.01171 | Chr8 | 73265436 | 73272618 | 1965 |
| MsG0480021242.01 | -1.73419 | 0.001061 | 0.011711 | Chr4 | 55720768 | 55747521 | 4437 |
| MsG0480019284.01 | 1.467596 | 0.001062 | 0.01172 | Chr4 | 17740072 | 17740458 | 387 |
| MsG0680030614.01 | -5.92931 | 0.001064 | 0.011734 | Chr6 | 6092955 | 6093215 | 261 |
| MsG0280008073.01 | -1.61293 | 0.001065 | 0.011738 | Chr2 | 25125481 | 25135921 | 2235 |
| novel.3209 | -1.65064 | 0.001065 | 0.011739 | Chr3 | 1.03E+08 | 1.03E+08 | 1442 |
| novel.8200 | 5.926609 | 0.001066 | 0.011746 | Chr8 | 76852124 | 76860202 | 964 |
| MsG0680034673.01 | 3.46129 | 0.001067 | 0.011755 | Chr6 | 92607313 | 92608352 | 984 |
| novel.6267 | -4.71333 | 0.001071 | 0.011789 | Chr6 | 18114781 | 18118470 | 734 |
| MsG0080048732.01 | -2.77012 | 0.001073 | 0.011802 | contig408end | 187455 | 194243 | 3141 |
| novel.5121 | -6.23014 | 0.001073 | 0.011805 | Chr5 | 43702461 | 43704897 | 2074 |
| MsG0880045955.01 | -1.70445 | 0.001076 | 0.011835 | Chr8 | 66893202 | 66897395 | 985 |
| MsG0780036029.01 | -3.7573 | 0.001077 | 0.011836 | Chr7 | 1524404 | 1532396 | 2289 |
| novel.2123 | 2.419965 | 0.001079 | 0.011851 | Chr3 | 325913 | 326906 | 994 |
| MsG0880042433.01 | 4.840083 | 0.001087 | 0.011933 | Chr8 | 8619287 | 8625268 | 2858 |
| MsG0780039740.01 | -1.56372 | 0.001087 | 0.011939 | Chr7 | 68486799 | 68490270 | 1136 |
| novel.9355 | -1.29153 | 0.001088 | 0.011941 | contig640end | 22810 | 27180 | 1717 |
| MsG0380017729.01 | -1.96629 | 0.001088 | 0.011941 | Chr3 | 1E+08 | 1E+08 | 3087 |
| MsG0580025218.01 | -2.42264 | 0.00109 | 0.011956 | Chr5 | 15407886 | 15409774 | 1773 |
| MsG0480020444.01 | -2.48458 | 0.001093 | 0.011979 | Chr4 | 41602271 | 41607523 | 1140 |
| MsG0380013832.01 | -1.63329 | 0.001099 | 0.012046 | Chr3 | 46287032 | 46293055 | 937 |
| novel.327 | -6.12254 | 0.001102 | 0.012072 | Chr1 | 60916882 | 60918018 | 1030 |
| novel.7736 | -2.15887 | 0.001103 | 0.012075 | Chr7 | 88874940 | 88876754 | 1815 |
| novel.332 | -1.88348 | 0.001104 | 0.01208 | Chr1 | 61424509 | 61427663 | 2809 |
| MsG0180006055.01 | 2.04535 | 0.001104 | 0.01208 | Chr1 | 99478476 | 99481349 | 1674 |
| MsG0280011203.01 | -5.98296 | 0.001104 | 0.01208 | Chr2 | 80907532 | 80911279 | 1978 |
| novel.6216 | 1.953891 | 0.001105 | 0.01208 | Chr6 | 11987509 | 11992465 | 3037 |
| MsG0580025208.01 | -2.34042 | 0.001105 | 0.01208 | Chr5 | 15339908 | 15341086 | 1179 |
| MsG0480019577.01 | 6.025444 | 0.001107 | 0.012095 | Chr4 | 22465294 | 22466505 | 1212 |
| MsG0880043433.01 | -6.38063 | 0.001108 | 0.0121 | Chr8 | 24765240 | 24767205 | 1299 |
| MsG0780036978.01 | 2.209611 | 0.00111 | 0.01212 | Chr7 | 17448241 | 17451895 | 744 |
| novel.8177 | 6.077984 | 0.001113 | 0.012149 | Chr8 | 73658046 | 73659282 | 721 |
| MsG0580026785.01 | -5.28669 | 0.001115 | 0.012162 | Chr5 | 44756020 | 44756356 | 234 |
| novel.7474 | -5.76103 | 0.001115 | 0.012164 | Chr7 | 40595977 | 40596578 | 467 |
| MsG0680033367.01 | -5.78972 | 0.00112 | 0.012211 | Chr6 | 63229244 | 63238489 | 1419 |
| MsG0580026001.01 | 5.34287 | 0.001123 | 0.012235 | Chr5 | 28946092 | 28947019 | 534 |
| MsG0380013852.01 | -3.14638 | 0.001123 | 0.012238 | Chr3 | 46707389 | 46707604 | 216 |
| MsG0880042740.01 | -6.14525 | 0.001125 | 0.012252 | Chr8 | 13389224 | 13390882 | 1659 |
| novel.3182 | -3.60932 | 0.001126 | 0.012263 | Chr3 | 99449549 | 99451389 | 604 |
| novel.5222 | 5.966592 | 0.001132 | 0.012319 | Chr5 | 64485887 | 64486345 | 459 |
| MsG0480018669.01 | -2.14818 | 0.001133 | 0.012327 | Chr4 | 8433023 | 8443876 | 2616 |
| novel.7254 | -2.92302 | 0.001135 | 0.012348 | Chr7 | 91324102 | 91327305 | 3204 |
| novel.4439 | -2.08929 | 0.001137 | 0.012363 | Chr5 | 24620987 | 24623073 | 1284 |
| MsG0780040598.01 | -3.82877 | 0.001138 | 0.012368 | Chr7 | 79774697 | 79781047 | 2355 |
| MsG0280010391.01 | 1.636379 | 0.001139 | 0.012372 | Chr2 | 68961887 | 68967391 | 1948 |
| MsG0480019593.01 | -3.112 | 0.00114 | 0.012378 | Chr4 | 22873706 | 22876175 | 993 |
| MsG0280010147.01 | 1.431473 | 0.001141 | 0.012384 | Chr2 | 65326309 | 65337591 | 1695 |
| novel.2010 | -5.9054 | 0.001145 | 0.01242 | Chr2 | 70515979 | 70522818 | 649 |
| MsG0580025682.01 | -2.61956 | 0.001146 | 0.01243 | Chr5 | 22587487 | 22601365 | 3114 |
| novel.2277 | 2.257745 | 0.001155 | 0.012522 | Chr3 | 25824296 | 25831217 | 3064 |
| novel.8013 | 3.005062 | 0.001157 | 0.012536 | Chr8 | 40929437 | 40932341 | 951 |
| MsG0280009470.01 | 5.790941 | 0.001157 | 0.012539 | Chr2 | 52564845 | 52602070 | 4134 |
| MsG0480023193.01 | -3.93709 | 0.00116 | 0.012561 | Chr4 | 82064106 | 82065436 | 369 |
| novel.3538 | 2.497219 | 0.001162 | 0.012581 | Chr4 | 63468119 | 63469997 | 1141 |
| MsG0480022638.01 | -1.60398 | 0.001167 | 0.012634 | Chr4 | 74942831 | 74943892 | 1062 |
| novel.1924 | -2.56827 | 0.001174 | 0.012702 | Chr2 | 54165263 | 54173546 | 881 |
| MsG0280007596.01 | -2.379 | 0.001176 | 0.012717 | Chr2 | 17834375 | 17849841 | 2613 |
| MsG0680034914.01 | -1.44042 | 0.001176 | 0.012717 | Chr6 | 96558736 | 96564110 | 2839 |
| novel.7615 | 5.720397 | 0.001177 | 0.012721 | Chr7 | 70652713 | 70653259 | 465 |
| MsG0680035841.01 | -2.43123 | 0.001186 | 0.012814 | Chr6 | 1.13E+08 | 1.13E+08 | 1980 |
| MsG0380016177.01 | 2.257575 | 0.001187 | 0.012815 | Chr3 | 80007678 | 80009175 | 969 |
| novel.3526 | 1.683762 | 0.001188 | 0.01283 | Chr4 | 60286676 | 60291453 | 951 |
| MsG0380015910.01 | 2.928656 | 0.00119 | 0.012845 | Chr3 | 76549794 | 76551607 | 948 |
| MsG0880041923.01 | -5.8931 | 0.001193 | 0.012869 | Chr8 | 1230491 | 1234728 | 1411 |
| MsG0780039016.01 | -2.89505 | 0.001193 | 0.012869 | Chr7 | 56831127 | 56839037 | 1890 |
| novel.5811 | -5.33584 | 0.001196 | 0.012891 | Chr6 | 60798807 | 60799697 | 891 |
| MsG0880042735.01 | -5.79502 | 0.001198 | 0.012914 | Chr8 | 13351814 | 13355366 | 2634 |
| novel.953 | -4.67799 | 0.001203 | 0.012957 | Chr1 | 70051097 | 70052318 | 768 |
| novel.4803 | 1.650618 | 0.001204 | 0.01296 | Chr5 | 95870112 | 95872088 | 801 |
| novel.1580 | 5.774618 | 0.001205 | 0.01297 | Chr2 | 78049822 | 78052610 | 572 |
| MsG0880041836.01 | 1.140205 | 0.001206 | 0.012976 | Chr8 | 40317 | 44715 | 1398 |
| MsG0480019046.01 | 2.237239 | 0.001207 | 0.012982 | Chr4 | 13750638 | 13751975 | 1191 |
| MsG0380016969.01 | 1.757681 | 0.001208 | 0.01299 | Chr3 | 90123894 | 90125264 | 703 |
| novel.4690 | 1.571121 | 0.001212 | 0.01303 | Chr5 | 76045533 | 76047018 | 593 |
| MsG0280011004.01 | 2.554002 | 0.001215 | 0.013054 | Chr2 | 78225884 | 78229968 | 538 |
| MsG0880046898.01 | -1.56607 | 0.001217 | 0.013072 | Chr8 | 79653843 | 79656675 | 1509 |
| MsG0880042912.01 | -3.31224 | 0.001222 | 0.013115 | Chr8 | 15814125 | 15814466 | 342 |
| MsG0880047603.01 | 1.463978 | 0.001222 | 0.013115 | Chr8 | 88629631 | 88630695 | 1065 |
| novel.150 | -5.40856 | 0.001223 | 0.013115 | Chr1 | 19337707 | 19340744 | 1403 |
| MsG0480019490.01 | 6.030089 | 0.001223 | 0.013115 | Chr4 | 21112057 | 21113184 | 951 |
| MsG0580028711.01 | 5.856099 | 0.001226 | 0.013143 | Chr5 | 85264352 | 85268774 | 2980 |
| novel.846 | 6.362893 | 0.001227 | 0.01315 | Chr1 | 43374428 | 43375640 | 1129 |
| novel.2887 | 2.14685 | 0.001229 | 0.013163 | Chr3 | 45449679 | 45456225 | 2749 |
| MsG0080048989.01 | 2.718535 | 0.001229 | 0.013163 | contig591end | 6096 | 9932 | 2012 |
| novel.1428 | -5.20785 | 0.00123 | 0.013163 | Chr2 | 48856437 | 48858441 | 391 |
| MsG0480021853.01 | 1.307125 | 0.00123 | 0.013163 | Chr4 | 65194743 | 65195129 | 387 |
| MsG0580028177.01 | 1.922455 | 0.001232 | 0.013177 | Chr5 | 76186436 | 76189684 | 2238 |
| MsG0180004244.01 | 1.88199 | 0.001236 | 0.013216 | Chr1 | 75492036 | 75492659 | 624 |
| novel.3761 | 6.232608 | 0.001237 | 0.013225 | Chr4 | 1848834 | 1849230 | 397 |
| MsG0380012088.01 | 6.196449 | 0.001242 | 0.013269 | Chr3 | 10759368 | 10760349 | 982 |
| novel.7422 | 1.808948 | 0.001242 | 0.013269 | Chr7 | 27207922 | 27211311 | 1733 |
| MsG0680033643.01 | 2.645386 | 0.001249 | 0.013336 | Chr6 | 70147087 | 70150144 | 1527 |
| MsG0180002460.01 | 1.130313 | 0.001252 | 0.013363 | Chr1 | 38651936 | 38654747 | 1157 |
| MsG0680030986.01 | 4.478156 | 0.001252 | 0.013363 | Chr6 | 12541592 | 12544983 | 1299 |
| novel.6373 | -4.65555 | 0.001253 | 0.013364 | Chr6 | 34425688 | 34426165 | 478 |
| novel.6749 | -3.37337 | 0.001255 | 0.013386 | Chr6 | 1.08E+08 | 1.08E+08 | 1919 |
| MsG0880047212.01 | 1.667591 | 0.001256 | 0.013389 | Chr8 | 83355836 | 83357713 | 1081 |
| novel.2138 | -4.66372 | 0.001257 | 0.013397 | Chr3 | 3136698 | 3138183 | 521 |
| MsG0380016449.01 | 2.548729 | 0.001259 | 0.013413 | Chr3 | 83655355 | 83655905 | 381 |
| MsG0480022058.01 | -1.31642 | 0.001262 | 0.013436 | Chr4 | 67527109 | 67529129 | 657 |
| novel.6054 | 6.13686 | 0.001264 | 0.013453 | Chr6 | 1.04E+08 | 1.04E+08 | 1665 |
| MsG0180004167.01 | 2.975957 | 0.001265 | 0.013465 | Chr1 | 74330893 | 74334352 | 3042 |
| MsG0480022619.01 | -1.32092 | 0.001267 | 0.013476 | Chr4 | 74778484 | 74782616 | 1224 |
| MsG0280010662.01 | 3.137272 | 0.001272 | 0.013523 | Chr2 | 73092414 | 73092683 | 270 |
| MsG0580029929.01 | -1.48154 | 0.001274 | 0.013547 | Chr5 | 1.04E+08 | 1.04E+08 | 2161 |
| MsG0180005918.01 | -1.02476 | 0.001278 | 0.013576 | Chr1 | 97541550 | 97544540 | 2808 |
| MsG0380012173.01 | 1.888609 | 0.001278 | 0.013576 | Chr3 | 12156096 | 12156350 | 255 |
| MsG0080047939.01 | 3.275476 | 0.001279 | 0.013576 | contig152end | 65919 | 70759 | 573 |
| MsG0580028840.01 | -2.16631 | 0.00128 | 0.013583 | Chr5 | 87356140 | 87366545 | 2847 |
| novel.5573 | 3.481082 | 0.00128 | 0.013583 | Chr6 | 10856274 | 10861746 | 960 |
| novel.1295 | 3.121514 | 0.001281 | 0.013583 | Chr2 | 20739978 | 20742373 | 1634 |
| novel.6458 | -5.7288 | 0.001282 | 0.01359 | Chr6 | 51847039 | 51847719 | 479 |
| MsG0480018593.01 | -2.8402 | 0.001285 | 0.013621 | Chr4 | 7086196 | 7094776 | 2811 |
| MsG0380015817.01 | 2.693185 | 0.001294 | 0.013709 | Chr3 | 75517037 | 75522473 | 1586 |
| novel.4251 | -2.06646 | 0.001295 | 0.013718 | Chr4 | 90280549 | 90286200 | 2703 |
| MsG0580029325.01 | -1.50662 | 0.001299 | 0.013755 | Chr5 | 95286929 | 95287571 | 522 |
| MsG0380014967.01 | -3.20691 | 0.001302 | 0.013778 | Chr3 | 63349048 | 63351207 | 1221 |
| MsG0680031118.01 | -2.3272 | 0.001303 | 0.013788 | Chr6 | 14744728 | 14758586 | 2276 |
| MsG0080048955.01 | 1.387347 | 0.001308 | 0.013831 | contig565end | 9976 | 13635 | 1289 |
| MsG0880046447.01 | 1.260394 | 0.001312 | 0.013869 | Chr8 | 73483021 | 73485242 | 892 |
| novel.4442 | -5.79411 | 0.001313 | 0.013872 | Chr5 | 24898662 | 24899167 | 412 |
| MsG0880047438.01 | 2.687977 | 0.001316 | 0.013904 | Chr8 | 86307958 | 86308233 | 276 |
| MsG0480023725.01 | -1.95557 | 0.001317 | 0.013909 | Chr4 | 89026818 | 89030433 | 2284 |
| MsG0880046859.01 | 1.312464 | 0.001318 | 0.01391 | Chr8 | 79167423 | 79171554 | 1351 |
| novel.422 | 5.568217 | 0.001318 | 0.013913 | Chr1 | 77459463 | 77463096 | 832 |
| MsG0880045356.01 | -2.83526 | 0.001319 | 0.01392 | Chr8 | 58012792 | 58015658 | 1077 |
| novel.1197 | 3.072954 | 0.001324 | 0.013964 | Chr2 | 4478910 | 4482496 | 981 |
| MsG0180004123.01 | -5.86048 | 0.001325 | 0.013964 | Chr1 | 73719218 | 73721437 | 1827 |
| novel.739 | -5.93181 | 0.001325 | 0.013964 | Chr1 | 19958297 | 19959015 | 483 |
| MsG0380012566.01 | 1.350448 | 0.001325 | 0.013964 | Chr3 | 19887993 | 19892757 | 1674 |
| MsG0780039955.01 | 1.452462 | 0.001326 | 0.013966 | Chr7 | 71067352 | 71069960 | 941 |
| MsG0880045884.01 | 2.56232 | 0.001328 | 0.01398 | Chr8 | 65969500 | 65970861 | 1362 |
| MsG0180001654.01 | 3.330135 | 0.00133 | 0.013994 | Chr1 | 24761115 | 24763919 | 2805 |
| MsG0280010529.01 | -5.79051 | 0.00133 | 0.013994 | Chr2 | 70986018 | 70989802 | 2031 |
| novel.5135 | 5.274316 | 0.001331 | 0.014 | Chr5 | 45266111 | 45267447 | 672 |
| MsG0180001723.01 | -2.71903 | 0.001333 | 0.014015 | Chr1 | 26060695 | 26061012 | 318 |
| MsG0380015162.01 | -1.76498 | 0.001334 | 0.014015 | Chr3 | 66016636 | 66032890 | 4423 |
| MsG0280010800.01 | -3.6439 | 0.001341 | 0.014084 | Chr2 | 75210084 | 75210758 | 675 |
| MsG0680032912.01 | 5.801832 | 0.001341 | 0.014084 | Chr6 | 52755411 | 52759407 | 1605 |
| MsG0880047352.01 | -2.34936 | 0.001343 | 0.014097 | Chr8 | 85140172 | 85143765 | 1897 |
| novel.2678 | -2.53961 | 0.001344 | 0.014108 | Chr3 | 976230 | 979248 | 3019 |
| MsG0680032312.01 | 1.45611 | 0.001346 | 0.014117 | Chr6 | 38305083 | 38308160 | 925 |
| novel.1109 | -2.70436 | 0.001348 | 0.01414 | Chr1 | 96228699 | 96235307 | 1007 |
| novel.6634 | 5.116611 | 0.001352 | 0.014171 | Chr6 | 89777319 | 89777977 | 270 |
| novel.7088 | 5.912441 | 0.001352 | 0.014172 | Chr7 | 66337397 | 66338080 | 684 |
| MsG0380016711.01 | 1.148764 | 0.001354 | 0.014182 | Chr3 | 86914055 | 86920594 | 1595 |
| MsG0380014940.01 | -1.41568 | 0.001357 | 0.01421 | Chr3 | 62812466 | 62816711 | 2034 |
| MsG0880042298.01 | -1.17955 | 0.001369 | 0.01433 | Chr8 | 6691123 | 6694274 | 672 |
| novel.3101 | 4.903342 | 0.00137 | 0.014336 | Chr3 | 87359047 | 87359950 | 820 |
| novel.5572 | 5.138144 | 0.00137 | 0.014336 | Chr6 | 10668609 | 10669728 | 849 |
| MsG0580029996.01 | -4.67226 | 0.001371 | 0.014341 | Chr5 | 1.05E+08 | 1.05E+08 | 2750 |
| novel.3116 | -1.87734 | 0.001373 | 0.014353 | Chr3 | 89503089 | 89506262 | 1473 |
| novel.4217 | 4.809647 | 0.001374 | 0.014353 | Chr4 | 85574515 | 85576219 | 979 |
| MsG0680035200.01 | 5.384292 | 0.001374 | 0.014355 | Chr6 | 1E+08 | 1E+08 | 2715 |
| MsG0780037751.01 | -1.53449 | 0.001378 | 0.014386 | Chr7 | 32684166 | 32689106 | 1534 |
| novel.1957 | 4.220168 | 0.00138 | 0.014403 | Chr2 | 62300413 | 62301194 | 782 |
| MsG0480021061.01 | 1.254082 | 0.00138 | 0.014403 | Chr4 | 52819311 | 52822101 | 762 |
| MsG0780039085.01 | -2.83052 | 0.00139 | 0.014501 | Chr7 | 57664527 | 57673058 | 3165 |
| MsG0280008096.01 | -5.27998 | 0.001393 | 0.014525 | Chr2 | 25430613 | 25432094 | 357 |
| novel.9033 | 1.369329 | 0.001397 | 0.014564 | contig448end | 20511 | 23933 | 3423 |
| MsG0580024601.01 | -2.00813 | 0.001402 | 0.014612 | Chr5 | 6778516 | 6781139 | 1152 |
| MsG0580027259.01 | -1.41565 | 0.001404 | 0.014626 | Chr5 | 57138915 | 57140051 | 1137 |
| novel.5963 | 4.205628 | 0.001407 | 0.01465 | Chr6 | 90586992 | 90589152 | 882 |
| novel.6893 | 8.388837 | 0.001409 | 0.014665 | Chr7 | 21886554 | 21888554 | 1691 |
| MsG0880042035.01 | 1.355758 | 0.001409 | 0.014666 | Chr8 | 2948138 | 2963755 | 2702 |
| MsG0480022095.01 | -2.2107 | 0.00141 | 0.014666 | Chr4 | 68135447 | 68135968 | 522 |
| MsG0580027825.01 | 1.64123 | 0.00141 | 0.014666 | Chr5 | 69178207 | 69183335 | 597 |
| novel.6103 | -1.36095 | 0.001411 | 0.014666 | Chr6 | 1.1E+08 | 1.1E+08 | 2332 |
| MsG0680030996.01 | -3.35947 | 0.001413 | 0.014679 | Chr6 | 12662425 | 12665538 | 1746 |
| MsG0180001404.01 | -3.56273 | 0.001416 | 0.014707 | Chr1 | 20636960 | 20650590 | 7028 |
| MsG0580024471.01 | 1.331525 | 0.001418 | 0.014722 | Chr5 | 5368202 | 5371691 | 1919 |
| MsG0580028962.01 | 2.001664 | 0.001419 | 0.014725 | Chr5 | 89664360 | 89665348 | 801 |
| MsG0480020529.01 | -5.91441 | 0.001421 | 0.014745 | Chr4 | 42941423 | 42942401 | 675 |
| MsG0680031487.01 | 2.151329 | 0.001425 | 0.014785 | Chr6 | 21119465 | 21121274 | 737 |
| novel.4849 | -5.85358 | 0.001426 | 0.014785 | Chr5 | 1.01E+08 | 1.01E+08 | 2144 |
| MsG0180005983.01 | -2.19905 | 0.001426 | 0.014785 | Chr1 | 98462283 | 98468138 | 1809 |
| MsG0880043587.01 | -5.867 | 0.001434 | 0.014854 | Chr8 | 27344346 | 27345878 | 750 |
| MsG0480019671.01 | -5.13333 | 0.001439 | 0.014904 | Chr4 | 24501063 | 24507862 | 2283 |
| MsG0580024340.01 | -1.53555 | 0.001443 | 0.014945 | Chr5 | 3560269 | 3560490 | 195 |
| novel.8337 | 3.071901 | 0.001445 | 0.014959 | Chr8 | 7453700 | 7457072 | 1337 |
| MsG0780036756.01 | -2.41081 | 0.001452 | 0.01502 | Chr7 | 13245295 | 13248152 | 77 |
| novel.3589 | -1.15502 | 0.001452 | 0.01502 | Chr4 | 72276253 | 72280682 | 2767 |
| MsG0280011256.01 | -1.06229 | 0.001453 | 0.015026 | Chr2 | 81506486 | 81520647 | 4583 |
| novel.4718 | 5.912604 | 0.001458 | 0.01507 | Chr5 | 80806211 | 80806763 | 459 |
| MsG0180003732.01 | -1.01272 | 0.001465 | 0.015134 | Chr1 | 67405425 | 67430883 | 6420 |
| novel.4282 | 3.852547 | 0.001465 | 0.015136 | Chr5 | 773738 | 777240 | 1152 |
| MsG0480021424.01 | 1.073417 | 0.001469 | 0.01517 | Chr4 | 57734891 | 57736732 | 1842 |
| novel.3976 | -2.36967 | 0.001474 | 0.015211 | Chr4 | 49647474 | 49655755 | 1638 |
| MsG0880044826.01 | 1.956175 | 0.001475 | 0.01522 | Chr8 | 49764886 | 49766347 | 1462 |
| MsG0380013445.01 | 1.70819 | 0.001477 | 0.015233 | Chr3 | 38114806 | 38115132 | 327 |
| MsG0780038054.01 | 5.100894 | 0.001478 | 0.015237 | Chr7 | 39239140 | 39240050 | 657 |
| novel.7161 | 2.376152 | 0.001481 | 0.015261 | Chr7 | 77331029 | 77334210 | 2103 |
| MsG0380012801.01 | -1.8078 | 0.001482 | 0.015261 | Chr3 | 24777426 | 24777743 | 318 |
| MsG0580025522.01 | 2.570536 | 0.001482 | 0.015261 | Chr5 | 19969379 | 19969870 | 492 |
| MsG0580027770.01 | 5.695866 | 0.001482 | 0.015261 | Chr5 | 68302860 | 68308712 | 1377 |
| novel.5077 | 2.511613 | 0.001483 | 0.015262 | Chr5 | 32005920 | 32009485 | 1526 |
| MsG0580024193.01 | -1.28757 | 0.001484 | 0.015273 | Chr5 | 1776713 | 1780108 | 792 |
| MsG0180000296.01 | -2.24113 | 0.001485 | 0.015279 | Chr1 | 4097582 | 4098007 | 426 |
| MsG0480023523.01 | -1.71287 | 0.001486 | 0.015281 | Chr4 | 86448239 | 86451430 | 1671 |
| novel.234 | 5.125763 | 0.00149 | 0.015318 | Chr1 | 36886758 | 36887745 | 880 |
| novel.3597 | -5.87403 | 0.001492 | 0.015327 | Chr4 | 73446745 | 73455406 | 672 |
| MsG0680035185.01 | -4.80877 | 0.001495 | 0.015362 | Chr6 | 1E+08 | 1E+08 | 2094 |
| MsG0680032790.01 | 1.774538 | 0.001496 | 0.015366 | Chr6 | 49736645 | 49747647 | 877 |
| novel.8601 | -5.88681 | 0.001498 | 0.015376 | Chr8 | 58920311 | 58921776 | 760 |
| MsG0680033089.01 | -1.79867 | 0.001499 | 0.015379 | Chr6 | 56445903 | 56456204 | 1829 |
| MsG0880046360.01 | 3.278787 | 0.001499 | 0.015382 | Chr8 | 72398400 | 72401610 | 546 |
| novel.3960 | 1.276595 | 0.001502 | 0.015406 | Chr4 | 46113396 | 46115492 | 1118 |
| MsG0180000670.01 | 2.655555 | 0.001503 | 0.015406 | Chr1 | 9519137 | 9520165 | 522 |
| MsG0080048668.01 | 1.195556 | 0.001503 | 0.015406 | contig397end | 20990 | 26558 | 1524 |
| MsG0580029519.01 | -3.62221 | 0.001505 | 0.015416 | Chr5 | 97997433 | 97998409 | 612 |
| novel.5912 | -2.25088 | 0.001507 | 0.015429 | Chr6 | 81588812 | 81590837 | 2026 |
| MsG0380012440.01 | -1.63088 | 0.00151 | 0.015457 | Chr3 | 16586275 | 16592977 | 4850 |
| MsG0580028179.01 | -1.82381 | 0.001511 | 0.015462 | Chr5 | 76212633 | 76215987 | 2224 |
| MsG0180003470.01 | -1.79748 | 0.001516 | 0.015512 | Chr1 | 62743220 | 62747149 | 1463 |
| novel.1461 | 2.739683 | 0.001519 | 0.015531 | Chr2 | 54585901 | 54586338 | 438 |
| novel.4016 | -2.68492 | 0.001521 | 0.01555 | Chr4 | 57145563 | 57159964 | 3114 |
| MsG0880045100.01 | 1.58727 | 0.001527 | 0.015597 | Chr8 | 54202258 | 54212318 | 2898 |
| novel.2553 | -3.08446 | 0.001529 | 0.01561 | Chr3 | 85561934 | 85562947 | 771 |
| MsG0780040676.01 | -1.5978 | 0.001533 | 0.015654 | Chr7 | 80984782 | 80994274 | 1584 |
| MsG0180002326.01 | -3.10081 | 0.001536 | 0.015677 | Chr1 | 36737918 | 36741943 | 1272 |
| MsG0880042626.01 | 6.250907 | 0.001542 | 0.015731 | Chr8 | 11610841 | 11616558 | 2916 |
| MsG0780036636.01 | -1.66528 | 0.001544 | 0.015749 | Chr7 | 11022721 | 11030154 | 1179 |
| MsG0380015644.01 | -1.27846 | 0.001545 | 0.015752 | Chr3 | 72960064 | 72965127 | 1477 |
| novel.4389 | 6.084407 | 0.001546 | 0.015752 | Chr5 | 16683331 | 16684300 | 935 |
| novel.696 | 1.639285 | 0.001546 | 0.015752 | Chr1 | 11694457 | 11695969 | 1513 |
| MsG0380016129.01 | -3.53754 | 0.001549 | 0.015777 | Chr3 | 79416045 | 79417117 | 801 |
| novel.2212 | -1.89816 | 0.001558 | 0.015861 | Chr3 | 14833041 | 14878052 | 3177 |
| MsG0480018402.01 | 1.114862 | 0.00156 | 0.015879 | Chr4 | 4397445 | 4399570 | 666 |
| MsG0780038789.01 | 6.11306 | 0.001561 | 0.015883 | Chr7 | 53150000 | 53152758 | 891 |
| novel.8783 | 3.626509 | 0.001562 | 0.015885 | Chr8 | 90580596 | 90584658 | 1182 |
| novel.1555 | 4.321314 | 0.001564 | 0.015904 | Chr2 | 75334613 | 75337487 | 1813 |
| novel.4452 | -5.37624 | 0.001567 | 0.015925 | Chr5 | 25970661 | 25972236 | 965 |
| novel.6273 | 2.854989 | 0.001568 | 0.01593 | Chr6 | 19067738 | 19068680 | 815 |
| novel.3484 | -3.42113 | 0.001571 | 0.015954 | Chr4 | 54771590 | 54772282 | 693 |
| MsG0080048397.01 | -1.64648 | 0.001573 | 0.015974 | contig2end | 987 | 7443 | 1759 |
| MsG0580029505.01 | 2.046708 | 0.001582 | 0.016055 | Chr5 | 97848381 | 97850344 | 1242 |
| novel.5046 | 4.410586 | 0.001582 | 0.016055 | Chr5 | 27062018 | 27064481 | 1306 |
| MsG0580025150.01 | 1.493866 | 0.001584 | 0.01607 | Chr5 | 14424256 | 14447670 | 5327 |
| MsG0180003564.01 | -1.15296 | 0.00159 | 0.016118 | Chr1 | 64254640 | 64265100 | 1693 |
| MsG0880045985.01 | -5.66631 | 0.00159 | 0.01612 | Chr8 | 67284587 | 67285493 | 731 |
| MsG0180005721.01 | -1.25901 | 0.001591 | 0.01612 | Chr1 | 95324185 | 95325516 | 1332 |
| MsG0480022885.01 | 5.800321 | 0.001593 | 0.016135 | Chr4 | 78062913 | 78069090 | 1551 |
| MsG0280010929.01 | -2.56474 | 0.001599 | 0.016188 | Chr2 | 77102582 | 77104119 | 1449 |
| MsG0680033894.01 | 3.836793 | 0.001599 | 0.016188 | Chr6 | 75767294 | 75767938 | 645 |
| MsG0280010735.01 | -1.34872 | 0.001603 | 0.016221 | Chr2 | 74058650 | 74077473 | 1909 |
| MsG0480020784.01 | -2.80954 | 0.001604 | 0.016223 | Chr4 | 48249193 | 48253740 | 2850 |
| MsG0480021740.01 | 1.437391 | 0.001605 | 0.016226 | Chr4 | 63734611 | 63736708 | 1704 |
| MsG0480023569.01 | -2.18509 | 0.001612 | 0.016297 | Chr4 | 87027251 | 87028758 | 1046 |
| novel.6886 | -2.77047 | 0.001614 | 0.016305 | Chr7 | 20942037 | 20945512 | 1417 |
| MsG0780039078.01 | -1.7927 | 0.001614 | 0.016305 | Chr7 | 57591745 | 57593160 | 540 |
| MsG0180004067.01 | -2.02695 | 0.001615 | 0.016306 | Chr1 | 72748696 | 72756741 | 921 |
| MsG0580025893.01 | -6.02344 | 0.001616 | 0.016308 | Chr5 | 26280453 | 26281798 | 739 |
| MsG0780037234.01 | -5.75027 | 0.001618 | 0.01633 | Chr7 | 22217498 | 22223654 | 1794 |
| MsG0180004899.01 | -3.0618 | 0.001621 | 0.016347 | Chr1 | 84156329 | 84156628 | 300 |
| MsG0580025074.01 | -1.46674 | 0.001621 | 0.01635 | Chr5 | 13553572 | 13556813 | 2016 |
| MsG0880043827.01 | -1.45771 | 0.001626 | 0.01639 | Chr8 | 31248332 | 31274484 | 5721 |
| MsG0280006762.01 | -2.45965 | 0.001626 | 0.01639 | Chr2 | 5948420 | 5949217 | 798 |
| novel.3800 | -3.46388 | 0.001628 | 0.016395 | Chr4 | 7428924 | 7434471 | 2073 |
| novel.3592 | -2.56171 | 0.00163 | 0.016411 | Chr4 | 72773820 | 72775944 | 1276 |
| novel.6992 | -2.58242 | 0.001631 | 0.016418 | Chr7 | 44229726 | 44231845 | 1883 |
| novel.4039 | -2.40312 | 0.001632 | 0.016421 | Chr4 | 59850639 | 59852781 | 2055 |
| MsG0780037962.01 | 5.819795 | 0.001633 | 0.016426 | Chr7 | 37135340 | 37138862 | 525 |
| MsG0680032310.01 | -1.8038 | 0.001633 | 0.016426 | Chr6 | 38262147 | 38265476 | 3330 |
| MsG0380016603.01 | -1.10584 | 0.001636 | 0.016442 | Chr3 | 85595760 | 85600378 | 1778 |
| MsG0780041447.01 | -1.39554 | 0.001637 | 0.016447 | Chr7 | 90995818 | 90997279 | 773 |
| MsG0480020879.01 | 1.763079 | 0.001637 | 0.016447 | Chr4 | 50043534 | 50043845 | 312 |
| MsG0180004163.01 | 1.267567 | 0.001638 | 0.016447 | Chr1 | 74257627 | 74258486 | 595 |
| MsG0380017791.01 | -1.04458 | 0.001641 | 0.016477 | Chr3 | 1.01E+08 | 1.01E+08 | 830 |
| novel.8244 | 1.434954 | 0.001644 | 0.016498 | Chr8 | 84825049 | 84826848 | 1800 |
| MsG0580028960.01 | 2.45178 | 0.001653 | 0.016587 | Chr5 | 89619760 | 89621113 | 786 |
| MsG0880042596.01 | 2.217227 | 0.001654 | 0.016587 | Chr8 | 11218275 | 11222721 | 1106 |
| MsG0180001066.01 | -2.44331 | 0.001664 | 0.01668 | Chr1 | 15444787 | 15449557 | 532 |
| MsG0280008162.01 | -2.58534 | 0.001664 | 0.01668 | Chr2 | 26517750 | 26520288 | 1488 |
| MsG0780039951.01 | 1.504548 | 0.001671 | 0.016739 | Chr7 | 71017617 | 71021658 | 1652 |
| MsG0880043425.01 | -2.0127 | 0.001673 | 0.016758 | Chr8 | 24602988 | 24610561 | 2297 |
| novel.9261 | -2.24141 | 0.001674 | 0.016764 | contig581end | 1 | 2405 | 1062 |
| MsG0280011395.01 | -1.28653 | 0.001682 | 0.016837 | Chr2 | 83416033 | 83424871 | 1713 |
| MsG0680034723.01 | -1.98128 | 0.001686 | 0.016868 | Chr6 | 93458415 | 93462233 | 3057 |
| novel.8909 | 5.965279 | 0.00169 | 0.016906 | contig275end | 3439 | 6780 | 1126 |
| MsG0480023604.01 | -2.18027 | 0.001691 | 0.016907 | Chr4 | 87540345 | 87545021 | 1596 |
| MsG0780040441.01 | -1.12504 | 0.001692 | 0.016914 | Chr7 | 77593370 | 77593726 | 357 |
| novel.1000 | -2.09086 | 0.001694 | 0.016922 | Chr1 | 82047247 | 82049319 | 631 |
| MsG0680032275.01 | -2.07183 | 0.001699 | 0.016965 | Chr6 | 37697261 | 37700746 | 3486 |
| MsG0480019739.01 | -2.9519 | 0.001699 | 0.016965 | Chr4 | 26184624 | 26185709 | 1086 |
| novel.2862 | 5.961194 | 0.0017 | 0.016966 | Chr3 | 36144221 | 36175620 | 265 |
| MsG0580029874.01 | -2.01795 | 0.001701 | 0.016966 | Chr5 | 1.03E+08 | 1.03E+08 | 435 |
| novel.761 | -1.52096 | 0.001701 | 0.016966 | Chr1 | 24805598 | 24812201 | 1855 |
| MsG0580028663.01 | 5.245401 | 0.001705 | 0.016994 | Chr5 | 84502930 | 84503244 | 315 |
| MsG0080048600.01 | -1.77241 | 0.001705 | 0.016994 | contig367end | 8452 | 10012 | 681 |
| MsG0880042953.01 | 1.858661 | 0.001705 | 0.016994 | Chr8 | 16573024 | 16574334 | 908 |
| MsG0280009894.01 | 2.346527 | 0.001707 | 0.017001 | Chr2 | 60256534 | 60264252 | 1135 |
| novel.4618 | 1.299549 | 0.001707 | 0.017001 | Chr5 | 61267660 | 61270687 | 748 |
| MsG0180000650.01 | -3.42461 | 0.001717 | 0.017093 | Chr1 | 9217686 | 9217976 | 291 |
| novel.6742 | -4.04832 | 0.001718 | 0.017093 | Chr6 | 1.07E+08 | 1.07E+08 | 644 |
| MsG0180000988.01 | 1.810178 | 0.001721 | 0.017125 | Chr1 | 14125175 | 14127600 | 939 |
| MsG0480018606.01 | -1.66831 | 0.001726 | 0.017163 | Chr4 | 7281456 | 7287972 | 1905 |
| MsG0680035725.01 | 4.08244 | 0.00173 | 0.017197 | Chr6 | 1.11E+08 | 1.11E+08 | 3859 |
| MsG0880044268.01 | 6.204383 | 0.001735 | 0.017246 | Chr8 | 39015735 | 39020933 | 1785 |
| novel.5242 | 4.847768 | 0.001738 | 0.017263 | Chr5 | 69085205 | 69086293 | 872 |
| MsG0280008557.01 | 4.426053 | 0.00174 | 0.017281 | Chr2 | 33926551 | 33927863 | 618 |
| MsG0580029070.01 | -3.17946 | 0.001745 | 0.017321 | Chr5 | 91328083 | 91333185 | 1575 |
| MsG0480021655.01 | 1.130852 | 0.00175 | 0.017367 | Chr4 | 61309867 | 61314375 | 1869 |
| MsG0180000619.01 | 2.397702 | 0.00175 | 0.017367 | Chr1 | 8739055 | 8742293 | 1530 |
| MsG0480019807.01 | 1.609815 | 0.001755 | 0.017411 | Chr4 | 27641551 | 27643849 | 1844 |
| novel.4649 | 2.302487 | 0.001757 | 0.017418 | Chr5 | 68615347 | 68616717 | 911 |
| MsG0480023370.01 | 2.181597 | 0.001758 | 0.017426 | Chr4 | 84271201 | 84273048 | 1629 |
| novel.943 | 5.010629 | 0.001759 | 0.017432 | Chr1 | 66989595 | 66992940 | 1685 |
| MsG0880047747.01 | -1.76427 | 0.001765 | 0.017479 | Chr8 | 90344959 | 90349098 | 1631 |
| MsG0380017708.01 | -1.6882 | 0.001767 | 0.017493 | Chr3 | 99825159 | 99827445 | 1902 |
| MsG0380013606.01 | 1.836016 | 0.001768 | 0.017505 | Chr3 | 41630219 | 41634593 | 1686 |
| MsG0880046441.01 | -1.29935 | 0.001772 | 0.01753 | Chr8 | 73435469 | 73437708 | 1405 |
| MsG0880047233.01 | -1.48701 | 0.001772 | 0.01753 | Chr8 | 83639197 | 83647059 | 4355 |
| MsG0680031646.01 | 1.604038 | 0.001773 | 0.01753 | Chr6 | 23886480 | 23886686 | 207 |
| novel.7225 | -5.9064 | 0.001774 | 0.017533 | Chr7 | 86112116 | 86117173 | 1312 |
| MsG0680034380.01 | 1.833309 | 0.001774 | 0.017533 | Chr6 | 86619624 | 86620640 | 1017 |
| MsG0280011194.01 | -2.36582 | 0.001775 | 0.017533 | Chr2 | 80813847 | 80817249 | 1062 |
| novel.5726 | -3.34318 | 0.001777 | 0.017549 | Chr6 | 39147004 | 39147865 | 698 |
| novel.6368 | -2.42527 | 0.001789 | 0.01766 | Chr6 | 33875984 | 33877707 | 1657 |
| MsG0280008249.01 | 3.37651 | 0.00181 | 0.017868 | Chr2 | 27990902 | 27994366 | 1391 |
| MsG0180006168.01 | -3.0879 | 0.001814 | 0.017898 | Chr1 | 1.01E+08 | 1.01E+08 | 1575 |
| MsG0380012808.01 | 1.983019 | 0.001817 | 0.017916 | Chr3 | 24848646 | 24856938 | 2910 |
| MsG0880045815.01 | -2.15809 | 0.001821 | 0.017953 | Chr8 | 64832018 | 64838516 | 3226 |
| MsG0180000653.01 | -2.4405 | 0.001822 | 0.017954 | Chr1 | 9262762 | 9264791 | 1551 |
| MsG0780040867.01 | 1.136104 | 0.001822 | 0.017954 | Chr7 | 83653373 | 83654233 | 861 |
| novel.2326 | 5.764898 | 0.001826 | 0.017985 | Chr3 | 38725063 | 38726608 | 586 |
| MsG0580029890.01 | 6.14382 | 0.001827 | 0.017986 | Chr5 | 1.03E+08 | 1.03E+08 | 750 |
| MsG0680031406.01 | 1.26532 | 0.00183 | 0.018018 | Chr6 | 19981823 | 19996192 | 2698 |
| MsG0180003879.01 | 2.036252 | 0.001841 | 0.01812 | Chr1 | 69644888 | 69647668 | 1665 |
| novel.1368 | 6.015029 | 0.001844 | 0.018143 | Chr2 | 33870709 | 33871754 | 689 |
| MsG0480022996.01 | 1.322973 | 0.001853 | 0.018223 | Chr4 | 79534194 | 79537581 | 1800 |
| novel.1391 | 5.113219 | 0.001857 | 0.018259 | Chr2 | 37477099 | 37479597 | 1973 |
| novel.1135 | 5.655119 | 0.001862 | 0.018295 | Chr1 | 99891828 | 99893061 | 536 |
| novel.8968 | 2.451291 | 0.001867 | 0.018341 | contig378end | 948 | 1688 | 646 |
| MsG0780038909.01 | 1.076361 | 0.001868 | 0.018341 | Chr7 | 55248161 | 55253248 | 634 |
| MsG0480023607.01 | -2.82736 | 0.001871 | 0.018359 | Chr4 | 87575106 | 87575622 | 414 |
| MsG0580026069.01 | -1.91608 | 0.001871 | 0.018359 | Chr5 | 30136692 | 30142537 | 1096 |
| MsG0480023066.01 | 3.432532 | 0.001871 | 0.018359 | Chr4 | 80361277 | 80365584 | 1197 |
| novel.9277 | 1.078067 | 0.001873 | 0.018368 | contig590end | 1 | 2982 | 1050 |
| MsG0480022114.01 | -1.831 | 0.001874 | 0.018371 | Chr4 | 68391482 | 68396820 | 1769 |
| MsG0780040784.01 | -2.99269 | 0.001878 | 0.018407 | Chr7 | 82395516 | 82404062 | 4431 |
| novel.7703 | -2.04514 | 0.001879 | 0.018407 | Chr7 | 84133414 | 84134319 | 592 |
| novel.487 | -6.06639 | 0.00188 | 0.018414 | Chr1 | 86856362 | 86856646 | 285 |
| MsG0780038309.01 | -2.23237 | 0.001886 | 0.018466 | Chr7 | 43913775 | 43917209 | 1314 |
| MsG0780039783.01 | -2.45659 | 0.001888 | 0.018483 | Chr7 | 69088926 | 69097127 | 3420 |
| MsG0280010893.01 | 4.381011 | 0.001894 | 0.018529 | Chr2 | 76461281 | 76461514 | 234 |
| novel.453 | -2.02894 | 0.001898 | 0.018562 | Chr1 | 82790964 | 82793018 | 2055 |
| novel.1859 | -2.19947 | 0.001898 | 0.018563 | Chr2 | 35691377 | 35696472 | 1368 |
| novel.1048 | -1.60345 | 0.001913 | 0.018692 | Chr1 | 87663971 | 87673049 | 4109 |
| novel.4890 | -3.15284 | 0.001913 | 0.018692 | Chr5 | 1.07E+08 | 1.07E+08 | 1519 |
| MsG0780036101.01 | -3.242 | 0.001914 | 0.018692 | Chr7 | 2608429 | 2609823 | 861 |
| novel.5549 | -5.77876 | 0.001914 | 0.018694 | Chr6 | 6255866 | 6257730 | 1621 |
| MsG0080048763.01 | -1.128 | 0.001917 | 0.018717 | contig427end | 9996 | 33205 | 14883 |
| MsG0580025113.01 | -6.15658 | 0.001923 | 0.018764 | Chr5 | 13989219 | 13995173 | 1035 |
| MsG0780036467.01 | 6.191514 | 0.001923 | 0.018764 | Chr7 | 8579632 | 8582839 | 696 |
| MsG0480023097.01 | 2.258803 | 0.001925 | 0.018774 | Chr4 | 80793999 | 80812063 | 4089 |
| novel.4142 | 5.12017 | 0.001933 | 0.018842 | Chr4 | 75647072 | 75648031 | 480 |
| novel.6066 | -5.71274 | 0.001933 | 0.018842 | Chr6 | 1.05E+08 | 1.05E+08 | 843 |
| MsG0280010404.01 | 1.979085 | 0.001934 | 0.018842 | Chr2 | 69093197 | 69100752 | 2559 |
| MsG0480023428.01 | -1.90961 | 0.001935 | 0.01885 | Chr4 | 85105126 | 85107728 | 849 |
| MsG0880045336.01 | -1.07203 | 0.001941 | 0.018895 | Chr8 | 57677323 | 57677802 | 480 |
| MsG0480018711.01 | -2.19085 | 0.001946 | 0.018937 | Chr4 | 8851243 | 8854596 | 3354 |
| MsG0380011676.01 | 1.338412 | 0.001948 | 0.018953 | Chr3 | 3362664 | 3362972 | 309 |
| MsG0780037206.01 | 5.764898 | 0.001952 | 0.01899 | Chr7 | 21842180 | 21842515 | 336 |
| MsG0380012366.01 | -1.33023 | 0.001956 | 0.019017 | Chr3 | 15535728 | 15535937 | 210 |
| MsG0480018670.01 | -1.75733 | 0.00196 | 0.019056 | Chr4 | 8450257 | 8451414 | 1158 |
| MsG0580027785.01 | -2.25323 | 0.001962 | 0.019062 | Chr5 | 68438817 | 68441341 | 678 |
| novel.1007 | -5.19725 | 0.001965 | 0.019086 | Chr1 | 82693927 | 82695134 | 1208 |
| MsG0680035296.01 | 2.088966 | 0.001966 | 0.019086 | Chr6 | 1.02E+08 | 1.02E+08 | 2695 |
| novel.3773 | -5.03985 | 0.001966 | 0.019086 | Chr4 | 3703265 | 3704474 | 508 |
| MsG0280008580.01 | -1.16759 | 0.001967 | 0.019086 | Chr2 | 34287598 | 34290898 | 1200 |
| MsG0680033699.01 | 1.443621 | 0.001973 | 0.019146 | Chr6 | 71865035 | 71873480 | 2051 |
| MsG0480019393.01 | 1.151702 | 0.001975 | 0.019159 | Chr4 | 19482271 | 19486891 | 949 |
| MsG0780037354.01 | 1.849763 | 0.001977 | 0.019172 | Chr7 | 24139875 | 24143485 | 1279 |
| MsG0280008835.01 | -2.54624 | 0.001985 | 0.019243 | Chr2 | 39785349 | 39797049 | 1953 |
| novel.4523 | -3.01614 | 0.001996 | 0.019337 | Chr5 | 43243399 | 43245768 | 680 |
| novel.3322 | 3.437507 | 0.001996 | 0.019337 | Chr4 | 18583801 | 18585628 | 663 |
| MsG0780035971.01 | -4.16305 | 0.001997 | 0.019339 | Chr7 | 812066 | 818340 | 495 |
| novel.3700 | 1.199586 | 0.002001 | 0.019358 | Chr4 | 86754956 | 86758321 | 1079 |
| MsG0080048733.01 | -5.62709 | 0.002001 | 0.019358 | contig408end | 198167 | 199003 | 837 |
| MsG0580030070.01 | -2.05586 | 0.002001 | 0.019358 | Chr5 | 1.06E+08 | 1.06E+08 | 1374 |
| MsG0280007756.01 | -2.01743 | 0.002009 | 0.019423 | Chr2 | 20125938 | 20127335 | 1398 |
| MsG0380014090.01 | -6.22856 | 0.002018 | 0.019499 | Chr3 | 49533453 | 49540361 | 1541 |
| MsG0880046436.01 | 6.02649 | 0.002018 | 0.019499 | Chr8 | 73355567 | 73358147 | 1042 |
| MsG0480023708.01 | 1.033393 | 0.002023 | 0.019542 | Chr4 | 88824465 | 88825562 | 1098 |
| MsG0180005912.01 | -2.54025 | 0.002028 | 0.019583 | Chr1 | 97464650 | 97468307 | 1754 |
| MsG0380014635.01 | 1.195432 | 0.00203 | 0.019589 | Chr3 | 57573483 | 57580507 | 1215 |
| MsG0880046768.01 | 1.721865 | 0.002033 | 0.019612 | Chr8 | 77835516 | 77836757 | 920 |
| MsG0380014542.01 | 2.740306 | 0.002034 | 0.019612 | Chr3 | 56281508 | 56282140 | 633 |
| MsG0280008280.01 | 3.050406 | 0.002034 | 0.019612 | Chr2 | 28555261 | 28559739 | 3191 |
| MsG0680031720.01 | 1.447529 | 0.002036 | 0.019626 | Chr6 | 25272390 | 25274854 | 915 |
| MsG0780039505.01 | 2.771343 | 0.00204 | 0.019655 | Chr7 | 65012852 | 65013079 | 228 |
| MsG0180000775.01 | -1.62646 | 0.002042 | 0.019668 | Chr1 | 10963845 | 10964279 | 347 |
| novel.5884 | -3.46777 | 0.002044 | 0.019681 | Chr6 | 75982112 | 75984588 | 943 |
| novel.7810 | -2.23944 | 0.002045 | 0.019681 | Chr8 | 3141377 | 3142649 | 638 |
| MsG0280009281.01 | -3.51491 | 0.002051 | 0.019735 | Chr2 | 49331003 | 49332415 | 1413 |
| MsG0580027612.01 | -5.34006 | 0.00206 | 0.019814 | Chr5 | 64322000 | 64324558 | 813 |
| MsG0880043091.01 | -1.70668 | 0.002062 | 0.019825 | Chr8 | 18981770 | 18982183 | 414 |
| MsG0180004997.01 | 1.248197 | 0.002065 | 0.019851 | Chr1 | 85439029 | 85442921 | 1044 |
| MsG0680030907.01 | 1.204696 | 0.002066 | 0.019851 | Chr6 | 11405980 | 11408008 | 1618 |
| MsG0580026658.01 | 2.232273 | 0.002066 | 0.019852 | Chr5 | 41486711 | 41487633 | 387 |
| MsG0580025415.01 | -5.16319 | 0.002068 | 0.019858 | Chr5 | 18252627 | 18257315 | 1398 |
| MsG0880046538.01 | -1.27795 | 0.002068 | 0.019858 | Chr8 | 74511015 | 74535359 | 4200 |
| novel.5760 | -1.55405 | 0.00207 | 0.019858 | Chr6 | 48427548 | 48439291 | 1712 |
| MsG0280010115.01 | 2.207117 | 0.00207 | 0.019858 | Chr2 | 64698572 | 64718689 | 2979 |
| MsG0480018811.01 | -2.74286 | 0.002071 | 0.019861 | Chr4 | 10178355 | 10187258 | 2786 |
| MsG0380014402.01 | -1.28678 | 0.002074 | 0.019877 | Chr3 | 54194716 | 54194940 | 225 |
| MsG0880047112.01 | 1.469422 | 0.002074 | 0.019877 | Chr8 | 82089737 | 82091542 | 780 |
| MsG0680030339.01 | -1.55988 | 0.002075 | 0.019877 | Chr6 | 821299 | 826220 | 1382 |
| MsG0880043498.01 | -1.89514 | 0.002075 | 0.019877 | Chr8 | 25709425 | 25709628 | 204 |
| MsG0180005889.01 | -1.93625 | 0.00208 | 0.019909 | Chr1 | 97175452 | 97182551 | 2604 |
| MsG0480018750.01 | -3.97379 | 0.00208 | 0.019909 | Chr4 | 9377927 | 9378556 | 630 |
| MsG0780037669.01 | -3.35912 | 0.00208 | 0.019909 | Chr7 | 30641439 | 30642074 | 636 |
| MsG0680034919.01 | -3.09838 | 0.002084 | 0.019937 | Chr6 | 96626244 | 96627893 | 453 |
| MsG0480020502.01 | 1.168548 | 0.002089 | 0.019971 | Chr4 | 42510348 | 42520140 | 1190 |
| MsG0780036700.01 | -4.38294 | 0.002089 | 0.019971 | Chr7 | 12099528 | 12102995 | 1932 |
| MsG0180004051.01 | -1.83441 | 0.002095 | 0.020022 | Chr1 | 72504950 | 72514677 | 3332 |
| MsG0780035938.01 | -2.17458 | 0.002097 | 0.020034 | Chr7 | 345299 | 352585 | 1857 |
| MsG0380011744.01 | -1.02784 | 0.002098 | 0.020036 | Chr3 | 4386171 | 4394862 | 1425 |
| MsG0480021826.01 | -1.55096 | 0.002098 | 0.020036 | Chr4 | 64878628 | 64889411 | 2974 |
| MsG0280006953.01 | -2.63255 | 0.002102 | 0.020063 | Chr2 | 8522412 | 8528507 | 1576 |
| MsG0480019151.01 | 2.793706 | 0.002104 | 0.020073 | Chr4 | 15077179 | 15080901 | 1251 |
| MsG0280009956.01 | -1.97997 | 0.002105 | 0.020079 | Chr2 | 61771745 | 61779685 | 2566 |
| MsG0480023525.01 | 1.743785 | 0.002112 | 0.020145 | Chr4 | 86463480 | 86468360 | 2759 |
| novel.8762 | 1.394887 | 0.002117 | 0.020183 | Chr8 | 88334247 | 88341695 | 1715 |
| MsG0380015192.01 | 2.802414 | 0.002118 | 0.020183 | Chr3 | 66523206 | 66523679 | 474 |
| MsG0680030981.01 | 3.89124 | 0.002118 | 0.020183 | Chr6 | 12496529 | 12501139 | 3030 |
| novel.318 | -1.41936 | 0.002124 | 0.020228 | Chr1 | 59802448 | 59804449 | 1074 |
| MsG0380016017.01 | -1.10165 | 0.002125 | 0.020231 | Chr3 | 77921833 | 77928448 | 1847 |
| MsG0680030521.01 | 1.864807 | 0.00213 | 0.020269 | Chr6 | 4579135 | 4581152 | 354 |
| novel.7276 | 1.64244 | 0.00213 | 0.020269 | Chr7 | 93464276 | 93466773 | 666 |
| MsG0680033370.01 | -5.63113 | 0.002131 | 0.020269 | Chr6 | 63339587 | 63340870 | 1284 |
| MsG0180000414.01 | -1.09502 | 0.002138 | 0.020318 | Chr1 | 5680948 | 5698623 | 5448 |
| MsG0480021535.01 | 6.408841 | 0.002138 | 0.020318 | Chr4 | 59223830 | 59233060 | 1518 |
| MsG0580026056.01 | -2.41091 | 0.002138 | 0.020318 | Chr5 | 29915687 | 29917239 | 1488 |
| MsG0580029432.01 | -2.96189 | 0.002141 | 0.020339 | Chr5 | 96913974 | 96920124 | 2151 |
| novel.5535 | -2.88072 | 0.002143 | 0.020346 | Chr6 | 4994324 | 4999079 | 3567 |
| novel.3448 | -1.93639 | 0.002143 | 0.020346 | Chr4 | 46660689 | 46661465 | 777 |
| novel.6948 | 3.408887 | 0.002144 | 0.020346 | Chr7 | 33972883 | 33981016 | 704 |
| MsG0780037184.01 | 1.436988 | 0.002144 | 0.020346 | Chr7 | 21420249 | 21422662 | 841 |
| novel.5043 | -5.61395 | 0.002146 | 0.020357 | Chr5 | 26631060 | 26631510 | 400 |
| MsG0580024646.01 | 4.353337 | 0.00215 | 0.02039 | Chr5 | 7607256 | 7612189 | 1986 |
| novel.2356 | 3.680212 | 0.00216 | 0.020471 | Chr3 | 46923092 | 46931759 | 949 |
| novel.7917 | 5.788372 | 0.002163 | 0.020492 | Chr8 | 21695941 | 21696592 | 572 |
| MsG0180004049.01 | 1.610907 | 0.002163 | 0.020492 | Chr1 | 72484075 | 72485797 | 1551 |
| MsG0680035612.01 | 1.03192 | 0.002166 | 0.020508 | Chr6 | 1.09E+08 | 1.09E+08 | 177 |
| MsG0680033926.01 | -1.50529 | 0.002169 | 0.020531 | Chr6 | 76605046 | 76606578 | 1533 |
| MsG0580024417.01 | 5.920586 | 0.00217 | 0.020534 | Chr5 | 4642684 | 4642995 | 312 |
| MsG0880044173.01 | -1.7432 | 0.002177 | 0.020595 | Chr8 | 36915208 | 36927886 | 3534 |
| MsG0480020077.01 | 5.615288 | 0.00218 | 0.020622 | Chr4 | 33592869 | 33597916 | 1110 |
| novel.4412 | 3.496731 | 0.002182 | 0.020628 | Chr5 | 20984353 | 20985675 | 1323 |
| novel.5083 | 6.1078 | 0.002183 | 0.020628 | Chr5 | 32777133 | 32778583 | 416 |
| novel.937 | -5.67385 | 0.002185 | 0.020647 | Chr1 | 65920961 | 65922058 | 432 |
| MsG0480019543.01 | -1.88222 | 0.00219 | 0.020689 | Chr4 | 21896633 | 21901438 | 489 |
| MsG0380014604.01 | 1.367654 | 0.002191 | 0.020692 | Chr3 | 57121227 | 57124150 | 1675 |
| MsG0180001722.01 | 1.294466 | 0.002195 | 0.020711 | Chr1 | 26057411 | 26060139 | 700 |
| MsG0180001453.01 | -3.37112 | 0.002195 | 0.020711 | Chr1 | 21444905 | 21457575 | 488 |
| MsG0880044318.01 | -1.37591 | 0.002199 | 0.020748 | Chr8 | 39914695 | 39916464 | 387 |
| MsG0180000439.01 | 1.197963 | 0.002201 | 0.02076 | Chr1 | 6035079 | 6035315 | 237 |
| novel.7481 | 6.088525 | 0.002207 | 0.020803 | Chr7 | 42665405 | 42666266 | 862 |
| MsG0080047872.01 | -1.21906 | 0.002211 | 0.020825 | contig131end | 12721 | 15858 | 1875 |
| novel.4899 | -4.30772 | 0.002211 | 0.020825 | Chr5 | 1.08E+08 | 1.08E+08 | 1367 |
| MsG0580028916.01 | 1.628497 | 0.002211 | 0.020825 | Chr5 | 88644385 | 88651499 | 1402 |
| MsG0480022991.01 | -5.12751 | 0.002212 | 0.020826 | Chr4 | 79483658 | 79487879 | 2703 |
| MsG0580024758.01 | 1.814501 | 0.002213 | 0.020826 | Chr5 | 9307042 | 9309691 | 1160 |
| MsG0180003542.01 | -1.80862 | 0.002213 | 0.020826 | Chr1 | 63947626 | 63951826 | 2139 |
| MsG0580026253.01 | 6.025549 | 0.002219 | 0.020875 | Chr5 | 33455162 | 33455512 | 351 |
| MsG0280010134.01 | -3.41449 | 0.002222 | 0.020892 | Chr2 | 65251180 | 65253221 | 1332 |
| novel.8267 | 6.029783 | 0.002222 | 0.020892 | Chr8 | 87887917 | 87888855 | 939 |
| novel.6617 | -4.39155 | 0.002228 | 0.020942 | Chr6 | 87775760 | 87777130 | 843 |
| novel.873 | 1.670654 | 0.00223 | 0.020952 | Chr1 | 52733456 | 52739523 | 1191 |
| MsG0580025191.01 | 2.606152 | 0.002234 | 0.020977 | Chr5 | 15033398 | 15045861 | 2706 |
| MsG0880045378.01 | 1.277152 | 0.002234 | 0.020978 | Chr8 | 58387013 | 58390439 | 1517 |
| novel.4283 | 5.76904 | 0.002237 | 0.021 | Chr5 | 777299 | 778186 | 425 |
| MsG0080048159.01 | 2.424861 | 0.00224 | 0.021014 | contig229end | 10723 | 24600 | 7048 |
| MsG0280006692.01 | -2.05021 | 0.002241 | 0.021019 | Chr2 | 5057470 | 5060941 | 1481 |
| novel.3683 | 3.449857 | 0.002245 | 0.021047 | Chr4 | 84706084 | 84707269 | 1088 |
| MsG0380016390.01 | 1.637548 | 0.002246 | 0.021051 | Chr3 | 82779700 | 82780398 | 534 |
| MsG0180004666.01 | 1.147417 | 0.002247 | 0.021055 | Chr1 | 81011037 | 81011642 | 606 |
| MsG0380016130.01 | 1.447818 | 0.002249 | 0.021066 | Chr3 | 79450165 | 79454917 | 2084 |
| novel.4749 | 3.507667 | 0.002261 | 0.02117 | Chr5 | 86248168 | 86252422 | 2467 |
| MsG0880042557.01 | 3.017688 | 0.002265 | 0.021201 | Chr8 | 10519815 | 10520495 | 681 |
| novel.2038 | -2.58356 | 0.002266 | 0.021211 | Chr2 | 74953818 | 74958586 | 2007 |
| MsG0480018834.01 | -1.97518 | 0.002271 | 0.021241 | Chr4 | 10407670 | 10410015 | 2346 |
| MsG0380016456.01 | 1.249157 | 0.002271 | 0.021241 | Chr3 | 83798583 | 83802806 | 1105 |
| MsG0480020713.01 | 1.323612 | 0.002273 | 0.021251 | Chr4 | 46812142 | 46830365 | 4692 |
| MsG0280006626.01 | -1.2736 | 0.002276 | 0.021273 | Chr2 | 4265903 | 4270818 | 1782 |
| novel.3440 | 2.491392 | 0.002277 | 0.021273 | Chr4 | 43532110 | 43535140 | 983 |
| novel.7201 | -5.69644 | 0.002278 | 0.021281 | Chr7 | 83335925 | 83337089 | 421 |
| MsG0680030704.01 | 5.57833 | 0.002281 | 0.021297 | Chr6 | 7423839 | 7425414 | 1028 |
| MsG0480019833.01 | -3.66786 | 0.002282 | 0.021306 | Chr4 | 28190806 | 28198800 | 1770 |
| MsG0880047192.01 | 1.620731 | 0.002289 | 0.021366 | Chr8 | 83158587 | 83160730 | 984 |
| MsG0380014845.01 | -2.30061 | 0.002291 | 0.021369 | Chr3 | 61102560 | 61120498 | 5424 |
| MsG0880046572.01 | -5.61424 | 0.002291 | 0.021369 | Chr8 | 75009809 | 75014052 | 1149 |
| novel.9252 | -1.48383 | 0.002294 | 0.02139 | contig573end | 2309 | 7294 | 3397 |
| novel.6876 | -5.99238 | 0.002298 | 0.021417 | Chr7 | 19419948 | 19422562 | 780 |
| novel.3947 | 4.805411 | 0.002299 | 0.021417 | Chr4 | 43178448 | 43180621 | 520 |
| novel.8057 | 2.216903 | 0.002317 | 0.021583 | Chr8 | 51782199 | 51786186 | 1258 |
| novel.4395 | 1.219363 | 0.002318 | 0.021583 | Chr5 | 17429317 | 17434011 | 3498 |
| MsG0280006428.01 | 4.635867 | 0.002319 | 0.021584 | Chr2 | 1846711 | 1848072 | 1362 |
| novel.6792 | 1.417742 | 0.002327 | 0.021652 | Chr6 | 1.14E+08 | 1.14E+08 | 2596 |
| MsG0580024583.01 | 1.78786 | 0.00233 | 0.021672 | Chr5 | 6586388 | 6587803 | 894 |
| MsG0680033403.01 | -3.15662 | 0.002337 | 0.021734 | Chr6 | 64106958 | 64108370 | 1413 |
| MsG0880047304.01 | -4.35887 | 0.002338 | 0.021734 | Chr8 | 84386836 | 84387297 | 462 |
| novel.779 | -3.66115 | 0.002344 | 0.021788 | Chr1 | 26709980 | 26711375 | 1396 |
| novel.7112 | 3.088478 | 0.002346 | 0.021799 | Chr7 | 70186295 | 70189197 | 1040 |
| MsG0480018132.01 | -1.83738 | 0.002348 | 0.021805 | Chr4 | 627536 | 632277 | 1841 |
| MsG0480022900.01 | -2.36218 | 0.002348 | 0.021805 | Chr4 | 78279564 | 78286458 | 2077 |
| MsG0680035364.01 | -1.63394 | 0.002349 | 0.021805 | Chr6 | 1.03E+08 | 1.03E+08 | 4210 |
| MsG0780040944.01 | -2.30345 | 0.002356 | 0.02186 | Chr7 | 84621577 | 84625253 | 1527 |
| MsG0380015098.01 | 1.869331 | 0.002356 | 0.02186 | Chr3 | 65178006 | 65181402 | 618 |
| novel.7427 | 5.93487 | 0.00236 | 0.021883 | Chr7 | 29221811 | 29223225 | 642 |
| MsG0880047224.01 | -5.71161 | 0.00236 | 0.021883 | Chr8 | 83556075 | 83556326 | 252 |
| novel.8887 | 6.343506 | 0.002364 | 0.02191 | contig253end | 2560 | 7473 | 478 |
| MsG0180001621.01 | 4.486741 | 0.002366 | 0.02192 | Chr1 | 24268211 | 24276184 | 1398 |
| novel.7323 | 5.620445 | 0.002376 | 0.022005 | Chr7 | 6653189 | 6654329 | 323 |
| novel.3586 | 1.48565 | 0.002384 | 0.022079 | Chr4 | 72236243 | 72242227 | 2772 |
| MsG0080049137.01 | 1.306249 | 0.002386 | 0.022084 | contig92end | 6619 | 7851 | 1233 |
| novel.8111 | -5.97113 | 0.002398 | 0.022195 | Chr8 | 61434374 | 61435369 | 996 |
| MsG0580029769.01 | -1.82189 | 0.002402 | 0.022216 | Chr5 | 1.02E+08 | 1.02E+08 | 2561 |
| novel.3841 | 4.372384 | 0.002402 | 0.022216 | Chr4 | 17963768 | 17964246 | 479 |
| MsG0080048256.01 | 1.435948 | 0.002404 | 0.022226 | contig255end | 521 | 3048 | 619 |
| MsG0880045015.01 | 1.288671 | 0.002405 | 0.022226 | Chr8 | 52776319 | 52776729 | 411 |
| MsG0180005607.01 | -2.30915 | 0.002406 | 0.022227 | Chr1 | 93912030 | 93915270 | 1235 |
| novel.135 | 5.750352 | 0.002409 | 0.022247 | Chr1 | 17451856 | 17452490 | 635 |
| novel.5928 | -5.0133 | 0.002412 | 0.022265 | Chr6 | 84357537 | 84358390 | 547 |
| MsG0280008322.01 | 2.197567 | 0.002415 | 0.022288 | Chr2 | 29205904 | 29206155 | 252 |
| novel.7816 | 6.486524 | 0.002418 | 0.022305 | Chr8 | 4292296 | 4301643 | 280 |
| MsG0280006789.01 | 1.658021 | 0.00242 | 0.022321 | Chr2 | 6222906 | 6223655 | 598 |
| MsG0480021021.01 | -1.20377 | 0.002423 | 0.022337 | Chr4 | 52180709 | 52187164 | 3273 |
| MsG0380016577.01 | 1.941607 | 0.002425 | 0.022351 | Chr3 | 85329226 | 85331411 | 701 |
| MsG0780038503.01 | -1.3715 | 0.002427 | 0.022363 | Chr7 | 47746004 | 47758716 | 2091 |
| MsG0180005984.01 | -2.59206 | 0.002428 | 0.022363 | Chr1 | 98481320 | 98486933 | 1602 |
| MsG0680033339.01 | -1.60201 | 0.002431 | 0.022382 | Chr6 | 62588165 | 62597394 | 2013 |
| MsG0580024406.01 | 5.275105 | 0.002434 | 0.022406 | Chr5 | 4408154 | 4410556 | 1383 |
| MsG0680032261.01 | -2.11518 | 0.002436 | 0.022413 | Chr6 | 37504182 | 37507622 | 3441 |
| MsG0680031471.01 | 5.72194 | 0.002439 | 0.022429 | Chr6 | 20901893 | 20910018 | 1896 |
| novel.7626 | -1.65848 | 0.002439 | 0.022429 | Chr7 | 72719570 | 72723121 | 3450 |
| MsG0380015885.01 | 1.461261 | 0.002443 | 0.022456 | Chr3 | 76282497 | 76290621 | 2297 |
| MsG0580026777.01 | 1.873 | 0.002444 | 0.02246 | Chr5 | 44569594 | 44569908 | 315 |
| MsG0480023761.01 | 1.958093 | 0.002445 | 0.022466 | Chr4 | 89514773 | 89516509 | 803 |
| MsG0480020964.01 | -1.1274 | 0.002446 | 0.022469 | Chr4 | 51271335 | 51274009 | 1185 |
| novel.1977 | 2.799167 | 0.002447 | 0.022472 | Chr2 | 65631566 | 65632311 | 638 |
| novel.4954 | -3.57962 | 0.00246 | 0.022577 | Chr5 | 8772730 | 8776874 | 1511 |
| MsG0380017477.01 | -1.97544 | 0.002461 | 0.022588 | Chr3 | 96693663 | 96696521 | 714 |
| novel.5367 | 2.884513 | 0.002472 | 0.022663 | Chr5 | 89550236 | 89552003 | 1196 |
| MsG0580024553.01 | -1.17491 | 0.002472 | 0.022663 | Chr5 | 6292663 | 6293555 | 768 |
| novel.3804 | 5.658074 | 0.002472 | 0.022663 | Chr4 | 8288351 | 8291525 | 3175 |
| MsG0280007584.01 | -1.89196 | 0.002473 | 0.022663 | Chr2 | 17594992 | 17597134 | 1256 |
| MsG0180000655.01 | -1.74972 | 0.002481 | 0.022734 | Chr1 | 9272688 | 9275184 | 735 |
| novel.7255 | -5.58018 | 0.002493 | 0.022833 | Chr7 | 91333206 | 91336183 | 1342 |
| MsG0880044237.01 | 1.555547 | 0.002495 | 0.022844 | Chr8 | 38219563 | 38219772 | 210 |
| MsG0180004499.01 | -1.2751 | 0.002499 | 0.022873 | Chr1 | 78917870 | 78922608 | 1453 |
| novel.8277 | 1.606654 | 0.002505 | 0.022925 | Chr8 | 88939229 | 88949568 | 4393 |
| MsG0680032303.01 | -3.57596 | 0.002511 | 0.022971 | Chr6 | 38076127 | 38076774 | 648 |
| MsG0280008293.01 | 1.397842 | 0.002517 | 0.023016 | Chr2 | 28776456 | 28779378 | 1080 |
| MsG0880044657.01 | -2.04326 | 0.002519 | 0.023024 | Chr8 | 46797944 | 46799098 | 1155 |
| novel.7822 | -5.01869 | 0.00252 | 0.023024 | Chr8 | 5290572 | 5294095 | 790 |
| MsG0180004631.01 | 1.663473 | 0.00252 | 0.023024 | Chr1 | 80496712 | 80500778 | 2565 |
| MsG0580025504.01 | 2.614327 | 0.002522 | 0.023032 | Chr5 | 19703207 | 19703617 | 411 |
| MsG0180000962.01 | -2.08562 | 0.002523 | 0.023039 | Chr1 | 13788651 | 13797165 | 2241 |
| MsG0580025253.01 | -2.74719 | 0.002524 | 0.02304 | Chr5 | 15914953 | 15917611 | 1689 |
| MsG0680030838.01 | -2.14475 | 0.002525 | 0.02304 | Chr6 | 10262493 | 10268470 | 2592 |
| MsG0680033049.01 | 2.464452 | 0.002534 | 0.023114 | Chr6 | 55499673 | 55507739 | 1819 |
| MsG0480019117.01 | 1.553663 | 0.002543 | 0.023196 | Chr4 | 14755543 | 14761242 | 2994 |
| novel.6644 | -3.83735 | 0.002549 | 0.02324 | Chr6 | 91371967 | 91377681 | 1100 |
| novel.5813 | -2.39322 | 0.002563 | 0.023365 | Chr6 | 61070047 | 61077605 | 3092 |
| MsG0780037534.01 | 1.868237 | 0.002573 | 0.023447 | Chr7 | 27866150 | 27872689 | 1931 |
| MsG0680031461.01 | 6.101472 | 0.002578 | 0.023482 | Chr6 | 20806391 | 20807452 | 792 |
| MsG0180001729.01 | -3.5152 | 0.002579 | 0.023482 | Chr1 | 26142765 | 26146224 | 1443 |
| MsG0680034489.01 | -1.59692 | 0.002581 | 0.023495 | Chr6 | 88463093 | 88465658 | 840 |
| MsG0080048106.01 | 1.195019 | 0.002589 | 0.023565 | contig20end | 4930 | 9646 | 3239 |
| novel.5668 | -5.63094 | 0.002592 | 0.02358 | Chr6 | 26202053 | 26205421 | 1959 |
| MsG0480021510.01 | 1.886946 | 0.002594 | 0.023588 | Chr4 | 58873258 | 58876974 | 1042 |
| novel.272 | 5.986328 | 0.002595 | 0.023591 | Chr1 | 46147308 | 46148783 | 788 |
| MsG0180002462.01 | 2.452651 | 0.002596 | 0.023597 | Chr1 | 38655734 | 38665953 | 525 |
| novel.1705 | -2.33961 | 0.002598 | 0.023605 | Chr2 | 7576593 | 7587277 | 1164 |
| MsG0180004158.01 | -1.43676 | 0.002604 | 0.023652 | Chr1 | 74187198 | 74191863 | 1218 |
| MsG0880045931.01 | -1.65224 | 0.002607 | 0.023675 | Chr8 | 66627175 | 66633937 | 1254 |
| MsG0580024205.01 | -1.13463 | 0.002608 | 0.023678 | Chr5 | 2001306 | 2003077 | 960 |
| novel.8053 | -2.63266 | 0.00261 | 0.023689 | Chr8 | 50987697 | 50997903 | 3431 |
| novel.537 | 3.885202 | 0.002613 | 0.023706 | Chr1 | 95631118 | 95635217 | 764 |
| MsG0280006856.01 | 2.238301 | 0.002614 | 0.023709 | Chr2 | 7123856 | 7132771 | 1494 |
| MsG0780037895.01 | -1.02608 | 0.002615 | 0.023714 | Chr7 | 36060047 | 36068502 | 3766 |
| novel.1650 | 5.889253 | 0.002617 | 0.023722 | Chr2 | 1351240 | 1352543 | 307 |
| MsG0280007551.01 | -3.56249 | 0.002621 | 0.023749 | Chr2 | 17036994 | 17037434 | 441 |
| MsG0680033759.01 | -3.18464 | 0.002624 | 0.023772 | Chr6 | 72933601 | 72935006 | 1227 |
| MsG0780040620.01 | 2.850015 | 0.002627 | 0.023782 | Chr7 | 80127763 | 80128516 | 666 |
| MsG0180006173.01 | 1.028358 | 0.002627 | 0.023782 | Chr1 | 1.01E+08 | 1.01E+08 | 246 |
| novel.3771 | 5.505889 | 0.002631 | 0.023815 | Chr4 | 3517987 | 3524662 | 662 |
| MsG0480021833.01 | 1.582708 | 0.002636 | 0.023848 | Chr4 | 64947521 | 64950206 | 845 |
| MsG0880044988.01 | 1.837695 | 0.002637 | 0.023848 | Chr8 | 52491004 | 52491833 | 636 |
| MsG0380017246.01 | -1.90111 | 0.002637 | 0.023848 | Chr3 | 93997821 | 93999457 | 1389 |
| MsG0580026072.01 | -5.79152 | 0.002642 | 0.02388 | Chr5 | 30188785 | 30189192 | 408 |
| MsG0080048333.01 | -3.01707 | 0.002644 | 0.023896 | contig273end | 20685 | 25116 | 399 |
| novel.2063 | 3.177095 | 0.002646 | 0.0239 | Chr2 | 77966263 | 77967511 | 1218 |
| MsG0580025398.01 | 1.400466 | 0.002652 | 0.023947 | Chr5 | 18033640 | 18039017 | 1600 |
| MsG0380012013.01 | -4.46789 | 0.002659 | 0.024004 | Chr3 | 9499412 | 9501154 | 1210 |
| MsG0080047940.01 | 2.093587 | 0.00266 | 0.024007 | contig152end | 101996 | 113225 | 2505 |
| novel.106 | -1.91747 | 0.002664 | 0.024029 | Chr1 | 13654951 | 13656682 | 1064 |
| MsG0880042537.01 | -2.84575 | 0.002664 | 0.024029 | Chr8 | 10222535 | 10224101 | 1339 |
| MsG0480021872.01 | 1.324605 | 0.002666 | 0.024038 | Chr4 | 65416192 | 65420324 | 971 |
| MsG0480022693.01 | 1.454515 | 0.002672 | 0.024085 | Chr4 | 75550630 | 75551428 | 488 |
| MsG0180005362.01 | 5.895113 | 0.002674 | 0.024101 | Chr1 | 90855527 | 90856545 | 624 |
| MsG0580027113.01 | 1.409548 | 0.00268 | 0.02414 | Chr5 | 53843926 | 53851407 | 1564 |
| MsG0780039096.01 | -2.28108 | 0.00268 | 0.02414 | Chr7 | 57891139 | 57895254 | 3012 |
| novel.2696 | 4.351399 | 0.002683 | 0.024161 | Chr3 | 4084459 | 4085177 | 627 |
| MsG0780036706.01 | -2.30103 | 0.002688 | 0.024193 | Chr7 | 12216031 | 12219171 | 1334 |
| MsG0880043615.01 | 1.253842 | 0.002689 | 0.024199 | Chr8 | 27801908 | 27811196 | 3906 |
| novel.6081 | -2.31184 | 0.002693 | 0.024223 | Chr6 | 1.07E+08 | 1.07E+08 | 1139 |
| novel.1666 | 2.325142 | 0.002703 | 0.024307 | Chr2 | 2621524 | 2623974 | 507 |
| MsG0280007668.01 | 1.211161 | 0.002704 | 0.024311 | Chr2 | 18918623 | 18923121 | 972 |
| MsG0880047436.01 | 1.774967 | 0.002705 | 0.024314 | Chr8 | 86290561 | 86294610 | 1309 |
| MsG0380016261.01 | -2.45016 | 0.002707 | 0.024325 | Chr3 | 81192985 | 81193557 | 573 |
| MsG0680032221.01 | 1.772425 | 0.002711 | 0.024346 | Chr6 | 36657960 | 36661957 | 1707 |
| novel.8776 | -1.00638 | 0.002714 | 0.024363 | Chr8 | 89797223 | 89803868 | 2013 |
| MsG0280007121.01 | -2.38971 | 0.002714 | 0.024363 | Chr2 | 10921036 | 10932177 | 1350 |
| novel.1917 | -1.72969 | 0.002716 | 0.024369 | Chr2 | 51924747 | 51925717 | 442 |
| MsG0380017187.01 | -1.62797 | 0.002716 | 0.02437 | Chr3 | 93128332 | 93136544 | 1683 |
| novel.2999 | 1.244846 | 0.002725 | 0.024439 | Chr3 | 71277688 | 71280864 | 1342 |
| novel.4603 | 6.377968 | 0.002727 | 0.024445 | Chr5 | 57549743 | 57551444 | 436 |
| novel.5922 | -1.70501 | 0.002727 | 0.024445 | Chr6 | 82932204 | 82938141 | 1900 |
| MsG0580025180.01 | -2.59435 | 0.002731 | 0.024462 | Chr5 | 14811712 | 14818235 | 1158 |
| MsG0180000451.01 | -1.5382 | 0.002731 | 0.024462 | Chr1 | 6230745 | 6236332 | 1218 |
| MsG0480020139.01 | 1.088332 | 0.002741 | 0.024547 | Chr4 | 34866298 | 34868690 | 1422 |
| novel.3125 | -2.83128 | 0.002742 | 0.024547 | Chr3 | 90926157 | 90929710 | 1389 |
| MsG0280008072.01 | -1.25194 | 0.002751 | 0.024613 | Chr2 | 25103180 | 25106203 | 1933 |
| MsG0480023735.01 | 2.574824 | 0.002751 | 0.024613 | Chr4 | 89171760 | 89178023 | 1677 |
| MsG0380014934.01 | -2.9008 | 0.00276 | 0.024682 | Chr3 | 62730558 | 62732167 | 771 |
| MsG0680035118.01 | -2.06668 | 0.002765 | 0.024719 | Chr6 | 99210841 | 99221885 | 5013 |
| MsG0280006401.01 | 1.378098 | 0.002767 | 0.024734 | Chr2 | 1371757 | 1372828 | 466 |
| novel.2126 | 2.716128 | 0.002772 | 0.024773 | Chr3 | 1177795 | 1186408 | 1370 |
| MsG0480021554.01 | 2.199344 | 0.002777 | 0.024796 | Chr4 | 59558499 | 59562220 | 1334 |
| MsG0180000288.01 | -1.77291 | 0.002777 | 0.024796 | Chr1 | 3889472 | 3892670 | 1406 |
| MsG0880042443.01 | -1.95704 | 0.002777 | 0.024796 | Chr8 | 8768488 | 8772996 | 3516 |
| MsG0380016056.01 | 1.658497 | 0.00278 | 0.024809 | Chr3 | 78487086 | 78489907 | 1338 |
| MsG0680034646.01 | -2.00662 | 0.002784 | 0.024841 | Chr6 | 92013472 | 92018004 | 3819 |
| MsG0780040886.01 | 3.257903 | 0.002785 | 0.024841 | Chr7 | 83840971 | 83843263 | 1347 |
| novel.8361 | -2.9476 | 0.002789 | 0.024869 | Chr8 | 10896481 | 10900662 | 1935 |
| MsG0280010296.01 | -3.2842 | 0.002795 | 0.024913 | Chr2 | 67547491 | 67551615 | 2502 |
| novel.1131 | 4.952066 | 0.0028 | 0.024949 | Chr1 | 99670121 | 99671473 | 486 |
| MsG0880045943.01 | 5.281125 | 0.002807 | 0.025012 | Chr8 | 66751786 | 66753532 | 951 |
| novel.12 | 2.893951 | 0.002813 | 0.025058 | Chr1 | 1777223 | 1819048 | 4616 |
| MsG0680030994.01 | 2.362848 | 0.002817 | 0.025081 | Chr6 | 12648576 | 12650552 | 1080 |
| MsG0480020937.01 | 3.416873 | 0.00282 | 0.025092 | Chr4 | 50755869 | 50756627 | 759 |
| MsG0180000889.01 | -3.12547 | 0.002825 | 0.025129 | Chr1 | 12816804 | 12818216 | 1413 |
| MsG0580025535.01 | 5.563896 | 0.002832 | 0.025187 | Chr5 | 20156870 | 20160578 | 1875 |
| MsG0680031274.01 | -3.94531 | 0.002844 | 0.025275 | Chr6 | 17224672 | 17224944 | 273 |
| novel.4094 | 4.467098 | 0.002844 | 0.025275 | Chr4 | 68721018 | 68722486 | 908 |
| MsG0880046968.01 | -1.34327 | 0.00285 | 0.025323 | Chr8 | 80503695 | 80511024 | 2541 |
| MsG0780037080.01 | -1.59102 | 0.002854 | 0.025349 | Chr7 | 19220315 | 19232785 | 1758 |
| MsG0780036991.01 | 1.942654 | 0.002855 | 0.025349 | Chr7 | 17635732 | 17639507 | 1111 |
| MsG0380014545.01 | -1.33023 | 0.002855 | 0.025349 | Chr3 | 56328309 | 56350115 | 2535 |
| novel.3079 | 4.780319 | 0.002858 | 0.025366 | Chr3 | 83837431 | 83838906 | 1386 |
| MsG0280010671.01 | 2.930356 | 0.00286 | 0.025375 | Chr2 | 73172547 | 73172822 | 276 |
| MsG0680035383.01 | -1.58841 | 0.002876 | 0.025508 | Chr6 | 1.04E+08 | 1.04E+08 | 1014 |
| novel.2124 | -2.58943 | 0.002888 | 0.025599 | Chr3 | 958272 | 959747 | 1476 |
| novel.5404 | 4.468743 | 0.002892 | 0.025626 | Chr5 | 95299179 | 95300761 | 761 |
| novel.3151 | 1.852241 | 0.002893 | 0.025628 | Chr3 | 95853414 | 95854674 | 1261 |
| novel.142 | 5.785401 | 0.002904 | 0.02572 | Chr1 | 18628779 | 18632842 | 1855 |
| novel.6517 | 1.41984 | 0.002909 | 0.025753 | Chr6 | 65782122 | 65786055 | 2993 |
| novel.7968 | 2.400409 | 0.00291 | 0.025753 | Chr8 | 32365023 | 32367478 | 1414 |
| novel.3632 | -2.33 | 0.002911 | 0.025753 | Chr4 | 78153690 | 78155438 | 674 |
| MsG0680031739.01 | -3.02295 | 0.002914 | 0.025767 | Chr6 | 25732734 | 25734146 | 1413 |
| novel.6664 | 5.80205 | 0.002914 | 0.025767 | Chr6 | 97474437 | 97477989 | 879 |
| MsG0180004632.01 | -4.18509 | 0.002922 | 0.025835 | Chr1 | 80507589 | 80508863 | 558 |
| MsG0580027358.01 | -1.65963 | 0.002925 | 0.025852 | Chr5 | 59003551 | 59005814 | 393 |
| MsG0880044953.01 | 2.80906 | 0.002931 | 0.025896 | Chr8 | 51951359 | 51951655 | 189 |
| novel.8920 | 5.979339 | 0.002933 | 0.025908 | contig295end | 2477 | 4767 | 969 |
| MsG0380016973.01 | -2.86707 | 0.002939 | 0.025954 | Chr3 | 90155466 | 90159263 | 792 |
| MsG0380017446.01 | -4.10581 | 0.00294 | 0.025954 | Chr3 | 96344742 | 96345352 | 327 |
| MsG0180000219.01 | -1.19469 | 0.002941 | 0.025954 | Chr1 | 3018375 | 3021641 | 1450 |
| MsG0380015550.01 | -1.20765 | 0.002943 | 0.025966 | Chr3 | 71724043 | 71731056 | 1883 |
| MsG0880046282.01 | -2.28009 | 0.002945 | 0.025975 | Chr8 | 71518089 | 71519030 | 942 |
| MsG0680032301.01 | -2.28125 | 0.002948 | 0.025989 | Chr6 | 38028611 | 38032207 | 3597 |
| MsG0580025813.01 | -1.7929 | 0.002951 | 0.02601 | Chr5 | 24567153 | 24577268 | 1527 |
| MsG0380013770.01 | 2.309807 | 0.002953 | 0.026023 | Chr3 | 45432874 | 45438022 | 2454 |
| MsG0480018511.01 | -2.35036 | 0.002954 | 0.026023 | Chr4 | 5991299 | 5996580 | 3180 |
| MsG0180000858.01 | -4.51508 | 0.00296 | 0.026063 | Chr1 | 12192013 | 12194106 | 1608 |
| MsG0680032103.01 | -3.55441 | 0.002962 | 0.026075 | Chr6 | 33683043 | 33685016 | 1881 |
| MsG0080048922.01 | 1.008448 | 0.002964 | 0.026082 | contig540end | 25879 | 26319 | 441 |
| novel.3530 | 5.951073 | 0.002968 | 0.02611 | Chr4 | 61897221 | 61900593 | 2579 |
| novel.3290 | 2.56556 | 0.002974 | 0.02616 | Chr4 | 12813752 | 12815973 | 607 |
| novel.5577 | -1.83038 | 0.002983 | 0.02623 | Chr6 | 11689394 | 11691607 | 1384 |
| novel.6852 | -2.26613 | 0.002989 | 0.026271 | Chr7 | 15255973 | 15258991 | 3019 |
| novel.694 | -2.16608 | 0.002989 | 0.026271 | Chr1 | 11611610 | 11613074 | 496 |
| novel.2672 | 2.543311 | 0.002993 | 0.026294 | Chr3 | 231786 | 234623 | 2838 |
| MsG0780037291.01 | -2.84057 | 0.003002 | 0.02636 | Chr7 | 23207115 | 23207430 | 222 |
| novel.9083 | -1.30849 | 0.003002 | 0.02636 | contig473end | 1777 | 7601 | 3754 |
| novel.9226 | 1.104467 | 0.003012 | 0.026436 | contig556end | 14683 | 17260 | 884 |
| novel.6506 | -3.19914 | 0.00302 | 0.026497 | Chr6 | 63290325 | 63290598 | 274 |
| novel.2328 | 1.28528 | 0.003022 | 0.026513 | Chr3 | 40056873 | 40060973 | 1464 |
| MsG0780040391.01 | -3.70388 | 0.003024 | 0.026521 | Chr7 | 76917387 | 76918125 | 285 |
| MsG0480023507.01 | 1.265034 | 0.003025 | 0.026521 | Chr4 | 86239600 | 86239914 | 315 |
| novel.1371 | -1.40163 | 0.003028 | 0.026532 | Chr2 | 34547255 | 34550311 | 809 |
| novel.3874 | 5.725686 | 0.003028 | 0.026532 | Chr4 | 24214241 | 24216193 | 665 |
| MsG0880043180.01 | -1.02241 | 0.003035 | 0.026588 | Chr8 | 20237216 | 20240232 | 2919 |
| novel.6182 | 5.528382 | 0.003049 | 0.026701 | Chr6 | 7448432 | 7451056 | 2070 |
| MsG0280007050.01 | -2.24492 | 0.003052 | 0.026715 | Chr2 | 9931512 | 9940612 | 1563 |
| MsG0780039910.01 | 2.442808 | 0.00306 | 0.026776 | Chr7 | 70337081 | 70350632 | 2958 |
| MsG0780036899.01 | 5.838828 | 0.003061 | 0.026779 | Chr7 | 15931327 | 15932286 | 636 |
| novel.3363 | -3.15333 | 0.003063 | 0.026788 | Chr4 | 25617144 | 25619369 | 2226 |
| MsG0680030472.01 | 2.357979 | 0.003065 | 0.0268 | Chr6 | 3405997 | 3408797 | 486 |
| MsG0680035117.01 | -3.61516 | 0.003067 | 0.02681 | Chr6 | 99208858 | 99209100 | 243 |
| MsG0680031254.01 | 3.760159 | 0.00307 | 0.02683 | Chr6 | 16866292 | 16867329 | 1038 |
| MsG0180003446.01 | 1.656493 | 0.003073 | 0.02685 | Chr1 | 62391634 | 62396283 | 1632 |
| MsG0380017024.01 | 1.742186 | 0.003076 | 0.026858 | Chr3 | 90816233 | 90824593 | 1329 |
| MsG0280009200.01 | 5.983482 | 0.003077 | 0.026858 | Chr2 | 47600914 | 47601966 | 600 |
| MsG0680031569.01 | -1.32328 | 0.003077 | 0.026858 | Chr6 | 22409788 | 22415361 | 2022 |
| MsG0380014600.01 | -1.05657 | 0.003088 | 0.026941 | Chr3 | 57003399 | 57016134 | 3044 |
| novel.3075 | -3.06679 | 0.003089 | 0.026941 | Chr3 | 82752471 | 82753708 | 1007 |
| MsG0280007318.01 | -4.38408 | 0.00309 | 0.026941 | Chr2 | 13535203 | 13542162 | 2646 |
| MsG0480021980.01 | 4.070107 | 0.00309 | 0.026941 | Chr4 | 66772438 | 66778774 | 2577 |
| MsG0080048343.01 | 5.501307 | 0.003092 | 0.026941 | contig279end | 36000 | 37794 | 1200 |
| novel.4576 | 1.249182 | 0.003093 | 0.026941 | Chr5 | 53911216 | 53917696 | 1757 |
| novel.3621 | 5.662162 | 0.003094 | 0.026941 | Chr4 | 77118885 | 77119581 | 697 |
| MsG0480022399.01 | -1.05297 | 0.003095 | 0.026941 | Chr4 | 72044953 | 72048855 | 1385 |
| novel.8447 | 5.640562 | 0.003095 | 0.026941 | Chr8 | 28993049 | 28996467 | 756 |
| MsG0680035893.01 | -2.21906 | 0.0031 | 0.02698 | Chr6 | 1.14E+08 | 1.14E+08 | 1653 |
| MsG0280006524.01 | 1.32086 | 0.003107 | 0.027034 | Chr2 | 3102464 | 3103849 | 1386 |
| MsG0780040245.01 | -1.03325 | 0.00311 | 0.027052 | Chr7 | 74882927 | 74888331 | 1217 |
| MsG0780041034.01 | -3.79955 | 0.003113 | 0.02707 | Chr7 | 85722152 | 85725569 | 1188 |
| MsG0380015975.01 | -4.90194 | 0.003124 | 0.027153 | Chr3 | 77402831 | 77404842 | 1332 |
| MsG0180001313.01 | 5.004404 | 0.003126 | 0.027169 | Chr1 | 19288335 | 19290777 | 567 |
| MsG0380014416.01 | -1.74614 | 0.00313 | 0.027188 | Chr3 | 54544991 | 54548457 | 3121 |
| MsG0380017445.01 | 5.673435 | 0.003133 | 0.027208 | Chr3 | 96337547 | 96343977 | 420 |
| MsG0680030696.01 | -2.19206 | 0.003134 | 0.027208 | Chr6 | 7300716 | 7302119 | 1404 |
| MsG0480018440.01 | -1.45897 | 0.003144 | 0.027283 | Chr4 | 4923716 | 4925842 | 1685 |
| MsG0180005668.01 | -1.12671 | 0.003145 | 0.027283 | Chr1 | 94744636 | 94748301 | 615 |
| MsG0880047271.01 | -1.58367 | 0.003147 | 0.027283 | Chr8 | 84001752 | 84004097 | 924 |
| novel.8851 | 6.289787 | 0.003148 | 0.027283 | contig195end | 12702 | 17663 | 819 |
| MsG0780037064.01 | -5.75205 | 0.003148 | 0.027283 | Chr7 | 18857363 | 18858271 | 909 |
| MsG0880042026.01 | 1.538882 | 0.003148 | 0.027283 | Chr8 | 2773989 | 2778117 | 1600 |
| novel.3792 | -4.80136 | 0.00315 | 0.027293 | Chr4 | 6371977 | 6374026 | 1847 |
| novel.755 | 2.377886 | 0.003157 | 0.027343 | Chr1 | 23026292 | 23031794 | 2411 |
| MsG0880044638.01 | -1.71569 | 0.003157 | 0.027343 | Chr8 | 46081368 | 46084669 | 2454 |
| MsG0580026467.01 | -2.50499 | 0.00316 | 0.027357 | Chr5 | 37935445 | 37940582 | 1287 |
| MsG0380014153.01 | 5.76943 | 0.003163 | 0.027364 | Chr3 | 50538754 | 50541736 | 1703 |
| MsG0880042389.01 | 2.226309 | 0.003163 | 0.027364 | Chr8 | 7982079 | 7982648 | 570 |
| MsG0480022245.01 | 1.32432 | 0.003167 | 0.02739 | Chr4 | 70074807 | 70075070 | 264 |
| MsG0780040053.01 | 1.321567 | 0.003168 | 0.02739 | Chr7 | 72468715 | 72479852 | 3267 |
| novel.440 | 5.491628 | 0.003169 | 0.027391 | Chr1 | 81104405 | 81105066 | 566 |
| novel.2385 | -4.92552 | 0.003171 | 0.027406 | Chr3 | 56129517 | 56130724 | 829 |
| novel.2347 | -1.16009 | 0.003177 | 0.027443 | Chr3 | 45202474 | 46052817 | 10977 |
| MsG0180002433.01 | -1.44255 | 0.003177 | 0.027443 | Chr1 | 38362201 | 38364888 | 2474 |
| MsG0480021016.01 | -2.60685 | 0.003188 | 0.027522 | Chr4 | 52125314 | 52125766 | 453 |
| novel.5029 | 3.645847 | 0.003189 | 0.027522 | Chr5 | 22798563 | 22802839 | 1261 |
| novel.7442 | -3.99427 | 0.003189 | 0.027522 | Chr7 | 32875084 | 32876648 | 743 |
| MsG0280008673.01 | -1.85237 | 0.003196 | 0.02757 | Chr2 | 36128657 | 36132509 | 1644 |
| novel.841 | 5.760265 | 0.003198 | 0.027585 | Chr1 | 42189904 | 42199646 | 623 |
| MsG0180002085.01 | -2.09862 | 0.0032 | 0.027588 | Chr1 | 32512645 | 32525486 | 7749 |
| novel.5287 | -1.34949 | 0.003202 | 0.027597 | Chr5 | 75384269 | 75388747 | 3583 |
| MsG0380018027.01 | -2.57049 | 0.003203 | 0.027598 | Chr3 | 1.03E+08 | 1.03E+08 | 766 |
| MsG0180000415.01 | -1.79865 | 0.003205 | 0.027603 | Chr1 | 5703921 | 5705665 | 1050 |
| MsG0380017781.01 | -1.33662 | 0.003206 | 0.027603 | Chr3 | 1.01E+08 | 1.01E+08 | 1402 |
| novel.9005 | -1.52905 | 0.003206 | 0.027603 | contig431end | 10913 | 35395 | 4895 |
| MsG0880046275.01 | -3.17347 | 0.003213 | 0.027653 | Chr8 | 71405265 | 71411493 | 531 |
| novel.2944 | -5.53247 | 0.003216 | 0.027671 | Chr3 | 60764816 | 60765403 | 588 |
| MsG0480022883.01 | -5.83598 | 0.003221 | 0.027707 | Chr4 | 78047080 | 78049258 | 1098 |
| MsG0380012583.01 | -1.50753 | 0.003224 | 0.027712 | Chr3 | 20170019 | 20173072 | 3054 |
| MsG0580028106.01 | 5.538592 | 0.003224 | 0.027712 | Chr5 | 74669359 | 74670230 | 681 |
| novel.1202 | -2.36124 | 0.003225 | 0.027712 | Chr2 | 6387745 | 6399309 | 1032 |
| MsG0580026416.01 | -1.14098 | 0.003226 | 0.027712 | Chr5 | 36790055 | 36794969 | 985 |
| MsG0780036485.01 | -2.24629 | 0.003227 | 0.027712 | Chr7 | 8803398 | 8809344 | 1330 |
| novel.4633 | -2.28487 | 0.003227 | 0.027712 | Chr5 | 64480558 | 64483220 | 1178 |
| MsG0380012946.01 | 5.667637 | 0.00323 | 0.027726 | Chr3 | 27444237 | 27454329 | 1015 |
| MsG0780040174.01 | 1.4577 | 0.003231 | 0.027727 | Chr7 | 73922453 | 73924329 | 899 |
| MsG0680031027.01 | 5.708869 | 0.003232 | 0.027727 | Chr6 | 13009484 | 13009932 | 207 |
| MsG0180000148.01 | -2.91836 | 0.003233 | 0.027729 | Chr1 | 2025099 | 2027643 | 774 |
| MsG0880044080.01 | -1.59598 | 0.003235 | 0.027729 | Chr8 | 35323568 | 35323974 | 291 |
| novel.7362 | 6.317565 | 0.003235 | 0.027729 | Chr7 | 15447805 | 15450652 | 1775 |
| novel.6327 | 2.964233 | 0.003237 | 0.027739 | Chr6 | 25775190 | 25781393 | 2413 |
| MsG0880045534.01 | 1.438784 | 0.003241 | 0.02777 | Chr8 | 60640481 | 60650554 | 2613 |
| novel.4734 | -3.69187 | 0.003243 | 0.027772 | Chr5 | 83824383 | 83828640 | 1288 |
| MsG0780036068.01 | -1.97098 | 0.003251 | 0.027831 | Chr7 | 2059309 | 2060204 | 519 |
| MsG0380017253.01 | -2.07801 | 0.003258 | 0.027873 | Chr3 | 94081287 | 94082117 | 831 |
| MsG0680035859.01 | 1.274224 | 0.003258 | 0.027873 | Chr6 | 1.14E+08 | 1.14E+08 | 894 |
| MsG0780038865.01 | -5.62796 | 0.00326 | 0.027875 | Chr7 | 54348690 | 54348923 | 234 |
| MsG0480020578.01 | -5.62796 | 0.00326 | 0.027875 | Chr4 | 43687906 | 43692070 | 1560 |
| novel.7554 | 4.781501 | 0.003264 | 0.027895 | Chr7 | 62897856 | 62903470 | 961 |
| MsG0180002335.01 | -1.57023 | 0.003265 | 0.027904 | Chr1 | 36920323 | 36942935 | 5544 |
| novel.2847 | 2.473118 | 0.003278 | 0.028005 | Chr3 | 33453154 | 33472852 | 3644 |
| MsG0080047818.01 | -5.6842 | 0.00328 | 0.028016 | contig113end | 37545 | 50036 | 1122 |
| MsG0380015468.01 | -2.09569 | 0.003283 | 0.02803 | Chr3 | 70600870 | 70605638 | 1535 |
| novel.7127 | 1.504867 | 0.003291 | 0.028093 | Chr7 | 73581648 | 73582369 | 414 |
| novel.6599 | 2.429191 | 0.003294 | 0.028106 | Chr6 | 82754770 | 82755196 | 386 |
| MsG0280007533.01 | 1.497788 | 0.0033 | 0.028147 | Chr2 | 16700945 | 16703926 | 1383 |
| MsG0180004239.01 | 1.343676 | 0.003301 | 0.028152 | Chr1 | 75400931 | 75407378 | 1895 |
| MsG0480023445.01 | -1.52657 | 0.00331 | 0.028216 | Chr4 | 85204548 | 85212199 | 1227 |
| novel.1730 | -5.51336 | 0.003311 | 0.028219 | Chr2 | 11341585 | 11342247 | 411 |
| MsG0180001937.01 | -1.1712 | 0.00332 | 0.028279 | Chr1 | 29575884 | 29578755 | 651 |
| MsG0680034469.01 | 5.439471 | 0.003324 | 0.028307 | Chr6 | 88055038 | 88056687 | 1650 |
| MsG0780036701.01 | -1.25753 | 0.003325 | 0.028309 | Chr7 | 12106684 | 12109341 | 861 |
| novel.7194 | -1.38414 | 0.003329 | 0.028333 | Chr7 | 81389171 | 81394893 | 1958 |
| MsG0880045553.01 | -1.30658 | 0.003334 | 0.028363 | Chr8 | 60994964 | 60996099 | 684 |
| MsG0480021067.01 | -4.16512 | 0.003335 | 0.028368 | Chr4 | 52960094 | 52960555 | 462 |
| MsG0380016847.01 | 1.879348 | 0.003336 | 0.028368 | Chr3 | 88552796 | 88554619 | 1824 |
| MsG0180004766.01 | -2.41185 | 0.003344 | 0.028423 | Chr1 | 82459021 | 82459365 | 345 |
| MsG0880045426.01 | 1.666486 | 0.003345 | 0.028423 | Chr8 | 59007944 | 59010636 | 1395 |
| MsG0580025850.01 | -1.67112 | 0.003346 | 0.028423 | Chr5 | 25140648 | 25151745 | 6390 |
| novel.6667 | -2.07896 | 0.003347 | 0.028423 | Chr6 | 97634531 | 97635673 | 698 |
| MsG0880046062.01 | -1.93504 | 0.003349 | 0.028423 | Chr8 | 68455035 | 68465211 | 2037 |
| MsG0480019123.01 | 3.242402 | 0.00335 | 0.028423 | Chr4 | 14814159 | 14815268 | 1110 |
| MsG0180001664.01 | 1.522041 | 0.00335 | 0.028423 | Chr1 | 24881154 | 24881515 | 362 |
| novel.8074 | 5.681559 | 0.003369 | 0.028575 | Chr8 | 55279634 | 55280912 | 994 |
| novel.1079 | -2.57396 | 0.003371 | 0.028583 | Chr1 | 91780053 | 91781150 | 1098 |
| MsG0180000355.01 | 1.134944 | 0.003374 | 0.028599 | Chr1 | 4830674 | 4834677 | 1452 |
| MsG0380014583.01 | 4.004425 | 0.003381 | 0.028656 | Chr3 | 56769529 | 56773355 | 2370 |
| MsG0780041579.01 | 2.369657 | 0.0034 | 0.028804 | Chr7 | 92661878 | 92669926 | 2283 |
| MsG0880046948.01 | -3.12776 | 0.003409 | 0.028873 | Chr8 | 80293907 | 80294664 | 678 |
| MsG0180005802.01 | 6.16336 | 0.003423 | 0.028986 | Chr1 | 96297612 | 96297824 | 213 |
| MsG0680033593.01 | -5.5945 | 0.003426 | 0.029003 | Chr6 | 69013022 | 69013252 | 231 |
| MsG0680034725.01 | 1.897317 | 0.003434 | 0.029062 | Chr6 | 93467241 | 93468590 | 498 |
| MsG0080048838.01 | 2.907795 | 0.003448 | 0.029164 | contig477end | 7749 | 8327 | 579 |
| MsG0080047803.01 | -3.10457 | 0.003448 | 0.029164 | contig108end | 18858 | 20540 | 907 |
| MsG0880047138.01 | 1.566954 | 0.003452 | 0.029183 | Chr8 | 82548963 | 82554880 | 1833 |
| MsG0780036323.01 | 3.111668 | 0.003453 | 0.029183 | Chr7 | 6443149 | 6445193 | 971 |
| MsG0780038752.01 | 6.224887 | 0.003454 | 0.029189 | Chr7 | 52513947 | 52514810 | 864 |
| MsG0580027954.01 | -1.85531 | 0.003464 | 0.029266 | Chr5 | 71686349 | 71687392 | 1044 |
| novel.1734 | -3.82274 | 0.003466 | 0.029273 | Chr2 | 12397405 | 12399829 | 1409 |
| MsG0180000526.01 | -2.19466 | 0.003469 | 0.029291 | Chr1 | 7425287 | 7426630 | 756 |
| novel.8826 | -1.36244 | 0.003486 | 0.029425 | contig156end | 4489 | 9506 | 1637 |
| MsG0680031243.01 | 1.209909 | 0.00349 | 0.029448 | Chr6 | 16562215 | 16567055 | 669 |
| MsG0880044707.01 | -2.33262 | 0.003491 | 0.029448 | Chr8 | 47986335 | 47996545 | 4881 |
| MsG0380017954.01 | 2.486791 | 0.003493 | 0.029457 | Chr3 | 1.03E+08 | 1.03E+08 | 660 |
| novel.8202 | 1.229669 | 0.003505 | 0.029547 | Chr8 | 77070894 | 77075333 | 3181 |
| MsG0680034479.01 | 5.549959 | 0.003506 | 0.029555 | Chr6 | 88295362 | 88296900 | 1539 |
| MsG0680035512.01 | -2.7482 | 0.003521 | 0.02967 | Chr6 | 1.07E+08 | 1.07E+08 | 813 |
| MsG0180003092.01 | -5.0801 | 0.003524 | 0.029682 | Chr1 | 56431632 | 56435267 | 1899 |
| MsG0480022394.01 | -3.37362 | 0.003548 | 0.029871 | Chr4 | 72010805 | 72012474 | 633 |
| novel.7186 | -4.51831 | 0.003548 | 0.029871 | Chr7 | 80657871 | 80661364 | 3422 |
| novel.3805 | -5.56954 | 0.003557 | 0.029942 | Chr4 | 8321294 | 8322405 | 1112 |
| novel.6989 | -2.69331 | 0.003559 | 0.029947 | Chr7 | 43447850 | 43448743 | 894 |
| MsG0780041699.01 | -4.05611 | 0.003561 | 0.029951 | Chr7 | 94119657 | 94122579 | 1480 |
| MsG0580025806.01 | -5.63054 | 0.003568 | 0.030009 | Chr5 | 24482553 | 24483488 | 936 |
| novel.494 | 4.349108 | 0.003574 | 0.030032 | Chr1 | 87469314 | 87469515 | 202 |
| MsG0580025584.01 | -1.33839 | 0.003574 | 0.030032 | Chr5 | 20853443 | 20864961 | 3526 |
| novel.6291 | 1.007917 | 0.003586 | 0.030123 | Chr6 | 21318775 | 21325357 | 999 |
| novel.6346 | -1.66241 | 0.003588 | 0.030126 | Chr6 | 30089238 | 30092208 | 1365 |
| novel.1839 | 5.054167 | 0.003594 | 0.030173 | Chr2 | 31555394 | 31557864 | 967 |
| MsG0580026726.01 | 1.304663 | 0.003597 | 0.030192 | Chr5 | 43067579 | 43067986 | 408 |
| novel.2022 | 2.278623 | 0.003602 | 0.030219 | Chr2 | 72311183 | 72313142 | 753 |
| MsG0380016578.01 | 1.765526 | 0.003603 | 0.030219 | Chr3 | 85334825 | 85339772 | 1511 |
| novel.4073 | 5.392245 | 0.003608 | 0.030246 | Chr4 | 65923598 | 65925882 | 1244 |
| MsG0080048684.01 | 1.598967 | 0.003611 | 0.030266 | contig39end | 24196 | 29948 | 1502 |
| novel.814 | 5.691804 | 0.00362 | 0.03033 | Chr1 | 34643045 | 34647117 | 1224 |
| MsG0280008460.01 | -1.53325 | 0.003622 | 0.030343 | Chr2 | 32242859 | 32244142 | 1284 |
| MsG0380014530.01 | -5.84818 | 0.003624 | 0.030349 | Chr3 | 56134598 | 56135047 | 450 |
| novel.8474 | 5.356309 | 0.003634 | 0.030415 | Chr8 | 34400336 | 34402816 | 724 |
| MsG0880047456.01 | 1.017358 | 0.003634 | 0.030415 | Chr8 | 86619573 | 86621817 | 773 |
| novel.6185 | -2.45936 | 0.003635 | 0.030415 | Chr6 | 7683990 | 7688450 | 3301 |
| MsG0880047750.01 | 4.127963 | 0.003637 | 0.03042 | Chr8 | 90399380 | 90405439 | 2126 |
| MsG0180006065.01 | 5.758099 | 0.003642 | 0.030455 | Chr1 | 99612395 | 99624997 | 1431 |
| novel.3294 | -1.47995 | 0.003645 | 0.030462 | Chr4 | 13615471 | 13618444 | 580 |
| MsG0780038914.01 | 3.947002 | 0.003645 | 0.030462 | Chr7 | 55300901 | 55307311 | 627 |
| MsG0180003422.01 | -2.10501 | 0.003646 | 0.030463 | Chr1 | 62133388 | 62134992 | 1368 |
| MsG0780040149.01 | -1.38686 | 0.003648 | 0.030465 | Chr7 | 73713404 | 73715765 | 1523 |
| novel.6254 | 4.865875 | 0.003648 | 0.030465 | Chr6 | 16858379 | 16859239 | 424 |
| novel.7576 | -2.67366 | 0.00365 | 0.030472 | Chr7 | 65572941 | 65575905 | 989 |
| novel.4337 | 5.504788 | 0.003659 | 0.030533 | Chr5 | 8886396 | 8889643 | 719 |
| MsG0380014363.01 | -2.03309 | 0.00366 | 0.030533 | Chr3 | 53508270 | 53511748 | 1712 |
| MsG0780038758.01 | 4.116171 | 0.003667 | 0.030581 | Chr7 | 52672092 | 52675375 | 1562 |
| MsG0380016025.01 | -2.25587 | 0.003667 | 0.030581 | Chr3 | 78104646 | 78107461 | 615 |
| novel.901 | 3.177985 | 0.003674 | 0.030631 | Chr1 | 59476810 | 59478732 | 511 |
| MsG0480022303.01 | -1.32891 | 0.003676 | 0.030635 | Chr4 | 70921534 | 70932783 | 1865 |
| novel.2984 | -5.47409 | 0.003688 | 0.030718 | Chr3 | 69059158 | 69060591 | 708 |
| MsG0880047325.01 | 1.085332 | 0.003689 | 0.030718 | Chr8 | 84707247 | 84709005 | 726 |
| novel.9362 | 1.087197 | 0.003689 | 0.030718 | contig648end | 7449 | 10543 | 2232 |
| novel.9134 | 1.395665 | 0.003695 | 0.030755 | contig510end | 14481 | 18790 | 2435 |
| novel.6697 | -5.62185 | 0.003698 | 0.030774 | Chr6 | 1.02E+08 | 1.02E+08 | 632 |
| novel.8845 | 2.291814 | 0.003711 | 0.030874 | contig185end | 7124 | 12670 | 2931 |
| MsG0280006999.01 | 5.931782 | 0.003712 | 0.030878 | Chr2 | 9253469 | 9254576 | 816 |
| novel.6006 | 4.937868 | 0.003724 | 0.030964 | Chr6 | 98461003 | 98468535 | 552 |
| MsG0880047324.01 | 2.265517 | 0.003729 | 0.030996 | Chr8 | 84706435 | 84707190 | 473 |
| novel.8355 | 6.19385 | 0.003732 | 0.031019 | Chr8 | 10179607 | 10180302 | 696 |
| MsG0480018989.01 | 1.146057 | 0.003734 | 0.031022 | Chr4 | 12888964 | 12893070 | 942 |
| MsG0180000230.01 | -1.10215 | 0.003763 | 0.031251 | Chr1 | 3186028 | 3195443 | 1431 |
| MsG0880047168.01 | -4.55397 | 0.00377 | 0.031301 | Chr8 | 82900062 | 82900850 | 789 |
| MsG0180001050.01 | -2.37706 | 0.003772 | 0.031308 | Chr1 | 15225137 | 15226541 | 1311 |
| novel.894 | -5.57005 | 0.003773 | 0.031308 | Chr1 | 57094281 | 57095391 | 442 |
| MsG0580029602.01 | 1.259638 | 0.003782 | 0.03137 | Chr5 | 99262970 | 99265636 | 645 |
| MsG0180002799.01 | 5.737731 | 0.003782 | 0.03137 | Chr1 | 45457280 | 45457786 | 507 |
| MsG0180003949.01 | -3.59783 | 0.003791 | 0.031432 | Chr1 | 70778392 | 70785753 | 578 |
| MsG0380018066.01 | 1.027127 | 0.003801 | 0.031503 | Chr3 | 1.04E+08 | 1.04E+08 | 810 |
| novel.4827 | -5.52532 | 0.003802 | 0.031503 | Chr5 | 99306450 | 99320868 | 2017 |
| MsG0780039542.01 | -3.99365 | 0.003802 | 0.031503 | Chr7 | 65564907 | 65572994 | 784 |
| novel.7033 | -4.00694 | 0.00382 | 0.031639 | Chr7 | 53934368 | 53934988 | 621 |
| MsG0480022702.01 | -2.79874 | 0.003828 | 0.031696 | Chr4 | 75625903 | 75627846 | 1068 |
| MsG0580027745.01 | 2.147674 | 0.003846 | 0.031833 | Chr5 | 67892794 | 67904992 | 8856 |
| MsG0280009901.01 | -2.11571 | 0.003847 | 0.031833 | Chr2 | 60406384 | 60420202 | 1153 |
| MsG0180001203.01 | 3.997087 | 0.003849 | 0.031846 | Chr1 | 17307487 | 17309902 | 579 |
| MsG0680031848.01 | -1.91179 | 0.00385 | 0.031846 | Chr6 | 28129823 | 28132719 | 2079 |
| novel.3289 | -1.84547 | 0.003855 | 0.031879 | Chr4 | 12564561 | 12569794 | 1836 |
| MsG0480018784.01 | -1.69917 | 0.003859 | 0.031897 | Chr4 | 9714606 | 9719193 | 1911 |
| MsG0480021175.01 | 1.373759 | 0.003864 | 0.03193 | Chr4 | 54706886 | 54710106 | 1834 |
| MsG0380012779.01 | 1.603037 | 0.003866 | 0.03193 | Chr3 | 24441633 | 24441941 | 309 |
| MsG0380017652.01 | 2.323189 | 0.003869 | 0.031945 | Chr3 | 99104456 | 99105490 | 1035 |
| novel.8461 | 2.02455 | 0.003871 | 0.031957 | Chr8 | 31394166 | 31397580 | 855 |
| novel.6072 | -5.42678 | 0.003876 | 0.031985 | Chr6 | 1.06E+08 | 1.06E+08 | 737 |
| MsG0480023954.01 | -5.44139 | 0.003877 | 0.031988 | Chr4 | 91644046 | 91644441 | 396 |
| MsG0380012702.01 | 1.314017 | 0.003881 | 0.032009 | Chr3 | 22970560 | 22971222 | 663 |
| MsG0480020910.01 | -1.80781 | 0.003885 | 0.032037 | Chr4 | 50451088 | 50457870 | 846 |
| MsG0280010517.01 | -1.13726 | 0.00389 | 0.032069 | Chr2 | 70836860 | 70839473 | 1465 |
| MsG0580025632.01 | -2.95363 | 0.0039 | 0.032142 | Chr5 | 21505920 | 21515193 | 1566 |
| MsG0680032422.01 | 4.099523 | 0.003905 | 0.032175 | Chr6 | 40825840 | 40827242 | 1350 |
| MsG0180001922.01 | -1.2444 | 0.003918 | 0.032259 | Chr1 | 29414271 | 29415911 | 1641 |
| MsG0180002283.01 | 2.597876 | 0.003919 | 0.032259 | Chr1 | 36232982 | 36237671 | 1632 |
| MsG0580027133.01 | 1.372503 | 0.003924 | 0.032292 | Chr5 | 54149102 | 54152219 | 1371 |
| novel.8086 | 1.492819 | 0.003925 | 0.032292 | Chr8 | 57370895 | 57373672 | 709 |
| MsG0880044524.01 | 2.133464 | 0.003931 | 0.032333 | Chr8 | 43819676 | 43820098 | 330 |
| MsG0880046474.01 | 1.920698 | 0.003932 | 0.032335 | Chr8 | 73810322 | 73811797 | 1476 |
| MsG0480020896.01 | -1.92362 | 0.003934 | 0.032336 | Chr4 | 50315600 | 50317317 | 624 |
| MsG0580025207.01 | 1.249895 | 0.003938 | 0.032363 | Chr5 | 15316078 | 15326050 | 3303 |
| MsG0180004662.01 | 1.013857 | 0.003941 | 0.032378 | Chr1 | 80973137 | 80979420 | 2347 |
| novel.6524 | 1.459528 | 0.003946 | 0.032409 | Chr6 | 67550507 | 67551500 | 664 |
| MsG0280010818.01 | 1.252086 | 0.003947 | 0.03241 | Chr2 | 75427438 | 75430978 | 1698 |
| MsG0480023941.01 | 1.134958 | 0.003951 | 0.032433 | Chr4 | 91508103 | 91510271 | 813 |
| MsG0880044252.01 | -5.54009 | 0.003953 | 0.032439 | Chr8 | 38589904 | 38590518 | 615 |
| MsG0480018525.01 | -1.13697 | 0.003959 | 0.032482 | Chr4 | 6145115 | 6145786 | 672 |
| novel.5937 | -2.6366 | 0.00396 | 0.032483 | Chr6 | 85559737 | 85562714 | 1538 |
| MsG0080048988.01 | 2.909925 | 0.003962 | 0.032486 | contig588end | 23080 | 23316 | 237 |
| MsG0780040161.01 | -1.40316 | 0.003963 | 0.032486 | Chr7 | 73847508 | 73853065 | 1064 |
| MsG0380011927.01 | 1.706056 | 0.003967 | 0.032514 | Chr3 | 8069165 | 8069455 | 291 |
| novel.7025 | -6.23736 | 0.003978 | 0.032596 | Chr7 | 51743113 | 51748928 | 616 |
| MsG0880042914.01 | -1.87295 | 0.003984 | 0.032632 | Chr8 | 15834982 | 15835926 | 945 |
| novel.7642 | 1.124462 | 0.003992 | 0.032688 | Chr7 | 75065956 | 75070645 | 4167 |
| MsG0480022003.01 | 1.984243 | 0.004002 | 0.03276 | Chr4 | 66968304 | 66970359 | 1029 |
| novel.5003 | -1.24309 | 0.004008 | 0.032804 | Chr5 | 16502834 | 16506426 | 3593 |
| novel.3832 | 2.482055 | 0.004011 | 0.032823 | Chr4 | 16821427 | 16822829 | 1403 |
| MsG0480018142.01 | -2.45447 | 0.004023 | 0.03291 | Chr4 | 818425 | 826380 | 2086 |
| novel.1014 | 5.501042 | 0.004026 | 0.032923 | Chr1 | 83966027 | 83967605 | 410 |
| novel.6165 | -2.95316 | 0.004028 | 0.032924 | Chr6 | 5018350 | 5023302 | 1431 |
| MsG0480023320.01 | -2.11552 | 0.004028 | 0.032924 | Chr4 | 83701443 | 83701805 | 363 |
| MsG0880046363.01 | 3.081026 | 0.004037 | 0.032985 | Chr8 | 72415508 | 72418156 | 513 |
| novel.4746 | 5.515558 | 0.004042 | 0.033014 | Chr5 | 85832988 | 85833753 | 766 |
| MsG0380016540.01 | 1.027668 | 0.004053 | 0.033095 | Chr3 | 84932943 | 84938489 | 2080 |
| MsG0680031194.01 | -2.53298 | 0.004064 | 0.033178 | Chr6 | 15799396 | 15801376 | 312 |
| MsG0880045194.01 | 2.064843 | 0.004065 | 0.033179 | Chr8 | 55600561 | 55603092 | 2355 |
| MsG0680030297.01 | 1.559961 | 0.004069 | 0.033201 | Chr6 | 271913 | 272242 | 330 |
| MsG0780038963.01 | -2.61497 | 0.00407 | 0.033201 | Chr7 | 55925199 | 55928474 | 1913 |
| novel.3973 | 3.083753 | 0.004072 | 0.033203 | Chr4 | 48822794 | 48823381 | 588 |
| MsG0180004129.01 | 1.458177 | 0.004072 | 0.033203 | Chr1 | 73743674 | 73745451 | 724 |
| MsG0580027827.01 | -1.9327 | 0.004074 | 0.033208 | Chr5 | 69226616 | 69229522 | 2907 |
| novel.3427 | 5.753695 | 0.004082 | 0.033262 | Chr4 | 39386196 | 39388168 | 642 |
| MsG0280006695.01 | 1.232533 | 0.004084 | 0.033268 | Chr2 | 5091435 | 5099625 | 2171 |
| MsG0280008419.01 | 1.063797 | 0.004092 | 0.033322 | Chr2 | 31506194 | 31519638 | 2308 |
| MsG0280009818.01 | -3.18789 | 0.004098 | 0.033362 | Chr2 | 58762834 | 58785432 | 9438 |
| novel.1276 | 3.105111 | 0.004103 | 0.033396 | Chr2 | 17110202 | 17112032 | 1831 |
| MsG0880043639.01 | 1.381055 | 0.004107 | 0.033424 | Chr8 | 28235363 | 28236126 | 300 |
| novel.4740 | -2.5693 | 0.004109 | 0.03343 | Chr5 | 84951039 | 84954405 | 1014 |
| MsG0780039527.01 | -1.66146 | 0.004122 | 0.033521 | Chr7 | 65289905 | 65293446 | 1243 |
| MsG0580026270.01 | -1.95254 | 0.004126 | 0.033537 | Chr5 | 33788394 | 33792603 | 1005 |
| novel.8405 | 1.269413 | 0.004128 | 0.033537 | Chr8 | 20071955 | 20073714 | 965 |
| MsG0380017859.01 | -1.23377 | 0.004128 | 0.033537 | Chr3 | 1.02E+08 | 1.02E+08 | 2578 |
| MsG0680034998.01 | 3.364582 | 0.004128 | 0.033537 | Chr6 | 97490122 | 97503101 | 4433 |
| MsG0880047651.01 | -3.26063 | 0.00413 | 0.033545 | Chr8 | 89210534 | 89215537 | 2422 |
| MsG0880042382.01 | 1.384876 | 0.00414 | 0.033617 | Chr8 | 7854634 | 7867438 | 2763 |
| novel.2820 | 5.623286 | 0.004146 | 0.033655 | Chr3 | 28206558 | 28208219 | 1031 |
| MsG0480023299.01 | 1.823484 | 0.004147 | 0.033655 | Chr4 | 83499012 | 83512408 | 2061 |
| MsG0680033884.01 | 1.817484 | 0.004148 | 0.033655 | Chr6 | 75631322 | 75631906 | 585 |
| novel.2319 | 2.966982 | 0.00415 | 0.033662 | Chr3 | 37103990 | 37107959 | 1716 |
| MsG0380017866.01 | 1.081844 | 0.004158 | 0.033718 | Chr3 | 1.02E+08 | 1.02E+08 | 1288 |
| MsG0680035349.01 | 1.500462 | 0.004164 | 0.033751 | Chr6 | 1.03E+08 | 1.03E+08 | 279 |
| MsG0780038532.01 | 5.454371 | 0.004169 | 0.033787 | Chr7 | 48250990 | 48256387 | 1953 |
| novel.1949 | -2.08729 | 0.004171 | 0.033796 | Chr2 | 60925726 | 60933225 | 1275 |
| MsG0380012690.01 | 3.838136 | 0.004175 | 0.033817 | Chr3 | 22803474 | 22804616 | 1143 |
| novel.992 | 3.833723 | 0.004187 | 0.033902 | Chr1 | 79787643 | 79790491 | 683 |
| novel.2292 | 3.012492 | 0.004191 | 0.033924 | Chr3 | 29977858 | 29978909 | 1052 |
| MsG0280011343.01 | -2.16311 | 0.004194 | 0.03394 | Chr2 | 82681749 | 82682511 | 501 |
| novel.6334 | 1.996523 | 0.004201 | 0.033991 | Chr6 | 26991570 | 26994856 | 3287 |
| MsG0680035694.01 | -1.87247 | 0.004205 | 0.034012 | Chr6 | 1.11E+08 | 1.11E+08 | 339 |
| novel.159 | -1.64615 | 0.004215 | 0.034082 | Chr1 | 22544637 | 22547318 | 1564 |
| MsG0680033187.01 | -4.32494 | 0.004217 | 0.034094 | Chr6 | 58987937 | 58990251 | 450 |
| MsG0680035309.01 | -1.78453 | 0.004219 | 0.034094 | Chr6 | 1.02E+08 | 1.02E+08 | 1363 |
| novel.2299 | -1.85179 | 0.004224 | 0.034128 | Chr3 | 31053058 | 31054157 | 448 |
| MsG0680034131.01 | 1.661763 | 0.004226 | 0.034138 | Chr6 | 81354960 | 81358221 | 627 |
| MsG0480019374.01 | -1.61512 | 0.004246 | 0.034281 | Chr4 | 19212230 | 19224274 | 4326 |
| MsG0080048621.01 | 1.002518 | 0.004246 | 0.034281 | contig374end | 101917 | 105590 | 995 |
| MsG0780038419.01 | 2.529352 | 0.004257 | 0.034356 | Chr7 | 46479335 | 46480826 | 1470 |
| MsG0680031114.01 | 2.371598 | 0.004262 | 0.034388 | Chr6 | 14688707 | 14692873 | 689 |
| MsG0580029691.01 | -1.46583 | 0.004264 | 0.034394 | Chr5 | 1.01E+08 | 1.01E+08 | 1191 |
| MsG0480021055.01 | 1.211055 | 0.004267 | 0.034408 | Chr4 | 52730873 | 52731280 | 408 |
| novel.1218 | -4.79939 | 0.004268 | 0.034408 | Chr2 | 8369682 | 8387565 | 1317 |
| novel.2287 | -2.7024 | 0.004282 | 0.034512 | Chr3 | 28183819 | 28188865 | 4041 |
| novel.450 | -3.46929 | 0.00429 | 0.034565 | Chr1 | 82450501 | 82452521 | 1989 |
| MsG0580028614.01 | -1.6981 | 0.004291 | 0.034565 | Chr5 | 83416971 | 83421051 | 2589 |
| MsG0780040190.01 | -1.03815 | 0.004294 | 0.034579 | Chr7 | 74144152 | 74151452 | 1539 |
| MsG0280009804.01 | -2.76264 | 0.004295 | 0.034579 | Chr2 | 58382075 | 58384545 | 1334 |
| MsG0480018412.01 | -3.43807 | 0.0043 | 0.034612 | Chr4 | 4579591 | 4580349 | 759 |
| MsG0780040094.01 | -1.16603 | 0.00431 | 0.034683 | Chr7 | 72988788 | 72991855 | 1512 |
| novel.2198 | -4.43499 | 0.004322 | 0.03477 | Chr3 | 12926267 | 12944142 | 1195 |
| novel.3242 | -1.29339 | 0.004329 | 0.034818 | Chr4 | 4865314 | 4869745 | 1020 |
| novel.2393 | -5.51637 | 0.004335 | 0.034856 | Chr3 | 58146145 | 58148542 | 280 |
| novel.6671 | -1.74144 | 0.004337 | 0.034861 | Chr6 | 98828743 | 98833124 | 3267 |
| novel.7737 | 4.117653 | 0.004345 | 0.034915 | Chr7 | 89117683 | 89119129 | 641 |
| novel.1396 | 5.784138 | 0.004354 | 0.034969 | Chr2 | 38392442 | 38394591 | 543 |
| MsG0680030561.01 | -1.58792 | 0.004358 | 0.034992 | Chr6 | 5293821 | 5295079 | 591 |
| MsG0180003541.01 | -1.10221 | 0.004362 | 0.035013 | Chr1 | 63933060 | 63939800 | 622 |
| MsG0580024382.01 | -1.50437 | 0.004365 | 0.035028 | Chr5 | 4072883 | 4073396 | 318 |
| MsG0280007016.01 | 4.044114 | 0.00437 | 0.035049 | Chr2 | 9469659 | 9485980 | 1443 |
| MsG0880042289.01 | 2.080772 | 0.00437 | 0.035049 | Chr8 | 6554956 | 6558901 | 2022 |
| novel.2950 | -2.90585 | 0.004382 | 0.035134 | Chr3 | 61090150 | 61095190 | 2247 |
| MsG0180003683.01 | 5.45802 | 0.004386 | 0.03516 | Chr1 | 66565748 | 66574268 | 774 |
| MsG0680033913.01 | 2.869146 | 0.004388 | 0.035163 | Chr6 | 76263711 | 76272109 | 654 |
| MsG0580028274.01 | -1.63809 | 0.00439 | 0.03517 | Chr5 | 77675039 | 77700782 | 6239 |
| MsG0380017656.01 | 1.473839 | 0.004392 | 0.035178 | Chr3 | 99152796 | 99153224 | 429 |
| MsG0680030556.01 | -3.64506 | 0.004399 | 0.035229 | Chr6 | 5260782 | 5268149 | 1623 |
| novel.6624 | -1.59935 | 0.004407 | 0.035277 | Chr6 | 88473164 | 88475296 | 1607 |
| MsG0280010792.01 | 1.021257 | 0.004408 | 0.035277 | Chr2 | 75154094 | 75157824 | 1100 |
| novel.1488 | 1.365629 | 0.00441 | 0.035285 | Chr2 | 61681766 | 61701473 | 567 |
| MsG0280007035.01 | -1.29333 | 0.004418 | 0.03533 | Chr2 | 9722845 | 9729735 | 2440 |
| MsG0380015324.01 | -1.29485 | 0.004418 | 0.03533 | Chr3 | 68452660 | 68455896 | 1487 |
| MsG0380015646.01 | 1.744953 | 0.004425 | 0.035377 | Chr3 | 73002045 | 73007079 | 1589 |
| MsG0880043195.01 | -2.24303 | 0.004426 | 0.035377 | Chr8 | 20436408 | 20440565 | 1361 |
| novel.7969 | 5.953712 | 0.004431 | 0.035404 | Chr8 | 33239031 | 33244081 | 360 |
| MsG0480018093.01 | 2.061646 | 0.004433 | 0.03541 | Chr4 | 151039 | 154126 | 990 |
| MsG0380013353.01 | -1.83838 | 0.004444 | 0.035493 | Chr3 | 35677397 | 35680962 | 882 |
| MsG0480021878.01 | -1.23823 | 0.004446 | 0.035495 | Chr4 | 65480243 | 65480880 | 534 |
| MsG0180003465.01 | -2.04283 | 0.004453 | 0.035534 | Chr1 | 62667787 | 62668901 | 876 |
| MsG0280006989.01 | 1.708876 | 0.004454 | 0.035534 | Chr2 | 9055771 | 9059825 | 777 |
| MsG0280007632.01 | -3.5989 | 0.004455 | 0.035534 | Chr2 | 18344920 | 18351967 | 1920 |
| MsG0780041250.01 | -1.05684 | 0.004455 | 0.035534 | Chr7 | 88801041 | 88805830 | 1497 |
| MsG0280010250.01 | -6.00613 | 0.004461 | 0.035568 | Chr2 | 66755954 | 66756418 | 465 |
| novel.2488 | 3.579465 | 0.004465 | 0.035592 | Chr3 | 76399582 | 76401735 | 892 |
| MsG0180000885.01 | -1.57894 | 0.004469 | 0.035613 | Chr1 | 12726486 | 12731048 | 798 |
| MsG0880042533.01 | -1.62606 | 0.004474 | 0.035643 | Chr8 | 10168692 | 10170510 | 682 |
| MsG0280008297.01 | -2.62174 | 0.004476 | 0.035653 | Chr2 | 28821465 | 28822950 | 642 |
| MsG0680031369.01 | 1.752582 | 0.00449 | 0.035752 | Chr6 | 19111556 | 19137136 | 2367 |
| MsG0180004203.01 | -1.59721 | 0.004506 | 0.035861 | Chr1 | 74813159 | 74817967 | 1454 |
| MsG0380017683.01 | -1.18381 | 0.004507 | 0.035861 | Chr3 | 99518423 | 99519406 | 984 |
| MsG0380013078.01 | -1.9472 | 0.004515 | 0.035916 | Chr3 | 29966798 | 29970525 | 1641 |
| novel.3317 | 3.956971 | 0.004519 | 0.035932 | Chr4 | 16450415 | 16454107 | 388 |
| MsG0880044342.01 | -1.42198 | 0.004528 | 0.035988 | Chr8 | 40320298 | 40324849 | 1914 |
| MsG0880043415.01 | -1.12897 | 0.004533 | 0.036017 | Chr8 | 24488151 | 24494847 | 2222 |
| MsG0180006094.01 | -1.08623 | 0.004534 | 0.036019 | Chr1 | 1E+08 | 1E+08 | 2622 |
| novel.3557 | 1.083065 | 0.004543 | 0.036077 | Chr4 | 66798772 | 66800677 | 1821 |
| MsG0280007460.01 | 5.425695 | 0.004554 | 0.036153 | Chr2 | 15793369 | 15794418 | 1050 |
| MsG0480022179.01 | -1.83542 | 0.004556 | 0.036162 | Chr4 | 69333395 | 69337195 | 1446 |
| MsG0380012330.01 | -2.90951 | 0.00456 | 0.03618 | Chr3 | 15012697 | 15013095 | 399 |
| MsG0180004547.01 | -1.95916 | 0.004571 | 0.036251 | Chr1 | 79460878 | 79462016 | 933 |
| novel.1549 | -2.37479 | 0.004576 | 0.036279 | Chr2 | 73812168 | 73813602 | 538 |
| MsG0580026052.01 | 3.088023 | 0.004584 | 0.036334 | Chr5 | 29871717 | 29873578 | 1603 |
| MsG0380011896.01 | 1.412018 | 0.004606 | 0.036501 | Chr3 | 7607277 | 7607645 | 369 |
| novel.668 | -2.94743 | 0.00461 | 0.036525 | Chr1 | 9616626 | 9618193 | 882 |
| MsG0680033737.01 | -5.37082 | 0.004612 | 0.036528 | Chr6 | 72431608 | 72432703 | 831 |
| MsG0880042317.01 | -4.2231 | 0.004614 | 0.036534 | Chr8 | 7002384 | 7004630 | 714 |
| MsG0180005132.01 | -1.58822 | 0.004619 | 0.036566 | Chr1 | 87434931 | 87437895 | 1456 |
| novel.2210 | -5.38726 | 0.004623 | 0.036589 | Chr3 | 14691600 | 14691972 | 373 |
| MsG0280008324.01 | -1.31322 | 0.004629 | 0.036621 | Chr2 | 29239695 | 29245559 | 898 |
| novel.8549 | 5.458978 | 0.00463 | 0.036623 | Chr8 | 52012811 | 52013757 | 492 |
| MsG0380016409.01 | -6.96713 | 0.004647 | 0.03675 | Chr3 | 83010906 | 83013329 | 1260 |
| novel.2494 | 2.770306 | 0.004653 | 0.036775 | Chr3 | 77621787 | 77622154 | 368 |
| novel.1169 | -5.51199 | 0.004656 | 0.036785 | Chr2 | 1518017 | 1518436 | 234 |
| MsG0680032277.01 | -4.06969 | 0.00467 | 0.036887 | Chr6 | 37737593 | 37739209 | 1431 |
| MsG0880046321.01 | 1.107535 | 0.004675 | 0.036895 | Chr8 | 71990995 | 71996165 | 1763 |
| novel.7103 | 1.577609 | 0.004676 | 0.036895 | Chr7 | 67916180 | 67919754 | 1413 |
| MsG0680033750.01 | -3.5199 | 0.004676 | 0.036895 | Chr6 | 72656720 | 72659858 | 366 |
| novel.4547 | 1.355458 | 0.004677 | 0.036895 | Chr5 | 46278683 | 46281350 | 924 |
| MsG0680035325.01 | -1.79452 | 0.004677 | 0.036895 | Chr6 | 1.02E+08 | 1.02E+08 | 2388 |
| MsG0280010272.01 | -1.18193 | 0.00468 | 0.036908 | Chr2 | 67084367 | 67089774 | 831 |
| MsG0480020797.01 | -1.13166 | 0.004683 | 0.036918 | Chr4 | 48493988 | 48495990 | 1211 |
| novel.5770 | -1.9516 | 0.004684 | 0.036918 | Chr6 | 49268305 | 49271590 | 2449 |
| MsG0880041884.01 | 4.236077 | 0.004691 | 0.036969 | Chr8 | 683956 | 684630 | 675 |
| MsG0380013813.01 | -3.15676 | 0.004694 | 0.036977 | Chr3 | 46032297 | 46038526 | 2835 |
| MsG0180000525.01 | -2.44147 | 0.004696 | 0.036986 | Chr1 | 7405884 | 7409048 | 918 |
| novel.3868 | -2.61102 | 0.004706 | 0.037056 | Chr4 | 22058646 | 22059487 | 842 |
| MsG0180003991.01 | 1.010339 | 0.004709 | 0.037066 | Chr1 | 71462525 | 71469197 | 1029 |
| MsG0480022508.01 | -2.74991 | 0.004713 | 0.037087 | Chr4 | 73405750 | 73407347 | 1527 |
| MsG0880046837.01 | -1.27044 | 0.004721 | 0.03714 | Chr8 | 78949474 | 78950547 | 1074 |
| novel.7792 | 1.239109 | 0.004722 | 0.037144 | Chr7 | 95574967 | 95578473 | 1913 |
| MsG0380012344.01 | -2.32265 | 0.004728 | 0.037181 | Chr3 | 15189230 | 15192696 | 3387 |
| MsG0380016349.01 | -1.98065 | 0.004733 | 0.037206 | Chr3 | 82296906 | 82300540 | 2038 |
| MsG0280009400.01 | -1.14119 | 0.004744 | 0.037289 | Chr2 | 51360136 | 51373956 | 1812 |
| MsG0680032607.01 | 1.946008 | 0.00475 | 0.037325 | Chr6 | 45877178 | 45877465 | 288 |
| novel.1439 | 3.478489 | 0.00476 | 0.037395 | Chr2 | 50400124 | 50401202 | 812 |
| MsG0280009528.01 | 2.839888 | 0.004762 | 0.037396 | Chr2 | 53750519 | 53754237 | 1636 |
| MsG0880042550.01 | 5.095496 | 0.00477 | 0.037451 | Chr8 | 10401944 | 10405594 | 307 |
| MsG0880045916.01 | 2.376357 | 0.004776 | 0.037483 | Chr8 | 66410837 | 66414885 | 2990 |
| novel.7087 | -2.29851 | 0.004777 | 0.037483 | Chr7 | 66244189 | 66247384 | 1792 |
| MsG0080047924.01 | -1.04334 | 0.004795 | 0.037619 | contig147end | 18104 | 30483 | 2158 |
| MsG0280007748.01 | -2.26083 | 0.004804 | 0.037679 | Chr2 | 19975913 | 19977120 | 1023 |
| novel.2327 | -5.52354 | 0.00481 | 0.037715 | Chr3 | 39323020 | 39330321 | 598 |
| MsG0880042001.01 | -1.13483 | 0.004813 | 0.037717 | Chr8 | 2327278 | 2328848 | 996 |
| MsG0580027831.01 | -3.00179 | 0.004813 | 0.037717 | Chr5 | 69295575 | 69296441 | 867 |
| MsG0080049065.01 | -4.10086 | 0.004814 | 0.037718 | contig645end | 4142 | 4834 | 693 |
| MsG0680031143.01 | 6.110381 | 0.004816 | 0.037718 | Chr6 | 15060355 | 15067464 | 1410 |
| MsG0280009823.01 | -2.24619 | 0.004817 | 0.037718 | Chr2 | 58931715 | 58938986 | 4094 |
| MsG0180000474.01 | -2.75007 | 0.004818 | 0.03772 | Chr1 | 6565765 | 6566515 | 534 |
| novel.6021 | 5.399536 | 0.004824 | 0.037747 | Chr6 | 1E+08 | 1E+08 | 672 |
| MsG0680030980.01 | 4.805218 | 0.004824 | 0.037747 | Chr6 | 12437277 | 12438042 | 633 |
| MsG0480018663.01 | 4.84013 | 0.004829 | 0.03777 | Chr4 | 8272826 | 8274091 | 1266 |
| novel.5125 | 4.8762 | 0.00483 | 0.03777 | Chr5 | 44337742 | 44338975 | 604 |
| novel.8052 | -2.65206 | 0.004848 | 0.0379 | Chr8 | 50967476 | 50969711 | 1443 |
| novel.4023 | -1.02668 | 0.004851 | 0.037918 | Chr4 | 57511557 | 57518047 | 710 |
| novel.5062 | -1.49617 | 0.004853 | 0.037923 | Chr5 | 29850446 | 29852597 | 568 |
| novel.5118 | 5.654791 | 0.004861 | 0.037972 | Chr5 | 43354061 | 43355101 | 855 |
| MsG0680030451.01 | 2.382598 | 0.004863 | 0.037982 | Chr6 | 3133594 | 3137375 | 2162 |
| MsG0480022467.01 | -1.65925 | 0.004866 | 0.037996 | Chr4 | 72877630 | 72885723 | 2453 |
| MsG0380011543.01 | -1.67694 | 0.00487 | 0.038011 | Chr3 | 1211267 | 1212412 | 1146 |
| MsG0280010300.01 | 2.136342 | 0.004877 | 0.038056 | Chr2 | 67590551 | 67594305 | 1094 |
| novel.6184 | -3.79342 | 0.00488 | 0.038071 | Chr6 | 7676662 | 7677233 | 548 |
| MsG0480018701.01 | -3.53748 | 0.004882 | 0.038077 | Chr4 | 8798147 | 8799013 | 867 |
| MsG0880046408.01 | -1.54072 | 0.004884 | 0.038082 | Chr8 | 73071154 | 73075069 | 1777 |
| MsG0380017707.01 | -1.17361 | 0.004904 | 0.038224 | Chr3 | 99819986 | 99824416 | 2244 |
| MsG0780040895.01 | 1.714142 | 0.004905 | 0.038224 | Chr7 | 83960557 | 83963564 | 909 |
| MsG0180001127.01 | -5.44356 | 0.004907 | 0.038233 | Chr1 | 16339011 | 16340803 | 1401 |
| MsG0580026251.01 | 3.958239 | 0.004915 | 0.038281 | Chr5 | 33442345 | 33447708 | 2235 |
| MsG0180000629.01 | -2.15269 | 0.004916 | 0.038281 | Chr1 | 8856351 | 8861786 | 1820 |
| MsG0180004987.01 | 1.083379 | 0.00492 | 0.038297 | Chr1 | 85262197 | 85269818 | 693 |
| novel.4469 | 2.112346 | 0.004921 | 0.038297 | Chr5 | 28247405 | 28251545 | 1569 |
| MsG0080048878.01 | 1.160773 | 0.004922 | 0.038297 | contig505end | 10001 | 16131 | 2277 |
| MsG0180001548.01 | 1.454944 | 0.004927 | 0.038324 | Chr1 | 23105644 | 23109133 | 1602 |
| MsG0180004470.01 | -1.81129 | 0.004936 | 0.03838 | Chr1 | 78503448 | 78520007 | 3078 |
| MsG0480020923.01 | -4.91691 | 0.004936 | 0.03838 | Chr4 | 50623137 | 50623901 | 765 |
| MsG0180005474.01 | 1.217014 | 0.004939 | 0.038389 | Chr1 | 92237364 | 92237726 | 363 |
| MsG0180000520.01 | -2.34161 | 0.004946 | 0.038432 | Chr1 | 7345158 | 7346397 | 471 |
| MsG0780039391.01 | -1.53651 | 0.004952 | 0.03847 | Chr7 | 63319880 | 63321148 | 1269 |
| MsG0180000452.01 | -1.4655 | 0.00497 | 0.038587 | Chr1 | 6247819 | 6249787 | 934 |
| novel.3747 | -1.95358 | 0.00497 | 0.038587 | Chr4 | 336355 | 337958 | 1112 |
| novel.8322 | 1.206406 | 0.004971 | 0.038587 | Chr8 | 3843740 | 3849314 | 3644 |
| MsG0480019575.01 | 1.471045 | 0.004972 | 0.038587 | Chr4 | 22436797 | 22439813 | 384 |
| novel.919 | 1.343834 | 0.004976 | 0.038605 | Chr1 | 63429197 | 63432363 | 585 |
| MsG0480019447.01 | -1.65998 | 0.004983 | 0.038652 | Chr4 | 20524772 | 20546450 | 4758 |
| MsG0580025625.01 | 1.178933 | 0.004989 | 0.038681 | Chr5 | 21423727 | 21426802 | 973 |
| novel.7295 | 2.637953 | 0.004989 | 0.038681 | Chr7 | 95351475 | 95353578 | 1164 |
| MsG0480022397.01 | -1.70685 | 0.004995 | 0.038714 | Chr4 | 72034045 | 72035008 | 369 |
| MsG0880042358.01 | -5.53276 | 0.004998 | 0.03873 | Chr8 | 7547894 | 7549756 | 618 |
| novel.334 | 1.62243 | 0.005002 | 0.038745 | Chr1 | 61655109 | 61661028 | 2629 |
| novel.6318 | 1.581069 | 0.005007 | 0.038778 | Chr6 | 24641490 | 24644240 | 2211 |
| MsG0880046024.01 | -1.84542 | 0.005016 | 0.038838 | Chr8 | 67822777 | 67824755 | 720 |
| MsG0180005807.01 | 1.238474 | 0.005018 | 0.038838 | Chr1 | 96389251 | 96389512 | 262 |
| MsG0780038123.01 | -1.92902 | 0.005051 | 0.039087 | Chr7 | 40636643 | 40638058 | 1416 |
| MsG0780041101.01 | 1.260829 | 0.005054 | 0.039099 | Chr7 | 86605146 | 86606198 | 612 |
| MsG0580024805.01 | 1.116812 | 0.005055 | 0.039099 | Chr5 | 9971617 | 9975362 | 2161 |
| MsG0780040536.01 | -1.21806 | 0.005059 | 0.039118 | Chr7 | 78916766 | 78919377 | 1050 |
| MsG0380015835.01 | -5.40848 | 0.005061 | 0.039121 | Chr3 | 75739448 | 75740026 | 579 |
| MsG0180001121.01 | -1.2796 | 0.005072 | 0.039198 | Chr1 | 16278868 | 16279920 | 1053 |
| MsG0380015008.01 | -2.7079 | 0.005076 | 0.039223 | Chr3 | 63878474 | 63880238 | 971 |
| MsG0180004445.01 | -1.41082 | 0.005081 | 0.039245 | Chr1 | 78126031 | 78126591 | 561 |
| novel.6098 | -2.86735 | 0.005082 | 0.039247 | Chr6 | 1.08E+08 | 1.08E+08 | 562 |
| MsG0880047665.01 | -2.09928 | 0.005084 | 0.039247 | Chr8 | 89398819 | 89401620 | 1906 |
| MsG0780038545.01 | 4.249251 | 0.005095 | 0.039326 | Chr7 | 48484483 | 48509428 | 8106 |
| MsG0880046349.01 | 1.28492 | 0.005096 | 0.039326 | Chr8 | 72288545 | 72289003 | 459 |
| MsG0780040294.01 | -2.79053 | 0.005106 | 0.039381 | Chr7 | 75574306 | 75576675 | 943 |
| novel.2141 | -1.43942 | 0.005106 | 0.039381 | Chr3 | 3814705 | 3835629 | 1635 |
| MsG0780036587.01 | -1.65815 | 0.005109 | 0.039381 | Chr7 | 10149369 | 10150805 | 1437 |
| MsG0480018303.01 | 5.565285 | 0.00511 | 0.039381 | Chr4 | 3131876 | 3135380 | 999 |
| novel.6421 | 4.95006 | 0.005112 | 0.039386 | Chr6 | 44762031 | 44763210 | 1180 |
| novel.8811 | 1.455659 | 0.005116 | 0.039404 | contig142end | 90658 | 95373 | 1199 |
| MsG0080048765.01 | -1.33683 | 0.005131 | 0.039511 | contig428end | 28067 | 45559 | 7242 |
| novel.1673 | -1.36349 | 0.005139 | 0.039559 | Chr2 | 3387228 | 3390916 | 939 |
| novel.6572 | -5.68953 | 0.005141 | 0.039559 | Chr6 | 75603751 | 75611272 | 1315 |
| MsG0880043918.01 | 2.900199 | 0.005141 | 0.039559 | Chr8 | 32560411 | 32561916 | 1506 |
| MsG0380011491.01 | -1.1802 | 0.005144 | 0.039571 | Chr3 | 298690 | 301629 | 1199 |
| MsG0780036984.01 | 5.479345 | 0.005149 | 0.039597 | Chr7 | 17512886 | 17513299 | 414 |
| MsG0380016078.01 | 1.26368 | 0.00516 | 0.039674 | Chr3 | 78817955 | 78818786 | 585 |
| MsG0880046059.01 | 1.884821 | 0.005166 | 0.039696 | Chr8 | 68408238 | 68411525 | 771 |
| MsG0180000553.01 | -1.18637 | 0.005172 | 0.039734 | Chr1 | 7713565 | 7717914 | 1275 |
| MsG0780038329.01 | -2.01982 | 0.005177 | 0.039764 | Chr7 | 44368544 | 44381027 | 1670 |
| MsG0180003534.01 | -1.06376 | 0.00519 | 0.039849 | Chr1 | 63852877 | 63856352 | 1963 |
| novel.3831 | -3.72083 | 0.005198 | 0.039905 | Chr4 | 16815503 | 16821374 | 911 |
| MsG0380015610.01 | -2.13166 | 0.005211 | 0.039994 | Chr3 | 72485404 | 72485775 | 372 |
| MsG0580025118.01 | 5.953917 | 0.005213 | 0.04 | Chr5 | 14043896 | 14047136 | 492 |
| MsG0480021356.01 | 1.442498 | 0.005223 | 0.040063 | Chr4 | 57023813 | 57028997 | 1229 |
| MsG0380012428.01 | -1.4358 | 0.005227 | 0.040082 | Chr3 | 16390068 | 16407481 | 3823 |
| MsG0680030560.01 | -1.02875 | 0.00523 | 0.040095 | Chr6 | 5290020 | 5292662 | 1796 |
| novel.7861 | 5.312981 | 0.005233 | 0.040108 | Chr8 | 11604429 | 11606488 | 791 |
| MsG0280011068.01 | 1.773754 | 0.005241 | 0.040157 | Chr2 | 79073683 | 79079144 | 1527 |
| MsG0780037220.01 | 5.349114 | 0.005252 | 0.040223 | Chr7 | 22020556 | 22023597 | 621 |
| MsG0880046434.01 | 5.349114 | 0.005252 | 0.040223 | Chr8 | 73339292 | 73339687 | 396 |
| novel.8840 | 4.658601 | 0.005258 | 0.04026 | contig175end | 11938 | 14860 | 1701 |
| novel.3837 | -4.80532 | 0.005278 | 0.040378 | Chr4 | 17682562 | 17684465 | 1313 |
| novel.9052 | -1.62021 | 0.005278 | 0.040378 | contig459end | 9249 | 18423 | 3304 |
| novel.1990 | -5.6321 | 0.005279 | 0.040378 | Chr2 | 67995547 | 67996536 | 950 |
| MsG0180000047.01 | -1.77594 | 0.005295 | 0.040489 | Chr1 | 794510 | 799633 | 2739 |
| MsG0380014449.01 | -2.1022 | 0.005297 | 0.040497 | Chr3 | 55002297 | 55004102 | 1574 |
| novel.816 | -6.33944 | 0.005302 | 0.040518 | Chr1 | 35154238 | 35156701 | 567 |
| MsG0580029763.01 | -1.2846 | 0.00531 | 0.040566 | Chr5 | 1.02E+08 | 1.02E+08 | 1966 |
| MsG0580028016.01 | 1.148896 | 0.005337 | 0.040757 | Chr5 | 73196013 | 73198522 | 1116 |
| novel.8844 | 1.426229 | 0.005344 | 0.040801 | contig178end | 20714 | 24166 | 1388 |
| MsG0580024083.01 | -3.39031 | 0.005355 | 0.040876 | Chr5 | 605309 | 609198 | 1920 |
| MsG0680031139.01 | 3.559568 | 0.005361 | 0.040913 | Chr6 | 15030772 | 15032796 | 2025 |
| MsG0380012512.01 | 2.307512 | 0.005364 | 0.040924 | Chr3 | 18877059 | 18877250 | 192 |
| MsG0180004947.01 | -2.43065 | 0.005372 | 0.040971 | Chr1 | 84769609 | 84769842 | 234 |
| MsG0680030851.01 | -2.79596 | 0.005379 | 0.041014 | Chr6 | 10487531 | 10488769 | 1239 |
| novel.5562 | 5.445721 | 0.005383 | 0.041034 | Chr6 | 7275062 | 7275601 | 423 |
| MsG0580028284.01 | 1.984647 | 0.005389 | 0.041055 | Chr5 | 77825269 | 77825651 | 276 |
| MsG0780039438.01 | -1.8003 | 0.005389 | 0.041055 | Chr7 | 64088491 | 64092265 | 1588 |
| MsG0580025310.01 | 1.89271 | 0.00539 | 0.041055 | Chr5 | 16818146 | 16821401 | 2511 |
| MsG0280010562.01 | -1.31963 | 0.005391 | 0.041055 | Chr2 | 71522999 | 71525324 | 1188 |
| novel.436 | 1.628124 | 0.005396 | 0.04108 | Chr1 | 80282480 | 80290501 | 844 |
| MsG0280008407.01 | 1.401452 | 0.005408 | 0.041159 | Chr2 | 30899066 | 30902727 | 2841 |
| MsG0780038953.01 | -1.3461 | 0.00541 | 0.041164 | Chr7 | 55716579 | 55717082 | 504 |
| novel.551 | -4.10015 | 0.005412 | 0.041166 | Chr1 | 97344952 | 97348200 | 910 |
| MsG0480023546.01 | -1.7498 | 0.005414 | 0.041166 | Chr4 | 86725861 | 86729091 | 906 |
| novel.3715 | -5.55252 | 0.005415 | 0.041166 | Chr4 | 88176855 | 88177890 | 607 |
| novel.6635 | 5.822384 | 0.005415 | 0.041166 | Chr6 | 89778126 | 89780012 | 510 |
| MsG0580029371.01 | -1.60071 | 0.005425 | 0.041219 | Chr5 | 96048918 | 96053207 | 1115 |
| MsG0280008221.01 | -4.36776 | 0.005427 | 0.041219 | Chr2 | 27360111 | 27361289 | 1179 |
| novel.1148 | -4.84531 | 0.005427 | 0.041219 | Chr1 | 1.01E+08 | 1.01E+08 | 615 |
| novel.5800 | 5.562102 | 0.005428 | 0.041219 | Chr6 | 55216924 | 55218745 | 573 |
| novel.7864 | 6.148186 | 0.005431 | 0.041235 | Chr8 | 11677323 | 11678314 | 660 |
| MsG0480023963.01 | -3.69615 | 0.005441 | 0.041296 | Chr4 | 91746749 | 91748643 | 870 |
| MsG0880042002.01 | -1.64323 | 0.005443 | 0.041296 | Chr8 | 2330100 | 2334085 | 831 |
| MsG0780041504.01 | -2.17991 | 0.005444 | 0.041296 | Chr7 | 91790432 | 91790827 | 396 |
| MsG0880044904.01 | 4.183608 | 0.005467 | 0.041464 | Chr8 | 51200529 | 51201043 | 273 |
| MsG0580025348.01 | 1.116117 | 0.005483 | 0.041568 | Chr5 | 17271067 | 17275670 | 2160 |
| MsG0780038482.01 | -1.01616 | 0.005484 | 0.041568 | Chr7 | 47399433 | 47413096 | 2436 |
| MsG0880041934.01 | 6.092263 | 0.005491 | 0.041608 | Chr8 | 1431337 | 1434768 | 1776 |
| MsG0880042965.01 | -1.59711 | 0.005492 | 0.041608 | Chr8 | 16791470 | 16795234 | 3021 |
| MsG0180002525.01 | -2.01618 | 0.005498 | 0.041642 | Chr1 | 39805202 | 39811124 | 1674 |
| MsG0580028637.01 | 1.54287 | 0.005505 | 0.041689 | Chr5 | 83949398 | 83951523 | 1210 |
| novel.3718 | 1.322349 | 0.005508 | 0.041697 | Chr4 | 88336103 | 88338829 | 848 |
| MsG0480021006.01 | 6.045723 | 0.00551 | 0.041697 | Chr4 | 51986332 | 51990301 | 586 |
| MsG0580027880.01 | -1.26626 | 0.005511 | 0.041697 | Chr5 | 70243353 | 70248196 | 2461 |
| novel.1163 | -1.2524 | 0.005522 | 0.041777 | Chr2 | 1187248 | 1189882 | 2635 |
| novel.7336 | 1.552318 | 0.005529 | 0.041812 | Chr7 | 10674604 | 10675820 | 1217 |
| novel.7881 | -3.28577 | 0.005532 | 0.041828 | Chr8 | 16610313 | 16614468 | 988 |
| novel.9239 | -2.30761 | 0.005536 | 0.041849 | contig564end | 12321 | 21558 | 2636 |
| novel.9178 | -2.6808 | 0.00554 | 0.041868 | contig531end | 2474 | 4137 | 1023 |
| MsG0380017940.01 | -2.12248 | 0.005543 | 0.041876 | Chr3 | 1.02E+08 | 1.02E+08 | 1080 |
| MsG0280008388.01 | -1.99702 | 0.005546 | 0.04189 | Chr2 | 30547511 | 30548143 | 633 |
| novel.6460 | -6.08164 | 0.005551 | 0.041916 | Chr6 | 51931579 | 51936368 | 506 |
| MsG0580027613.01 | -1.4087 | 0.005553 | 0.041925 | Chr5 | 64324616 | 64337404 | 2570 |
| MsG0280006600.01 | -1.37752 | 0.005564 | 0.041998 | Chr2 | 3867835 | 3882638 | 3426 |
| MsG0880047748.01 | -1.5849 | 0.005567 | 0.042003 | Chr8 | 90354526 | 90355461 | 432 |
| MsG0780037802.01 | 6.294428 | 0.005569 | 0.042008 | Chr7 | 33439223 | 33441598 | 603 |
| novel.6376 | 5.302248 | 0.005588 | 0.042143 | Chr6 | 35184538 | 35191138 | 349 |
| MsG0380016720.01 | -3.06339 | 0.005602 | 0.042233 | Chr3 | 87041278 | 87043192 | 1233 |
| MsG0380015970.01 | 1.281301 | 0.005604 | 0.042233 | Chr3 | 77352022 | 77353884 | 550 |
| MsG0480021511.01 | 1.370159 | 0.005604 | 0.042233 | Chr4 | 58877298 | 58878527 | 735 |
| novel.5649 | -1.60847 | 0.005608 | 0.042242 | Chr6 | 22928657 | 22932651 | 550 |
| MsG0580027191.01 | -1.36943 | 0.005611 | 0.042242 | Chr5 | 55893038 | 55895689 | 1767 |
| novel.5789 | 1.09298 | 0.005611 | 0.042242 | Chr6 | 52749603 | 52754946 | 4221 |
| MsG0480021767.01 | 1.434726 | 0.005611 | 0.042242 | Chr4 | 64045808 | 64050896 | 512 |
| MsG0880044727.01 | -2.80779 | 0.005619 | 0.042294 | Chr8 | 48370465 | 48371934 | 1470 |
| novel.6582 | 5.614995 | 0.005622 | 0.042297 | Chr6 | 79549581 | 79550366 | 694 |
| MsG0680032107.01 | -2.60562 | 0.005623 | 0.042297 | Chr6 | 33844826 | 33871229 | 4443 |
| MsG0880047443.01 | -2.44818 | 0.005634 | 0.042373 | Chr8 | 86365143 | 86365691 | 549 |
| MsG0180006226.01 | 1.569225 | 0.005643 | 0.042428 | Chr1 | 1.02E+08 | 1.02E+08 | 520 |
| novel.1961 | -5.45335 | 0.005644 | 0.042428 | Chr2 | 62405805 | 62407141 | 708 |
| MsG0880045958.01 | -1.85782 | 0.005647 | 0.042441 | Chr8 | 66942863 | 66944593 | 1039 |
| MsG0480018351.01 | 2.185457 | 0.005652 | 0.042465 | Chr4 | 3678094 | 3680316 | 342 |
| MsG0880043955.01 | -1.77262 | 0.005659 | 0.042503 | Chr8 | 33374107 | 33379933 | 636 |
| MsG0280008643.01 | 1.236291 | 0.00566 | 0.042506 | Chr2 | 35623236 | 35629745 | 2657 |
| MsG0180000412.01 | 1.106357 | 0.005674 | 0.042586 | Chr1 | 5664397 | 5667373 | 1113 |
| novel.3422 | 1.889577 | 0.005674 | 0.042586 | Chr4 | 38923595 | 38925830 | 2144 |
| MsG0480019034.01 | -1.3172 | 0.005677 | 0.042587 | Chr4 | 13607672 | 13613606 | 1800 |
| MsG0280007137.01 | 1.390116 | 0.005678 | 0.042587 | Chr2 | 11099120 | 11104429 | 2101 |
| novel.412 | 1.232554 | 0.005678 | 0.042587 | Chr1 | 75830760 | 75834706 | 1766 |
| MsG0380015197.01 | 2.210471 | 0.005697 | 0.04269 | Chr3 | 66678400 | 66679565 | 387 |
| novel.7927 | -4.26213 | 0.005697 | 0.04269 | Chr8 | 23374103 | 23374806 | 417 |
| MsG0180004939.01 | 3.147849 | 0.005699 | 0.04269 | Chr1 | 84704453 | 84711770 | 1446 |
| MsG0480018797.01 | -5.30761 | 0.0057 | 0.04269 | Chr4 | 10058462 | 10058962 | 501 |
| novel.7330 | 1.168143 | 0.0057 | 0.04269 | Chr7 | 9591523 | 9595275 | 744 |
| MsG0680031993.01 | 1.945528 | 0.005701 | 0.04269 | Chr6 | 31530459 | 31540877 | 4071 |
| MsG0480018594.01 | -2.75896 | 0.005712 | 0.042763 | Chr4 | 7116075 | 7123564 | 1965 |
| novel.3007 | -1.80239 | 0.005735 | 0.042916 | Chr3 | 72573863 | 72584365 | 2789 |
| MsG0180001111.01 | 1.070479 | 0.005746 | 0.042984 | Chr1 | 16116128 | 16119198 | 1326 |
| MsG0480018749.01 | -5.01312 | 0.005755 | 0.043039 | Chr4 | 9375193 | 9377872 | 2625 |
| MsG0880046325.01 | -1.66194 | 0.005758 | 0.043055 | Chr8 | 72035498 | 72038219 | 1044 |
| MsG0480020025.01 | 1.353654 | 0.005763 | 0.043069 | Chr4 | 32190111 | 32190584 | 474 |
| novel.2361 | 5.296881 | 0.005764 | 0.043069 | Chr3 | 48193221 | 48193975 | 268 |
| novel.3615 | 5.296881 | 0.005764 | 0.043069 | Chr4 | 75509750 | 75510072 | 323 |
| novel.4114 | 6.048106 | 0.005766 | 0.043073 | Chr4 | 70274837 | 70275692 | 715 |
| MsG0780036983.01 | -5.80812 | 0.005771 | 0.043097 | Chr7 | 17508022 | 17508435 | 414 |
| MsG0580024924.01 | 1.462494 | 0.005784 | 0.043181 | Chr5 | 11702261 | 11709025 | 1698 |
| novel.6060 | 4.183023 | 0.005787 | 0.043194 | Chr6 | 1.05E+08 | 1.05E+08 | 1030 |
| novel.68 | 5.442968 | 0.005802 | 0.043295 | Chr1 | 8694464 | 8694870 | 407 |
| novel.203 | -5.32118 | 0.005822 | 0.04342 | Chr1 | 28626996 | 28629380 | 359 |
| MsG0380012658.01 | 1.965666 | 0.005823 | 0.04342 | Chr3 | 22016395 | 22025252 | 2409 |
| MsG0280011253.01 | -1.30591 | 0.005823 | 0.04342 | Chr2 | 81477536 | 81479595 | 1578 |
| novel.977 | 6.154559 | 0.005825 | 0.04342 | Chr1 | 77690364 | 77691817 | 485 |
| MsG0380012187.01 | -1.60752 | 0.005827 | 0.043426 | Chr3 | 12386998 | 12390770 | 838 |
| MsG0680030624.01 | 6.178908 | 0.005834 | 0.043465 | Chr6 | 6314090 | 6319955 | 3122 |
| MsG0880044798.01 | -1.59384 | 0.005842 | 0.043521 | Chr8 | 49353510 | 49357994 | 1731 |
| MsG0780041660.01 | 3.648551 | 0.005851 | 0.04357 | Chr7 | 93629418 | 93634487 | 1408 |
| novel.6778 | 4.267411 | 0.005852 | 0.043574 | Chr6 | 1.12E+08 | 1.12E+08 | 1244 |
| novel.8606 | -1.13707 | 0.005863 | 0.043618 | Chr8 | 61024087 | 61029836 | 1552 |
| MsG0180002918.01 | -1.67632 | 0.005863 | 0.043618 | Chr1 | 52956123 | 52957496 | 1374 |
| MsG0780036965.01 | 1.642878 | 0.00588 | 0.043734 | Chr7 | 17235444 | 17236609 | 627 |
| MsG0780039969.01 | -3.51727 | 0.005888 | 0.04378 | Chr7 | 71223307 | 71231828 | 2169 |
| novel.4700 | -2.66434 | 0.00589 | 0.043788 | Chr5 | 77703007 | 77707798 | 2022 |
| MsG0380013369.01 | -2.8541 | 0.005894 | 0.043807 | Chr3 | 35967146 | 35976511 | 1704 |
| MsG0580024820.01 | 1.246915 | 0.005901 | 0.043849 | Chr5 | 10277874 | 10279962 | 1463 |
| MsG0780040258.01 | -2.60763 | 0.005906 | 0.043874 | Chr7 | 75027592 | 75027879 | 288 |
| novel.128 | 1.189673 | 0.005914 | 0.04391 | Chr1 | 16882776 | 16885944 | 1490 |
| MsG0680034492.01 | -1.57064 | 0.005914 | 0.04391 | Chr6 | 88492563 | 88499667 | 1080 |
| MsG0180000188.01 | -1.10716 | 0.005918 | 0.04393 | Chr1 | 2615262 | 2622228 | 2544 |
| MsG0180003071.01 | 2.106515 | 0.005925 | 0.043972 | Chr1 | 56167424 | 56174032 | 1521 |
| MsG0880042969.01 | -1.32603 | 0.005931 | 0.043991 | Chr8 | 16823631 | 16823990 | 360 |
| MsG0080049135.01 | 1.551517 | 0.005932 | 0.043991 | contig91end | 3012 | 3607 | 489 |
| MsG0280007873.01 | 1.114254 | 0.005933 | 0.043991 | Chr2 | 22140865 | 22156054 | 3594 |
| MsG0380011706.01 | 1.186994 | 0.005934 | 0.043991 | Chr3 | 3872989 | 3886261 | 1075 |
| novel.2145 | 2.69099 | 0.005938 | 0.044009 | Chr3 | 4515795 | 4524828 | 2028 |
| MsG0880046036.01 | 2.149504 | 0.005943 | 0.044039 | Chr8 | 68037582 | 68052104 | 2989 |
| MsG0180003650.01 | -4.78549 | 0.005952 | 0.044095 | Chr1 | 65778569 | 65780068 | 1188 |
| MsG0380016628.01 | -1.2511 | 0.005957 | 0.044119 | Chr3 | 85870591 | 85870935 | 345 |
| novel.9100 | -1.55566 | 0.005959 | 0.044121 | contig483end | 9863 | 15225 | 2572 |
| MsG0280007131.01 | 2.231515 | 0.005963 | 0.044143 | Chr2 | 11026743 | 11028141 | 1125 |
| MsG0280010827.01 | -4.36441 | 0.005974 | 0.044199 | Chr2 | 75528421 | 75528831 | 411 |
| MsG0280009386.01 | -1.41436 | 0.005974 | 0.044199 | Chr2 | 51207024 | 51210424 | 720 |
| MsG0880047055.01 | 1.454263 | 0.005975 | 0.044199 | Chr8 | 81404771 | 81413336 | 1101 |
| MsG0380011663.01 | -1.68144 | 0.005981 | 0.04423 | Chr3 | 3152754 | 3158119 | 2850 |
| MsG0180002992.01 | -1.07019 | 0.005982 | 0.04423 | Chr1 | 54514104 | 54516181 | 1160 |
| MsG0880044373.01 | -5.90797 | 0.005992 | 0.044276 | Chr8 | 40966194 | 40975918 | 2481 |
| novel.3434 | -5.60177 | 0.005998 | 0.044315 | Chr4 | 41874383 | 41875069 | 591 |
| novel.162 | -5.31491 | 0.006013 | 0.04441 | Chr1 | 22992036 | 22997126 | 1051 |
| novel.7313 | -1.004 | 0.006016 | 0.044427 | Chr7 | 4189140 | 4190350 | 498 |
| MsG0380016729.01 | 1.187631 | 0.006022 | 0.044448 | Chr3 | 87137682 | 87142318 | 2329 |
| novel.1173 | -1.14628 | 0.006033 | 0.044512 | Chr2 | 2274090 | 2277678 | 1761 |
| MsG0780040736.01 | -2.68104 | 0.006035 | 0.044518 | Chr7 | 81794682 | 81796295 | 1614 |
| MsG0680030858.01 | 1.127763 | 0.006037 | 0.04452 | Chr6 | 10574417 | 10579512 | 1505 |
| MsG0480019122.01 | -5.46404 | 0.006039 | 0.044526 | Chr4 | 14804238 | 14805590 | 1353 |
| novel.4934 | 1.326197 | 0.00604 | 0.044526 | Chr5 | 5302574 | 5305930 | 1001 |
| MsG0780039847.01 | -1.69984 | 0.006047 | 0.044564 | Chr7 | 69695001 | 69701395 | 1396 |
| novel.7606 | 1.617507 | 0.006051 | 0.044583 | Chr7 | 69512145 | 69516437 | 1298 |
| novel.6780 | 1.756743 | 0.006055 | 0.044597 | Chr6 | 1.12E+08 | 1.12E+08 | 992 |
| MsG0480021100.01 | 2.945978 | 0.006061 | 0.044625 | Chr4 | 53462389 | 53463715 | 672 |
| MsG0380017238.01 | 1.281825 | 0.006061 | 0.044625 | Chr3 | 93865781 | 93867441 | 1477 |
| MsG0780036116.01 | -2.08067 | 0.00607 | 0.044667 | Chr7 | 2869907 | 2872561 | 2460 |
| MsG0680031460.01 | 1.209347 | 0.00607 | 0.044667 | Chr6 | 20801044 | 20805263 | 1177 |
| MsG0480023484.01 | -1.40442 | 0.006073 | 0.044676 | Chr4 | 85830916 | 85841260 | 3714 |
| MsG0280010218.01 | -1.7254 | 0.006081 | 0.044722 | Chr2 | 66323311 | 66325655 | 2148 |
| novel.3308 | 6.226905 | 0.00609 | 0.044782 | Chr4 | 15193734 | 15195044 | 525 |
| MsG0580029374.01 | 1.171474 | 0.006096 | 0.044811 | Chr5 | 96066959 | 96072195 | 811 |
| novel.3067 | -2.10822 | 0.006103 | 0.044851 | Chr3 | 80937030 | 80938629 | 1600 |
| MsG0580025869.01 | -2.16027 | 0.006105 | 0.044855 | Chr5 | 25544149 | 25545126 | 978 |
| novel.2175 | -5.63208 | 0.006106 | 0.044855 | Chr3 | 8920531 | 8957281 | 310 |
| novel.7561 | -4.15895 | 0.006109 | 0.044868 | Chr7 | 63745638 | 63745967 | 235 |
| MsG0880042609.01 | -1.6033 | 0.006119 | 0.044926 | Chr8 | 11429474 | 11437111 | 5345 |
| MsG0480020557.01 | 1.771885 | 0.006123 | 0.044932 | Chr4 | 43297133 | 43298649 | 858 |
| MsG0880042290.01 | 1.247561 | 0.006124 | 0.044932 | Chr8 | 6578571 | 6583979 | 2804 |
| MsG0680033879.01 | 2.518622 | 0.006124 | 0.044932 | Chr6 | 75503695 | 75504399 | 705 |
| MsG0580026719.01 | 2.112763 | 0.00613 | 0.044962 | Chr5 | 42806117 | 42807571 | 1455 |
| novel.8273 | -1.87107 | 0.006143 | 0.045051 | Chr8 | 88573052 | 88575269 | 2218 |
| novel.2265 | 4.57304 | 0.006145 | 0.045054 | Chr3 | 22784428 | 22789318 | 2370 |
| MsG0680033412.01 | -1.41635 | 0.006152 | 0.045088 | Chr6 | 64261946 | 64264752 | 1209 |
| MsG0780040553.01 | -3.47616 | 0.006153 | 0.045088 | Chr7 | 79134153 | 79135986 | 909 |
| novel.1325 | 6.061785 | 0.006169 | 0.045179 | Chr2 | 27029299 | 27032109 | 588 |
| novel.6765 | -1.94077 | 0.006169 | 0.045179 | Chr6 | 1.1E+08 | 1.1E+08 | 1294 |
| MsG0580028707.01 | -1.94618 | 0.006176 | 0.045225 | Chr5 | 85127293 | 85133208 | 3054 |
| novel.6498 | 1.52051 | 0.006178 | 0.045227 | Chr6 | 61083071 | 61086968 | 1750 |
| novel.1830 | -1.2673 | 0.00618 | 0.04523 | Chr2 | 28757833 | 29036516 | 2885 |
| MsG0380013761.01 | 5.577051 | 0.006183 | 0.045241 | Chr3 | 45316642 | 45318889 | 1881 |
| MsG0180003742.01 | 4.833029 | 0.006185 | 0.045247 | Chr1 | 67540397 | 67542942 | 1581 |
| novel.4101 | -2.78839 | 0.006207 | 0.045387 | Chr4 | 69109588 | 69111229 | 636 |
| MsG0480022105.01 | -1.84923 | 0.006208 | 0.045387 | Chr4 | 68295955 | 68299255 | 2832 |
| novel.2452 | -1.50406 | 0.006213 | 0.045415 | Chr3 | 69791430 | 69792805 | 1376 |
| novel.636 | 1.736065 | 0.006217 | 0.045431 | Chr1 | 4946574 | 4948905 | 1806 |
| MsG0180002424.01 | 1.968445 | 0.006221 | 0.045449 | Chr1 | 38274034 | 38276847 | 1277 |
| MsG0780040357.01 | -1.56463 | 0.006222 | 0.045449 | Chr7 | 76384959 | 76388219 | 2094 |
| MsG0680030599.01 | 1.259852 | 0.006227 | 0.045473 | Chr6 | 5862287 | 5878817 | 2781 |
| MsG0880043158.01 | -1.88087 | 0.006242 | 0.04555 | Chr8 | 19892766 | 19896377 | 935 |
| MsG0180005256.01 | 1.532215 | 0.006242 | 0.04555 | Chr1 | 89236144 | 89236644 | 501 |
| MsG0280007800.01 | 1.491458 | 0.006246 | 0.045563 | Chr2 | 20734940 | 20738256 | 927 |
| MsG0380013379.01 | -2.03856 | 0.006248 | 0.045569 | Chr3 | 36427065 | 36432436 | 1337 |
| novel.4138 | -4.69061 | 0.006255 | 0.04561 | Chr4 | 75118702 | 75119599 | 898 |
| novel.6115 | 1.311184 | 0.006271 | 0.045715 | Chr6 | 1.11E+08 | 1.11E+08 | 1041 |
| MsG0280007445.01 | 1.07218 | 0.0063 | 0.045914 | Chr2 | 15571898 | 15576183 | 794 |
| MsG0180004331.01 | 1.240081 | 0.006307 | 0.045956 | Chr1 | 76476829 | 76485100 | 3276 |
| MsG0880045439.01 | -1.20226 | 0.006326 | 0.046078 | Chr8 | 59154227 | 59168092 | 3332 |
| MsG0580024272.01 | 5.431155 | 0.006327 | 0.046078 | Chr5 | 2806775 | 2809683 | 1957 |
| MsG0480023067.01 | -1.1669 | 0.00633 | 0.046089 | Chr4 | 80376188 | 80376670 | 483 |
| MsG0380018000.01 | -1.77954 | 0.006338 | 0.046135 | Chr3 | 1.03E+08 | 1.03E+08 | 1372 |
| MsG0680030663.01 | 1.403718 | 0.006353 | 0.046227 | Chr6 | 6850196 | 6856993 | 1065 |
| MsG0180004497.01 | 1.659235 | 0.006354 | 0.046227 | Chr1 | 78904057 | 78904948 | 807 |
| novel.4386 | 4.749979 | 0.006357 | 0.046241 | Chr5 | 15931095 | 15931722 | 481 |
| novel.131 | -1.68034 | 0.006362 | 0.046265 | Chr1 | 17165355 | 17168215 | 2861 |
| MsG0380014550.01 | 2.138398 | 0.006374 | 0.04634 | Chr3 | 56406451 | 56415621 | 1380 |
| MsG0380014289.01 | 5.275968 | 0.006386 | 0.046419 | Chr3 | 52505007 | 52509031 | 756 |
| novel.1240 | -5.39671 | 0.00639 | 0.046437 | Chr2 | 12944883 | 12945431 | 452 |
| MsG0080047841.01 | 5.551473 | 0.006395 | 0.046455 | contig126end | 77560 | 81102 | 1752 |
| MsG0880047662.01 | -2.64918 | 0.006413 | 0.046581 | Chr8 | 89353989 | 89355180 | 999 |
| novel.15 | 3.683287 | 0.006419 | 0.046608 | Chr1 | 1903445 | 1905177 | 671 |
| MsG0180001063.01 | 1.015737 | 0.006423 | 0.046628 | Chr1 | 15415621 | 15420238 | 934 |
| MsG0180003146.01 | -1.55751 | 0.006426 | 0.046636 | Chr1 | 57371721 | 57382136 | 2756 |
| MsG0280007597.01 | -1.89783 | 0.006445 | 0.046764 | Chr2 | 17867345 | 17870619 | 819 |
| novel.3618 | 4.053453 | 0.00645 | 0.046788 | Chr4 | 76531879 | 76533173 | 674 |
| MsG0680032883.01 | 5.350063 | 0.00647 | 0.046926 | Chr6 | 51895445 | 51895750 | 306 |
| MsG0180005333.01 | 4.828911 | 0.006475 | 0.046949 | Chr1 | 90451611 | 90454361 | 1554 |
| novel.4905 | -4.33222 | 0.006477 | 0.046951 | Chr5 | 1.09E+08 | 1.09E+08 | 1035 |
| MsG0380017232.01 | -2.2181 | 0.006479 | 0.046955 | Chr3 | 93835653 | 93836463 | 334 |
| MsG0480021793.01 | -1.72721 | 0.006481 | 0.046955 | Chr4 | 64341066 | 64342025 | 960 |
| novel.9129 | 1.47715 | 0.006521 | 0.047236 | contig507end | 9952 | 12759 | 2052 |
| MsG0880042069.01 | -3.48493 | 0.006523 | 0.047236 | Chr8 | 3339161 | 3341537 | 516 |
| MsG0680035753.01 | 1.820866 | 0.006527 | 0.047256 | Chr6 | 1.12E+08 | 1.12E+08 | 510 |
| MsG0180002842.01 | 2.931307 | 0.006537 | 0.047306 | Chr1 | 47001010 | 47030701 | 3795 |
| novel.7503 | -5.28653 | 0.006551 | 0.047389 | Chr7 | 47885149 | 47886665 | 401 |
| MsG0180000292.01 | -1.20679 | 0.006552 | 0.047389 | Chr1 | 4044164 | 4046779 | 1242 |
| novel.7778 | -4.13394 | 0.00656 | 0.047435 | Chr7 | 94840956 | 94842507 | 1396 |
| MsG0480022123.01 | 4.642382 | 0.006563 | 0.047435 | Chr4 | 68559384 | 68560450 | 861 |
| MsG0780039507.01 | -1.34533 | 0.006563 | 0.047435 | Chr7 | 65026202 | 65027820 | 1395 |
| MsG0580024360.01 | -1.4393 | 0.00657 | 0.047476 | Chr5 | 3866087 | 3868576 | 1336 |
| MsG0180004792.01 | 1.059606 | 0.006583 | 0.047546 | Chr1 | 82783672 | 82784067 | 396 |
| novel.5455 | -2.78404 | 0.006583 | 0.047546 | Chr5 | 1.03E+08 | 1.03E+08 | 1249 |
| MsG0780040611.01 | -1.90402 | 0.006586 | 0.047554 | Chr7 | 79975706 | 79976176 | 471 |
| MsG0780036812.01 | -1.3614 | 0.006597 | 0.047627 | Chr7 | 14322161 | 14324958 | 2008 |
| MsG0280006915.01 | -2.57514 | 0.006606 | 0.047679 | Chr2 | 7905989 | 7911241 | 2991 |
| MsG0780038857.01 | -1.31774 | 0.006608 | 0.047683 | Chr7 | 54145960 | 54152166 | 3149 |
| MsG0180002506.01 | -1.51877 | 0.006612 | 0.047699 | Chr1 | 39350395 | 39358538 | 2776 |
| MsG0580028202.01 | 5.24254 | 0.006618 | 0.047729 | Chr5 | 76492225 | 76492768 | 190 |
| MsG0580025219.01 | -2.61235 | 0.006631 | 0.047804 | Chr5 | 15409885 | 15413934 | 1182 |
| novel.1969 | 2.396482 | 0.006632 | 0.047804 | Chr2 | 63286047 | 63301744 | 1964 |
| MsG0380015858.01 | 1.925206 | 0.006634 | 0.047808 | Chr3 | 75948480 | 75954454 | 1763 |
| MsG0780040952.01 | 1.361297 | 0.006636 | 0.047815 | Chr7 | 84727359 | 84730666 | 1388 |
| MsG0380015210.01 | -5.33117 | 0.006641 | 0.047836 | Chr3 | 66875642 | 66876334 | 693 |
| MsG0480018810.01 | -4.66396 | 0.006643 | 0.047836 | Chr4 | 10181559 | 10184453 | 2895 |
| MsG0280009734.01 | 1.144872 | 0.006656 | 0.04792 | Chr2 | 57318611 | 57318898 | 288 |
| novel.7758 | -1.38751 | 0.006658 | 0.04792 | Chr7 | 91930283 | 91932355 | 1260 |
| MsG0180000530.01 | -1.19266 | 0.006663 | 0.047942 | Chr1 | 7447119 | 7450625 | 1117 |
| MsG0580027776.01 | -1.81199 | 0.006664 | 0.047942 | Chr5 | 68336233 | 68336943 | 711 |
| novel.5277 | -5.33846 | 0.006668 | 0.047961 | Chr5 | 73771645 | 73772100 | 456 |
| MsG0880047107.01 | 3.47183 | 0.006675 | 0.047998 | Chr8 | 82020356 | 82023808 | 1812 |
| novel.8802 | -4.31335 | 0.006678 | 0.048009 | contig123end | 1909 | 2953 | 661 |
| novel.5358 | -2.19319 | 0.006686 | 0.048053 | Chr5 | 88498786 | 88501827 | 833 |
| novel.3547 | -1.01213 | 0.006689 | 0.048058 | Chr4 | 64592588 | 64599551 | 4736 |
| MsG0080048352.01 | -2.78307 | 0.006697 | 0.048101 | contig27end | 48693 | 49389 | 606 |
| MsG0880044997.01 | 5.616039 | 0.006709 | 0.048174 | Chr8 | 52564143 | 52566202 | 1237 |
| MsG0880044636.01 | -1.23278 | 0.006712 | 0.048181 | Chr8 | 46051511 | 46057130 | 2271 |
| MsG0080048382.01 | -1.66597 | 0.006724 | 0.04825 | contig294end | 15305 | 18903 | 825 |
| novel.838 | -1.75458 | 0.006725 | 0.04825 | Chr1 | 40260967 | 40263204 | 680 |
| novel.7522 | 4.680594 | 0.006735 | 0.048307 | Chr7 | 53545649 | 53546928 | 660 |
| MsG0480023280.01 | -3.52823 | 0.006736 | 0.048307 | Chr4 | 83156829 | 83157143 | 315 |
| novel.7229 | -1.9927 | 0.006737 | 0.048307 | Chr7 | 87394155 | 87395500 | 366 |
| MsG0880044411.01 | -1.25154 | 0.006748 | 0.048375 | Chr8 | 41588003 | 41589936 | 1053 |
| MsG0880042922.01 | -1.29217 | 0.006755 | 0.048407 | Chr8 | 16026341 | 16028371 | 2031 |
| MsG0580025465.01 | 4.746799 | 0.006763 | 0.048453 | Chr5 | 19139467 | 19139860 | 300 |
| MsG0680030393.01 | 1.104798 | 0.006764 | 0.048453 | Chr6 | 1633556 | 1637775 | 897 |
| novel.7209 | -3.38084 | 0.006769 | 0.048477 | Chr7 | 84113214 | 84117144 | 637 |
| MsG0580027659.01 | -1.51407 | 0.006773 | 0.048479 | Chr5 | 65383720 | 65386590 | 2871 |
| MsG0780037942.01 | 1.083714 | 0.006773 | 0.048479 | Chr7 | 36682317 | 36691750 | 2364 |
| MsG0180002458.01 | 1.265507 | 0.006774 | 0.048479 | Chr1 | 38639370 | 38644234 | 864 |
| MsG0580026857.01 | 5.513777 | 0.00678 | 0.048505 | Chr5 | 46531810 | 46533302 | 837 |
| MsG0280010629.01 | -3.06778 | 0.006781 | 0.048505 | Chr2 | 72551083 | 72556979 | 1931 |
| novel.7705 | 1.933772 | 0.006803 | 0.048651 | Chr7 | 84201416 | 84205824 | 1955 |
| MsG0380015505.01 | -5.29256 | 0.006811 | 0.048696 | Chr3 | 71171767 | 71176600 | 909 |
| MsG0180005036.01 | -1.97371 | 0.006814 | 0.048705 | Chr1 | 86044035 | 86051977 | 4284 |
| MsG0880047427.01 | 1.329119 | 0.006826 | 0.048781 | Chr8 | 86132389 | 86133405 | 544 |
| MsG0280009708.01 | 1.841199 | 0.006829 | 0.048787 | Chr2 | 56931756 | 56935989 | 1050 |
| MsG0280006743.01 | 1.396984 | 0.006842 | 0.048872 | Chr2 | 5736018 | 5736911 | 894 |
| MsG0580029433.01 | -1.63271 | 0.006851 | 0.04892 | Chr5 | 96921071 | 96926322 | 3306 |
| novel.31 | -2.17023 | 0.006854 | 0.048931 | Chr1 | 4166349 | 4169321 | 1703 |
| novel.783 | 3.13462 | 0.006865 | 0.048997 | Chr1 | 26801894 | 26802860 | 862 |
| MsG0180000440.01 | -2.0855 | 0.006871 | 0.04903 | Chr1 | 6052683 | 6055938 | 1602 |
| novel.5756 | 6.021698 | 0.006876 | 0.049053 | Chr6 | 46872711 | 46883433 | 505 |
| MsG0280010712.01 | 1.242929 | 0.006882 | 0.049087 | Chr2 | 73781307 | 73785862 | 569 |
| MsG0880045664.01 | 1.527298 | 0.006893 | 0.049149 | Chr8 | 62350570 | 62355274 | 2029 |
| MsG0880046267.01 | 1.093155 | 0.0069 | 0.049188 | Chr8 | 71312068 | 71316705 | 359 |
| MsG0180001463.01 | -3.83021 | 0.006911 | 0.049258 | Chr1 | 21731260 | 21732356 | 795 |
| novel.7952 | -1.45032 | 0.006929 | 0.049371 | Chr8 | 29951047 | 29955346 | 3050 |
| MsG0380015014.01 | 1.043309 | 0.006931 | 0.049371 | Chr3 | 63966284 | 63977303 | 1611 |
| novel.298 | 3.672736 | 0.006933 | 0.049376 | Chr1 | 55472796 | 55473917 | 835 |
| MsG0480019102.01 | 5.963736 | 0.00694 | 0.049413 | Chr4 | 14555666 | 14562313 | 3954 |
| MsG0180002749.01 | -2.21326 | 0.006944 | 0.049429 | Chr1 | 44388324 | 44392820 | 3816 |
| MsG0280011163.01 | -1.60474 | 0.006945 | 0.049429 | Chr2 | 80391028 | 80397824 | 3615 |
| novel.9363 | -2.30524 | 0.006955 | 0.049482 | contig649end | 2261 | 3618 | 826 |
| novel.2871 | 3.320454 | 0.006956 | 0.049482 | Chr3 | 38620984 | 38623399 | 731 |
| MsG0680033983.01 | 5.307429 | 0.006961 | 0.049506 | Chr6 | 78055568 | 78056361 | 576 |
| MsG0380017173.01 | -4.81821 | 0.006963 | 0.049506 | Chr3 | 92989380 | 92994606 | 2511 |
| MsG0280007908.01 | -2.23011 | 0.006968 | 0.04952 | Chr2 | 22782678 | 22784309 | 588 |
| MsG0480021293.01 | 1.144863 | 0.006968 | 0.04952 | Chr4 | 56385519 | 56388168 | 836 |
| MsG0180005917.01 | -1.39095 | 0.006987 | 0.049641 | Chr1 | 97512576 | 97517303 | 1291 |
| novel.5351 | -3.90019 | 0.006989 | 0.049644 | Chr5 | 87447228 | 87454272 | 513 |
| MsG0180005696.01 | -1.92329 | 0.006999 | 0.049693 | Chr1 | 95041763 | 95044312 | 1089 |
| novel.1506 | 1.570101 | 0.006999 | 0.049693 | Chr2 | 65887901 | 65888406 | 463 |
| MsG0780039064.01 | 1.168439 | 0.007002 | 0.049702 | Chr7 | 57419573 | 57423760 | 1464 |
| MsG0280007548.01 | -1.3782 | 0.007004 | 0.049706 | Chr2 | 17017060 | 17019220 | 441 |
| MsG0180001712.01 | -1.60596 | 0.007009 | 0.049725 | Chr1 | 25862916 | 25865604 | 825 |
| novel.2282 | -5.54933 | 0.007014 | 0.049748 | Chr3 | 27184500 | 27186814 | 2315 |
| MsG0580027641.01 | 2.138057 | 0.007023 | 0.049801 | Chr5 | 64966044 | 64970318 | 2521 |
| novel.1589 | 1.074295 | 0.007026 | 0.049803 | Chr2 | 79143104 | 79144829 | 839 |
| MsG0880047408.01 | -2.07166 | 0.007026 | 0.049803 | Chr8 | 85848759 | 85850514 | 1199 |
| novel.1387 | -2.15954 | 0.007034 | 0.049841 | Chr2 | 36659972 | 36662553 | 1150 |
| MsG0880047506.01 | -2.49184 | 0.007038 | 0.049859 | Chr8 | 87258774 | 87259202 | 429 |
| MsG0280006324.01 | 1.969862 | 0.007044 | 0.049893 | Chr2 | 438073 | 441073 | 408 |
| MsG0180004277.01 | -1.66731 | 0.007057 | 0.049969 | Chr1 | 75929462 | 75935228 | 2017 |
| novel.7505 | 1.345496 | 0.007058 | 0.049969 | Chr7 | 47969291 | 47974916 | 2310 |
| novel.6838 | -4.42979 | 0.00706 | 0.049969 | Chr7 | 12170725 | 12172627 | 1759 |
