# Supplementary material for: Overexpression of an NF-YC2 gene confers alkali tolerance to transgenic alfalfa (Medicago sativa L.)
Source: Front Plant Sci. 2022 Aug 5;13:960160. doi: 10.3389/fpls.2022.960160 (PMC9389336; doi:10.3389/fpls.2022.960160)
Supplement: Supplementary file 5 [file Table_5.docx]

| **Supplementary Table 5.Differently-expressed genes of the OE lines and WT plants under normal and salt stress.** | | | | |
| --- | --- | --- | --- | --- |
| gene_id | gene_chr | gene_start | gene_end | gene_length |
| MsG0180001988.01 | Chr1 | 30384576 | 30392007 | 2232 |
| MsG0180000201.01 | Chr1 | 2760862 | 2761116 | 255 |
| novel.8244 | Chr8 | 84825049 | 84826848 | 1800 |
| MsG0180004244.01 | Chr1 | 75492036 | 75492659 | 624 |
| MsG0580025323.01 | Chr5 | 16939463 | 16939720 | 258 |
| novel.5925 | Chr6 | 83775770 | 83780992 | 2008 |
| MsG0580025808.01 | Chr5 | 24521163 | 24526043 | 873 |
| MsG0380013128.01 | Chr3 | 30933916 | 30936289 | 757 |
| MsG0580028316.01 | Chr5 | 78243669 | 78249445 | 999 |
| MsG0380016317.01 | Chr3 | 81918119 | 81919183 | 621 |
| MsG0480021254.01 | Chr4 | 55940798 | 55954485 | 1584 |
| MsG0380014402.01 | Chr3 | 54194716 | 54194940 | 225 |
| novel.1214 | Chr2 | 7427578 | 7429721 | 2144 |
| MsG0680030648.01 | Chr6 | 6612783 | 6618921 | 1856 |
| MsG0680035646.01 | Chr6 | 1.1E+08 | 1.1E+08 | 570 |
| MsG0880047188.01 | Chr8 | 83105754 | 83106287 | 534 |
| MsG0180005889.01 | Chr1 | 97175452 | 97182551 | 2604 |
| MsG0480018241.01 | Chr4 | 2077386 | 2085603 | 2535 |
| novel.1065 | Chr1 | 90259957 | 90265273 | 3816 |
| novel.7845 | Chr8 | 9341711 | 9344104 | 650 |
| MsG0180000653.01 | Chr1 | 9262762 | 9264791 | 1551 |
| MsG0780039166.01 | Chr7 | 58963496 | 58965039 | 795 |
| novel.664 | Chr1 | 8878191 | 8886212 | 1104 |
| MsG0180003465.01 | Chr1 | 62667787 | 62668901 | 876 |
| MsG0780037414.01 | Chr7 | 25399657 | 25401254 | 1131 |
| MsG0680034725.01 | Chr6 | 93467241 | 93468590 | 498 |
