# Supplementary material for: Overexpression of an NF-YC2 gene confers alkali tolerance to transgenic alfalfa (Medicago sativa L.)
Source: Front Plant Sci. 2022 Aug 5;13:960160. doi: 10.3389/fpls.2022.960160 (PMC9389336; doi:10.3389/fpls.2022.960160)
Supplement: Supplementary file 7 [file Table_7.docx]

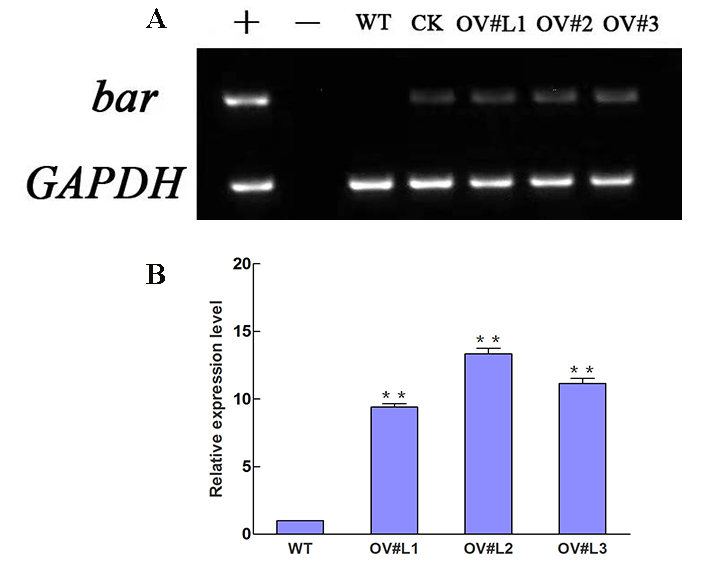


**Supplementary Figure 2:** Identification of transgenic overexpressing *MsNF-YC2*. (A) Bar gene analysis of overexpression *MsNF-YC2* plants. (B) Real-time PCR. Each value is the mean ± SE of three independent measurements. Each value is the mean ± SE of three biological replicates. Significant differences by different letters above the bars at the *P* < 0.05 and *P* < 0.01 level according to Duncan’s multiple range test
