# Supplementary material for: Overexpression of an NF-YC2 gene confers alkali tolerance to transgenic alfalfa (Medicago sativa L.)
Source: Front Plant Sci. 2022 Aug 5;13:960160. doi: 10.3389/fpls.2022.960160 (PMC9389336; doi:10.3389/fpls.2022.960160)
Supplement: Supplementary file 8 [file Table_8.docx]

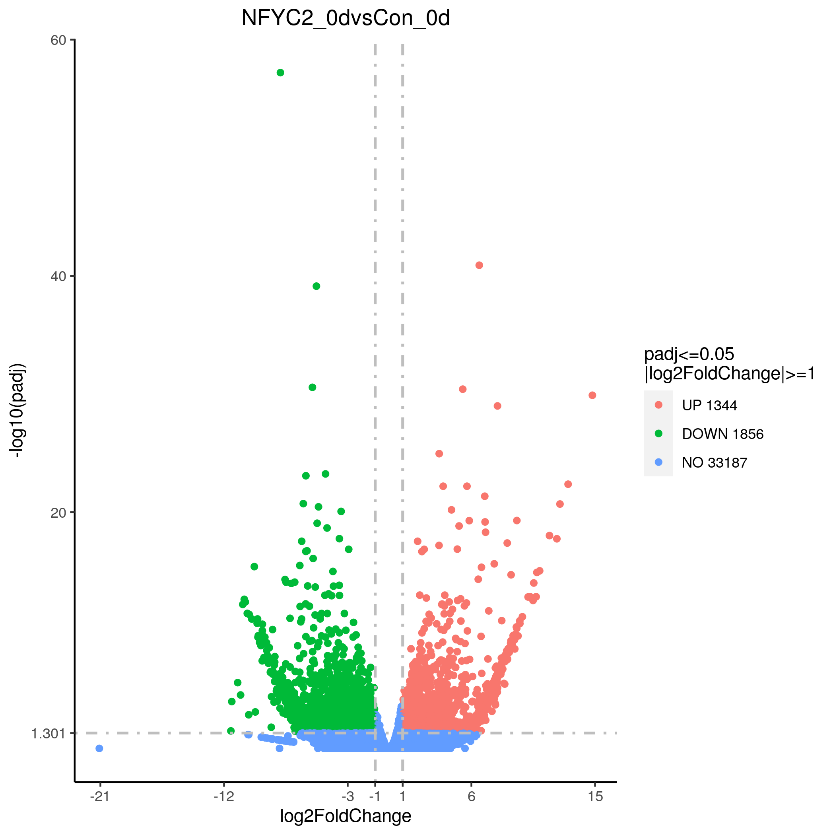


**Supplementary Figure 3. Volcano plot showed the numbers of DEGs identified in comparisons between pairs of libraries.**
