# Supplementary material for: Overexpression of an NF-YC2 gene confers alkali tolerance to transgenic alfalfa (Medicago sativa L.)
Source: Front Plant Sci. 2022 Aug 5;13:960160. doi: 10.3389/fpls.2022.960160 (PMC9389336; doi:10.3389/fpls.2022.960160)
Supplement: Supplementary file 10 [file Table_10.docx]

CW vs. AW CN vs. AN


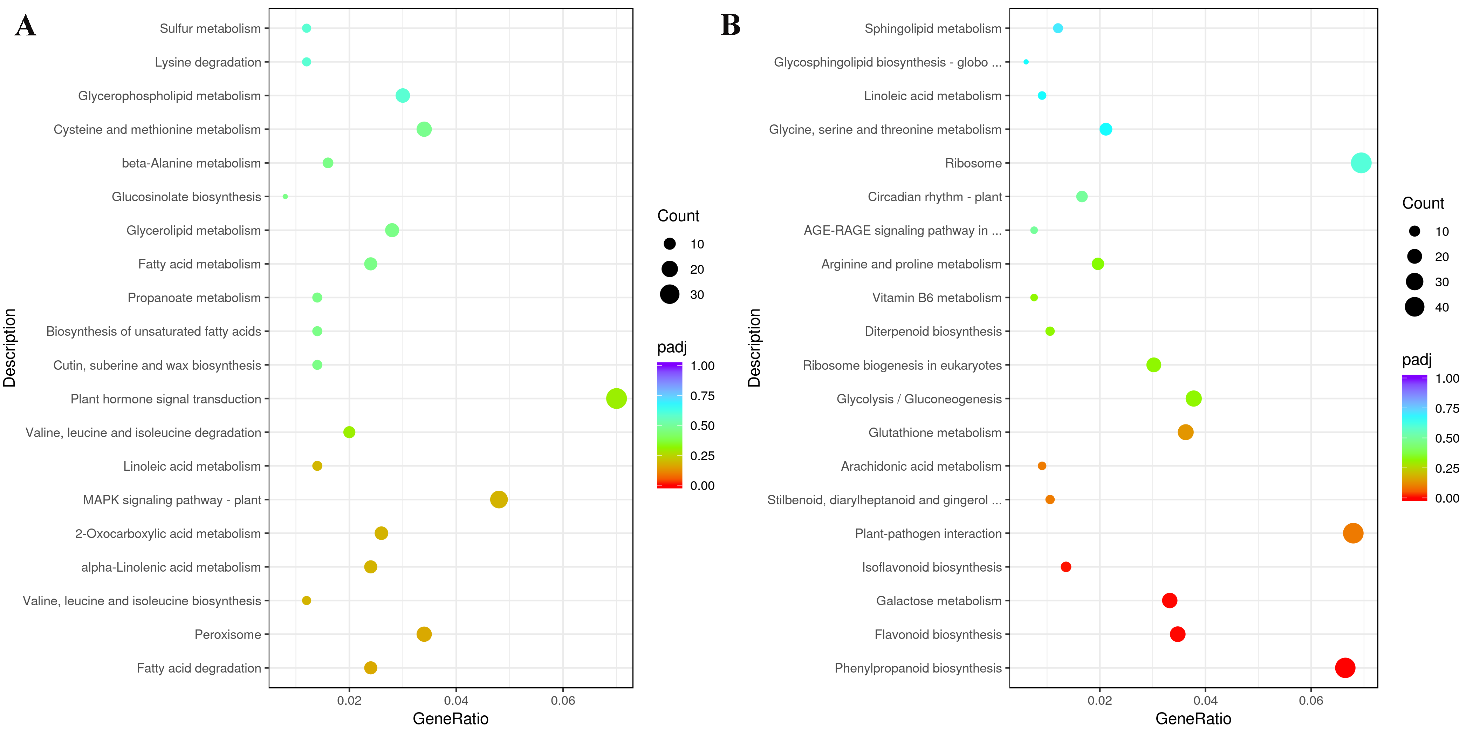


**Supplementary Figure 5.** Kyoto Encyclopedia of Genetics and Genomics (KEGG) database analyses of DEGs enriched in different biological pathways.
